# SmI_2_‐Catalyzed Coupling of Alkyl Housane Ketones and Alkenes in an Approach to Norbornanes

**DOI:** 10.1002/anie.202512018

**Published:** 2025-08-21

**Authors:** Debayan Roy, Jack I. Mansell, Giorgia Barison, Song Yu, Rocco Katavic, Ciro Romano, Nikolas Kaltsoyannis, David J. Procter

**Affiliations:** ^1^ Department of Chemistry University of Manchester Oxford Road Manchester M13 9PL UK; ^2^ Department of Chemical Sciences University of Padova Via Francesco Marzolo 1 Padova 35131 Italy

**Keywords:** Catalysis, Housane, Radicals, Samarium, Strain release

## Abstract

Cross‐coupling strategies involving strain release have gained significant recent attention for the construction of complex molecular frameworks, particularly in the context of preparing bioisosteres for medicinal chemistry. While the reactivity of cyclopropanes and bicyclo[1.1.0]butanes (BCBs) has been extensively studied, higher homologues are emerging as valuable substrates for synthesis. For example, methods for the fragmentation and coupling of bicyclo[2.1.0]pentane, or housane, ketones show promise but are currently limited in substrate scope. Here, we describe a mild, atom‐economical, samarium diiodide (Sml_2_)‐catalyzed fragmentation and coupling of alkyl and aryl housane ketones with alkenes that grants access to functionalized norbornane structural motifs, not easily accessible by classical cycloaddition approaches, and with considerable potential for further manipulation.

## Graphical abstract

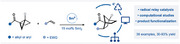

## Introduction

**Figure 1:**
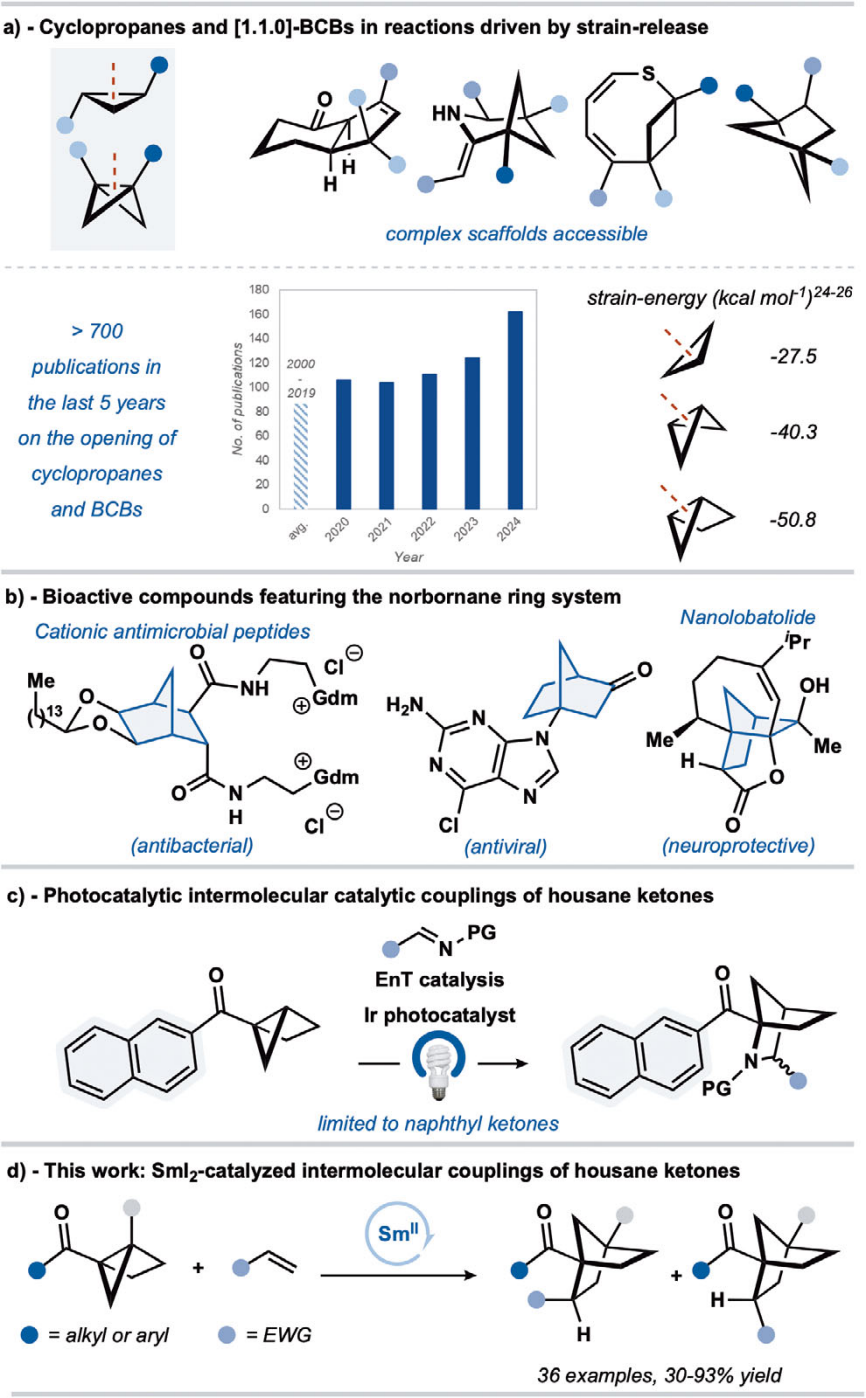
a) Selected products of strain‐release approaches using cyclopropanes and [1.1.0]‐bicyclobutanes (BCBs). b) Bioactive compounds featuring the norbornane ring system. c) Photocatalytic intermolecular couplings of housane ketones. d) Our strategy to access norbornane derivatives by SmI_2_‐catalyzed intermolecular coupling of housane ketones and alkenes. Gdm = guanidinium; PG = protecting group; EnT = energy transfer.

Synthetic methods that exploit the strain energy of small rings to drive the construction of complex molecular frameworks have attracted intense recent interest.^[^ ^1^, ^2^, ^3^, ^4^, ^5^, ^6^, ^7^, ^8^, ^9^, ^10^, ^11^, ^12^ ^]^ In particular, strained cyclopropanes and bicyclo[1.1.0]butanes (BCBs) have captured the attention of synthetic chemists due to their unique reactivity and the potential for generating reactive intermediates through fragmentation reactions (Figure 1a);^[^ ^7^, ^8^, ^9^, ^10^, ^11^, ^12^ ^]^ over 700 reports focusing on the opening of these ring systems have been published in the last 5 years. Recent advances in the catalysis of ring‐opening reactions of strained cycles, such as cyclopropanes and BCBs, have utilized metal catalysts and organocatalysts and embraced new mechanistic manifolds.^[^ ^1^, ^2^, ^3^, ^4^, ^5^, ^6^ ^]^ These additions to the synthetic toolbox are often selective, operate under mild conditions, and can be fine‐tuned to control reaction outcome. Thus, substrates containing strained ring systems are now indispensable intermediates in synthesis, allowing efficient access to an expanding range of functionalized molecular scaffolds (Figure 1a). The further development of efficient catalytic processes, driven by the rupture of strained rings, promises very different routes to new and established, important compound classes, thus accelerating discovery programs underpinned by synthesis and screening.

The norbornane (bicyclo[2.2.1]heptane) ring system is a classical molecular architecture,^[^ ^13^, ^14^, ^15^, ^16^, ^17^, ^18^, ^19^ ^]^ now gaining attention in medicinal chemistry due to its rigidity and three‐dimensionality; norbornanes are conformationally locked and present their substituents in a defined manner, thus making them valuable scaffolds for designing bioactive molecules.

For example, norbornane‐based cationic antimicrobial peptides have shown potent antibacterial activity for methicillin‐resistant *Staphylococcus aureus* (MRSA),^[^ ^18^ ^]^ and the natural product nanolobatolide has promising neuroprotective properties (Figure 1b).^[^ ^19^ ^]^ Traditional approaches to the norbornane ring system are dominated by Diels–Alder cycloadditions, followed by reduction of the residual alkene, and often require forcing reaction conditions.^[^ ^20^, ^21^ ^]^ Hence, complementary access to the norbornane ring system, through the catalytic ring‐opening reactions of strained ring precursors, would represent a valuable new synthetic strategy, delivering new, differently substituted norbornane architectures that would be otherwise difficult to obtain through traditional Diels–Alder approaches due to electronic mismatching.^[^ ^22^, ^23^ ^]^

The housane (bicyclo[2.1.0]pentane) ring system possesses strain energy higher than that of a cyclopropane ring and similar to that of the BCB system.^[^ ^24^, ^25^, ^26^ ^]^ Thus, housanes are attractive potential substrates for strain‐release strategies. However, there are currently few synthetic approaches that exploit housane substrates.^[^ ^27^ ^]^ Of particular note, Brown and coworkers recently exploited energy transfer (EnT) catalysis to fragment naphthyl housane ketones and generate aza‐norbornane products through couplings with imines (Figure 1c).^[^ ^28^ ^]^ With a focus on constructing aza‐norbornanes, couplings with alkenes involved aza‐housanes, with the presence of a naphthyl ring system essential for efficient EnT.

Here, we describe a mild, atom‐economical, samarium diiodide (Sml_2_)‐catalyzed fragmentation and coupling of alkyl and aryl housane ketones with alkenes that enables access to functionalized norbornane structural motifs with considerable potential for further manipulation (Figure 1d). The coupling proceeds through a radical relay mechanism triggered by single‐electron transfer (SET) from the SmI_2_ catalyst.^[^ ^29^, ^30^, ^31^, ^32^, ^33^, ^34^, ^35^, ^36^, ^37^, ^38^, ^39^, ^40^, ^41^, ^42^, ^43^, ^44^ ^]^ Of note, the use of Sml_2_ as a catalyst lends the approach significant substrate scope, in that typically unreactive *alkyl* housane ketones are excellent substrates.

## Results and Discussion

### Reaction Optimization and Tolerance

We began by examining the coupling of housane ketone **1a** and acrylonitrile, using SmI_2_ (25 mol%), at −10 °C (Table 1, entry 1). Pleasingly, the targeted norbornane **2a** was formed in quantitative yield with modest diastereocontrol (2.7:1, *exo*/*endo*). Further screening of conditions showed that a lower catalyst loading (15 mol%) could be employed in this system without a drop in yield (entry 2). Three‐day‐old SmI_2_ performed reasonably well under the reaction conditions, with only a moderate decrease in yield observed (entry 3). Control experiments (entries 4–7), highlighted the essential role of Sm(II), as removal or replacement of the SmI_2_ catalyst with SmI_3_ failed to lead to product formation. The decrease in the amount of recovered starting material observed in the presence of Sm(III) suggests that a Lewis acid‐mediated decomposition process is out‐competed by the desired coupling.

**Table 1:** Reaction optimization and control experiments.

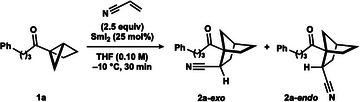		
Entry	Deviation from above	^1^H NMR yield (%)
1	None	>99 (2.7:1 d.r.)
2	SmI_2_ (15 mol%)	>99 (2.8:1 d.r.)
3	3 days old SmI_2_ (15 mol%)	61 (2.6:1 d.r.)
4	SmI_2_ (15 mol%) + no acrylonitrile	n.d. (50% rsm)
5	No SmI_2_	n.d. (>99% rsm)
6	SmI_3_ (15 mol%) instead of SmI_2_	n.d. (80% rsm)
7	SmI_3_ (15 mol%) + no acrylonitrile	n.d. (75% rsm)

Reaction conditions: **1a** (0.1 mmol, 1 equiv), acrylonitrile (2.5 equiv), SmI_2_ (25 mol%), in THF (0.1 M) under N_2_ (−10 °C, 30 min). The yield and diastereoisomeric ratio were determined by ^1^H qNMR analysis of the crude reaction mixture with CH_2_Br_2_ as internal standard (0.05 mmol). rsm = recovered starting material.

**Scheme 1:**
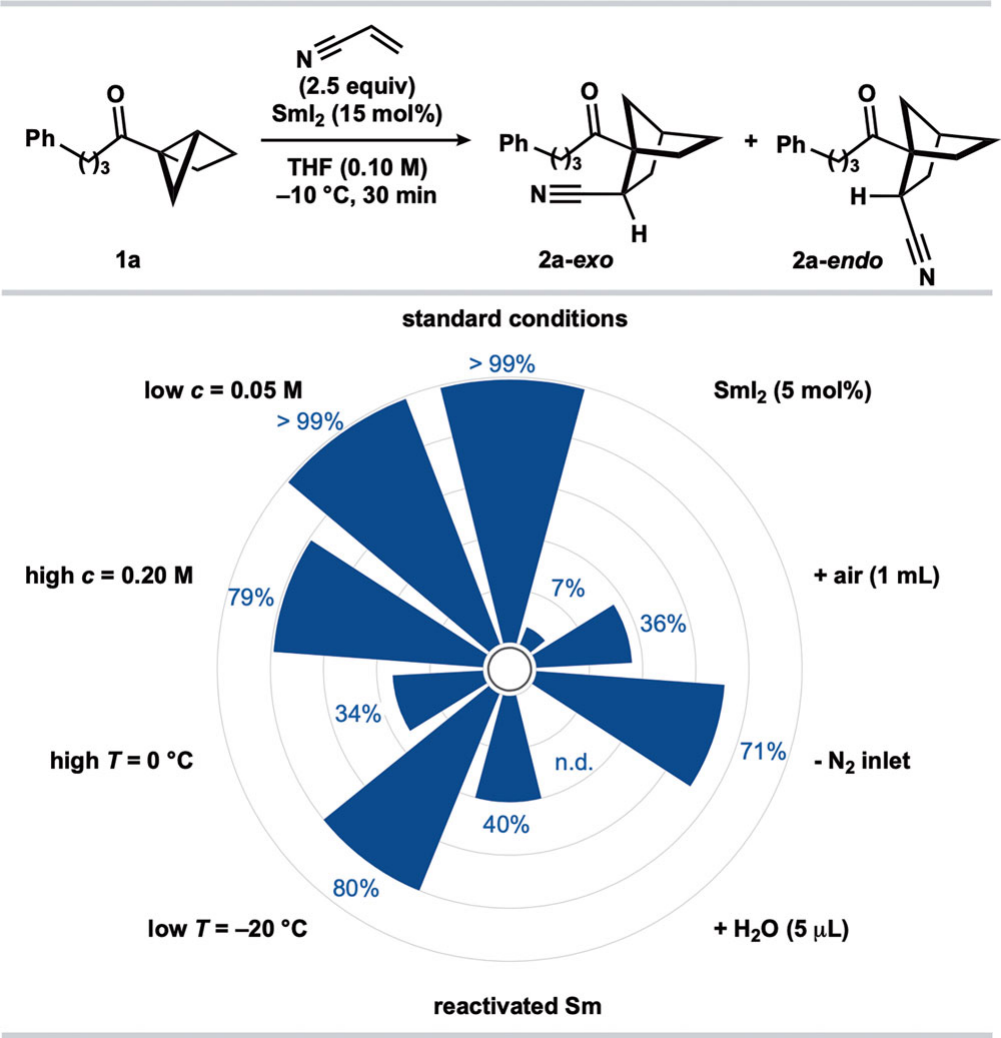
Reaction tolerance screening. See Supporting Information for experimental details.

Next, the reaction tolerance was evaluated using the optimal conditions (Scheme 1 and Table 1, entry 2). Higher reaction concentration gave a moderate decrease in yield of **2a**; however, lowering the concentration had no impact. The use of higher temperature led to lower product yield. While lower temperature proved detrimental to conversion; extended reaction times would likely improve conversion at −20 °C.

Batch‐to‐batch inconsistencies in the quality of Sm metal used to prepare SmI_2_ can affect its reactivity.^[^ ^45^ ^]^ To evaluate the impact that this would have on catalysis, an old batch of Sm metal was reactivated by heating the metal under vacuum with vigorous agitation and storage under an inert atmosphere prior to the preparation of SmI_2_.^[^ ^45^ ^]^ While SmI_2_ prepared from this reactivated metal was a competent catalyst, product yield was diminished, thus highlighting the importance of SmI_2_ quality when working in catalysis. The addition of a proton source, H_2_O (5 µL), led to recovery of starting material with no product detected. We next probed the sensitivity of catalysis to oxygen. Removal of the N_2_ inlet to the reaction vessel led to a moderate decrease in yield; the addition of air (1 mL) led to a further decrease in product formation. Finally, a lower catalyst loading (5 mol%) was found to return only traces of product.

### Reaction Scope

**Scheme 2:**
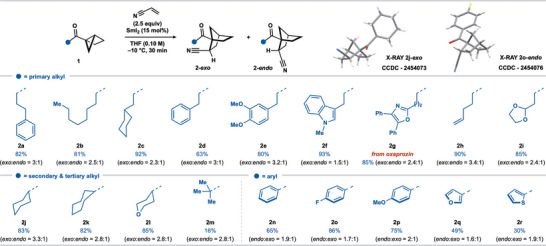
Scope of the SmI_2_‐catalyzed coupling of alkyl and aryl housane ketones with acrylonitrile. Reaction conditions: **1** (0.2 mmol, 1 equiv), acrylonitrile (2.5 equiv), SmI_2_ (15 mol%), in THF (0.1 M) under N_2_ (−10 °C, 30 min). The isolated yield is reported unless otherwise stated. The diastereoisomeric ratio was determined by ^1^H qNMR analysis of the crude reaction mixture with CH_2_Br_2_ as an internal standard (0.05 mmol).

We evaluated the scope of the SmI_2_‐catalyzed coupling of housane ketones with alkenes using the optimal conditions (Table 1, entry 2). Regarding the carbonyl substituent, an array of housane ketones was subjected to the coupling with acrylonitrile (Scheme 2). Housane ketones bearing primary alkyl substituents afforded norbornane ketones **2a−i** in good to excellent yield. More hindered housane ketones bearing secondary alkyl substituents were also competent substrates for the catalytic coupling providing **2j−l** in good yield. However, a tertiary butyl ketone substrate gave **2m** in a much lower yield.

The reaction is not limited to alkyl ketones; aryl housane ketones afforded the norbornane products in good yields (**2n–p**). Aryl groups bearing electron‐withdrawing (**2o**) and electron‐donating (**2p**) substituents were compatible, as were heteroaryl groups; the corresponding norbornane ketones **2q** and **2r** were obtained in moderate yield. The catalytic coupling showed good functional group compatibility in tolerating ether (**2e**, **2l**, **2p**), alkenyl (**2h**), acetal (**2i**), fluoro (**2o**), indolyl (**2f**), oxazolyl (**2g**), furanyl (**2q**), and thienyl (**2r**) motifs in the housane ketone substrates. A housane derived from oxaprozin, a nonsteroidal anti‐inflammatory drug (NSAID), proved an effective partner furnishing norbornane ketone product **2g** in 85% yield. Of note, a substrate bearing a terminal alkene underwent smooth coupling to give **2h** despite the potential for the putative ketyl radical intermediate (see Scheme 5)^[^ ^46^ ^]^ to undergo 5‐*exo‐trig* cyclization.^[^ ^47^ ^]^

In all cases, the couplings proceeded with complete regiocontrol and with moderate diastereocontrol—norbornanes were obtained in approximately 3:1 dr. Products can be epimerized to give single diastereosiomers. For example, the crude mixture of diastereoisomers of **2a** could be directly converted to a single *exo*‐diastereoisomer of the corresponding norbornane ketoacid in (81% overall yield) upon basic epimerization/nitrile hydrolysis (vide infra). Of note, *exo‐*diastereoisomers were the major products when employing alkyl housane ketones (products **2a**−**m**), while *endo*‐diastereoisomers were formed in excess from the couplings of aryl housane ketones (**2n**−**r**). The relative stereochemistry of the major diastereoisomers of **2j‐*exo*** and **2o**‐***endo*** was confirmed by X‐ray crystallographic analysis.^[^ ^48^ ^]^

**Scheme 3:**
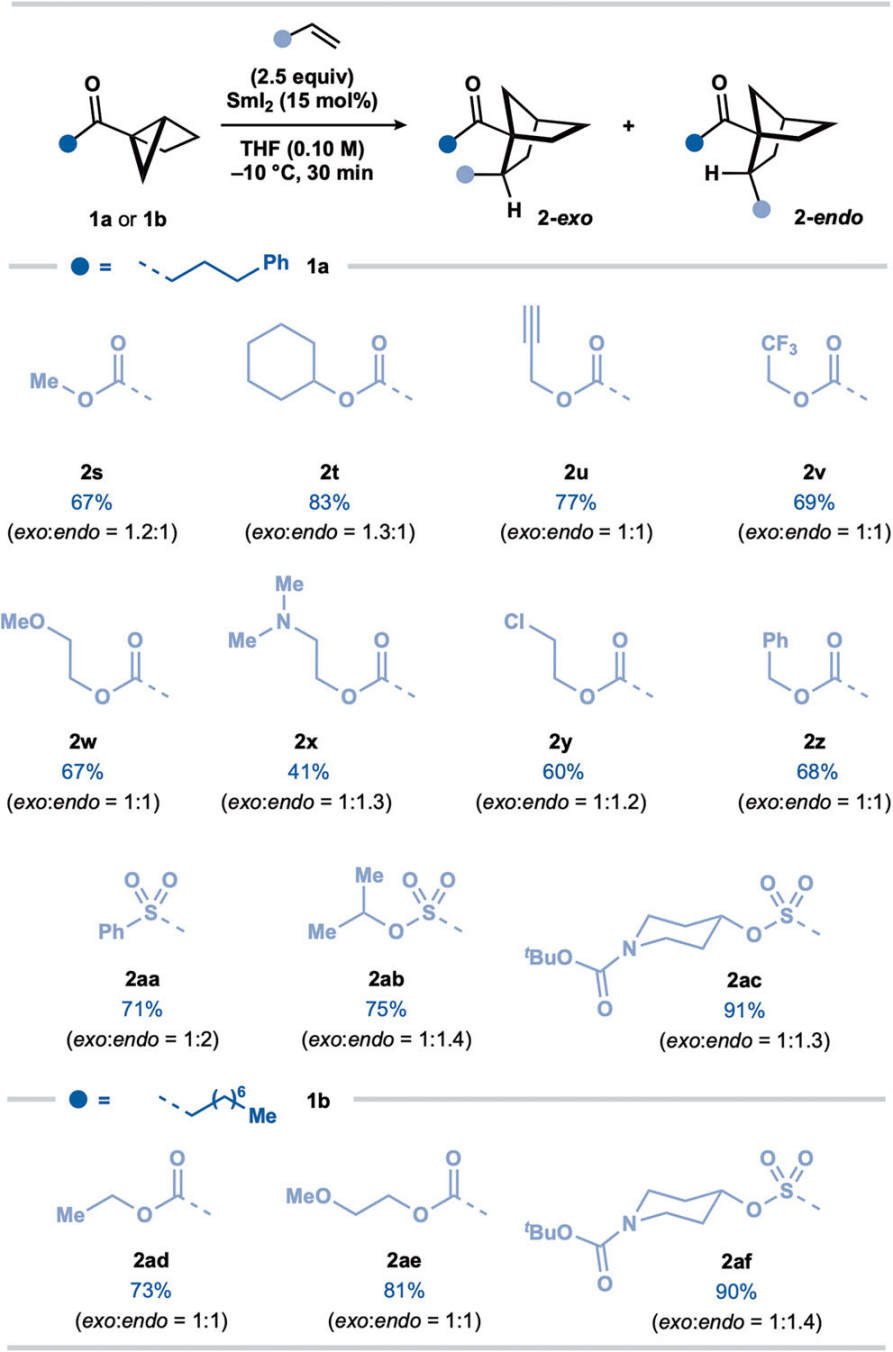
Scope of the SmI_2_‐catalyzed coupling of alkyl housane ketones **1a** and **1b** with electron‐deficient alkenes. Reaction conditions: **1a,b** (0.2 mmol, 1 equiv), alkene (2.5 equiv), SmI_2_ (15 mol%), in THF (0.1 M) under N_2_ (−10 °C, 30 min). The isolated yield is reported unless otherwise stated. The diastereoisomeric ratio was determined by ^1^H qNMR analysis of the crude reaction mixture with CH_2_Br_2_ as an internal standard (0.05 mmol).

We next sought to engage alternative electron‐deficient alkenes in the coupling with alkyl housane ketone **1a** (Scheme 3). In addition to acrylonitrile, acrylates proved competent partners in the SmI_2_‐catalyzed coupling, delivering norbornane ketones **2s–z** in moderate to good yield. The successfully engaged acrylate partners highlight the compatibility of the process with alkynyl (**2u**), trifluoromethyl (**2v**), ether (**2w**), amino (**2x**), and chloro (**2y**) motifs. Vinyl sulfone and vinyl sulfonate esters also engaged as effective coupling partners, forming **2aa–2ac** in good yield. Finally, alternative alkyl housane ketone **1b** underwent smooth coupling with two acrylates and a vinyl sulfonate ester to give norbornanes **2ad–2af** in high yield. While the couplings with acrylates, vinyl sulfones, and vinyl sulfonates proceeded with little diastereocontrol, products were amenable to epimerization to give single diastereoisomers (vide infra).

**Scheme 4:**
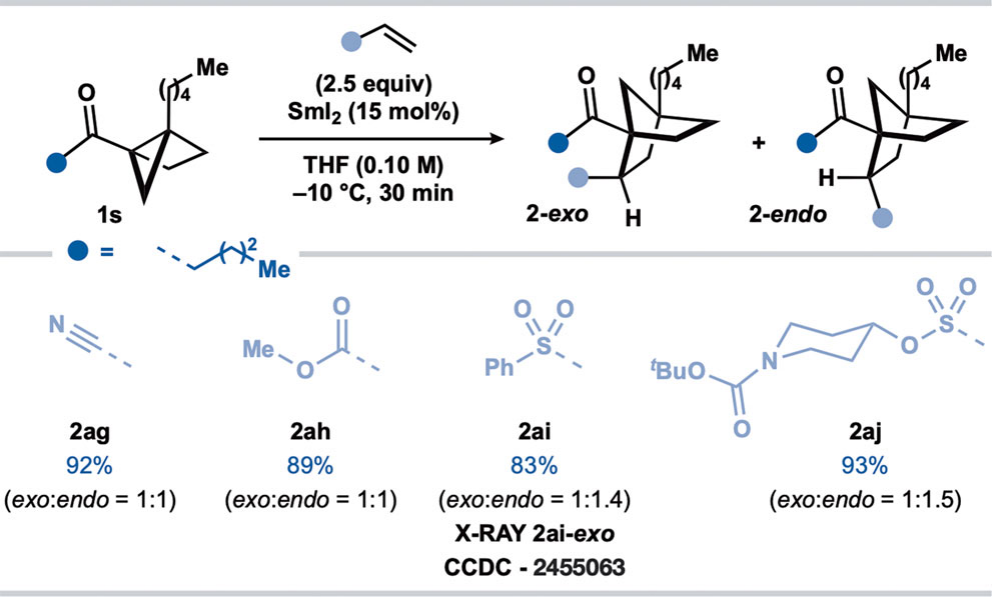
Scope of the SmI_2_‐catalyzed coupling of bridgehead‐substituted **1s** with electron‐deficient alkenes. Reaction conditions: **1s** (0.2 mmol, 1 equiv), alkene (2.5 equiv), SmI_2_ (15 mol%), in THF (0.1 M) under N_2_ (−10 °C, 30 min). The isolated yield is reported unless otherwise stated. The diastereoisomeric ratio was determined by ^1^H qNMR analysis of the crude reaction mixture with CH_2_Br_2_ as an internal standard (0.05 mmol).

To further probe the scope of the reaction, bridgehead‐substituted housane ketone **1s** was prepared and coupled under our standard catalytic conditions to furnish norbornane products **2ag–2aj** in excellent yield (Scheme 4), indicating that tertiary alkyl radicals are competent intermediates in the intermolecular couplings. The structure of **2ai‐*exo*** was confirmed by X‐ray crystallographic analysis.^[^ ^48^ ^]^

### Computational Studies

**Scheme 5:**
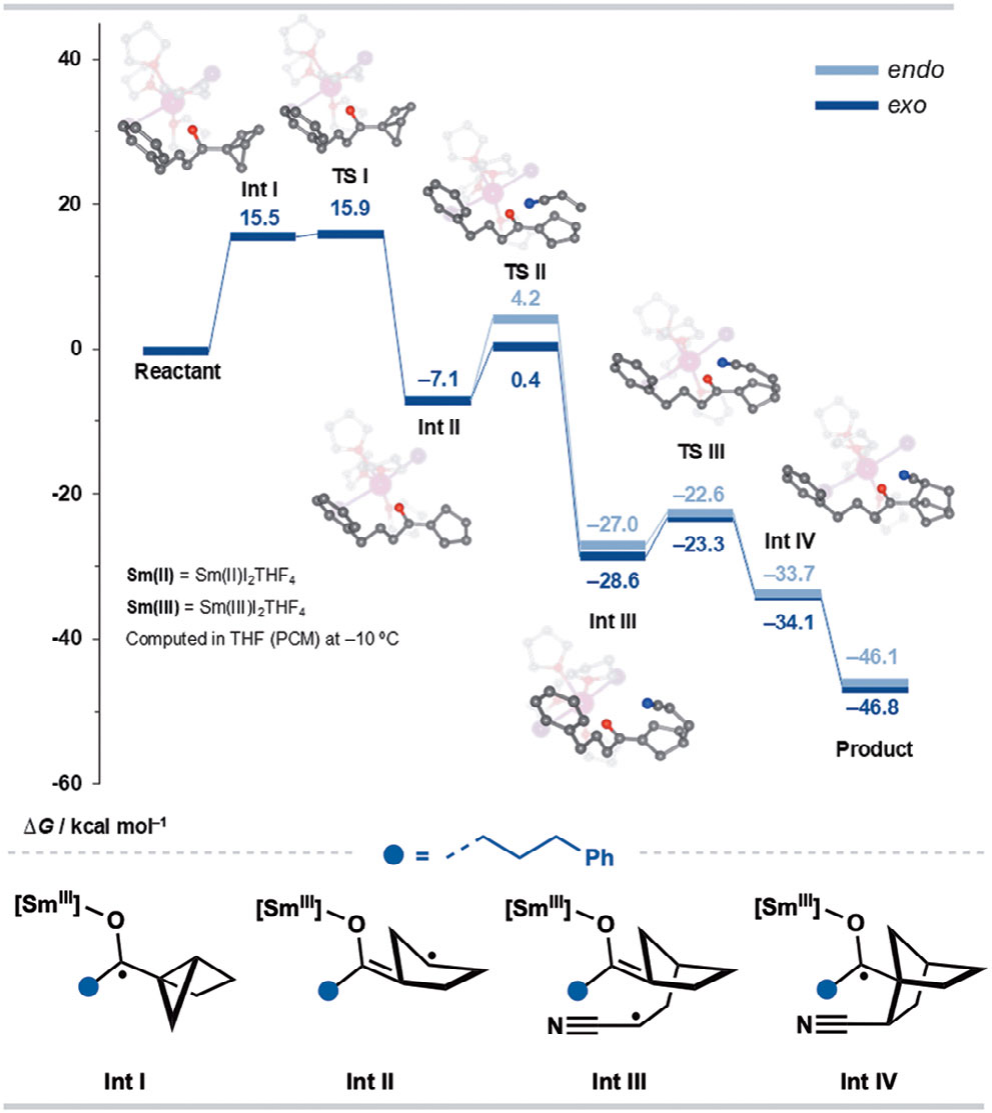
Gibbs free energy profiles (∆*G*, in kcal mol^−1^) for the SmI_2_‐catalyzed coupling of **1a** with acrylonitrile. For clarity, only structures leading to the major *exo*‐diastereoisomer are shown. Geometry optimizations were carried out at the PBE0‐D3(BJ) level using Stuttgart‐Köln ECPs and associated valence basis sets for Sm and I, with cc‐pVDZ basis sets for the remaining elements. Subsequent single‐point energy calculations were performed at the THF(PCM)‐PBE0‐D3(BJ) level, incorporating the DKH Hamiltonian, with the SARC basis set for Sm, the Jorge basis set for I and cc‐pVTZ basis sets for the remaining elements.

Computational studies (PBE0‐D3(BJ) level) were performed to probe the mechanism of the SmI_2_‐catalyzed coupling between model housane ketone **1a** and acrylonitrile (see Supporting Information for computational details).^[^ ^7^, ^10^, ^30^, ^49^, ^50^ ^]^ The computed potential energy surface (Scheme 5) shows that the ketone undergoes an inner‐sphere SET with an activation energy of 15.5 kcal mol^−1^, leading to the formation of a ketyl radical intermediate **Int I**. Then, **Int I** undergoes facile fragmentation, driven by the relief of strain in the housane, via transition state **TS I** (15.9 kcal mol^−1^), yielding **Int II**. Subsequently, **Int II** is trapped by acrylonitrile through transition state **TS II**, with barrier energies of 4.2 and 0.4 kcal mol^−1^ for the *endo* and *exo* pathways, respectively. This step is followed by the rebound, ring‐closing reaction of the radical adduct intermediate **Int III** to give Sm(III)‐bound ketyl radical **Int IV**, which then undergoes back electron transfer, regenerating Sm(II) and yielding the final products. The computational results suggest that the overall barrier for housane ketone coupling is only slightly higher than that calculated for the coupling of highly strained bicyclo[1.1.0]butane (BCB) ketones.^[^ ^49^ ^]^

### Conformational Analysis

**Scheme 6:**
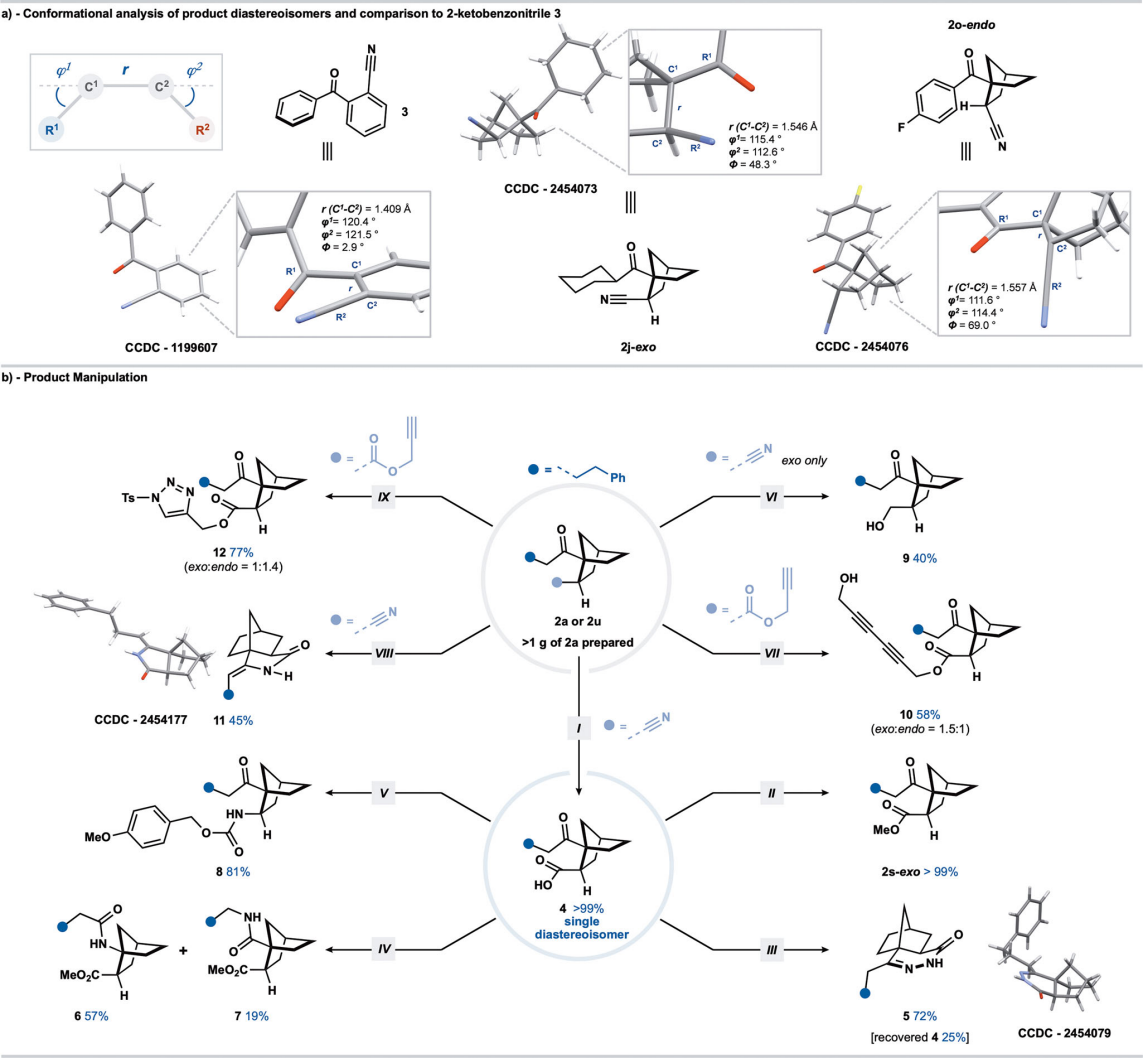
a) Exit vector analysis of representative product diastereoisomers. b) Scale‐up SmI_2_‐catalyzed alkene insertion into alkyl housane ketones and norbornane product manipulation. Reaction conditions: **I** – **2a** (1.0 equiv), KOH (9.0 equiv), EtOH:H_2_O (1:1, 0.25 M), Δ. **II** – **4** (1.0 eq), MeI (1.6 equiv), K_2_CO_3_ (2.0 equiv), DMF (0.2 M). **III** – step **II**, then, N_2_H_4_•H_2_O (5.0 equiv), DME (0.2 M), Δ. **IV** – step **II**, then, NH_2_OH•HCl (8.0 equiv), NH_4_OAC (8.0 equiv), EtOH:H_2_O (1:1, 0.08 M), Δ, then I_2_ (50 mol%), MeCN (0.1 M), Δ. **V** – **4** (1.0 equiv), DPPA (1.5 equiv), Et_3_N (1.5 equiv), PhMe (0.1 M), Δ, then 4‐OMe‐benzyl alcohol (3.0 equiv), PhMe (0.1 M), Δ. **VI** – Performed on exo isomer only; **2a** (1.0 equiv), [RuCl_2_(*p*‐cymene)]_2_ (1.5 mol%), (CH_2_O)*_n_* (9.0 equiv), PhMe:H_2_O (1:1, 0.25 M), Δ. **VII** – **2u** (1.2 equiv), 3‐bromoprop‐2‐yn‐1‐ol (1.0 equiv), CuBr (10 mol%), *^n^* BuNH_2_ (1.2 equiv), Na‐ascorbate (1.0 eq), EtOH (0.1 M). **VIII** – **2a** (1.0 equiv), *^t^* BuOK (2.0 equiv), *^t^* BuOH (0.5 M), THF (0.1 M). Isolated yield is based on conversion of the *exo*‐isomer. **XI** – **2u** (1.0 equiv), TsN_3_ (1.0 equiv), CuTc (10 mol%), PhMe (0.1 M).

The use of rigid, sp^3^‐rich carbocyclic motifs as replacements for benzene rings in biologically active molecules is now an established strategy in drug discovery.^[^ ^51^, ^52^ ^]^ While recent work by Grygorenko and coworkers compared 1,4‐disubstituted norbornanes to *para*‐substituted benzenes,^[^ ^53^ ^]^ other substitution patterns on the norbornane ring system have yet to be considered as rigid alternatives to substituted benzenes with very different exit vectors. Motivated by this, we compared the X‐ray stuctures of **2j‐*exo*** and **2o‐*endo***—representative products of our catalytic process—to that of a known 2‐ketobenzonitrile **3** (Scheme 6a).^[^ ^48^, ^54^ ^]^ As expected, the C1─C2 bond length (*r*) was longer (1.557 and 1.546 Å) for both norbornane diastereoisomers, when compared with the analogous bond in **3** (1.409 Å). Furthermore, the *φ*1 and *φ*2 bond angles in both diastereoisomers were slightly smaller than in **3** (120.4° and 121.5°, respectively). Also, as expected, the torsion angle *ϕ* (the angle between the planes defined by the R^1^−C^1^−C^2^ and C^1^−C^2^−R^2^ groups) is very different for the three compounds; *ϕ* = 2.9° in **3**, while much larger angles, *ϕ* = 69.0° and *ϕ* = 48.3°, were measured for the norbornanes **2o‐*endo*** and **2j‐*exo***.

### Manipulation of Norbornane Ketones

The SmI_2_‐catalyzed coupling of housane **1a** with acrylonitrile was carried out on a larger scale (5.0 mmol) to give 1.07 g of **2a** (79% yield, 2.7:1 mixture of *exo*:*endo* diastereoisomers) (Scheme 6b). Epimerization of the products of catalysis allows convenient access to single diastereoisomers. For example, straightforward hydrolysis of the nitrile motif in **2a** allowed access to the carboxylic acid **4** in quantitative yield and as a single *exo* diastereoisomer. Diastereomerically pure acid **4** was converted to the corresponding methyl ester **2s‐*exo*** that was then converted to either the dihydropyridazinone **5**, whose structure was confirmed by X‐ray crystallographic analysis,^[^ ^48^ ^]^ or to amide regioisomers **6** and **7** after Beckmann rearrangement. The diastereoisomerically pure acid **4** was also subjected to a Curtius rearrangement to generate *exo*‐carbamate **8**. The *exo* isomer of **2a** underwent selective nitrile reduction, under transfer hydrogenation conditions, to generate primary alcohol **9**. Norbornane **2u** bearing a propargyl ester group was prepared on a 1.0 mmol scale and underwent Cadiot–Chodkiewicz cross‐coupling, to afford diyne **10**,^[^ ^55^ ^]^ and copper‐catalyzed azide‐alkyne cycloaddition to generate triazole **12**.^[^ ^56^ ^]^ Finally, a diastereoisomeric mixture of **2a** was converted to cyclic enamide **11** upon treatment with base. The structure of **11** was confirmed by X‐ray crystallographic analysis.^[^ ^48^ ^]^

## Conclusion

Samarium diiodide (Sml_2_) is an effective catalyst for the mild, atom‐economical coupling of bicyclo[2.1.0]pentane, or housane, ketones, and electron‐deficient alkenes to give functionalized norbornane structural motifs not easily accessible by classical cycloaddition approaches. In addition to aryl‐substituted housane ketones, harder‐to‐reduce alkyl housane ketones are also excellent substrates for the radical cross‐coupling. The products of catalysis have considerable potential for further manipulation to give diastereoisomerically pure norbornane derivatives and norbornane‐fused heterocycles. The catalytic coupling constitutes a promising platform for the construction of sp^3^‐rich, conformationally defined, 3D architectures for use, for example, in medicinal chemistry.

## Supporting Information

Experimental procedures, characterization data, computational results, X‐ray data, and NMR spectra for all new compounds. The authors have cited additional references within the Supporting Information.

## Conflict of Interests

The authors declare no conflict of interest.

## Supporting information



Supporting Information

## Data Availability

The data that support the findings of this study are available in the Supporting Information of this article.
