# Supplementary material for: SmI2‐Catalyzed Coupling of Alkyl Housane Ketones and Alkenes in an Approach to Norbornanes
Source: Angew Chem Int Ed Engl. 2025 Aug 21;64(39):e202512018. doi: 10.1002/anie.202512018 (PMC12455418; doi:10.1002/anie.202512018)

SmI_2-Catalyzed Coupling of Alkyl Housane Ketones and Alkenes in an Approach to Norbornanes

Debayan Roy[a], Jack I. Mansell[a], Giorgia Barison[a][b], Song Yu[a], Rocco Katavic[a], Ciro Romano[a], Nikolas Kaltsoyannis[a], and David J. Procter[a]*

[a] – Department of Chemistry, The University of Manchester, Oxford Road, Manchester, M13 9PL (UK).

[b] – Department of Chemical Sciences, University of Padova, Via Francesco Marzolo 1, 35131 Padova, Italy

*email: david.j.procter@manchester.ac.uk

Supporting information

Table of Contents

1. Figure 1A Graph Page 4

2. General information Page 4

3. Abbreviations Page 6

4. Preparation of reagents Page 8

5. Starting material preparation Page 13

5.1 General Procedures for making housane ketones **(GP 1)** Page 13

5.1.1 General Procedure 2 **(GP 2)** Page 13

5.1.2 General Procedure 3 **(GP 3)** Page 37

5.1.3 Synthesis of 1-(bicyclo[2.1.0]pentan-1-yl)-2,2-dimethylpropan-1-one **(1m)** Page 53

5.1.4 Synthesis of 1-(4-pentylbicyclo[2.1.0]pentan-1-yl)pentan-1-one **(1s)** Page 56

5.2 Preparation of electron-deficient alkenes Page 60

6. SmI_2-catalyzed cross-coupling of housane ketones and alkynes/alkenes Page 63

6.1 Tolerance Experiments Page 63

6.2 Isolation of a Trace Byproduct Page 64

6.3 General procedure for SmI_2-catalyzed intermolecular coupling reactions Page 65

6.3.1 General procedure 4 **(GP 4)** Page 65

7. Manipulation of norbornane products of SmI_2 catalysis Page 107

7.1 Larger scale SmI_2-catalyzed coupling of alkyl housane ketones and alkenes Page 107

7.2 1-(4-Phenylbutanoyl)bicyclo[2.2.1]heptane-2-carboxylic acid **(4)** Page 108

7.3 Methyl 1-(4-phenylbutanoyl)bicyclo[2.2.1]heptane-2-carboxylate **(2s-*exo*)** Page 109

7.4 4-(3-Phenylpropyl)-6,7,8,8a-tetrahydro-2*H*-4a,7-methanophthalazin-1(5*H*)-one **(5)** Page 110

7.5 Methyl 1-(4-phenylbutanamido)bicyclo[2.2.1]heptane-2-carboxylate **(6)** and methyl 1-((3- phenylpropyl)carbamoyl)bicyclo[2.2.1]heptane-2-carboxylate **(7)** Page 111

7.6 2-(4-Methoxyphenyl)-*N*-(1-(4-phenylbutanoyl)bicyclo[2.2.1]heptan-2-yl)acetamide **(8)** Page 113

7.7 1-(2-(Hydroxymethyl)bicyclo[2.2.1]heptan-1-yl)-4-phenylbutan-1-one **(9)** Page 114

7.8 6-Hydroxyhexa-2,4-diyn-1-yl-1-(4-phenylbutanoyl)bicyclo[2.2.1]heptane-2-carboxylate **(10)** Page 115

7.9 3-(3-Phenylpropylidene)hexahydro-3a,6-methanoisoindol-1(4*H*)-one **(11)** Page 117

7.10 (1-Tosyl-1*H*-1,2,3-triazol-4-yl)methyl-1-(4-phenylbutanoyl)bicyclo[2.2.1]heptane-2- carboxylate **(12)** Page 118

8. X-ray diffraction analysis data Page 120

8.1 Data for **2j** [CCDC 2454073] Page 120

8.2 Data for **2o** [CCDC 2454076] Page 121

8.3 Data for **2ai** [CCDC 2455063] Page 122

8.4 Data for **5** [CCDC 2454079] Page 123

8.5 Data for **11** [CCDC 2454177] Page 124

9. Computational studies Page 125

9.1 Computational details Page 125

9.2 Coordinates and energies Page 125

10. References Page 148

11. ^{1}H, $^{13}C\{H\}$, and ^{19}F NMR Spectra Page 150

1. Figure 1A Graph

A search was made using the Scopus database [accessed 3-12-2024] with the following search terms “cyclopropane” OR “bicyclobutane” OR “bcb” OR “bicyclo[1.1.0]butane” OR “[1.1.0]-bcb” AND “ring AND opening” OR “strain AND release” to generate the data used to create the graph in Figure 1A.

2. General information

All reactions were carried out in oven-dried glassware and cleaned using base ($KOH_{(aq)}$, ^{i}PrOH) and acid ($HCl_{(aq)}$) baths. All reactions were considered air/moisture sensitive and performed under a dry nitrogen atmosphere unless otherwise noted. Reactions were magnetically stirred. All reported reaction temperatures correspond to external bath temperatures. Room temperature was approximately 23°C. Yields refer to chromatographically and spectroscopically (^{1}H and ^{13}C NMR) homogeneous materials, unless otherwise stated. Reagents were purchased at the highest commercial quality and used without further purification unless otherwise stated. Diiodoethane was dissolved in Et_2O and washed with saturated aqueous sodium thiosulfate, then dried under vacuum (rt, 24 h, dark) before use. All organolithium reagents were titrated against diphenylacetic acid (recrystallized from PhMe) in THF prior to use. Triethylamine (Et_3N) was distilled over CaH_2 prior to use in titrations of SmI_2 and was stored under an inert atmosphere.

^{1}H, ^{13}C, and ^{19}F NMR spectra were recorded on Bruker Avance III 400 MHz and 500 MHz instruments fitted with 5 mm Prodigy cryoprobes. All NMR spectra were processed using Mestrenova© NMR software. Chemical shifts are reported in parts per million (ppm). ^{1}H and ^{13}C, NMR spectra were referenced to the residual solvent peak (Tetramethylsilane (TMS): ^{1}H = 0.00 ppm, $CHCl_3$: ^{1}H = 7.26 ppm, ^{13}C = 77.16 ppm, C_6H_6: ^{1}H = 7.16 ppm, ^{13}C = 128.06 ppm). The following abbreviations were used to indicate the multiplicities of signals: s = singlet, d = doublet, t = triplet, q = quartet, p = pentet, sx = sextet, h = heptet, and m = multiplet. Coupling constants, *J*, are reported in Hertz and are rounded to the nearest 0.1 Hz. Integration of peaks is provided with the assignments indicated where appropriate. ^{1}H NMR and ^{13}C NMR spectra were assigned with the aid of COSY, HSQC, HMBC, DEPT-135, and nOe NMR experiments, and stereochemistry was assigned with the aid of X-ray crystallography. Stereochemical assignments for structurally similar products were made by analogy. Infrared (IR) spectra were recorded on an FTIR spectrometer, and mass spectra were obtained using positive or

negative electrospray ionization (ESI) and atmospheric pressure chemical ionization (APCI) techniques. High-resolution mass data are reported in the form of m/z (intensity relative to the base peak = 100).

All reactions were monitored by thin-layer chromatography (TLC) carried out on 0.20 mm precoated POLYGRAM SIL G/U254 plates. UV light was used as a visualising agent, and potassium permanganate ($KMnO_4$), phosphomolybdic acid (PMA), cerium(IV) sulfate (CAS), cerium ammonium molybdate (CAM), and heat as developing agents. Substrates were purified using Supleco silica gel (60 Å, technical grade, 400 mesh, particle size 0.040-0.063 mm) for column chromatography. Preparative thin-layer chromatography separations were carried out on 0.50 mm E. Merck silica gel plates (60F-254).

All solvents for air- and moisture-sensitive techniques were purchased at the highest commercial grade with ACROS seals and used as received or after distillation from sodium/benzophenone under nitrogen (ethereal solvents). Solvents for filtration, transfers, chromatography, and recrystallisation were acetone (Supleco), dichloromethane (CH_2Cl_2) (Sigma, amylene stabilised, HPLC grade), diethyl ether (Et_2O) (Sigma, BHT stabilised ACS grade), EtOH (Sigma, HPLC grade), ethyl acetate (EtOAc) (Sigma, ACS grade), hexane (Sigma, HPLC grade), methanol (MeOH) (Sigma, ACS grade), pentane (ACS grade), petroleum ether 40-60 °C (Fisher, Analytical grade).

3. Abbreviations

**APCI**	Atmospheric pressure chemical ionization
**CAM**	Cerium ammonium molybdate
**cat**	Catalyst
**CCDC**	Cambridge Crystallographic Data Centre
**cc-pVDZ**	Dunning’s correlation-consistent double-zeta + polarization basis sets
**CDI**	1,1'-Carbonyldiimidazole
**DHK**	Douglas-Kroll-Hess 2nd order scalar relativistic calculations
**DHKSO**	Douglas-Kroll-Hess 4th order relativistic calculations incorporating spin-orbit coupling
**DIBAL-H**	Diisobutylaluminium hydride
**DMF**	Dimethylformamide
**ECP**	Effective core potential
**eq**	Equivalents
**ESI**	Electrospray ionisation
**Et**	Ethyl
**h**	Hour
**HRMS**	High resolution mass spectrometry
**IR**	Infrared radiation
**^{i}Pr**	Isopropyl
**KHMDS**	Potassium bis(trimethylsilyl)amide
**Me**	Methyl
**min**	Minute
**^{n}Bu**	Normal-butyl
**NMR**	Nuclear magnetic resonance
**Ph**	Phenyl
**PMA**	Phosphomolybdic acid
**R_f**	Retention factor
**RT**	Room temperature
**^{t}Bu**	Tertiary-butyl
**THF**	Tetrahydrofuran
**TLC**	Thin layer chromatography

**TS**	Transition state
**SARC**	Segmented all-electron relativistically contracted
**SET**	Single electron transfer
**SOC**	Spin-orbit coupling

4. Preparation of reagents

4.1. Samarium(II) diiodide (SmI_2)[1]

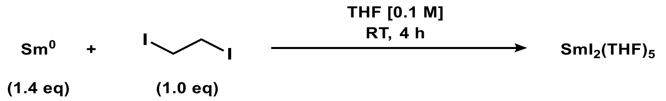

An oven-dried Schlenk flask, equipped with a stirrer bar, was flushed with a strong flow of N_2 (30 min). Subsequently, the flask was loaded with samarium metal (~40 mesh, 1.4 eq) and purified diiodoethane (1.0 eq). The flask was flushed with N_2 (30 min), before freshly distilled and degassed THF (0.1 M) was added, followed by continuous vigorous stirring (> 4 h, room temperature). Finally, the resultant deep blue mixture was allowed to settle (>15 min) prior to use.

Volumetric titration procedure

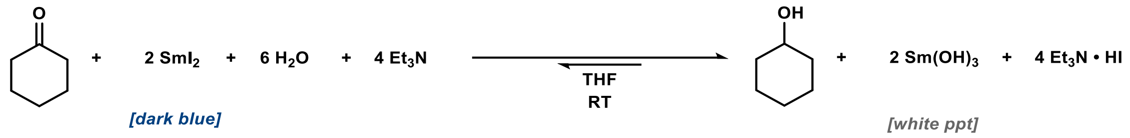

Procedure adapted from that of Hilmersson and co-workers.[2]

An oven-dried vial, equipped with a stirrer bar, was flushed with a strong flow of N_2 (10 min). Subsequently, the vial was charged Et_3N (100 µL, 0.72 mmol), H_2O (20 µL), and freshly prepared SmI_2 (2.5 mL, unknown concentration in THF) was added, followed by continuous vigorous stirring during which a deep purple solution formed. To a separate oven-dried vial, equipped with a stirrer bar, was added cyclohexanone (100 µL, 94.8 mg) and freshly distilled and degassed THF (4 mL) and the solution was stirred (rt, 5 min). Cyclohexanone solution was then added dropwise with vigorous stirring until the endpoint – deep blue/purple solution had turned a pale green, and a white precipitate had formed – and the volume was noted. The final calculation of [SmI_2] could then be carried out using the balanced equation, shown above, simplified to **Eq.1**.

$$\frac{\text{Mass of ketone (mg)} \times \text{Titrant vol added (mL)}}{\left(\frac{\text{MW of ketone (g mol}^{-1}) \times \text{Total titrant vol (4 mL)}}{2}\right) \times \text{SmI}_2 \text{ vol (2.5 mL)}} = [\text{SmI}_2]\ \text{M} \qquad \textbf{Eq. 1}$$

4.2. Preparation of Grignard reagents

4.2.1 General Procedure 1 (GP 1):

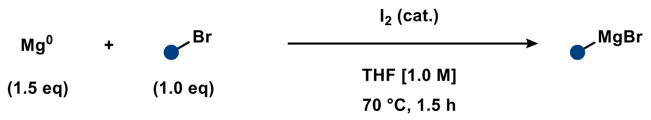

A flame-dried Schlenk flask, equipped with a stirrer bar, and charged with magnesium turnings (365 mg, 15.00 mmol, 1.5 eq) was flame-dried under vacuum (15 min), then under a nitrogen atmosphere, a single I_2 crystal was added with vigorous stirring and gentle heating until a purple vapour was observed. Dry THF (5 mL) was added. To the resultant dark brown solution, alkyl bromide (10.00 mmol, 1.0 eq) in THF (3 mL + 2 mL for rinsing) was added dropwise. The mixture was then transferred to a pre-heated oil bath at 70 °C and stirred vigorously for 1.5 h. The mixture was then cooled to room temperature and titrated prior to use according to the procedure reported by Love and co-workers.[3]

(3-Phenylpropyl)magnesium bromide

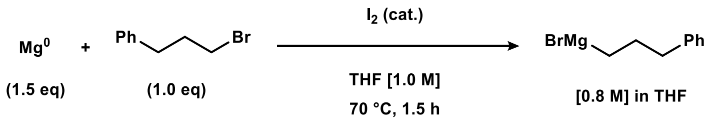

Prepared according to **GP 1** using (3-bromopropyl)benzene (1.53 mL, 10.0 mmol, 1.0 eq). The concentration of (3-phenylpropyl)magnesium bromide in THF was found to be 0.8 M.

(2-Cyclohexylethyl)magnesium bromide

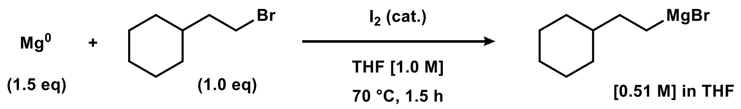

Prepared according to **GP 1** using (2-bromoethyl)cyclohexane (1.56 mL, 10.0 mmol, 1.0 eq). The concentration of (2-cyclohexylethyl)magnesium bromide in THF was found to be 0.51 M.

(2-(1-Methyl-1*H*-indol-3-yl)ethyl)magnesium bromide

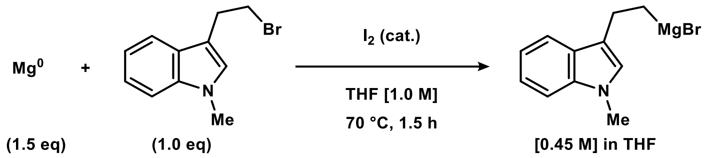

Prepared according to **GP 1** using 3-(2-bromoethyl)-1-methyl-1*H*-indole (2.38 g, 10.0 mmol, 1.0 eq). The concentration of (2-(1-methyl-1*H*-indol-3-yl)ethyl)magnesium bromide in THF was found to be 0.45 M.

(3-(4,5-Diphenyloxazol-2-yl)propyl)magnesium bromide

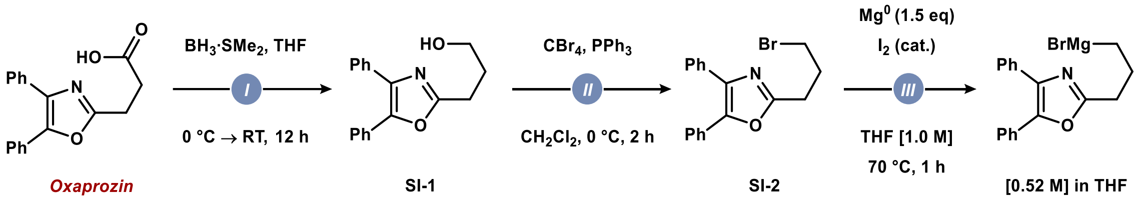

Step I: A flame-dried flask, equipped with a stirrer bar, was charged with oxaprozin (3.52 g, 12.0 mmol, 1.0 eq), and THF (60 mL, 0.2 M). The resultant solution was cooled to 0 °C, and $BH_3{\cdot}Me_2S$ (2M in THF, 12 mL, 24.0 mmol, 2.0 eq) was added dropwise. The resultant solution was gradually warmed to room temperature and allowed to stir for 12 h. At this stage, TLC analysis showed complete consumption of the starting material [R_f of **SI-1** = 0.3 in hexane:EtOAc = 2:1]. The reaction mixture was quenched with MeOH (5 mL) and saturated aqueous NH_4Cl (30 mL), phases were separated, and the aqueous phase was extracted with EtOAc (3 × 20 mL). The combined organic layers were washed with saturated aqueous NaCl (20 mL), dried over anhydrous $MgSO_4$, filtered, and concentrated *in vacuo*. The crude alcohol **SI-1** was used directly for the next step without further purification**.**

Step II: A flame-dried flask, equipped with a stirrer bar, was charged with **SI-1** (3.35 g, 12.0 mmol, assuming 100% pure, 1.0 eq), CBr_4 (4.38 g, 13.2 mmol, 1.1 eq.), and CH_2Cl_2 (60 mL, 0.2 M). The resultant solution was cooled to 0 °C, and PPh_3 (3.78 g, 12 mL, 14.4 mmol, 1.2 eq) was added in one portion. The resultant solution was stirred vigorously at the same temperature until the consumption of starting alcohol **SI-1** was observed by TLC analysis (2 h). The reaction mixture was quenched with H_2O (30 mL). Phases were separated, and the aqueous phase was extracted with CH_2Cl_2 (3 × 30 mL). The

combined organic layers were dried over anhydrous $MgSO_4$, filtered, and concentrated *in vacuo*. The crude bromoalkane was purified by column chromatography on silica gel (petroleum ether:Et_2O = 4:1) to afford the bromide **SI-2** (3.7 g, 10.8 mmol, 90% yield over two steps).

2-(3-Bromopropyl)-4,5-diphenyloxazole (SI-2)

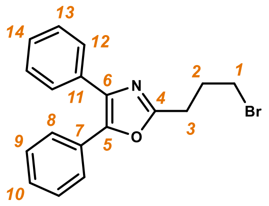

R_f = 0.4, hexane:Et_2O = 4:1, Colourless oil.

^{1}H NMR (400 MHz, $CDCl_3$) δ 7.58 – 7.52 (m, 2H, *2H-12*), 7.52 – 7.47 (m, 2H, *2H-8*), 7.31 – 7.20 (m, 6H, *2H-13* + *H-14* + *2H-9* + *H-10*), 3.49 (t, *J* = 6.5 Hz, 2H, *2H-1*), 2.96 (t, *J* = 7.3 Hz, 2H, *2H-3*), 2.39 – 2.29 (m, 2H, *2H-2*).

^{13}C{H} NMR (101 MHz, $CDCl_3$) δ 162.1 (*C-4*), 145.5 (*C-5*), 135.2 (*C-6*), 132.5 (*C-7*), 129.0 (*C-11*), 128.8 (*2C-9*), 128.7 (*2C-13*), 128.6 (*C-10*), 128.2 (*C-14*), 128.0 (*2C-8*), 126.5 (*2C-12*), 32.6 (*C-1*), 29.9 (*C-3*), 26.8 (*C-2*).

IR (neat, cm^{-1}): 3328, 2924, 2869, 1567, 1443, 1312, 1226, 1060 (fingerprint region excluded).

HRMS (ESI+): calculated for $C_{18}H_{16}ONBrNa$ ($M+Na^+$): 364.0307 Found: 364.0315.

Step III: Prepared according to **GP 1** using 2-(3-bromopropyl)-4,5-diphenyloxazole **SI-2** from step II (3.42 g, 10.0 mmol, 1.0 eq). The concentration of (3-(4,5-diphenyloxazol-2-yl)propyl)magnesium bromide in THF was found to be 0.52 M.

4.2.2 (Tetrahydro-2*H*-pyran-4-yl)magnesium bromide

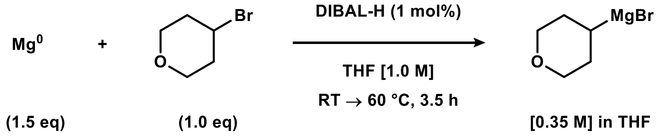

A flame-dried three-necked round-bottom flask equipped with a stirrer bar, reflux condenser, and a dropping funnel was charged with well-ground magnesium turnings (1.09 g, 45.0 mmol, 1.5 eq). The flask was further flame-dried under vacuum (15 min), and then flushed with nitrogen, after which THF (20 mL) and DIBAL-H (1.0 M solution in *n*-hexane, 0.3 mL, 0.30 mmol, 1 mol%) were added. The

suspension was stirred for 30 min at room temperature and then warmed to 60 °C. A solution of 4-bromotetrahydro-2*H*-pyran (3.4 mL, 30.0 mmol, 1.0 eq) in THF (7 mL + 3 mL for rinsing) was added through a dropping funnel at a rate of one drop every 5 s (it took about 40–50 min to complete the addition). The reaction mixture was stirred at 60 °C for 3 h in total and left at room temperature until two phases were clearly separated. The supernatant liquid was titrated to determine the concentration of (tetrahydro-2*H*-pyran-4-yl)magnesium bromide to be 0.35 M.

4.2.3 (2-(1,3-Dioxolan-2-yl)ethyl)magnesium bromide

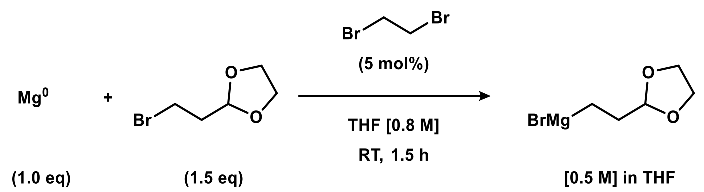

Procedure adapted from Garg and co-workers.[4]

A flame-dried round-bottom flask equipped with a stirrer bar was charged with well-ground magnesium turnings (972 mg, 40.0 mmol, 1.0 eq). The flask was further flame-dried under vacuum (15 min), and then flushed with nitrogen, after which THF (20 mL) was introduced. 1,2-Dibromoethane (0.17 mL, 2.00 mmol, 5 mol%) was then added dropwise, and the reaction mixture was allowed to stir for 5 minutes. The THF in the flask was then removed, and the magnesium was resuspended in fresh THF (75 mL, 0.8 M). 2-(2-Bromoethyl)-1,3-dioxolane (7.0 mL, 60.0 mmol, 1.50 eq) was then added dropwise, and the solution was allowed to stir at 23 ºC for 1.5 h. After this time, the magnesium was completely consumed. The resulting yellow (2-(1,3-dioxolan-2-yl)ethyl)magnesium bromide solution was titrated to determine the concentration to be 0.5 M.

5. Starting material preparation

5.1 General Procedures for making housane ketones

5.1.1 General Procedure 2 (GP 2):

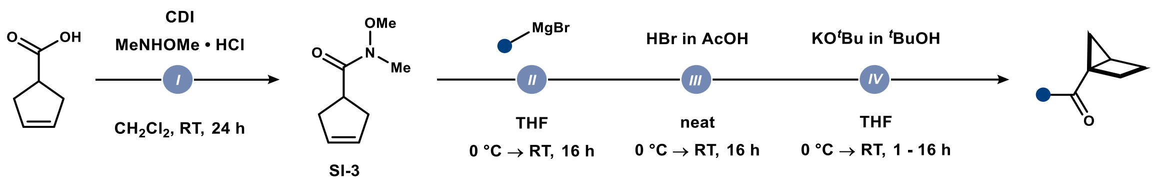

Step I: To a solution of the cyclopent-3-ene-1-carboxylic acid (1 mL,10.0 mmol, 1.0 eq) in dry CH_2Cl_2 (30 mL) was added carbonyldiimidazole (CDI) (1.87 g, 11.5 mmol, 1.15 eq) portion-wise and the solution was stirred at room temperature for 1 h. Subsequently, N_2 was bubbled through the solution for 30 min. *N,O*-Dimethylhydroxylamine hydrochloride (1.27 g, 13.0 mmol, 1.3 eq) was added in one portion, and the reaction mixture was stirred at room temperature for 24 h. The reaction mixture was quenched by the addition of saturated aqueous NH_4Cl (30 mL), layers were separated, and the aqueous layer was extracted with CH_2Cl_2 (3 × 30 mL). The combined organic layers were dried over anhydrous $MgSO_4$, filtered, and concentrated *in vacuo*. The crude product was purified by column chromatography on silica gel (petroleum ether:Et_2O = 1:1) to afford the Weinreb amide **SI-3** (1.23 g, 7.90 mmol, 79% yield).

N-Methoxy-N-methylcyclopent-3-ene-1-carboxamide (SI-3)

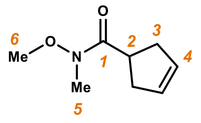

R_f = 0.22, hexane: Et_2O = 2:1. Colourless oil.

^{1}H NMR (400 MHz, $CDCl_3$) δ 5.62 (s, 2H, *2H-4*), 3.67 (s, 3H, *3H-6*), 3.45 (t, J = 8.3 Hz, 1H, *H-2*), 3.17 (s, 3H, *3H-5*), 2.69 – 2.42 (m, 4H, *4H-3*).

Data in accordance with those previously reported.[5]

Step II: A flame-dried flask, equipped with a stirrer bar, was charged with **SI-3** (775 mg, 5.00 mmol, 1.0 eq), and THF or Et_2O (20 mL, 0.25 M). The resultant solution was cooled to 0 °C, and freshly prepared alkyl magnesium bromide (1.3 – 2.0 eq) was added dropwise. The resultant solution was gradually

warmed to room temperature and allowed to stir for 16 h. The reaction mixture was quenched with saturated aqueous NH_4Cl (15 mL), phases were separated, and the aqueous phase was extracted with EtOAc (3 × 15 mL). The combined organic layers were washed with saturated aqueous NaCl (20 mL), dried over anhydrous $MgSO_4$, filtered, and concentrated *in vacuo*. The crude product was purified by column chromatography on silica gel to afford the target product ketone.

Step III: An oven-dried flask, equipped with a stirrer bar, was charged with the ketone from step II (usually 5.00 mmol, 1.0 eq unless otherwise mentioned) and cooled to 0 °C. A solution of 33% HBr in glacial acetic acid (2.6 mL, 15.00 mmol, 3.0 eq) was then added over a period of 5 min. The mixture was gradually warmed to room temperature and stirred vigorously for 16 h, after which it was carefully quenched with dropwise addition of saturated aqueous $NaHCO_3$ (15 mL) and transferred into a separatory funnel containing H_2O (10 mL) and Et_2O (20 mL). The phases were separated, and the aqueous phase was extracted with EtOAc (3 × 20 mL). The combined organic phases were washed with saturated aqueous NaCl solution (20 mL), dried over anhydrous $MgSO_4$, filtered, and concentrated *in vacuo*. The resultant crude material was purified by column chromatography on silica gel to afford the target product bromoketone.

Step IV: An oven-dried flask, equipped with a stirrer bar, was charged with the bromoketone from step III (1.00 mmol, 1.0 eq), and THF (10 mL, 0.1 M). The resultant solution was cooled to 0 °C under nitrogen atmosphere. A 1 M solution of KO^{t}Bu in ^{t}BuOH (1.2 mL, 1.2 mmol,1.2 eq) was then added dropwise, and the reaction mixture was gradually warmed to room temperature. The progress of the reaction was monitored by TLC analysis. Upon completion, the reaction mixture was quenched with saturated aqueous NH_4Cl (5 mL), diluted with water and EtOAc, the layers were separated and the aqueous phase was extracted with EtOAc (3 × 10 mL). The combined organic layers were washed with saturated aqueous NaCl (10 mL), dried over anhydrous $MgSO_4$, filtered, and concentrated *in vacuo*. The resultant crude material was purified by column chromatography on silica gel to afford the target product housane ketone.

Synthesis of 1-(bicyclo[2.1.0]pentan-1-yl)-4-phenylbutan-1-one (1a)

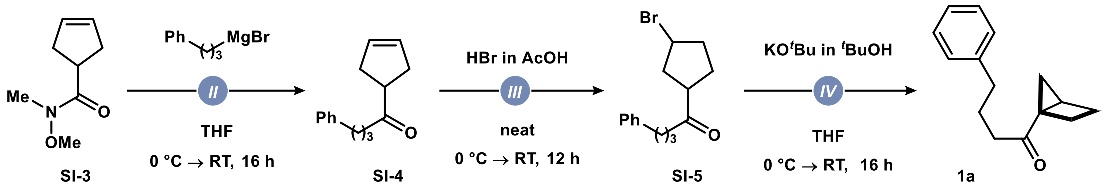

1-(Cyclopent-3-en-1-yl)-4-phenylbutan-1-one (SI-4)

Prepared according to **GP 2** (step II) using (3-phenylpropyl)magnesium bromide (0.8 M in THF; 9.4 mL, 7.50 mmol, 1.5 eq). The crude residue was purified by column chromatography on silica gel (hexane:Et_2O = 2:1) to afford the target ketone **SI-4** (857 mg, 4.00 mmol, 80% yield).

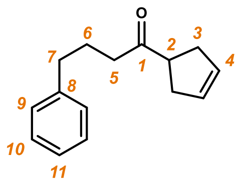

R_f = 0.45, hexane:Et_2O = 2:1, Colourless oil.

^{1}H NMR (400 MHz, $CDCl_3$) δ 7.23 – 7.17 (m, 2H, *2H-9*), 7.14 – 7.06 (m, 3H, *2H-10* + *H-11*), 5.59 – 5.50 (m, 2H, *2H-4*), 3.19 – 3.06 (m, 1H, *H-2*), 2.54 (dd, *J* = 8.4, 6.8 Hz, 2H, *2H-7*), 2.51 – 2.43 (m, 4H, *4H-3*), 2.40 (t, *J* = 7.3 Hz, 2H, *2H-5*), 1.91 – 1.80 (m, 2H, *2H-6*).

^{13}C{H} NMR (101 MHz, $CDCl_3$) δ 211.9 (*C-1*), 141.8 (*C-8*), 129.0 (*2C-10*), 128.5 (*2C-4*), 128.4 (*2C-9*), 126.0 (*C-11*), 49.1 (*C-2*), 40.6 (*C-5*), 35.24 (*C-7*), 35.22 (*2-C3*), 25.3 (*C-6*).

IR (neat, cm^{-1}): 2927,1706, 1495, 1452 (fingerprint region excluded).

HRMS (APCI+): calculated for $C_{15}H_{19}O$ (M+H+): 215.1430 Found: 215.1428.

1-(3-Bromocyclopentyl)-4-phenylbutan-1-one (SI-5)

Prepared according to **GP 2** (step III) using **SI-4** (1.07 g, 5.00 mmol, 1.0 eq). The crude residue was purified by column chromatography on silica gel (petroleum ether:Et_2O = 9:1 to 6:1 to 4:1) to afford two diastereoisomers of the target bromo-ketone **SI-5** (1.34 g, 4.55 mmol, 91% combined yield; d.r. = 1:1). The diastereoisomers were partially separable. Therefore, each isomer was separated for NMR analysis.

DIASTEREOISOMER 1

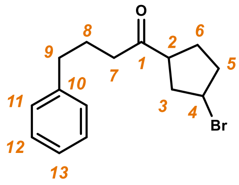

R_f = 0.35, hexane: Et_2O = 2:1. Colourless oil.

^{1}H NMR (400 MHz, $CDCl_3$) δ 7.24 – 7.17 (m, 2H, *2H-11*), 7.15 – 7.07 (m, 3H, *2H-12* + *H-13*), 4.52 – 4.41 (m, 1H, *H-4*), 3.29 – 3.17 (m, 1H, *H-2*), 2.55 (t, *J* = 7.6 Hz, 2H, *2H-9*), 2.49 – 2.33 (m, 2H, *2H-7*), 2.25 (ddd, *J* = 14.1, 8.5, 5.4 Hz, 1H, *H-3a*), 2.17 – 1.98 (m, 4H, *H-3b* + *2H-5* + *H-6a*), 1.89 – 1.81 (m, 2H, *2H-8*), 1.78 – 1.69 (m, 1H, *H-6b*).

^{13}C{H} NMR (101 MHz, $CDCl_3$) δ 211.8 (*C-1*), 141.6 (*C-10*), 128.6 (*2C-12*), 128.5 (*2C-11*), 126.1 (*C-13*), 53.5 (*C-4*), 48.8 (*C-2*), 41.5 (*C-7*), 40.3 (*C-3*), 37.4 (*C-5*), 35.2 (*C-9*), 26.7 (*C-6*), 25.2 (*C-8*).

IR (neat, cm^{-1}): 2938,1706, 1495, 1453 (fingerprint region excluded).

HRMS (APCI+): calculated for $C_{15}H_{20}BrO$ (M+H+): 295.0692 Found: 295.0695.

DIASTEREOISOMER 2

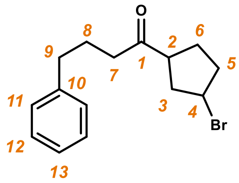

R_f = 0.25, hexane: Et_2O = 2:1. Colourless oil.

^{1}H NMR (400 MHz, $CDCl_3$) δ 7.24 – 7.17 (m, 2H, *2H-11*), 7.15 – 7.04 (m, 3H, *2H-12* + *H-13*), 4.25 – 4.12 (m, 1H, *H-4*), 2.86 – 2.73 (m, 1H, *H-2*), 2.55 (t, *J* = 7.6 Hz, 2H, *2H-9*), 2.47 – 2.31 (m, 3H, *2H-7* + *H-3a*), 2.28 – 2.18 (m, 1H, *H-3b*), 2.11 – 1.94 (m, 3H, *2H-5* + *H-6a*), 1.91 – 1.76 (m, 3H, *2H-8* + *H-6b*).

^{13}C{H} NMR (101 MHz, $CDCl_3$) δ 210.3 (*C-1*), 141.7 (*C-10*), 128.6 (2*C-12*), 128.5 (2*C-11*), 126.1 (*C-13*), 50.1 (*C-4*), 49.6 (*C-2*), 40.5 (*C-7*), 39.5 (*C-3*), 37.7 (*C-5*), 35.2 (*C-9*), 26.9 (*C-6*), 25.2 (*C-8*).

IR (neat, cm^{-1}): 2941, 1707, 1501, 1449 (fingerprint region excluded).

HRMS (APCI+): calculated for $C_{15}H_{20}BrO$ (M+H+): 295.0692 Found: 295.0698.

1-(Bicyclo[2.1.0]pentan-1-yl)-4-phenylbutan-1-one (1a)

Prepared according to **GP 2** (step IV) using **SI-5** (295 mg, 1.00 mmol, 1.0 eq). The crude residue was purified by column chromatography on silica gel (hexane:EtOAc = 4:1) to afford the target housane ketone **1a** (176 mg, 0.82 mmol, 82% yield).

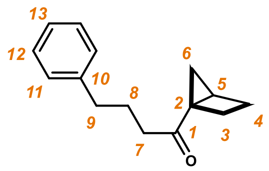

R_f = 0.5, petroleum ether: Et_2O = 4:1. Colourless oil.

^{1}H NMR (500 MHz, $CDCl_3$) δ 7.23 – 7.15 (m, 2H, *2H-11*), 7.14 – 7.03 (m, 3H, *2H-12* + *H-13*), 2.59 – 2.47 (m, 2H, *2H-9*), 2.41 (tdd, *J* = 11.1, 4.2, 1.7 Hz, 1H, *H-3a*), 2.32 – 2.22 (m, 1H, *H-5*), 2.20 – 2.03 (m, 2H, *2H-7*), 1.99 (ttd, *J* = 11.0, 4.6, 1.5 Hz, 1H, *H-4a*), 1.89 – 1.75 (m, 2H, *2H-8*), 1.67 – 1.60 (m, 1H, *H-6a*), 1.56 – 1.48 (m, 1H, *H-3b*), 1.35 – 1.27 (m, 1H, *H-4b*), 1.23 (dd, *J* = 4.7, 2.8 Hz, 1H, *H-6b*).

^{13}C{H} NMR (126 MHz, $CDCl_3$) δ 208.9 (*C-1*), 141.8 (*C-10*), 128.6 (*2C-12*), 128.4 (*2C-11*), 126.0 (*C-13*), 36.2 (*C-7*), 35.3 (*C-9*), 34.4 (*C-2*), 30.1 (*C-5*), 26.3 (*C-6*), 25.5 (*C-8*), 22.4 (*C-3*), 20.6 (*C-4*).

IR (neat, cm^{-1}): 2936, 1668, 1453, 1382 (fingerprint region excluded).

HRMS (APCI+): calculated for $C_{15}H_{19}O$ (M+H+): 215.1430 Found: 215.1431.

Synthesis of 1-(bicyclo[2.1.0]pentan-1-yl)nonan-1-one (1b)

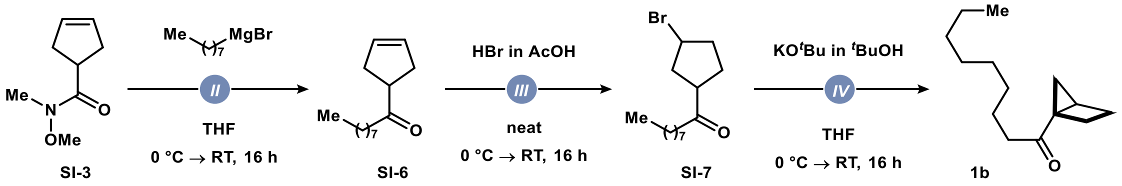

1-(Cyclopent-3-en-1-yl)nonan-1-one (SI-6)

Prepared according to **GP 2** (step II) using 1-octylmagnesium bromide (2.0 M in THF; 3.5 mL, 7.00 mmol, 1.4 eq). The crude residue was purified by column chromatography on silica gel (hexane:EtOAc = 97:3) to afford the target ketone **SI-6** (916.7 mg, 4.40 mmol, 88% yield).

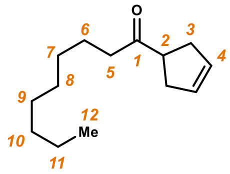

R_f = 0.55, hexane:EtOAc = 9:1. Colourless oil.

^{1}H NMR (500 MHz, $CDCl_3$) δ 5.59 – 5.54 (m, 2H, *2H-4*), 3.17 (tt, *J* = 9.0, 7.2 Hz, 1H, *H-2*), 2.56 – 2.44 (m, 4H, *4H-3*), 2.39 (t, *J* = 7.4 Hz, 2H, *2H-5*),

1.56 – 1.47 (m, 2H, *2H-6*), 1.26 – 1.13 (m, 10H, *2H-7* + *2H-8* + *2H-9* + *2H-10* + *2H-11*), 0.81 (t, *J* = 6.9 Hz, 3H, 3*H-12*).

^{13}C{H} NMR (126 MHz, $CDCl_3$) δ 212.5 (*C-1*), 129.0 (*C-4*), 49.1 (*C-2*), 41.5 (*C-5*), 35.3 (*C-3*), 32.0 (*CH_2*), 29.5 (*CH_2*), 29.5 (*CH_2*), 29.3 (*CH_2*), 24.0 (*CH_2*), 22.8 (*CH_2*), 14.2 (*C-12*).

IR (neat, cm^{-1}): 2923, 2854, 1709, 1459, 1376, 1181 (fingerprint region excluded).

HRMS (ESI+): calculated for $C_{14}H_{24}ONa$ (M+Na$^+$): 231.1719 Found: 231.1719.

1-(3-Bromocyclopentyl)nonan-1-one (SI-7)

Prepared according to **GP 2** (step III) using **SI-6** (1.04 g, 5.00 mmol, 1.0 eq). The crude residue was purified by column chromatography on silica gel (petroleum ether:Et_2O = 97:3) to afford an inseparable mixture of two diastereoisomers of the target bromo-ketone **SI-7** (1.3 g, 4.50 mmol, 90% combined yield; d.r. = 1:0.6).

MIXTURE OF DIASTEREOISOMERS

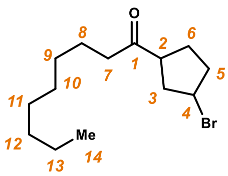

R_f = 0.52, hexane:EtOAc = 9:1. Colourless oil.

^{1}H NMR (400 MHz, $CDCl_3$) δ 4.54 – 4.43 (m, 1H, *H-4 major*), 4.26 – 4.16 (m, 1H, *H-4 minor*), 3.31 – 3.23 (m, 1H, *H-2 major*), 2.88 – 2.79 (m, 1H, *H-2 minor*), 2.46 – 2.32 (m, 5H, 2*H-7 major* + *H-3a major* + *2H-7 minor*), 2.31 – 2.21 (m, 2H, *H-3b major* + *H-3a minor*), 2.19 – 1.95 (m, 7H, 2*H-5 major* + *H-6a major* + *H-3b minor* + *2H-5 minor* + *H-6a minor*), 1.88 – 1.80 (m, 1H, *H-6b minor*), 1.80 – 1.72 (m, 1H, *H-6b major*), 1.55 – 1.46 (m, 4H, 2*H-8 major* + 2*H-8 minor*), 1.27 – 1.14 (m, 20H, *5CH_2 major* + *5 CH_2 minor*), 0.81 (t, *J* = 6.8 Hz, 6H, *3H-14 major* + *3H-14 minor*).

^{13}C{H} NMR (101 MHz, $CDCl_3$) δ 212.2 (*C-1 major*), 210.7 (*C-1 minor*), 53.5 (*C-4 major*), 50.1 (*C-2 minor*), 49.5 (*C-4 minor*), 48.7 (*C-2 major*), 42.4 (*C-7 major*), 41.5 (*C-7 minor*), 40.2 (*C-3 major*), 39.5 (*C-3 minor*), 37.7(*C-5 minor*), 37.4 (*C-5 major*), 31.9 (*CH_2*), 29.5 (*CH_2*), 29.5 (*CH_2*), 29.4 (*CH_2*), 29.2 (*CH_2*), 26.9 (*C-6 minor*), 26.6 (*C-6 major*), 23.9 (*C-8 minor*), 23.8 (*C-8 major*), 22.7 (*CH_2*), 14.2 (*C-14 major* + *C-14 minor*).

IR (neat, cm^{-1}): 2924, 2853, 1709, 1459, 1370, 1217 (fingerprint region excluded).

HRMS (ESI+): calculated for $C_{14}H_{25}OBrNa$ (M+Na$^+$): 311.0981 Found: 311.0985.

1-(Bicyclo[2.1.0]pentan-1-yl)nonan-1-one (1b)

Prepared according to **GP 2** (step IV) using **SI-7** (289 mg, 1.00 mmol, 1.0 eq). The crude residue was purified by column chromatography on silica gel (hexane:EtOAc = 19:1) to afford the target housane ketone **1b** (150 mg, 0.84 mmol, 84% yield).

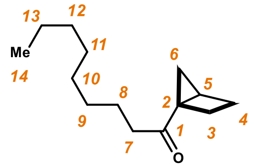

R_f = 0.5, hexane:EtOAc = 9:1. Colourless oil.

^{1}H NMR (500 MHz, $CDCl_3$) δ 2.45 (tdd, *J* = 11.1, 4.1, 1.7 Hz, 1H, *H-3a*), 2.30 (dtd, *J* = 6.4, 3.0, 1.4 Hz, 1H, *H-5*), 2.17 – 1.96 (m, 3H, *2H-7* + *H-4a*), 1.68 (ddd, *J* = 6.3, 4.7, 1.6 Hz, 1H, *H-6a*), 1.54 (dddd, *J* = 11.2, 6.2, 4.5, 1.5 Hz, 1H, *H-3b*), 1.51 – 1.39 (m, 2H, *2H-8*), 1.33 (dddd, *J* = 11.0, 6.6, 4.2, 1.2 Hz, 1H, *H-4b*), 1.24 (dd, *J* = 4.7, 2.8 Hz, 1H, *H-6b*), 1.23 – 1.11 (m, 10H, *5CH$_2$*), 0.84 – 0.74 (m, 3H, *3H-14*).

^{13}C{H} NMR (126 MHz, $CDCl_3$) δ 209.4 (*C-1*), 37.1 (*C-7*), 34.3 (*C-2*), 31.9 (*CH$_2$*), 29.8 (*C-5*), 29.5 (*CH$_2$*), 29.4 (*CH$_2$*), 29.2 (*CH$_2$*), 26.2 (*C-6*), 24.2 (*CH$_2$*), 22.7 (*CH$_2$*), 22.4 (*C-3*), 20.5 (*C-4*), 14.2 (*C-14*).

IR (neat, cm^{-1}): 2924, 2855, 1674, 1463, 1380, 1254, 1135, 1009 (fingerprint region excluded).

HRMS (ESI+): calculated for $C_{14}H_{24}ONa$ (M+Na$^+$): 231.1719 Found: 231.1716.

Synthesis of 1-(bicyclo[2.1.0]pentan-1-yl)-3-phenylpropan-1-one (1d)

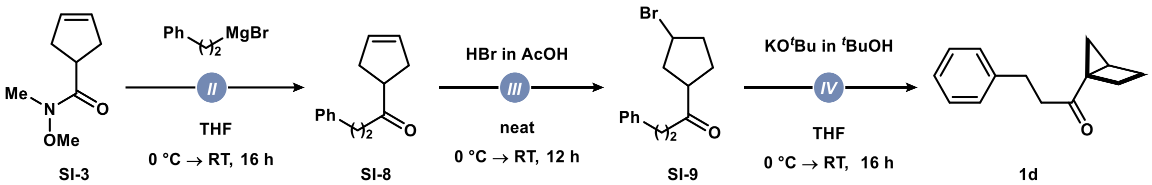

1-(Cyclopent-3-en-1-yl)-3-phenylpropan-1-one (SI-8)

Prepared according to **GP 2** (step II) using phenethylmagnesium bromide (0.82 M in THF; 9.15 mL, 7.50 mmol, 1.5 eq). The crude residue was purified by column chromatography on silica gel (hexane:Et_2O = 2:1) to afford the target ketone **SI-8** (821 mg, 4.10 mmol, 82% yield).

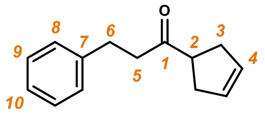

R_f = 0.45, hexane:Et_2O = 2:1, Colourless oil.

^{1}H NMR (500 MHz, $CDCl_3$) δ 7.24 – 7.17 (m, 2H, *2H-8*), 7.16 – 7.07 (m, 3H, *2H-9* + *H-10*), 5.60 – 5.49 (m, 2H, *2H-4*), 3.19 – 3.07 (m, 1H, *H-2*), 2.87 – 2.81 (m, 2H, *2H-6*), 2.75 – 2.68 (m, 2H, *2H-5*), 2.52 – 2.40 (m, 4H, *2H-3*).

^{13}C{H} NMR (126 MHz, $CDCl_3$) δ 211.1 (*C-1*), 141.4 (*C-7*), 129.0 (*2C-4*), 128.6 (*2C-9*), 128.5 (*2C-8*), 126.2 (*C-10*), 49.3 (*C-2*), 43.0 (*C-5*), 35.1 (*2C-3*), 30.0 (*C-6*).

IR (neat, cm^{-1}): 2924, 2851, 1707, 1495, 1407, 1365 (fingerprint region excluded).

HRMS (APCI+): calculated for $C_{14}H_{17}O$ (M+H+): 201.1274 Found: 201.1268.

1-(3-Bromocyclopentyl)-4-phenylbutan-1-one (SI-9)

Prepared according to **GP 2** (step III) using **SI-8** (1.00 g, 5.00 mmol, 1.0 eq). The crude residue was purified by column chromatography on silica gel (petroleum ether:Et_2O = 9:1 to 6:1 to 4:1) to afford two diastereoisomers of the target bromo-ketone **SI-9** (1.27 g, 4.50 mmol, 90% combined yield; d.r. = 1:0.9). The diastereoisomers were partially separable. Therefore, each isomer was separated for NMR analysis.

DIASTEREOISOMER 1

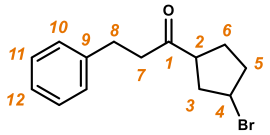

R_f = 0.65, hexane: Et_2O = 2:1. Colourless oil.

^{1}H NMR (500 MHz, $CDCl_3$) δ 7.30 – 7.26 (m, 2H, *2H-10*), 7.21 – 7.16 (m, 3H, *H-12* + *2H-11*), 4.52 (tt, J = 5.4, 2.8 Hz, 1H, *H-4*), 3.36 – 3.25 (m, 1H, *H-2*), 2.91 (t, J = 7.8 Hz, 2H, *2H-8*), 2.86 – 2.72 (m, 2H, *2H-7*), 2.31 (ddd, J = 14.1, 8.5, 5.4 Hz, 1H, *H-3a*), 2.24 – 2.06 (m, 4H, *H-6a* + *2H-5* + *H-3b*), 1.83 – 1.74 (m, 1H, *H-6b*).

^{13}C{H} NMR (126 MHz, $CDCl_3$) δ 210.9 (*C-1*), 141.2 (*C-9*), 128.7 (*2C-11*), 128.5 (*2C-10*), 126.3 (*C-12*), 53.4 (*C-4*), 49.1 (*C-2*), 44.0 (*C-7*), 40.1 (*C-3*), 37.4 (*C-5*), 29.9 (*C-8*), 26.5 (*C-6*).

IR (neat, cm^{-1}): 2927, 1708, 1453, 1368, 1230 (fingerprint region excluded).

HRMS (APCI+): calculated for $C_{14}H_{18}OBr$ (M+H+): 281.0536 Found: 281.0544.

DIASTEREOISOMER 2

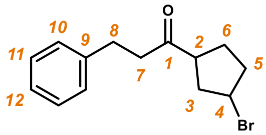

R_f = 0.50, hexane: Et_2O = 2:1. Colourless oil.

^{1}H NMR (500 MHz, $CDCl_3$) δ 7.30 – 7.26 (m, 2H, *2H-10*), 7.21 – 7.17 (m, 3H, *H-12* + *2H-11*), 4.27 (p, J = 6.2 Hz, 1H, *H-4*), 2.93 (t, J = 7.5 Hz, 2H, *2H-8*), 2.89 – 2.85 (m, 1H, *H-2*), 2.83 – 2.77 (m, 2H, *2H-7*), 2.43 (ddd, J = 13.8, 9.0, 6.4 Hz, 1H, *H-3a*), 2.31 (ddd, J = 14.2, 7.9, 6.4 Hz, 1H, *H-3b*), 2.18 – 2.02 (m, 3H, *H-6a* + *2H-5*), 1.91 – 1.84 (m, 1H, *H-6b*).

^{13}C{H} NMR (126 MHz, $CDCl_3$) δ 209.5 (*C-1*), 141.1(*C-9*), 128.5 (*2C-11*), 128.4 (*2C-10*), 126.2 (*C-12*), 50.2 (*C-4*), 49.5, (*C-2*), 43.0 (*C-7*), 39.2 (*C-3*), 37.6 (*C-5*), 29.9 (*C-8*), 26.7 (*C-6*).

IR (neat, cm^{-1}): 2930, 1709, 1455, 1372, 1225 (fingerprint region excluded).

HRMS (APCI+): calculated for $C_{14}H_{18}OBr$ (M+H+): 281.0536 Found: 281.0544.

1-(Bicyclo[2.1.0]pentan-1-yl)-3-phenylpropan-1-one (1d)

Prepared according to **GP 2** (step IV) using **SI-9** (281 mg, 1.00 mmol, 1.0 eq). The crude residue was purified by column chromatography on silica gel (hexane:EtOAc = 19:1) to afford the target housane ketone **1d** (176 mg, 0.88 mmol, 88% yield).

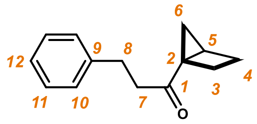

R_f = 0.5 (Hexane: Et_2O = 4:1). Colourless oil.

^{1}H NMR (500 MHz, $CDCl_3$) δ 7.24 – 7.16 (m, 2H, *2H-10*), 7.16 – 7.04 (m, 3H, *2H-11* + *H-12*), 2.86 – 2.73 (m, 2H, *2H-8*), 2.53 – 2.30 (m, 3H, *2H-7* + *H-3a*), 2.24 (dddd, *J* = 6.1, 4.4, 2.9, 1.3 Hz, 1H, *H-5*), 1.96 (ttd, *J* = 11.0, 4.6, 1.5 Hz, 1H, *H-4a*), 1.65 (ddt, *J* = 6.3, 4.7, 1.6 Hz, 1H, *H-6a*), 1.56 – 1.48 (m, 1H, *H-3b*), 1.30 (dddd, *J* = 11.1, 6.6, 4.2, 1.1 Hz, 1H, *H-4b*), 1.23 (dd, *J* = 4.7, 2.9 Hz, 1H, *H-6b*).

^{13}C{H} NMR (126 MHz, $CDCl_3$) δ 208.2 (*C-1*), 141.5 (*C-9*), 128.6 (*2C-11*), 128.5 (*2C-10*), 126.2 (*C-12*), 39.0 (*C-7*), 34.4 (*C-2*), 30.3 (*C-8*), 30.2 (*C-5*), 26.3 (*C-4*), 22.4 (*C-3*), 20.6 (*C-6*).

IR (neat, cm^{-1}): 2960, 2931, 2873, 1668, 1383 (fingerprint region excluded).

HRMS (APCI+): calculated for $C_{14}H_{17}O$ (M+H+): 201.1274 Found: 201.1274.

Synthesis of bicyclo[2.1.0]pentan-1-yl(cyclohexyl)methanone (1j)

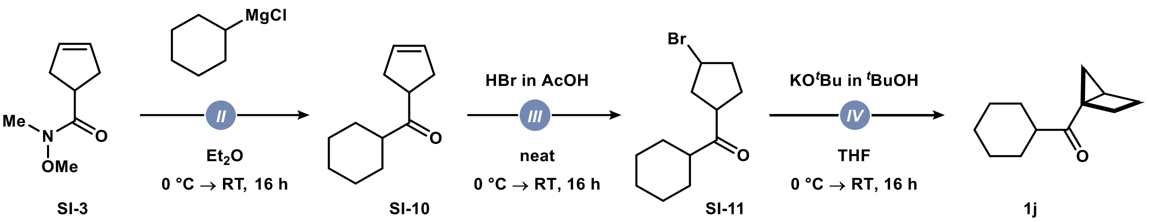

Cyclohexyl(cyclopent-3-en-1-yl)methanone (SI-10)

Prepared according to **GP 2** (step II) using cyclohexylmagnesium chloride (2.0 M in THF; 5 mL, 10.00 mmol, 2.0 eq), and Et_2O as the solvent. The crude residue was purified by column chromatography on silica gel (hexane:EtOAc = 19:1) to afford the target cyclopropyl ketone **SI-10** (597 mg, 3.35 mmol, 67% yield).

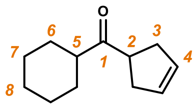

R_f = 0.55, hexane:EtOAc = 9:1. Light-yellow oil.

^{1}H NMR (400 MHz, $CDCl_3$) δ 5.60 – 5.51 (m, 2H, *2H-4*), 3.32 (tt, *J* = 9.1, 7.2 Hz, 1H, *H-2*), 2.55 – 2.45 (m, 4H, *4H-3*), 2.41 (tt, *J* = 11.4, 3.3 Hz, 1H, *H-5*), 1.79 – 1.68 (m, 4H, *2H-6a* + *2H-7a*), 1.65 – 1.57 (m, 1H, *H-8a*), 1.37 – 1.06 (m, 5H, *H-8b* + *2H-6b* + *2H-7b*).

^{13}C{H} NMR (101 MHz, $CDCl_3$) δ 215.5 (*C-1*), 129.0 (*C-4*), 50.1 (*C-2*), 47.1 (*C-5*), 35.6 (*C-3*), 28.9 (*C-6*), 26.0 (*C-8*), 25.9 (*C-7*).

IR (neat, cm^{-1}): 2925, 2853, 1702, 1449, 1259, 1240, 1173, 1145, 1073, 1028 (fingerprint region excluded).

HRMS (ESI+): calculated for $C_{12}H_{18}ONa$ (M+Na$^+$): 201.1250 Found: 201.1250.

(3-Bromocyclopentyl)(cyclohexyl)methanone (SI-11)

Prepared according to **GP 2** (step III) using **SI-10** (891 mg, 5.00 mmol, 1.0 eq). The crude residue was purified by column chromatography on silica gel (petroleum ether:Et_2O = 9:1 to 6:1 to 4:1) to afford two diastereoisomers of the target bromo-ketone **SI-11** (1.17 g, 4.50 mmol, 90% combined yield; d.r. =

1:0.8). The diastereoisomers were partially separable. Therefore, each isomer was separated for NMR analysis.

DIASTEREOISOMER 1

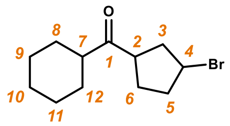

R_f = 0.55, hexane:EtOAc = 9:1. Colourless oil.

^{1}H NMR (400 MHz, $CDCl_3$) δ 4.51 (tt, *J* = 5.1, 2.7 Hz, 1H, *H-4*), 3.48 – 3.34 (m, 1H, *H-2*), 2.43 – 2.33 (m, 1H, *H-7*), 2.26 (ddd, *J* = 14.1, 8.6, 5.4 Hz, 1H, *H-3a*), 2.18 – 1.99 (m, 4H, *H-3b* + *2H-5* + *H-6a*), 1.83 – 1.68 (m, 5H, *H-6b* + *H-8a* + *H-9a* + *H-11a* + *H-12a*), 1.65 – 1.56 (m, 1H, *H-10a*), 1.36 – 1.07 (m, 5H, *H-10b* + *H-8b* + *H-9b* + *H-11b* + *H-12b*).

^{13}C{H} NMR (101 MHz, $CDCl_3$) δ 215.5 (*C-1*), 53.9 (*C-4*), 50.7 (*C-7*), 46.8 (*C-2*), 40.7 (*C-3*), 37.5 (*C-5*), 28.7 (*C-8*), 28.5 (*C-11*), 27.1 (*C-6*), 25.9 (*C-9*), 25.8 (*C-11*), 25.7 (*C-10*).

IR (neat, cm^{-1}): 2926, 2853, 1703, 1448, 1373, 1346, 1316, 1217, 1144 (fingerprint region excluded).

HRMS (ESI+): calculated for $C_{12}H_{19}OBrNa$ (M+Na$^+$): 281.0511 Found: 281.0511.

DIASTEREOISOMER 2

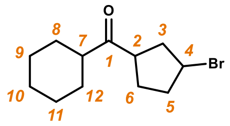

R_f = 0.52, hexane:EtOAc = 9:1. Colourless oil.

^{1}H NMR (400 MHz, $CDCl_3$) δ 4.18 (dq, *J* = 7.4, 6.3 Hz, 1H, *H-4*), 2.97 (qd, *J* = 8.8, 7.0 Hz, 1H, *H-2*), 2.45 – 2.32 (m, 2H, *H-7* + *H-3a*), 2.26 – 2.15 (m, 1H, *H-3b*), 2.15 – 2.07 (m, 1H, *H-6a*), 2.01 (dddd, *J* = 10.3, 9.4, 8.2, 4.8 Hz, 2H, *2H-5*), 1.85 – 1.66 (m, 5H, 5H, *H-6b* + *H-8a* + *H-9a* + *H-11a* + *H-12a*), 1.64 – 1.56 (m, 1H, *H-10a*), 1.37 – 1.07 (m, 5H, *H-10b* + *H-8b* + *H-9b* + *H-11b* + *H-12b*).

^{13}C{H} NMR (101 MHz, $CDCl_3$) δ 213.9 (*C-1*), 50.0 (*C-4*), 49.3 (*C-7*), 48.3 (*C-2*), 40.0 (*C-3*), 37.7 (*C-5*), 28.9 (*C-8*), 28.8 (*C-11*), 27.3 (*C-6*), 26.0 (*C-9*), 25.9 (*C-11*), 25.8 (*C-10*).

IR (neat, cm^{-1}): 2930, 1705, 1454, 1370, 1355, 1323, 1210, 1142 (fingerprint region excluded).

HRMS (ESI+): calculated for $C_{12}H_{19}OBrNa$ (M+Na$^+$): 281.0511 Found: 281.0516.

Bicyclo[2.1.0]pentan-1-yl(cyclohexyl)methanone (1j)

Prepared according to **GP 2** (step IV) using **SI-11** (259 mg, 1.00 mmol, 1.0 eq). The crude residue was purified by column chromatography on silica gel (hexane:EtOAc = 19:1) to afford the target housane ketone **1j** (150 mg, 0.84 mmol, 84% yield).

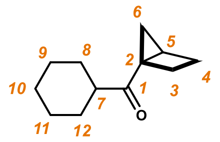

R_f = 0.52, hexane:EtOAc = 9:1. Colourless oil.

^{1}H NMR (400 MHz, $CDCl_3$) δ 2.44 (tdd, *J* = 11.0, 4.2, 1.7 Hz, 1H, *H-3a*), 2.29 (dddd, *J* = 6.1, 4.4, 2.9, 1.4 Hz, 1H, *H-5*), 2.19 (tt, *J* = 11.4, 3.3 Hz, 1H, *H-7*), 2.01 (ttd, *J* = 11.0, 4.6, 1.5 Hz, 1H, *H-4a*), 1.74 – 1.53 (m, 7H, *H-6a* + *H-3b* + *H-8a* + *H-12a* + *H-9a* + *H-11a* + *H-10a*), 1.38 – 1.09 (m, 7H, *H-4b* + *H-6b* + *H-8b* + *H-12b* + *H-9b* + *H-11b* + *H-10b*).

^{13}C{H} NMR (101 MHz, $CDCl_3$) δ 212.4 (*C-1*), 45.0 (*C-7*), 33.3 (*C-2*), 29.8 (*C-5*), 28.9 (*C-8*), 28.7 (*C-12*), 26.3 (*C-6*), 25.9 (*C-9*), 25.9 (*C-11*), 25.8 (*C-10*), 22.8 (*C-3*), 20.6 (*C-4*).

IR (neat, cm^{-1}): 2927, 2854, 1667, 1448, 1386, 1255, 1144, 1012 (fingerprint region excluded).

HRMS (ESI+): calculated for $C_{12}H_{19}O$ (M+H$^+$): 179.1430 Found: 179.1431.

Synthesis of bicyclo[2.1.0]pentan-1-yl(tetrahydro-2*H*-pyran-4-yl)methanone (1l)

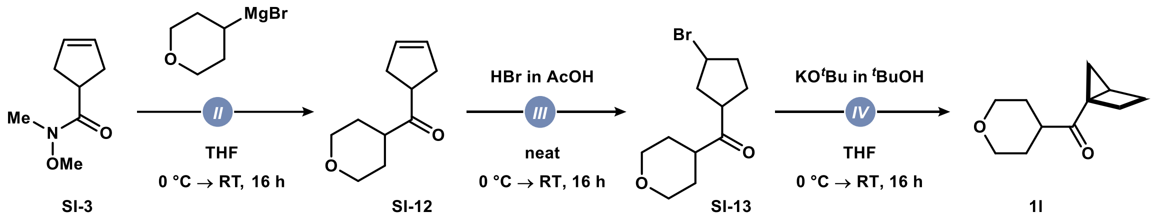

Cyclopent-3-en-1-yl(tetrahydro-2*H*-pyran-4-yl)methanone (SI-12)

Prepared according to **GP 2** (step II) using (tetrahydro-2*H*-pyran-4-yl)magnesium bromide (0.35 M in THF; 28.6 mL, 10.0 mmol, 2.0 eq). The crude residue was purified by column chromatography on silica gel (hexane:EtOAc = 6:1) to afford the target ketone **SI-12** (189 mg, 1.05 mmol, 21% yield).

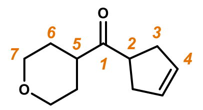

R_f = 0.25. (Hexane:EtOAc = 4:1). Colourless oil.

^{1}H NMR (400 MHz, $CDCl_3$) δ 5.61 – 5.54 (m, 2H, *H-4*), 3.95 (dt, *J* = 11.5, 3.5 Hz, 2H, *H-7a*), 3.42 – 3.28 (m, 3H, *H-7b* + *H-2*), 2.63 (ddd, *J* = 15.4, 8.9, 7.0 Hz, 1H, H-5), 2.56 – 2.47 (m, 4H, H-3), 1.71 – 1.64 (m, 4H, H-6).

^{13}C{H} NMR (101 MHz, $CDCl_3$) δ 213.4 (*C-1*), 128.9 (*C-4*), 67.5 (*C-7*), 46.9 (*C-5*), 46.8 (*C-2*), 35.7 (*C-3*), 28.6 (*C-6*).

IR (neat, cm^{-1}): 2948, 2922, 2847, 1703, 1444, 1274, 1240, 1148, 1115, 1093, 1023 (fingerprint region excluded).

HRMS (ESI+): calculated for $C_{11}H_{16}O_2Na$ (M+Na$^+$): 203.1043 Found: 203.1039.

(3-Bromocyclopentyl)(tetrahydro-2*H*-pyran-4-yl)methanone (SI-13)

Prepared according to **GP 2** (step **III**) using **SI-12** (180 mg, 1.00 mmol, 1.0 eq). The crude residue was purified by column chromatography on silica gel (hexane:Et_2O = 2:1) to afford two diastereoisomers of the target bromo-ketone **SI-13** (230 mg, 0.88 mmol, 88% combined yield; d.r. = 1:1). The diastereoisomers were partially separable. Therefore, each isomer was separated for NMR analysis.

DIASTEREOISOMER 1

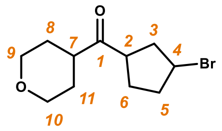

R_f = 0.25. (Hexane:EtOAc = 4:1). Colourless oil.

^{1}H NMR (400 MHz, $CDCl_3$) δ 4.52 (tt, *J* = 5.2, 2.6 Hz, 1H, *H-4*), 3.94 (ddd, *J* = 11.4, 4.4, 2.0 Hz, 2H, *H-9a* + *H-10a*), 3.49 – 3.31 (m, 3H, *H-2* + *H-9b* + *H-10b*), 2.60 (tt, *J* = 10.3, 4.6 Hz, 1H, *H-7*), 2.27 (ddd, *J* = 14.1, 8.7, 5.4 Hz, 1H, *H-3a*), 2.20 – 2.00 (m, 4H, *H-3b* + *H-5a* + *H-6a* + *H-5b*), 1.81 – 1.55 (m, 5H, *H-6b* + 2*H-8* + 2*H-11*).

^{13}C{H} NMR (101 MHz, $CDCl_3$) δ 213.3 (*C-1*), 67.3 (*C-9*), 67.3 (*C-10*), 53.6 (*C-4*), 47.4 (*C-2*), 46.4 (*C-7*), 40.7 (*C-3*), 37.4 (*C-5*), 28.3 (*C-11*), 28.2 (*C-8*), 27.1 (*C-6*).

IR (neat, cm^{-1}): 2950, 2845, 1703, 1443, 1274, 1254, 1239, 1147, 1114, 1092, 1022 (fingerprint region excluded).

HRMS (ESI+): calculated for $C_{24}H_{24}O_2NBrNa$ (M+Na$^+$): 460.0883 Found: 460.0890.

DIASTEREOISOMER 2

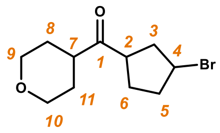

R_f = 0.22. (Hexane:EtOAc = 4:1). Colourless oil.

^{1}H NMR (400 MHz, $CDCl_3$) δ 4.21 (p, *J* = 6.3 Hz, 1H, *H-4*), 3.94 (dt, *J* = 11.5, 3.4 Hz, 2H, *H-9a* + *H-10a*), 3.43 – 3.30 (m, 2H, *H-9b* + *H-10b*), 3.06 – 2.94 (m, 1H, *H-2*), 2.69 – 2.56 (m, 1H, *H-7*), 2.40 (ddd, *J* = 13.4, 8.8, 6.4 Hz, 1H, *H-3a*), 2.24 (ddd, *J* = 14.3, 8.1, 6.8 Hz, 1H, *H-3b*), 2.16 – 2.08 (m, 1H, *H-5a*), 2.08 – 1.97 (m, 2H, *H-6a* + *H-5b*), 1.88 – 1.79 (m, 1H, *H-6b*), 1.74 – 1.58 (m, 4H, 2*H-8* + *2H-11*).

^{13}C{H} NMR (101 MHz, $CDCl_3$) δ 211.8 (*C-1*), 67.3 (*C-9*), 67.3 (*C-10*), 49.2 (*C-4*), 47.8 (*C-2*), 46.6 (*C-7*), 39.9 (*C-3*), 37.7 (*C-5*), 28.5 (*C-11*), 28.4 (*C-8*), 27.2 (*C-6*).

IR (neat, cm^{-1}): 2953, 2849, 1705, 1439, 1270, 1249, 1142, 1090, 1019 (fingerprint region excluded).

HRMS (ESI+): calculated for $C_{11}H_{17}O_2BrNa$ (M+Na$^+$): 283.0304 Found: 283.0305.

Bicyclo[2.1.0]pentan-1-yl(tetrahydro-2*H*-pyran-4-yl)methanone (1l)

Prepared according to **GP 2** (step IV) using **SI-13** (261 mg, 1.00 mmol, 1.0 eq). The crude residue was purified by column chromatography on silica gel (hexane:EtOAc = 3:1) to afford the target housane ketone **1l** (144 mg, 0.80 mmol, 80% yield).

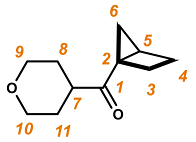

R_f = 0.24. (Hexane:EtOAc = 4:1). Colourless oil.

^{1}H NMR (400 MHz, $CDCl_3$) δ 3.91 (dddd, *J* = 12.4, 6.3, 4.7, 2.5 Hz, 2H, *H-9a* + *H-10a*), 3.34 (tt, *J* = 11.5, 2.1 Hz, 2H, *H-9b* + *H-10b*), 2.56 – 2.40 (m, 2H, *H-7* + *H-3a*), 2.34 (dddd, *J* = 6.1, 4.4, 2.9, 1.4 Hz, 1H, *H-5*), 2.04 (ttd, *J* = 11.0, 4.6, 1.5 Hz, 1H, *H-4a*), 1.76 – 1.56 (m, 4H, *H-8a* + *H-11a*+ *H-6a* + *H-3b*), 1.52 (dddq, *J* = 13.7, 8.9, 4.5, 2.4 Hz, 2H, *H-8b* + *H-11b*), 1.36 (dddd, *J* = 10.9, 6.6, 4.1, 1.2 Hz, 1H, *H-4b*), 1.27 (dd, *J* = 4.6, 2.9 Hz, 1H, *H-6b*).

^{13}C{H} NMR (101 MHz, $CDCl_3$) δ 210.2 (*C-1*), 67.3 (*C-9*), 67.3 (*C-9'*), 42.2 (*C-7*), 33.2 (*C-2*), 30.3 (*C-5*), 28.4 (*C-8*), 28.3 (*C-8'*), 26.6 (*C-6*), 22.9 (*C-3*), 20.6 (*C-4*).

IR (neat, cm^{-1}): 2946, 2846, 1666, 1384, 1350, 1260, 1239, 1124, 1089, 1021 (fingerprint region excluded).

HRMS (ESI+): calculated for $C_{11}H_{16}O_2Na$ (M+Na$^+$): 203.1043 Found: 203.1043.

Synthesis of bicyclo[2.1.0]pentan-1-yl)(4-fluorophenyl)methanone (1o)

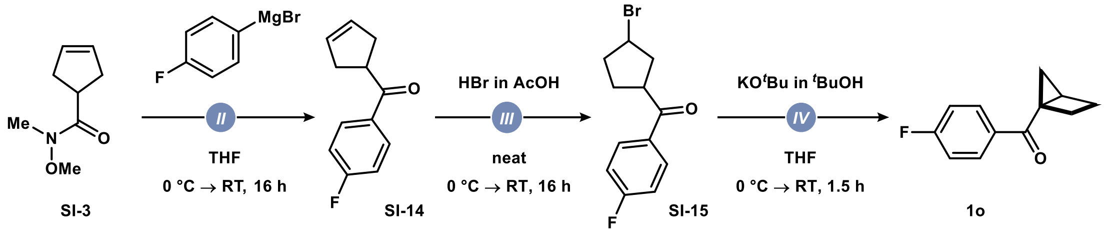

Cyclopent-3-en-1-yl(4-fluorophenyl)methanone (SI-14)

Prepared according to **GP 2** (step II) using (4-fluorophenyl)magnesium bromide (1.0 M in THF; 6.5 mL, 6.50 mmol, 1.3 eq). The crude residue was purified by column chromatography on silica gel (hexane:EtOAc = 19:1) to afford the target ketone **SI-14** (571 mg, 3.00 mmol, 60% yield).

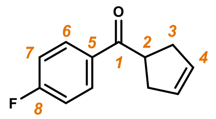

R_f = 0.55. (Hexane:Et_2O = 4:1). Colourless oil.

^{1}H NMR (500 MHz, $CDCl_3$) δ 7.96 – 7.89 (m, 2H, *H-6*), 7.09 – 7.02 (m, 2H, *H-7*), 5.66 – 5.53 (m, 2H, *H-4*), 3.95 (tt, *J* = 9.5, 6.3 Hz, 1H, *H-2*), 2.72 – 2.58 (m, 4H, *H-3*).

^{13}C{H} NMR (126 MHz, $CDCl_3$) δ 199.9 (*C-1*), 165.7 (d, *J* = 254.3 Hz, *C-8*), 132.9 (d, *J* = 3.0 Hz, *C-5*), 131.3 (d, *J* = 9.2 Hz, *C-6*), 129.0 (*C-4*), 115.8 (d, *J* = 21.9 Hz, *C-7*), 44.2 (*C-2*), 36.4 (*C-3*).

^{19}F NMR (376 MHz, $CDCl_3$) δ -105.78 (tt, *J* = 8.4, 5.4 Hz).

IR (neat, cm^{-1}): 1681, 1595, 1505, 1355, 1216, 1155, 1015 (fingerprint region excluded).

HRMS (ESI+): calculated for $C_{12}H_{12}OF$ (M+H$^+$): 191.0867 Found: 191.0866.

(3-Bromocyclopentyl)(4-fluorophenyl)methanone (SI-15)

Prepared according to **GP 2** (step III) using **SI-14** (951 mg, 5.00 mmol, 1.0 eq). The crude residue was purified by column chromatography on silica gel (hexane:Et_2O = 9:1 to 6:1) to afford two diastereoisomers of the target bromo-ketone **SI-15** (1.08 g, 4.00 mmol, 80% combined yield; d.r. = 1:1). The diastereoisomers were partially separable. Therefore, each isomer was separated for NMR analysis.

DIASTEREOISOMER 1

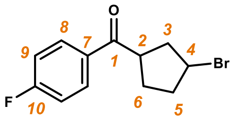

R_f = 0.5. (Hexane:Et_2O = 4:1). Colourless oil.

^{1}H NMR (500 MHz, $CDCl_3$) δ 7.99 – 7.90 (m, 2H, *H-8*), 7.12 – 7.04 (m, 2H, *H-9*), 4.57 (tt, *J* = 5.3, 2.8 Hz, 1H, *H-4*), 4.05 (dtd, *J* = 10.4, 8.2, 5.7 Hz, 1H, *H-2*), 2.50 (ddd, *J* = 14.0, 8.3, 5.4 Hz, 1H, *H-3a*), 2.36 – 2.24 (m, 2H, *H-6a* + *H-3b*), 2.20 – 2.07 (m, 2H, 2*H-5*), 1.89 (dddd, *J* = 12.9, 8.5, 5.7, 4.2 Hz, 1H, *H-6b*).

^{13}C{H} NMR (126 MHz, $CDCl_3$) δ 199.8 (*C-1*), 165.9 (d, *J* = 254.9 Hz, *C-10*), 132.8 (d, *J* = 3.0 Hz, *C-7*), 131.3 (d, *J* = 9.3 Hz, *C-8*), 115.9 (d, *J* = 21.9 Hz, *C-9*), 53.9 (*C-4*), 44.2 (*C-2*), 40.9 (*C-3*), 37.6 (*C-5*), 28.0 (*C-6*).

^{19}F NMR (376 MHz, $CDCl_3$) δ -105.03 (tt, *J* = 8.4, 5.4 Hz), -105.22 (tt, *J* = 8.4, 5.3 Hz).

IR (neat, cm^{-1}): 1679, 1594, 1505, 1409, 1355, 1317, 1298, 1216, 1154, 1013 (fingerprint region excluded).

HRMS (ESI+): calculated for $C_{12}H_{12}OBrFNa$ (M+Na$^+$): 292.9948 Found: 292.9947.

DIASTEREOISOMER 2

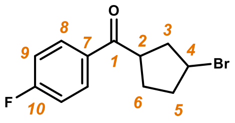

R_f = 0.3. (Hexane:Et_2O = 4:1). Colourless oil.

^{1}H NMR (500 MHz, $CDCl_3$) δ 7.94 – 7.86 (m, 2H, *H-8*), 7.10 – 7.03 (m, 2H, *H-9*), 4.31 – 4.21 (m, 1H, *H-4*), 3.69 – 3.59 (m, 1H, *H-2*), 2.52 (ddd, *J* = 13.6, 9.0, 6.5 Hz, 1H, *H-3a*), 2.39 (dt, *J* = 14.5, 7.5 Hz, 1H, *H-3b*), 2.25 – 2.12 (m, 2H, *H-5a* + *H-6a*), 2.10 – 2.00 (m, 1H, *H-5b*), 1.99 – 1.88 (m, 1H, *H-6b*).

^{13}C{H} NMR (126 MHz, $CDCl_3$) δ 198.6 (*C-1*), 165.8 (d, *J* = 254.8 Hz, *C-10*), 132.8 (d, *J* = 3.0 Hz, *C-7*), 131.2 (d, *J* = 9.3 Hz, *C-8*), 115.9 (d, *J* = 21.8 Hz, *C-9*), 49.1 (*C-4*), 45.3 (*C-2*), 40.3 (*C-3*), 37.9 (*C-5*), 28.2 (*C-6*).

^{19}F NMR (471 MHz, $CDCl_3$) δ -105.01, -105.20.

IR (neat, cm^{-1}): 1680, 1596, 1503, 1405, 1360, 1324, 1289, 1213, 1149, 1015 (fingerprint region excluded).

HRMS (ESI+): calculated for $C_{12}H_{12}OBrFNa$ (M+Na$^+$): 292.9948 Found: 292.9950.

Bicyclo[2.1.0]pentan-1-yl)(4-fluorophenyl)methanone (1o)

Prepared according to **GP 2** (step IV) using **SI-15** (261 mg, 1.00 mmol, 1.0 eq). The crude residue was purified by column chromatography on silica gel (hexane:EtOAc = 4:1) to afford the target housane ketone **1o** (162 mg, 0.85 mmol, 85% yield).

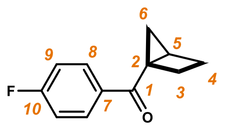

R_f = 0.42. (Hexane:Et_2O = 4:1). Colourless oil.

^{1}H NMR (400 MHz, $CDCl_3$) δ 7.74 – 7.62 (m, 2H, *H-8*), 7.10 – 6.94 (m, 2H, *H-9*), 2.54 (dddd, *J* = 6.3, 4.7, 2.9, 1.4 Hz, 1H, *H-5*), 2.47 (tdt, *J* = 10.9, 4.3, 1.4 Hz, 1H, *H-3a*), 2.12 – 2.01 (m, 1H, *H-4a*), 1.94 (dtt, *J* = 10.9, 4.6, 1.3 Hz, 1H, *H-3b*), 1.70 (ddq, *J* = 5.9, 4.3, 1.5 Hz, 1H, *H-6a*), 1.44 (ddd, *J* = 4.2, 2.9, 1.0 Hz, 1H, *H-6b*), 1.39 (ddd, *J* = 10.8, 6.1, 4.4 Hz, 1H, *H-4b*).

^{13}C{H} NMR (101 MHz, $CDCl_3$) δ 200.6 (*C-1*), 165.1 (d, *J* = 253.2 Hz, *C-10*), 133.6 (d, *J* = 3.0 Hz, *C-7*), 130.7 (d, *J* = 9.1 Hz, *C-8*), 115.5 (d, *J* = 21.7 Hz, *C-9*), 32.6 (*C-2*), 30.3 (*C-5*), 30.0 (*C-6*), 26.2 (*C-3*), 21.0 (*C-4*).

^{19}F NMR (376 MHz, $CDCl_3$) δ -106.57 (ttd, *J* = 8.5, 5.5, 2.9 Hz).

IR (neat, cm^{-1}): 2936, 1650, 1600, 1505, 1409, 1355, 1296, 1284, 1261, 1223, 1206, 1152, 1029 (fingerprint region excluded).

HRMS (ESI+): calculated for $C_{12}H_{11}OFNa$ (M+Na$^+$): 213.0686 Found: 213.0687.

Synthesis of bicyclo[2.1.0]pentan-1-yl(4-methoxyphenyl)methanone (1p)

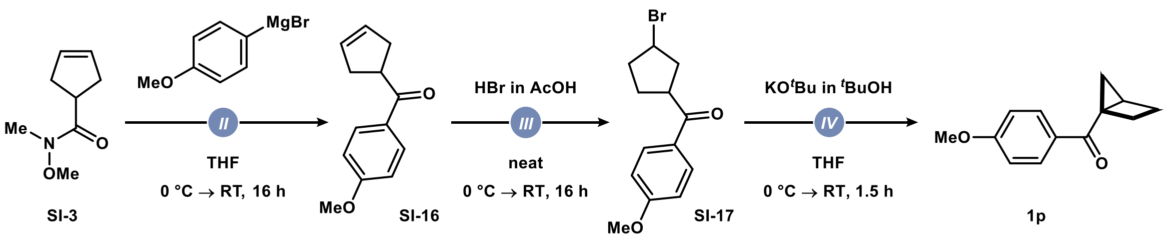

Cyclopent-3-en-1-yl(4-methoxyphenyl)methanone (SI-16)

Prepared according to **GP 2** (step II) using (4-methoxyphenyl)magnesium bromide (0.5 M in THF; 13.0 mL, 6.50 mmol, 1.3 eq). The crude residue was purified by column chromatography on silica gel (hexane:EtOAc = 4:1) to afford the target ketone **SI-16** (718 mg, 3.55 mmol, 71% yield).

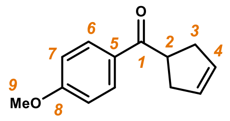

R_f = 0.3 (Hexane: Et_2O = 4:1). Colourless oil.

^{1}H NMR (400 MHz, $CDCl_3$) δ 7.90 (d, *J* = 8.9 Hz, 2H, *2H-6*), 6.88 (d, *J* = 8.9 Hz, 2H, *2H-7*), 5.66 – 5.57 (m, 2H, *2H-4*), 3.97 (tt, *J* = 9.6, 6.3 Hz, 1H, *H-2*), 3.81 (s, 3H, *3H-9*), 2.76 – 2.55 (m, 4H, *4H-3*).

^{13}C{H} NMR (126 MHz, $CDCl_3$) δ 200.3 (*C-1*), 163.4 (*C-8*), 131.0 (*C-6*), 129.6 (*C-5*), 129.1 (*C-4*), 113.9 (*C-7*), 55.6 (*C-9*), 44.0 (*C-2*), 36.6 (*2C-3*).

IR (neat, cm^{-1}): 1672, 1559, 1509, 1259, 1225 (fingerprint region excluded).

HRMS (APCI+): calculated for $C_{13}H_{15}O_2$ (M+H+): 203.1067 Found: 203.1068.

(3-Bromocyclopentyl)(4-methoxyphenyl)methanone (SI-17)

Prepared according to **GP 2** (step **III**) using **SI-16** (1.01 g, 5.00 mmol, 1.0 eq). The crude residue was purified by column chromatography on silica gel (hexane:Et_2O = 6:1 to 4:1 to 3:1) to afford two diastereoisomers of the target bromo-ketone **SI-17** (906 mg, 3.20 mmol, 64% combined yield; d.r. = 1:0.8). The diastereoisomers were partially separable. Therefore, each isomer was separated for NMR analysis.

MAJOR DIASTEREOISOMER

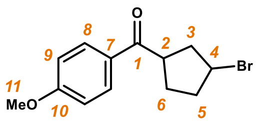

R_f = 0.3 (Hexane: Et_2O = 4:1). Colourless oil.

^{1}H NMR (500 MHz, $CDCl_3$) δ 7.96 – 7.83 (m, 2H, *2H-8*), 6.95 – 6.82 (m, 2H, *2H-9*), 4.57 (tt, *J* = 5.3, 2.8 Hz, 1H, *H-4*), 4.04 (dtd, *J* = 10.3, 8.1, 5.7 Hz, 1H, *H-2*), 3.80 (s, 3H, *3H-11*), 2.50 (ddd, *J* = 14.0, 8.2, 5.4 Hz, 1H, *H-3a*), 2.34 – 2.22 (m, 2H, *H-3b* + *H-6a*), 2.21 – 2.05 (m, 2H, *2H-5*), 1.89 (dddd, *J* = 12.8, 8.5, 5.7, 4.2 Hz, 1H, *H-6b*).

^{13}C{H} NMR (126 MHz, $CDCl_3$) δ 200.0 (*C-1*), 163.7 (*C-10*), 131.0 (*C-8*), 129.4 (*C-7*), 114.0 (*C-9*), 55.6 (*C-11*), 54.2 (*C-4*), 43.9 (*C-2*), 41.1 (*C-3*), 37. 6 (*C-5*), 28.1 (*C-6*).

IR (neat, cm^{-1}): 2962, 2934, 1672, 1599, 1509, 1259, 1224, 1170, 1029 (fingerprint region excluded).

HRMS (APCI+): calculated for $C_{13}H_{16}O_2Br$ (M+H+): 283.0328 Found: 283.0333.

MINOR DIASTEREOISOMER

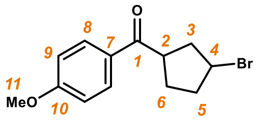

R_f = 0.25 (Hexane: Et_2O = 4:1). Colourless oil.

^{1}H NMR (500 MHz, $CDCl_3$) δ 7.86 (d, *J* = 9.0 Hz, 2H, *2H-8*), 6.87 (d, *J* = 8.9 Hz, 2H, *2H-9*), 4.31 – 4.18 (m, 1H, *H-4*), 3.80 (s, 3H, *3H-11*), 3.70 – 3.58 (m, 1H, *H-2*), 2.51 (ddd, *J* = 13.7, 8.8, 6.6 Hz, 1H, *H-3a*), 2.38 (dt, *J* = 14.0, 7.9 Hz, 1H, *H-3b*), 2.25 – 2.11 (m, 2H, *H-5a* + *H-6a*), 2.08 – 1.99 (m, 1H, *H-5b*), 1.97 – 1.86 (m, 1H, *H-6b*).

^{13}C{H} NMR (126 MHz, $CDCl_3$) δ 198.8 (*C-1*), 163.6 (*C-10*), 130.9 (*C-8*), 129.4 (*C-7*), 114.0 (*C-9*), 55.6 (*C-11*), 49.2 (*C-4*), 45.0 (*C-2*), 40.5 (*C-3*), 37.9 (*C-5*), 28.3 (*C-6*).

IR (neat, cm^{-1}): 2951, 2924, 1674, 1600, 1510, 1260, 1234, 1171, 1028 (fingerprint region excluded).

HRMS (APCI+): calculated for $C_{13}H_{16}O_2Br$ (M+H+): 283.0328 Found: 283.0332.

Bicyclo[2.1.0]pentan-1-yl(4-methoxyphenyl)methanone (1p)

Prepared according to **GP 2** (step IV) using **SI-17** (283 mg, 1.0 mmol, 1.0 eq). The crude residue was purified by column chromatography on silica gel (hexane:Et_2O = 4:1) to afford the target housane ketone **1p** (162 mg, 0.80 mmol, 80% yield).

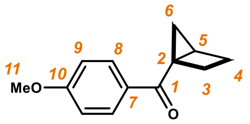

R_f = 0.28 (Hexane:Et_2O = 4:1). Colourless oil.

^{1}H NMR (400 MHz, $CDCl_3$) δ 7.75 (d, *J* = 8.8 Hz, 2H, *2H-8*), 6.91 (d, *J* = 8.9 Hz, 2H, *2H-9*), 3.85 (s, 3H, *3H-11*), 2.64 – 2.51 (m, 2H, *H-3a* + *H-5*), 2.13 (ttd, *J* = 10.8, 4.7, 1.6 Hz, 1H, *H-4a*), 2.03 (dddd, *J* = 10.8, 6.0, 4.4, 1.4 Hz, 1H, *H-3b*), 1.73 (ddt, *J* = 5.9, 3.4, 1.6 Hz, 1H, *H-6a*), 1.50 – 1.39 (m, 2H, *H-4b* + *H-6b*).

^{13}C{H} NMR (101 MHz, $CDCl_3$) δ 200.7 (*C-1*), 162.9 (*C-10*), 130.5 (*2C-8*), 130.2 (*C-7*), 113.6 (*2C-9*), 55.5 (*C-11*), 32.5 (*C-2*), 29.8 (*C-6*), 29.5 (*C-5*), 26.7 (*C-3*), 21.1 (*C-4*).

IR (neat, cm^{-1}): 2934, 1641, 1596, 1418, 1286, 1251 (fingerprint region excluded).

HRMS (APCI+): calculated for $C_{13}H_{15}O_2$ (M+H+): 203.1067 Found: 203.1067.

Synthesis of bicyclo[2.1.0]pentan-1-yl(furan-3-yl)methanone (1q)

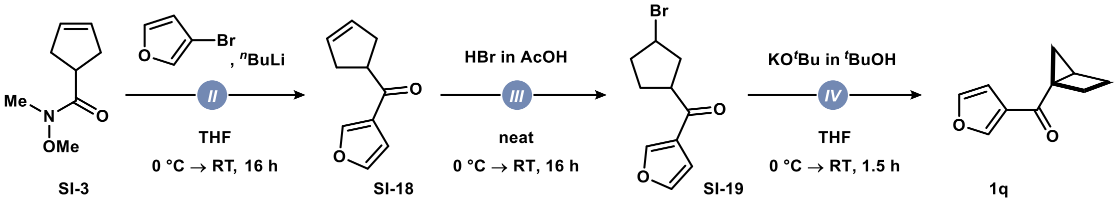

Cyclopent-3-en-1-yl(furan-3-yl)methanone (SI-18)

A flame-dried flask, equipped with a stirrer bar, was cooled under vacuum. After backfilling with N_2 (× 3) THF (25 mL, 0.2 M) and ^{n}BuLi (1.6 M in hexane, 4.7 mL, 7.50 mmol, 1.5 eq) were introduced. The reaction was cooled to -78 °C in a dry ice/acetone bath (15 min), and a solution of 3-bromofuran (0.65 mL, 7.50 mmol, 1.5 eq) was added dropwise over a period of 5 min. The reaction mixture was then stirred vigorously at the same temperature for 1.5 h. A solution of the Weinreb amide **SI-3** in THF (3 mL + 2 mL for rinsing) was added dropwise at -78 °C over a period of 10 min. Then, the resultant reaction mixture was allowed to slowly warm up to room temperature and stirred overnight (16 h) before being quenched with saturated NH_4Cl solution (15 mL). After diluting with water (10 mL) and Et_2O (10 mL), the phases were separated, and the aqueous phase was extracted with Et_2O (3 × 10 mL). The combined organic layers were washed with saturated aqueous NaCl solution (15 mL), dried over anhydrous $MgSO_4$, filtered, and concentrated *in vacuo*. The crude product was purified by column chromatography on silica gel (hexane/ Et_2O = 4:1) to afford the target ketone **SI-18** (632 mg, 3.90 mmol, 78% yield).

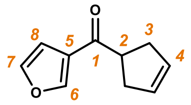

R_f = 0.5. (Hexane:Et_2O = 4:1). Light yellow oil.

^{1}H NMR (500 MHz, $CDCl_3$) δ 8.03 – 7.87 (m, 1H, *H-6*), 7.43 – 7.34 (m, 1H, *H-7*), 6.77 – 6.66 (m, 1H, *H-8*), 5.70 – 5.47 (m, 2H, *2H-4*), 3.61 (tt, *J* = 9.7, 6.3 Hz, 1H, *H-2*), 2.74 – 2.65 (m, 2H, *2H-3a*), 2.63 – 2.54 (m, 2H, *2H-3b*).

^{13}C{H} NMR (126 MHz, $CDCl_3$) δ 196.6 (*C-1*), 147.2 (*C-6*), 144.2 (*C-7*), 129.0 (*C-4*), 127.1 (*C-5*), 109.1 (*C-8*), 46.7 (*C-2*), 36.3 (*C-3*).

IR (neat, cm^{-1}): 1654, 1518, 1413, 1358, 1234, 1220, 1171, 1067 (fingerprint region excluded).

HRMS (APCI+): calculated for $C_{10}H_{11}O_2$ (M+H+): 163.0754 Found: 163.0758.

(3-Bromocyclopentyl)(furan-3-yl)methanone (SI-19)

Prepared according to **GP 2** (step **III**) using **SI-18** (811 mg, 5.00 mmol, 1.0 eq). The crude residue was purified by column chromatography on silica gel (petroleum ether:Et_2O = 4:1 to 3:1) to afford two diastereoisomers of the target bromo-ketone **SI-19** (912 mg, 3.75 mmol, 75% combined yield; d.r. = 1:0.7). The diastereoisomers were partially separable. Therefore, each isomer was separated for NMR analysis.

MAJOR DIASTEREOISOMER

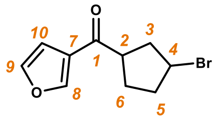

R_f = 0.5 (Hexane: Et_2O = 3:1). Colourless oil.

^{1}H NMR (400 MHz, $CDCl_3$) δ 8.02 – 7.97 (m, 1H, *H-8*), 7.41 – 7.36 (m, 1H, *H-9*), 6.74 – 6.68 (m, 1H, *H-10*), 4.55 (tt, *J* = 5.3, 2.9 Hz, 1H, *H-4*), 3.69 (dtd, *J* = 10.2, 8.1, 5.6 Hz, 1H, *H-2*), 2.47 (ddd, *J* = 14.0, 8.2, 5.5 Hz, 1H, *H-3a*), 2.30 – 2.19 (m, 2H, *H-3b* + *H-6a*), 2.19 – 2.04 (m, 2H, *2H-5*), 1.91 (dddd, *J* = 12.4, 8.2, 5.7, 4.1 Hz, 1H, *H-6b*).

^{13}C{H} NMR (101 MHz, $CDCl_3$) δ 196.3 (*C-1*), 147.6 (*C-8*), 144.4 (*C-9*), 127.3 (*C-7*), 109.0 (*C-10*), 53.8 (*C-4*), 46.5 (*C-2*), 40.8 (*C-3*), 37.6 (*C-5*), 27.9 (*C-6*).

IR (neat, cm^{-1}): 3138, 2970, 2950, 1674, 1561, 1510, 1155 (fingerprint region excluded).

HRMS (APCI+): calculated for $C_{10}H_{12}O_2Br$ (M+H+): 243.0015 Found: 243.0019.

MINOR DIASTEREOISOMER

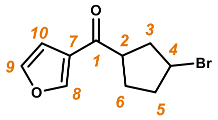

R_f = 0.3 (Hexane: Et_2O = 3:1). Colourless oil.

^{1}H NMR (400 MHz, $CDCl_3$) δ 7.97 – 7.90 (m, 1H, *H-8*), 7.41 – 7.34 (m, 1H, *H-9*), 6.75 – 6.66 (m, 1H, *H-10*), 4.29 – 4.17 (m, 1H, *H-4*), 3.36 – 3.25 (m, 1H, *H-2*), 2.52 – 2.34 (m, 2H, *2H-3*), 2.23 – 2.12 (m, 2H, *H-5a* + *H-6a*), 2.04 (dddd, *J* = 17.0, 10.3, 7.5, 4.3 Hz, 1H, *H-5b*), 1.97 – 1.83 (m, 1H, *H-6b*).

^{13}C{H} NMR (101 MHz, $CDCl_3$) δ 195.0 (*C-1*), 147.3 (*C-8*), 144.4 (*C-9*), 127.1 (*C-7*), 109.1 (*C-10*), 49.0 (*C-4*), 47.6 (*C-2*), 40.1 (*C-3*), 37.8 (*C-5*), 28.1 (*C-6*).

IR (neat, cm^{-1}): 3136, 2970, 1674, 1561, 1510, 1156 (fingerprint region excluded).

HRMS (APCI+): calculated for $C_{10}H_{12}O_2Br$ (M+H+): 243.0015 Found: 243.0019.

Bicyclo[2.1.0]pentan-1-yl(furan-3-yl)methanone (1q)

Prepared according to **GP 2** (step IV) using **SI-19** (243 mg, 1.00 mmol, 1.0 eq). The crude residue was purified by column chromatography on silica gel (hexane:Et_2O = 3:1) to afford the target housane ketone **1q** (143 mg, 0.88 mmol, 88% yield).

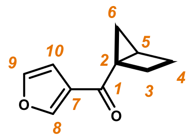

R_f = 0.35 (Hexane:Et_2O = 3:1). Colourless oil.

^{1}H NMR (400 MHz, $CDCl_3$) δ 7.85 (t, J = 1.1 Hz, 1H, *H-8*), 7.39 (t, J = 1.7 Hz, 1H, *H-9*), 6.68 (dd, J = 1.9, 0.8 Hz, 1H, *H-10*), 2.68 – 2.58 (m, 2H, *H-5* + *H-3a*), 2.14 (ttd, J = 11.0, 4.6, 1.6 Hz, 1H, *H-4a*), 1.93 (dddd, J = 10.8, 6.1, 4.3, 1.4 Hz, 1H, *H-3b*), 1.78 (ddt, J = 6.1, 4.2, 1.7 Hz, 1H, *H-6a*), 1.50 – 1.42 (m, 2H, *H-6b* + *H-4b*).
^{13}C{H} NMR (101 MHz, $CDCl_3$) δ 195.0 (*C-1*), 147.3 (*C-8*), 144.4 (*C-9*), 127.1 (*C-7*), 109.1 (*C-10*), 49.0 (*C-4*), 47.6 (*C-2*), 40.1 (*C-3*), 37.8 (*C-5*), 28.1 (*C-6*).
IR (neat, cm^{-1}): 3136, 2970, 1674, 1561, 1510, 1156 (fingerprint region excluded).
HRMS (APCI+): calculated for $C_{10}H_{11}O_2$ (M+H+): 163.0754 Found: 163.0750.

Synthesis of bicyclo[2.1.0]pentan-1-yl)(thiophen-2-yl)methanone (1r)

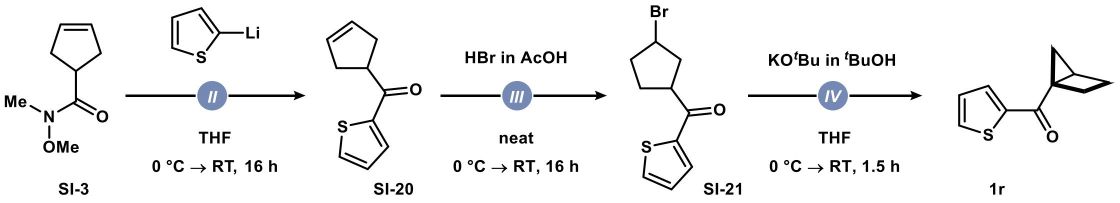

Cyclopent-3-en-1-yl(thiophen-2-yl)methanone (SI-20)

Prepared according to **GP 2** (step II) using 2-thienyllithium solution (1.0 M in THF; 6.5 mL, 6.5 mmol, 1.3 eq). The crude residue was purified by column chromatography on silica gel (hexane:Et_2O = 4:1) to afford the target ketone **SI-20** (624 mg, 3.50 mmol, 70% yield).

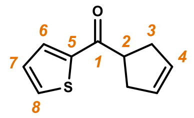

R_f = 0.42. (Hexane:Et_2O = 4:1). Light yellow oil.

^{1}H NMR (500 MHz, $CDCl_3$) δ 7.65 (d, *J* = 4.0 Hz, 1H, *H-8*), 7.56 (d, *J* = 4.9, 1H, *H-6*), 7.07 (dd, *J* = 5.0, 3.8 Hz, 1H, *H-7*), 5.65 – 5.59 (m, 2H, *H-4*), 3.86 (tt, *J* = 9.7, 6.4 Hz, 1H, *H-2*), 2.79 – 2.69 (m, 2H, *H-3a*), 2.69 – 2.58 (m, 2H, *H-3b*).

^{13}C{H} NMR (126 MHz, $CDCl_3$) δ 194.9 (*C-1*), 144.2 (*C-5*), 133.5 (*C-8*), 131.9 (*C-6*), 129.0 (*C-4*), 128.2 (*C-7*), 45.7 (*C-2*), 36.8 (*C-3*).

IR (neat, cm^{-1}): 1654, 1518, 1413, 1358, 1234, 1220, 1171, 1067 (fingerprint region excluded).

HRMS (ESI+): calculated for $C_{10}H_{10}ONaS$ (M+Na$^+$): 201.0345 Found: 201.0342.

(3-Bromocyclopentyl)(thiophen-2-yl)methanone (SI-21)

Prepared according to **GP 2** (step **III**) using **SI-20** (891 mg, 5.00 mmol, 1.0 eq). The crude residue was purified by column chromatography on silica gel (hexane:Et_2O = 6:1) to afford two diastereoisomers of the target bromo-ketone **SI-21** (1.06 g, 4.1 mmol, 82% combined yield; d.r. = 1:1). The diastereoisomers were separable. Therefore, each of the isomers was separated for NMR analysis.

DIASTEREOISOMER 1

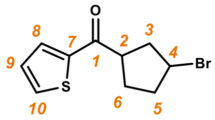

R_f = 0.42. (Hexane:Et_2O = 4:1) Colourless oil.

^{1}H NMR (500 MHz, $CDCl_3$) δ 7.70 – 7.68 (m, 1H, *H-10*), 7.61 – 7.56 (m, 1H, *H-8*), 7.10 – 7.07 (m, 1H, *H-9*), 4.61 – 4.51 (m, 1H, *H-4*), 4.00 – 3.88 (m, 1H, *H-2*), 2.55 – 2.44 (m, 1H, *H-3a*), 2.34 – 2.23 (m, 2H, *H-3b* + *H-5a*), 2.23 – 2.13 (m, 1H, *H-6a*), 2.14 – 2.05 (m, 1H, *H-5b*), 2.00 – 1.90 (m, 1H, *H-6b*).

^{13}C{H} NMR (126 MHz, $CDCl_3$) δ 194.6 (*C-1*), 144.0 (*C-7*), 134.1 (*C-8*), 132.3 (*C-10*), 128.3 (*C-9*), 53.8 (*C-4*), 45.4 (*C-2*), 41.3 (*C-3*), 37.7 (*C-5*), 28.3 (*C-6*).

IR (neat, cm^{-1}): 1649, 1519, 1411, 1369, 1313, 1230, 1219, 1070 (fingerprint region excluded).

HRMS (ESI+): calculated for $C_{10}H_{11}OBrNaS$ (M+Na$^+$): 280.9606 Found: 280.9605.

DIASTEREOISOMER 2

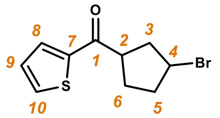

R_f = 0.3. (Hexane:Et_2O = 2:1). Colourless oil.

^{1}H NMR (500 MHz, $CDCl_3$) δ 7.63 (dd, *J* = 3.8, 1.1 Hz, 1H, *H-10*), 7.58 (dd, *J* = 4.9, 1.1 Hz, 1H, *H-8*), 7.07 (dd, *J* = 4.9, 3.8 Hz, 1H, *H-9*), 4.29 – 4.19 (m, 1H, *H-4*), 3.60 – 3.50 (m, 1H, *H-2*), 2.53 (ddd, *J* = 14.0, 8.8, 6.5 Hz, 1H, *H-3a*), 2.41 (dt, *J* = 14.0, 7.9 Hz, 1H, *H-3b*), 2.26 – 2.16 (m, 2H, *H-6a* + *H-5a*), 2.12 – 2.02 (m, 1H, *H-5b*), 2.00 – 1.89 (m, 1H, *H-6b*).

^{13}C{H} NMR (126 MHz, $CDCl_3$) δ 193.3 (*C-1*), 143.8 (*C-7*), 133.9 (*C-8*), 132.0 (*C-10*), 128.3 (*C-9*), 48.9 (*C-4*), 46.6 (*C-2*), 40.7 (*C-3*), 37.9 (*C-5*), 28.6 (*C-6*).

IR (neat, cm^{-1}): 1654, 1516, 1413, 1356, 1306, 1235, 1222, 1058 (fingerprint region excluded).

HRMS (ESI+): calculated for $C_{10}H_{11}OBrNaS$ ($M+Na^+$): 280.9606 Found: 280.9603.

Bicyclo[2.1.0]pentan-1-yl)(thiophen-2-yl)methanone (1r)

Prepared according to **GP 2** (step IV) using **SI-21** (259 mg, 1.00 mmol, 1.0 eq). The crude residue was purified by column chromatography on silica gel (hexane:Et_2O = 6:1) to afford the target housane ketone **1r** (162 mg, 0.86 mmol, 86% yield).

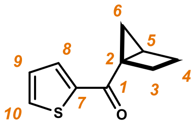

R_f = 0.33. (Hexane:Et_2O = 4:1). Colourless oil.

^{1}H NMR (400 MHz, $CDCl_3$) δ 7.57 – 7.43 (m, 2H, *H-10* + *H-8*), 7.03 (dd, *J* = 4.9, 3.8 Hz, 1H, *H-9*), 2.71 – 2.58 (m, 2H, *H-3a* + *H-5*), 2.11 (ttd, *J* = 10.9, 4.6, 1.6 Hz, 1H, *H-4a*), 1.98 (dddd, *J* = 10.8, 6.1, 4.4, 1.4 Hz, 1H, *H-3b*), 1.77 (ddt, *J* = 5.9, 4.1, 1.7 Hz, 1H, *H-6a*), 1.46 – 1.38 (m, 2H, *H-6b* + *H-4b*).

^{13}C{H} NMR (101 MHz, $CDCl_3$) δ 193.6 (*C-1*), 143.4 (*C-7*), 132.5 (*C-10*), 131.5 (*C-8*), 127.9 (*C-9*), 32.9 (*C-2*), 30.7 (*C-5*), 30.2 (*C-6*), 26.0 (*C-3*), 21.4 (*C-4*).

IR (neat, cm^{-1}): 2935, 1620, 1516, 1412, 1359, 1290, 1260, 1234, 1217, 1199, 1185, 1055, 1011 (fingerprint region excluded).

HRMS (ESI+): calculated for $C_{10}H_{11}OS$ ($M+H^+$): 179.0525 Found: 179.0526.

5.1.2 General Procedure 3 (GP 3):

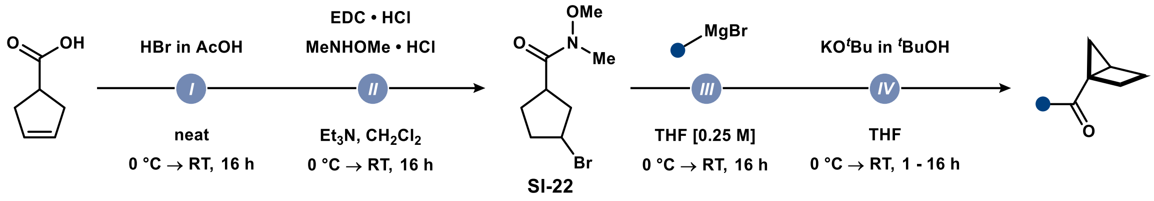

Step I: To an ice-cold solution of 33% HBr in glacial acetic acid (24 mL, 140 mmol, 2.0 eq), cyclopent-3-ene-1-carboxylic acid (7.2 mL, 70.0 mmol, 1.0 eq) was added dropwise. The mixture was gradually warmed to room temperature and stirred vigorously for 16 h, after which it was poured into water (100 mL) and transferred into a separatory funnel with H_2O (20 mL) and EtOAc (40 mL). The phases were separated, and the aqueous phase was extracted with EtOAc (4 × 50 mL). The combined organic phases were washed with saturated aqueous NaCl solution (100 mL), then dried over anhydrous $MgSO_4$, filtered, and concentrated *in vacuo* to afford the crude product, which was used directly without further purification**.**

Step II: A flame-dried flask, equipped with a stirrer bar, was charged with crude product from Step I (70.0 mmol, 1 eq, assuming 100% conversion in Step I) and CH_2Cl_2 (120 mL). The resultant solution was cooled to 0 °C. *N,O*-dimethylhydroxylamine hydrochloride (7.2 g, 73.5 mmol, 1.05 eq) and 1-ethyl-3-(3-dimethylaminopropyl) carbodiimide hydrochloride (14.8 g, 77.0 mmol, 1.1 eq) were successively added, followed by addition of triethylamine (19 mL, 140.0 mmol, 2 eq). The resultant solution was gradually warmed to room temperature and stirred vigorously for 16 h. The reaction mixture was quenched with saturated aqueous NH_4Cl (50 mL), and the phases were separated. The aqueous phase was extracted with Et_2O (3 × 50 mL). The combined organic layers were washed with saturated aqueous NaCl (50 mL), dried over anhydrous $MgSO_4$, filtered, and concentrated *in vacuo*. The resultant crude material was purified by column chromatography on silica gel (hexane:EtOAc = 4:1 to 3:1) to afford the target Weinreb amide **SI-22** (1.30 g, 55.3 mmol, 79% yield, d.r. = 2:1).

3-Bromo-*N*-methoxy-*N*-methylcyclopentane-1-carboxamide (SI-22)

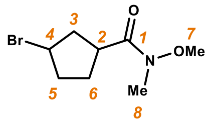

R_f = 0.2. (petroleum ether: EtOAc = 4:1). Colourless oil.

^{1}H NMR (500 MHz, CDCl$_3$) δ 4.54 (tt, *J* = 5.7, 3.0 Hz, 1H, *H-4$_{minor}$*), 4.18 (dt, *J* = 7.5, 6.4 Hz, 1H, *H-4$_{major}$*), 3.66 (s, 3H, *3H-7$_{minor}$*), 3.62 (s, 3H, *3H-7$_{major}$*), 3.56 – 3.42 (m, 1H, *H-2$_{minor}$*), 3.16 – 3.03 (m, 7H, *H-2$_{major}$* + *3H-8$_{major}$* + *3H-8$_{minor}$*), 2.45 (ddd, *J* = 14.6, 8.5, 6.6 Hz, 1H, *H-3a$_{major}$*), 2.36 (ddd, *J* = 14.1, 8.4, 5.5 Hz, 1H, *H-3a$_{minor}$*), 2.27 – 1.99 (m, 7H, *H-3b$_{major}$* + *2H-5$_{major}$* + *H-6a$_{major}$* + *H-3b$_{minor}$* + *H-5a$_{minor}$* + *H-6a$_{minor}$*), 1.90 – 1.75 (m, 3H, *H-6b$_{major}$* + *H-6b$_{minor}$* + *H-5b$_{minor}$*).

^{13}C{H} NMR (126 MHz, CDCl$_3$) δ 176.4 (*C-1$_{minor}$*), 175.4 (*C-1$_{major}$*), 61.4 (*C-7$_{minor}$*), 61.3 (*C-7$_{major}$*), 53.7 (*C-4$_{minor}$*), 48.7 (*C-4$_{major}$*), 41.3 (*C-3$_{minor}$*), 40.7 (*C-3$_{major}$*), 39.5 (*C-2$_{major}$*), 38.2 (*C-2$_{minor}$*), 37.52 (*C-5$_{major}$*), 37.50 (*C-5$_{minor}$*), 32.4 (*C-8$_{major}$* + *C-8$_{minor}$*), 27.9 (*C-6$_{major}$*), 27.8 (*C-6$_{minor}$*).

IR (neat, cm^{-1}): 2967, 1655, 1461, 1384, 1175 (fingerprint region excluded).

HRMS (APCI+): calculated for $C_8H_{15}BrNO_2$ (M+H$^+$): 236.0281 Found: 236.0284.

Step III: A flame-dried flask, equipped with a stirrer bar, was charged with the Weinreb amide **SI-22** from step II (1.18 g, 5.00 mmol, 1.0 eq), and THF (0.25 M). The resultant solution was cooled to 0 °C, and freshly prepared alkyl magnesium bromide (1.5 eq) was added dropwise via cannula. The resultant solution was gradually warmed to room temperature and allowed to stir for 16 h. The reaction mixture was quenched with saturated aqueous NH_4Cl (20 mL), phases were separated, and the aqueous phase was extracted with Et_2O (3 × 25 mL). The combined organic layers were washed with saturated aqueous NaCl (30 mL), dried over anhydrous $MgSO_4$, filtered, and concentrated *in vacuo*. The crude product was purified by column chromatography on silica gel to afford the target product ketone.

Step IV: A flame-dried flask, equipped with a stirrer bar, was charged with the bromoketone from step III (1.00 mmol, 1.0 eq) and THF (0.2 M). The resultant solution was cooled to 0 °C under nitrogen atmosphere. A 1 M solution of KO^{t}Bu in ^{t}BuOH (6 mL, 6.00 mmol,1.2 eq) was added dropwise, and the reaction mixture was gradually warmed to room temperature. The progress of the reaction was monitored by TLC analysis. Upon completion, the reaction mixture was quenched with saturated aqueous NH_4Cl (15 mL), diluted with water and EtOAc, the phases were separated, and the aqueous phase was extracted with EtOAc (3 × 20 mL). The combined organic layers were washed with saturated

aqueous NaCl (20 mL), dried over anhydrous $MgSO_4$, filtered, and concentrated *in vacuo*. The resultant crude material was purified by column chromatography on silica gel to afford the target product housane ketone.

Synthesis of 1-(bicyclo[2.1.0]pentan-1-yl)-3-cyclohexylpropan-1-one (1c)

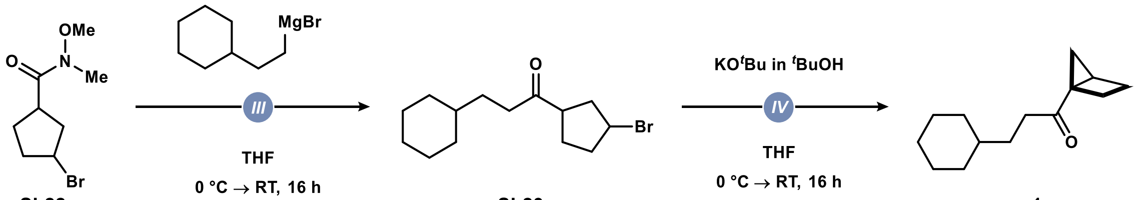

SI-22 **SI-23** **1c**

1-(3-Bromocyclopentyl)-3-cyclohexylpropan-1-one (SI-23)

Prepared according to **GP 3** (step **III**) using (2-cyclohexylethyl)magnesium bromide (0.51 M in THF; 14.7 mL, 7.50 mmol, 1.5 eq). The crude residue was purified by column chromatography on silica gel (hexane:Et_2O = 19:1 to 9:1) to afford two diastereoisomers of the target bromo-ketone **SI-23** (1.03 g, 3.60 mmol, 72% combined yield; d.r. = 1:0.7). The diastereoisomers were partially separable. Therefore, each isomer was separated for NMR analysis.

MINOR DIASTEREOISOMER

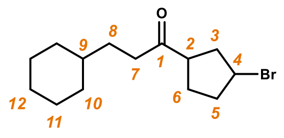

R_f = 0.8. (Hexane:Et_2O = 2:1). Colourless oil.

^{1}H NMR (400 MHz, $CDCl_3$) δ 4.53 – 4.45 (m, 1H, *H-4*), 3.28 (qd, *J* = 8.4, 6.0 Hz, 1H, *H-2*), 2.41 (q, *J* = 7.5 Hz, 2H, 2*H-7*), 2.27 (ddd, *J* = 14.1, 8.5, 5.4 Hz, 1H, *H-3a*), 2.21 – 1.99 (m, 4H, *H-3b* + 2*H-5* + *H-6a*), 1.82 – 1.71 (m, 1H, *H-6b*), 1.68 – 1.53 (m, 5H, *2CH₂* + *H-12a*), 1.40 (q, *J* = 7.5 Hz, 2H, 2*H-8*), 1.21 – 1.03 (m, 4H, *H-9* + CH_2 + *H-12b*), 0.87 – 0.76 (m, 2H, CH_2).

^{13}C{H} NMR (101 MHz, $CDCl_3$) δ 212.5 (*C-1*), 53.6 (*C-4*), 48.8 (*C-2*), 40.4 (*C-7*), 40.0 (*C-3*), 37.5 (*C-5* + *C-9*), 33.3 (CH_2), 33.3 (CH_2), 31.3 (*C-8*), 26.7 (CH_2), 26.7 (*C-6*), 26.4 ($2CH_2$).

IR (neat, cm^{-1}): 2930, 2836, 1706, 1459, 1360, 1299, 1241, 1111 (fingerprint region excluded).

HRMS (ESI+): calculated for $C_{14}H_{23}OBrNa$ ($M+Na^+$): 309.0824 Found: 309.0823.

MAJOR DIASTEREOISOMER

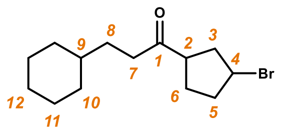

R_f = 0.76 (Hexane:Et_2O = 2:1). Colourless oil.

^{1}H NMR (400 MHz, $CDCl_3$) δ 4.21 (p, *J* = 6.2 Hz, 1H, *H-4*), 2.84 (tt, *J* = 9.0, 7.6 Hz, 1H, *H-2*), 2.47 – 2.33 (m, 3H, *H-3a* + 2*H-7*), 2.24 (ddd, *J* = 14.3, 8.1, 6.6 Hz, 1H, *H-3b*), 2.16 – 1.93 (m, 3H, 2*H-5* + *H-6a*), 1.89 – 1.77 (m, 1H, *H-6b*), 1.66 – 1.53 (m, 5H, 2 CH_2 + *H-12a*), 1.41 (q, *J* = 7.4 Hz, 2H, 2*H-8*), 1.21 – 1.00 (m, 4H, *H-9* + CH_2 + *H-12b*), 0.88 – 0.75 (m, 2H, CH_2).

^{13}C{H} NMR (101 MHz, $CDCl_3$) δ 211.1 (*C-1*), 50.1 (*C-4*), 49.6 (*C-2*), 39.6 (*C-7*), 39.0 (*C-3*), 37.7 (*C-5*), 37.4 (*C-9*), 33.3 (CH_2), 33.3 (CH_2), 31.3 (*C-8*), 26.9 (CH_2), 26.7 (*C-6*), 26.4 (2CH_2).

IR (neat, cm^{-1}): 2920, 2849, 1708, 1448, 1367, 1308, 1229, 1105 (fingerprint region excluded).

HRMS (ESI+): calculated for $C_{14}H_{23}OBrNa$ (M+Na^+): 309.0824 Found: 309.0821.

1-(Bicyclo[2.1.0]pentan-1-yl)-3-cyclohexylpropan-1-one (1c)

Prepared according to **GP 3** (step IV) using **SI-23** (287 mg, 1.00 mmol, 1.0 eq). The crude residue was purified by column chromatography on silica gel (hexane:Et_2O = 4:1) to afford the target housane ketone **1c** (179.5 mg, 0.87 mmol, 87% yield).

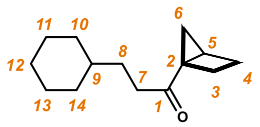

R_f = 0.75 (Hexane:Et_2O = 2:1). Colourless oil.

^{1}H NMR (500 MHz, $CDCl_3$) δ 2.44 (tdt, *J* = 11.2, 4.3, 1.4 Hz, 1H, *H-3a*), 2.35 – 2.24 (m, 1H, *H-5*), 2.20 – 1.95 (m, 3H, 2*H-7* + *H-4a*), 1.68 (tt, *J* = 4.8, 1.4 Hz, 1H, *H-6a*), 1.66 – 1.49 (m, 6H, *H-10a* + *H-11a* + *H-12a* + *H-13a* + *H-14a* + *H-3b*), 1.44 – 1.28 (m, 3H, *H-4b* + 2*H-8*), 1.27 – 1.22 (m, 1H, *H-6b*), 1.20 – 0.99 (m, 4H, *H-9* + *H-11b* + *H-13b* + *H-12b*), 0.87 – 0.73 (m, 2H, *H-10b* + *H-14b*).

^{13}C{H} NMR (126 MHz, $CDCl_3$) δ 209.8 (*C-1*), 37.5 (*C-9*), 34.7 (*C-7*), 34.3 (*C-2*), 33.3 (*C-10*), 33.2 (*C-14*), 31.6 (*C-8*), 30.0 (*C-5*), 26.7 (*C-6*), 26.4 (*C-11* + *C-13*), 26.3 (*C-12*), 22.5 (*C-3*), 20.6 (*C-4*).

IR (neat, cm^{-1}): 2920, 2850, 1672, 1448, 1383, 1252, 1135, 1049, 1035, 1012 (fingerprint region excluded).

HRMS (ESI+): calculated for $C_{14}H_{22}ONa$ (M+Na^+): 229.1563 Found: 229.1560.

Synthesis of 1-(bicyclo[2.1.0]pentan-1-yl)-3-(3,4-dimethoxyphenyl)propan-1-one (1e)

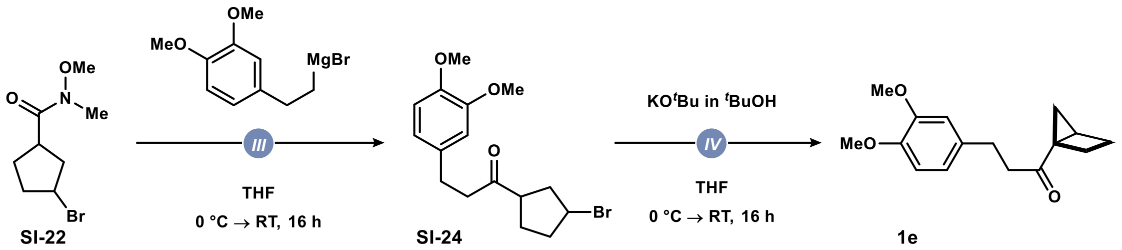

1-(3-Bromocyclopentyl)-3-(3,4-dimethoxyphenyl)propan-1-one (SI-24)

Prepared according to **GP 3** (step **III**) using (3,4-dimethoxyphenethyl)magnesium bromide (0.65 M in THF; 11.5 mL, 7.50 mmol, 1.5 eq). The crude residue was purified by column chromatography on silica gel (hexane:Et_2O = 2:1 to 1:1).

NOTE: The minor diastereoisomer of the bromo-ketone **SI-24** could not be separated from the unreacted Weinreb amide **SI-22**. Therefore, we proceeded further with the major diastereoisomer of the target bromo-ketone **SI-24** (478 mg, 1.40 mmol, 28% yield).

MAJOR DIASTEREOISOMER

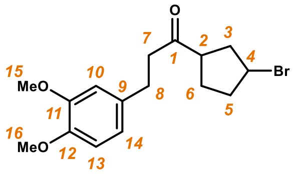

R_f = 0.22 (Hexane:Et_2O = 1:1). Colourless oil.

^{1}H NMR (400 MHz, $CDCl_3$) δ 6.75 – 6.69 (m, 1H, *H-13*), 6.68 – 6.61 (m, 2H, *H-10* + *H-14*), 4.20 (p, *J* = 6.1 Hz, 1H, *H-4*), 3.80 (s, 3H, 3*H-15*), 3.78 (s, 3H, 3*H-16*), 2.86 – 2.74 (m, 3H, 2*H-8* + *H-2*), 2.74 – 2.63 (m, 2H, 2*H-7*), 2.36 (ddd, *J* = 13.8, 9.1, 6.4 Hz, 1H, *H-3a*), 2.23 (ddd, *J* = 14.2, 7.9, 6.3 Hz, 1H, *H-3b*), 2.12 – 1.92 (m, 3H, 2*H-5* + *H-6a*), 1.88 – 1.73 (m, 1H, *H-6b*).

^{13}C{H} NMR (101 MHz, $CDCl_3$) δ 209.7 (*C-1*), 149.0 (*C-11*), 147.5 (*C-12*), 133.9 (*C-9*), 120.3 (*C-10*), 111.9 (*C-14*), 111.5 (*C-13*), 56.1 (*C-15*), 56.0 (*C-16*), 50.4 (*C-2*), 49.7 (*C-4*), 43.4 (*C-7*), 39.3 (*C-3*), 37.8 (*C-5*), 29.6 (*C-8*), 26.8 (*C-6*).

IR (neat, cm^{-1}): 2934, 1707, 1590, 1513, 1463, 1450, 1258, 1233, 1154, 1140, 1026 (fingerprint region excluded).

HRMS (ESI+): calculated for $C_{16}H_{21}O_3BrNa$ (M+Na$^+$): 363.0566 Found: 363.0563.

1-(Bicyclo[2.1.0]pentan-1-yl)-3-(3,4-dimethoxyphenyl)propan-1-one (1e)

Prepared according to **GP 3** (step IV) using **SI-24** (341 mg, 1.00 mmol, 1.0 eq). The crude residue was purified by column chromatography on silica gel (hexane:Et_2O = 1:1) to afford the target housane ketone **1e** (221 mg, 0.85 mmol, 85% yield).

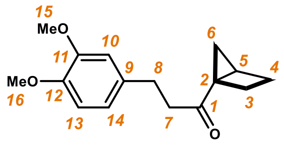

R_f = 0.23 (Hexane:Et_2O = 1:1). Colourless oil.

^{1}H NMR (500 MHz, $CDCl_3$) δ 6.73 – 6.69 (m, 1H, *H-13*), 6.65 – 6.61 (m, 2H, *H-14* + *H-10*), 3.79 (s, 3H, 3*H-15*), 3.78 (s, 3H, 3*H-16*), 2.82 – 2.68 (m, 2H, 2*H-8*), 2.48 – 2.38 (m, 2H, *H-7a* + *H-3a*), 2.34 (ddd, *J* = 16.3, 8.9, 6.6 Hz, 1H, *H-7b*), 2.25 (tdd, *J* = 6.1, 2.7, 1.3 Hz, 1H, *H-5*), 1.96 (ttd, *J* = 11.0, 4.7, 1.5 Hz, 1H, *H-4a*), 1.65 (ddt, *J* = 6.4, 4.7, 1.9 Hz, 1H, *H-6a*), 1.53 (dddd, *J* = 11.2, 6.2, 4.5, 1.4 Hz, 1H, *H-3b*), 1.31 (dddd, *J* = 11.0, 6.5, 4.2, 1.1 Hz, 1H, *H-4b*), 1.24 (dd, *J* = 4.7, 2.9 Hz, 1H, *H-6b*).

^{13}C{H} NMR (126 MHz, $CDCl_3$) δ 208.3 (*C-1*), 148.9 (*C-11*), 147.4 (*C-12*), 134.1 (*C-9*), 120.2 (*C-14*), 111.8 (*C-10*), 111.4 (*C-13*), 56.0 (*C-15*), 55.9 (*C-16*), 39.1 (*C-7*), 34.4 (*C-2*), 30.1 (*C-5*), 29.9 (*C-8*), 26.3 (*C-6*), 22.3 (*C-3*), 20.5 (*C-4*).

IR (neat, cm^{-1}): 2934, 1668, 1513, 1463, 1451, 1382, 1256, 1234, 1153, 1137, 1026 (fingerprint region excluded).

HRMS (ESI+): calculated for $C_{16}H_{21}O_3$ (M+H$^+$): 261.1485 Found: 261.1484.

Synthesis of 1-(bicyclo[2.1.0]pentan-1-yl)-3-(1-methyl-1*H*-indol-3-yl)propan-1-one (1f)

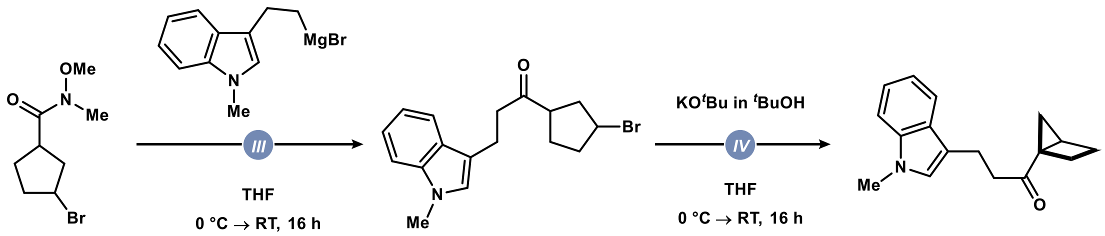

SI-22 SI-25 1f

1-(3-Bromocyclopentyl)-3-(1-methyl-1*H*-indol-3-yl)propan-1-one (SI-25)

Prepared according to **GP 3** (step III) using (2-(1-methyl-1*H*-indol-3-yl)ethyl)magnesium bromide (0.45 M in THF; 16.7 mL, 7.50 mmol, 1.5 eq). The crude residue was purified by column chromatography on

silica gel (hexane:Et_2O = 9:1) to afford two diastereoisomers of the target bromo-ketone **SI-25** (401 mg, 1.20 mmol, 24% combined yield; d.r. = 1:0.8). The diastereoisomers were partially separable. Therefore, each isomer was separated for NMR analysis.

MINOR DIASTEREOISOMER

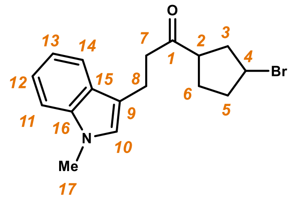

R_f = 0.8. (Hexane:Et_2O = 3:2). Light-yellow oil.

^{1}H NMR (400 MHz, $CDCl_3$) δ 7.49 (d, *J* = 7.9 Hz, 1H, *H-14*), 7.21 (d, *J* = 8.2 Hz, 1H, *H-11*), 7.15 (ddd, *J* = 8.3, 6.8, 1.2 Hz, 1H, *H-12*), 7.03 (ddd, *J* = 8.0, 6.8, 1.1 Hz, 1H, *H-13*), 6.76 (s, 1H, *H-10*), 4.44 (tt, *J* = 5.4, 2.9 Hz, 1H, *H-4*), 3.66 (s, 3H, 3*H-17*), 3.30 – 3.18 (m, 1H, *H-2*), 2.98 (t, *J* = 7.4 Hz, 2H, 2*H-8*), 2.88 – 2.71 (m, 2H, 2*H-7*), 2.30 – 2.21 (m, 1H, *H-3a*), 2.21 – 1.93 (m, 4H, *H-3b* + 2*H-5* + *H-6a*), 1.79 – 1.66 (m, 1H, *H-6b*).

^{13}C{H} NMR (101 MHz, $CDCl_3$) δ 211.6 (*C-1*), 137.1 (*C-16*), 127.6 (*C-15*), 126.5 (*C-10*), 121.8 (*C-13*), 118.9 (*C-12* + *C-14*), 113.8 (*C-9*), 109.4 (*C-11*), 53.4 (*C-2*), 49.1 (*C-4*), 43.1 (*C-7*), 40.1 (*C-3*), 37.4 (*C-5*), 32.7 (*C-17*), 26.5 (*C-6*), 19.4 (*C-8*).

IR (neat, cm^{-1}): 2923, 1708, 1483, 1472, 1374, 1154, 1012 (fingerprint region excluded).

HRMS (ESI+): calculated for $C_{17}H_{21}ONBr$ ($M+Na^+$): 334.0801 Found: 334.0799.

MAJOR DIASTEREOISOMER

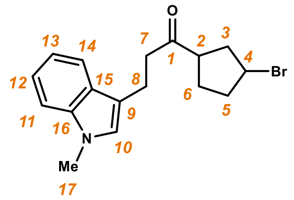

R_f = 0.75. (Hexane:Et_2O = 3:2). Light-yellow oil.

^{1}H NMR (400 MHz, $CDCl_3$) δ 7.49 (dt, *J* = 7.9, 1.0 Hz, 1H, *H-14*), 7.20 (dt, *J* = 8.2, 1.0 Hz, 1H, *H-11*), 7.14 (ddd, *J* = 8.2, 6.9, 1.2 Hz, 1H, *H-12*), 7.03 (ddd, *J* = 8.0, 6.9, 1.2 Hz, 1H, *H-13*), 6.76 (s, 1H, *H-10*), 4.17 (p, *J* = 6.2 Hz, 1H, *H-4*), 3.65 (s, 3H, 3*H-17*), 2.98 (t, *J* = 7.4 Hz, 2H, 2*H-8*), 2.85 – 2.70 (m, 3H, 2*H-7* + *H-2*), 2.32 (ddd, *J* = 13.9, 9.0, 6.4 Hz, 1H, *H-3a*), 2.22 (ddd, *J* = 14.2, 7.9, 6.4 Hz, 1H, *H-3b*), 2.10 – 1.89 (m, 3H, 2*H-5* + *H-6a*), 1.84 – 1.70 (m, 1H, *H-6b*).

^{13}C{H} NMR (101 MHz, $CDCl_3$) δ 210.3 (*C-1*), 137.1 (*C-16*), 127.6 (*C-15*), 126.6 (*C-10*), 121.7 (*C-13*), 118.8 (*C-12* + *C-14*), 113.8 (*C-9*), 109.4 (*C-11*), 50.3 (*C-2*), 49.7 (*C-4*), 42.3 (*C-7*), 39.3 (*C-3*), 37.7 (*C-5*), 32.7 (*C-17*), 26.7 (*C-6*), 19.4 (*C-8*).

IR (neat, cm^{-1}): 2927, 1709, 1490, 1465, 1380, 1149, 1001 (fingerprint region excluded).

HRMS (ESI+): calculated for $C_{17}H_{21}ONBr$ (M+Na$^+$): 334.0801 Found: 334.0803.

1-(Bicyclo[2.1.0]pentan-1-yl)-3-(1-methyl-1*H*-indol-3-yl)propan-1-one (1f)

Prepared according to **GP 3** (step IV) using **SI-25** (334 mg, 1.00 mmol, 1.0 eq). The crude residue was purified by column chromatography on silica gel (hexane:Et_2O = 2:1) to afford the target housane ketone **1f** (228 mg, 0.90 mmol, 90% yield).

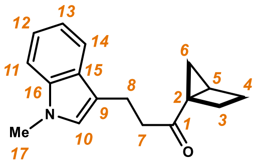

R_f = 0.32. (Hexane:Et_2O = 2:1). Light-yellow oil.

^{1}H NMR (500 MHz, $CDCl_3$) δ 7.48 (dt, *J* = 7.8, 1.0 Hz, 1H, *H-14*), 7.23 – 7.20 (m, 1H, *H-11*), 7.15 (ddd, *J* = 8.2, 6.9, 1.2 Hz, 1H, *H-12*), 7.03 (ddd, *J* = 7.8, 6.8, 1.0 Hz, 1H, *H-13*), 6.77 (s, 1H, *H-10*), 3.66 (s, 3H, 3*H-17*), 3.00 – 2.88 (m, 2H, 2*H-8*), 2.54 (ddd, *J* = 16.5, 8.6, 6.5 Hz, 1H, *H-7a*), 2.48 – 2.39 (m, 2H, *H-7b*+ *H-3a*), 2.23 (dddd, *J* = 6.0, 4.3, 2.8, 1.3 Hz, 1H, *H-5*), 1.93 (ttd, *J* = 11.0, 4.7, 1.5 Hz, 1H, *H-4a*), 1.66 (ddt, *J* = 6.4, 4.7, 1.7 Hz, 1H, *H-6a*), 1.52 (dddd, *J* = 11.2, 6.2, 4.5, 1.4 Hz, 1H, *H-3b*), 1.29 (dddd, *J* = 11.0, 6.6, 4.2, 1.1 Hz, 1H, *H-4b*), 1.22 (dd, *J* = 4.7, 2.9 Hz, 1H, *H-6b*).

^{13}C{H} NMR (126 MHz, $CDCl_3$) δ 208.9 (*C-1*), 137.1 (*C-16*), 127.7 (*C-15*), 126.5 (*C-10*), 121.7 (*C-13*), 119.0 (*C-12*), 118.8 (*C-14*), 114.2 (*C-9*), 109.3 (*C-11*), 38.1(*C-7*), 34.5 (*C-2*), 32.7 (*C-17*), 30.1 (*C-5*), 26.4 (*C-6*), 22.4 (*C-3*), 20.6 (*C-4*), 19.7 (*C-8*).

IR (neat, cm^{-1}): 2934, 1671, 1483, 1472, 1376, 1324, 1250, 1132 (fingerprint region excluded).

HRMS (ESI+): calculated for $C_{17}H_{19}ONNa$ (M+Na$^+$): 276.1359 Found: 276.1359.

Synthesis of 1-(bicyclo[2.1.0]pentan-1-yl)-4-(4,5-diphenyloxazol-2-yl)butan-1-one (1g)

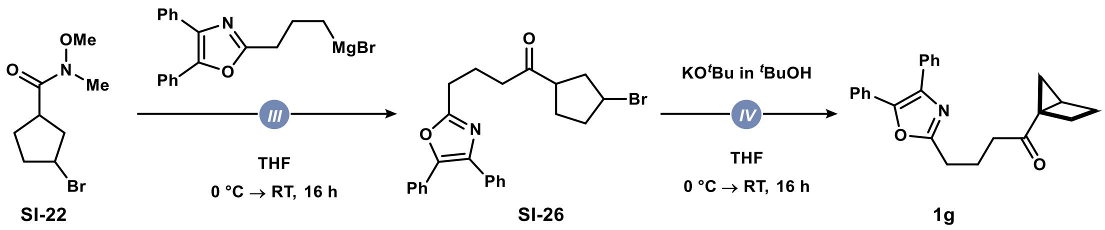

1-(3-Bromocyclopentyl)-4-(4,5-diphenyloxazol-2-yl)butan-1-one (SI-26)

Prepared according to **GP 3** (step III) using (3-(4,5-diphenyloxazol-2-yl)propyl)magnesium bromide (0.52 M in THF; 14.4 mL, 7.50 mmol, 1.5 eq). The crude residue was purified by column chromatography on silica gel (hexane:Et_2O = 3:2) to afford two diastereoisomers of the target bromo-ketone **SI-26** (658 mg, 1.5 mmol, 30% combined yield; d.r. = 1:0.7). The diastereoisomers were partially separable. Therefore, each isomer was separated for NMR analysis.

MINOR DIASTEREOISOMER

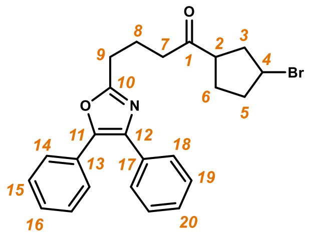

R_f = 0.48. (Hexane:Et_2O = 1:1). Light-yellow oil.

^{1}H NMR (400 MHz, $CDCl_3$) δ 7.66 – 7.61 (m, 2H, 2*H-18*), 7.60 – 7.55 (m, 2H, 2*H-14*), 7.40 – 7.29 (m, 6H, 2*H-15* + *H-16* + 2*H-19* + *H-20*), 4.54 (tt, *J* = 5.3, 2.9 Hz, 1H, *H-4*), 3.42 – 3.30 (m, 1H, *H-2*), 2.89 (t, *J* = 7.2 Hz, 2H, *2H-9*), 2.77 – 2.58 (m, 2H, *2H-7*), 2.36 (ddd, *J* = 14.1, 8.5, 5.4 Hz, 1H, *H-3a*), 2.27 – 2.08 (m, 6H, *H-3b* + *H-6a* + *2H-5* + *2H-8*), 1.91 – 1.80 (m, 1H, *H-6b*).

^{13}C{H} NMR (101 MHz, $CDCl_3$) δ 211.0 (*C-1*), 162.9 (*C-10*), 145.5 (*C-11*), 135.2 (*C-12*), 132.6 (*C-13*), 129.2 (*C-17*), 128.8 (*C-15*), 128.7 (*C-19*), 128.6 (*C-16*), 128.2 (*C-20*), 128.0 (*C-14*), 126.6 (*C-18*), 53.5 (*C-4*), 48.9 (*C-2*), 41.1 (*C-7*), 40.2 (*C-3*), 37.4 (*C-5*), 27.5 (*C-9*), 26.7 (*C-6*), 21.1 (*C-8*).

IR (neat, cm^{-1}): 1709, 1570, 1445, 1218, 1059, 1025 (fingerprint region excluded).

HRMS (ESI+): calculated for $C_{24}H_{24}O_2NBrNa$ (M+Na$^+$): 460.0883 Found: 460.0890.

MAJOR DIASTEREOISOMER

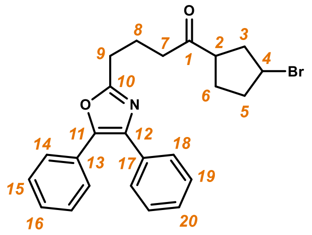

R_f = 0.32. (Hexane:Et_2O = 1:1). Light-yellow oil.

^{1}H NMR (400 MHz, $CDCl_3$) δ 7.68 – 7.61 (m, 2H, m, 2H, 2*H-18*), 7.61 – 7.54 (m, 2H, 2*H-14*), 7.41 – 7.28 (m, 6H, 2*H-15* + *H-16* + 2*H-19* + *H-20*), 4.27 (p, *J* = 6.1 Hz, 1H, *H-4*), 2.98 – 2.85 (m, 3H, *H-2* + 2*H-9*), 2.66 (td, *J* = 7.1, 2.6 Hz, 2H, *2H-7*), 2.46 (ddd, *J* = 14.1, 9.0, 6.4 Hz, 1H, *H-3a*), 2.34 (ddd, *J* = 14.2, 7.9, 6.4 Hz, 1H, *H-3b*), 2.21 – 2.10 (m, 4H, *2H-5* + *2H-8*), 2.10 – 2.03 (m, 1H, *H-6a*), 1.97 – 1.86 (m, 1H, *H-6b*).

^{13}C{H} NMR (101 MHz, $CDCl_3$) δ 209.7 (*C-1*), 162.9 (*C-10*), 145.4 (*C-11*), 135.2 (*C-12*), 132.7 (*C-13*), 129.2 (*C-17*), 128.8 (*C-15*), 128.7 (*C-19*), 128.6 (*C-16*), 128.2 (*C-20*), 128.0 (*C-14*), 126.6 (*C-18*), 50.2 (*C-4*), 49.6 (*C-2*), 40.2 (*C-7*), 39.5 (*C-3*), 37.7 (*C-5*), 27.5 (*C-9*), 26.9 (*C-6*), 21.2 (*C-8*).

IR (neat, cm^{-1}): 1708, 1562, 1449, 1210, 1064, 1029 (fingerprint region excluded).

HRMS (ESI+): calculated for $C_{24}H_{24}O_2NBrNa$ (M+Na$^+$): 460.0883 Found: 460.0882.

1-(Bicyclo[2.1.0]pentan-1-yl)-4-(4,5-diphenyloxazol-2-yl)butan-1-one (1g)

Prepared according to **GP 3** (step IV) using **SI-26** (438 mg, 1.00 mmol, 1.0 eq). The crude residue was purified by column chromatography on silica gel (hexane:Et_2O = 3:1) to afford the target housane ketone **1g** (300 mg, 0.84 mmol, 84% yield).

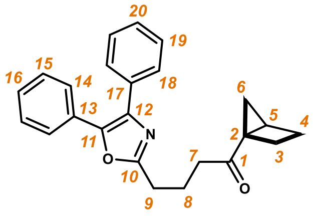

R_f = 0.6. (Hexane:Et_2O = 2:1). Light-yellow oil.

^{1}H NMR (400 MHz, $CDCl_3$) δ 7.59 – 7.54 (m, 2H, 2*H-18*), 7.53 – 7.48 (m, 2H, 2*H-14*), 7.33 – 7.21 (m, 6H, 2*H-15* + *H-16* + 2*H-19* + *H-20*), 2.79 (t, *J* = 7.2 Hz, 2H, 2*H-9*), 2.45 (tdd, *J* = 11.1, 4.2, 1.7 Hz, 1H, *H-3a*), 2.39 – 2.28 (m, 2H, *H-7a* + *H-5*), 2.23 (dt, *J* = 16.6, 7.2 Hz, 1H, *H-7b*), 2.10 – 1.94 (m, 3H, 2*H-8* + *H-4a*), 1.71 (ddt, *J* = 6.3, 4.6, 1.6 Hz, 1H, *H-6a*), 1.58 – 1.50 (m, 1H, *H-3b*), 1.36 – 1.28 (m, 1H, *H-4b*), 1.26 (dd, *J* = 4.7, 2.9 Hz, 1H, *H-6b*).

^{13}C{H} NMR (101 MHz, $CDCl_3$) δ 208.2 (*C-1*), 163.0 (*C-10*), 145.4 (*C-11*), 135.1 (*C-12*), 132.7 (*C-13*), 129.2 (*C-17*), 128.8 (*C-15*), 128.7 (*C-19*), 128.5 (*C-16*), 128.1 (*C-20*), 128.0 (*C-14*), 126.6 (*C-18*), 35.9 (*C-7*), 34.4 (*C-2*), 30.3 (*C-5*), 27.6 (*C-9*), 26.4 (*C-6*), 22.3 (*C-3*), 21.4 (*C-8*), 20.6 (*C-4*).

IR (neat, cm^{-1}): 2935, 1672, 1570, 1445, 1385, 1217, 1059, 1024 (fingerprint region excluded).

Synthesis of 1-(bicyclo[2.1.0]pentan-1-yl)hex-5-en-1-one (1h)

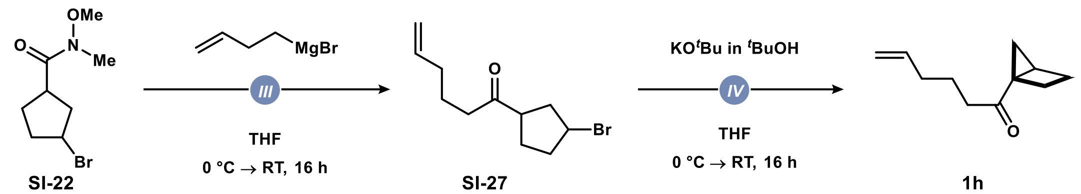

SI-22 SI-27 1h

1-(3-Bromocyclopentyl)hex-5-en-1-one (SI-27)

Prepared according to **GP 3** (step III) using 4-pentenylmagnesium bromide (0.50 M in THF; 15.0 mL, 7.50 mmol, 1.5 eq). The crude residue was purified by column chromatography on silica gel (hexane:Et_2O = 9:1) to afford two diastereoisomers of the target bromo-ketone **SI-27** (1.08 g, 4.40 mmol, 88% combined yield; d.r. = 1:0.4).

MIXTURE OF DIASTEREOISOMERS

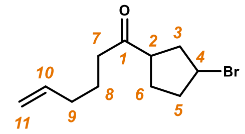

R_f = 0.3 (Hexane:Et_2O = 9:1). Colourless oil.

^{1}H NMR (400 MHz, $CDCl_3$) δ 5.80 – 5.59 (m, 1H, *H-10$_{major}$* + *H-10$_{minor}$*), 5.04 – 4.82 (m, 3H, *2H-11$_{major}$* + *2H-11$_{minor}$*), 4.53 – 4.45 (m, 1H, *H-4$_{minor}$*), 4.26 – 4.16 (m, 1H, *H-4$_{major}$*), 3.32 – 3.22 (m, 1H, *H-2$_{minor}$*), 2.89 – 2.77 (m, 1H, *H-2$_{major}$*), 2.50 – 2.33 (m, 6H, *2H-7$_{major}$* + *H-3a$_{major}$* + *2H-7$_{minor}$* + *H-3a$_{minor}$*), 2.32 – 2.21 (m, 2H, *H-3b$_{major}$* + *H-3b$_{minor}$*), 2.20 – 1.94 (m, 10H, *2H-4$_{major}$* + *H-6a$_{major}$* + *2H-9$_{major}$* + *2H-4$_{minor}$* + *H-6a$_{minor}$* + *2H-9$_{minor}$*), 1.90 – 1.72 (m, 2H, *H-6b$_{major}$* + *H-6b$_{minor}$*), 1.70 – 1.58 (m, 4H, *2H-8$_{major}$* + *2H-8$_{minor}$*).

^{13}C{H} NMR (101 MHz, $CDCl_3$) δ 211.9 (*C-1$_{minor}$*), 210.5 (*C-1$_{major}$*), 138.11 (*C-10$_{major}$*), 138.06 (*C-10$_{minor}$*), 115.44 (*C-11$_{minor}$*), 115.40 (*C-11$_{major}$*), 53.6 (*C-4$_{minor}$*), 50.2 (*C-4$_{major}$*), 49.6 (*C-2$_{major}$*), 48.9 (*C-2$_{minor}$*), 41.5 (*C-7$_{minor}$*), 40.5 (*C-7$_{major}$*), 40.3 (*C-3$_{minor}$*), 39.5 (*C-3$_{major}$*), 37.7 (*C-5$_{major}$*), 37.4 (*C-5$_{minor}$*), 33.2 (*C-9$_{major}$* + *C-9$_{minor}$*), 26.9 (*C-6$_{major}$*), 26.7 (*C-6$_{minor}$*), 22.8 (*C-8$_{major}$*), 22.8 (*C-8$_{minor}$*).

IR (neat, cm^{-1}): 2941, 2253, 1708, 1439, 1369, 1219 (fingerprint region excluded).

HRMS (APCI+): calculated for $C_{11}H_{18}OBr$ (M+H+): 245.0536 Found: 245.0536.

1-(Bicyclo[2.1.0]pentan-1-yl)hex-5-en-1-one (1h)

Prepared according to **GP 3** (step IV) using **SI-27** (245 mg, 1.00 mmol, 1.0 eq). The crude residue was purified by column chromatography on silica gel (hexane:Et_2O = 4:1) to afford the target housane ketone **1c** (120 mg, 0.73 mmol, 73% yield).

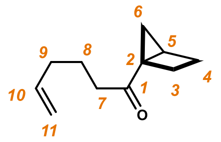

R_f = 0.3 (Hexane: Et_2O = 9:1). Colourless oil.

^{1}H NMR (500 MHz, $CDCl_3$) δ 5.69 (ddt, *J* = 16.9, 10.2, 6.7 Hz, 1H, *H-10*), 5.00 – 4.86 (m, 2H, *2H-11*), 2.45 (tdd, *J* = 11.2, 4.3, 1.7 Hz, 1H, *H-3a*), 2.30 (dddd, *J* = 6.1, 4.4, 2.8, 1.3 Hz, 1H, *H-5*), 2.15 (ddd, *J* = 16.3, 8.1, 6.7 Hz, 1H, *H-7a*), 2.11 – 1.94 (m, 4H, *H-7b* + *H-4a* + *2H-9*), 1.68 (ddt, *J* = 6.3, 4.6, 1.6 Hz, 1H, *H-6a*), 1.64 – 1.51 (m, 3H, *2H-8* + *H-3b*), 1.37 – 1.30 (m, 1H, *H-4b*), 1.25 (dd, *J* = 4.7, 2.8 Hz, 1H, *H-6b*).

^{13}C{H} NMR (126 MHz, $CDCl_3$) δ 209.1 (*C-1*), 138.3 (*C-10*), 115.2 (*C-11*), 36.2 (*C-7*), 34.4 (*C-2*), 33.3 (*C-9*), 30.0 (*C-5*), 26.3 (*C-6*), 23.2 (*C-8*), 22.4 (*C-3*), 20.6 (*C-4*).

IR (neat, cm^{-1}): 2932, 2863, 1707, 1669, 1640, 1448, 1376 (fingerprint region excluded).

HRMS (APCI+): calculated for $C_{22}H_{33}O_2$ (2M+H+): 329.2475 Found: 329.2470.

Synthesis of 1-(bicyclo[2.1.0]pentan-1-yl)-3-(1,3-dioxolan-2-yl)propan-1-one (1i)

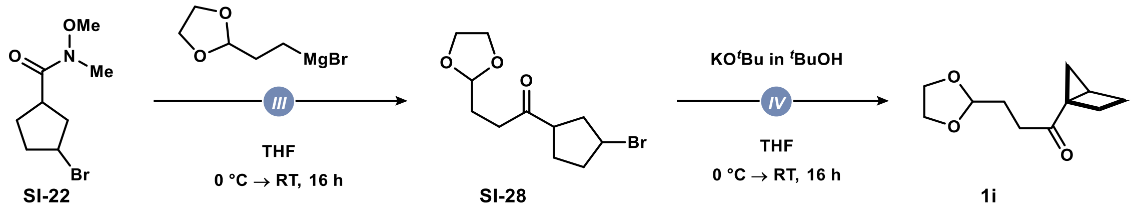

1-(3-Bromocyclopentyl)-3-(1,3-dioxolan-2-yl)propan-1-one (SI-28)

Prepared according to **GP 3** (step III) using (2-(1,3-dioxolan-2-yl)ethyl)magnesium bromide (0.50 M in THF; 15.0 mL, 7.50 mmol, 1.5 eq). The crude residue was purified by column chromatography on silica gel (hexane:EtOAc = 4:1) to afford two diastereoisomers of the target bromo-ketone **SI-28** (970 mg, 3.50 mmol, 70% combined yield; d.r. = 1:0.9). The diastereoisomers were partially separable. Therefore, each isomer was separated for NMR analysis.

MINOR DIASTEREOISOMER

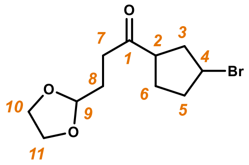

R_f = 0.35. (Hexane:EtOAc = 2:1). Colourless oil.

^{1}H NMR (400 MHz, $CDCl_3$) δ 4.87 (t, *J* = 4.3 Hz, 1H, *H-9*), 4.52 (td, *J* = 5.2, 2.8 Hz, 1H, *H-4*), 3.97 – 3.86 (m, 2H, *H-10a* + *H-11a*), 3.86 – 3.76 (m, 2H, *H-10b* + *H-11b*), 3.32 (dtd, *J* = 9.9, 8.3, 6.2 Hz, 1H, *H-2*), 2.67 – 2.48 (m, 2H, 2*H-7*), 2.32 (ddd, *J* = 14.0, 8.4, 5.4 Hz, 1H, *H-3a*), 2.25 – 2.02 (m, 4H, *H-3b* + *H-6a* + 2*H-5*), 1.95 (td, *J* = 7.3, 4.3 Hz, 2H, 2*H-8*), 1.87 – 1.76 (m, 1H, *H-6b*).

^{13}C{H} NMR (101 MHz, $CDCl_3$) δ 210.9 (*C-1*), 103.3 (*C-9*), 65.0 (*C-10* + *C-11*), 53.4 (*C-4*), 48.8 (*C-2*), 40.2 (*C-3*), 37.4 (*C-5*), 35.9 (*C-7*), 27.5 (*C-8*), 26.6 (*C-6*).

IR (neat, cm^{-1}): 2965, 2883, 1708, 1435, 1410, 1372, 1216, 1139, 1103, 1027 (fingerprint region excluded).

HRMS (ESI+): calculated for $C_{11}H_{17}O_3BrNa$ (M+Na^+): 299.0253 Found: 299.0258.

MAJOR DIASTEREOISOMER

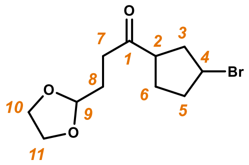

R_f = 0.52. (Hexane:EtOAc = 2:1). Colourless oil.

^{1}H NMR (400 MHz, $CDCl_3$) δ 4.87 (t, *J* = 4.3 Hz, 1H, *H-9*), 4.25 (p, *J* = 6.2 Hz, 1H, *H-4*), 3.98 – 3.86 (m, 2H, *H-10a* + *H-11a*), 3.86 – 3.77 (m, 2H, *H-10b* + *H-11b*), 2.90 (tt, *J* = 9.0, 7.4 Hz, 1H, *H-2*), 2.56 (td, *J* = 7.2, 3.3 Hz, 2H, 2*H-7*), 2.44 (ddd, *J* = 15.3, 9.1, 6.4 Hz, 1H, *H-3a*), 2.29 (ddd, *J* = 14.3, 8.1, 6.9 Hz, 1H, *H-3b*), 2.17 – 1.99 (m, 3H, *H-6a* + 2*H-5*), 1.95 (td, *J* = 7.3, 4.3 Hz, 2H, 2*H-8*), 1.92 – 1.83 (m, 1H, *H-6b*).

^{13}C{H} NMR (101 MHz, $CDCl_3$) δ 209.6 (*C-1*), 103.3 (*C-9*), 65.0 (*C-10* + *C-11*), 50.1 (*C-4*), 49.6 (*C-2*), 39.4 (*C-3*), 37.7 (*C-5*), 35.1 (*C-7*), 27.6 (*C-8*), 26.8 (*C-6*).

IR (neat, cm^{-1}): 2970, 1707, 1431, 1405, 1379, 1221, 1145, 1100, 1029 (fingerprint region excluded).

HRMS (ESI+): calculated for $C_{11}H_{17}O_3BrNa$ (M+Na^+): 299.0253 Found: 299.0255.

1-(Bicyclo[2.1.0]pentan-1-yl)-3-(1,3-dioxolan-2-yl)propan-1-one (1i)

Prepared according to **GP 3** (step IV) using **SI-28** (277 mg, 1.00 mmol, 1.0 eq). The crude residue was purified by column chromatography on silica gel (hexane:Et_2O = 2:1) to afford the target housane ketone **1i** (173 mg, 0.88 mmol, 88% yield).

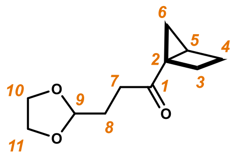

R_f = 0.35. (Hexane:Et_2O = 1:1). Colourless oil.

^{1}H NMR (400 MHz, $CDCl_3$) δ 4.87 (t, *J* = 4.5 Hz, 1H, *H-9*), 3.98 – 3.89 (m, 2H, *H-10a* + *H-11a*), 3.87 – 3.78 (m, 2H, *H-10b* + *H-11b*), 2.51 (tdd, *J* = 11.1, 4.2, 1.7 Hz, 1H, *H-3a*), 2.41 – 2.19 (m, 3H, *H-5* + *2H-7*), 2.06 (ttd, *J* = 11.0, 4.7, 1.5 Hz, 1H, *H-4a*), 2.00 – 1.84 (m, 2H, 2*H-8*), 1.79 – 1.75 (m, 1H, *H-6a*), 1.64 – 1.56 (m, 1H, *H-3b*), 1.38 (dddd, *J* = 11.0, 6.6, 4.2, 1.1 Hz, 1H, *H-4b*), 1.32 (dd, *J* = 4.7, 2.9 Hz, 1H, *H-6b*).

^{13}C{H} NMR (101 MHz, $CDCl_3$) δ 208.0 (*C-1*), 103.6 (*C-9*), 65.0 (*C-10* + *C-11*), 34.3 (*C-2*), 31.0 (*C-7*), 30.2 (*C-5*), 27.8 (*C-8*), 26.3 (*C-6*), 22.3 (*C-3*), 20.6 (*C-4*).

IR (neat, cm^{-1}): 2936, 2867, 1673, 1387, 1137, 1094, 1032 (fingerprint region excluded).

HRMS (ESI+): calculated for $C_{11}H_{16}O_3Na$ (M+Na$^+$): 219.0992 Found: 219.0992.

Synthesis of bicyclo[2.1.0]pentan-1-yl(cycloheptyl)methanone (1k)

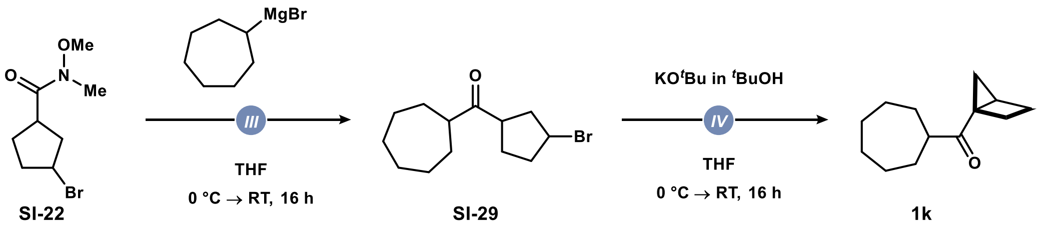

(3-Bromocyclopentyl)(cycloheptyl)methanone (SI-29)

Prepared according to **GP 3** (step III) using cycloheptylmagnesium bromide (2.0 M in THF; 3.75 mL, 7.50 mmol, 1.5 eq). The crude residue was purified by column chromatography on silica gel (hexane:Et_2O = 9:1) to afford an inseparable mixture of diastereoisomers of the target bromo-ketone **SI-29** (751 mg, 2.75 mmol, 55% combined yield; d.r. = 1:0.75).

MIXTURE OF DIASTEREOISOMERS

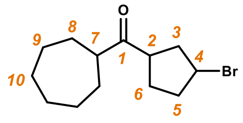

R_f (diastereoisomer 1) = 0.55 and R_f (diastereoisomer 2) = 0.51. (Hexane:Et_2O = 9:1). Colourless oil.

^{1}H NMR (400 MHz, $CDCl_3$) δ 4.57 – 4.48 (m, 1H, *H-4 major*), 4.24 – 4.12 (m, 1H, *H-4 minor*), 3.45 – 3.34 (m, 1H, *H-2 major*), 3.02 – 2.92 (m, 1H, *H-2 minor*), 2.56 (tdt, *J* = 9.5, 7.8, 4.0 Hz, 2H, *H-7 major* + *H-7 minor*), 2.42 – 2.34 (m, 1H, *H-3a minor*), 2.31 – 2.19 (m, 2H, *H-3b minor* + *H-3a major*), 2.19 – 1.93 (m, 7H, *H-3b major* + 2*H-5 major* + 2*H-5 minor* + *H-6a major* + *H-6a minor*), 1.84 – 1.61 (m, 10H, *H-6b major* + *H-6b minor* + 4*H-8 major* + 4*H-8 minor*), 1.58 – 1.33 (m, 16H, 4*H-9 major* + 4*H-9 minor* + 4*H-10 major* + 4*H-10 minor*).

^{13}C{H} NMR (101 MHz, $CDCl_3$) δ 216.0 (*C-1 major*), 214.4 (*C-1 minor*), 54.0 (*C-4 major*), 52.3 (*C-7 major*), 51.4 (*C-7 minor*), 49.3 (*C-4 minor*), 48.6 (*C-2 minor*), 47.1 (*C-2 major*), 41.0 (*C-3 major*), 40.3 (*C-3 minor*), 37.7 (*C-5 major*), 37.6 (*C-5 minor*), 30.2 (*CH_2*), 30.2 (*CH_2*), 30.1 (*CH_2*), 29.9 (*CH_2*), 28.4 (*CH_2*), 27.5 (*C-6 major*), 27.5 (*C-6 minor*), 26.8 (*CH_2*), 26.8 (*CH_2*).

IR (neat, cm^{-1}): 2921, 2853, 1704, 1459, 1445, 1218, 1094, 1042 (fingerprint region excluded).

HRMS (ESI+): calculated for $C_{13}H_{21}OBrNa$ (M+Na$^+$): 295.0668 Found: 295.0666.

Bicyclo[2.1.0]pentan-1-yl(cycloheptyl)methanone (1k)

Prepared according to **GP 3** (step IV) using **SI-29** (273 mg, 1.00 mmol, 1.0 eq). The crude residue was purified by column chromatography on silica gel (hexane:Et_2O = 19:1) to afford the target housane ketone **1k** (106 mg, 0.55 mmol, 55% yield).

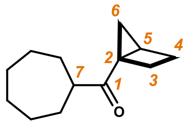

R_f = 0.45. (Hexane:Et_2O = 9:1). Colourless oil.

^{1}H NMR (400 MHz, $CDCl_3$) δ 2.48 – 2.32 (m, 2H, *H-3a* + *H-7*), 2.28 (tdd, *J* = 6.0, 2.6, 1.2 Hz, 1H, *H-5*), 2.01 (ttd, *J* = 11.0, 4.6, 1.6 Hz, 1H, *H-4a*), 1.78 – 1.26 (m, 15H, *H-6a* + *H-3b* + *H-4b* + *6CH_2*), 1.22 (dd, *J* = 4.5, 2.8 Hz, 1H, *H-6b*).

^{13}C{H} NMR (101 MHz, $CDCl_3$) δ 213.0 (*C-1*), 46.3 (*C-7*), 33.0 (*C-2*), 30.3 (*CH_2*), 30.3 (*CH_2*), 29.8 (*C-5*), 28.4 (*CH_2*), 28.3 (*CH_2*), 27.0 (*CH_2*), 26.8 (*CH_2*), 26.4 (*C-6*), 22.9 (*C-3*), 20.6 (*C-4*).

IR (neat, cm^{-1}): 2924, 2858, 1672, 1460, 1446, 1385, 1135, 1047 (fingerprint region excluded).

HRMS (ESI+): calculated for $C_{13}H_{21}O$ (M+H$^+$): 193.1587 Found: 193.1587.

Synthesis of bicyclo[2.1.0]pentan-1-yl(phenyl)methanone (1n)

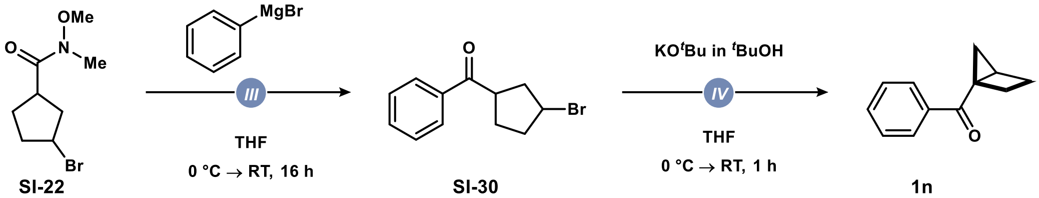

SI-22 SI-30 1n

(3-Bromocyclopentyl)(phenyl)methanone (SI-30)

Prepared according to **GP 3** (step III) using phenylmagnesium bromide (3.0 M in Et_2O; 2.5 mL, 7.50 mmol, 1.5 eq). The crude residue was purified by column chromatography on silica gel (hexane:Et_2O = 14:1) to afford an inseparable mixture of diastereoisomers of the target bromo-ketone **SI-30** (848 mg, 3.35 mmol, 67% combined yield; d.r. = 1:0.36).

MIXTURE OF DIASTEREOISOMERS

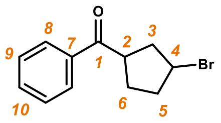

R_f (diastereoisomer 1) = 0.35 and R_f (diastereoisomer 2) = 0.3. (Hexane:EtOAc = 19:1). Colourless oil.

^{1}H NMR (400 MHz, $CDCl_3$) δ 7.94 – 7.83 (m, 4H, 2*H-8 major* + *2H-8 minor*), 7.53 – 7.45 (m, 2H, *H-10 major* + *H-10 minor*), 7.44 – 7.36 (m, 4H, 2*H-9 major* + *2H-9 minor*), 4.57 (tt, *J* = 5.4, 2.8 Hz, 1H, *H-4 major*), 4.30 – 4.21 (m, 1H, *H-4 minor*), 4.09 (dtd, *J* = 10.4, 8.2, 5.7 Hz, 1H, *H-2 major*), 3.73 – 3.62 (m, 1H, *H-2 minor*), 2.57 – 2.47 (m, 1H, *H-3a major* + *H-3a minor*), 2.43 – 1.97 (m, 6H, *H-3b major* + 2*H-5 major* + *H-6a major* + *H-3b minor* + 2*H-5 minor* + *H-6a minor*), 1.97 – 1.84 (m, 1H, *H-6b major* + *H-6b minor*).

^{13}C{H} NMR (101 MHz, $CDCl_3$) δ 201.4 (*C-1 major*), 200.2 (*C-1 minor*), 136.4 (*C-7 minor*), 136.4 (*C-7 major*), 133.3 (*C-10 major*), 133.2 (*C-10 minor*), 128.8 (*2C-8 major*), 128.8 (*2C-8 minor*), 128.7 (*2C-9 major*), 128.6 (*2C-9 minor*), 54.0 (*C-4 major*), 49.2 (*C-4 minor*), 45.3 (*C-2 minor*), 44.2 (*C-2 major*), 40.9 (*C-3 major*), 40.3 (*C-3 minor*), 37.8 (*C-5 minor*), 37.6 (*C-5 major*), 28.1 (*C-6 minor*), 28.0 (*C-6 major*).

IR (neat, cm^{-1}): 1678, 1596, 1579, 1447, 1356, 1217, 1179, 1014 (fingerprint region excluded).

HRMS (ESI+): calculated for $C_{12}H_{13}OBrNa$ (M+Na$^+$): 275.0042 Found: 275.0039.

Bicyclo[2.1.0]pentan-1-yl(phenyl)methanone (1n)

Prepared according to **GP 3** (step IV) using **SI-30** (253 mg, 1.00 mmol, 1.0 eq). The crude residue was purified by column chromatography on silica gel (hexane:EtOAc = 19:1) to afford the target housane ketone **1n** (150 mg, 0.87 mmol, 87% yield).

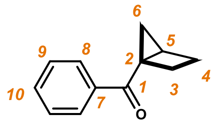

R_f = 0.5. (Hexane: EtOAc = 9:1). Colourless oil.

^{1}H NMR (400 MHz, $CDCl_3$) δ 7.68 – 7.60 (m, 2H, *2H-8*), 7.46 – 7.39 (m, 1H, *H-10*), 7.35 (ddt, *J* = 8.3, 6.6, 1.2 Hz, 2H, *2H-9*), 2.56 (dddd, *J* = 6.4, 4.9, 2.9, 1.3 Hz, 1H, *H-5*), 2.49 (tdd, *J* = 10.9, 4.1, 1.6 Hz, 1H, *H-3a*), 2.06 (ttd, *J* = 10.9, 4.7, 1.6 Hz, 1H, *H-4a*), 1.93 (dddd, *J* = 10.9, 6.1, 4.4, 1.4 Hz, 1H, *H-3b*), 1.72 (ddt, *J* = 5.9, 4.1, 1.6 Hz, 1H, *H-6a*), 1.44 (dd, *J* = 4.1, 2.9 Hz, 1H, *H-6b*), 1.42 – 1.34 (m, 1H, *H-4b*).

^{13}C{H} NMR (101 MHz, $CDCl_3$) δ 202.3 (*C-1*), 137.4 (*C-7*), 132.1 (*C-10*), 128.4 (*C-8*), 128.1 (*C-9*), 32.9 (*C-2*), 30.4 (*C-5*), 29.9 (*C-6*), 26.1 (*C-3*), 21.0 (*C-4*).

IR (neat, cm^{-1}): 2935, 1649, 1598, 1579, 1447, 1357, 1284, 1259, 1218, 1207, 1173, 1034, 1020 (fingerprint region excluded).

HRMS (ESI+): calculated for $C_{12}H_{12}ONa$ (M+Na$^+$): 195.0780 Found: 195.0781.

5.1.3 Synthesis of 1-(bicyclo[2.1.0]pentan-1-yl)-2,2-dimethylpropan-1-one (1m)

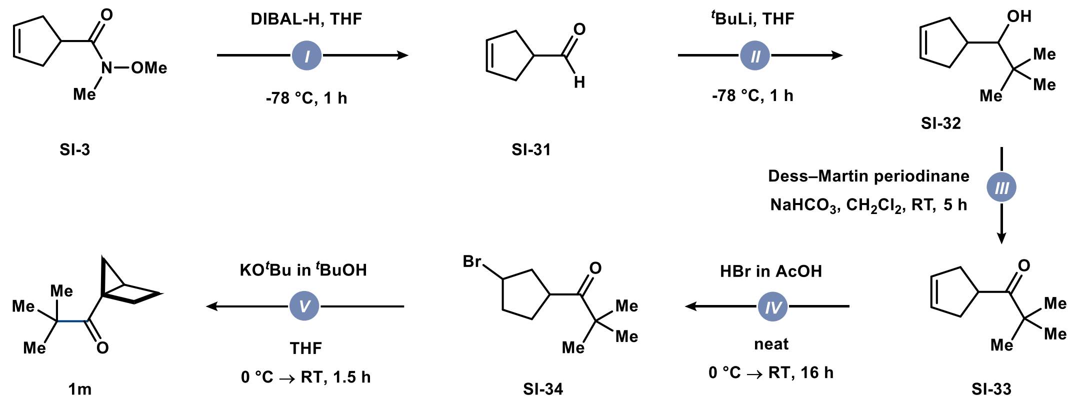

Step I: A flame-dried flask, equipped with a stirrer bar, was charged with **SI-3** (1.55 g, 10.0 mmol, 1.0 eq), and THF (30 mL, 0.2 M). The resultant solution was cooled to -78 °C. DIBAL-H (1.0 M in hexanes, 20.0 mL, 20.0 mmol, 2.0 eq) was added drop-wise to the solution over a period of 15 min, and the resultant solution was stirred at the same temperature for 1 h. The reaction was quenched at −78° C

by the addition of saturated aqueous potassium sodium tartrate solution (25 mL), and the reaction mixture was then stirred at room temperature for 1.5 h before dilution with Et_2O (30 mL). The phases were separated, and the aqueous phase was extracted with Et_2O (3 × 15 mL). The combined organic layers were washed with saturated aqueous NaCl (20 mL), dried over anhydrous $MgSO_4$, filtered, and concentrated *in vacuo* to provide the crude aldehyde **SI-31**, which was used for the next step without further purification.

Step II: A flame-dried flask, equipped with a stirrer bar, was charged with the crude aldehyde **SI-31** from step I (672 mg, 7.00 mmol, assuming 100% pure, 1.0 eq). The flask was then sealed with a rubber septum and purged three times with nitrogen. THF (35 mL, 0.2 M) was added, and the resultant solution was cooled to -78 °C. *tert*-Butyl lithium (1.7 M in pentane, 7.0 mL, 8.40 mmol, 1.2 equiv.) was added dropwise. The resultant solution was vigorously stirred at the same temperature for 1 h. The reaction mixture was quenched with saturated aqueous NH_4Cl (15 mL), phases were separated, and the aqueous phase was extracted with Et_2O (3 × 10 mL). The combined organic layers were washed with saturated aqueous NaCl (20 mL), dried over anhydrous $MgSO_4$, filtered, and concentrated *in vacuo* to provide the crude alcohol **SI-32**, which was used for the next step without further purification.

Step III: An oven-dried flask, equipped with a stirrer bar, was charged with the crude alcohol **SI-32** from step II (1.08 g, 7.00 mmol, assuming 100% pure, 1.0 eq), CH_2Cl_2 (35 mL, 0.2 M), and $NaHCO_3$ (1.76 g, 21.0 mmol, 3.0 eq). Dess-Martin periodinane (4.45 g, 10.5 mmol, 1.5 eq) was then slowly added in one portion, and the mixture was stirred vigorously at room temperature until complete consumption of alcohol **SI-32** was observed (TLC analysis). After completion, the reaction mixture was quenched with saturated aqueous $Na_2S_2O_3$, phases were separated, and the aqueous phase was extracted with CH_2Cl_2 (3 × 15 mL). The combined organic layers were washed with saturated aqueous NaCl (20 mL), dried over anhydrous $MgSO_4$, filtered, and concentrated *in vacuo* to provide the crude ketone **SI-33**, which was used for the next step without further purification.

Step IV: An oven-dried flask, equipped with a stirrer bar, was charged with the ketone **SI-33** from step III (1.08 g, 6.00 mmol, assuming 100% pure, 1.0 eq) and cooled to 0 °C. A solution of 33% HBr in glacial acetic acid (3.1 mL, 18.0 mmol, 3.0 eq) was then added over a period of 5 min. The mixture was gradually warmed to room temperature and stirred vigorously for 16 h, after which it was carefully quenched with dropwise addition of saturated aqueous $NaHCO_3$ (15 mL) and transferred into a

separatory funnel containing H_2O (10 mL) and Et_2O (20 mL). The phases were separated, and the aqueous phase was extracted with EtOAc (3 × 20 mL). The combined organic phases were washed with saturated aqueous NaCl solution (20 mL), dried over anhydrous $MgSO_4$, filtered, and concentrated *in vacuo*. The resultant crude material was purified by column chromatography on silica gel to afford the target product bromoketone **SI-34**.

1-(3-Bromocyclopentyl)-2,2-dimethylpropan-1-one (SI-34)

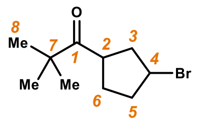

R_f (diastereoisomer 1) = 0.78 and R_f (diastereoisomer 2) = 0.73. (Hexane:Et_2O = 4:1). Colourless oil.

^{1}H NMR (400 MHz, $CDCl_3$) δ 4.61 – 4.50 (m, 1H, *H-4 major*), 4.13 (dq, *J* = 8.5, 6.9 Hz, 1H, *H-4 minor*), 3.65 (tdd, *J* = 9.5, 8.1, 6.3 Hz, 1H, *H-2 major*), 3.22 (tt, *J* = 9.2, 7.9 Hz, 1H, *H-2 minor*), 2.35 (dt, *J* = 14.1, 7.3 Hz, 1H, *H-3a minor*), 2.27 – 2.00 (m, 8H, 2*H-3 major* + 2*H-5 major* + *H-6a major* + *H-3b minor* + *2H-5 minor*), 1.88 (dddd, *J* = 12.5, 8.7, 7.6, 6.2 Hz, 1H, *H-6a minor*), 1.79 – 1.70 (m, 1H, *H-6b minor*), 1.69 – 1.58 (m, 1H, *H-6b major*), 1.09 (s, 9H, *9H-8 major*), 1.07 (s, 9H, *9H-8 minor*).

^{13}C{H} NMR (101 MHz, $CDCl_3$) δ 218.7 (*C-1 major*), 217.0 (*C-1 minor*), 54.3 (*C-4 major*), 48.6 (*C-4 minor*), 44.5 (*C-7 major*), 44.4 (*C-7 minor*), 43.9 (*C-2 minor*), 42.9 (*C-3 major*), 42.4 (*C-2 major*), 42.4 (*C-3 minor*), 37.9 (*C-5 major*), 37.8 (*C-5 minor*), 29.8 (*C-6 minor*), 29.4 (*C-6 major*), 26.2 (*C-8 minor*), 26.2 (*C-8 major*).

IR (neat, cm^{-1}): 2925, 2853, 1702, 1449, 1259, 1240, 1173, 1145, 1073, 1028 (fingerprint region excluded).

HRMS (ESI+): calculated for $C_{10}H_{17}OBrNa$ (M+Na$^+$): 255.0355 Found: 255.0353.

1-(Bicyclo[2.1.0]pentan-1-yl)-2,2-dimethylpropan-1-one (1m)

Prepared according to **GP 3** (step IV) using **SI-34** (175 mg, 0.75 mmol, 1.0 eq). The crude residue was purified by column chromatography on silica gel (hexane:Et_2O = 19:1) to afford the target housane ketone **1m** (63.3 mg, 0.42 mmol, 56% yield).

[***NOTE:*** Product was partially lost during rotary evaporation due to a relatively low boiling point. To minimize this, the temperature of the water bath of the rotary evaporator was maintained at 20 °C.]

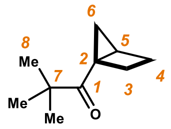

R_f = 0.75. (Hexane:Et_2O = 4:1). Colourless oil.

^{1}H NMR (400 MHz, $CDCl_3$) δ 2.55 (tdd, *J* = 10.9, 3.9, 1.7 Hz, 1H, *H-3a*), 2.32 (dddt, *J* = 6.1, 4.9, 2.5, 1.1 Hz, 1H, *H-5*), 2.02 (ttd, *J* = 11.0, 4.7, 1.7 Hz, 1H, *H-4a*), 1.82 (dddd, *J* = 11.0, 6.2, 4.6, 1.4 Hz, 1H, *H-3b*), 1.45 (ddt, *J* = 5.7, 3.5, 1.7 Hz, 1H, *H-6a*), 1.34 – 1.27 (m, 1H, *H-4b*), 1.15 – 1.09 (m, 10H, *9H-8* + *H-6b*).

^{13}C{H} NMR (101 MHz, $CDCl_3$) δ 214.7 (*C-1*), 44.5 (*C-7*), 32.4 (*C-2*), 29.6 (*C-6*), 28.6 (*C-5*), 27.1 (*C-3*), 26.7 (*C-8*), 21.0 (*C-4*).

IR (neat, cm^{-1}): 2924, 2854, 1672, 1455, 1439, 1389, 1129, 1065 (fingerprint region excluded).

HRMS (ESI+): calculated for $C_{10}H_{16}ONa$ (M+Na$^+$): 175.1093 Found: 175.1097.

5.1.4 Synthesis of 1-(4-pentylbicyclo[2.1.0]pentan-1-yl)pentan-1-one (1s)

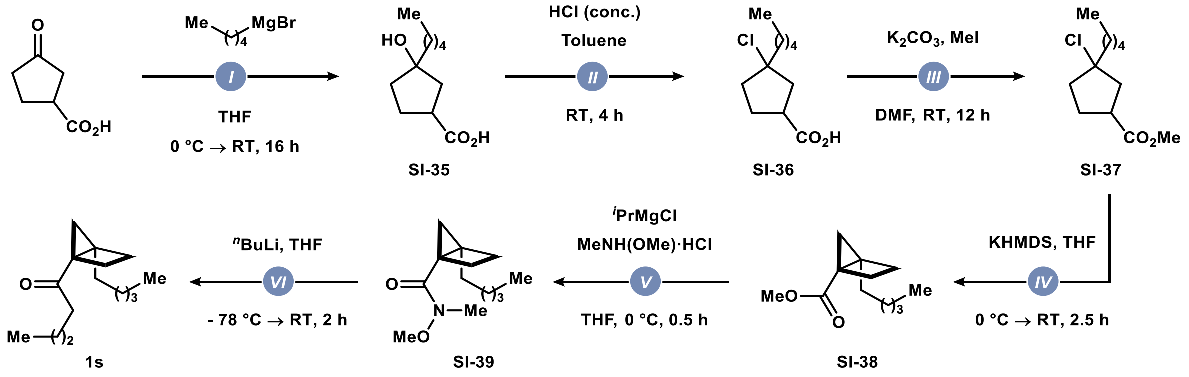

Procedure adapted from Brown and co-workers.[6]

Step I: An oven-dried 250 mL round-bottom flask equipped with a stir bar was cooled under vacuum. After backfilling with N_2 (× 3) and capping with a septum, 1-pentylmagnesium bromide (2 M in Et_2O, 25 mL, 50.0 mmol, 2.5 eq) and THF (50 mL) were introduced. The reaction was cooled to 0 °C in an ice/water bath and a solution of 3-oxocyclopentane-1-carboxylic acid (2.56 g, 20.0 mmol, 1.0 eq) in THF (25 mL) was added dropwise over a period of 10 min. The reaction mixture was then allowed to slowly warm up to room temperature and stirred overnight (16 h) before being quenched with saturated NH_4Cl solution (20 mL). The reaction mixture was then diluted with water (40 mL). The aqueous phase was separated from the organic phase [the non-polar impurities are removed in this step] and the pH of the aqueous phase was adjusted to 1 by dropwise addition of 1 N HCl under vigorous stirring. 25 mL of

EtOAc was then added to the aqueous solution, the phases were separated, and the aqueous phase was extracted with EtOAc (4 × 15 mL). The combined organic phases were washed with saturated aqueous NaCl solution (50 mL), then dried over anhydrous $MgSO_4$, and concentrated *in vacuo* to afford the crude product **SI-35**, which was used directly without further purification.

Step II: A 100 mL round-bottom flask equipped with a magnetic stir bar was charged with the above crude acid **SI-35** (3.53 g, 16.0 mmol, assuming 100% pure, 1.0 eq), concentrated HCl solution (32 mL, 0.5 M), and toluene (32 mL, 0.5 M). The flask was capped with a septum. The reaction was vigorously stirred for 4.5 h at room temperature. The phases were separated, and the aqueous layer was extracted with EtOAc (3 x 25 mL). The combined organic layers were washed with saturated aqueous NaCl solution (50 mL), dried over anhydrous $MgSO_4$, filtered, and concentrated *in vacuo*. The crude product **SI-36** was used directly without further purification.

[***NOTE:*** No caution taken for avoiding O_2 and moisture in this reaction.]

Step III: A 250 mL round-bottom flask equipped with a magnetic stir bar was charged with the above crude acid **SI-36** (3.28 g, 15.0 mmol, assuming 100% pure, 1.0 eq) and DMF (50 mL). K_2CO_3 (4.15 g, 30.0 mmol, 2.0 eq) and MeI (1.40 mL, 22.5 mmol, 1.5 eq) were added sequentially to the solution. The flask was capped with a septum. The reaction was stirred at room temperature for 12 h. The solution was then diluted with H_2O (50 mL) and extracted with Et_2O (3 × 40 mL). The combined organic layers were washed with saturated aqueous NaCl solution (5 × 30 mL), dried over anhydrous $MgSO_4$, filtered, and concentrated *in vacuo* to afford the crude ester **SI-37,** which was used directly without further purification.

[***NOTE:*** No caution taken for avoiding O_2 and moisture in this reaction.]

Step IV: An oven-dried 100 mL round-bottom flask equipped with a stir bar was cooled under vacuum. After backfilling with N_2 (× 3) and capping with a septum, ester **SI-37** (3.49, 15.0 mmol, assuming 100% pure, 1.0 eq) and THF (75 mL, 0.2 M) were introduced. The reaction mixture was cooled to 0 °C in an ice/water bath, and KHMDS (27.0 mL, 1.0 M in THF, 27.0 mmol, 1.8 eq) was added dropwise over a period of 5 min. The reaction was stirred for 0.5 h at the same temperature. The ice/water bath was removed, and the reaction was further stirred for 2 h at room temperature before being quenched with saturated NH_4Cl solution (20 mL). The phases were separated, and the aqueous layer was extracted with Et_2O (3 × 20 mL). The combined organic layers were washed with saturated aqueous NaCl solution

(30 mL), dried over anhydrous $MgSO_4$, filtered, and concentrated *in vacuo* to afford the crude which was purified by column chromatography on neutral alumina (hexane:Et_2O = 19:1 to 9:1 to 4:1 to 1:1 to 2:3) to afford the housane ester **SI-38** (2.12 g, 10.8 mmol, 54% yield over 4 steps).

[***NOTE:*** Attempted purification of the housane **SI-38** using column chromatography over silica, Et_3N-neutralised silica failed due to decomposition of **SI-38**. It also undergoes gradual decomposition upon standing in chloroform-*d*.]

Methyl 4-pentylbicyclo[2.1.0]pentane-1-carboxylate (SI-38)

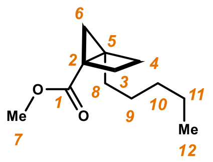

R_f = 0.75. (Hexane:Et_2O = 4:1). Colourless oil.

^{1}H NMR (400 MHz, C_6D_6) δ 3.41 (s, 3H, *3H-7*), 2.51 (tdd, *J* = 11.0, 4.3, 1.7 Hz, 1H, *H-4a*), 1.74 (tdd, *J* = 10.9, 4.3, 2.0 Hz, 1H, *H-3a*), 1.67 – 1.53 (m, 3H, *CH_2* + *H-6a*), 1.48 – 1.40 (m, 1H, *H-4b*), 1.39 – 1.12 (m, 7H, *3CH_2* + *H-3b*), 1.04 (d, *J* = 4.0 Hz, 1H, *H-6b*), 0.86 (t, *J* = 7.0 Hz, 3H, *3H-12*).

^{13}C{H} NMR (101 MHz, C_6D_6) δ 172.1 (*C-1*), 50.9 (*C-7*), 40.6 (*C-2*), 32.0 (*CH_2*), 30.9 (*CH_2*), 29.5 (*C-6*), 29.4 (*C-5*), 27.1 (*CH_2*), 24.8 (*C-3*), 23.0 (*CH_2*), 22.0 (*C-4*), 14.3 (*C-12*).

IR (neat, cm^{-1}): 2953, 2928, 1715, 1436, 1343, 1231, 1193, 1135 (fingerprint region excluded).

HRMS (ESI+): calculated for $C_{12}H_{21}O_2$ (M+H$^+$): 197.1536 Found: 197.1539.

Step V: A flame-dried flask was charged with housane **SI-38** from step IV (225 mg, 1.15 mmol, 1.0 eq) and dry THF (11.5 mL, 0.1 M). The resultant solution was cooled to 0 °C in an ice/water bath. MeNH(OMe)·HCl (248 mg, 2.54 mmol, 2.2 eq) and ^{i}PrMgCl (2 M in THF, 2.35 mL, 4.70 mmol, 4.08 eq) were sequentially added to the solution. After stirring at the same temperature for 35 min, the reaction was quenched by the addition of saturated NH_4Cl solution (7 mL) and diluted with water (8 mL) and Et_2O (5 mL). The phases were separated, and the aqueous layer was extracted with Et_2O (2 × 5 mL) and EtOAc (5 mL). The combined organic layers were washed with saturated aqueous NaCl solution (10 mL), dried over anhydrous Na_2SO_4, filtered, and concentrated *in vacuo* (water bath temperature 39 °C) to afford the crude Weinreb amide **SI-39** as a colourless oil**,** which was used directly without further purification**.**

Step VI: A flame-dried flask was charged with the crude Weinreb amide **SI-39** from step V (245 mg, 1.08 mmol, assuming 100% pure, 1.0 eq) and dry THF (15 mL). The resultant solution was cooled to -78 °C in a dry ice/acetone bath for 15 min, and ^{n}BuLi (1.6 M in hexane, 1.0 mL, 1.62 mmol, 1.5 eq) was added dropwise. After stirring the resultant solution at the same temperature for 0.5 h, the dry ice/acetone bath was removed, and the reaction was stirred at room temperature for 1 h 45 min before being quenched with saturated NH_4Cl solution (10 mL) and diluted with water (5 mL) and Et_2O (5 mL). The phases were separated, and the aqueous layer was extracted with Et_2O (2 × 5 mL) and EtOAc (5 mL). The combined organic layers were washed with saturated aqueous NaCl solution (10 mL), dried over anhydrous Na_2SO_4, filtered, and concentrated *in vacuo* (water bath temperature 39 °C) to afford the crude housane **1s** (236 mg, 1.06 mmol, 92% yield over 2 steps), which was sufficiently pure to be used directly for the next step.

[***NOTE:*** Attempted purification of the housane **1s** using column chromatography over silica, Et_3N-neutralised silica, and neutral alumina failed due to instant decomposition of **1s**. Housane **1s** also undergoes gradual decomposition upon standing in chloroform-*d*.]

1-(4-Pentylbicyclo[2.1.0]pentan-1-yl)pentan-1-one (1s)

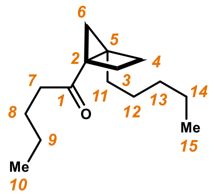

R_f = 0.75. (Hexane:Et_2O = 4:1). Colourless oil.

^{1}H NMR (400 MHz, C_6D_6) δ 2.39 – 2.18 (m, 2H, *H-4a* + *H-7a*), 2.10 (dt, *J* = 16.4, 7.3 Hz, 1H, *H-7b*), 1.78 – 1.69 (m, 2H, *H-3a* + *H-6a*), 1.66 – 1.50 (m, 4H, *2CH₂*), 1.41 (ddd, *J* = 11.2, 6.8, 4.5 Hz, 1H, *H-4b*), 1.37 – 1.12 (m, 9H, *H-3b* + *3CH₂*), 1.05 (d, *J* = 3.8 Hz, 1H, *H-6b*), 0.90 – 0.81 (m, 6H, 3*H-10* + 3*H-15*).

^{13}C{H} NMR (101 MHz, C_6D_6) δ 205.7 (*C-1*), 44.2 (*C-2*), 39.9 (*C-7*), 38.3 (*C-5*), 32.1 (CH_2), 31.1 (*C-6*), 30.7 (CH_2), 27.4 (CH_2), 26.3 (CH_2), 24.8 (*C-3*), 23.0 (CH_2), 22.8 (CH_2), 22.5 (*C-4*), 14.3 (CH_3), 14.2 (CH_3).

IR (neat, cm^{-1}): 2957, 2928, 1671, 1466, 1381, 1042 (fingerprint region excluded).

HRMS (ESI+): calculated for $C_{15}H_{27}O$ (M+H$^+$): 223.2056 Found: 223.2067.

5.2 Preparation of electron-deficient alkenes

tert-Butyl 4-((vinylsulfonyl)oxy)piperidine-1-carboxylate (SI-40)

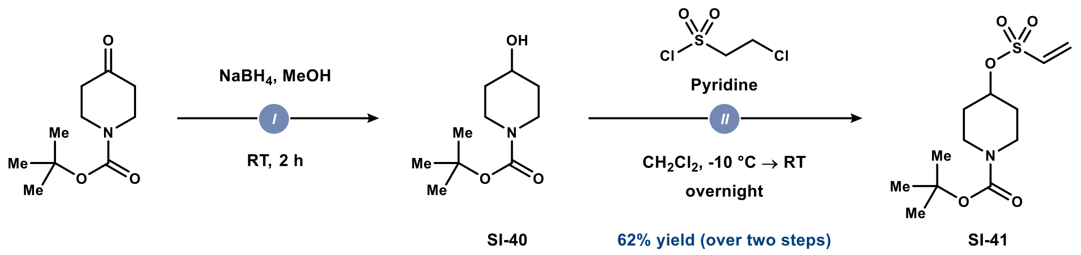

Step I: An oven-dried flask, equipped with a stirrer bar, was charged with *tert*-butyl 4-oxopiperidine-1-carboxylate (996 mg, 5.00 mmol, 1.0 eq) and methanol (16.5 mL, 0.3 M). $NaBH_4$ (246 mg, 6.50 mmol, 1.3 eq) was added at room temperature, and the resultant reaction mixture was stirred at the same temperature until complete consumption of the ketone was observed (TLC analysis). After completion (2 h), the solvent was evaporated *in vacuo*, and the crude was redissolved in water (15 mL) and ethyl acetate (15 mL). The phases were separated, and the aqueous layer was extracted with EtOAc (3 x 7 mL). The combined organic layers were washed with saturated aqueous NaCl solution (15 mL), dried over anhydrous $MgSO_4$, filtered, and concentrated *in vacuo*. The crude alcohol **SI-40** was used directly without further purification**.**

Step II: An oven-dried flask, equipped with a stirrer bar, was charged with crude alcohol **SI-40** from step I (1.00 g, 5.00 mmol, assuming 100% pure, 1.0 eq), 2-chloroethane-1-sulfonyl chloride (0.63 mL, 6.00 mmol, 1.2 eq), and CH_2Cl_2 (20 mL, 0.25 M) under nitrogen. The solution was then cooled to –10 °C and pyridine (0.8 mL, 10.0 mmol, 2.0 eq) in CH_2Cl_2 (5 mL) was added dropwise under vigorous stirring. The resultant solution was gradually warmed to room temperature and stirred overnight. After completion, the reaction was quenched with saturated NH_4Cl solution (15 mL) and diluted with water (10 mL) and CH_2Cl_2 (15 mL). The phases were separated, and the aqueous layer was extracted with CH_2Cl_2 (3 × 7 mL). The combined organic layers were washed with saturated aqueous NaCl solution (10 mL), dried over anhydrous Na_2SO_4, filtered, and concentrated *in vacuo*. The crude alkene was purified by column chromatography on silica gel (hexane:Et_2O = 5:1) to afford the vinyl sulfonate **SI-41** (903 mg, 3.10 mmol, 62% yield over 2 steps).

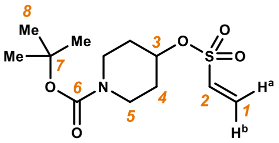

R_f = 0.47, hexane:Et_2O = 5:1. White amorphous solid.

^{1}H NMR (500 MHz, $CDCl_3$) δ 6.51 (dd, *J* = 16.6, 9.9 Hz, 1H, *H-2*), 6.36 (d, *J* = 16.6 Hz, 1H, *H-1a*), 6.04 (d, *J* = 9.9 Hz, 1H, *H-1b*), 4.67 (tt, *J* = 7.5, 3.7 Hz, 1H, *H-3*), 3.60 (ddd, *J* = 13.8, 7.4, 3.9 Hz, 2H, *2H-5a*), 3.24 (ddd, *J* = 13.8, 7.8, 3.9 Hz, 2H, *2H-5b*), 1.86 (ddt, *J* = 14.4, 7.8, 3.8 Hz, 2H, *2H-4a*), 1.78 – 1.69 (m, 2H, *2H-4b*), 1.39 (s, 9H, *9H-8*).

^{13}C{H} NMR (126 MHz, $CDCl_3$) δ 154.7 (*C-6*), 133.7 (*C-2*), 129.7 (*C-1*), 80.1 (*C-7*), 78.7 (*C-3*), 40.5 (*C-5*), 31.6 (*C-4*), 28.5 (*C-8*).

IR (neat, cm^{-1}): 1689, 1422, 1391, 1365, 1276, 1239, 1170, 1012 (fingerprint region excluded)

HRMS (ESI+): calculated for $C_{13}H_{25}NNaO_5S$ (M+Na+): 330.1346 Found: 330.1352.

Isopropyl ethenesulfonate (SI-42)

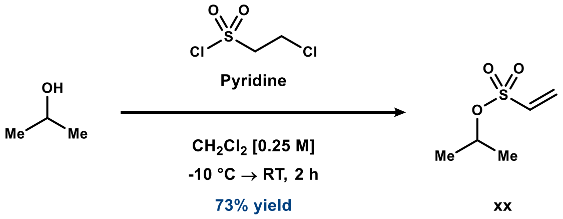

xx

Step II: An oven-dried flask, equipped with a stirrer bar, was charged with isopropanol (0.38 mL, 5.00 mmol, 1.0 eq), 2-chloroethane-1-sulfonyl chloride (0.63 mL, 6.00 mmol, 1.2 eq), and CH_2Cl_2 (25 mL, 0.2 M) under nitrogen. The solution was then cooled to –10 °C and pyridine (0.8 mL, 10.0 mmol, 2.0 eq) in CH_2Cl_2 (5 mL) was added dropwise under vigorous stirring. The resultant solution was gradually warmed to room temperature and stirred for 2 h. After completion, the reaction was quenched with saturated NH_4Cl solution (15 mL) and diluted with water (10 mL) and CH_2Cl_2 (15 mL). The phases were separated, and the aqueous layer was extracted with CH_2Cl_2 (3 × 7 mL). The combined organic layers were washed with saturated aqueous NaCl solution (10 mL), dried over anhydrous Na_2SO_4, filtered, and concentrated *in vacuo* to afford the crude alkene, which was purified by column chromatography on silica gel (hexane:Et_2O = 5:1) to afford the vinyl sulfonate **SI-42** (541 mg, 3.10 mmol, 73% yield over 2 steps).

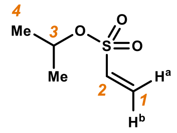

R_f = 0.33, hexane:Et_2O = 4:1. Colourless oil.

^{1}H NMR (400 MHz, $CDCl_3$) δ 6.55 (dd, *J* = 16.7, 9.9 Hz, 1H, *H-2*), 6.38 (d, *J* = 16.7 Hz, 1H, *H-1a*), 6.07 (d, *J* = 9.9 Hz, 1H, *H-1b*), 4.79 (hept, *J* = 6.3 Hz, 1H, *H-3*), 1.39 (d, *J* = 6.3 Hz, 6H, *6H-4*)

^{13}C{H} NMR (101 MHz, $CDCl_3$) δ 133.8 (*C-2*), 129.1 (*C-1*), 77.8 (*C-3*), 23.1 (*C-4*).

Data in accordance with those previously reported.[7]

6. SmI_2-catalyzed cross-coupling of housane ketones and alkynes/alkenes

6.1 Tolerance Experiments.

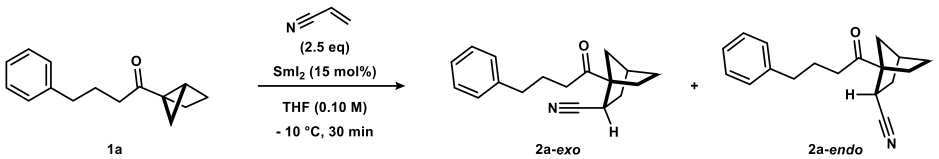

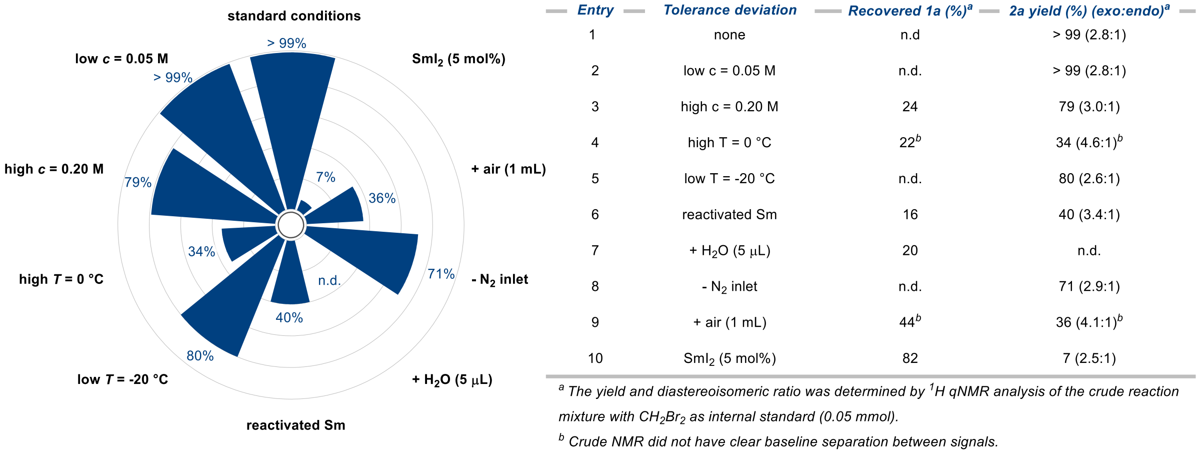

Entry	Tolerance deviation	Recovered 1a (%)[a]	2a yield (%) (exo:endo)[a]
1	none	n.d	> 99 (2.8:1)
2	low c = 0.05 M	n.d.	> 99 (2.8:1)
3	high c = 0.20 M	24	79 (3.0:1)
4	high T = 0 °C	22[b]	34 (4.6:1)[b]
5	low T = -20 °C	n.d.	80 (2.6:1)
6	reactivated Sm	16	40 (3.4:1)
7	+ H_2O (5 µL)	20	n.d.
8	- N_2 inlet	n.d.	71 (2.9:1)
9	+ air (1 mL)	44[b]	36 (4.1:1)[b]
10	SmI_2 (5 mol%)	82	7 (2.5:1)

[a] *The yield and diastereoisomeric ratio was determined by ^1H qNMR analysis of the crude reaction mixture with CH_2Br_2 as internal standard (0.05 mmol).*
[b] *Crude NMR did not have clear baseline separation between signals.*

Experimental variation from the general procedure:

Entry 6 – Sm metal from an older bottle of reagent (>3 years) was first activated by vigorous stirring under high vacuum (60 °C, 16 h) and then used to make SmI_2 according to the previously described method.

Entry 7 – The reaction was set up as with the standard protocol, but degassed H_2O (5 µL) was added prior to cooling and addition of SmI_2.

Entry 8 – The reaction was set up as with the standard protocol, but the N_2 inlet line was removed after cooling and prior to the addition of SmI_2.

Entry 9 – The reaction was set up as with the standard protocol, but the N_2 inlet line was removed, and a syringe filled with air (1 mL) was injected prior to cooling and addition of SmI_2.

To obtain ^1H qNMR yields for reactions, the filtrate was concentrated *in vacuo* and CH_2Br_2 in $CDCl_3$ (1 mL, 0.05 M, 0.05 mmol) was added to the crude residue, and samples were submitted directly for quantitative ^1H NMR.

6.2 Isolation of a Trace Byproduct

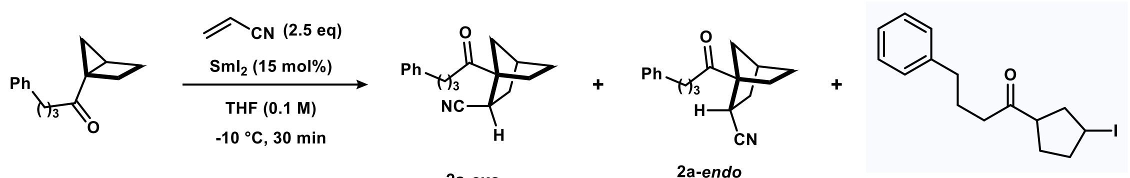

2a-*exo* 2a-*endo* SI-43

In several reactions, we detected trace amounts (< 8%) of byproducts as mixtures of diastereoisomers. From this mixture, we isolated iodide **SI-43**, which likely results from either trapping of the ring-opened carbon-centred radical (cf. Int II, Scheme 5), or a minor competing anionic opening of the housane ketone by iodide. Other side products appear to arise from ketone reduction, post ring-opening.

1-(3-Iodocyclopentyl)-4-phenylbutan-1-one (SI-43)

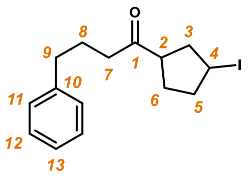

R_f = 0.65, hexane:Et_2O = 4:1, Colourless oil.

^{1}H NMR (500 MHz, $CDCl_3$) δ 7.26 – 7.17 (m, 2H, *2H-11*), 7.16 – 7.04 (m, 3H, *2H-12* + *H-13*), 4.38 – 4.30 (m, 1H, *H-4*), 3.22 – 3.13 (m, 1H, *H-2*), 2.54 (t, *J* = 7.6 Hz, 2H, *2H-9*), 2.46 – 2.32 (m, 2H, *2H-7*), 2.24 – 2.11 (m, 2H, *2H-3*), 2.07 – 1.99 (m, 3H, *2H-5* + *H-6a*), 1.84 (p, *J* = 7.3 Hz, 2H, *2H-8*), 1.74 – 1.67 (m, 1H, *H-6b*).

^{13}C{H} NMR (126 MHz, $CDCl_3$) δ 211.6 (*C-1*), 141.6 (*C-10*), 128.55 (*2C-12*), 128.50 (*2C-11*), 126.1 (*C-13*), 49.4 (*C-2*), 42.1 (*C-3*), 41.4 (*C-7*), 39.5 (*C-5*), 35.2 (*C-9*), 28.0 (*C-4*), 27.4 (*C-6*), 25.2 (*C-8*).

IR (neat, cm^{-1}): 2925,1705, 1453, 1370, 1198, 746, 699, 492 (C-I stretching).

HRMS (ESI+): Calculated for $C_{15}H_{19}INaO$ (M+Na$^+$): 365.0373 Found: 365.0376.

[***N.B.:*** The ^{1}H and ^{13}C NMR spectra of **SI-43** can be found on Page 286.]

6.3 General procedure for SmI_2-catalyzed intermolecular coupling reactions

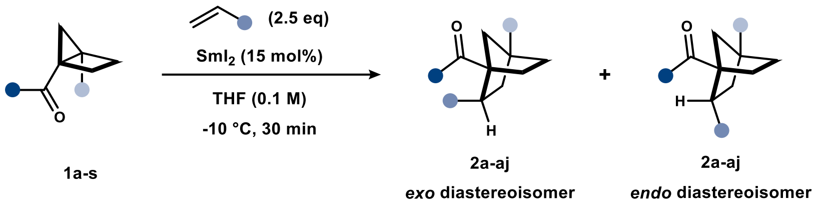

6.3.1 General procedure 4 (GP 4):

An oven-dried microwave reaction vial containing a magnetic stirrer bar was cooled under a stream of N_2 (1 min), immediately sealed, and placed under a positive pressure of N_2. The vial was charged with the corresponding housane ketone (0.20 mmol, 1.0 eq), alkene (0.50 mmol, 2.5 eq), and dry THF (1.7 mL). The vial was placed in an ice/acetone bath (-10° C) for 10 min. Freshly prepared SmI_2 (0.30 mL, 0.10 M, 15 mol%) was introduced by syringe, and the reaction was stirred at -10 °C for 30 min. The cap was removed, and the reaction was quenched by addition of 2 mL of CH_2Cl_2. The resultant mixture was filtered through a silica gel pad (1.5 cm x 4 cm) using ethyl acetate (15 mL) as the eluent. The solvent was removed under reduced pressure. CH_2Br_2 (7 µL, 0.1 mmol) was added as an internal standard for crude ^{1}H NMR analysis to determine the yield and diastereomeric ratio (d.r.).

For isolated yields, the filtrate was concentrated in vacuo, and the crude residue was purified by silica gel column chromatography.

1-(4-Phenylbutanoyl)bicyclo[2.2.1]heptane-2-carbonitrile (2a)

Prepared according to **GP 4** using housane **1a** (43 mg, 0.20 mmol, 1.0 eq) and acrylonitrile (33 µL, 0.50 mmol, 2.5 eq). The crude residue was purified by column chromatography on silica gel (hexane:Et_2O = 4:1 to 2:1 to 3:2) to afford two diastereoisomers of the norbornane **2a** (43.9 mg, 0.16 mmol, 82% combined yield; *exo*:*endo* = 2.8:1). The diastereoisomers were partially separable. Therefore, each isomer was separated for NMR analysis.

ENDO DIASTEREOISOMER (2a-*endo*)

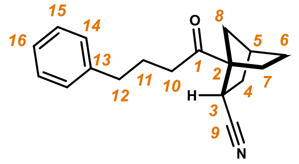

R_f = 0.3. (Hexane:EtOAc = 2:1). Colourless oil.

^{1}H NMR (400 MHz, $CDCl_3$) δ 7.24 – 7.18 (m, 2H, *2H-14*), 7.15 – 7.07 (m, 3H, *2H-15* + *H-16*), 3.02 (ddd, *J* = 12.3, 4.8, 2.6 Hz, 1H, *H-3*), 2.59 – 2.53 (m, 2H, *2H-12*), 2.53 – 2.41 (m, 2H, *2H-10*), 2.41 – 2.37 (m, 1H, *H-5*), 2.11 – 1.98 (m, 2H, *H-4a* + *H-7a*), 1.92 – 1.73 (m, 4H, *2H-11* + *H-6a* + *H-7b*), 1.64 (ddd, *J* = 9.9, 2.6, 1.7 Hz, 1H, *H-8a*), 1.57 – 1.42 (m, 3H, *H-4b* + *H-6b* + *H-8b*).

^{13}C{H} NMR (101 MHz, $CDCl_3$) δ 210.2 (*C-1*), 141.6 (*C-13*), 128.6 (*2C-15*), 128.5 (*2C-14*), 126.1 (*C-16*), 121.7 (*C-9*), 62.6 (*C-2*), 42.9 (*C-8*), 38.1 (*C-10*), 37.8 (*C-5*), 36.1 (*C-4*), 35.0 (*C-12*), 31.7 (*C-3*), 29.7 (*C-6*), 28.9 (*C-7*), 24.9 (*C-11*).

IR (neat, cm^{-1}): 2952, 2875, 2235, 1689, 1602, 1453 (fingerprint region excluded)

HRMS (ESI+): calculated for $C_{18}H_{22}ON$ (M+H+): 268.1696 Found: 268.1694.

EXO DIASTEREOISOMER (2a-*exo*)

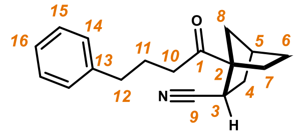

R_f = 0.28. (Hexane:EtOAc = 2:1). Colourless oil.

^{1}H NMR (400 MHz, $CDCl_3$) δ 7.24 – 7.19 (m, 2H, *2H-14*), 7.15 – 7.09 (m, 3H, *2H-15* + *H-16*), 2.66 (ddd, *J* = 9.2, 4.8, 1.7 Hz, 1H, *H-3*), 2.61 – 2.55 (m, 2H, *2H-12*), 2.55 – 2.43 (m, 2H, *2H-10*), 2.43 – 2.39 (m, 1H, *H-5*), 1.95 – 1.85 (m, 3H, *2H-11* + *H-4a*), 1.85 – 1.74 (m, 2H, *H-8a* + *H-4b*), 1.70 – 1.61 (m, 3H, *H-8b* + *H-6a* + *H-7a*), 1.45 – 1.36 (m, 1H, *H-6b*), 1.36 – 1.26 (m, 1H, *H-7b*).

^{13}C{H} NMR (101 MHz, $CDCl_3$) δ 210.2 (*C-1*), 141.7 (*C-13*), 128.6 (*2C-15*), 128.5 (*2C-14*), 126.1 (*C-16*), 121.9 (*C-9*), 63.3 (*C-2*), 39.1 (*C-10*), 39.0 (*C-8*), 36.7 (*C-5*), 36.3 (*C-4*), 35.0 (*C-12*), 33.3 (*C-6* + *C-3*), 29.4 (*C-7*), 24.7 (*C-11*).

IR (neat, cm^{-1}): 2955, 2881, 2233, 1690, 1601, 1461 (fingerprint region excluded)

HRMS (ESI+): calculated for $C_{18}H_{22}ON$ (M+H+): 268.1696 Found: 268.1692.

1-Nonanoylbicyclo[2.2.1]heptane-2-carbonitrile (2b)

Prepared according to **GP 4** using housane **1b** (41.7 mg, 0.20 mmol, 1.0 eq) and acrylonitrile (33 μL, 0.50 mmol, 2.5 eq). The crude residue was purified by column chromatography on silica gel (hexane:EtOAc = 9:1) to afford two diastereoisomers of the norbornane **2b** (42.3 mg, 0.16 mmol, 81% combined yield; *exo*:*endo* = 2.5:1). The diastereoisomers were partially separable. Therefore, each isomer was separated for NMR analysis.

ENDO DIASTEREOISOMER (2b-*endo*)

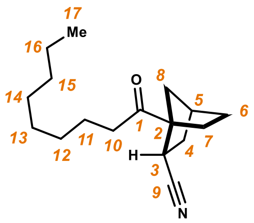

R_f = 0.22. (Hexane:EtOAc = 2:1). Colourless oil.

^{1}H NMR (400 MHz, $CDCl_3$) δ 3.04 (ddd, *J* = 12.3, 4.8, 2.7 Hz, 1H, *H-3*), 2.53 – 2.38 (m, 3H, *2H-10* + *H-5*), 2.13 – 2.01 (m, 2H, *H-4a* + *H-7a*), 1.92 (tdd, *J* = 12.7, 4.3, 2.7 Hz, 1H, *H-7b*), 1.80 (ttd, *J* = 12.3, 4.3, 3.0 Hz, 1H, *H-6a*), 1.68 (ddd, *J* = 10.0, 2.7, 1.8 Hz, 1H, *H-8a*), 1.58 – 1.45 (m, 5H, *2H-11* + *H-4b* + *H-6b* + *H-8b*), 1.26 – 1.14 (m, 10H, *5CH$_2$*), 0.84 – 0.78 (m, 3H, *3H-17*).

^{13}C{H} NMR (101 MHz, $CDCl_3$) δ 210.7 (*C-1*), 121.8 (*C-9*), 62.7 (*C-2*), 42.9 (*C-8*), 39.1 (*C-10*), 37.8 (*C-5*), 36.1 (*C-4*), 32.0 (*CH$_2$*), 31.7 (*C-3*), 29.8 (*CH$_2$*), 29.5 (*C-6*), 29.3 (*CH$_2$*), 29.3 (*CH$_2$*), 28.9 (*C-7*), 23.6 (*C-11*), 22.8 (*CH$_2$*), 14.3 (*C-17*).

IR (neat, cm^{-1}): 2954, 2923, 2874, 2854, 2238, 1699, 1457, 1375, 1195, 1172 (fingerprint region excluded).

HRMS (ESI+): calculated for $C_{17}H_{28}ON$ (M+H$^+$): 262.2165 Found: 262.2159.

EXO DIASTEREOISOMER (2b-*exo*)

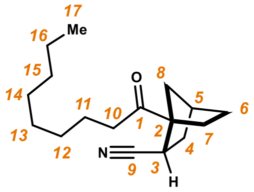

R_f = 0.19. (Hexane:EtOAc = 2:1). Colourless oil.

^{1}H NMR (400 MHz, $CDCl_3$) δ 2.67 (ddd, *J* = 9.1, 4.8, 1.7 Hz, 1H, *H-3*), 2.57 – 2.39 (m, 3H, *2H-10* + *H-5*), 1.91 (dtd, *J* = 12.9, 4.6, 2.5 Hz, 1H, *H-4a*), 1.86 – 1.75 (m, 2H, *H-8a* + *H-4b*), 1.74 – 1.63 (m, 3H, *H-7a* + *H-6a* + *H-8b*), 1.62 – 1.48 (m, 2H, 2*H-11*), 1.47 – 1.40 (m, 1H, *H-6b*), 1.39 – 1.29 (m, 1H, *H-7b*), 1.28 – 1.13 (m, 10H, *5CH$_2$*), 0.81 (t, *J* = 6.9 Hz, 3H, *3H-17*).

^{13}C{H} NMR (101 MHz, $CDCl_3$) δ 210.5 (*C-1*), 121.9 (*C-9*), 63.4 (*C-2*), 40.0 (*C-10*), 39.1 (*C-8*), 36.7 (*C-5*), 36.4 (*C-4*), 33.4 (*C-3*), 33.3 (*C-6*), 32.0 (*CH_2*), 29.6 (*CH_2*), 29.5 (*CH_2*), 29.30 (*CH_2*), 29.28 (*C-7*), 23.3 (*C-11*), 22.8 (*CH_2*), 14.2 (*C-17*).

IR (neat, cm^{-1}): 2955, 2929, 2859, 2239, 1697, 1459, 1369, 1195, 1168 (fingerprint region excluded).

HRMS (ESI+): calculated for $C_{17}H_{28}ON$ (M+H$^+$): 262.2165 Found: 262.2163.

1-(3-Cyclohexylpropanoyl)bicyclo[2.2.1]heptane-2-carbonitrile (2c)

Prepared according to **GP 4** using housane **1c** (41 mg, 0.20 mmol, 1.0 eq) and acrylonitrile (33 µL, 0.50 mmol, 2.5 eq). The crude residue was purified by column chromatography on silica gel (hexane:EtOAc = 9:1 to 4:1) to afford two diastereoisomers of the norbornane **2c** (47.7 mg, 0.18 mmol, 92% combined yield; *exo*:*endo* = 2.3:1). The diastereoisomers were partially separable. Therefore, each isomer was separated for NMR analysis.

ENDO DIASTEREOISOMER (2c-*endo*)

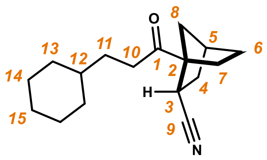

R_f = 0.31. (Hexane:Et_2O = 3:1). Colourless oil.

^{1}H NMR (400 MHz, $CDCl_3$) δ 3.04 (ddd, *J* = 12.2, 4.8, 2.7 Hz, 1H, *H-3*), 2.55 – 2.38 (m, 3H, *2H-10* + *H-5*), 2.13 – 2.01 (m, 2H, *H-7a* + *H-4a*), 1.92 (tdd, *J* = 12.7, 4.3, 2.8 Hz, 1H, *H-7b*), 1.80 (dddd, *J* = 17.2, 9.1, 4.4, 3.0 Hz, 1H, *H-6a*), 1.69 (ddd, *J* = 10.0, 2.7, 1.8 Hz, 1H, *H-8a*), 1.66 – 1.45 (m, 8H, *H-4b* + *H-6b* + *H-8b* + *5H$_{cyclohexyl}$*), 1.41 (q, *J* = 7.2 Hz, 2H, *2H-11*), 1.20 – 1.00 (m, 4H, *H-12* + *3H$_{cyclohexyl}$*), 0.88 – 0.76 (m, 2H, *CH_2 $_{cyclohexyl}$*).

^{13}C{H} NMR (101 MHz, $CDCl_3$) δ 210.8 (*C-1*), 121.7 (*C-9*), 62.7 (*C-2*), 43.0 (*C-8*), 37.8 (*C-5*), 37.3 (*C-12*), 36.7 (*C-10*), 36.1 (*C-4*), 33.3 (*CH_2 cyclohexyl*), 33.3 (*CH_2 cyclohexyl*), 31.8 (*C-3*), 31.0 (*C-11*), 29.8 (*C-6*), 28.9 (*C-7*), 26.7 (*CH_2 cyclohexyl*), 26.4 (2*CH_2 cyclohexyl*).

IR (neat, cm^{-1}): 2920, 2850, 2236, 1699, 1449, 1367, 1197, 1134 (fingerprint region excluded)..

HRMS (ESI+): calculated for $C_{17}H_{26}ON$ (M+H$^+$): 260.2009 Found: 260.2002.

EXO DIASTEREOISOMER (2c-*exo*)

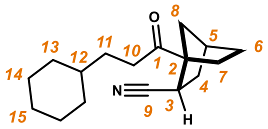

R_f = 0.29. (Hexane:Et_2O = 3:1). Colourless oil.

^{1}H NMR (400 MHz, $CDCl_3$) δ 2.67 (ddd, *J* = 9.1, 4.8, 1.7 Hz, 1H, *H-3*), 2.58 – 2.40 (m, 3H, *2H-10* + *H-5*), 1.94 – 1.54 (m, 12H, *H-4a* + *H-8a* + *H-4b* + *H-6a* + *H-7a* + *H-8b* + *3 $CH_{2\ cyclohexyl}$*), 1.51 – 1.37 (m, 3H, *2H-11* + *H-6b*), 1.36 – 1.28 (m, 1H, *H-7b*), 1.21 – 1.00 (m, 3H, *$CH_{2\ cyclohexyl}$* + *H-12*), 0.90 – 0.75 (*$CH_{2\ cyclohexyl}$*).

^{13}C{H} NMR (126 MHz, $CDCl_3$) δ 210.8 (*C-1*), 121.9 (*C-9*), 63.5 (*C-2*), 39.1 (*C-8*), 37.5 (*C-10*), 37.3 (*C-12*), 36.7 (*C-5*), 36.4 (*C-4*), 33.4 (*C-3*), 33.4 (*$CH_{2\ cyclohexyl}$*), 33.3 (*$CH_{2\ cyclohexyl}$*), 33.2 (*C-6*), 30.6 (*C-11*), 29.5 (*C-7*), 26.7 (*$CH_{2\ cyclohexyl}$*), 26.4 (2*$CH_{2\ cyclohexyl}$*).

IR (neat, cm^{-1}): 2925, 2853, 2241, 1700, 1436, 1354, 1191, 1129 (fingerprint region excluded)..

HRMS (ESI+): calculated for $C_{17}H_{26}ON$ (M+H$^+$): 260.2009 Found: 260.2006.

1-(3-Phenylpropanoyl)bicyclo[2.2.1]heptane-2-carbonitrile (2d)

Prepared according to **GP 4** using housane **1d** (40.0 mg, 0.20 mmol, 1.0 eq) and acrylonitrile (33 μL, 0.50 mmol, 2.5 eq). The crude residue was purified by column chromatography on silica gel (hexane:Et_2O = 4:1) to afford two diastereoisomers of the norbornane **2d** (32 mg, 0.13 mmol, 63% combined yield; *exo*:*endo* = 3:1).

MIXTURE OF DIASTEREOISOMERS (2d-*endo* and 2d-*exo*)

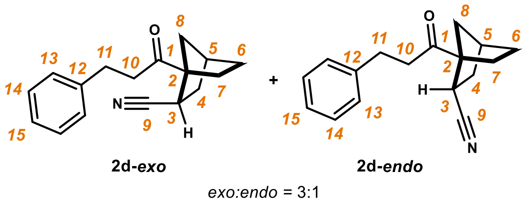

R_f = 0.28. (Hexane:Et_2O = 4:1). Colourless oil.

^{1}H NMR (500 MHz, $CDCl_3$) δ 7.27 – 7.18 (m, 4H, *2H-13$_{exo}$* + *2H-13$_{endo}$*), 7.18 – 7.04 (m, 6H, *2H-14$_{exo}$* + *H-15$_{exo}$* + *2H-14$_{endo}$* + *H-15$_{endo}$*), 3.01 (ddd, *J* = 12.2, 4.9, 2.8 Hz, 1H, *H-3$_{endo}$*), 2.95 – 2.70 (m, 8H, *2H-10$_{exo}$* + *2H-11$_{exo}$* + *2H-10$_{endo}$* + *2H-11$_{endo}$*), 2.66 (ddd, *J* = 9.2, 4.8, 1.7 Hz, 1H, *H-3$_{exo}$*), 2.46 – 2.33 (m, 2H, *H-5$_{exo}$* + *H-5$_{endo}$*), 2.09 – 1.97 (m, 2H, *H-4a$_{endo}$* + *H-7a$_{endo}$*), 1.93 – 1.72 (m, 5H, *H-8a$_{exo}$* + *2H-4$_{exo}$* + *H-6a$_{endo}$* + *H-7b$_{endo}$*), 1.70 – 1.43 (m, 5H, *H-8b$_{exo}$* + *H-6a$_{exo}$* + *H-7a$_{exo}$* + *H-4b$_{endo}$* + *H-6b$_{endo}$*), 1.43 – 1.26 (m, 3H, *H-6b$_{exo}$* + *H-7b$_{exo}$* + *H-8b$_{endo}$*).

^{13}C{H} NMR (126 MHz, $CDCl_3$) δ 209.5 (*C-1*$_{endo}$), 209.4 (*C-1*$_{exo}$), 141.1 (*C-12*$_{exo}$), 141.0 (*C-12*$_{endo}$), 128.7 (*2C-14*$_{endo}$), 128.7 (*2C-14*$_{exo}$), 128.5 (*2C-13*$_{exo}$), 128.5 (*2C-13*$_{endo}$), 121.9 (*C-15*$_{endo}$), 121.6 (*C-15*$_{exo}$), 63.4 (*C-2*$_{exo}$), 62.7 (*C-2*$_{endo}$), 42.8 (*C-8*$_{endo}$), 41.9 (*C-10*$_{exo}$), 40.9 (*C-10*$_{endo}$), 39.0 (*C-8*$_{exo}$), 37.8 (*C-5*$_{endo}$), 36.7 (*C-5*$_{exo}$), 36.3 (*C-4*$_{exo}$), 36.0 (*C-4*$_{endo}$), 33.3 (*C-3*$_{exo}$), 33.1 (*C-6*$_{exo}$), 31.6 (*C-3*$_{endo}$), 29.8 (*C-11*$_{endo}$), 29.7 (*C-6*$_{endo}$), 29.5 (*C-11*$_{exo}$), 29.4 (*C-7*$_{exo}$), 28.8 (*C-7*$_{endo}$).

IR (neat, cm^{-1}): 2955, 2877, 2236, 1699, 1463, 1396 (fingerprint region excluded).

HRMS (ESI+): calculated for $C_{17}H_{20}NO$ (M+H+): 254.1539 Found: 254.1535.

1-(3-(3,4-Dimethoxyphenyl)propanoyl)bicyclo[2.2.1]heptane-2-carbonitrile (2e)

Prepared according to **GP 4** using housane **1e** (52 mg, 0.20 mmol, 1.0 eq) and acrylonitrile (33 µL, 0.50 mmol, 2.5 eq). The crude residue was purified by column chromatography on silica gel (hexane:Et_2O = 1:1) to afford two diastereoisomers of the norbornane **2e** (50.1 mg, 0.16 mmol, 80% combined yield; *exo*:*endo* = 3.2:1).

MIXTURE OF DIASTEREOISOMERS (2e-*endo* and 2e-*exo*)

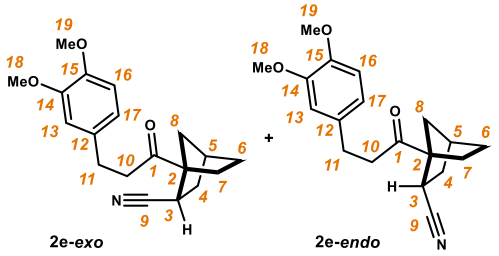

2e-*exo* **2e-*endo***

exo:endo = 3.2:1

R_f = 0.6. (Hexane:Et_2O = 1:4). Colourless oil.

^{1}H NMR (400 MHz, $CDCl_3$) δ 6.74 – 6.69 (m, 2H, *H-16*$_{exo}$ + *H-16*$_{endo}$), 6.69 – 6.61 (m, 4H, *H-17*$_{exo}$ + *H-13*$_{exo}$ + *H-17*$_{endo}$ + *H-13*$_{endo}$), 3.95 – 3.62 (m, 12H, 3*H-18*$_{exo}$ + 3*H-19*$_{exo}$ + 3*H-18*$_{endo}$ + 3*H-19*$_{endo}$), 3.00 (ddd, *J* = 12.3, 4.8, 2.7 Hz, 1H, *H-3*$_{endo}$), 2.90 – 2.69 (m, 8H, 2*H-10*$_{exo}$ + 2*H-11*$_{exo}$ + 2*H-10*$_{endo}$ + 2*H-11*$_{endo}$), 2.69 – 2.63 (m, 1H, *H-3*$_{exo}$), 2.45 – 2.34 (m, 2H, *H-5*$_{exo}$ + *H-5*$_{endo}$), 2.09 – 1.96 (m, 2H, *H-4a*$_{endo}$ + *H-7a*$_{endo}$), 1.89 (dtd, *J* = 13.0, 4.6, 2.3 Hz, 1H, *H-4a*$_{exo}$), 1.84 (dd, *J* = 4.3, 2.8 Hz, 1H, *H-7b*$_{endo}$), 1.83 – 1.71 (m, 3H, *H-8a*$_{exo}$ + *H-4b*$_{exo}$ + *H-6a*$_{endo}$), 1.70 – 1.57 (m, 4H, *H-7a*$_{exo}$ + *H-6a*$_{exo}$ + *H-8b*$_{exo}$ + *H-8a*$_{endo}$), 1.52 (ddd, *J* = 12.7, 4.9, 2.6 Hz, 1H, *H-4b*$_{endo}$), 1.47 (dddd, *J* = 12.1, 9.4, 4.2, 2.3 Hz, 1H, *H-6b*$_{endo}$), 1.42 – 1.25 (m, 3H, *H-6b*$_{exo}$ + *H-7b*$_{exo}$ + *H-8b*$_{endo}$).

^{13}C{H} NMR (101 MHz, $CDCl_3$) δ 209.7 (*C-1*$_{endo}$), 209.5 (*C-1*$_{exo}$), 149.0 (*C-14*$_{exo}$ + *C-14*$_{endo}$), 147.52 (*C-15*$_{endo}$), 147.48 (*C-15*$_{exo}$), 133.7 (*C-12*$_{exo}$), 133.6 (*C-12*$_{endo}$), 121.9 (*C-9*$_{exo}$), 121.6 (*C-9*$_{endo}$), 120.3 (*C-*

17_{exo} + C-17_{endo}), 111.9 (C-13_{exo}), 111.8 (C-13_{endo}), 111.4 (C-16_{endo}), 111.4 (C-16_{exo}), 63.4 (C-2_{exo}), 62.7 (C-2_{endo}), 56.0 (C-18_{exo} + C-18_{endo}), 55.96 (C-19_{exo} + C-19_{endo}), 42.7 (C-8_{endo}), 42.1 (C-10_{exo}), 41.1 (C-10_{endo}), 39.0 (C-8_{exo}), 37.7 (C-5_{endo}), 36.7 (C-5_{exo}), 36.3 (C-4_{exo}), 36.0 (C-4_{endo}), 33.2 (C-3_{exo}), 33.1 (C-6_{exo}), 31.6 (C-3_{endo}), 29.7 (C-6_{endo}), 29.4 (C-11_{endo}), 29.4 (C-7_{exo}), 29.1 (C-11_{exo}), 28.7 (C-7_{endo}).

IR (neat, cm^{-1}): 2953, 2236, 1698, 1514, 1453, 1258, 1236, 1154, 1139, 1027 (fingerprint region excluded).

HRMS (ESI+): calculated for $C_{19}H_{23}O_3NNa$ (M+Na$^+$): 336.1570 Found: 336.1562.

1-(3-(1-Methyl-1*H*-indol-3-yl)propanoyl)bicyclo[2.2.1]heptane-2-carbonitrile (2f)

Prepared according to **GP 4** using housane **1f** (50.6 mg, 0.20 mmol, 1.0 eq) and acrylonitrile (33 µL, 0.50 mmol, 2.5 eq). The crude residue was purified by column chromatography on silica gel (hexane:Et_2O = 1:1) to afford two diastereoisomers of the norbornane **2f** (57 mg, 0.19 mmol, 93% combined yield; *exo*:*endo* = 1.5:1).

MIXTURE OF DIASTEREOISOMERS (2f-*endo* and 2f-*exo*)

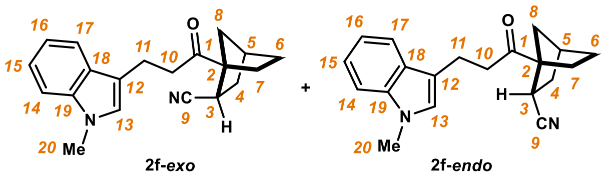

R_f = 0.28. (Hexane:Et_2O = 1:1). Colourless oil.

^{1}H NMR (400 MHz, $CDCl_3$) δ 7.53 – 7.44 (m, 2H, H-17_{exo} + H-17_{endo}), 7.23 – 7.17 (m, 2H, H-14_{exo} + H-14_{endo}), 7.16 – 7.09 (m, 2H, H-15_{exo} + H-15_{endo}), 7.07 – 6.98 (m, 2H, H-16_{exo} + H-16_{endo}), 6.79 (s, 1H, H-13_{exo}), 6.76 (s, 1H, H-13_{endo}), 3.643 (s, 3H, H-20_{endo}), 3.64 (s, 3H, H-20_{exo}), 3.08 – 2.71 (m, 9H, 2H-11_{exo} + 2H-10_{exo} + 2H-11_{endo} + 2H-10_{endo} + H-3_{endo}), 2.62 (ddd, *J* = 9.2, 4.8, 1.7 Hz, 1H, H-3_{exo}), 2.39 – 2.30 (m, 2H, H-5_{exo} + H-5_{endo}), 2.06 – 1.91 (m, 2H, H-$4a_{endo}$ + H-$7a_{endo}$), 1.90 – 1.77 (m, 2H, H-$4a_{exo}$ + H-$7b_{endo}$), 1.77 – 1.53 (m, 7H, 2H-8_{exo} + H-$7a_{exo}$ + H-$4b_{exo}$ + H-$6a_{exo}$ + H-$6a_{endo}$ + H-$8a_{endo}$), 1.52 – 1.39 (m, 2H, H-$4b_{endo}$ + H-$6b_{endo}$), 1.39 – 1.29 (m, 2H, H-$6b_{exo}$ + H-$8b_{endo}$), 1.29 – 1.22 (m, 1H, H-$7b_{exo}$).

^{13}C{H} NMR (101 MHz, $CDCl_3$) δ 210.2 (C-1_{endo}), 210.1 (C-1_{exo}), 137.1 (C-19_{exo} + C-19_{endo}), 127.61 (C-18_{exo}), 127.56 (C-18_{endo}), 126.8 (C-13_{exo}), 126.7 (C-13_{endo}), 121.9 (C-9_{exo} + C-19_{endo}), 121.7 (C-15_{endo}), 121.6 (C-15_{exo}), 118.9 (C-16_{endo}), 118.80 (C-16_{exo}), 118.79 (C-17_{exo}), 118.76 (C-17_{endo}), 113.6 (C-12_{exo}),

113.5 (*C-12*$_{endo}$), 109.4 (*C-14*$_{endo}$), 109.3 (*C-14*$_{exo}$), 63.4 (*C-2*$_{exo}$), 62.7 (*C-2*$_{endo}$), 42.7 (*C-8*$_{endo}$), 40.9 (*C-10*$_{exo}$), 40.0 (*C-10*$_{endo}$), 39.0 (*C-8*$_{exo}$), 37.7 (*C-5*$_{endo}$), 36.7 (*C-5*$_{exo}$), 36.3 (*C-4*$_{exo}$), 36.0 (*C-4*$_{endo}$), 33.2 (*C-3*$_{exo}$), 33.1 (*C-6*$_{exo}$), 32.7 (*C-20*$_{endo}$), 32.6 (*C-20*$_{exo}$), 31.5 (*C-3*$_{endo}$), 29.7 (*C-6*$_{endo}$), 29.4 (*C-7*$_{exo}$), 28.7 (*C-7*$_{endo}$), 19.2 (*C-11*$_{endo}$), 18.9 (*C-11*$_{exo}$).

IR (neat, cm^{-1}): 2951, 2923, 2235, 1697, 1472, 1454, 1375, 1358, 1326, 1248, 1131 (fingerprint region excluded).

HRMS (ESI+): calculated for $C_{20}H_{23}ON_2$ (M+H$^+$): 307.1805 Found: 307.1798.

1-(4-(4,5-Diphenyloxazol-2-yl)butanoyl)bicyclo[2.2.1]heptane-2-carbonitrile (2g)

Prepared according to **GP 4** using housane **1g** (71.5 mg, 0.20 mmol, 1.0 eq) and acrylonitrile (33 μL, 0.50 mmol, 2.5 eq). The crude residue was purified by column chromatography on silica gel (hexane:Et_2O = 2:1) to afford two diastereoisomers of the norbornane **2g** (69.8 mg, 0.17 mmol, 85% combined yield; *exo*:*endo* = 2.4:1).

MIXTURE OF DIASTEREOISOMERS (2g-*endo* and 2g-*exo*)

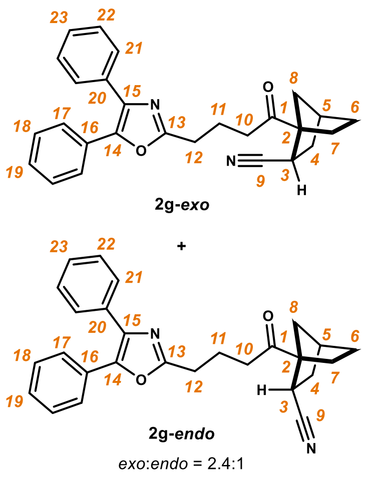

R_f = 0.28. (Hexane:Et_2O = 2:1). Colourless oil.

^{1}H NMR (400 MHz, $CDCl_3$) δ 7.60 – 7.53 (m, 4H, *2H-21*$_{exo}$ + *2H-21*$_{endo}$), 7.53 – 7.47 (m, 4H, *2H-17*$_{exo}$ + *2H-17*$_{endo}$), 7.32 – 7.21 (m, 12H, *2H-22*$_{exo}$ + *H-23*$_{exo}$ + *2H-18*$_{exo}$ + *H-19*$_{exo}$ + *2H-22*$_{endo}$ + *H-23*$_{endo}$ + *2H-18*$_{endo}$ + *H-19*$_{endo}$), 3.03 (ddd, *J* = 12.2, 4.8, 2.7 Hz, 1H, *H-3* $_{endo}$), 2.87 – 2.57 (m, 9H, *2H-12*$_{exo}$ + *H-10*$_{exo}$ + *H-3*$_{exo}$ + *2H-12*$_{endo}$ + *2H-10*$_{endo}$), 2.43 – 2.34 (m, 2H, *H-5*$_{exo}$ + *H-5*$_{endo}$), 2.18 – 1.98 (m, 6H, *2H-11*$_{exo}$ + *2H-11*$_{endo}$ + *H-7a*$_{endo}$ + *H-4a*$_{endo}$), 1.97 – 1.59 (m, 9H, *2H-4*$_{exo}$ + *2H-8*$_{exo}$ + *H-6a*$_{exo}$ + *H-7a*$_{exo}$ + *H-7b*$_{endo}$ + *H-6a*$_{endo}$ + *H-8a*$_{endo}$), 1.55 – 1.37 (m, 4H, *H-6b*$_{exo}$ + *H-4b*$_{endo}$ + *H-6b*$_{endo}$ + *H-8b*$_{endo}$), 1.30 (ddt, *J* = 12.3, 9.4, 3.3 Hz, 1H, *H-8b*$_{exo}$).

^{13}C{H} NMR (101 MHz, $CDCl_3$) δ 209.58 (*C-1*$_{endo}$), 209.55 (*C-1*$_{exo}$), 162.9 (*C-13*$_{exo}$), 162.7 (*C-13*$_{endo}$), 145.4 (*C-14*$_{endo}$), 145.4 (*C-14*$_{exo}$), 135.1 (*C-15*$_{exo}$ + *C-15*$_{endo}$), 132.65 (*C-16*$_{exo}$), 132.59 (*C-16*$_{endo}$), 129.11

(C-20_{exo}), 129.08 (C-20_{endo}), 128.75 ($2C$-18_{endo}), 128.73 ($2C$-18_{exo}), 128.66 ($2C$-22_{endo}), 128.64 ($2C$-22_{exo}), 128.54 (C-19_{endo}), 128.50 (C-19_{exo}), 128.13 (C-23_{endo}), 128.10 (C-23_{exo}), 127.96 ($2C$-17_{exo}), 127.94 ($2C$-17_{endo}), 126.5 ($2C$-21_{exo} + $2C$-21_{endo}), 121.8 (C-9_{exo}), 121.7 (C-9_{endo}), 63.3 (C-2_{exo}), 62.6 (C-2_{endo}), 42.9 (C-8_{endo}), 39.0 (C-8_{exo}), 38.7 (C-10_{exo}), 37.82 (C-10_{endo}), 37.76 (C-5_{endo}), 36.7 (C-5_{exo}), 36.3 (C-4_{exo}), 36.0 (C-8_{endo}), 33.3 (C-3_{exo}), 33.2 (C-6_{exo}), 31.6 (C-3_{endo}), 29.7 (C-6_{endo}), 29.4 (C-7_{exo}), 28.9 (C-7_{endo}), 27.3 (C-12_{exo} + C-12_{endo}), 20.9 (C-11_{endo}), 20.6 (C-11_{exo}).

IR (neat, cm^{-1}): 2954, 2236, 1701, 1446, 1060 (fingerprint region excluded).

HRMS (ESI+): calculated for $C_{27}H_{27}O_2N_2$ (M+H$^+$): 411.2067 Found: 411.2058.

1-(Hex-5-enoyl)bicyclo[2.2.1]heptane-2-carbonitrile (2h)

Prepared according to **GP 4** using housane **1h** (33 mg, 0.20 mmol, 1.0 eq) and acrylonitrile (33 µL, 0.50 mmol, 2.5 eq). The crude residue was purified by column chromatography on silica gel (hexane:EtOAc = 9:1 to 6:1 to 4:1) to afford two diastereoisomers of the norbornane **2h** (39 mg, 0.18 mmol, 90% combined yield; *exo*:*endo* = 3.4:1).

MIXTURE OF DIASTEREOISOMERS (2h-*endo* and 2h-*exo*)

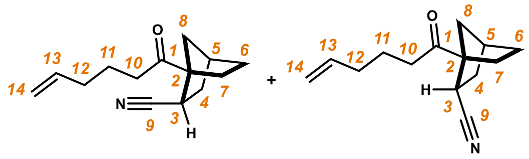

R_f = 0.35. (Hexane:EtOAc = 4:1). Colourless oil.

^{1}H NMR (400 MHz, $CDCl_3$) δ 5.78 – 5.60 (m, 2H, H-13_{exo} + H-13_{endo}), 5.01 – 4.85 (m, 4H, $2H$-14_{exo} + $2H$-14_{endo}), 3.04 (ddd, J = 12.2, 4.9, 2.7 Hz, 1H, H-3_{endo}), 2.67 (ddd, J = 9.2, 4.8, 1.8 Hz, 1H, H-3_{exo}), 2.60 – 2.39 (m, 6H, H-5_{exo} + $2H$-10_{exo} + H-5_{endo} + $2H$-10_{endo}), 2.13 – 1.94 (m, 5H, $2H$-12_{exo} + H-$7a_{endo}$ + $2H$-12_{endo}), 1.94 – 1.87 (m, 2H, H-$4a_{exo}$ + H-$7b_{endo}$), 1.87 – 1.75 (m, 4H, H-$8a_{exo}$ + H-$4b_{exo}$ + H-$6a_{endo}$ + H-$4a_{endo}$), 1.75 – 1.58 (m, 8H, H-$8b_{exo}$ + H-$6a_{exo}$ + H-$7a_{exo}$ + $2H$-11_{exo} + H-$8a_{endo}$ + $2H$-11_{endo}), 1.58 – 1.40 (m, 4H, H-$6b_{exo}$ + H-$8b_{endo}$ + H-$4b_{endo}$ + H-$6b_{endo}$), 1.40 – 1.29 (m, 1H, H-$7b_{exo}$).

^{13}C{H} NMR (101 MHz, $CDCl_3$) δ 210.2 (C-1_{exo} + C-1_{endo}), 138.1 (C-13_{exo}), 138.0 (C-13_{endo}), 121.9 (C-9_{exo}), 121.7 (C-9_{endo}), 115.5 (C-14_{endo}), 115.4 (C-14_{exo}), 63.3 (C-2_{exo}), 62.6 (C-2_{endo}), 42.9 (C-8_{endo}), 39.0 (C-8_{exo}), 38.9 (C-10_{exo}), 38.0 (C-10_{endo}), 37.8 (C-5_{endo}), 36.7 (C-5_{exo}), 36.3 (C-4_{exo}), 36.0 (C-4_{endo}), 33.3

(C-3_{exo} + C-12_{exo}), 33.0 (C-12_{endo}), 33.0 (C-6_{exo}), 31.7 (C-3_{endo}), 29.7 (C-6_{endo}), 29.4 (C-7_{exo}), 28.8 (C-7_{endo}), 22.5 (C-11_{endo}), 22.1 (C-11_{exo}).

IR (neat, cm^{-1}): 2952, 2877, 2236, 1699, 1454, 1368 (fingerprint region excluded).

HRMS (ESI+): calculated for $C_{14}H_{20}NO$ (M+H+): 218.1539 Found: 218.1540.

1-(3-(1,3-Dioxolan-2-yl)propanoyl)bicyclo[2.2.1]heptane-2-carbonitrile (2i)

Prepared according to **GP 4** using housane **1i** (39.3 mg, 0.20 mmol, 1.0 eq) and acrylonitrile (33 µL, 0.50 mmol, 2.5 eq). The crude residue was purified by column chromatography on silica gel (hexane:Et_2O = 2:1) to afford two diastereoisomers of the norbornane **2i** (42.4 mg, 0.17 mmol, 85% combined yield; *exo*:*endo* = 2.4:1).

MIXTURE OF DIASTEREOISOMERS (2i-*endo* and 2i-*exo*)

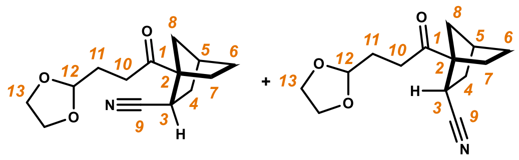

2i-*exo* **2i-*endo***

exo:*endo* = 2.4:1

R_f = 0.3. (Hexane:Et_2O = 2:1). Colourless oil.

^{1}H NMR (500 MHz, $CDCl_3$) δ 4.85 (q, J = 4.5 Hz, 2H, H-12_{exo} + H-12_{endo}), 3.93 – 3.85 (m, 4H, $2H$-$13a_{exo}$ + $2H$-$13a_{endo}$), 3.82 – 3.74 (m, 4H, $2H$-$13b_{exo}$ + $2H$-$13b_{endo}$), 3.06 (ddd, J = 12.3, 4.8, 2.8 Hz, 1H, H-3_{endo}), 2.73 – 2.65 (m, 2H, H-$10a_{exo}$ + H-3_{exo}), 2.64 – 2.53 (m, 3H, H-$10b_{exo}$ + $2H$-10_{endo}), 2.43 (ddq, J = 8.3, 4.3, 2.0 Hz, 2H, H-5_{exo} + H-5_{endo}), 2.13 – 2.01 (m, 2H, H-$4a_{endo}$ + H-$7a_{endo}$), 2.01 – 1.67 (m, 13H, $2H$-4_{exo} + $2H$-8_{exo} + $2H$-11_{exo} + H-$6a_{exo}$ + H-$7a_{exo}$ + $2H$-11_{endo} + H-$6a_{endo}$ + H-$7b_{endo}$ + H-$8a_{endo}$), 1.58 – 1.40 (m, 4H, H-$6b_{exo}$ + H-$6b_{endo}$ + H-$4b_{endo}$ + H-$8b_{endo}$), 1.37 – 1.29 (m, 1H, H-$7b_{exo}$).

^{13}C{H} NMR (126 MHz, $CDCl_3$) δ 209.4 (C-1_{exo}), 209.3 (C-1_{endo}), 121.8 (C-9_{exo}), 121.6 (C-9_{endo}), 103.2 (C-12_{exo}), 103.2 (C-12_{endo}), 65.0 (C-13_{exo}), 65.0 (C-13_{endo}), 63.3 (C-2_{exo}), 62.5 (C-2_{endo}), 43.0 (C-8_{endo}), 39.1 (C-8_{exo}), 37.8 (C-5_{endo}), 36.7 (C-5_{exo}), 36.3 (C-4_{exo}), 36.1 (C-4_{endo}), 33.7 (C-10_{exo}), 33.3 (C-6_{exo}), 33.3 (C-3_{exo}), 32.8 (C-10_{endo}), 31.7 (C-3_{endo}), 29.7 (C-6_{endo}), 29.5 (C-7_{exo}), 28.9 (C-7_{endo}), 27.3 (C-10_{endo}), 27.0 (C-10_{exo}).

IR (neat, cm^{-1}): 2956, 2878, 2236, 1701, 1134, 1070, 1032 (fingerprint region excluded).

HRMS (ESI+): calculated for $C_{14}H_{19}O_3NNa$ (M+Na$^+$): 272.1257 Found: 272.1251.

1-(Cyclohexanecarbonyl)bicyclo[2.2.1]heptane-2-carbonitrile (2j)

Prepared according to **GP 4** using housane **1j** (35.7 mg, 0.20 mmol, 1.0 eq) and acrylonitrile (33 µL, 0.50 mmol, 2.5 eq). The crude residue was purified by column chromatography on silica gel (hexane:Et_2O = 2:1) to afford two diastereoisomers of the norbornane **2j** (38.4 mg, 0.16 mmol, 83% combined yield; *exo*:*endo* = 3.3:1). The diastereoisomers were partially separable. Therefore, each isomer was separated for NMR analysis.

ENDO DIASTEREOISOMER (2j-*endo*)

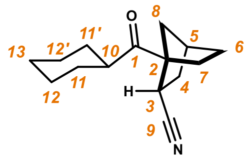

R_f = 0.2. (Hexane:EtOAc = 9:1). Colourless oil.

¹H NMR (500 MHz, $CDCl_3$) δ 3.05 (ddd, *J* = 12.3, 4.8, 2.7 Hz, 1H, *H-3*), 2.64 (tt, *J* = 11.6, 3.3 Hz, 1H, *H-10*), 2.42 (td, *J* = 4.2, 2.0 Hz, 1H, *H-5*), 2.11 – 2.00 (m, 2H, *H-4a* + *H-7a*), 1.96 (tdd, *J* = 13.0, 4.6, 2.7 Hz, 1H, *H-7b*), 1.81 (ttd, *J* = 12.4, 4.6, 3.1 Hz, 1H, *H-6a*), 1.72 (dddd, *J* = 10.0, 7.2, 5.1, 2.6 Hz, 3H, *H-8a* + $2H_{cyclohexyl}$), 1.66 – 1.50 (m, 5H, *H-4b* + *H-6b* + $3H_{cyclohexyl}$), 1.46 (dq, *J* = 9.9, 2.0 Hz, 1H, *H-8b*), 1.35 (dddd, *J* = 17.9, 16.1, 9.1, 4.5 Hz, 2H, $2H_{cyclohexyl}$), 1.25 – 1.15 (m, 3H, $3H_{cyclohexyl}$).

¹³C{H} NMR (101 MHz, $CDCl_3$) δ 213.8 (*C-1*), 121.8 (*C-9*), 62.8 (*C-2*), 46.8 (*C-10*), 42.9 (*C-8*), 37.8 (*C-5*), 36.1 (*C-4*), 31.5 (*C-3*), 29.8 (*C-6*), 29.4 ($CH_{2\ cyclohexyl}$), 29.3 ($CH_{2\ cyclohexyl}$), 28.5 (*C-7*), 25.8 ($CH_{2\ cyclohexyl}$), 25.7 ($CH_{2\ cyclohexyl}$), 25.7 ($CH_{2\ cyclohexyl}$).

IR (neat, cm⁻¹): 2933, 2880, 2235, 1694, 1455, 1365, 1189, 1140, 1119 (fingerprint region excluded).

HRMS (ESI+): calculated for $C_{15}H_{21}ONNa$ ($M+Na^+$): 254.1515 Found: 254.1512.

EXO DIASTEREOISOMER (2j-*exo*)

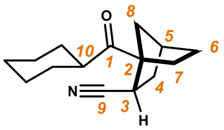

R_f = 0.19. (Hexane:EtOAc = 9:1). White crystalline solid.

¹H NMR (400 MHz, $CDCl_3$) δ 2.68 (ddd, *J* = 9.1, 4.7, 1.9 Hz, 1H, *H-3*), 2.61 (tt, *J* = 11.5, 3.2 Hz, 1H, *H-10*), 2.44 (td, *J* = 4.1, 1.9 Hz, 1H, *H-5*), 1.94 – 1.85 (m, 2H, *H-4a* + *H-8a*), 1.83 – 1.57 (m, 9H, *H-6a* + *H-4b* + *H-8b* + *H-7a* + $5H_{cyclohexyl}$), 1.53 – 1.13 (m, 7H, *H-6b* + *H-7b* + $5H_{cyclohexyl}$).

^{13}C{H} NMR (126 MHz, $CDCl_3$) δ 213.7 (*C-1*), 122.2 (*C-9*), 63.7 (*C-2*), 48.0 (*C-10*), 39.1 (*C-8*), 36.8 (*C-4*), 36.3 (*C-5*), 33.4 (*C-3*), 33.2 (*C-6*), 29.4 ($CH_{2\ cyclohexyl}$), 29.1 ($CH_{2\ cyclohexyl}$), 28.8 (*C-7*), 25.9 ($CH_{2\ cyclohexyl}$), 25.8 ($CH_{2\ cyclohexyl}$), 25.6 ($CH_{2\ cyclohexyl}$).

IR (neat, cm^{-1}): 2929, 2876, 2855, 2235, 1693, 1450, 1371, 1197, 1142, 1123 (fingerprint region excluded).

HRMS (ESI+): calculated for $C_{15}H_{21}ONNa$ (M+Na$^+$): 254.1515 Found: 254.1508.

Melting Point (from hexane:EtOAc): 60 – 63 °C.

1-(Cycloheptanecarbonyl)bicyclo[2.2.1]heptane-2-carbonitrile (2k)

Prepared according to **GP 4** using housane **1k** (38.5 mg, 0.20 mmol, 1.0 eq) and acrylonitrile (33 µL, 0.50 mmol, 2.5 eq). The crude residue was purified by column chromatography on silica gel (hexane:Et_2O = 2:1) to afford two diastereoisomers of the norbornane **2k** (40.2 mg, 0.16 mmol, 82% combined yield; *exo*:*endo* = 2.8:1).

MIXTURE OF DIASTEREOISOMERS (2k-*endo* and 2k-*exo*)

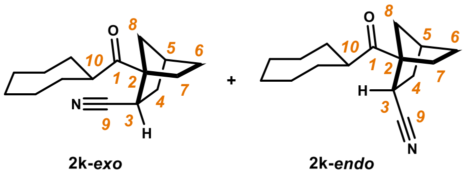

2k-*exo* **2k-*endo***

exo:endo = 2.8:1

R_f = 0.45. (Hexane:Et_2O = 2:1). Colourless oil.

^{1}H NMR (400 MHz, $CDCl_3$) δ 3.07 (ddd, *J* = 12.3, 4.8, 2.7 Hz, 1H, $H\text{-}3_{endo}$), 2.82 – 2.70 (m, 2H, $H\text{-}10_{exo}$ + $H\text{-}10_{endo}$), 2.67 (ddd, *J* = 9.2, 4.8, 1.8 Hz, 1H, $H\text{-}3_{exo}$), 2.47 – 2.40 (m, 2H, $H\text{-}5_{exo}$ + $H\text{-}5_{endo}$), 1.99 – 1.28 (m, 28H, $2H\text{-}8_{exo}$ + $2H\text{-}8_{endo}$ + $2H\text{-}4_{exo}$ + $2H\text{-}4_{endo}$ + $2H\text{-}6_{exo}$ + $2H\text{-}6_{endo}$ + $2H\text{-}7_{exo}$ + $2H\text{-}7_{endo}$ + $6H_{cycloheptyl\text{-}exo}$ + $6H_{cycloheptyl\text{-}endo}$).

^{13}C{H} NMR (101 MHz, $CDCl_3$) δ 214.3 ($C\text{-}1_{endo}$), 214.1 ($C\text{-}1_{exo}$), 122.2 ($C\text{-}9_{exo}$), 121.8 ($C\text{-}9_{endo}$), 63.7 ($C\text{-}2_{exo}$), 62.7 ($C\text{-}2_{endo}$), 48.9 ($C\text{-}10_{exo}$), 47.6 ($C\text{-}10_{endo}$), 43.0 ($C\text{-}8_{endo}$), 39.2 ($C\text{-}8_{exo}$), 37.8 ($C\text{-}5_{endo}$), 36.9 ($C\text{-}5_{exo}$), 36.3 ($C\text{-}4_{exo}$), 36.1 ($C\text{-}4_{endo}$), 33.4 ($C\text{-}6_{exo}$), 33.3 ($C\text{-}3_{exo}$), 31.5 ($C\text{-}3_{endo}$), 31.1, 30.8, 30.6, 30.4, 29.8, 29.4, 28.5, 28.4, 28.3, 28.2, 26.9, 26.8.

IR (neat, cm^{-1}): 2925, 2857, 2235, 1697, 1457, 1376, 1308, 1196, 1129 (fingerprint region excluded).

HRMS (ESI+): calculated for $C_{16}H_{24}ON$ (M+H$^+$): 246.1852 Found: 246.1853.

1-(Tetrahydro-2*H*-pyran-4-carbonyl)bicyclo[2.2.1]heptane-2-carbonitrile (2l)

Prepared according to **GP 4** using housane **1l** (36 mg, 0.20 mmol, 1.0 eq) and acrylonitrile (33 µL, 0.50 mmol, 2.5 eq). The crude residue was purified by column chromatography on silica gel (hexane:EtOAc = 6:1 to 4:1 to 2:1) to afford two diastereoisomers of the norbornane **2l** (39.7 mg, 0.17 mmol, 85% combined yield; *exo*:*endo* = 2.8:1).

MIXTURE OF DIASTEREOISOMERS (2l-*endo* and 2l-*exo*)

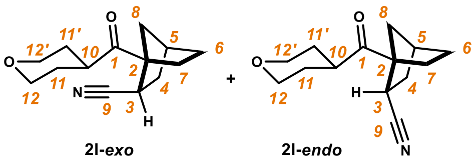

exo:*endo* = 2.8:1

R_f = 0.45. (Hexane:Et_2O = 1:2). Colourless oil.

^{1}H NMR (400 MHz, $CDCl_3$) δ 4.01 – 3.88 (m, 4H, *H-12a*$_{exo}$ + *H-12'a*$_{exo}$ + *H-12a*$_{endo}$ + *H-12'a*$_{endo}$), 3.43 – 3.30 (m, 4H, *H-12b*$_{exo}$ + *H-12'b*$_{exo}$ + *H-12b*$_{endo}$ + *H-12'b*$_{endo}$), 3.06 (ddd, *J* = 12.3, 4.9, 2.8 Hz, 1H, *H-3*$_{endo}$), 2.98 – 2.80 (m, 2H, *H-10*$_{exo}$ + *H-10*$_{endo}$), 2.71 (ddd, *J* = 9.2, 4.8, 1.8 Hz, 1H, *H-3*$_{exo}$), 2.56 – 2.40 (m, 2H, *H-5*$_{exo}$ + *H-5*$_{endo}$), 2.14 – 2.02 (m, 2H, *H-4a*$_{endo}$ + *H-7a*$_{endo}$), 2.01 – 1.31 (m, 21H, 2*H-4*$_{exo}$ + 2*H-6*$_{exo}$ + 2*H-8*$_{exo}$ + 2*H-7*$_{exo}$ + 2*H-11*$_{exo}$ + 2*H-11'*$_{exo}$ + *H-4b*$_{endo}$ + 2*H-6*$_{endo}$ + 2*H-8*$_{endo}$ + 2*H-11*$_{endo}$ + 2*H-11'*$_{endo}$).

^{13}C{H} NMR (101 MHz, $CDCl_3$) δ 211.9 (*C-1*$_{endo}$), 211.8 (*C-1*$_{exo}$), 122.0 (*C-9*$_{exo}$), 121.6 (*C-9*$_{endo}$), 67.2 (*C-12*$_{exo}$), 67.1 (*C-12*$_{endo}$ + *C-12'*$_{endo}$), 67.0 (*C-12'*$_{exo}$), 63.7 (*C-2*$_{exo}$), 62.7 (*C-2*$_{endo}$), 44.9 (*C-10*$_{exo}$), 43.6 (*C-10*$_{endo}$), 42.8 (*C-8*$_{endo}$), 39.0 (*C-8*$_{exo}$), 37.8 (*C-5*$_{endo}$), 36.7 (*C-5*$_{exo}$), 36.1 (*C-4*$_{exo}$), 35.9 (*C-4*$_{endo}$), 33.4 (*C-3*$_{exo}$), 33.2 (*C-6*$_{exo}$), 31.6 (*C-3*$_{endo}$), 29.7 (*C-6*$_{endo}$), 29.3 (*C-7*$_{exo}$), 29.0 (*C-11*$_{endo}$), 28.7 (*C-11'*$_{endo}$), 28.7 (*C-11*$_{exo}$), 28.5 (*C-7*$_{endo}$), 28.4 (*C-11'*$_{exo}$).

IR (neat, cm^{-1}): 2963, 2849, 2236, 1695, 1445, 1241, 1122, 1090 (fingerprint region excluded).

HRMS (ESI+): calculated for $C_{14}H_{19}O_2NNa$ ($M+Na^+$): 256.1308 Found: 256.1302.

1-Pivaloylbicyclo[2.2.1]heptane-2-carbonitrile (2m)

Prepared according to **GP 4** using housane **1m** (30.4 mg, 0.20 mmol, 1.0 eq) and acrylonitrile (33 µL, 0.50 mmol, 2.5 eq). The crude residue was purified by column chromatography on silica gel (hexane:Et_2O = 2:1) to afford two diastereoisomers of the norbornane **2m** (7.4 mg, 0.04 mmol, 18% combined yield; *exo*:*endo* = 2.8:1).

MIXTURE OF DIASTEREOISOMERS (2m-*endo* and 2m-*exo*)

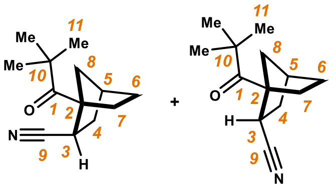

R_f = 0.3. (Hexane:Et_2O = 2:1). Colourless oil.

^{1}H NMR (400 MHz, $CDCl_3$) δ 3.11 (ddd, *J* = 12.3, 4.9, 2.7 Hz, 1H, *H-3*$_{endo}$), 2.85 (ddd, *J* = 9.3, 4.9, 1.7 Hz, 1H, *H-3*$_{exo}$), 2.44 – 2.38 (m, 2H, *H-5*$_{exo}$ + *H-5*$_{endo}$), 2.23 – 2.05 (m, 2H, *2H-7*$_{endo}$ + *H-4a*$_{endo}$), 2.04 – 1.56 (m, 9H, *2H-8*$_{exo}$ + *2H-6*$_{exo}$ + *H-4a*$_{exo}$ + *H-7a*$_{exo}$ + *2H-8*$_{endo}$ + *H-6a*$_{endo}$), 1.52 – 1.44 (m, 3H, *H-4b*$_{exo}$ + *H-4b*$_{endo}$ + *H-6b*$_{endo}$), 1.34 – 1.26 (m, 1H, *H-7b*$_{exo}$), 1.19 (s, 9H, *9H-11*$_{exo}$), 1.17 (s, 9H, *9H-11*$_{endo}$).

^{13}C{H} NMR (101 MHz, $CDCl_3$) δ 214.6 (*C-1*$_{exo}$), 214.3 (*C-1*$_{endo}$), 122.6 (*C-9*$_{exo}$), 122.2 (*C-9*$_{endo}$), 63.6 (*C-2*$_{exo}$), 62.4 (*C-2*$_{endo}$), 45.7 (*C-10*$_{exo}$), 45.5 (*C-10*$_{endo}$), 43.7 (*C-8*$_{endo}$), 40.0 (*C-8*$_{exo}$), 37.7 (*C-5*$_{endo}$), 36.8 (*C-5*$_{exo}$), 35.7 (*C-3*$_{exo}$), 35.4 (*C-4*$_{endo}$), 35.0 (*C-6*$_{exo}$), 34.7 (*C-3*$_{endo}$), 34.5 (*C-4*$_{exo}$), 29.7 (*C-6*$_{endo}$), 29.4 (*C-7*$_{endo}$), 29.0 (*C-7*$_{exo}$), 27.4 (*C-11*$_{endo}$), 27.0 (*C-11*$_{exo}$).

IR (neat, cm^{-1}): 2961, 2877, 2236, 1680, 1480, 1459, 1366, 1174, 1124 (fingerprint region excluded).

HRMS (ESI+): calculated for $C_{13}H_{20}ON$ (M+H$^+$): 206.1539 Found: 206.1542.

1-Benzoylbicyclo[2.2.1]heptane-2-carbonitrile (2n)

Prepared according to **GP 4** using housane **1n** (34.4 mg, 0.20 mmol, 1.0 eq) and acrylonitrile (33 μL, 0.50 mmol, 2.5 eq). The crude residue was purified by column chromatography on silica gel (hexane:EtOAc = 19:1 to 9:1) to afford two diastereoisomers of the norbornane **2n** (29.3 mg, 0.13 mmol, 65% combined yield; *exo*:*endo* = 1:1.9). The diastereoisomers were partially separable. Therefore, each isomer was separated for NMR analysis.

ENDO DIASTEREOISOMER (2n-*endo*)

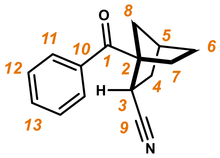

R_f = 0.25. (Hexane:EtOAc = 9:1). Colourless oil.

^{1}H NMR (400 MHz, $CDCl_3$) δ 7.79 – 7.70 (m, 2H, 2*H-11*), 7.51 – 7.43 (m, 1H, *H-13*), 7.38 (ddt, *J* = 8.3, 6.7, 1.2 Hz, 2H, 2*H-12*), 3.24 (ddd, *J* = 12.2, 5.1, 2.6 Hz, 1H, *H-3*), 2.50 (tt, *J* = 4.0, 1.7 Hz, 1H, *H-5*), 2.25 – 2.10 (m,

3H, *2H-7* + *H-4a*), 2.08 (ddd, *J* = 10.2, 2.6, 1.8 Hz, 1H, *H-8a*), 1.92 – 1.82 (m, 1H, *H-6a*), 1.75 (dq, *J* = 10.3, 2.1 Hz, 1H, *H-8b*), 1.61 – 1.53 (m, 2H, *H-4b* + *H-6b*).

^{13}C{H} NMR (101 MHz, $CDCl_3$) δ 202.6 (*C-1*), 137.2 (*C-10*), 132.4 (*C-13*), 128.6 (*2C-12*), 128.2 (*2C-11*), 121.9 (*C-9*), 62.6 (*C-2*), 44.2(*C-8*), 38.1 (*C-5*), 35.3 (*C-4*), 33.9 (*C-3*), 30.2 (*C-6*), 30.0 (*C-7*).

IR (neat, cm^{-1}): 2957, 2923, 2237, 1662, 1597, 1446, 1288, 1236, 1215, 1177 (fingerprint region excluded).

HRMS (ESI+): calculated for $C_{15}H_{15}ONNa$ (M+Na$^+$): 248.1046 Found: 248.1044.

EXO DIASTEREOISOMER (2n-*exo*)

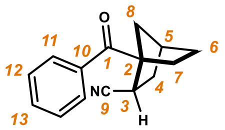

R_f = 0.23. (Hexane:EtOAc = 9:1). Colourless oil.

^{1}H NMR (400 MHz, $CDCl_3$) δ 7.81 – 7.73 (m, 2H, 2*H-11*), 7.53 – 7.44 (m, 1H, *H-13*), 7.39 (dd, *J* = 8.3, 6.8 Hz, 2H, 2*H-12*), 2.94 (ddd, *J* = 9.2, 4.7, 1.7 Hz, 1H, *H-3*), 2.45 (t, *J* = 4.3 Hz, 1H, *H-5*), 1.99 (tddt, *J* = 10.6, 8.6, 5.6, 2.9 Hz, 4H, *2H-8* + *H-4a* + *H-6a*), 1.91 – 1.73 (m, 3H, *H-4b* + *H-6b* + *H-7a*), 1.49 – 1.38 (m, 1H, *H-7b*).

^{13}C{H} NMR (101 MHz, $CDCl_3$) δ 201.9 (*C-1*), 137.7 (*C-10*), 132.7 (*C-13*), 128.7 (*2C-12*), 128.3 (*2C-11*), 121.6 (*C-9*), 63.6 (*C-2*), 40.8 (*C-8*), 36.6 (*C-4*), 36.1 (*C-5*), 34.9 (*C-3*), 34.4 (*C-6*), 30.0 (*C-7*).

IR (neat, cm^{-1}): 2959, 2925, 2238, 1665, 1589, 1435, 1275, 1218, 1200, 1169 (fingerprint region excluded).

HRMS (ESI+): calculated for $C_{15}H_{15}ONNa$ (M+Na$^+$): 248.1046 Found: 248.1039.

1-(4-Fluorobenzoyl)bicyclo[2.2.1]heptane-2-carbonitrile (2o)

Prepared according to **GP 4** using housane **1o** (38 mg, 0.20 mmol, 1.0 eq) and acrylonitrile (33 μL, 0.50 mmol, 2.5 eq). The crude residue was purified by column chromatography on silica gel (hexane:EtOAc = 9:1 to 4:1) to afford two diastereoisomers of the norbornane **2o** (41.8 mg, 0.17 mmol, 86% combined yield; *exo*:*endo* = 1:1.7). The diastereoisomers were partially separable. Therefore, each isomer was separated for NMR analysis.

ENDO DIASTEREOISOMER (2o-*endo*)

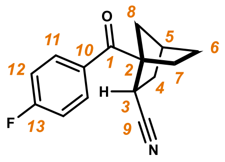

R_f = 0.28. (Hexane:Et_2O = 2:1). White crystalline solid.

^{1}H NMR (500 MHz, $CDCl_3$) δ 7.85 – 7.79 (m, 2H, 2*H-11*), 7.10 – 7.02 (m, 2H, 2*H-12*), 3.21 (ddd, *J* = 12.2, 5.0, 2.8 Hz, 1H, *H-3*), 2.53 – 2.48 (m, 1H, *H-5*), 2.23 (dddd, *J* = 14.4, 9.1, 5.4, 2.3 Hz, 1H, *H-7a*), 2.19 – 2.08 (m, 2H, *H-4a* + *H-7b*), 2.05 (dt, *J* = 10.3, 2.1 Hz, 1H, *H-8a*), 1.88 (tddd, *J* = 12.6, 5.4, 4.3, 3.1 Hz, 1H, *H-6a*), 1.76 (dq, *J* = 10.2, 2.1 Hz, 1H, *H-8b*), 1.59 (dtd, *J* = 12.6, 4.9, 2.5 Hz, 2H, *H-6b* + *H-4b*).

^{13}C{H} NMR (126 MHz, $CDCl_3$) δ 200.6 (*C-1*), 165.3 (d, *J* = 254.5 Hz, *C-13*), 133.2 (d, *J* = 3.3 Hz, *C-10*), 131.2 (d, *J* = 9.1 Hz, *C-11*), 121.9 (*C-9*), 115.8 (d, *J* = 21.9 Hz, *C-12*), 62.5 (*C-2*), 44.3 (*C-8*), 38.0 (*C-5*), 35.3 (*C-4*), 34.1 (*C-3*), 30.3 (*C-6*), 30.1 (*C-7*).

IR (neat, cm^{-1}): 2956, 2237, 1664, 1596, 1505, 1311, 1291, 1277, 1235, 1153 (fingerprint region excluded).

HRMS (ESI+): calculated for $C_{15}H_{14}ONFNa$ (M+Na^+): 266.0952 Found: 266.0948.

Melting Point (from hexane:EtOAc): 91 – 93 °C.

EXO DIASTEREOISOMER (2o-*exo*)

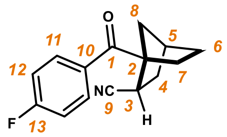

R_f = 0.25. (Hexane:Et_2O = 2:1). Colourless oil.

^{1}H NMR (400 MHz, $CDCl_3$) δ 7.88 – 7.78 (m, 2H, 2*H-11*), 7.12 – 7.01 (m, 2H, 2*H-12*), 2.93 (ddd, *J* = 9.1, 4.7, 1.7 Hz, 1H, *H-3*), 2.45 (td, *J* = 4.3, 2.0 Hz, 1H, *H-5*), 2.05 – 1.92 (m, 4H, *H-4a* + *H-6a* + 2*H-8*), 1.88 (ddd, *J* = 12.9, 9.1, 2.3 Hz, 1H, *H-4b*), 1.84 – 1.71 (m, 2H, *H-7a* + *H-6b*), 1.44 (ddt, *J* = 13.7, 10.5, 4.0 Hz, 1H, *H-7b*)

^{13}C{H} NMR (101 MHz, $CDCl_3$) δ 200.0 (*C-1*), 165.5 (d, *J* = 254.8 Hz, *C-13*), 133.8 (d, *J* = 3.3 Hz, *C-10*), 131.1 (d, *J* = 9.2 Hz, *C-11*), 121.5 (*C-9*), 115.9 (d, *J* = 21.9 Hz, *C-12*), 63.5 (*C-2*), 40.9 (*C-8*), 36.6 (*C-4*), 36.0 (*C-5*), 35.0 (*C-3*), 34.3 (*C-6*), 30.0 (*C-7*).

IR (neat, cm^{-1}): 2967, 2932, 1702, 1478, 1465, 1366, 1073 (fingerprint region excluded).

HRMS (ESI+): calculated for $C_{15}H_{14}ONFNa$ (M+Na^+): 266.0952 Found: 266.0950.

1-(4-Methoxybenzoyl)bicyclo[2.2.1]heptane-2-carbonitrile (2p)

Prepared according to **GP 4** using housane **1p** (40.5 mg, 0.20 mmol, 1.0 eq) and acrylonitrile (33 μL, 0.50 mmol, 2.5 eq). The crude residue was purified by column chromatography on silica gel (hexane:EtOAc = 4:1 to 3:1) to afford two diastereoisomers of the norbornane **2p** (38 mg, 0.15 mmol, 75% combined yield; *exo*:*endo* = 1:2). The diastereoisomers were partially separable. Therefore, each isomer was separated for NMR analysis.

ENDO DIASTEREOISOMER (2p-*endo*)

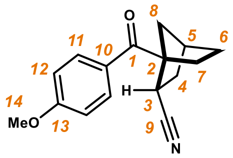

R_f = 0.2. (Hexane:EtOAc = 4:1). Pale-yellow oil.

^{1}H NMR (500 MHz, $CDCl_3$) δ 7.84 (d, *J* = 8.9 Hz, 2H, *2H-11*), 6.86 (d, *J* = 8.9 Hz, 2H, *2H-12*), 3.80 (s, 3H, *3H-14*), 3.20 (ddd, *J* = 12.2, 5.1, 2.6 Hz, 1H, *H-3*), 2.49 (td, *J* = 4.2, 2.0 Hz, 1H, *H-5*), 2.27 – 2.17 (m, 2H, *2H-7*), 2.17 – 2.10 (m, 1H, *H-4a*), 2.10 – 2.06 (m, 1H, *H-8a*), 1.93 – 1.84 (m, 1H, *H-6a*), 1.74 (dq, *J* = 10.3, 2.0 Hz, 1H, *H-8b*), 1.58 (tdd, *J* = 13.0, 4.8, 2.5 Hz, 2H, *H-6b* + *H-4b*).

^{13}C{H} NMR (126 MHz, $CDCl_3$) δ 199.9 (*C-1*), 163.2 (*C-13*), 131.2 (*2C-11*), 129.4 (*C-10*), 122.2 (*C-9*), 113.8 (*2C-12*), 62.4 (*C-2*), 55.6 (*C-14*), 44.3 (*C-8*), 38.0 (*C-5*), 35.2 (*C-4*), 34.2 (*C-3*), 30.4 (*C-6*), 30.3 (*C-7*).

IR (neat, cm^{-1}): 2955, 2925, 2876, 2236, 1653, 1596 (fingerprint region excluded).

HRMS (ESI+): calculated for $C_{16}H_{18}O_2N$ (M+H+): 256.1332 Found: 256.1338.

EXO DIASTEREOISOMER (2p-*exo*)

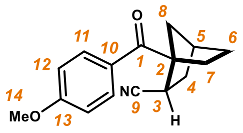

R_f = 0.18. (Hexane:EtOAc = 4:1). Pale-yellow oil.

^{1}H NMR (500 MHz, $CDCl_3$) δ 7.82 (d, *J* = 8.9 Hz, 2H, *2H-11*), 6.87 (d, *J* = 8.9 Hz, 2H, *2H-12*), 3.80 (s, 3H, *3H-14*), 2.95 (ddd, *J* = 9.3, 4.7, 1.5 Hz, 1H, *H-3*), 2.47 – 2.38 (m, 1H, *H-5*), 2.04 – 1.92 (m, 4H, *2H-8* + *H-4a* + *H-6a*), 1.87 (ddd, *J* = 12.9, 9.1, 2.1 Hz, 1H, *H-4b*), 1.83 – 1.74 (m, 2H, *H-7a* + *H-6b*), 1.47 – 1.39 (m, 1H, *H-7b*).

$^{13}C\{H\}$ NMR (126 MHz, $CDCl_3$) δ 199.5 (*C-1*), 163.3 (*C-13*), 131.0 (2*C-11*), 130.1 (*C-10*), 121.7 (*C-9*), 113.9 (2*C-12*), 63.4 (*C-2*), 55.6 (*C-14*), 40.9 (*C-8*), 36.7 (*C-4*), 35.9 (*C-5*), 35.2 (*C-3*), 34.3 (*C-6*), 30.1 (*C-7*).

IR (neat, cm^{-1}): 2959, 2929, 2868, 2235, 1654, 1599 (fingerprint region excluded).

HRMS (ESI+): calculated for $C_{16}H_{18}O_2N$ (M+H+): 256.1332 Found: 256.1339.

1-(Furan-3-carbonyl)bicyclo[2.2.1]heptane-2-carbonitrile (2q)

Prepared according to **GP 4** using housane **1q** (32 mg, 0.20 mmol, 1.0 eq) and acrylonitrile (33 µL, 0.50 mmol, 2.5 eq). The crude residue was purified by column chromatography on silica gel (hexane:EtOAc = 4:1 to 3:1) to afford two diastereoisomers of the norbornane **2q** (21 mg, 0.10 mmol, 49% combined yield; *exo*:*endo* = 1:1.6). The diastereoisomers were partially separable. Therefore, each isomer was separated for NMR analysis.

ENDO DIASTEREOISOMER (2q-*endo*)

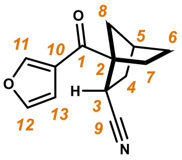

R_f = 0.4. (Hexane:EtOAc = 3:1). Pale-yellow oil.

^1H NMR (500 MHz, $CDCl_3$) δ 8.02 (t, *J* = 1.1 Hz, 1H, *H-11*), 7.37 (t, *J* = 1.7 Hz, 1H, *H-12*), 6.74 (dd, *J* = 2.1, 0.8 Hz, 1H, *H-13*), 3.12 (ddd, *J* = 12.3, 5.0, 2.8 Hz, 1H, *H-3*), 2.48 (dq, *J* = 4.0, 2.0 Hz, 1H, *H-5*), 2.23 (dddd, *J* = 14.0, 9.1, 5.1, 2.3 Hz, 1H, *H-7a*), 2.17 – 2.06 (m, 2H, *H-4a* + *H-7b*), 1.93 – 1.84 (m, 2H, *H-8a* + *H-6a*), 1.71 (dq, *J* = 10.3, 2.1 Hz, 1H, *H-8b*), 1.62 – 1.55 (m, 2H, *H-6b* + *H-4b*).

$^{13}C\{H\}$ NMR (126 MHz, $CDCl_3$) δ 195.3 (*C-1*), 147.2 (*C-11*), 143.7 (*C-12*), 125.5 (*C-10*), 121.8 (*C-9*), 109.9 (*C-13*), 62.6 (*C-3*), 43.7 (*C-8*), 37.8 (*C-5*), 35.5 (*C-4*), 33.3 (*C-3*), 30.14 (*C-6*), 30.11 (*C-7*).

IR (neat, cm^{-1}): 2955, 2928, 2237, 1661, 1595, 1440 (fingerprint region excluded).

HRMS (ESI+): calculated for $C_{13}H_{13}O_2NNa$ (M+Na$^+$): 238.0838 Found: 238.0845.

EXO DIASTEREOISOMER (2q-*exo*)

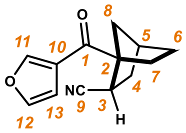

R_f = 0.3. (Hexane:EtOAc = 3:1). Brownish-white solid.

^{1}H NMR (500 MHz, $CDCl_3$) δ 8.02 – 7.95 (m, 1H, *H-11*), 7.41 – 7.34 (m, 1H, *H-12*), 6.76 (dd, *J* = 2.0, 0.8 Hz, 1H, *H-13*), 2.83 (ddd, *J* = 9.1, 4.7, 1.8 Hz, 1H, *H-3*), 2.52 – 2.44 (m, 1H, *H-5*), 2.04 – 2.00 (m, 1H, *H-8a*), 1.99 – 1.94 (m, 1H, *H-4a*), 1.93 – 1.82 (m, 3H, *H-6a* + *H-8b* + *H-4b*), 1.77 (ttd, *J* = 12.3, 4.5, 3.0 Hz, 1H, *H-7a*), 1.67 – 1.61 (m, 1H, *H-6b*), 1.44 – 1.38 (m, 1H, *H-7b*).

^{13}C{H} NMR (126 MHz, $CDCl_3$) δ 194.9 (*C-1*), 146.8 (*C-11*), 143.9 (*C-12*), 126.2 (*C-10*), 121.6 (*C-9*), 109.8 (*C-13*), 63.3 (*C-2*), 39.9 (*C-8*), 36.6 (*C-5*), 36.2 (*C-4*), 34.8 (*C-3*), 34.2 (*C-6*), 29.7 (*C-7*).

IR (neat, cm^{-1}): 2959, 2929, 22367, 1665, 1600, 1444 (fingerprint region excluded).

HRMS (ESI+): calculated for $C_{13}H_{13}O_2NNa$ ($M+Na^+$): 238.0838 Found: 238.0846.

Melting Point (from hexane:EtOAc): 109 – 111 °C.

1-(Thiophene-2-carbonyl)bicyclo[2.2.1]heptane-2-carbonitrile (2r)

Prepared according to **GP 4** using housane **1r** (35.7 mg, 0.20 mmol, 1.0 eq) and acrylonitrile (33 µL, 0.50 mmol, 2.5 eq). The crude residue was purified by column chromatography on silica gel (hexane:EtOAc = 6:1 to 4:1 to 2:1) to afford two diastereoisomers of the norbornane **2r** (14 mg, 0.06 mmol, 30% combined yield; *exo*:*endo* = 1:1.9). The diastereoisomers were partially separable. Therefore, each isomer was separated for NMR analysis.

ENDO DIASTEREOISOMER (2r-*endo*)

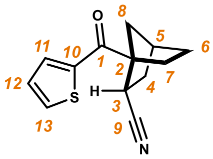

R_f = 0.5. (Hexane:EtOAc = 3:1). Colourless oil.

^{1}H NMR (400 MHz, $CDCl_3$) δ 7.75 (dd, *J* = 3.9, 1.1 Hz, 1H, *H-13*), 7.58 (dd, *J* = 5.0, 1.1 Hz, 1H, *H-11*), 7.07 (dd, *J* = 5.0, 3.8 Hz, 1H, *H-12*), 3.17 (ddd, *J* = 12.2, 5.0, 2.5 Hz, 1H, *H-3*), 2.51 (td, *J* = 4.2, 2.0 Hz, 1H, *H-5*), 2.35 – 2.22 (m, 2H, *H-7a* + *H-4a*), 2.19 – 2.06 (m, 2H, *H-7b* + *H-8a*), 1.98 – 1.86 (m, 1H, *H-6a*), 1.77 (dq, *J* = 10.3, 2.0 Hz, 1H, *H-8b*), 1.66 – 1.55 (m, 2H, *H-6b* + *H-4b*).

^{13}C{H} NMR (101 MHz, $CDCl_3$) δ 193.3 (*C-1*), 142.3 (*C-10*), 133.8 (*C-11*), 132.7 (*C-13*), 128.2 (*C-12*), 121.8 (*C-9*), 62.2 (*C-2*), 44.3 (*C-8*), 38.0 (*C-5*), 35.5 (*C-4*), 33.7 (*C-3*), 30.8 (*C-6*), 30.2 (*C-7*).

IR (neat, cm^{-1}): 2956, 2238, 1637, 1411, 1355, 1304, 1289, 1238, 1217, 1062 (fingerprint region excluded).

HRMS (ESI+): calculated for $C_{13}H_{13}ONNaS$ (M+Na$^+$): 254.0610 Found: 254.0609.

EXO DIASTEREOISOMER (2r-*exo*)

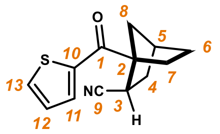

R_f = 0.4. (Hexane:EtOAc = 3:1). Colourless oil.

^{1}H NMR (400 MHz, $CDCl_3$) δ 7.72 (dd, *J* = 3.9, 1.1 Hz, 1H, *H-13*), 7.59 (dd, *J* = 5.0, 1.1 Hz, 1H, *H-11*), 7.08 (dd, *J* = 5.0, 3.9 Hz, 1H, *H-12*), 2.95 (ddd, *J* = 9.1, 4.7, 1.6 Hz, 1H, *H-3*), 2.51 – 2.45 (m, 1H, *H-5*), 2.11 – 1.95 (m, 4H, *H-4a* + *H-6a* + *2H-8*), 1.92 – 1.86 (m, 1H, *H-4b*), 1.86 – 1.72 (m, 2H, *H-6b* + *H-7a*), 1.47 – 1.39 (m, 1H, *H-7b*).

^{13}C{H} NMR (101 MHz, $CDCl_3$) δ 192.7 (*C-1*), 142.6 (*C-10*), 133.9 (*C-11*), 132.4 (*C-13*), 128.2 (*C-12*), 121.5 (*C-9*), 63.0 (*C-2*), 40.4 (*C-8*), 36.5 (*C-4*), 36.4 (*C-5*), 35.2 (*C-3*), 34.6 (*C-6*), 29.9 (*C-7*).

IR (neat, cm^{-1}): 2949, 2229, 1636, 1415, 1309, 1289, 1250, 1208, 1050 (fingerprint region excluded).

HRMS (ESI+): calculated for $C_{13}H_{13}ONNaS$ (M+Na$^+$): 254.0610 Found: 254.0601.

Methyl 1-(4-phenylbutanoyl)bicyclo[2.2.1]heptane-2-carboxylate (2s)

Prepared according to **GP 4** using housane **1a** (43.0 mg, 0.20 mmol, 1.0 eq) and methyl acrylate (45 μL, 0.50 mmol, 2.5 eq). The crude residue was purified by column chromatography on silica gel (hexane:Et_2O = 3:1) to afford two diastereoisomers of the norbornane **2s** (40.2 mg, 0.13 mmol, 67% combined yield; *exo*:*endo* = 1.2:1).

MIXTURE OF DIASTEREOISOMERS (2s-*endo* and 2s-*exo*)

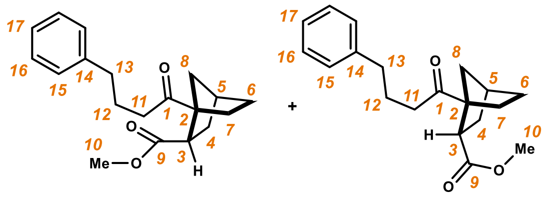

R_f = 0.5. (Hexane:EtOAc = 4:1). Colourless oil.

^{1}H NMR (500 MHz, $CDCl_3$) δ 7.24 – 7.17 (m, 4H, *2H-15*$_{exo}$ + *2H-15*$_{endo}$), 7.15 – 7.06 (m, 6H, *2H-16*$_{exo}$ + *H-17*$_{exo}$ + *2H-16*$_{endo}$ + *H-17*$_{endo}$), 3.53 (s, 6H, *3H-10*$_{exo}$ + *3H-10*$_{endo}$), 3.21 (ddd, *J* = 11.9, 4.9, 2.7 Hz, 1H, *H-3*$_{endo}$), 2.73 – 2.65 (m, 1H, *H-3*$_{exo}$), 2.62 – 2.50 (m, 5H, *2H-13*$_{exo}$ + *H-11a*$_{endo}$ + *2H-13*$_{endo}$), 2.50 – 2.40 (m, 3H, *2H-11*$_{exo}$ + *H-11b*$_{endo}$), 2.33 – 2.25 (m, 2H, *H-5*$_{exo}$ + *H-5*$_{endo}$), 1.98 – 1.93 (m, 1H, *H-8a*$_{exo}$), 1.92 – 1.78 (m, 6H, *2H-12*$_{exo}$+ *H-4a*$_{endo}$ + *H-7a*$_{endo}$ + *2H-12*$_{endo}$), 1.76 – 1.69 (m, 1H, *H-4b*$_{endo}$), 1.69 – 1.42 (m, 9H, *H-8b*$_{exo}$ + *H-6a*$_{exo}$ + *H-7a*$_{exo}$ + *2H-4*$_{exo}$+ *H-6a*$_{endo}$ + *H-7b*$_{endo}$ + *2H-8*$_{endo}$), 1.42 – 1.33 (m, 2H, *H-6b*$_{exo}$ + *H-6b*$_{endo}$), 1.32 – 1.25 (m, 1H, *H-7b*$_{exo}$).

^{13}C{H} NMR (126 MHz, $CDCl_3$) δ 212.8 (*C-1*$_{exo}$), 211.7 (*C-1*$_{endo}$), 175.8 (*C-9*$_{exo}$), 174.0 (*C-9*$_{endo}$), 142.1 (*C-14*$_{exo}$), 142.0 (*C-14*$_{endo}$), 128.60 (*2C-16*$_{exo}$), 128.59 (*2C-16*$_{endo}$), 128.5 (*2C-15*$_{endo}$), 128.4 (*2C-15*$_{exo}$), 126.0 (*C-17*$_{endo}$), 125.9 (*C-17*$_{exo}$), 62.8 (*C-2*$_{exo}$), 62.4 (*C-2*$_{endo}$), 51.81 (*C-10*$_{endo}$), 51.79 (*C-10*$_{exo}$), 48.7 (*C-3*$_{endo}$), 48.3 (*C-3*$_{exo}$), 45.4 (*C-8*$_{endo}$), 39.1 (*C-8*$_{exo}$), 39.0 (*C-11*$_{exo}$), 38.4 (*C-11*$_{endo}$), 38.3 (*C-5*$_{endo}$), 36.3 (*C-5*$_{exo}$), 36.0 (*C-4*$_{exo}$), 35.20 (*C-13*$_{exo}$), 35.17 (*C-13*$_{endo}$), 34.3 (*C-6*$_{exo}$), 33.1 (*C-4*$_{endo}$), 29.6 (*C-6*$_{endo}$), 29.4 (*C-7*$_{exo}$), 26.4 (*C-7*$_{endo}$), 25.1 (*C-12*$_{endo}$), 24.9 (*C-12*$_{exo}$).

IR (neat, cm^{-1}): 2952, 1731, 1697, 1453, 1435 (fingerprint region excluded).

HRMS (ESI+): calculated for $C_{19}H_{25}O_3$ (M+H+): 301.1798 Found: 301.1794.

Cyclohexyl 1-(4-phenylbutanoyl)bicyclo[2.2.1]heptane-2-carboxylate (2t)

Prepared according to **GP 4** using housane **1a** (43.0 mg, 0.20 mmol, 1.0 eq) and cyclohexyl acrylate (79 μL, 0.50 mmol, 2.5 eq). The crude residue was purified by column chromatography on silica gel (hexane:Et_2O = 9:1) to afford two diastereoisomers of the norbornane **2t** (61.2 mg, 0.16 mmol, 83% combined yield; *exo*:*endo* = 1.3:1).

MIXTURE OF DIASTEREOISOMERS (2t-*endo* and 2t-*exo*)

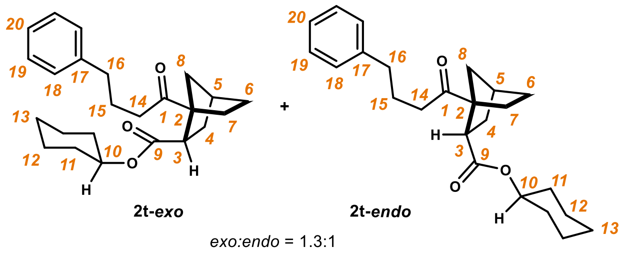

R_f = 0.7. (Hexane:Et_2O = 2:1). Colourless oil.

^{1}H NMR (400 MHz, $CDCl_3$) δ 7.24 – 7.16 (m, 4H, *2H-18$_{exo}$* + *2H-18$_{endo}$*), 7.15 – 7.06 (m, 6H, *2H-19$_{exo}$* + *2H-19$_{endo}$* + *H-20$_{exo}$* + *H-20$_{endo}$*), 4.71 – 4.53 (m, 2H, *H-10$_{exo}$* + *H-10$_{endo}$*), 3.19 (ddd, *J* = 11.6, 5.0, 2.7 Hz, 1H, *H-3$_{endo}$*), 2.66 (ddd, *J* = 9.4, 5.7, 1.6 Hz, 1H, *H-3$_{exo}$*), 2.63 – 2.41 (m, 8H, *2H-14$_{exo}$* + *2H-14$_{endo}$* + *H-16$_{exo}$* + *H-16$_{endo}$*), 2.33 – 2.23 (m, 2H, *H-5$_{exo}$* + *H-5$_{endo}$*), 1.97 – 1.12 (m, 40H, *2H-8$_{exo}$* + *2H-8$_{endo}$* + *2H-4$_{exo}$* + *2H-4$_{endo}$* + *2H-6$_{exo}$* + *2H-6$_{endo}$* + *2H-7$_{exo}$* + *2H-7$_{endo}$* + *2H-15$_{exo}$* + *2H-15$_{endo}$* + *5CH$_{2\ cyclohexyl\ (exo)}$* + *5CH$_{2\ cyclohexyl\ (endo)}$*).

^{13}C{H} NMR (101 MHz, $CDCl_3$) δ 212.6 (*C-1$_{exo}$*), 211.4 (*C-1$_{endo}$*), 174.7 (*C-9$_{exo}$*), 172.8 (*C-9$_{endo}$*), 142.1 (*C-17$_{exo}$*), 142.0 (*C-17$_{endo}$*), 128.61 (*2C-19$_{exo}$*), 128.58 (*2C-19$_{endo}$*), 128.5 (*2C-18$_{endo}$*), 128.4 (*2C-18$_{exo}$*), 126.0 (*C-20$_{endo}$*), 125.9 (*C-20$_{exo}$*), 73.1 (*C-10$_{endo}$*), 72.7 (*C-10$_{exo}$*), 62.6 (*C-2$_{exo}$*), 62.4 (*C-2$_{endo}$*), 49.1 (*C-3$_{endo}$*), 48.8 (*C-3$_{exo}$*), 45.6 (*C-8$_{endo}$*), 39.12 (*C-14$_{exo}$*), 39.10 (*C-8$_{exo}$*), 38.4 (*C-5$_{endo}$*), 38.3 (*C-14$_{endo}$*), 36.24 (*C-4$_{exo}$*), 36.20 (*C-5$_{exo}$*), 35.29 (*C-16$_{endo}$*), 35.25 (*C-16$_{exo}$*), 34.4 (*C-4$_{endo}$*), 32.8 (*C-6$_{endo}$*), 31.7, 31.6, 31.6, 29.6, 29.4, 26.1, 25.5, 25.4, 25.2, 24.9, 23.9, 23.7, 23.7.

NOTE: Only diagnostic signals are assigned.

IR (neat, cm^{-1}): 2937, 2860, 1722, 1701, 1451, 1358, 1192 (fingerprint region excluded).

HRMS (ESI+): calculated for $C_{24}H_{32}O_3Na$ ($M+Na^+$): 391.2244 Found: 391.2242.

Prop-2-yn-1-yl 1-(4-phenylbutanoyl)bicyclo[2.2.1]heptane-2-carboxylate (2u)

Prepared according to **GP 4** using housane **1a** (43.0 mg, 0.20 mmol, 1.0 eq) and propargyl acrylate (56 µL, 0.50 mmol, 2.5 eq). The crude residue was purified by column chromatography on silica gel (hexane:Et_2O = 4:1) to afford two diastereoisomers of the norbornane **2u** (33.5 mg, 0.15 mmol, 77% combined yield; *exo*:*endo* = 1:1).

MIXTURE OF DIASTEREOISOMERS (2u-*endo* and 2u-*exo*)

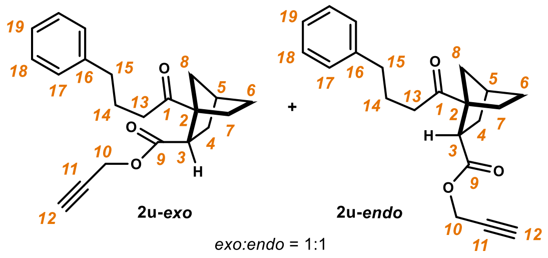

R_f = 0.22. (Hexane:Et_2O = 4:1). Colourless oil.

^{1}H NMR (400 MHz, $CDCl_3$) δ 7.24 – 7.17 (m, 4H, *2H-17*$_{endo}$ + *2H-17*$_{exo}$), 7.16 – 7.05 (m, 6H, *2H-18*$_{endo}$ + *H-19*$_{endo}$ + *2H-18*$_{exo}$ + *H-19*$_{exo}$), 4.65 – 4.42 (m, 4H, *2H-10*$_{endo}$ + *2H-10*$_{exo}$), 3.26 (ddd, *J* = 11.8, 4.8, 2.7 Hz, 1H, *H-3*$_{endo}$), 2.73 (ddd, *J* = 9.0, 5.9, 1.5 Hz, 1H, *H-3*$_{exo}$), 2.64 – 2.44 (m, 8H, *2H-13*$_{endo}$ + *2H-15*$_{endo}$ + *2H-13*$_{exo}$ + *2H-15*$_{exo}$), 2.36 (t, *J* = 2.5 Hz, 1H, *H-12*$_{exo}$), 2.34 – 2.28 (m, 3H, *H-12*$_{endo}$ + *H-5*$_{endo}$ + *H-5*$_{exo}$), 2.00 – 1.94 (m, 1H, *H-8a*$_{exo}$), 1.93 – 1.79 (m, 6H, *H-4a*$_{endo}$ + *H-7a*$_{endo}$ + *2H-14*$_{endo}$ + *2H-14*$_{exo}$), 1.78 – 1.56 (m, 8H, *H-8a*$_{endo}$ + *H-4b*$_{endo}$ + *H-6a*$_{endo}$ + *H-7b*$_{endo}$ + *H-6a*$_{exo}$ + *H-7a*$_{exo}$ + *2H-4*$_{exo}$), 1.56 – 1.52 (m, 1H, *H-8b*$_{exo}$), 1.50 – 1.46 (m, 1H, *H-8b*$_{endo}$), 1.38 (dtt, *J* = 11.8, 4.1, 2.5 Hz, 2H, *H-6b*$_{endo}$ + *H-6b*$_{exo}$), 1.33 – 1.26 (m, 1H, *H-7b*$_{exo}$).

^{13}C{H} NMR (126 MHz, $CDCl_3$) δ 212.6 (*C-1*$_{exo}$), 211.5 (*C-1*$_{endo}$), 174.5 (*C-9*$_{exo}$), 172.8 (*C-9*$_{endo}$), 142.1 (*C-16*$_{endo}$ + *C-16*$_{exo}$), 128.6 (*2C-18*$_{endo}$ + *2C-18*$_{exo}$), 128.5 (*2C-17*$_{endo}$), 128.4 (*2C-17*$_{exo}$), 126.0 (*C-19*$_{endo}$), 125.9 (*C-19*$_{exo}$), 77.8 (*C-11*$_{exo}$), 77.7 (*C-11*$_{endo}$), 75.02 (*C-12*$_{endo}$), 74.95 (*C-12*$_{exo}$), 62.9 (*C-2*$_{exo}$), 62.4 (*C-2*$_{endo}$), 52.11 (*C-10*$_{exo}$), 52.07 (*C-10*$_{endo}$), 48.5 (*C-3*$_{endo}$), 48.0 (*C-3*$_{exo}$), 45.5 (*C-8*$_{endo}$), 39.3 (*C-8*$_{exo}$), 38.9 (*C-13*$_{exo}$), 38.7 (*C-13*$_{endo}$), 38.3 (*C-5*$_{endo}$), 36.4 (*C-5*$_{exo}$), 35.9 (*C-4*$_{exo}$), 35.21 (*C-15*$_{endo}$), 35.19 (*C-15*$_{exo}$), 34.4 (*C-6*$_{exo}$), 33.1 (*C-4*$_{endo}$), 29.6 (*C-6*$_{endo}$), 29.4 (*C-7*$_{exo}$), 26.5 (*C-7*$_{endo}$), 25.1 (*C-14*$_{endo}$), 24.9 (*C-14*$_{exo}$).

IR (neat, cm^{-1}): 2952, 2877, 2236, 1699, 1454, 1368 (fingerprint region excluded).

HRMS (ESI+): calculated for $C_{14}H_{20}NO$ (M+H+): 218.1539 Found: 218.1540.

2,2,2-Trifluoroethyl 1-(4-phenylbutanoyl)bicyclo[2.2.1]heptane-2-carboxylate (2v)

Prepared according to **GP 4** using housane **1a** (43 mg, 0.20 mmol, 1.0 eq) and 2,2,2-trifluoroethyl acrylate (69 µL, 0.50 mmol, 2.5 eq). The crude residue was purified by column chromatography on silica gel (hexane:Et_2O = 9:1) to afford two diastereoisomers of the norbornane **2v** (51 mg, 0.14 mmol, 69% combined yield; *exo*:*endo* = 1:1).

MIXTURE OF DIASTEREOISOMERS (2v-*endo* and 2v-*exo*)

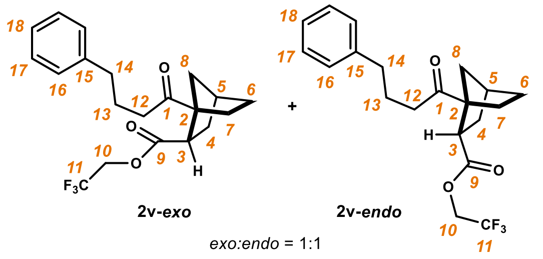

R_f = 0.43. (Hexane:Et_2O = 4:1). Colourless oil.

^{1}H NMR (500 MHz, $CDCl_3$) δ 7.25 – 7.16 (m, 4H, *2H-16$_{endo}$* + *2H-16$_{exo}$*), 7.16 – 7.05 (m, 6H, *2H-17$_{endo}$* + *H-18$_{endo}$* + *2H-17$_{exo}$* + *H-18$_{exo}$*), 4.47 – 4.27 (m, 3H, *2H-10$_{endo}$* + *H-10a$_{exo}$*), 4.21 (dq, *J* = 12.7, 8.4 Hz, 1H, *H-10b$_{exo}$*), 3.33 (ddd, *J* = 11.9, 4.8, 2.6 Hz, 1H, *H-3$_{endo}$*), 2.76 (ddd, *J* = 9.5, 5.7, 1.5 Hz, 1H, *H-3$_{exo}$*), 2.59 – 2.42 (m, 8H, *2H-12$_{endo}$* + *2H-14$_{endo}$* + *2H-12$_{exo}$* + *2H-14$_{exo}$*), 2.37 – 2.30 (m, 2H, *H-5$_{endo}$* + *H-5$_{exo}$*), 1.98 – 1.94 (m, 1H, *H-8a$_{exo}$*), 1.93 – 1.80 (m, 6H, *H-4a$_{endo}$* + *H-7a$_{endo}$* + *2H-13$_{endo}$* + *2H-13$_{exo}$*), 1.76 – 1.69 (m, 2H, *H-4b$_{endo}$* + *H-4a$_{exo}$*), 1.69 – 1.48 (m, 8H, *H-6a$_{endo}$* + *H-7b$_{endo}$* + *2H-8$_{endo}$* + *H-8b$_{exo}$* + *H-4b$_{exo}$* + *H-6a$_{exo}$* + *H-7a$_{exo}$*), 1.41 – 1.27 (m, 3H, *H-6b$_{endo}$* + *H-6b$_{exo}$* + *H-7b$_{exo}$*).

^{13}C{H} NMR (126 MHz, $CDCl_3$) δ 212.5 (*C-1$_{exo}$*), 211.0 (*C-1$_{endo}$*), 173.8 (*C-9$_{exo}$*), 172.1 (*C-9$_{endo}$*), 141.9 (*C-15$_{endo}$* + *C-15$_{exo}$*), 128.6 (*2C-17$_{exo}$*), 128.6 (*2C-17$_{endo}$*), 128.5 (*2C-16$_{endo}$*), 128.4 (*2C-16$_{exo}$*), 126.0 (*C-18$_{endo}$* + *C-18$_{exo}$*), 123.1 (q, $^1J_{C-F}$ = 277.3 Hz, *C-11$_{exo}$*), 123.0 (q, $^1J_{C-F}$ = 277.7 Hz, *C-11$_{endo}$*), 63.2 (*C-2$_{exo}$*), 62.4 (*C-2$_{endo}$*), 60.33 (q, $^2J_{C-F}$ = 36.7 Hz, *C-10$_{exo}$*), 60.31 (q, $^2J_{C-F}$ = 36.6 Hz, *C-10$_{endo}$*), 48.0 (*C-3$_{endo}$*), 47.5 (*C-3$_{exo}$*), 45.5 (*C-8$_{endo}$*), 39.5 (*C-8$_{exo}$*), 38.6 (*C-12$_{exo}$*), 38.5 (*C-12$_{endo}$*), 38.2 (*C-5$_{endo}$*), 36.6 (*C-5$_{exo}$*), 36.1 (*C-4$_{exo}$*), 35.13 (*C-14$_{endo}$*), 35.11 (*C-14$_{exo}$*), 34.4 (*C-6$_{exo}$*), 33.2 (*C-4$_{endo}$*), 29.6 (*C-6$_{endo}$*), 29.3 (*C-7$_{exo}$*), 26.6 (*C-7$_{endo}$*), 25.0 (*C-13$_{endo}$*), 24.8 (*C-13$_{exo}$*).

^{19}F NMR (471 MHz, $CDCl_3$) δ -73.6 (t, *J* = 8.6 Hz, *$CF_{3\ endo}$*), -73.7 (t, *J* = 8.3 Hz, *$CF_{3\ exo}$*)

IR (neat, cm^{-1}): 2956, 2874, 1753, 1699, 1496, 1453 (fingerprint region excluded).

HRMS (ESI+): calculated for $C_{20}H_{24}O_3F_3$ (M+H+): 349.1565 Found: 349.1561.

2-Methoxyethyl 1-(4-phenylbutanoyl)bicyclo[2.2.1]heptane-2-carboxylate (2w)

Prepared according to **GP 4** using housane **1a** (43.0 mg, 0.20 mmol, 1.0 eq) and 2-methoxyethyl acrylate (64 µL, 0.50 mmol, 2.5 eq). The crude residue was purified by column chromatography on silica gel (hexane:Et_2O = 4:1) to afford two diastereoisomers of the norbornane **2w** (46.1 mg, 0.13 mmol, 67% combined yield; *exo*:*endo* = 1:1). The diastereoisomers were partially separable. Therefore, each isomer was separated for NMR analysis.

ENDO DIASTEREOISOMER (2w-*endo*)

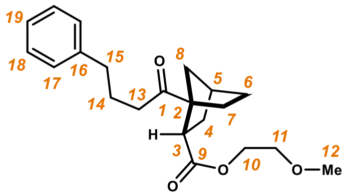

R_f = 0.3. (Hexane:EtOAc = 4:1). Pale-yellow oil.

^{1}H NMR (400 MHz, $CDCl_3$) δ 7.27 – 7.18 (m, 2H, *2H-17*), 7.17 – 7.07 (m, 3H, *2H-18 + H-19*), 4.14 (dt, *J* = 12.0, 4.5 Hz, 1H, *H-10a*), 4.06 (dt, *J* = 12.0, 4.7 Hz, 1H, *H-10b*), 3.47 – 3.39 (m, 2H, *2H-11*), 3.33 – 3.20 (m, 4H, *H-3 + 3H-12*), 2.66 – 2.53 (m, 3H, *H-13a + 2H-15*), 2.53 – 2.42 (m, 1H, *H-13b*), 2.35 – 2.27 (m, 1H, *H-5*), 1.95 – 1.80 (m, 4H, *H-4a + H-7a + 2H-14*), 1.74 (ddd, *J* = 12.5, 5.0, 2.6 Hz, 1H, *H-4b*), 1.67 – 1.49 (m, 3H, *H-6a + H-8a + H-7b*), 1.49 – 1.44 (m, 1H, *H-8b*), 1.40 – 1.32 (m, 1H, *H-6b*).

^{13}C{H} NMR (126 MHz, $CDCl_3$) δ 211.7 (*C-1*), 173.5 (*C-9*), 142.1 (*C-16*), 128.6 (*2C-18*), 128.5 (*2C-17*), 126.0 (*C-19*), 70.4 (*C-11*), 63.6 (*C-10*), 62.4 (*C-2*), 58.9 (*C-12*), 48.8 (*C-3*), 45.4 (*C-8*), 38.5 (*C-13*), 38.3 (*C-5*), 35.3 (*C-15*), 33.0 (*C-4*), 29.6 (*C-6*), 26.3 (*C-7*), 25.2 (*C-14*).

IR (neat, cm^{-1}): 2951, 1730, 1697, 1453 (fingerprint region excluded).

HRMS (ESI+): calculated for $C_{21}H_{29}O_4$ (M+H+): 345.2060 Found: 345.2056.

EXO DIASTEREOISOMER (2w-*exo*)

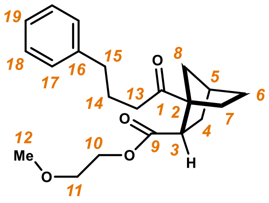

R_f = 0.26. (Hexane:EtOAc = 4:1). Pale-yellow oil.

^{1}H NMR (500 MHz, $CDCl_3$) δ 7.25 – 7.17 (m, 2H, *2H-17*), 7.16 – 7.05 (m, 3H, *2H-18 + H-19*), 4.08 (qdd, *J* = 12.0, 5.8, 3.8 Hz, 2H, *2H-10*), 3.47 (qdd, *J* = 11.1, 7.8, 4.4 Hz, 2H, *2H-11*), 3.29 (s, 3H, *3H-12*), 2.77 – 2.70 (m, 1H, *H-3*), 2.58 – 2.52 (m, 2H, *2H-15*), 2.51 – 2.44 (m, 2H, *2H-13*), 2.32 – 2.25 (m, 1H, *H-5*), 1.99 – 1.94 (m, 1H, *H-8a*), 1.91 – 1.78 (m, *J* = 6.9 Hz, 2H, *2H-14*), 1.74 – 1.65 (m, 2H, *2H-4*), 1.65 – 1.55 (m, 2H, *H-6a + H-7a*), 1.55 – 1.50 (m, 1H, *H-8b*), 1.38 (ddd, *J* = 12.0, 7.0, 4.1 Hz, 1H, *H-6b*), 1.28 (ddt, *J* = 9.2, 7.0, 2.7 Hz, 1H, *H-7b*).

^{13}C{H} NMR (126 MHz, $CDCl_3$) δ 212.7 (*C-1*), 175.4 (*C-9*), 142.1 (*C-16*), 128.6 (*2C-18*), 128.4 (*2C-17*), 125.9 (*C-19*), 70.6 (*C-11*), 63.5 (*C-10*), 62.9 (*C-2*), 59.1 (*C-12*), 48.3 (*C-3*), 39.2 (*C-8*), 39.0 (*C-13*), 36.3 (*C-5*), 36.1 (*C-4*), 35.2 (*C-15*), 34.3 (*C-6*), 29.4 (*C-7*), 24.9 (*C-14*).

IR (neat, cm^{-1}): 2955, 1729, 1700, 1455 (fingerprint region excluded).

HRMS (ESI+): calculated for $C_{21}H_{29}O_4$ (M+H+): 345.2060 Found: 345.2058.

Cyclohexyl 1-(4-phenylbutanoyl)bicyclo[2.2.1]heptane-2-carboxylate (2x)

Prepared according to **GP 4** using housane **1a** (43.0 mg, 0.20 mmol, 1.0 eq) and 2-(dimethylamino)ethyl acrylate (76 µL, 0.50 mmol, 2.5 eq). The crude residue was purified by column chromatography on silica gel (CH_2Cl_2:MeOH = 49:1 to 19:1 to 9:1) to afford two diastereoisomers of the norbornane **2x** (29.3 mg, 0.08 mmol, 41% combined yield; *exo*:*endo* = 1:1.3).

MIXTURE OF DIASTEREOISOMERS (2x-*endo* and 2x-*exo*)

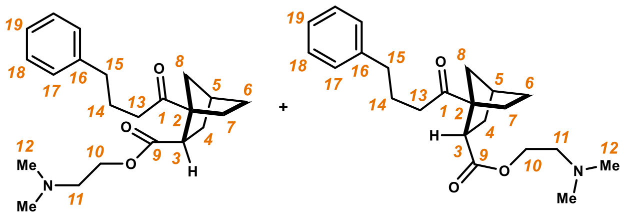

R_f (**2x-*endo***) = 0.75 and R_f (**2x-*exo***) = 0.72. (CH_2Cl_2:MeOH = 9:1). Colourless oil.

^{1}H NMR (400 MHz, $CDCl_3$) 7.27 – 7.16 (m, 4H, *2H-17$_{exo}$* + *2H-17$_{endo}$*), 7.11 (dtt, *J* = 7.5, 4.8, 2.1 Hz, 6H, *2H-18$_{exo}$* + *2H-18$_{endo}$* + *H-19$_{exo}$* + *H-19$_{endo}$*), 4.16 – 3.97 (m, 4H, *2H-10$_{exo}$* + *2H-10$_{endo}$*), 3.23 (ddd, *J* = 11.8, 4.9, 2.6 Hz, 1H, *H-3$_{endo}$*), 2.75 – 2.68 (m, 1H, *H-3$_{exo}$*), 2.64 – 2.37 (m, 12H, *2H-11$_{exo}$* + *2H-11$_{endo}$* + *2H-13$_{exo}$* + *2H-13$_{endo}$* + *2H-15$_{exo}$* + *2H-15$_{endo}$*), 2.33 – 2.26 (m, 2H, *H-5$_{exo}$* + *H-5$_{endo}$*), 2.19 (s, 6H, *6H-12$_{exo}$*), 2.15 (s, 6H, *6H-12$_{endo}$*), 1.99 – 1.78 (m, 7H, *2H-15$_{exo}$* + *2H-15$_{endo}$* + *H-8a$_{exo}$* + *H-7a$_{endo}$* + *H-4a$_{endo}$*), 1.76 – 1.24 (m, 12H, *2H-7$_{exo}$* + *2H-6$_{endo}$* + *2H-6$_{exo}$* + *2H-8$_{endo}$* + *2H-4$_{exo}$* + *H-7b$_{endo}$* + *H-4b$_{endo}$* + *H-8b$_{exo}$*).

^{13}C{H} NMR (101 MHz, $CDCl_3$) δ 212.7 (*C-1$_{exo}$*), 211.6 (*C-1$_{endo}$*), 175.5 (*C-9$_{exo}$*), 173.5 (*C-9$_{endo}$*), 142.1 (*C-16$_{exo}$*), 142.0 (*C-16$_{endo}$*), 128.61 (*2C-18$_{exo}$*), 128.58 (*2C-18$_{endo}$*), 128.5 (*2C-17$_{endo}$*), 128.4 (*2C-17$_{exo}$*), 126.0 (*C-19$_{endo}$*), 125.9 (*C-19$_{exo}$*), 62.9 (*C-2$_{exo}$*), 62.5 (*C-2$_{endo}$*), 62.4 (*C-10$_{endo}$*), 62.3 (*C-10$_{exo}$*), 57.8 (*C-11$_{exo}$*), 57.8 (*C-11$_{endo}$*), 48.7 (*C-3$_{endo}$*), 48.3 (*C-3$_{exo}$*), 45.83 (*2C-12$_{exo}$*), 45.79 (*2C-12$_{endo}$*), 45.5 (*C-8$_{endo}$*), 39.2 (*C-8$_{exo}$*), 39.0 (*C-13$_{exo}$*), 38.4 (*C-13$_{endo}$*), 38.3 (*C-5$_{endo}$*), 36.3 (*C-5$_{exo}$*), 36.2 (*C-4$_{exo}$*), 35.3 (*C-15$_{endo}$*), 35.2 (*C-15$_{exo}$*), 34.3 (*C-6$_{exo}$*), 33.1 (*C-4$_{endo}$*), 29.6 (*C-6$_{endo}$*), 29.4 (*C-7$_{exo}$*), 26.3 (*C-7$_{endo}$*), 25.2 (*C-15$_{endo}$*), 25.0 (*C-15$_{exo}$*).

IR (neat, cm^{-1}): 2952, 2872, 1732, 1699, 1454, 1178 (fingerprint region excluded).

HRMS (ESI+): calculated for $C_{22}H_{31}O_3N$ (M+H$^+$): 358.2377 Found: 358.2374.

2-Chloroethyl 1-(4-phenylbutanoyl)bicyclo[2.2.1]heptane-2-carboxylate (2y)

Prepared according to **GP 4** using housane **1a** (43.0 mg, 0.20 mmol, 1.0 eq) and 2-chloroethyl acrylate (60 µL, 0.50 mmol, 2.5 eq). The crude residue was purified by column chromatography on silica gel (hexane:Et_2O = 6:1) to afford two diastereoisomers of the norbornane **2y** (42 mg, 0.12 mmol, 60% combined yield; *exo*:*endo* = 1:1.2).

MIXTURE OF DIASTEREOISOMERS (2y-*endo* and 2y-*exo*)

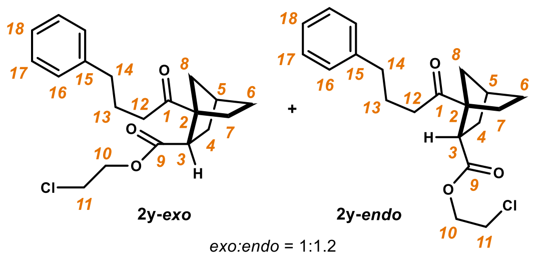

R_f = 0.2. (Hexane:Et_2O = 4:1). Colourless oil.

^{1}H NMR (400 MHz, $CDCl_3$) δ 7.24 – 7.17 (m, 4H, *2H-16*$_{endo}$ + *2H-16*$_{exo}$), 7.16 – 7.06 (m, 6H, *2H-17*$_{endo}$ + *H-18*$_{endo}$ + *2H-17*$_{exo}$ + *H-18*$_{exo}$), 4.29 – 4.09 (m, 4H, *2H-10*$_{endo}$ + *2H-10*$_{exo}$), 3.59 – 3.47 (m, 4H, *2H-11*$_{endo}$ + *2H-11*$_{exo}$), 3.28 (ddd, *J* = 11.8, 4.9, 2.7 Hz, 1H, *H-3*$_{endo}$), 2.72 (ddd, *J* = 9.3, 5.7, 1.5 Hz, 1H, *H-3*$_{exo}$), 2.66 – 2.40 (m, 8H, *2H-12*$_{endo}$ + *2H-14*$_{endo}$ + *2H-12*$_{exo}$ + *2H-14*$_{exo}$), 2.35 – 2.27 (m, 2H, *H-5*$_{endo}$ + *H-5*$_{exo}$), 1.97 (dq, *J* = 9.9, 2.2 Hz, 1H, *H-8a*$_{exo}$), 1.94 – 1.77 (m, 6H, *H-4a*$_{endo}$ + *H-7a*$_{endo}$ + *2H-13*$_{endo}$ + *2H-13*$_{exo}$), 1.77 – 1.58 (m, 7H, *H-6a*$_{endo}$ + *H-4b*$_{endo}$ + *H-8a*$_{endo}$ + *2H-4*$_{exo}$ + *H-6a*$_{exo}$ + *H-7a*$_{exo}$), 1.57 – 1.50 (m, 2H, *H-7b*$_{endo}$ + *H-8b*$_{exo}$), 1.50 – 1.46 (m, 1H, *H-8b*$_{endo}$), 1.43 – 1.33 (m, 2H, *H-6b*$_{endo}$ + *H-6b*$_{exo}$), 1.33 – 1.26 (m, 1H, *H-7b*$_{exo}$).

^{13}C{H} NMR (126 MHz, $CDCl_3$) δ 212.7 (*C-1*$_{exo}$), 211.4 (*C-1*$_{endo}$), 175.0 (*C-9*$_{exo}$), 173.2 (*C-9*$_{endo}$), 142.0 (*C-15*$_{endo}$ + *C-15*$_{exo}$), 128.6 (*2C-17*$_{endo}$ + *2C-17*$_{exo}$), 128.5 (*2C-16*$_{endo}$), 128.4 (*2C-16*$_{exo}$), 125.99 (*C-18*$_{endo}$), 125.96 (*C-18*$_{exo}$), 64.3 (*C-10*$_{endo}$), 64.0 (*C-10*$_{exo}$), 63.0 (*C-2*$_{exo}$), 62.4 (*C-2*$_{endo}$), 48.5 (*C-3*$_{endo}$), 48.1 (*C-3*$_{exo}$), 45.5 (*C-8*$_{endo}$), 41.7 (*C-11*$_{exo}$), 41.6 (*C-11*$_{endo}$), 39.4 (*C-8*$_{exo}$), 38.8 (*C-12*$_{exo}$), 38.5 (*C-12*$_{endo}$), 38.3 (*C-5*$_{endo}$), 36.4 (*C-5*$_{exo}$), 36.1 (*C-4*$_{exo}$), 35.20 (*C-14*$_{endo}$), 35.17 (*C-14*$_{exo}$), 34.4 (*C-6*$_{exo}$), 33.0 (*C-4*$_{endo}$), 29.6 (*C-6*$_{endo}$), 29.4 (*C-7*$_{exo}$), 26.5 (*C-7*$_{endo}$), 25.1 (*C-13*$_{endo}$), 24.9 (*C-13*$_{exo}$).

IR (neat, cm^{-1}): 2981, 1735, 1700, 1452 (fingerprint region excluded).

HRMS (ESI+): calculated for $C_{20}H_{26}O_3Cl$ (M+H+): 349.1565 Found: 349.1561.

Benzyl 1-(4-phenylbutanoyl)bicyclo[2.2.1]heptane-2-carboxylate (2z)

Prepared according to **GP 4** using housane **1a** (43.0 mg, 0.20 mmol, 1.0 eq) and benzyl acrylate (81 mg, 0.50 mmol, 2.5 eq). The crude residue was purified by column chromatography on silica gel (hexane:Et_2O = 3:1) to afford two diastereoisomers of the norbornane **2z** (51.2 mg, 0.14 mmol, 68% combined yield; *exo*:*endo* = 1:1).

MIXTURE OF DIASTEREOISOMERS (2z-*endo* and 2z-*exo*)

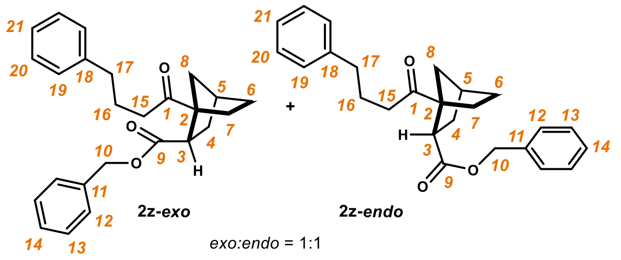

R_f = 0.33. (Hexane:EtOAc = 9:1). Colourless oil.

^{1}H NMR (400 MHz, $CDCl_3$) 7.29 – 7.14 (m, 14H), 7.12 – 7.06 (m, 4H), 7.06 – 7.00 (m, 2H), 5.04 – 4.87 (m, 4H, *2H-10*$_{exo}$ + *2H-10*$_{endo}$), 3.26 (ddd, *J* = 11.7, 4.9, 2.7 Hz, 1H, *H-3*$_{endo}$), 2.74 (ddd, *J* = 7.8, 6.2, 1.5 Hz, 1H, *H-3*$_{exo}$), 2.58 – 2.23 (m, 10H, *2H-15*$_{exo}$ + *2H-15*$_{endo}$ + *2H-17*$_{exo}$ + *2H-17*$_{endo}$ + *H-5*$_{exo}$ + *H-5*$_{endo}$), 1.98 – 1.92 (m, 1H, *H-8a*$_{exo}$), 1.91 – 1.30 (m, 18H, *2H-8*$_{endo}$ + *2H-4*$_{exo}$ + *2H-4*$_{endo}$ + *2H-6*$_{exo}$ + *2H-6*$_{endo}$ + *2H-7*$_{endo}$ + *2H-16*$_{exo}$ + *2H-16*$_{endo}$ + *H-7a*$_{exo}$ + *H-8b*$_{exo}$), 1.30 – 1.22 (m, 1H, *H-7b*$_{exo}$).

^{13}C{H} NMR (101 MHz, $CDCl_3$) δ 212.6 (*C-1*$_{exo}$), 211.5 (*C-1*$_{endo}$), 175.1 (*C-9*$_{exo}$), 173.3 (*C-9*$_{endo}$), 142.1 (*C-18*$_{exo}$), 142.0 (*C-18*$_{endo}$), 136.1 (*C-11*$_{exo}$), 135.8 (*C-11*$_{endo}$), 128.6, 128.6, 128.5, 128.5, 128.4, 128.4, 128.3, 128.2, 125.92 (*C-21*$_{endo}$), 125.90 (*C-21*$_{exo}$), 66.7 (*C-10*$_{endo}$), 66.4 (*C-10*$_{exo}$), 62.8 (*C-2*$_{exo}$), 62.3 (*C-2*$_{endo}$), 48.8 (*C-3*$_{endo}$), 48.4 (*C-3*$_{exo}$), 45.5 (*C-8*$_{endo}$), 39.2 (*C-15*$_{exo}$), 39.0 (*C-8*$_{exo}$), 38.5 (*C-15*$_{endo}$), 38.3 (*C-5*$_{endo}$), 36.3 (*C-5*$_{exo}$), 36.0 (*C-4*$_{exo}$), 35.2 (*C-17*$_{exo}$), 35.1 (*C-17*$_{endo}$), 34.4 (*C-6*$_{exo}$), 33.0 (*C-4*$_{endo}$), 29.6 (*C-6*$_{endo}$), 29.4 (*C-7*$_{exo}$), 26.4 (*C-7*$_{endo}$), 25.0 (*C-16*$_{endo}$), 24.8 (*C-16*$_{exo}$).

NOTE: Only diagnostic signals are assigned.

IR (neat, cm^{-1}): 2952, 1731, 1698, 1454, 1168 (fingerprint region excluded).

HRMS (ESI+): calculated for $C_{25}H_{28}NaO_3$ (M+Na^+): 399.1931 Found: 399.1941.

4-Phenyl-1-(2-(phenylsulfonyl)bicyclo[2.2.1]heptan-1-yl)butan-1-one (2aa)

Prepared according to **GP 4** using housane **1a** (43.0 mg, 0.20 mmol, 1.0 eq) and Phenyl vinyl sulfone (84 mg, 0.50 mmol, 2.5 eq). The crude residue was purified by column chromatography on silica gel (hexane:EtOAc = 4:1) to afford two diastereoisomers of the norbornane **2aa** (54 mg, 0.14 mmol, 71% combined yield; *exo*:*endo* = 1:2). The diastereoisomers were separable. Therefore, each isomer was separated for NMR analysis.

ENDO DIASTEREOISOMER (2aa-*endo*)

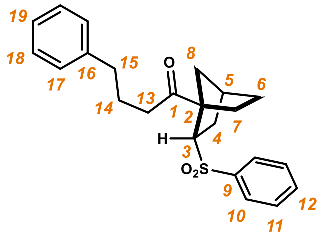

R_f = 0.2. (Hexane:EtOAc = 4:1). Colourless oil.

^{1}H NMR (400 MHz, $CDCl_3$) δ 7.81 – 7.73 (m, 2H, *2H-11*), 7.57 – 7.49 (m, 1H, *H-12*), 7.48 – 7.40 (m, 2H, *2H-10*), 7.26 – 7.19 (m, 2H, *2H-17*), 7.15 – 7.08 (m, 3H, *2H-18* + *H-19*), 4.10 (ddd, *J* = 11.5, 5.3, 2.6 Hz, 1H, *H-3*), 2.64 – 2.42 (m, 5H, *H-7a* + *2H-13* + *2H-15*), 2.42 – 2.37 (m, 1H, *H-5*), 2.04 – 1.88 (m, 2H, *H-4a* + *H-7b*), 1.88 – 1.73 (m, 4H, *H-4b* + *H-6a* + *2H-14*), 1.73 – 1.63 (m, 1H, *H-6b*), 1.57 – 1.45 (m, 2H, *2H-8*).

^{13}C{H} NMR (101 MHz, $CDCl_3$) δ 210.1 (*C-1*), 142.0 (*C-16*), 140.9 (*C-9*), 133.6 (*C-12*), 129.4 (*C-10*), 128.7 (*C-18*), 128.5 (*C-17*), 128.1 (*C-11*), 126.0 (*C-19*), 68.0 (*C-3*), 62.1 (*C-2*), 47.2 (*C-8*), 39.1 (*C-13*), 38.5 (*C-5*), 35.1 (*C-15*), 32.9 (*C-4*), 29.2 (*C-6*), 26.4 (*C-7*), 24.9 (*C-14*).

IR (neat, cm^{-1}): 2955, 1699, 1450, 1309 (fingerprint region excluded).

HRMS (ESI+): calculated for $C_{23}H_{27}O_3S$ (M+H+): 383.1675 Found: 383.1672.

EXO DIASTEREOISOMER (2aa-*exo*)

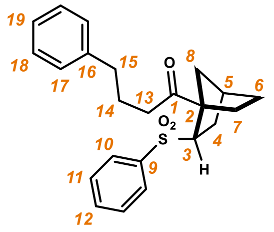

R_f = 0.15. (Hexane:EtOAc = 4:1). Colourless oil.

^{1}H NMR (400 MHz, $CDCl_3$) δ 7.77 – 7.68 (m, 2H, *2H-11*), 7.59 – 7.52 (m, 1H, *H-12*), 7.51 – 7.42 (m, 2H, *2H-10*), 7.25 – 7.19 (m, 2H, *2H-17*), 7.17 – 7.07 (m, 3H, *2H-18* + *H-19*), 3.47 (ddd, *J* = 9.1, 5.4, 1.5 Hz, 1H, *H-3*), 2.75 – 2.52 (m, 4H, *2H-13* + *2H-15*), 2.37 – 2.31 (m, 1H, *H-5*), 2.22 (dtd, *J* =

12.5, 5.0, 2.0 Hz, 1H, *H-6a*), 2.10 (dq, *J* = 10.3, 2.1 Hz, 1H, *H-8a*), 1.96 – 1.85 (m, 2H, *2H-14*), 1.63 – 1.45 (m, 4H, *H-6b* + *H-7a* + *2H-4*), 1.45 – 1.40 (m, 1H, *H-8b*), 1.24 – 1.19 (m, 1H, *H-7b*).

^{13}C{H} NMR (101 MHz, $CDCl_3$) δ 210.3 (*C-1*), 142.2 (*C-16*), 139.8 (*C-9*), 133.7 (*C-12*), 129.2 (*C-10*), 128.7 (*C-18*), 128.6 (*C-11*), 128.4 (*C-17*), 125.9 (*C-19*), 69.9 (*C-3*), 62.5 (*C-2*), 40.6 (*C-13*), 38.1 (*C-8*), 35.6 (*C-5*), 35.2 (*C-15*), 35.0 (*C-4*), 33.6 (*C-6*), 29.1 (*C-7*), 24.8 (*C-14*).

IR (neat, cm^{-1}): 2954, 1699, 1446, 1305 (fingerprint region excluded).

HRMS (ESI+): calculated for $C_{23}H_{27}O_3S$ (M+H+): 383.1675 Found: 383.1671.

Isopropyl 1-(4-phenylbutanoyl)bicyclo[2.2.1]heptane-2-sulfonate (2ab)

Prepared by following the **GP 4** from **1a** (43.0 mg, 0.20 mmol, 1.0 eq), isopropyl ethenesulfonate **S-42** (75 mg, 0.50 mmol, 2.5 eq), SmI_2 (0.3 mL, 0.1 M, 15 mol%). The crude residue was purified by column chromatography on silica gel (hexane:Et_2O = 3:1 to 2:1) to afford two diastereoisomers of the norbornane **2ab** (54.7 mg, 0.15 mmol, 75% combined yield; *exo*:*endo* = 1:1.4). The diastereoisomers were partially separable. Therefore, each isomer was separated for NMR analysis.

ENDO DIASTEREOISOMER (2ab-*endo*)

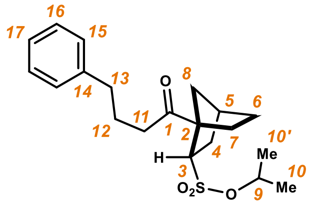

R_f = 0.45. (Hexane:EtOAc = 4:1). Colourless oil.

^{1}H NMR (400 MHz, $CDCl_3$) δ 7.24 – 7.17 (m, 2H, *2H-15*), 7.16 – 7.05 (m, 3H, *2H-16* + *H-17*), 4.84 (hept, *J* = 6.3 Hz, 1H, *H-9*), 4.09 (ddd, *J* = 11.8, 4.9, 2.4 Hz, 1H, *H-3*), 2.64 – 2.45 (m, 4H, *2H-11* + *2H-13*), 2.44 – 2.37 (m, 1H, *H-5*), 2.35 – 2.24 (m, 1H, *H-7a*), 2.05 (tt, *J* = 12.2, 3.6 Hz, 1H, *H-4a*), 1.98 – 1.67 (m, 5H, *2H-12* + *H-7b* + *H-6a* + *H-4b*), 1.61 – 1.50 (m, 3H, *2H-8* + *H-6b*), 1.28 (d, *J* = 6.2 Hz, 6H, *3H-10* + *3H-10'*).

^{13}C{H} NMR (101 MHz, $CDCl_3$) δ 209.9 (*C-1*), 141.9 (*C-14*), 128.6 (*2C-16*), 128.5 (*2C-15*), 126.0 (*C-17*), 76.5 (*C-9*), 64.0 (*C-3*), 61.8 (*C-2*), 46.5 (*C-8*), 39.2 (*C-11*), 38.4 (*C-5*), 35.1 (*C-13*), 33.5 (*C-4*), 29.0 (*C-6*), 26.0 (*C-7*), 24.9 (*C-12*), 23.4 (*C-10*), 23.2 (*C-10'*).

IR (neat, cm^{-1}): 2950, 1704, 1454, 1344, 1169 (fingerprint region excluded).

HRMS (ESI+): calculated for $C_{20}H_{28}NaO_4S$ (M+Na$^+$): 387.1601 Found: 387.1615.

EXO DIASTEREOISOMER (2ab-*exo*)

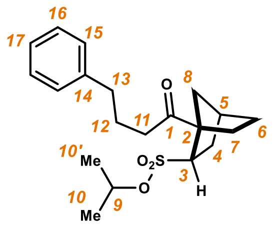

R_f = 0.4. (Hexane:EtOAc = 4:1). Colourless oil.

^{1}H NMR (400 MHz, $CDCl_3$) δ 7.24 – 7.16 (m, 2H, *2H-15*), 7.16 – 7.06 (m, 3H, *2H-16* + *H-17*), 4.82 (hept, *J* = 6.3 Hz, 1H, *H-9*), 3.36 (ddd, *J* = 9.2, 5.5, 1.3 Hz, 1H, *H-3*), 2.69 – 2.50 (m, 4H, *2H-11* + *2H-13*), 2.37 – 2.31 (m, 1H, *H-5*), 2.21 – 2.11 (m, 2H, *H-8a* + *H-6a*), 1.93 – 1.82 (m, 2H, *2H-12*), 1.76 (ddd, *J* = 13.5, 9.0, 2.5 Hz, 1H, *H-6b*), 1.67 – 1.53 (m, 3H, *2H-4* + *H-7a*), 1.42 – 1.37 (m, 1H, *H-8b*), 1.32 – 1.28 (m, 6H, *3H-10* + *3H-10'*).

^{13}C{H} NMR (101 MHz, $CDCl_3$) δ 209.9 (*C-1*), 142.2 (*C-14*), 128.6 (*2C-16*), 128.4 (*2C-15*), 125.9 (*C-17*), 77.0 (*C-9*), 66.2 (*C-3*), 61.9 (*C-2*), 40.6 (*C-11*), 37.5 (*C-8*), 35.4 (*C-5*), 35.2 (*C-13*), 34.6 (*C-4*), 34.4 (*C-6*), 29.0 (*C-7*), 24.8 (*C-12*), 23.4 (*C-10*), 23.2 (*C-10'*).

IR (neat, cm^{-1}): 2954, 1703, 1450, 1350, 1171 (fingerprint region excluded).

HRMS (ESI+): calculated for $C_{20}H_{28}NaO_4S$ (M+Na$^+$): 387.1601 Found: 387.1622.

tert-Butyl 4-(((1-(4-phenylbutanoyl)bicyclo[2.2.1]heptan-2-yl)sulfonyl)oxy)piperidine-1-carboxylate (2ac)

Prepared according to **GP 4** using housane **1a** (43 mg, 0.20 mmol, 1.0 eq) and *tert*-butyl 4-((vinylsulfonyl)oxy)piperidine-1-carboxylate **SI-41** (145.7 mg, 0.50 mmol, 2.5 eq). The crude residue was purified by column chromatography on silica gel (hexane:Et_2O = 4:1 to 2:1 to 3:2) to afford two diastereoisomers of the norbornane **2ac** (92 mg, 0.18 mmol, 91% combined yield; *exo*:*endo* = 1:1.3). The diastereoisomers were partially separable. Therefore, each isomer was separated for NMR analysis.

ENDO DIASTEREOISOMER (2ac-*endo*)

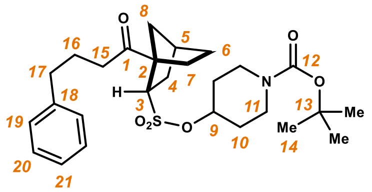

R_f = 0.22. (Hexane:Et_2O = 1:1). Pale-yellow solid.

^{1}H NMR (400 MHz, $CDCl_3$) δ 7.24 – 7.17 (m, 2H, *2H-19*), 7.15 – 7.06 (m, 3H, *2H-20* + *H-21*), 4.77 (tt, *J* = 7.6, 3.7 Hz, 1H, *H-9*), 4.13 (ddd, *J* = 11.8, 4.8, 2.4 Hz, 1H, *H-3*), 3.55 (ddd, *J* = 13.5, 7.2, 3.9 Hz, 2H, *2H-11a*), 3.30 – 3.14 (m, 2H, *2H-11b*), 2.62 – 2.46 (m, 4H, *2H-17* + *2H-15*), 2.45 – 2.39 (m, 1H, *H-5*), 2.32 – 2.22 (m, 1H, *H-7a*), 2.12 – 2.01 (m, 1H, *H-4a*), 1.98 – 1.61 (m, 9H, *2H-16* + *4H-10* + *H-7b* + *H-4b* + *H-6a*), 1.60 – 1.49 (m, 3H, *H-6b* + *2H-8*), 1.38 (s, 9H, *9H-14*).

^{13}C{H} NMR (101 MHz, $CDCl_3$) δ 209.7 (*C-1*), 154.7 (*C-12*), 141.8 (*C-18*), 128.6 (*2C-20*), 128.5 (*2C-19*), 126.0 (*C-21*), 80.0 (*C-13*), 77.4 (*C-9*), 64.0 (*C-3*), 61.8 (*C-2*), 46.5 (*C-8*), 40.5 (2*C-11*), 39.0 (*C-15*), 38.3 (*C-5*), 35.0 (*C-17*), 33.4 (*C-4*), 31.7 (2*C-10*), 29.0 (*C-6*), 28.5 (*3C-14*), 26.0 (*C-7*), 24.8 (*C-16*).

IR (neat, cm^{-1}): 1690, 1421, 1365, 1237, 1165 (fingerprint region excluded).

HRMS (ESI+): calculated for $C_{27}H_{39}NNaO_6S$ (M+Na+): 528.2390 Found: 528.2419.

Melting Point (from hexane:EtOAc): 52 – 55 °C.

EXO DIASTEREOISOMER (2ac-*exo*)

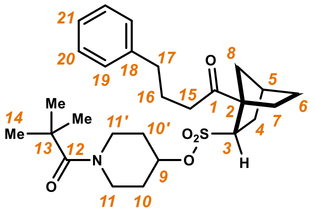

R_f = 0.2. (Hexane:Et_2O = 1:1). White solid.

^{1}H NMR (400 MHz, $CDCl_3$) δ 7.24 – 7.17 (m, 2H, *2H-19*), 7.15 – 7.06 (m, 3H, *2H-20* + *H-21*), 4.74 (tt, *J* = 7.4, 3.7 Hz, 1H, *H-9*), 3.65 – 3.49 (m, 2H, *2H-11a*), 3.45 – 3.35 (m, 1H, *H-3*), 3.25 (ddd, *J* = 13.6, 7.7, 3.9 Hz, 2H, *2H-11b*), 2.67 – 2.49 (m, 4H, *2H-17* + *2H-15*), 2.35 (d, *J* = 4.1 Hz, 1H, *H-5*), 2.20 – 2.10 (m, 2H, *H-8a* + *H-6a*), 1.91 – 1.53 (m, 10H, *2H-16* + *4H-10* + *2H-4* + *H-6b* + *H-7a*), 1.45 – 1.35 (m, 10H, *9H-14* + *H-8b*), 1.31 – 1.24 (m, 1H, *H-7b*).

^{13}C{H} NMR (101 MHz, $CDCl_3$) δ 209.8 (*C-1*), 154.7 (*C-12*), 142.1 (*C-18*), 128.6 (*2C-20*), 128.4 (*2C-19*), 125.9 (*C-21*), 80.0 (*C-13*), 77.8 (*C-9*), 66.4 (*C-3*), 61.9 (*C-2*), 40.6 (*C-11* + *C-11′* + *C-15*), 37.6 (*C-8*), 35.3 (*C-5*), 35.2 (*C-17*), 34.6 (*C-4*), 34.4 (*C-6*), 31.8 (*C-10*), 31.6 (*C-10′*), 28.9 (*C-7*), 28.5 (*3C-14*), 24.8 (*C-16*).

IR (neat, cm^{-1}): 1689, 1421, 1365, 1237, 1163 (fingerprint region excluded).

HRMS (ESI+): calculated for $C_{27}H_{39}NN_aO_6S$ (M+Na+): 528.2390 Found: 528.2411.

Melting Point (from hexane:EtOAc): 105 – 107 °C.

Ethyl 1-nonanoylbicyclo[2.2.1]heptane-2-carboxylate (2ad)

Prepared according to **GP 4** using housane **1b** (41.6 mg, 0.20 mmol, 1.0 eq) and ethyl acrylate (53 µL, 0.50 mmol, 2.5 eq). The crude residue was purified by column chromatography on silica gel (hexane:EtOAc = 99:1 to 39:1 to 24:1) to afford two diastereoisomers of the norbornane **2ad** (45 mg, 0.15 mmol, 73% combined yield; *exo*:*endo* = 1:1). The diastereoisomers were partially separable. Therefore, each isomer was separated for NMR analysis.

ENDO DIASTEREOISOMER (2ad-*endo*)

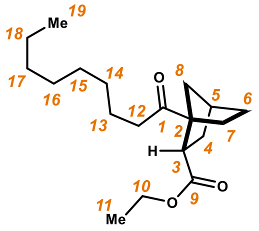

R_f = 0.4. (Hexane:Et_2O = 4:1). Colourless oil.

^{1}H NMR (400 MHz, $CDCl_3$) δ 4.12 – 3.94 (m, 2H, *2H-10*), 3.22 (ddd, *J* = 11.8, 4.9, 2.7 Hz, 1H, *H-3*), 2.60 – 2.48 (m, 1H, *H-12a*), 2.48 – 2.38 (m, 1H, *H-12b*), 2.35 – 2.29 (m, 1H, *H-5*), 1.94 – 1.82 (m, 2H, *H-4a* + *H-7a*), 1.74 (ddd, *J* = 12.5, 4.9, 2.5 Hz, 1H, *H-4b*), 1.69 – 1.58 (m, 2H, *H-8a* + *H-6a*), 1.58 – 1.45 (m, 4H, *H-7b* + *H-8b* + *CH_2*), 1.37 (dddd, *J* = 11.5, 9.0, 4.8, 2.3 Hz, 1H, *H-6b*), 1.30 – 1.16 (m, 10H, *5CH_2*), 1.13 (t, *J* = 7.1 Hz, 3H, *3H-11*), 0.84 – 0.79 (m, 3H, *3H-19*).

^{13}C{H} NMR (101 MHz, $CDCl_3$) δ 212.1 (*C-1*), 173.6 (*C-9*), 62.4 (*C-2*), 60.7 (*C-10*), 48.9 (*C-3*), 45.5 (*C-8*), 39.3 (*C-12*), 38.3 (*C-5*), 33.0 (*C-4*), 32.0 (*CH_2*), 29.7 (*CH_2*), 29.6 (*CH_2*), 29.5 (*CH_2*), 29.3 (*C-6*), 26.3 (*C-7*), 23.8 (*CH_2*), 22.8 (*CH_2*), 14.3 (*C-11*), 14.2 (*C-19*).

IR (neat, cm^{-1}): 2955, 2925, 2871, 2855, 1730, 1701, 1371, 1345, 1190, 1132, 1055 (fingerprint region excluded).

HRMS (ESI+): calculated for $C_{19}H_{32}O_3Na$ (M+Na$^+$): 331.2244 Found: 331.2243.

EXO DIASTEREOISOMER (2ad-*exo*)

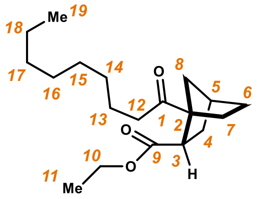

R_f = 0.37. (Hexane:Et_2O = 4:1). Colourless oil.

^{1}H NMR (400 MHz, $CDCl_3$) δ 3.99 (q, *J* = 7.1 Hz, 2H, *2H-10*), 2.67 (ddd, *J* = 8.0, 6.3, 1.5 Hz, 1H, *H-3*), 2.51 – 2.37 (m, 2H, *2H-12*), 2.29 (dq, *J* = 3.8, 1.9 Hz, 1H, *H-5*), 1.97 (dq, *J* = 9.9, 2.1 Hz, 1H, *H-8a*), 1.73 – 1.58 (m, 4H, *H-4a* + *H-6a* + *CH_2*), 1.57 – 1.43 (m, 3H, *H-8b* + *CH_2*), 1.43 – 1.36 (m, 1H, *H-4b*), 1.29 (ddd, *J* = 11.6, 6.0, 2.7 Hz, 1H, *H-6b*), 1.26 – 1.11 (m, 11H, *3H-11* + *4CH_2*), 0.85 – 0.75 (m, 3H, *3H-19*).

^{13}C{H} NMR (101 MHz, $CDCl_3$) δ 213.1 (*C-1*), 175.4 (*C-9*), 62.8 (*C-2*), 60.5 (*C-10*), 48.5 (*C-3*), 39.9 (*C-12*), 39.1 (*C-8*), 36.3 (*C-5*), 36.1 (*CH_2*), 34.4 (*C-4*), 32.0 (*CH_2*), 29.6 (*CH_2*), 29.5 (*CH_2*), 29.4 (*CH_2*), 29.3 (*C-6*), 23.4 (*CH_2*), 22.8 (*C-7*), 14.3 (*C-11*), 14.2 (*C-19*).

IR (neat, cm^{-1}): 2956, 2931, 2869, 1731, 1700, 1380, 1349, 1185, 1130, 1050 (fingerprint region excluded).

HRMS (ESI+): calculated for $C_{19}H_{32}O_3Na$ (M+Na$^+$): 331.2244 Found: 331.2239.

2-Methoxyethyl 1-nonanoylbicyclo[2.2.1]heptane-2-carboxylate (2ae)

Prepared according to **GP 4** using housane **1b** (41.6 mg, 0.20 mmol, 1.0 eq) and 2-methoxyethyl acrylate (64 µL, 0.50 mmol, 2.5 eq). The crude residue was purified by column chromatography on silica gel (Hexane:Et_2O = 19:1 to 9:1 to 4:1) to afford two diastereoisomers of the norbornane **2ae** (63 mg, 0.16 mmol, 81% combined yield; *exo*:*endo* = 1:1). The diastereoisomers were partially separable. Therefore, each isomer was separated for NMR analysis.

ENDO DIASTEREOISOMER (2ae-*endo*)

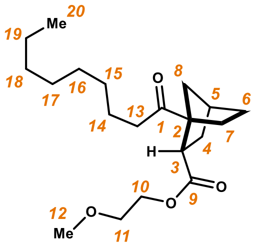

R_f = 0.25. (Hexane:Et_2O = 4:1). Colourless oil.

^{1}H NMR (500 MHz, $CDCl_3$) δ 4.16 (dt, *J* = 11.9, 4.8 Hz, 1H, *H-10a*), 4.08 (dt, *J* = 12.0, 4.7 Hz, 1H, *H-10b*), 3.45 (dd, *J* = 5.3, 4.4 Hz, 2H, *2H-11*), 3.27 (s, 4H, *3H-12* + *H-3*), 2.60 – 2.50 (m, 1H, *H-13a*), 2.50 – 2.41 (m, 1H, *H-13b*), 2.32 (td, *J* = 4.2, 2.0 Hz, 1H, *H-5*), 1.93 – 1.82 (m, 2H, *H-4a* + *H-7a*), 1.74 (ddd, *J* = 12.5, 4.9, 2.6 Hz, 1H, *H-4b*), 1.67 – 1.59 (m, 2H, *H-8a* + *H-6a*), 1.58 – 1.45 (m, 4H, *H-7b*

+ *H-8b* + *CH₂*), 1.37 (dddd, *J* = 11.4, 8.9, 4.8, 2.3 Hz, 1H, *H-6b*), 1.27 – 1.14 (m, 10H, *5CH₂*), 0.81 (t, *J* = 6.8 Hz, 3H, *3H-20*).

^{13}C{H} NMR (126 MHz, $CDCl_3$) δ 212.1 (*C-1*), 173.6 (*C-9*), 70.4 (*C-11*), 63.6 (*C-10*), 62.4 (*C-2*), 58.9 (*C-12*), 48.7 (*C-3*), 45.4 (*C-8*), 39.3 (*C-13*), 38.3 (*C-5*), 33.1 (*C-4*), 32.0 (*CH_2*), 29.7 (*CH_2 + C-6*), 29.5 (*CH_2*), 29.3 (*CH_2*), 26.3 (*C-7*), 23.7 (*CH_2*), 22.8 (*CH_2*), 14.2 (*C-20*).

IR (neat, cm^{-1}): 2953, 2924, 2873, 2854, 1735, 1700, 1455, 1176, 1130, 1055 (fingerprint region excluded).

HRMS (ESI+): calculated for $C_{20}H_{34}O_4Na$ (M+Na$^+$): 361.2349 Found: 361.2345.

EXO DIASTEREOISOMER (2ae-*exo*)

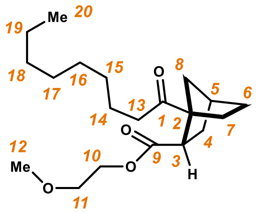

R_f = 0.22. (Hexane:Et_2O = 4:1). Colourless oil.

^{1}H NMR (500 MHz, $CDCl_3$) δ 4.14 – 4.05 (m, 2H, *2H-10*), 3.54 – 3.43 (m, 2H, *2H-11*), 3.30 (s, 3H, *3H-12*), 2.73 (ddd, *J* = 9.0, 5.8, 1.5 Hz, 1H, *H-3*), 2.51 – 2.38 (m, 2H, *2H-13*), 2.32 – 2.27 (m, 1H, *H-5*), 2.01 – 1.94 (m, 1H, *H-8a*), 1.75 – 1.43 (m, 7H, *2H-4* + *H-6a* + *H-7a* + *H-8b* + *CH_2*), 1.44 – 1.36 (m, 1H, *H-6b*), 1.33 – 1.26 (m, 1H, *H-7b*), 1.26 – 1.12 (m, 10H, *5CH_2*), 0.80 (t, *J* = 6.9 Hz, 3H, *3H-20*).

^{13}C{H} NMR (101 MHz, $CDCl_3$) δ 213.1 (*C-1*), 175.4 (*C-9*), 70.6 (*C-11*), 63.5 (*C-10*), 63.0 (*C-2*), 59.1 (*C-12*), 48.2 (*C-3*), 39.9 (*C-13*), 39.3 (*C-8*), 36.3 (*C-5*), 36.2 (*C-4*), 34.4 (*C-6*), 32.0 (*CH_2*), 29.6 (*C-7*), 29.5 (*CH_2*), 29.4 (*CH_2*), 29.3 (*CH_2*), 23.4 (*CH_2*), 22.8 (*CH_2*), 14.2 (*C-20*).

IR (neat, cm^{-1}): 2949, 2926, 2873, 2850, 1734, 1700, 1449, 1176, 1130, 1055 (fingerprint region excluded).

HRMS (ESI+): calculated for $C_{20}H_{34}O_4Na$ (M+Na$^+$): 361.2349 Found: 361.2343.

tert-Butyl 4-(((1-nonanoylbicyclo[2.2.1]heptan-2-yl)sulfonyl)oxy)piperidine-1-carboxylate (2af)

Prepared by following the **GP 4** from **1b** (41.6 mg, 0.20 mmol, 1.0 eq), *tert*-butyl 4-((vinylsulfonyl)oxy)piperidine-1-carboxylate **SI-41** (146 mg, 0.50 mmol, 2.5 eq). The crude residue was purified by column chromatography on silica gel (hexane:EtOAc = 9:1) to afford two diastereoisomers

of the norbornane **2af** (89.9 mg, 0.18 mmol, 90% combined yield; *exo*:*endo* = 1:1.4). The diastereoisomers were partially separable. Therefore, each isomer was separated for NMR analysis.

ENDO DIASTEREOISOMER (2af-*endo*)

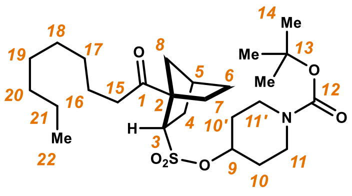

R_f = 0.45. (Hexane:EtOAc = 9:1). Colourless gummy liquid.

^{1}H NMR (500 MHz, $CDCl_3$) δ 4.78 (tt, *J* = 7.6, 3.7 Hz, 1H, *H-9*), 4.14 (ddd, *J* = 11.7, 4.8, 2.4 Hz, 1H, *H-3*), 3.56 (ddd, *J* = 12.0, 7.0, 3.9 Hz, 2H, *H-11a* + *H-11′a*), 3.28 – 3.19 (m, 2H, *H-11b* + *H-11′b*), 2.55 – 2.46 (m, 2H, *2H-15*), 2.46 – 2.40 (m, 1H, *H-5*), 2.35 – 2.25 (m, 1H, *H-7a*), 2.12 – 2.04 (m, 1H, *H-4a*), 1.98 (tdd, *J* = 12.9, 5.4, 2.5 Hz, 1H, *H-7b*), 1.90 – 1.73 (m, 4H, *H-10a* + *H-10′a* + *H-4b* + *H-6a*), 1.73 – 1.47 (m, 7H, *H-10b* + *H-10′b* + *2H-8* + *H-6b* + *CH_2*), 1.38 (s, 9H, *9H-14*), 1.27 – 1.14 (m, 10H, *5CH_2*), 0.81 (t, *J* = 6.8 Hz, 3H, *3H-22*).

^{13}C{H} NMR (126 MHz, $CDCl_3$) δ 210.2 (*C-1*), 154.7 (*C-12*), 80.0 (*C-13*), 77.4 (*C-9*), 64.0 (*C-3*), 61.9 (*C-2*), 46.5 (*C-8*), 41.0 (*C-11*), 40.3 (*C-11′*), 40.0 (*C-15*), 38.3 (*C-5*), 33.5 (*C-4*), 31.93 (*CH_2*), 31.89 (*C-10* + *C-10′*), 29.5 (*CH_2*), 29.3 (*CH_2*), 29.2 (*CH_2*), 29.0 (*C-6*), 28.5 (*3C-14*), 26.1 (*C-7*), 23.4 (*CH_2*), 22.8 (*CH_2*), 14.2 (*C-22*).

IR (neat, cm^{-1}): 2956, 2926, 1699, 1421, 1365, 1350, 1318, 1276, 1237, 1168 (fingerprint region excluded).

HRMS (ESI+): calculated for $C_{26}H_{45}NN_aO_6S$ (M+Na+): 522.2860 Found: 522.2869.

EXO DIASTEREOISOMER (2af-*exo*)

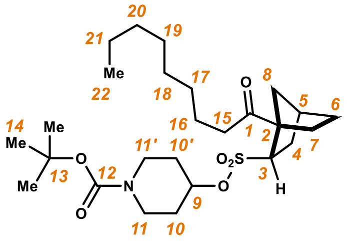

R_f = 0.3. (Hexane:EtOAc = 9:1). Colourless gummy liquid.

^{1}H NMR (500 MHz, $CDCl_3$) δ 4.75 (tt, *J* = 7.4, 3.7 Hz, 1H, *H-9*), 3.57 (ddt, *J* = 11.9, 8.2, 4.0 Hz, 2H, *2H-11a*), 3.45 – 3.37 (m, 1H, *H-3*), 3.31 – 3.19 (m, 2H, *2H-11b*), 2.63 – 2.47 (m, 2H, *2H-15*), 2.40 – 2.32 (m, 1H, *H-5*), 2.21 – 2.12 (m, 2H, *H-8a* + *H-6a*), 1.90 – 1.43 (m, 11H, *4H-10* + *2H-4* + *H-6b* + *H-7a* + *H-8b* + *CH_2*), 1.39 (s, 9H, *9H-14*), 1.34 – 1.26 (m, 1H, *H-7b*), 1.25 – 1.12 (m, 10H, *5CH_2*), 0.81 (t, *J* = 6.9 Hz, 3H, *3H-22*).

^{13}C{H} NMR (126 MHz, $CDCl_3$) δ 210.3 (*C-1*), 154.7 (*C-12*), 80.0 (*C-13*), 77.8 (*C-9*), 66.4 (*C-3*), 62.0 (*C-2*), 41.4 (*C-15*), 40.9 (*C-11*), 40.0 (*C-11′*), 37.7 (*C-8*), 35.4 (*C-5*), 34.6 (*C-4*), 34.5 (*C-6*), 32.0 (*CH_2*), 31.9 (*C-10*), 31.6 (*C-10′*), 29.6 (*CH_2*), 29.33 (*CH_2*), 29.3 (*CH_2*), 29.0 (*C-7*), 28.5 (*3C-14*), 23.3 (*CH_2*), 22.8 (*CH_2*), 14.2 (*C-22*).

IR (neat, cm^{-1}): 2945, 2942, 2854, 1694, 1545, 1419, 1364, 1341, 1320, 1275, 1236, 1165, 1010 (fingerprint region excluded).

HRMS (ESI+): calculated for $C_{26}H_{45}NN_aO_6S$ (M+Na+): 522.2860 Found: 522.2869.

1-Pentanoyl-4-pentylbicyclo[2.2.1]heptane-2-carbonitrile (2ag)

Prepared according to **GP 4** using housane **1s** (44 mg, 0.20 mmol, 1.0 eq) and acrylonitrile (33 µL, 0.50 mmol, 2.5 eq). The crude residue was purified by column chromatography on silica gel (hexane:Et_2O = 9:1 to 6:1 to 4:1) to afford two diastereoisomers of the norbornane **2ag** (50.4 mg, 0.18 mmol, 92% combined yield; *exo*:*endo* = 1:1). The diastereoisomers were separable. Therefore, each isomer was separated for NMR analysis.

ENDO DIASTEREOISOMER (2ag-*endo*)

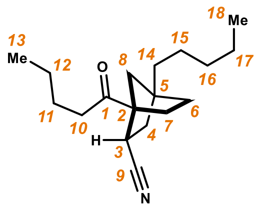

R_f = 0.28. (Hexane:Et_2O = 4:1). Colourless oil.

^{1}H NMR (400 MHz, $CDCl_3$) δ 3.10 (ddd, *J* = 12.2, 4.7, 2.7 Hz, 1H, *H-3*), 2.53 – 2.36 (m, 2H, *2H-10*), 2.11 (dddd, *J* = 13.8, 9.0, 5.0, 2.5 Hz, 1H, *H-7a*), 2.00 (tdd, *J* = 13.0, 5.0, 2.7 Hz, 1H, *H-7b*), 1.89 (td, *J* = 12.5, 3.5 Hz, 1H, *H-4a*), 1.68 – 1.45 (m, 6H, *2H-6* + *H-8a* + *H-4b* + *CH_2*), 1.41 (dd, *J* = 9.1, 6.4 Hz, 2H, *CH_2*), 1.35 (dt, *J* = 9.8, 2.4 Hz, 1H, *H-8b*), 1.31 – 1.11 (m, 8H, *4CH_2*), 0.83 (2 × triplets, 6H, *3H-13* + *3H-18*).

^{13}C{H} NMR (101 MHz, $CDCl_3$) δ 210.4 (*C-1*), 121.7 (*C-9*), 62.5 (*C-2*), 49.4 (*C-5*), 47.2 (*C-8*), 40.2 (*C-4*), 38.6 (*C-10*), 34.7 (*CH_2*), 34.2 (*C-6*), 32.6 (*CH_2*), 32.2 (*C-3*), 29.7 (*C-7*), 25.70 (*CH_2*), 25.66 (*CH_2*), 22.7 (*CH_2*), 22.4 (*CH_2*), 14.1 (*CH_3*), 14.0 (*CH_3*).

IR (neat, cm^{-1}): 2955, 2927, 2238, 1699, 1457, 1188, 1169 (fingerprint region excluded).

HRMS (ESI+): calculated for $C_{18}H_{29}NNaO$ (M+Na^+): 298.2141 Found: 298.2157.

EXO DIASTEREOISOMER (2ag-*exo*)

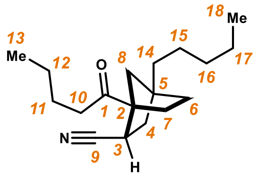

R_f = 0.25. (Hexane:Et_2O = 4:1). Colourless oil.

^{1}H NMR (400 MHz, $CDCl_3$) δ 2.77 (ddd, *J* = 9.2, 4.8, 1.7 Hz, 1H, *H-3*), 2.46 (qdd, *J* = 17.7, 7.9, 6.6 Hz, 2H, *2H-10*), 1.86 – 1.67 (m, 4H, *2H-4* + *H-8a* + *H-6a*), 1.62 (dt, *J* = 10.3, 2.1 Hz, 1H, *H-8b*), 1.58 – 1.43 (m, 6H, *2CH$_2$* + *H-6b* + *H-7a*), 1.39 – 1.32 (m, 1H, *H-7b*), 1.31 – 1.17 (m, 8H, *4CH$_2$*), 0.86 – 0.80 (m, 6H, *3H-13* + *3H-18*).

^{13}C{H} NMR (101 MHz, $CDCl_3$) δ 210.4 (*C-1*), 122.0 (*C-9*), 63.2 (*C-2*), 48.5 (*C-5*), 43.3 (*C-8*), 40.4 (*C-4*), 39.4 (*C-10*), 34.6 (*CH$_2$*), 34.1 (*C-3*), 34.1 (*C-6*), 33.8 (*C-7*), 32.5 (*CH$_2$*), 25.6 (*CH$_2$*), 25.3 (*CH$_2$*), 22.6 (*CH$_2$*), 22.3 (*CH$_2$*), 14.1 (*CH$_3$*), 13.9 (*CH$_3$*).

IR (neat, cm^{-1}): 2955, 2927, 2235, 1702, 1455, 1377, 1190 (fingerprint region excluded).

HRMS (ESI+): calculated for $C_{18}H_{29}NNaO$ (M+Na$^+$): 298.2141 Found: 298.2157.

1-(4-Pentyl-2-(phenylsulfonyl)bicyclo[2.2.1]heptan-1-yl)pentan-1-one (2ah)

Prepared according to **GP 4** using housane **1s** (44 mg, 0.20 mmol, 1.0 eq) and methyl acrylate (45 µL, 0.50 mmol, 2.5 eq). The crude residue was purified by column chromatography on silica gel (hexane:Et_2O = 19:1 to 10:1) to afford two diastereoisomers of the norbornane **2ah** (54 mg, 0.18 mmol, 89% combined yield; *exo*:*endo* = 1:1). The diastereoisomers were partially separable. Therefore, each isomer was separated for NMR analysis.

ENDO DIASTEREOISOMER (2ah-*endo*)

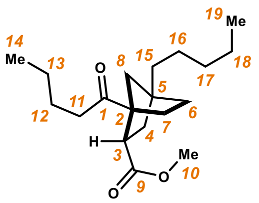

R_f = 0.55. (Hexane:Et_2O = 4:1). Colourless oil.

^{1}H NMR (400 MHz, $CDCl_3$) δ 3.56 (s, 3H, *3H-10*), 3.30 (ddd, *J* = 11.8, 5.0, 2.6 Hz, 1H, *H-3*), 2.56 – 2.35 (m, 2H, *2H-11*), 1.96 (dddd, *J* = 12.8, 11.6, 5.8, 2.7 Hz, 1H, *H-7a*), 1.80 (ddd, *J* = 12.4, 5.0, 2.6 Hz, 1H, *H-4a*), 1.70 (td, *J* = 12.2, 3.1 Hz, 1H, *H-4b*), 1.62 – 1.35 (m, 9H, *H-7b* + *2H-8* + *2H-6* + *2CH$_2$*), 1.30 – 1.14 (m, 8H, *4CH$_2$*), 0.87 – 0.78 (m, 6H, *3H-14* + *3H-19*).

^{13}C{H} NMR (101 MHz, $CDCl_3$) δ 211.9 (*C-1*), 174.1 (*C-9*), 62.3 (*C-2*), 51.8 (*C-10*), 49.7 (*C-8*), 49.6 (*C-5*), 49.2 (*C-3*), 38.8 (*C-11*), 37.4 (*C-4*), 35.5 (*CH_2*), 34.1 (*C-6*), 32.8 (*CH_2*), 27.4 (*C-7*), 25.8 (*CH_2*), 25.7 (*CH_2*), 22.7 (*CH_2*), 22.5 (*CH_2*), 14.2 (*CH_3*), 14.1 (*CH_3*).

IR (neat, cm^{-1}): 2955, 2926, 1738, 1701, 1457, 1435, 1195, 1172 (fingerprint region excluded).

HRMS (ESI+): calculated for $C_{19}H_{32}NaO_3$ (M+Na$^+$): 331.2244 Found: 331.2254.

EXO DIASTEREOISOMER (2ah-*exo*)

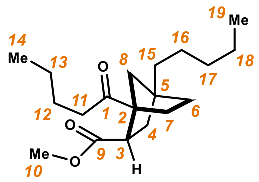

R_f = 0.52. (Hexane:Et_2O = 4:1). Colourless oil.

^{1}H NMR (400 MHz, $CDCl_3$) δ 3.55 (s, 3H, *3H-10*), 2.79 (ddd, *J* = 9.5, 5.3, 1.6 Hz, 1H, *H-3*), 2.49 – 2.35 (m, 2H, *2H-11*), 1.88 (dt, *J* = 9.8, 2.3 Hz, 1H, *H-8a*), 1.76 – 1.66 (m, 2H, *H-4a* + *H-6a*), 1.53 – 1.39 (m, 8H, *H-4b* + *H-6b* + *H-7a* + *H-8b* + *2CH_2*), 1.38 – 1.30 (m, 1H, *H-7b*), 1.28 – 1.15 (m, 8H, *4CH_2*), 0.85 – 0.80 (m, 6H, *3H-14* + *3H-19*).

^{13}C{H} NMR (101 MHz, $CDCl_3$) δ 213.2 (*C-1*), 176.0 (*C-9*), 62.8 (*C-2*), 51.8 (*C-10*), 49.3 (*C-3*), 47.7 (*C-5*), 43.6 (*C-8*), 40.3 (*C-4*), 39.4 (*C-11*), 35.2 (*C-6*), 35.1 (*CH_2*), 33.9 (*C-7*), 32.8 (*CH_2*), 25.8 (*CH_2*), 25.5 (*CH_2*), 22.7 (*CH_2*), 22.5 (*CH_2*), 14.2 (*CH_3*), 14.1 (*CH_3*).

IR (neat, cm^{-1}): 2955, 2927, 1736, 1702, 1455, 1435, 1358, 1198, 1170 (fingerprint region excluded).

HRMS (ESI+): calculated for $C_{19}H_{32}NaO_3$ (M+Na$^+$): 331.2244 Found: 331.2257.

1-(4-Pentyl-2-(phenylsulfonyl)bicyclo[2.2.1]heptan-1-yl)pentan-1-one (2ai)

Prepared according to **GP 4** using housane **1s** (44 mg, 0.20 mmol, 1.0 eq) and phenyl vinyl sulfone (84 mg, 0.50 mmol, 2.5 eq). The crude residue was purified by column chromatography on silica gel (hexane:Et_2O = 1:1) to afford the two diastereoisomers of the norbornane **2ai** (*exo*:*endo* = 1:1.4). The diastereoisomers were separable. Therefore, each isomer was separated for NMR analysis.

ENDO DIASTEREOISOMER (2ai-*endo*)

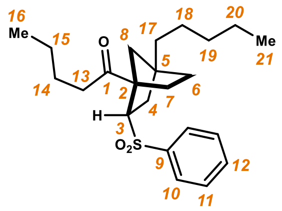

Yield: 39 mg, 0.10 mmol, 48.4%.

R_f = 0.65. (Hexane:Et_2O = 1:1). Colourless oil.

^{1}H NMR (400 MHz, $CDCl_3$) δ 7.82 – 7.74 (m, 2H, *2H-10*), 7.57 – 7.50 (m, 1H, *H-12*), 7.49 – 7.41 (m, 2H, *2H-11*), 4.16 (ddd, *J* = 11.8, 5.1, 2.5 Hz, 1H, *H-3*), 2.67 – 2.56 (m, 1H, *H-8a*), 2.54 – 2.37 (m, 2H, *2H-13*), 2.11 (tdd, *J* = 13.0, 5.5, 2.6 Hz, 1H, *H-8b*), 1.96 (ddd, *J* = 12.4, 5.2, 2.8 Hz, 1H, *H-4a*), 1.75 (dddd, *J* = 11.5, 8.9, 5.3, 2.2 Hz, 1H, *H-6a*), 1.70 – 1.55 (m, 2H, *H-4b* + *H-6b*), 1.50 – 1.40 (m, 3H, *H-8a* + CH_2), 1.37 (dt, *J* = 9.4, 2.4 Hz, 3H, *H-8b* + CH_2), 1.28 – 1.13 (m, 8H, $4CH_2$), 0.86 – 0.77 (m, 6H, *3H-16* + *3H-21*).

^{13}C{H} NMR (101 MHz, $CDCl_3$) δ 210.3 (*C-1*), 140.9 (*C-9*), 133.5 (*C-12*), 129.3 (*C-11*), 128.0 (*C-10*), 68.0 (*C-3*), 61.9 (*C-2*), 51.3 (*C-8*), 50.0 (*C-5*), 39.5 (*C-13*), 36.8 (*C-4*), 35.2 (CH_2), 33.8 (*C-6*), 32.6 (CH_2), 27.3 (*C-7*), 25.6 (CH_2), 25.5 (CH_2), 22.7 (CH_2), 22.3 (CH_2), 14.1 (CH_3), 14.1 (CH_3).

IR (neat, cm^{-1}): 2955, 2927, 1699, 1447, 1306, 1276, 1146, 1086 (fingerprint region excluded).

HRMS (ESI+): calculated for $C_{23}H_{34}NaO_3S$ (M+Na$^+$): 413.2121 Found: 413.2141.

EXO DIASTEREOISOMER (2ai-*exo*)

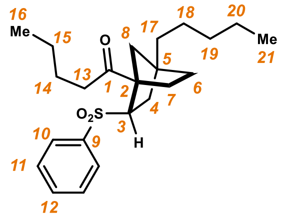

Yield: 27 mg, 0.07 mmol, 34.6%.

R_f = 0.44. (Hexane:Et_2O = 1:1). Colourless crystalline solid.

^{1}H NMR (400 MHz, $CDCl_3$) δ 7.78 – 7.70 (m, 2H, *2H-10*), 7.59 – 7.52 (m, 1H, *H-12*), 7.51 – 7.43 (m, 2H, *2H-11*), 3.58 (ddd, *J* = 9.2, 5.5, 1.5 Hz, 1H, *H-3*), 2.64 (ddd, *J* = 18.1, 8.4, 6.4 Hz, 1H, *H-13a*), 2.51 (ddd, *J* = 18.0, 8.3, 6.4 Hz, 1H, *H-13b*), 2.09 – 1.96 (m, 2H, *H-4a* + *H-8a*), 1.75 – 1.33 (m, 9H, *2H-6* + *H-4b* + *H-8b* + *H-7a* + $2CH_2$), 1.33 – 1.13 (m, 9H, $4CH_2$ + *H-7b*), 0.88 – 0.79 (m, 6H, *3H-16* + *3H-21*).

^{13}C{H} NMR (101 MHz, $CDCl_3$) δ 210.8 (*C-1*), 139.8 (*C-9*), 133.6 (*C-12*), 129.1 (*C-11*), 128.6 (*C-10*), 70.7 (*C-3*), 62.4 (*C-2*), 47.3 (*C-5*), 42.4 (*C-8*), 40.8 (*C-13*), 37.6 (*C-4*), 35.9 (*C-6*), 34.8 (CH_2), 33.5 (*C-7*), 32.7 (CH_2), 25.6 (CH_2), 25.4 (CH_2), 22.7 (CH_2), 22.4 (CH_2), 14.2 (CH_3), 14.1 (CH_3).

IR (neat, cm^{-1}): 2955, 2927, 1703, 1447, 1307, 1147, 1086 (fingerprint region excluded).

HRMS (ESI+): calculated for $C_{23}H_{34}NaO_3S$ (M+Na$^+$): 413.2121 Found: 413.2118.

Melting Point (from hexane:EtOAc): 62 – 65 °C.

tert-Butyl 4-(((1-pentanoyl-4-pentylbicyclo[2.2.1]heptan-2-yl)sulfonyl)oxy)piperidine-1-carboxylate (2aj)

Prepared according to **GP 4** using housane **1s** (44 mg, 0.20 mmol, 1.0 eq) and *tert*-butyl 4-((vinylsulfonyl)oxy)piperidine-1-carboxylate **SI-41** (146 mg, 0.50 mmol, 2.5 eq). The crude residue was purified by column chromatography on silica gel (hexane:Et_2O = 4:1 to 2:1 to 3:2) to afford the two diastereoisomers of the norbornane **2aj** (*exo*:*endo* = 1:1.5). The diastereoisomers were separable. Therefore, each isomer was separated for NMR analysis.

ENDO DIASTEREOISOMER (2aj-*endo*)

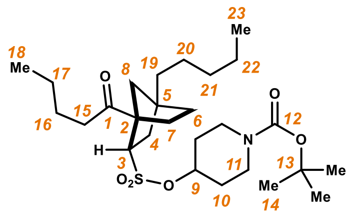

Yield: 56.5 mg, 0.11 mmol, 55%.

R_f = 0.5. (Hexane:Et_2O = 1:1). Colourless oil.

^{1}H NMR (400 MHz, $CDCl_3$) δ 4.78 (tt, *J* = 7.5, 3.7 Hz, 1H, *H-9*), 4.19 (ddd, *J* = 9.6, 7.1, 2.3 Hz, 1H, *H-3*), 3.56 (ddd, *J* = 13.7, 7.2, 3.9 Hz, 2H, *2H-11a*), 3.30 – 3.17 (m, 2H, *2H-11b*), 2.48 (td, *J* = 7.1, 2.2 Hz, 2H, *2H-15*), 2.39 – 2.28 (m, 1H, *H-7a*), 2.13 – 2.01 (m, 1H, *H-7b*), 1.95 – 1.79 (m, 4H, *2H-4* + *2H-10a*), 1.75 – 1.64 (m, 2H, *2H-10b*), 1.63 – 1.53 (m, 2H, *2H-6*), 1.53 – 1.40 (m, 6H, *2H-8* + *2CH₂*), 1.38 (s, 9H, *9H-14*), 1.29 – 1.16 (m, 8H, *4CH₂*), 0.86 – 0.79 (m, 6H, *3H-18* + *3H-23*).

^{13}C{H} NMR (101 MHz, $CDCl_3$) δ 209.9 (*C-1*), 154.7 (*C-12*), 79.9 (*C-13*), 77.3 (*C-9*), 64.2 (*C-3*), 61.7 (*C-2*), 50.6 (*C-8*), 50.0 (*C-5*), 40.5 (*C-11*), 39.4 (*C-15*), 37.4 (*C-4*), 35.1 (*CH_2*), 33.6 (*C-6*), 32.6 (*CH_2*), 31.7 (*C-10*), 28.5 (3*C-14*), 26.9 (*C-7*), 25.6 (*CH_2*), 25.4 (*CH_2*), 22.6 (*CH_2*), 22.3 (*CH_2*), 14.1 (*CH_3*), 14.0 (*CH_3*).

IR (neat, cm^{-1}): 2956, 2928, 1695, 1420, 1365, 1237, 1165 (fingerprint region excluded).

HRMS (ESI+): calculated for $C_{27}H_{47}NNaO_6S$ (M+Na^+): 536.3016 Found: 536.3026.

EXO DIASTEREOISOMER (2aj-*exo*)

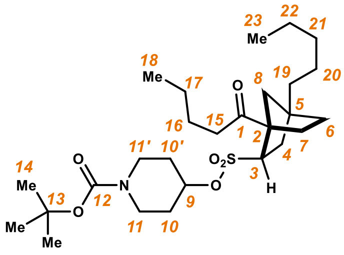

Yield: 39 mg, 0.08 mmol, 38%.

R_f = 0.35. (Hexane:Et_2O = 1:1). Colourless oil.

^{1}H NMR (400 MHz, $CDCl_3$) δ 4.75 (tt, *J* = 7.4, 3.7 Hz, 1H, *H-9*), 3.64 – 3.53 (m, 2H, *2H-11a*), 3.50 (ddd, *J* = 9.3, 5.8, 1.3 Hz, 1H, *H-3*), 3.32 – 3.20 (m, 2H, *2H-11b*), 2.62 – 2.43 (m, 2H, *2H-15*), 2.08 (dt, *J* = 10.4, 2.2 Hz, 1H, *H-8a*), 1.98 (ddd, *J* = 13.4, 5.6, 3.3 Hz, 1H, *H-4a*), 1.90 – 1.79 (m, 3H, *2H-10a* + *H-4b*), 1.79 – 1.62 (m, 4H, *2H-10b* + *2H-6*), 1.57 – 1.41 (m, 5H, *H-7a* + *2CH*$_2$), 1.39 (s, 9H, *9H-14*), 1.36 – 1.17 (m, 10H, *H-7b* + *H-8b* + *4CH*$_2$), 0.86 – 0.80 (m, 6H, *3H-18* + *3H-23*).

^{13}C{H} NMR (101 MHz, $CDCl_3$) δ 210.3 (*C-1*), 154.7 (*C-12*), 80.0 (*C-13*), 77.8 (*C-9*), 67.2 (*C-3*), 61.9 (*C-2*), 47.0 (*C-5*), 42.0 (*C-8*), 40.9 (*C-15*), 40.5 (*C-11* + *C-11'*), 38.5 (*C-4*), 35.6 (*C-6*), 34.7 (*CH*$_2$), 33.4 (*C-7*), 32.6 (*CH*$_2$), 31.8 (*C-10*), 31.6 (*C-10'*), 28.5 (3*C-14*), 25.6 (*CH*$_2$), 25.3 (*CH*$_2$), 22.7 (*CH*$_2$), 22.4 (*CH*$_2$), 14.2 (*CH*$_3$), 14.1 (*CH*$_3$).

IR (neat, cm^{-1}): 2956, 2928, 1696, 1420, 1365, 1329, 1237, 1165 (fingerprint region excluded).

HRMS (ESI+): calculated for $C_{27}H_{47}NNaO_6S$ (M+Na^+): 536.3016 Found: 536.3037.

7. Manipulation of norbornane products of SmI_2 catalysis

7.1 Larger scale SmI_2-catalyzed coupling of alkyl housane ketones and alkenes

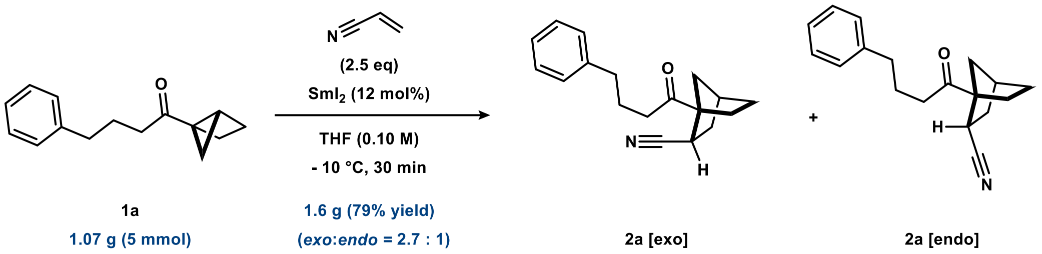

An oven-dried round-bottom flask was charged with a magnetic stir-bar and ketone **1a** (1.07 g, 5.00 mmol, 1 equiv.). The flask was sealed and flushed with N_2. After 10 min, freshly distilled anhydrous THF (44 mL) was introduced under N_2, and an outlet needle was inserted into the septum to replace any trace of air with nitrogen in the flask. After stirring for 5 min, the outlet needle was removed, and acrylonitrile (0.82 mL, 12.5 mmol, 2.5 equiv.) was added by a nitrogen-purged syringe. The flask was placed in a cold bath at –10 °C, and the reaction mixture was stirred at this temperature for 10 min. Then, freshly prepared SmI_2 (6 mL, 12 mol%, 0.1 M) was added dropwise [at this point, the total volume of THF becomes 50 mL, rendering the overall concentration of the reaction mixture 0.1 M]. The reaction mixture was vigorously stirred at –10 °C for 30 min. After completion, the septum was removed and the blue-coloured reaction mixture was quenched with CH_2Cl_2. The resultant mixture was filtered through a silica gel pad (diameter = 3.5 cm and height = 12 cm) using ethyl acetate (30 mL) as the eluent. The solvent was removed under reduced pressure, and the crude residue was purified by silica gel column chromatography (silica gel 100-200 mesh size; hexane/ethyl acetate) to afford the mixture of **2a-*exo*** and **2a-*endo*** (1.60 g, 3.95 mmol, 79% yield, *exo*:*endo* = 2.7:1) as a Colourless oil.

7.2 1-(4-Phenylbutanoyl)bicyclo[2.2.1]heptane-2-carboxylic acid (4)

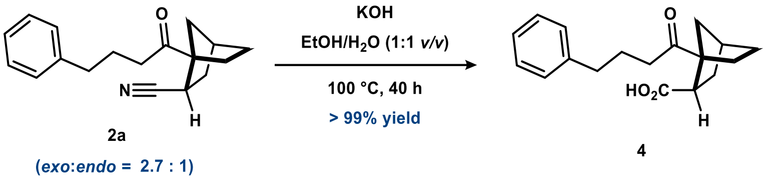

To an oven-dried 100 mL round-bottom flask containing a magnetic stir-bar, nitrile **2a** (*exo*:*endo* = 2.7:1; 1.34 g, 5.00 mmol, 1.0 equiv.) and KOH (2.52 g, 45.0 mmol, 9.0 equiv.) were introduced under air. Ethanol (10.0 mL, 0.5 M) and water (10.0 mL, 0.5 M) were added by syringe, and the flask was placed in a pre-heated oil bath at 100 °C. The reaction mixture was vigorously stirred at the same temperature for 40 h. The reaction mixture was allowed to reach room temperature, and the volume of the reaction mixture was reduced to half by evaporating under reduced pressure. The reaction mixture was diluted with water (10 mL) and EtOAc (20 mL), and the pH of the resultant mixture was adjusted to 1 by dropwise addition of 5 M HCl solution under vigorous stirring. The phases were separated, and the aqueous layer was extracted with EtOAc (3 × 20 mL). The combined organic layers were washed with saturated aqueous NaCl solution (30 mL), dried over anhydrous $MgSO_4$, filtered, and concentrated *in vacuo* to afford **4** (1.43 g, 5.00 mmol, 100% yield) as a single diastereoisomer.

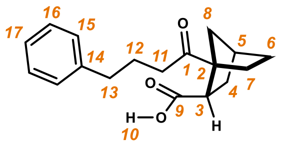

R_f = 0.35. (Hexane:EtOAc = 3:2). Colourless oil.

^{1}H NMR (400 MHz, $CDCl_3$) δ 10.88 (brs, 1H, *H-10*), 7.22 – 7.14 (m, 2H, *2H-15*), 7.14 – 7.03 (m, 3H, *2H-16* + *H-17*), 2.76 – 2.62 (m, 1H, *H-3*), 2.51 (t, *J* = 7.7 Hz, 2H, *2H-13*), 2.44 (t, *J* = 7.0 Hz, 2H, *2H-11*), 2.31 – 2.22 (m, 1H, *H-5*), 1.89 (dd, *J* = 10.0, 2.2 Hz, 1H, *H-8a*), 1.87 – 1.76 (m, 2H, *2H-12*), 1.76 – 1.47 (m, 5H, *2H-4* + *H-7a* + *H-6a* + *H-8b*), 1.41 – 1.32 (m, 1H, *H-6b*), 1.32 – 1.22 (m, 1H, *H-7b*).

^{13}C{H} NMR (101 MHz, $CDCl_3$) δ 212.8 (*C-1*), 181.4 (*C-9*), 142.0 (*C-14*), 128.5 (*2C-16*), 128.4 (*2C-15*), 125.9 (*C-17*), 62.6 (*C-2*), 48.1 (*C-3*), 39.1 (*C-8*), 38.7 (*C-10*), 36.3 (*C-5*), 36.1 (*C-4*), 35.0 (*C-12*), 34.3 (*C-6*), 29.3 (*C-7*), 24.8 (*C-11*).

IR (neat, cm^{-1}): 3026, 2955, 1733, 1701, 1454, 1235, 1129 (fingerprint region excluded).

HRMS (ESI+): calculated for $C_{18}H_{23}O_3$ (M-H$^-$): 285.1496 Found: 285.1511.

7.3 Methyl 1-(4-phenylbutanoyl)bicyclo[2.2.1]heptane-2-carboxylate (2s-*exo*)

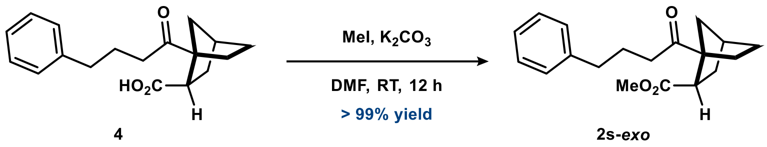

To an oven-dried 100 mL round-bottom flask containing a magnetic stir-bar, acid **4** (1.43 g, 5.00 mmol, 1.0 equiv.) and DMF (25 mL, 0.2 M) were introduced under air. K_2CO_3 (1.38 g, 10.0 mmol, 2.0 eq) and iodomethane (0.5 mL, 7.5 mmol, 1.6 eq) were added sequentially to the solution. The flask was capped with a septum. The reaction was stirred at room temperature for 12 h. The solution was then diluted with H_2O (30 mL) and extracted with Et_2O (3 × 20 mL). The combined organic layers were washed with saturated aqueous NaCl solution (5 × 20 mL), dried over anhydrous $MgSO_4$, filtered, and concentrated *in vacuo*. The crude ester was purified by column chromatography on silica gel (hexane:Et_2O = 2:1) to afford the desired ester **2s-*exo*** (1.49 g, 5.00 mmol, 99.6% yield).

[***NOTE:*** No caution taken for avoiding O_2 and water in this reaction.]

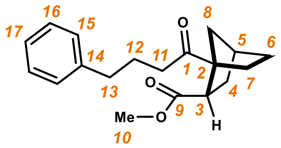

R_f = 0.28. (Hexane:Et_2O = 4:1). Colourless oil.

^{1}H NMR (400 MHz, $CDCl_3$) δ 7.23 – 7.16 (m, 2H, *2H-15*), 7.15 – 7.07 (m, 3H, *2H-16* + *H-17*), 3.53 (s, 3H, *3H-10*), 2.73 – 2.65 (m, 1H, *H-3*), 2.54 (t, *J* = 7.7 Hz, 2H, *2H-13*), 2.47 (t, *J* = 7.1 Hz, 2H, *2H-11*), 2.31 – 2.24 (m, 1H, *H-5*), 1.99 – 1.92 (m, 1H, *H-8a*), 1.90 – 1.76 (m, 2H, *2H-12*), 1.69 – 1.49 (m, 5H, *2H-4* + *H-7a* + *H-6a* + *H-8b*), 1.43 – 1.34 (m, 1H, *H-6b*), 1.32 – 1.24 (m, 1H, *H-7b*)

^{13}C{H} NMR (101 MHz, $CDCl_3$) δ 212.7 (*C-1*), 175.7 (*C-9*), 142.0 (*C-14*), 128.6 (*2C-16*), 128.4 (*2C-15*), 125.9 (*C-17*), 62.8 (*C-2*), 51.7 (*C-10*), 48.3 (*C-3*), 39.1 (*C-8*), 38.9 (*C-11*), 36.3 (*C-5*), 35.9 (*C-4*), 35.1 (*C-13*), 34.2 (*C-6*), 29.4 (*C-7*), 24.9 (*C-11*).

IR (neat, cm^{-1}): 2950, 1731, 1698, 1453, 1434, 1198, 1172, 1130, 1056 (fingerprint region excluded).

HRMS (ESI+): calculated for $C_{19}H_{24}NaO_3$ (M+Na^+): 323.1618 Found: 323.1633.

7.4 4-(3-Phenylpropyl)-6,7,8,8a-tetrahydro-2*H*-4a,7-methanophthalazin-1(5*H*)-one (5)

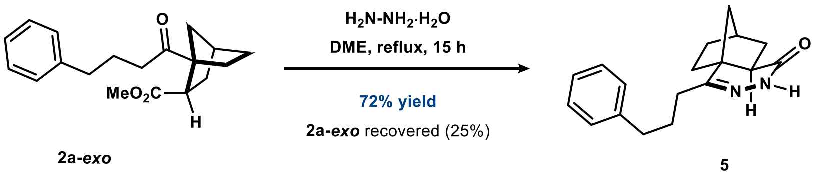

An oven-dried two-necked round-bottom flask equipped with a stirrer bar and reflux condenser was charged with ketone **2a-*exo*** (53.4 mg, 0.20 mmol, 1.0 equiv.), hydrazine hydrate (32 mg, 1.00 mmol, 5 equiv.), and anhydrous DME (1.0 mL, 0.2 M). The reaction mixture was then heated under reflux until the TLC analysis showed complete consumption of keto-ester. After completion, the reaction mixture was cooled to room temperature, and ethanol was evaporated *in vacuo*. The resultant mixture was diluted with water (5 mL) and EtOAc (7 mL), phases were separated, and the aqueous layer was extracted with EtOAc (3 × 7 mL). The combined organic layers were washed with saturated aqueous NaCl solution (10 mL), dried over $MgSO_4$, and concentrated *in vacuo*. The crude product was purified by column chromatography on silica gel (hexane/EtOAc = 4:1 to 2:1 to 3:2) to afford 4,5-dihydropyridazin-3(2*H*)-one derivative **5** (40.7 mg, 0.14 mmol, 72% yield) and unreacted starting material **2a-*exo*** (13.4 mg, 0.05 mmol, 25%).

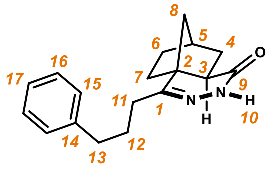

R_f = 0.45. (Hexane:EtOAc = 2:1). White crystalline solid.

^{1}H NMR (400 MHz, $CDCl_3$) δ 8.40 (s, 1H, *H-10*), 7.25 – 7.19 (m, 2H, *2H-15*), 7.17 – 7.07 (m, 3H, *2H-16* + *H-17*), 2.67 – 2.55 (m, 2H, *2H-13*), 2.40 – 2.21 (m, 3H, *2H-11* + *H-5*), 2.18 – 2.10 (m, 1H, *H-3*), 2.08 – 2.00 (m, 1H, *H-4a*), 1.92 – 1.80 (m, 3H, *2H-12* + *H-6a*), 1.74 (ddd, *J* = 12.5, 9.7, 2.4 Hz, 1H, *H-4b*), 1.69 – 1.52 (m, 1H, *H-7a*), 1.47 – 1.41 (m, 1H, *H-8a*), 1.31 – 1.23 (m, 3H, *H-6b* + *H-7b* + *H-8b*).

^{13}C{H} NMR (126 MHz, $CDCl_3$) δ 171.0 (*C-9*), 157.8 (*C-1*), 141.9 (*C-14*), 128.6 (*2C-16*), 128.5 (*2C-15*), 126.1 (*C-17*), 48.8 (*C-2*), 43.1 (*C-5*), 40.1 (*C-8*), 36.6 (*C-3*), 35.5 (*C-13*), 35.0 (*C-4*), 33.2 (*C-11*), 32.1 (*C-6*), 29.2 (*C-7*), 27.9 (*C-12*).

IR (neat, cm^{-1}): 3232, 2952, 2873, 1666, 1454, 1368, 1280 (fingerprint region excluded).

HRMS (APCI+): calculated for $C_{18}H_{23}N_2O$ (M+H+): 283.1805 Found: 283.1803.

Melting Point (from hexane:EtOAc): 97 – 99 °C.

7.5 Methyl 1-(4-phenylbutanamido)bicyclo[2.2.1]heptane-2-carboxylate (6) and methyl 1-((3-phenylpropyl)carbamoyl)bicyclo[2.2.1]heptane-2-carboxylate (7)

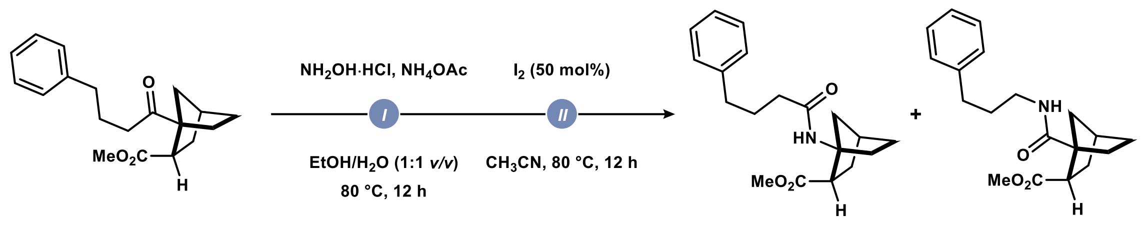

2s-*exo*

6
57% yield

7
19% yield

Prepared by adapting a literature procedure.[8]

Step I: An oven-dried two-necked round-bottom flask equipped with a stirrer bar and reflux condenser was charged with ketone **2s-exo** (60 mg, 0.20 mmol, 1.0 equiv.), hydroxylamine hydrochloride (118 mg, 1.70 mmol, 8.5 equiv.), ethanol (0.8 mL, 0.25 M), and water (1.33 mL, 0.15 M). To the mixture was added NaOAc (131 mg, 1.60 mmol, 8.0 eq), and the reaction was heated under reflux until the TLC analysis showed complete consumption of keto-ester. After completion, the reaction mixture was cooled to room temperature, and 1 M HCl (15 mL) and water (50 mL) were added. The phases were separated, and the aqueous layer was extracted with EtOAc (3 × 10 mL). The combined organic layers were washed with saturated aqueous NaCl solution (10 mL), dried over $MgSO_4$, and concentrated *in vacuo*. The crude oxime was directly used for the next step without further purification.

Step II: An oven-dried two-necked round-bottom flask equipped with a stirrer bar and reflux condenser was charged with crude oxime (from step **I**), iodine (25.0 mg, 1.00 mmol, 0.5 eq), and anhydrous acetonitrile (2 mL). The resultant solution was then vigorously stirred under reflux for 12 h. After completion, the solvent was removed *in vacuo*, and the mixture was treated with saturated aqueous $Na_2S_2O_3$ (10 mL) and EtOAc (10 mL). The phases were separated, and the aqueous layer was extracted with EtOAc (3 × 7 mL), the combined organic layers were washed with saturated aqueous NaCl solution (10 mL), dried over $MgSO_4$, and concentrated *in vacuo*. The crude product was purified by column chromatography on silica gel (hexane/EtOAc = 4:1 to 3:2 to 1:1 to 1:2) to afford **6** (36.0 mg, 0.11 mmol, 57% yield) and **7** (12.0 mg, 0.04 mmol, 19% yield).

Methyl 1-(4-phenylbutanamido)bicyclo[2.2.1]heptane-2-carboxylate (6)

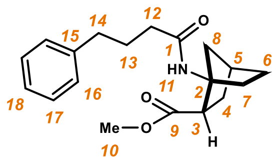

R_f = 0.32. (Hexane:EtOAc = 3:2). Brown solid.

^{1}H NMR (400 MHz, $CDCl_3$) δ 7.26 – 7.16 (m, 2H, *2H-16*), 7.16 – 7.03 (m, 3H, *2H-17* + *H-18*), 5.94 (s, 1H, *H-11*), 3.52 (s, 3H, *3H-10*), 3.00 (ddd, *J* = 9.2, 5.3, 1.3 Hz, 1H, *H-3*), 2.55 (t, *J* = 7.5 Hz, 2H, *2H-14*), 2.18 – 2.11 (m, 1H, *H-5*), 2.11 – 1.99 (m, 3H, *2H-12* + *H-6a*), 1.97 – 1.80 (m, 4H, *2H-13* + *H-4a* + *H-8a*), 1.77 – 1.59 (m, 3H, *H-7a* + *H-6b* + *H-4b*), 1.57 – 1.49 (m, 1H, *H-8b*), 1.41 – 1.30 (m, 1H, *H-7b*).

^{13}C{H} NMR (101 MHz, $CDCl_3$) δ 175.5 (*C-9*), 173.0 (*C-1*), 141.7 (*C-15*), 128.6 (*2C-17*), 128.5 (*2C-16*), 126.0 (*C-18*), 65.3 (*C-2*), 51.7 (*C-10*), 47.7 (*C-3*), 40.5 (*C-8*), 36.1 (*C-12*), 35.4 (*C-4*), 35.2 (*C-14*), 34.3 (*C-6*), 33.7 (*C-5*), 30.1 (*C-7*), 27.2 (*C-13*).

IR (neat, cm^{-1}): 3293, 2945, 1729, 1648, 1539, 1454, 1434, 1363, 1318, 1198, 1169 (fingerprint region excluded).

HRMS (ESI+): calculated for $C_{19}H_{25}NNaO_3$ ($M+Na^+$): 338.1727 Found: 338.1740.

Melting Point (from hexane:EtOAc): 84 – 86 °C.

Methyl 1-((3-phenylpropyl)carbamoyl)bicyclo[2.2.1]heptane-2-carboxylate (7)

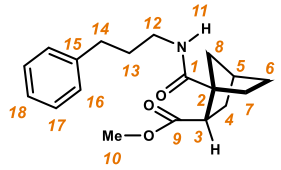

R_f = 0.15. (Hexane:EtOAc = 3:2). Light-yellow gummy liquid.

^{1}H NMR (400 MHz, $CDCl_3$) δ 7.25 – 7.16 (m, 2H, *2H-16*), 7.16 – 7.06 (m, 3H, *2H-17* + *H-18*), 5.51 (s, 1H, *H-11*), 3.54 (s, 3H, *3H-10*), 3.22 (qd, *J* = 7.0, 2.3 Hz, 2H, *2H-12*), 2.68 (ddd, *J* = 9.2, 5.7, 1.5 Hz, 1H, *H-3*), 2.59 (t, *J* = 7.6 Hz, 2H, *2H-14*), 2.32 – 2.25 (m, 1H, *H-5*), 1.95 – 1.88 (m, 1H, *H-8a*), 1.85 – 1.51 (m, 7H, *2H-13* + *2H-4* + *2H-6* + *H-7a*), 1.34 – 1.26 (m, 2H, *H-8b* + *H-7b*).

^{13}C{H} NMR (101 MHz, $CDCl_3$) δ 175.9 (*C-9*), 174.2 (*C-1*), 141.7 (*C-15*), 128.61 (*2C-17*), 128.55 (*2C-16*), 126.1 (*C-18*), 57.5 (*C-2*), 51.8 (*C-10*), 48.7 (*C-3*), 39.2 (*C-12*), 38.6 (*C-8*), 36.5 (*C-5*), 36.0 (*C-4*), 35.0 (*C-6*), 33.5 (*C-14*), 31.3 (*C-13*), 29.3 (*C-7*).

IR (neat, cm^{-1}): 3329, 2950, 2926, 1733, 1638, 1536, 1454, 1435, 1363, 1318, 1199, 1175 (fingerprint region excluded).

HRMS (ESI+): calculated for $C_{19}H_{25}NNaO_3$ ($M+Na^+$): 338.1727 Found: 338.1740.

7.6 2-(4-Methoxyphenyl)-*N*-(1-(4-phenylbutanoyl)bicyclo[2.2.1]heptan-2-yl)acetamide (8)

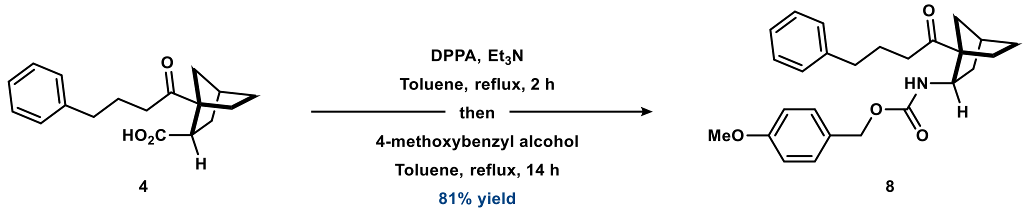

An oven-dried microwave vial equipped with a magnetic stir bar was charged with acid **4** (57 mg, 0.20 mmol, 1.0 equiv.). The vial was then sealed and purged three times with nitrogen. Toluene (2 mL, 0.1 M), Et_3N (42 μL, 0.30 mmol, 1.5 eq), and diphenylphosphoryl azide (DPPA) (65 μL, 0.30 mmol, 1.5 eq) were then sequentially introduced at room temperature. The mixture was heated to reflux for 2 h and then brought to room temperature. 4-Methoxybenzyl alcohol (75 μL, 0.60 mmol, 3.0 eq) was added and the resultant solution was heated under reflux overnight. The reaction mixture was cooled to room temperature, quenched with saturated $NaHCO_3$ solution (3 mL), and diluted with EtOAc (2 mL). The phases were separated, the aqueous layer was extracted with EtOAc (3 × 5 mL), and the combined organic layers were washed with saturated aqueous NaCl solution (7 mL), dried over $MgSO_4$, and concentrated *in vacuo*. The crude product was purified by column chromatography on silica gel (hexane/EtOAc = 6:1 to 4:1) to afford **8** (64.9 mg, 0.16 mmol, 81% yield).

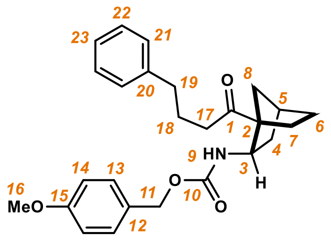

R_f = 0.22. (Hexane:EtOAc = 4:1). Colourless oil.

^{1}H NMR (500 MHz, $CDCl_3$) δ 7.27 – 7.02 (m, 7H, *2H-13* + *2H-21* + *2H-22* + *H-23*), 6.74 (d, *J* = 8.2 Hz, 2H, *2H-14*), 4.87 (s, 2H, *2H-11*), 4.71 (d, *J* = 9.5 Hz, 1H, *H-9*), 4.02 – 3.85 (m, 1H, *H-3*), 3.67 (s, 3H, *3H-16*), 2.68 – 2.30 (m, 4H, *H-17a* + *H-19a* + *H-19b* + *H-17b*), 2.21 – 2.07 (m, 1H, *H-5*), 2.00 – 1.86 (m, 1H, *H-8a*), 1.83 – 1.66 (m, 2H, *2H-18*), 1.62 – 1.43 (m, 4H, *2H-6* + *H-7a* + *H-4a*), 1.36 – 1.12 (m, 3H, *H-4b* + *H-8b* + *H-7b*).

^{13}C{H} NMR (126 MHz, $CDCl_3$) δ 212.3 (*C-1*), 159.6 (*C-10*), 155.6 (*C-15*), 142.2 (*C-20*), 130.0 (*2C-13*), 128.5 (*2C-22* + *C-12*), 128.4 (*2C-21*), 125.9 (*C-23*), 114.0 (*2C-14*), 66.6 (*C-11*), 63.6 (*C-2*), 56.0 (*C-3*), 55.3 (*C-16*), 42.1 (*C-8*), 39.5 (*C-17*), 37.2 (*C-4*), 35.2 (*C-19*), 34.7 (*C-5*), 30.5 (*C-6*), 29.1 (*C-7*), 24.8 (*C-18*).

IR (neat, cm^{-1}): 3332, 2952, 1693, 1514, 1494, 1240, 1175, 1034 (fingerprint region excluded).

HRMS (ESI+): calculated for $C_{26}H_{31}NNaO_4$ (M+Na^+): 444.2145 Found: 444.2147.

7.7 1-(2-(Hydroxymethyl)bicyclo[2.2.1]heptan-1-yl)-4-phenylbutan-1-one (9)

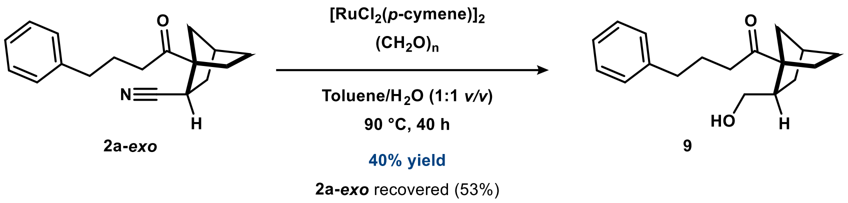

Following the literature procedure,[8] an oven-dried microwave reaction vial containing a magnetic stir-bar was charged with **2a-*exo*** (53.5 mg, 0.20 mmol, 1.0 equiv.), paraformaldehyde (54.0 mg, 1.80 mmol, 9 equiv.), and $[RuCl_2(p\text{-cymene})]_2$ (1.9 mg, 0.003 mmol, 1.5 mol%). The vial was then sealed under air. Toluene (0.5 M, 0.4 mL) and water (0.5 M, 0.4 mL) were added by syringe, and the vial was placed in a pre-heated oil bath at 90 °C. The reaction mixture was vigorously stirred at 90 °C overnight. The reaction mixture was allowed to reach room temperature, and the cap was then slowly opened for a slow gas release. The crude mixture was neutralised using a saturated aqueous Na_2CO_3 solution. The phases were separated, and the aqueous layer was extracted with EtOAc (3 × 5 mL). The combined organic layers were washed with saturated aqueous NaCl solution (10 mL), dried over anhydrous $MgSO_4$, filtered, and concentrated *in vacuo*. The crude alcohol was purified by column chromatography on silica gel (hexane:EtOAc = 6:1 to 4:1 to 3:1) to afford the desired alcohol **9** (21.8 mg, 0.08 mmol, 40% yield) and unreacted starting material (28 mg, 0.11 mmol, 53%).

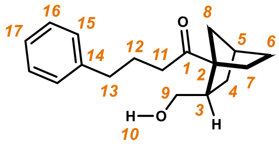

R_f = 0.4. (Hexane:EtOAc = 1:2). Colourless oil.

^{1}H NMR (400 MHz, $CDCl_3$) δ 7.24 – 7.17 (m, 2H, *2H-15*), 7.15 – 7.05 (m, 3H, *2H-16* + *H-17*), 3.46 – 3.27 (m, 1H, *H-9a*), 3.27 – 3.09 (m, 1H, *H-9b*), 2.69 – 2.22 (m, 4H, *2H-13* + *2H-11*), 2.29 – 2.07 (m, 2H, *H-5* + *H-10*), 2.05 – 1.92 (m, 1H, *H-3*), 1.92 – 1.73 (m, 2H, *2H-12*), 1.66 – 1.45 (m, 5H, *2H-6* + *H-4a* + *H-7a* + *H-8a*), 1.34 – 1.22 (m, 2H, *H-7b* + *H-8b*), 1.16 – 1.05 (m, 1H, *H-4b*).

^{13}C{H} NMR (101 MHz, $CDCl_3$) δ 215.8 (*C-1*), 142.0 (*C-14*), 128.6 (*2C-16*), 128.5 (*2C-15*), 126.0 (*C-17*), 65.7 (*C-9*), 62.2 (*C-2*), 48.0 (*C-3*), 40.1 (*C-11*), 37.9 (*C-8*), 35.7 (*C-5*), 35.3 (*C-13*), 35.0 (*C-4*), 34.4 (*C-6*), 29.7 (*C-7*), 25.0 (*C-12*).

IR (neat, cm^{-1}): 2948, 2868, 1694, 1453, 1367, 1063, 1029 (fingerprint region excluded).

HRMS (ESI+): calculated for $C_{18}H_{24}NaO_2$ (M+Na$^+$): 295.1669 Found: 295.1683.

7.8 6-Hydroxyhexa-2,4-diyn-1-yl-1-(4-phenylbutanoyl)bicyclo[2.2.1]heptane-2-carboxylate (10)

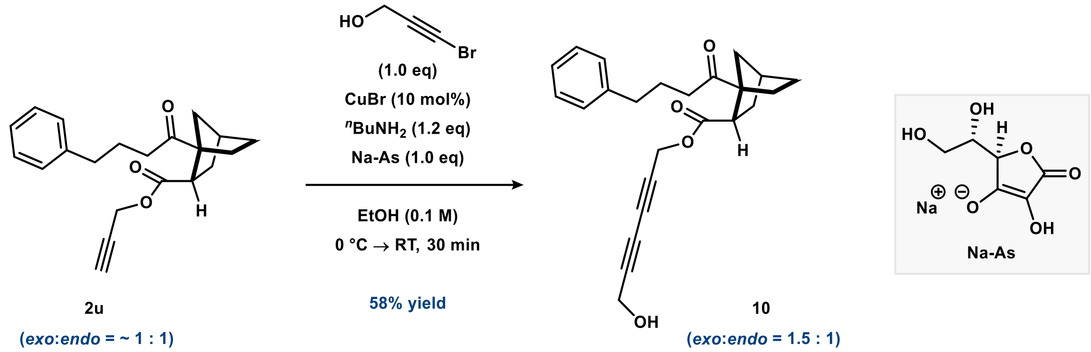

Prepared by adapting a literature procedure.[9]

3-Bromoprop-2-yn-1-ol was prepared from propargyl alcohol according to known procedures.[10]

An oven-dried vial fitted with a stirrer bar was charged with CuBr (1.5 mg, 0.01 mmol, 10 mol%) and sodium ascorbate (20 mg, 0.10 mmol, 1.0 eq) open to the air. A solution of **2u** (39 mg, 0.12 mmol, 1.2 eq) in EtOH (0.25 mL) was added and the solution was cooled (0 °C, 10 min) followed by the addition of ^{n}BuNH$_2$ (21 µL, 0.12 mmol, 1.2 eq). A solution of 3-bromoprop-2-yn-1-ol (13.5 mg, 0.10 mmol, 1.0 eq) in EtOH (0.25 mL) was added, the reaction mixture was removed from the ice bath and allowed to stir (RT, 30 min). After TLC indicated that the reaction was complete, the reaction mixture was concentrated *in vacuo*. The crude product was purified by column chromatography on silica gel (Hexanes:EtOAc = 7:3) to afford **10** as an inseparable mixture of diastereoisomers (22 mg, 0.06 mmol, 58% yield; *exo*:*endo* = 1.5:1).

NOTE: Only diagnostic signals are assigned.

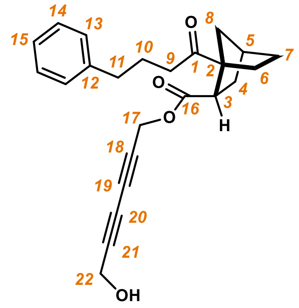

R_f = 0.30, (Hexane: EtOAc = 7:3). Yellow oil.

^{1}H NMR (500 MHz, C_6D_6, mixture of diastereoisomers) δ 7.36 – 6.95 (m, 10H, *2H-13*$_{endo}$ + *2H-13*$_{exo}$ + *2H-14*$_{endo}$ + *2H-14*$_{exo}$ + *H-15*$_{endo}$ + *H-15*$_{exo}$), 4.62 – 4.15 (m, 4H, *2H-17*$_{endo}$ + *2H-17*$_{exo}$), 3.68 – 3.40 (m, 4H, *2H-22*$_{endo}$ + *2H-22*$_{exo}$), 3.09 (ddd, J = 11.9, 4.8, 2.6 Hz, 1H, *H-3*$_{endo}$), 2.69 – 2.51 (m, 4H, *2H-11+H-3*$_{exo}$), 2.43 – 2.22 (m, 4H), 2.09 – 1.92 (m, 5H), 1.84 – 1.62 (m, 3H), 1.62 – 1.49 (m, 2H), 1.42 – 1.17 (m, 5H), 1.16 – 1.00 (m, 2H), 0.96 – 0.80 (m, 2H).

^{13}C{H} NMR (126 MHz, C_6D_6) δ 210.7 (*C-1*$_{endo}$), 209.4 (*C-1*$_{exo}$), 174.0 (*C-16*$_{endo}$), 172.2 (*C-16*$_{exo}$), 142.4 (*C-12*$_{endo}$), 142.3 (*C-12*$_{exo}$), 128.7, 128.6, 128.5, 128.2, 126.03, 125.99, 79.1 (*C-21*$_{exo}$), 78.9 (*C-21*$_{endo}$), 73.8 (*C-18*$_{endo}$), 73.6 (*C-18*$_{exo}$), 71.0 (*C-19*$_{exo}$), 70.9 (*C-19*$_{endo}$), 69.5 (*C-22*$_{endo}$), 69.3 (*C-20b*), 62.7 (*C-2*$_{endo}$), 62.2 (*C-2*$_{exo}$), 52.0 (*C-17*$_{exo}$), 51.9 (*C-17*$_{endo}$), 50.78 (*C-22*$_{exo}$), 50.77 (*C-22*$_{endo}$), 48.3 (*C-3*$_{endo}$), 47.9 (*C-3*$_{exo}$), 44.9, 38.9, 38.6, 38.3, 38.1 (*C-5*$_{endo}$), 36.3 (*C-5*$_{exo}$), 35.6, 35.3, 35.2, 33.9, 33.0, 29.5, 29.1, 26.4, 25.3, 25.0.

IR (neat, cm^{-1}): 3436 (broad), 2953, 2872, 1737, 1693, 1495, 1453 (fingerprint region excluded).

HRMS (ESI+): calculated for $C_{24}H_{26}O_4Na$ (M+Na+): 401.1723 Found: 401.1738.

7.9 3-(3-Phenylpropylidene)hexahydro-3a,6-methanoisoindol-1(4*H*)-one (11)

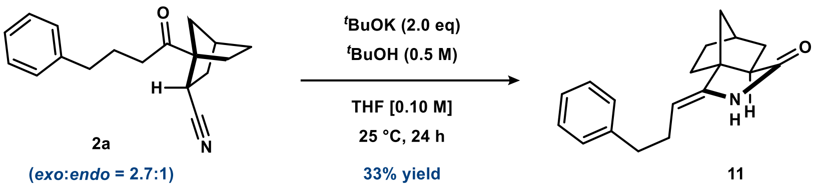

An oven-dried two-neck flask vial fitted with a stirrer bar was charged with **2a** (191 mg, 0.71 mmol, 1.0 eq) and THF (7 mL) followed by dropwise addition of ^{t}BuOK in ^{t}BuOH (3.2 mL, 3.2 mmol, 0.1M) under N_2 and the resultant mixture was stirred at room temperature for 24 h. After TLC analysis indicated the reaction was complete, the reaction was quenched with H_2O (10 mL) and saturated aqueous NaCl (10 mL), and the phases were separated. The aqueous phase was extracted with EtOAc (3 x 25 mL). The combined organic layers were washed with saturated aqueous NaCl (50 mL), dried over anhydrous $MgSO_4$, and concentrated *in vacuo*. The crude product mixture was purified by column chromatography on silica gel (R_f = 0.15, petroleum ether: EtOAc = 4:1) to afford **11** (62 mg, 0.58 mmol, 33% yield).

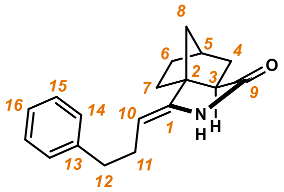

R_f = 0.15, (Petroleum ether: EtOAc = 4:1). Pale-yellow needles.

^{1}H NMR (500 MHz, CD_3CN) δ 7.57 (s, 1H, N-H), 7.31 – 7.14 (m, 5H, *2H-14* + *2H-15* + *H-16*), 4.53 (t, *J* = 7.5 Hz, 1H, H-10), 2.70 – 2.60 (m, 2H, *2H-12*), 2.37 – 2.23 (m, 4H, *H-3* + *H-5* + *2H-11*), 1.86 – 1.66 (m, 3H, *H-4a* + *2H-7*), 1.57 (ddd, *J* = 12.0, 9.5, 2.2 Hz, 1H *H-4b*), 1.43 – 1.24 (m, 4H, *2H-6* + *2H-8*).

^{13}C{H} NMR (126 MHz, CD_3CN) δ 178.5 (*C-9*), 143.1 (*C-13*), 139.7 (*C-1*), 129.6 (*C-14*), 129.2 (*C-15*), 126.8 (*C-16*), 99.8 (*C-10*), 55.8 (*C-2*), 50.4 (*C-3*), 45.2 (*C-8*), 39.3 (*C-5*), 36.7 (*C-12*), 34.0 (*C-4*), 29.6 (*C-7*), 29.1 (*C-11*), 28.9 (*C-6*).

IR (neat, cm^{-1}): 3194 (broad), 3061, 3025, 2952, 2869, 1713, 1687, 1454 (fingerprint region excluded).

HRMS (APCI+): calculated for $C_{18}H_{22}NO$ (M+H+): 268.1696 Found: 268.1697.

Melting Point (from petroleum ether: EtOAc)**:** 113 – 115 °C.

7.10 (1-Tosyl-1*H*-1,2,3-triazol-4-yl)methyl-1-(4-phenylbutanoyl)bicyclo[2.2.1]heptane-2-carboxylate (12)

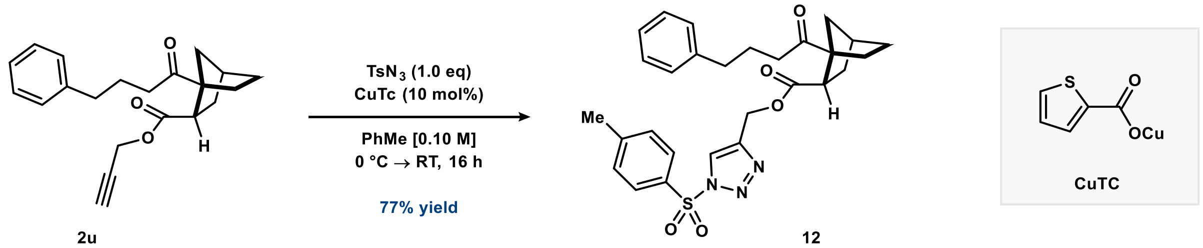

2u (*exo*:*endo* = ~ 1 : 1) **12** (*exo*:*endo* = 1:1.4)

Prepared by adapting a literature procedure.[11]

An oven-dried microwave vial fitted with a stirrer bar was charged with **2u** (32.0 mg, 0.10 mmol, 1.0 eq) and CuTc (2 mg, 0.01 mmol, 10 mol%) and sealed. PhMe (0.8 mL) was then added and the solution was cooled (0 °C, 10 min), followed by the addition of TsN_3 (0.2 mL, 11-15% w/w in PhMe, 0.10 mmol, 1.0 eq) under N_2. The resulting mixture was stirred with gradual warming (0 °C→RT, 16 h). After TLC indicated the reaction was complete, the mixture was transferred into a 25 mL flask and dry loaded onto silica gel under reduced pressure. The crude product mixture was purified by column chromatography on silica gel (hexanes:EtOAc = 7:3) to afford **12** as an inseparable mixture of diastereoisomers (40.3 mg, 0.077 mmol, 77% yield; *exo*:*endo* = 1:1.4).

NOTE: Only diagnostic signals are assigned.

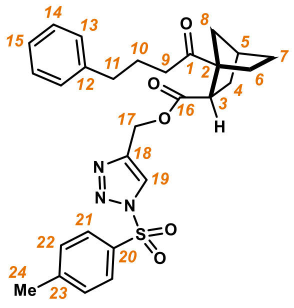

Rf = 0.30 (Hexanes:EtOAc = 7:3). Pale-yellow oil.

^{1}H NMR (400 MHz, C_6D_6, mixture of diastereoisomers) δ 7.99 (s, 1H, *H*-19_{exo}), 7.92 (s, 1H, *H*-19_{endo}), 7.85 – 7.75 (m, 5H, both diastereoisomers), 7.31 – 7.13 (m, 9H, both diastereoisomers + overlapping residual C_6H_6), 7.10 – 7.01 (m, 3H, both diastereoisomers), 6.55 – 6.48 (m, 5H), 5.03 – 4.88 (m, 2H, *2H*-17_{endo}), 4.83 – 4.75 (m, 2H, *2H*-17_{exo}), 2.99 (ddd, *J* = 11.8, 4.8, 2.6 Hz, 1H, *H*-3_{endo}), 2.57 (ddd, *J* = 9.5, 5.6, 1.5 Hz, 1H, *H*-3_{exo}), 2.55 – 2.45 (m, 5H), 2.36 – 2.05 (m, 5H), 2.01 – 1.79 (m, 6H), 1.80 – 1.73 (m, 2H), 1.77 – 1.57 (m, 8H, *3H-24 both diastereoisomers*), 1.60 – 1.41 (m, 4H), 1.39 – 1.15 (m, 9H), 1.12 – 0.99 (m, 3H).

^{13}C{H} NMR (126 MHz, C_6D_6) δ 211.4 (*C-1*$_{endo}$), 210.0 (*C-1*$_{exo}$), 174.9 (*C-16*$_{exo}$), 172.7 (*C-16*$_{endo}$), 146.7 (*C-23*$_{endo}$), 146.6 (*C-23*$_{exo}$), 143.2, 143.1, 142.4, 142.3, 133.8, 133.7, 130.30, 130.27, 128.91, 128.87, 128.83, 128.81, 128.75, 128.73, 126.24, 126.22, 123.8 (*C-19*$_{exo}$), 123.5 (*C-19*$_{endo}$), 63.2 (*C-2*$_{exo}$), 62.4 (*C-2*$_{endo}$), 57.3 (*C-17*$_{endo}$), 57.12 (*C-17*$_{exo}$), 48.4 (*C-3*$_{endo}$), 48.0 (*C-3*$_{exo}$), 44.9, 39.2, 38.6, 38.4, 38.0, 36.5, 35.6, 35.3, 35.3, 34.2, 33.0, 29.6, 29.2, 26.4, 25.3, 25.1, 21.2 (*C-24*$_{exo}$ + *C-24*$_{endo}$).

IR (neat, cm^{-1}): 3367 (broad), 3150, 3026, 2966, 2874, 1737, 1695, 1596, 1495, 1454 (fingerprint region excluded).

HRMS (ESI+): calculated for $C_{28}H_{31}N_3O_5SNa$ (M+Na+): 544.1877 Found: 544.1881.

8. X-ray diffraction analysis data

8.1 Data for 2j [CCDC 2454073]

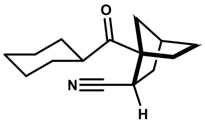

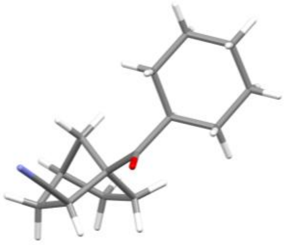

Bond precision:	C-C = 0.0051 A		Wavelength = 1.54184
Cell	a = 9.1680 (5)<br>alpha = 90	b = 11.6251 (7)<br>beta = 90	c = 11.9796 (4)<br>gamma = 90
Temperature:	100 K		

	**Calculated**	**Reported**
Volume	1276.77 (11)	1276.77 (11)
Space group	P 21 21 21	P 21 21 21
Hall group	P 2ac 2ab	P 2ac 2ab
Moiety formula	C15 H21 N O	C15 H21 N O
Sum formula	C15 H21 N O	C15 H21 N O
Mr	231.33	231.33
Dx,g cm^{-3}	1.204	1.203
Z	4	4
Mu (mm^{-1})	0.576	0.576
F000	504.0	504.0
F000′	505.33	
h,k,lmax	11,14,15	11,14,14
Nref	2715 [1571]	2647
Tmin,Tmax	0.923,0.967	0.871,1.000
Tmin′	0.896	

Correction method = #

Reported T Limits: Tmin = 0.871 Tmax = 1.000

AbsCorr = MULTI-SCAN

Data completeness = 1.68/0.97

Theta(max) = 77.625

R(reflections) = 0.0571 (2256)

wR2 (reflections) = 0.0836(2959)

S = 1.058

Npar = 175

8.2 Data for 2o [CCDC 2454076]

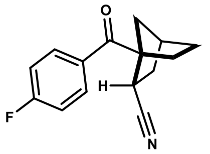

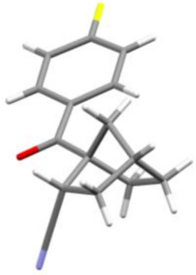

Bond precision:	C-C = 0.0018 A		Wavelength = 1.54184
Cell	a = 10.38066 (18)<br>alpha = 90	b = 8.63180 (17)<br>beta = 99.6602(18)	c = 13.5108 (2)<br>gamma = 90
Temperature:	100 K		

	Calculated	Reported
Volume	1193.45 (4)	1193.45 (4)
Space group	P 21/n	P 1 21/n 1
Hall group	-P 2yn	-P 2yn
Moiety formula	C15 H14 F N O	C15 H14 F N O
Sum formula	C15 H14 F N O	C15 H14 F N O
Mr	243.27	243.27
Dx,g cm^{-3}	1.354	1.354
Z	4	4
Mu (mm^{-1})	0.782	0.782
F000	512.0	512.0
F000′	513.62	
h,k,lmax	12,10,16	12,10,16
Nref	2103	2080
Tmin,Tmax	0.964,0.975	0.963,1.000
Tmin′	0.935	

Correction method = #

Reported T Limits: Tmin = 0.963 Tmax = 1.000

AbsCorr = GAUSSIAN

Data completeness = 0.989

Theta(max) = 66.585

R(reflections) = 0.0327 (1825)

wR2 (reflections) = 0.0864 (2080)

S = 1.057

Npar = 163

8.3 Data for 2ai [CCDC 2455063]

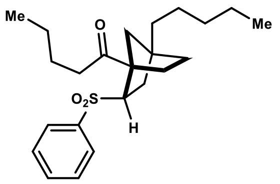

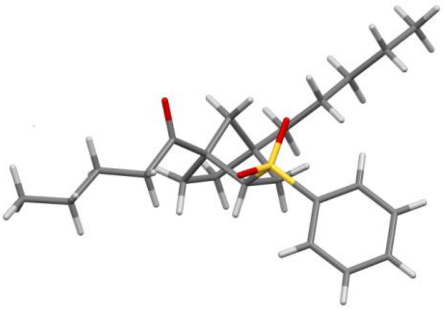

Bond precision:	C-C = 0.0101 A		Wavelength = 1.54184
Cell	a = 11.2672 (4)<br>alpha = 93.703 (3)	b = 14.1337 (8)<br>beta = 90.749 (3)	c = 41.3857 (11)<br>gamma = 93.856 (4)
Temperature:	100 K		

	**Calculated**	**Reported**
Volume	6560.9 (5)	6560.9 (5)
Space group	P -1	P -1
Hall group	-P 1	-P 1
Moiety formula	C23 H34 O3 S	C23 H34 O3 S
Sum formula	C23 H34 O3 S	C23 H34 O3 S
Mr	390.56	390.56
Dx,g cm^{-3}	1.186	1.186
Z	12	12
Mu (mm^{-1})	1.458	1.458
F000	2544.0	2544.0
F000′	2554.52	
h,k,lmax	12,15,45	12,15,45
Nref	18257	17942
Tmin,Tmax	0.886,0.957	0.667,1.000
Tmin′	0.489	

Correction method = #

Reported T Limits: Tmin = 0.667 Tmax = 1.000

AbsCorr = GAUSSIAN

Data completeness = 0.983

Theta(max) = 57.904

R(reflections) = 0.0987 (12275)

wR2 (reflections) = 0.2987 (17942)

S = 1.040

Npar = 1471

8.4 Data for 5 [CCDC 2454079]

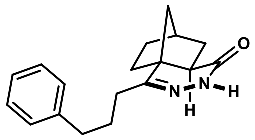

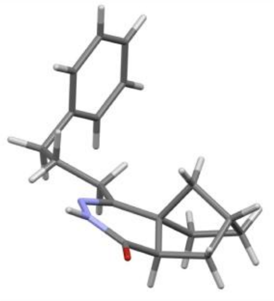

Bond precision:	C-C = 0.0000 A		Wavelength = 1.54184
Cell	a = 6.4034(6)<br>alpha = 90	b = 19.585 (3)<br>beta = 100.015 (10)	c = 12.1599 (12)<br>gamma = 90
Temperature:	100 K		

	**Calculated**	**Reported**
Volume	1501.7 (3)	1501.7 (3)
Space group	P 21/n	P 1 21/n 1
Hall group	-P 2yn	-P 2yn
Moiety formula	C18 H22 N2 O	1 (C18 H22 N2 O)
Sum formula	C18 H22 N2 O	C18 H22 N2 O
Mr	282.38	282.38
Dx,g cm^{-3}	1.249	1.249
Z	4	4
Mu (mm^{-1})	0.608	0.608
F000	608.0	608.0
F000′	609.64	
h,k,lmax	8,25,15	8,24,15
Nref	3359	3147
Tmin,Tmax	0.948,0.979	0.684,1.000
Tmin′	0.875	

Correction method = #

Reported T Limits: Tmin = 0.684 Tmax = 1.000

AbsCorr = MULTI-SCAN

Data completeness = 0.937

Theta(max) = 82.902

R(reflections) = 0.0933 (2413)

wR2 (reflections) = 0.2957 (3147)

S = 1.099

Npar = 418

8.5 Data for 11 [CCDC 2454177]

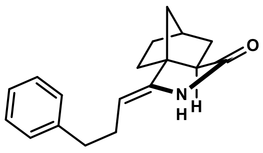

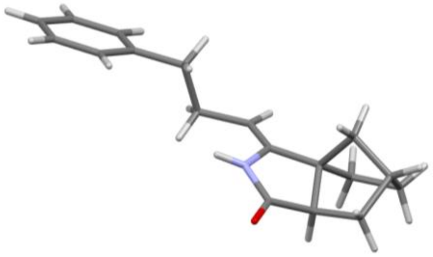

Bond precision:	C-C = 0.0021 A		Wavelength = 1.54184
Cell	a = 6.3882 (2)<br>alpha = 90	b = 18.6389 (5)<br>beta = 90.214 (3)	c = 12.2686(4)<br>gamma = 90
Temperature:	100 K		

	**Calculated**	**Reported**
Volume	1460.80 (8)	1460.80 (8)
Space group	P 21/n	P 1 21/n 1
Hall group	-P 2yn	-P 2yn
Moiety formula	C18 H21 N O	C18 H21 N O
Sum formula	C18 H21 N O	C18 H21 N O
Mr	267.36	267.36
Dx,g cm^{-3}	1.216	1.216
Z	4	4
Mu (mm^{-1})	0.577	0.577
F000	576.0	576.0
F000′	577.54	
h,k,lmax	8,23,15	7,23,15
Nref	3029	2943
Tmin,Tmax	0.953,0.971	0.707,1.000
Tmin′	0.811	

Correction method = #

Reported T Limits: Tmin = 0.707 Tmax = 1.000

AbsCorr = MULTI-SCAN

Data completeness = 0.972

Theta(max) = 75.574

R(reflections) = 0.0489 (2387)

wR2 (reflections) 0.1398 (2943)

S = 1.057

Npar = 255

9. Computational Studies

9.1 Computational Details

Geometry optimisations were performed using Gaussian 16, Revision C.01.,[12] employing the PBE0 functional[13] and Dunning's correlation-consistent double-zeta + polarization basis sets (cc-pVDZ)[14] for C, H, N and O. For Sm and I, Stuttgart-Köln effective core potentials (ECPs) and associated valence basis sets were used.[15] The dispersion corrections from Grimme's D3 model[16] with Becke-Johnson damping factors[17] were incorporated in geometry optimisations. Harmonic vibrational frequency calculations were performed at 263.15 K using the same level of theory to confirm the nature of stationary points as either true minima or transition states and to provide thermodynamic corrections.

Subsequent single-point calculations were carried out on above optimised geometries, integrating the Douglas-Kroll-Hess 2nd order scalar relativistic Hamiltonian,[18] and utilizing the SARC basis set for Sm, Jorge basis set for I[19] and cc-pVTZ[14] for the remaining elements. These calculations incorporated solvent effects using the polarizable continuum model[20] in THF. The enthalpic corrections were derived from frequency calculations, and the entropic contributions were determined from frequency calculations and adjusted using the quasi-harmonic approximation proposed by Grimme.[21] Specifically, $-TS$ terms were corrected using GoodVibes,[22] with a cut-off frequency set to 100 cm^{-1}.

Previous calculations on systems of this type suggest that reactivity takes place via the quintet spin state, with antiparallel coupling of ligand radical anion to Sm(III) $4f^5$ metal center. Hence the calculations have been performed with the spin multiplicity of 7 for the initial reactants and final products and with the spin multiplicity of 5 for all other intermediates and transition states.

9.2 Coordinates and Energies

Cartesian coordinates (in Å) of geometries optimised at PBE0 functional with cc-pVDZ basis sets for C, H, N and O, and ECPs for Sm and I, single point energies E computed in THF (PCM), enthalpic corrections H_{cor}, entropic terms corrected by Grimme's method $-TS_{cor}$ and Gibbs free energies G (in Hartree).

Reactant

E = -26222.9822

$\boldsymbol{H}_{\mathbf{cor}}$ = 0.8102

−*TS* = -0.1073

G = -26222.2793

C	1.30270100	2.62181100	-0.80052100
O	0.33319000	1.88819000	-0.59579600
Sm	-0.93136100	-0.20063200	-0.10427800
O	-2.26955500	1.12727700	-1.94717000
C	-1.60439900	1.72785900	-3.06207500
C	-1.67438100	3.24617800	-2.83309100
C	-2.63971000	3.39660300	-1.64403000
C	-3.30638800	2.03368400	-1.56980100
I	0.74965200	-1.73035400	-2.37544400
I	-2.48955500	1.38846400	2.24545600
O	-1.10858600	-2.36750500	1.32248400
C	-1.13023600	-3.68182300	0.74085600
C	-1.18809200	-4.64191300	1.91696200
C	-1.96868500	-3.82834100	2.94575800
C	-1.40563100	-2.43601200	2.72575900
O	1.10652200	-0.33848800	1.48892700
C	1.95393100	-1.49690400	1.60229400
C	3.13488600	-1.05212300	2.44389100
C	2.48600800	-0.02414100	3.36713800
C	1.51438800	0.66931700	2.42767400
O	-3.05734600	-1.55481500	-0.75365400
C	-3.45347200	-1.85104900	-2.10681900
C	-4.92010600	-2.25517200	-2.02935700
C	-5.39261700	-1.52534700	-0.77448500
C	-4.18233200	-1.67165700	0.12634700
H	-2.02446300	-3.75945000	0.09982600
H	-0.24186500	-3.78987700	0.10193600
H	-0.46941100	-2.28593800	3.29401800
H	-2.09114600	-1.61244800	2.97165200
H	-0.17347400	-4.85606900	2.29066500
H	-1.66229700	-5.59789400	1.65503300
H	-1.83736300	-4.17745900	3.97929800
H	-3.04592200	-3.84658300	2.71307900
H	1.37986300	-2.30023400	2.09391800
H	2.21798600	-1.82551700	0.58740800
H	2.00938100	1.48582900	1.87237100
H	0.61066700	1.06780300	2.91187800
H	3.90247000	-0.58332100	1.80887500
H	3.60703500	-1.88933000	2.97547000
H	3.20191600	0.67570400	3.82055600
H	1.93707800	-0.52573100	4.18072600
H	-3.30204300	-0.93706200	-2.70076600
H	-2.79084700	-2.63563400	-2.50027600
H	-4.16357600	-2.66612900	0.61147600
H	-4.08934500	-0.89552800	0.90090100
H	-5.01769900	-3.34418800	-1.89228100
H	-5.47580700	-1.97886000	-2.93613700
H	-6.30565800	-1.94894200	-0.33378600
H	-5.57401300	-0.45972600	-0.98901400
H	-0.59033900	1.30890000	-3.10043800
H	-2.13440400	1.43566800	-3.98622300
H	-4.14614600	1.94381900	-2.28639100
H	-3.64417400	1.75996900	-0.56050100
H	-2.05078400	3.75515900	-3.73229300
H	-0.68745600	3.67075300	-2.60305400
H	-3.36263500	4.21399600	-1.77293800
H	-2.08673100	3.56617400	-0.70883200
C	2.44952400	2.11645900	-1.63246900
H	3.10225500	2.93145900	-1.97757600

C	3.25031100	1.08048900	-0.84072500
C	4.33248800	0.39843900	-1.67376200
H	3.70998600	1.55438400	0.04430500
H	2.55076200	0.31532300	-0.47317500
H	2.01571100	1.61757300	-2.51404900
C	1.54212800	4.85850200	1.80801300
H	1.67168600	4.25281100	2.71460700
H	1.54333400	5.92294800	2.08974700
C	1.34151300	3.92619200	-0.14739100
C	2.52710300	4.48103600	0.70800400
H	3.49371700	3.99353600	0.85006500
C	0.35955000	4.35419900	0.93889900
H	-0.18003900	3.50481300	1.37869800
H	-0.37473800	5.11052200	0.62381300
C	2.17267700	5.11075800	-0.58439600
H	1.67043500	6.08303400	-0.60263600
H	2.86710500	4.93884800	-1.40745800
H	3.76676200	-2.24963800	-1.47721200
H	5.04626000	1.14934300	-2.05269000
H	3.85604600	-0.07764000	-2.54493800
C	5.05566100	-0.63746400	-0.85785800
C	6.13318200	-0.28298800	-0.03829600
C	6.75247300	-1.22696900	0.77873500
C	6.30072100	-2.54585500	0.78679600
C	5.23230100	-2.91136400	-0.03108000
C	4.61421100	-1.96546800	-0.84674000
H	6.49377800	0.74972000	-0.04334900
H	7.59445700	-0.93251300	1.40910600
H	6.78555700	-3.28824300	1.42418800
H	4.87527100	-3.94366600	-0.03669000

Int I

E = -26222.1385

$\boldsymbol{H}_{\mathrm{cor}}$ = 0.8087

$\boldsymbol{-TS}_{\mathrm{cor}}$ = -0.1027

G = -26222.2546

C	1.13994100	2.57465100	-0.94910400
O	0.28018000	1.62770500	-0.55079500
Sm	-0.83808900	-0.08409400	-0.08775700
O	-2.17707300	0.91220200	-1.95453900
C	-1.51755600	1.58410100	-3.03993900
C	-1.77331900	3.07339700	-2.81111800
C	-2.97894400	3.10277900	-1.84954600
C	-3.39200300	1.64168100	-1.74208400
I	0.69072500	-1.80059600	-2.15261600
I	-2.58412200	1.31475000	2.05198900
O	-1.05781900	-2.21575300	1.34364500
C	-0.96641800	-3.56378600	0.84436400
C	-0.75602100	-4.42842900	2.07234300
C	-1.56199600	-3.67338500	3.12443000
C	-1.26625000	-2.22664300	2.77143200
O	0.99752300	-0.29761300	1.55995100
C	1.97258000	-1.35904700	1.65751900
C	3.18282100	-0.73821600	2.33006100
C	2.54800500	0.33634300	3.20773300
C	1.43688500	0.85616100	2.31673600
O	-2.90593100	-1.48731200	-0.69683700
C	-3.19870900	-1.98526000	-2.02188300
C	-4.45289800	-2.82689200	-1.86790600
C	-5.16200600	-2.12726600	-0.71291400
C	-3.99593700	-1.78836200	0.19404200
H	-1.91048400	-3.81152900	0.32854200
H	-0.15273200	-3.59774200	0.10749700

H	-0.34522200	-1.86188200	3.25559100
H	-2.07613000	-1.52218000	3.00437900
H	0.31095200	-4.45195900	2.34707500
H	-1.09019000	-5.46347200	1.91700900
H	-1.27887300	-3.91558900	4.15794900
H	-2.63709600	-3.88648500	3.00821900
H	1.53578400	-2.16935400	2.26543600
H	2.17026300	-1.73921500	0.64557300
H	1.78867300	1.61791000	1.60617200
H	0.56292000	1.24904500	2.85485200
H	3.84763300	-0.28812300	1.57847100
H	3.76782300	-1.47969800	2.89042100
H	3.24861000	1.13148900	3.49788700
H	2.13180600	-0.10545000	4.12793600
H	-3.35868500	-1.11795200	-2.68055900
H	-2.31661800	-2.53387600	-2.37965500
H	-3.71252100	-2.65095100	0.82266400
H	-4.15185700	-0.91698800	0.84490200
H	-4.19504000	-3.86098000	-1.58692000
H	-5.04348100	-2.86428700	-2.79355300
H	-5.91659400	-2.74939700	-0.21225700
H	-5.65310900	-1.20508200	-1.06338200
H	-0.46223800	1.29045100	-3.01523200
H	-1.97028600	1.22404600	-3.98070400
H	-4.11243800	1.35518600	-2.53128800
H	-3.78755900	1.35891400	-0.75798400
H	-1.98037200	3.58590500	-3.76109700
H	-0.89235900	3.54271400	-2.35171300
H	-3.80447300	3.73220100	-2.20980300
H	-2.67622100	3.47086800	-0.85918900
C	2.28803000	2.09217000	-1.77746700
H	2.83561700	2.93961200	-2.21939900
C	3.23322200	1.19951700	-0.96922200
C	4.31844600	0.50984600	-1.79376800
H	3.70292600	1.78454200	-0.15809900
H	2.62057500	0.42239700	-0.48603800
H	1.89464300	1.48849900	-2.61586000
C	0.78177900	4.83563600	1.72406300
H	0.53958200	4.28289100	2.64282200
H	0.77456800	5.91496500	1.95340100
C	1.14613200	3.82049700	-0.19586900
C	2.02809800	4.32062800	1.02952600
H	2.87609500	3.82426700	1.50862900
C	-0.08547300	4.36931000	0.52808000
H	-0.78153600	3.57039600	0.81357900
H	-0.63597400	5.15153000	-0.02225600
C	2.15541800	4.91572900	-0.33633500
H	1.81848400	5.94382700	-0.52054700
H	3.07150900	4.64639500	-0.86644900
H	3.85499800	-2.14589300	-1.46180800
H	4.99673000	1.26143800	-2.23138000
H	3.84184100	-0.02664800	-2.62971800
C	5.09154800	-0.45906100	-0.94376600
C	6.16349100	-0.02901500	-0.15289000
C	6.81707200	-0.90742800	0.70932700
C	6.40859400	-2.23778800	0.79433300
C	5.34699100	-2.68039300	0.00600700
C	4.69502900	-1.79894900	-0.85412000
H	6.48877600	1.01325100	-0.21479000
H	7.65305800	-0.55202100	1.31598400
H	6.92147900	-2.92853600	1.46688900
H	5.02394400	-3.72276900	0.05813800

TS I

E = -26222.9587

$\boldsymbol{H}_{\mathbf{cor}}$ = 0.6940

$\boldsymbol{-TS}_{\mathbf{cor}}$ = -0.1025

G = -26222.2539

C	1.17125100	2.55112700	-0.94570000
O	0.27269600	1.62596500	-0.58459000
Sm	-0.84722300	-0.07816800	-0.09351900
O	-2.18684500	0.89580400	-1.97591800
C	-1.52093800	1.55639300	-3.06453800
C	-1.75406800	3.05017100	-2.83785000
C	-2.95404500	3.09808000	-1.86982500
C	-3.39079500	1.64402900	-1.76363800
I	0.67402000	-1.82985200	-2.13342500
I	-2.59603700	1.34989300	2.02475600
O	-1.05857100	-2.18145800	1.37599800
C	-0.95724900	-3.53811200	0.90208900
C	-0.74850400	-4.37866100	2.14686300
C	-1.56481500	-3.60951300	3.18054400
C	-1.27707900	-2.16746300	2.80258500
O	0.99317400	-0.24788600	1.55385300
C	1.96096600	-1.31168100	1.68554700
C	3.17388400	-0.67860500	2.34136100
C	2.54339400	0.42241500	3.18903200
C	1.43306200	0.92317400	2.28573200
O	-2.91420800	-1.48924200	-0.67761800
C	-3.21362600	-1.99944300	-1.99666700
C	-4.45949100	-2.84973600	-1.82573200
C	-5.16689000	-2.14312100	-0.67395300
C	-3.99813900	-1.78575900	0.22249300
H	-1.89714700	-3.80029400	0.38577500
H	-0.13962200	-3.58117900	0.17017900
H	-0.36227600	-1.78645100	3.28598900
H	-2.09366000	-1.46486000	3.01667300
H	0.31706900	-4.39060500	2.42764200
H	-1.07559100	-5.41839000	2.00873800
H	-1.28544400	-3.83057300	4.21979900
H	-2.63785600	-3.83201100	3.06299500
H	1.51914300	-2.10090500	2.31707400
H	2.15831000	-1.72369500	0.68600300
H	1.78531500	1.67199400	1.56097100
H	0.55917400	1.32633100	2.81641400
H	3.84161000	-0.25313800	1.57822100
H	3.75446500	-1.40745900	2.92260500
H	3.24719100	1.22268000	3.45648000
H	2.12714600	0.00735000	4.12156600
H	-3.38629400	-1.13852400	-2.66050300
H	-2.32970600	-2.54345300	-2.35688400
H	-3.70604300	-2.63875300	0.86002900
H	-4.15603400	-0.90717600	0.86312100
H	-4.19112300	-3.87849600	-1.53518700
H	-5.05562200	-2.90199600	-2.74709400
H	-5.91370000	-2.76546500	-0.16202200
H	-5.66716900	-1.22858800	-1.03138100
H	-0.47038700	1.24607200	-3.04379700
H	-1.98277500	1.20183300	-4.00297000
H	-4.11468600	1.36929500	-2.55380700
H	-3.79159700	1.36670000	-0.78014500
H	-1.96034700	3.56237400	-3.78820200
H	-0.86592800	3.51108000	-2.38419800
H	-3.77049700	3.74251600	-2.22417000
H	-2.63954900	3.45788300	-0.87996100
C	2.29754100	2.04554100	-1.79344200
H	2.85775000	2.87959900	-2.24388300

C	3.23058300	1.14048800	-0.98858700
C	4.30163200	0.42921600	-1.81263400
H	3.71218100	1.72424400	-0.18379800
H	2.60927600	0.37571800	-0.49723500
H	1.87833700	1.44919700	-2.62387400
C	0.88939300	4.84643100	1.69704900
H	0.70453400	4.30426700	2.63544700
H	0.80055800	5.92878500	1.90793900
C	1.16607700	3.80248200	-0.28796000
C	2.17700300	4.43683300	1.02886700
H	3.03866200	3.97452700	1.51469300
C	0.00360600	4.31748500	0.54922000
H	-0.65438200	3.50324200	0.87736300
H	-0.60780900	5.07614700	0.02804600
C	2.20544400	4.86511200	-0.39369500
H	1.87795500	5.88606400	-0.64966900
H	3.11255600	4.57461100	-0.93083600
H	3.81490100	-2.21433500	-1.41898600
H	4.98206500	1.16807800	-2.26820500
H	3.81368400	-0.11775800	-2.63507100
C	5.07420400	-0.52992200	-0.95082200
C	6.15834100	-0.09371400	-0.18034100
C	6.81080000	-0.95930100	0.69557100
C	6.38862900	-2.28268000	0.81512400
C	5.31491900	-2.73156400	0.04703200
C	4.66430000	-1.86295300	-0.82708500
H	6.49384800	0.94337600	-0.26884500
H	7.65633500	-0.59931200	1.28606000
H	6.90031000	-2.96339400	1.49873300
H	4.98126700	-3.76888800	0.12614700

Int II

E = -26222.9939

H_{cor} = 0.8078

$-TS_{cor}$ = -0.1044

G =

C	1.00328300	2.69752600	-0.47520700
O	0.22364200	1.63217200	-0.26797300
Sm	-0.76587500	-0.20658400	-0.05569200
O	-2.18324300	0.92389500	-1.79432000
C	-1.59501200	1.80701100	-2.76053300
C	-2.03340800	3.20521700	-2.33505400
C	-3.31635300	2.95667100	-1.51362200
C	-3.49828200	1.44304900	-1.56271800
I	0.91496900	-1.53336400	-2.28744500
I	-2.68213300	0.78915400	2.16846800
O	-0.82032500	-2.48347100	1.13508500
C	-0.60039300	-3.75474400	0.49375300
C	-0.35559600	-4.73360200	1.62617300
C	-1.24753200	-4.16567600	2.72565900
C	-1.05624700	-2.67063000	2.54584100
O	1.06610200	-0.43283200	1.60407000
C	2.12268300	-1.41732400	1.60281500
C	3.26838900	-0.78504200	2.37139800
C	2.53449600	0.13119800	3.34644900
C	1.40288300	0.66241300	2.48822000
O	-2.68162300	-1.71439800	-0.88203200
C	-2.91953100	-2.06589900	-2.26448800
C	-4.19888200	-2.88905900	-2.26520900
C	-4.91939100	-2.37479000	-1.02267900
C	-3.76201600	-2.19288700	-0.06293600
H	-1.50393100	-4.01573000	-0.08367400
H	0.23510300	-3.64049300	-0.21003800
H	-0.17563300	-2.29704200	3.09460600

H	-1.92188800	-2.05713200	2.82989300
H	0.70183200	-4.70689100	1.93548300
H	-0.60169300	-5.76695700	1.34557200
H	-0.97473900	-4.50183800	3.73538300
H	-2.29902400	-4.44354000	2.54729300
H	1.74430400	-2.32486300	2.10291000
H	2.36670200	-1.66087300	0.55916300
H	1.71182200	1.52156900	1.87344000
H	0.49172500	0.93008400	3.04093900
H	3.90812600	-0.20291800	1.69198100
H	3.90072700	-1.53770400	2.86106400
H	3.16478800	0.93850000	3.74389600
H	2.13620100	-0.44377600	4.19849000
H	-3.02126900	-1.12770400	-2.82996000
H	-2.03605900	-2.60012300	-2.64068400
H	-3.46865000	-3.15450300	0.39623200
H	-3.92920700	-1.45535700	0.73423700
H	-3.97085700	-3.96133100	-2.15393200
H	-4.77211900	-2.76131900	-3.19371100
H	-5.68361600	-3.06374600	-0.63740300
H	-5.39973600	-1.40382000	-1.22465600
H	-0.51284000	1.63706500	-2.74641400
H	-1.99080500	1.53186500	-3.75466700
H	-4.14656300	1.13284800	-2.40351800
H	-3.87054300	1.00866000	-0.62624000
H	-2.20728400	3.84919600	-3.20837100
H	-1.25546300	3.67082500	-1.71505200
H	-4.19446500	3.47803000	-1.91934800
H	-3.18148000	3.28047500	-0.47215400
C	2.18074900	2.43471900	-1.37326500
H	2.67695000	3.37566500	-1.65517700
C	3.16794800	1.49120500	-0.69441000
C	4.31644600	1.01933800	-1.58276700
H	3.57765100	1.97729700	0.20901700
H	2.61049900	0.60481800	-0.35439600
H	1.82138900	1.95456300	-2.30062200
C	6.19978400	0.43629400	-0.00219600
C	6.92265200	-0.48402000	0.75471800
C	6.62309400	-1.84329200	0.67549600
H	3.89863800	0.55853800	-2.49199000
C	5.60081200	-2.27188400	-0.17042500
C	4.87955000	-1.34874100	-0.92509000
H	6.43882500	1.50131300	0.06499500
C	5.16665000	0.01937200	-0.84925600
H	7.72689100	-0.13807100	1.40794300
H	7.19008200	-2.56644000	1.26550300
H	5.36307400	-3.33548800	-0.24650000
H	4.92791000	1.87992900	-1.90178300
H	4.06992600	-1.68626200	-1.57772700
C	0.57678700	6.21013400	0.44975900
H	0.77973600	7.28150200	0.40418300
C	1.52502900	5.16758100	-0.04739100
H	1.83224300	5.34262500	-1.09714700
C	-0.40601000	4.12594500	1.05793800
H	-0.10323900	3.93744700	2.10550300
H	-1.25446300	3.45522700	0.86587600
C	-0.72409800	5.61791200	0.87683500
H	-1.14208000	6.08833900	1.78280300
H	-1.49484900	5.74989300	0.08645400
C	0.75350500	3.87869200	0.13036900
H	2.47292900	5.17593100	0.53331000

TS II_endo

E = -26393.7556

$\boldsymbol{H}_{cor}$ = 0.8645

$\boldsymbol{-TS}_{cor}$ = -0.1113

G = -26393.0023

C	-1.19486000	2.26365400	-0.39345400
O	-0.87273200	0.97881000	-0.24090700
Sm	0.01803400	-0.92241800	-0.05223800
O	-1.78539500	-1.39679900	-1.71272000
C	-2.20194300	-0.39463400	-2.65403200
C	-3.54400600	0.11167700	-2.13092000
C	-4.03865800	-1.02366400	-1.21070500
C	-2.98623300	-2.10908200	-1.37549000
I	1.98979700	-0.37480300	-2.37311400
I	-1.77551300	-1.81194900	2.30610400
O	1.87405000	-2.32976100	1.05459900
C	2.99042000	-2.91087600	0.35336900
C	3.97280300	-3.31599200	1.43552500
C	3.03634000	-3.70343600	2.57532400
C	1.94444000	-2.65519600	2.45856000
O	1.38861900	0.41401700	1.53427200
C	2.81521900	0.63115600	1.49120900
C	3.05433900	1.91224300	2.26818600
C	1.91785400	1.88603800	3.28630700
C	0.76900300	1.33167600	2.46562300
O	0.03330100	-3.36258000	-0.85549200
C	0.10889300	-3.77355400	-2.23999800
C	0.09146000	-5.29268600	-2.21420000
C	-0.70652100	-5.58237600	-0.94680700
C	-0.18377200	-4.50921500	-0.01386800
H	2.62900700	-3.78333400	-0.21799600
H	3.36838500	-2.16647600	-0.36021500
H	2.19305700	-1.73472800	3.01299600
H	0.94985200	-2.98980100	2.78250700
H	4.59976000	-2.45755900	1.72634100
H	4.63795100	-4.12873600	1.11281300
H	3.51508800	-3.69705100	3.56413500
H	2.62274800	-4.71073000	2.40506500
H	3.30829700	-0.23346400	1.96600700
H	3.12352800	0.68262900	0.43756600
H	0.25080700	2.11320100	1.88875300
H	0.02656800	0.76257300	3.04216600
H	2.96691000	2.78400500	1.60343100
H	4.05429100	1.93808200	2.72163400
H	1.68590900	2.87455000	3.70602100
H	2.15772400	1.20760000	4.12131400
H	-0.76498400	-3.35374700	-2.76030500
H	1.01582500	-3.33246600	-2.67595600
H	0.77860800	-4.80721000	0.43955800
H	-0.87426900	-4.21318500	0.78806900
H	1.11422300	-5.69205500	-2.11914000
H	-0.35397400	-5.71682600	-3.12450100
H	-0.55579300	-6.59554800	-0.54949800
H	-1.78424400	-5.43855300	-1.12585300
H	-1.40809400	0.35891500	-2.70409500
H	-2.29751400	-0.88221300	-3.64057300
H	-3.22197200	-2.79935400	-2.20704600
H	-2.79031300	-2.68372200	-0.46127200
H	-4.24091800	0.31456400	-2.95603900
H	-3.40409700	1.04105300	-1.56307300
H	-5.04601000	-1.37468000	-1.46723900
H	-4.06267300	-0.69086300	-0.16433300
C	-0.28886000	3.03177500	-1.31751900
H	-0.72685000	4.00813000	-1.57555100

C	1.09239300	3.21063000	-0.69809100
C	2.13318300	3.82488800	-1.63107500
H	1.00880900	3.82707400	0.21501200
H	1.45261800	2.21968000	-0.38220300
H	-0.18026500	2.46049200	-2.25597200
C	3.81621300	4.95443000	-0.12284000
C	5.01908800	4.96206500	0.58094100
C	5.90563600	3.89275900	0.46143200
H	2.19980000	3.21158900	-2.54365900
C	5.58099700	2.82277800	-0.37164300
C	4.37702200	2.81832100	-1.07337000
H	3.12469000	5.79596000	-0.02463000
C	3.47533400	3.88268000	-0.95589900
H	5.26741400	5.80950300	1.22386000
H	6.85000200	3.89774900	1.00972700
H	6.27236400	1.98378400	-0.47942200
H	1.81662900	4.83611300	-1.93725100
H	4.11952600	1.97266300	-1.71672000
C	-3.70551900	4.39158500	1.25872300
H	-4.04409300	5.36291400	1.63094900
C	-2.61527800	4.28177700	0.24087200
H	-2.94523700	4.62383800	-0.76003900
C	-3.11799400	2.07144800	1.23725700
H	-2.57332000	1.31123900	1.81593900
H	-3.90750200	1.52379400	0.69219300
C	-3.75574400	3.16276800	2.10617200
H	-3.15131300	3.32877900	3.02273600
H	-4.76939500	2.91012000	2.45795800
C	-2.22638500	2.81934300	0.27905600
H	-1.76818600	4.94818200	0.50506900
C	-5.67065100	3.25935500	-1.06795200
H	-5.16117100	3.39015000	-2.02562800
C	-6.19775500	1.96482500	-0.81407100
N	-6.62980400	0.89810300	-0.63092300
C	-5.75062600	4.27017600	-0.17115200
H	-6.31809800	4.15932500	0.75376500
H	-5.42436700	5.27155400	-0.45144700

TS II_exo

E = -26393.7620

$\boldsymbol{H}_{cor}$ = 0.8646

$\boldsymbol{-TS}_{cor}$ = -0.1111

G = -26393.0084

C	1.58164200	1.73665400	-1.25691100
O	0.47719800	1.15454600	-0.78957000
Sm	-1.08456300	-0.06003200	-0.07293700
O	-2.22143600	1.19125400	-1.93074600
C	-1.50821100	1.54391600	-3.12399300
C	-1.29262500	3.04930800	-3.01710600
C	-2.44510800	3.52230800	-2.10546200
C	-3.18236100	2.23571100	-1.74082700
I	-0.43943000	-2.34126000	-2.04967700
I	-2.05899600	2.05145100	1.97995600
O	-1.82350500	-1.83836200	1.62337600
C	-2.21282600	-3.18542300	1.29086100
C	-2.16353100	-3.94885200	2.60087100
C	-2.55937300	-2.87107900	3.60530800
C	-1.84658500	-1.65022900	3.05320700
O	0.76253300	-0.73607100	1.43276700
C	1.34491400	-2.05309400	1.57017400
C	2.73760500	-1.82578200	2.12759000
C	2.56733300	-0.53469300	2.92198400
C	1.62983500	0.25545700	2.03084500
O	-3.54721700	-0.74363700	-0.36147000

C	-4.12858800	-1.23650400	-1.59030700
C	-5.58346300	-1.53031000	-1.26347400
C	-5.86838700	-0.53589300	-0.14231600
C	-4.57056900	-0.58677400	0.63737500
H	-3.23334900	-3.15966700	0.87126700
H	-1.53059400	-3.55449100	0.51305100
H	-0.80495600	-1.58352200	3.40910800
H	-2.34238900	-0.69241200	3.26134500
H	-1.13961200	-4.30387800	2.80055400
H	-2.83146300	-4.82117000	2.60088600
H	-2.25965600	-3.09674500	4.63787500
H	-3.65079400	-2.71810700	3.59344300
H	0.71097800	-2.63136200	2.26316000
H	1.33423000	-2.54086000	0.58531800
H	2.16670100	0.77591800	1.22337700
H	0.98651700	0.97343200	2.55924600
H	3.45933500	-1.68170400	1.31133000
H	3.08268100	-2.67488600	2.73240000
H	3.51844200	-0.01130300	3.08868000
H	2.09772000	-0.73044200	3.90010700
H	-4.02154400	-0.44728700	-2.34980000
H	-3.54763800	-2.10984400	-1.91728200
H	-4.54539800	-1.45789700	1.31628100
H	-4.33903400	0.31786500	1.21638500
H	-5.69765400	-2.56221900	-0.89388800
H	-6.23669200	-1.41086400	-2.13871200
H	-6.74002900	-0.79886200	0.47267800
H	-6.02686000	0.47507800	-0.55134100
H	-0.59132000	0.94472200	-3.14988500
H	-2.14031400	1.27459300	-3.98933900
H	-4.03820500	2.04736000	-2.41545800
H	-3.51704200	2.19618100	-0.69645700

H	-1.31045400	3.52642300	-4.00683800
H	-0.31767000	3.25435300	-2.55412000
H	-3.11627800	4.23796700	-2.60046700
H	-2.05484800	3.99930700	-1.19565900
C	2.43017600	0.86142800	-2.13679500
H	3.22620400	1.44904400	-2.61898800
C	3.02643300	-0.28566800	-1.32913900
C	3.81181900	-1.30557000	-2.14788500
H	3.68256300	0.12498200	-0.54433600
H	2.20144700	-0.81128100	-0.82420000
H	1.79589800	0.44273800	-2.93832800
C	5.46014200	-2.23572700	-0.47880400
C	5.87107400	-3.21927900	0.41826100
C	5.13272200	-4.39405300	0.55824600
H	3.16341200	-1.72188800	-2.93546100
C	3.98150000	-4.57594300	-0.20674600
C	3.57357200	-3.59127700	-1.10548100
H	6.02687800	-1.30484400	-0.55923000
C	4.30764100	-2.40939100	-1.25500900
H	6.77185600	-3.06360200	1.01604600
H	5.45501700	-5.16592100	1.26054100
H	3.39584100	-5.49275000	-0.10607800
H	4.66172000	-0.81167000	-2.64801200
H	2.66305100	-3.73047400	-1.69434800
C	3.00622700	5.07549000	-0.64860400
H	3.68000600	5.91165500	-0.85604900
C	3.13092200	3.76408100	-1.35132000
H	3.15427800	3.91655900	-2.45186800
C	1.06134000	3.85375600	0.01054900
H	1.16533500	3.53057900	1.06162200
H	-0.01392700	3.76776300	-0.20291500
C	1.60629200	5.27651500	-0.17129100

H	1.52992500	5.90176600	0.73350700
H	1.02753100	5.81226400	-0.95396200
C	1.90410300	3.00039300	-0.90411400
H	4.08050900	3.24879400	-1.11336400
C	3.95508700	3.22830600	1.87717100
H	3.09242100	2.98352300	2.50115500
C	4.72583500	2.12576000	1.42036500
N	5.33960700	1.20050100	1.06418300
C	4.22471000	4.50530400	1.52666200
H	5.11955700	4.75623300	0.95632900
H	3.64353800	5.32423200	1.95061700

Int III_endo

E = -26393.8109

$\boldsymbol{H}_{\mathbf{cor}}$ = 0.8685

$\boldsymbol{-TS}_{\mathbf{cor}}$ = -0.1097

G = -26393.0521

C	-0.87981000	2.41029300	-0.39041400
O	-0.76278300	1.08604200	-0.27788300
Sm	-0.13285100	-0.91670600	-0.05276000
O	-1.96087900	-1.19076800	-1.73358700
C	-2.19643600	-0.17082500	-2.71595400
C	-3.42615000	0.56908900	-2.20988900
C	-4.17308700	-0.48500100	-1.36619700
C	-3.26270900	-1.70834800	-1.41586400
I	1.91336600	-0.67116600	-2.35649900
I	-2.06251100	-1.53009600	2.28507400
O	1.50912700	-2.53426500	1.09621400
C	2.53537600	-3.28188400	0.41551600
C	3.45364900	-3.78484500	1.51265600
C	2.47229900	-4.01026300	2.65853100
C	1.53385100	-2.82636300	2.50881600
O	1.39744300	0.24710300	1.53010200
C	2.84047700	0.27708700	1.48383600
C	3.24639100	1.51723100	2.25783300
C	2.11776300	1.64272000	3.27733300
C	0.90482300	1.23961800	2.46039400
O	-0.41777200	-3.35931900	-0.81350300
C	-0.38888700	-3.80942100	-2.18806100
C	-0.69325100	-5.29876000	-2.13880000
C	-1.52337700	-5.41922000	-0.86469500
C	-0.80939500	-4.44327200	0.04695900
H	2.05849100	-4.11375400	-0.13067000
H	3.00846200	-2.61603700	-0.31874200
H	1.90665600	-1.93359600	3.03833200
H	0.50229800	-3.01123900	2.83728800
H	4.19071500	-3.01117900	1.78238900
H	4.00302300	-4.68881700	1.21580400
H	2.94693900	-4.04235800	3.64881000
H	1.92531500	-4.95592800	2.51330900
H	3.21781500	-0.64374300	1.95887900
H	3.15086400	0.28605400	0.42973200
H	0.49038500	2.08027400	1.88240800
H	0.09729200	0.77303000	3.04147200
H	3.27377000	2.39162800	1.59142200
H	4.24154200	1.41220700	2.71036400
H	2.01708200	2.65433500	3.69394700
H	2.26880700	0.94174700	4.11449700
H	-1.15438400	-3.24138400	-2.73682000
H	0.59451400	-3.56122900	-2.61100500
H	0.09597200	-4.89810600	0.48851500
H	-1.42557800	-4.02483200	0.85468400
H	0.23616600	-5.88277600	-2.04195300
H	-1.21565800	-5.64365400	-3.04163400

H	-1.55552700	-6.43679500	-0.45167500
H	-2.55837500	-5.08477200	-1.04111700
H	-1.28509800	0.43388900	-2.78823900
H	-2.37651200	-0.66992900	-3.68524100
H	-3.55551200	-2.41227100	-2.21654700
H	-3.18433700	-2.24457100	-0.46163300
H	-4.03584000	0.96191300	-3.03490900
H	-3.10987600	1.40866300	-1.57777600
H	-5.17605400	-0.70578600	-1.75402400
H	-4.28832100	-0.14222700	-0.32931100
C	0.11005900	3.04947600	-1.32543200
H	-0.19370100	4.07718100	-1.57776100
C	1.50738300	3.04322200	-0.71525200
C	2.61764200	3.50915600	-1.65402200
H	1.51221800	3.66770400	0.19598100
H	1.73418600	2.01411800	-0.39749900
H	0.13290500	2.46682800	-2.26262100
C	4.44519500	4.40324900	-0.15600600
C	5.63718500	4.24631100	0.54887000
C	6.36375600	3.06163300	0.43833600
H	2.59757600	2.89100400	-2.56557200
C	5.89145900	2.04197200	-0.38721600
C	4.69887500	2.20217700	-1.09006600
H	3.87915200	5.33449900	-0.06453600
C	3.95641000	3.38396100	-0.98112900
H	6.00265300	5.05501400	1.18571600
H	7.29934300	2.93734600	0.98761600
H	6.45753100	1.11305700	-0.48810900
H	2.44064200	4.55355100	-1.96106200
H	4.32466300	1.39630300	-1.72721800
C	-3.31711400	4.80961900	1.08126400
H	-3.32435800	5.75668200	1.64384300
C	-1.96521100	4.59588100	0.38153600
H	-1.92322500	5.08059400	-0.60846600
C	-2.74837300	2.44048600	1.30401600
H	-2.25030000	1.73525600	1.98765600
H	-3.49303700	1.82955600	0.76405100
C	-3.41586500	3.61033300	2.03572000
H	-2.84948800	3.84658000	2.95160000
H	-4.45037600	3.39697000	2.34518500
C	-1.78709500	3.09505500	0.34229200
H	-1.16844800	5.06940500	0.98461700
C	-4.55674200	3.80435000	-0.91760700
H	-3.74947200	3.68890600	-1.64464000
C	-5.58088600	2.86958500	-0.96412600
N	-6.44366500	2.07172300	-1.00535300
C	-4.49514800	4.90160300	0.08803600
H	-5.44147200	4.94913400	0.64746300
H	-4.39440900	5.85638400	-0.46072200

Int III_exo

E = -26393.8136

H_{cor} = 0.8687

$-TS_{cor}$ = -0.1098

G = -26393.0547

C	1.59378000	1.83585700	-1.14660200
O	0.53440200	1.20010100	-0.65193600
Sm	-1.05035900	-0.05886300	-0.04640100
O	-2.13200700	1.25876700	-1.87744700
C	-1.42984900	1.61010500	-3.07642100
C	-1.26911400	3.12226700	-2.99767800
C	-2.46381300	3.57404800	-2.13147700
C	-3.13309800	2.26760400	-1.70493900
I	-0.34853200	-2.22440100	-2.13339100

I	-2.09986100	1.93560600	2.07418900
O	-1.84838700	-1.93258400	1.51593000
C	-2.23395800	-3.25604200	1.09682700
C	-2.21681200	-4.09432600	2.36088600
C	-2.63943300	-3.07633100	3.41539000
C	-1.91599700	-1.82453500	2.95318500
O	0.73142800	-0.83607000	1.47362700
C	1.30605400	-2.16278800	1.54096500
C	2.69234400	-1.97378800	2.12606800
C	2.51277800	-0.74082100	3.00552900
C	1.58739900	0.10927600	2.15902300
O	-3.50670600	-0.72892400	-0.44652400
C	-4.04528800	-1.17942100	-1.70964900
C	-5.49672800	-1.52979600	-1.42993000
C	-5.83946500	-0.57899800	-0.28747800
C	-4.56170100	-0.61667100	0.52590100
H	-3.24483500	-3.20255900	0.65673300
H	-1.53475800	-3.58238400	0.31504700
H	-0.88580300	-1.77350800	3.34280900
H	-2.42374100	-0.88224600	3.20004400
H	-1.19782300	-4.46054300	2.56522800
H	-2.88364800	-4.96487300	2.29351700
H	-2.36503500	-3.36083100	4.44041400
H	-3.73062400	-2.92411500	3.38539700
H	0.66218100	-2.77637900	2.19341700
H	1.30251500	-2.59208700	0.52922200
H	2.13590600	0.68262900	1.39767900
H	0.93581600	0.78827600	2.72721300
H	3.42193000	-1.77211200	1.32922100
H	3.03271300	-2.86261300	2.67370100
H	3.46040100	-0.22789900	3.21608200
H	2.03070500	-1.00288100	3.96184600
H	-3.94907800	-0.35329200	-2.43095300
H	-3.42977400	-2.01771700	-2.06354100
H	-4.53307000	-1.50108600	1.18686500
H	-4.36778500	0.27965500	1.13090100
H	-5.58570900	-2.57575300	-1.09424200
H	-6.13195600	-1.40434300	-2.31748900
H	-6.71872600	-0.88486600	0.29599100
H	-6.01723700	0.43878800	-0.67087100
H	-0.49271300	1.04231300	-3.08844900
H	-2.04803300	1.30001500	-3.93833000
H	-3.99374200	2.01876500	-2.35315500
H	-3.44510100	2.24959300	-0.65291800
H	-1.26926100	3.57959300	-3.99669500
H	-0.31660300	3.36637500	-2.50759700
H	-3.16696300	4.21689500	-2.67918800
H	-2.12063100	4.12882400	-1.24743100
C	2.47578600	0.99123400	-2.02416400
H	3.27733200	1.59848000	-2.47016500
C	3.05867200	-0.18188600	-1.24482600
C	3.86802400	-1.16101500	-2.08988900
H	3.69318800	0.19717200	-0.42746400
H	2.22714800	-0.73267400	-0.77985200
H	1.86678000	0.59461200	-2.85673800
C	5.48206400	-2.16421400	-0.42922800
C	5.88126500	-3.19081600	0.42370900
C	5.15167700	-4.37781700	0.48167000
H	3.23941300	-1.54219400	-2.91064600
C	4.02103300	-4.52807800	-0.31999300
C	3.62475400	-3.49977600	-1.17385800
H	6.04342500	-1.22677100	-0.44729900
C	4.35041600	-2.30524500	-1.24161600
H	6.76541000	-3.05949200	1.05126900

H	5.46466400	-5.18365500	1.14911600
H	3.44242000	-5.45409800	-0.28333900
H	4.72631800	-0.64153300	-2.54830500
H	2.73013900	-3.61477600	-1.79148600
C	3.11785600	5.03317400	-0.18055600
H	3.56150000	5.97236400	-0.54772300
C	2.95238000	4.00839500	-1.31097800
H	2.66976300	4.54347200	-2.23792900
C	0.93464100	3.92957000	0.09164400
H	0.74586900	3.38414400	1.03094800
H	-0.07122500	4.08921700	-0.33133100
C	1.67520500	5.26350600	0.30064300
H	1.63097800	5.62457600	1.34026500
H	1.21768800	6.04875900	-0.32164900
C	1.82412100	3.13419600	-0.83305900
H	3.88387800	3.46445300	-1.52806800
C	3.65573900	3.20551500	1.51764500
H	2.70060500	3.10841400	2.03807400
C	4.47879000	2.08853800	1.49208800
N	5.17227300	1.13873700	1.46308600
C	4.05718800	4.51164100	0.92188900
H	5.07929300	4.43083200	0.52209000
H	4.08693900	5.26785800	1.72918900

TS III_endo

E = -26393.8056

$\boldsymbol{H_{cor}}$ = 0.8682

$\boldsymbol{-TS_{cor}}$ = -0.1078

G = -26393.0451

C	0.96693100	2.48985200	-0.30460100
O	0.17301300	1.44827800	-0.16294200
Sm	-0.73354000	-0.47156100	0.04981600
O	-2.27160700	0.61104100	-1.52438500
C	-1.82520700	1.33602700	-2.69370200
C	-3.08437300	1.92053100	-3.34021800
C	-4.23890200	1.29718600	-2.55059700
C	-3.60663000	1.04762900	-1.19768300
I	0.85054800	-1.62901000	-2.34179100
I	-2.42991600	0.30462800	2.47644800
O	-0.54649400	-2.80191400	1.13291200
C	-0.33038400	-4.03094900	0.41207700
C	0.05109100	-5.05076800	1.46785500
C	-0.76470200	-4.57709100	2.66619400
C	-0.65382100	-3.06793700	2.54765800
O	1.27834700	-0.68853800	1.50511900
C	2.35022600	-1.64978600	1.38018200
C	3.52184500	-1.05215500	2.13626600
C	2.82102500	-0.23258800	3.21609900
C	1.63627800	0.33655000	2.45986500
O	-2.62149500	-2.06591400	-0.64402100
C	-2.94191900	-2.40544300	-2.01260100
C	-4.10982500	-3.37378900	-1.92986900
C	-4.79344700	-2.94311900	-0.63606800
C	-3.60025700	-2.63500700	0.24540400
H	-1.26859400	-4.30527700	-0.10010900
H	0.43654000	-3.84590500	-0.35193900
H	0.25376100	-2.68060400	3.04056300
H	-1.51711400	-2.51082200	2.93543200
H	1.12940600	-4.99210800	1.68781200
H	-0.17611900	-6.07937500	1.15569500
H	-0.39130200	-4.94704400	3.63090000
H	-1.81502900	-4.89503000	2.56430300
H	2.01249800	-2.59753800	1.83067900
H	2.54197000	-1.81327400	0.31084000

H	1.89991700	1.24979700	1.90340200
H	0.74866600	0.53628400	3.07614100
H	4.11488500	-0.40491300	1.47338900
H	4.19252200	-1.82580500	2.53345800
H	3.45395200	0.55584300	3.64596400
H	2.47760100	-0.88181200	4.03802900
H	-3.20856000	-1.47369500	-2.53295100
H	-2.03880300	-2.81850300	-2.48275800
H	-3.18392900	-3.55545800	0.69224500
H	-3.78799600	-1.90746900	1.04702800
H	-3.74875200	-4.41128900	-1.84228600
H	-4.75904800	-3.31527800	-2.81419900
H	-5.44800100	-3.71222000	-0.20356600
H	-5.39339900	-2.03311200	-0.79865700
H	-1.12291200	2.11606900	-2.36815100
H	-1.29070100	0.60994800	-3.32226200
H	-4.07107400	0.24934800	-0.60457400
H	-3.54814700	1.97110700	-0.60199300
H	-3.13758800	1.69139600	-4.41343600
H	-3.09697600	3.01038000	-3.21056800
H	-4.56806700	0.34773200	-3.00402900
H	-5.10858900	1.96428200	-2.47929600
C	2.08167000	2.32725400	-1.29438700
H	2.52623200	3.30169700	-1.54989500
C	3.14749600	1.37580000	-0.75781100
C	4.23071100	0.99460500	-1.76403900
H	3.61944600	1.81754000	0.13794700
H	2.64151300	0.45482600	-0.43043300
H	1.65990000	1.89534900	-2.21970500
C	6.24807600	0.38281900	-0.37121900
C	7.05396300	-0.55591100	0.27032700
C	6.78900300	-1.91743000	0.12973200
H	3.75159200	0.57461900	-2.66260200
C	5.71789700	-2.32944400	-0.66222500
C	4.91384700	-1.38794100	-1.30175100
H	6.46080600	1.44939800	-0.25660200
C	5.16513700	-0.01790900	-1.16212600
H	7.89607600	-0.22252800	0.88090400
H	7.42084800	-2.65476600	0.62927200
H	5.50679400	-3.39406500	-0.78661700
H	4.78926400	1.89206600	-2.07824700
H	4.06762600	-1.71364900	-1.91259600
C	0.78284400	5.88100800	1.11108100
H	1.28765200	6.73897600	1.57996000
C	1.72493700	4.75786100	0.67640100
H	2.36045000	5.02698700	-0.18039800
C	-0.29507400	3.71888200	1.52344800
H	-0.04915300	2.96560400	2.28941100
H	-1.30711800	3.46594100	1.17817400
C	-0.17291100	5.15759000	2.07643600
H	0.25793700	5.14876100	3.08961300
H	-1.14517500	5.66795200	2.14659200
C	0.71332100	3.66821500	0.39631100
H	2.38714700	4.45437600	1.50759200
C	-0.30528700	5.05073400	-0.95485200
H	0.27380700	4.84951800	-1.86026600
C	-1.64535300	4.63894600	-1.03530000
N	-2.75620000	4.26331100	-1.05570200
C	0.04543100	6.29923100	-0.16756400
H	-0.85826000	6.87883100	0.07780800
H	0.69507000	6.94845000	-0.77628100

TS III_exo

E = -26393.8063

$\boldsymbol{H}_{cor}$ = 0.8681

$\boldsymbol{-TS}_{cor}$ = -0.1080

G = -26393.0462

C	1.61687300	1.96822600	-0.87686500
O	0.58525600	1.24375800	-0.48557100
Sm	-0.99090400	-0.11855700	-0.02023800
O	-1.84740000	1.16215800	-2.00039900
C	-1.07710600	1.28700300	-3.21037100
C	-0.89737800	2.77724000	-3.39765900
C	-2.24541800	3.31376600	-2.92090100
C	-2.60518600	2.37162700	-1.77662500
I	-0.20031200	-2.26677000	-2.10047500
I	-2.11695500	1.74711000	2.15818300
O	-1.88425300	-2.04341400	1.41054100
C	-2.29771500	-3.33636900	0.92703300
C	-2.23159100	-4.24837200	2.13555700
C	-2.64070400	-3.30068000	3.25840800
C	-1.95157100	-2.00904600	2.85450100
O	0.72565000	-0.95471500	1.51835000
C	1.33271500	-2.26781000	1.55344900
C	2.71545600	-2.05549800	2.13587600
C	2.50102100	-0.87400900	3.07652800
C	1.51901700	-0.02063300	2.29997000
O	-3.43897500	-0.61344100	-0.54983700
C	-3.91950700	-1.00107200	-1.85426900
C	-5.27235600	-1.63305600	-1.59877300
C	-5.78903200	-0.78467300	-0.44147300
C	-4.53430200	-0.58691100	0.39078400
H	-3.32850100	-3.25460100	0.53863000
H	-1.63296100	-3.61425500	0.09839900
H	-0.92138500	-1.94762600	3.23953400
H	-2.48353500	-1.09427300	3.14884400
H	-1.20098600	-4.60740700	2.28715700
H	-2.88703900	-5.12435000	2.03503900
H	-2.33343500	-3.63898000	4.25739600
H	-3.73486000	-3.16870400	3.26685400
H	0.70902600	-2.91459100	2.19487300
H	1.33409700	-2.67091600	0.53149200
H	2.03004000	0.64919200	1.59776700
H	0.82485500	0.56230100	2.92127900
H	3.42699200	-1.79218000	1.34128600
H	3.09287600	-2.95670900	2.63731200
H	3.42624900	-0.31952800	3.28306000
H	2.06246300	-1.20496900	4.03219400
H	-4.00523900	-0.09759000	-2.48040600
H	-3.17153100	-1.66988000	-2.30153100
H	-4.38570000	-1.40861000	1.11010600
H	-4.49044200	0.36354900	0.93906000
H	-5.15380800	-2.68373300	-1.28749000
H	-5.91853700	-1.61101400	-2.48705500
H	-6.59702200	-1.25953200	0.13184600
H	-6.16049800	0.18319200	-0.81475700
H	-0.14735200	0.72294100	-3.07066700
H	-1.64701400	0.82756600	-4.03703300
H	-3.67499300	2.11185000	-1.74634000
H	-2.31881400	2.76884800	-0.79077900
H	-0.66703000	3.04623100	-4.43765000
H	-0.07895000	3.13343000	-2.75312700
H	-2.99001800	3.24433400	-3.72925000
H	-2.20503000	4.36054600	-2.58902200
C	2.64981500	1.24821000	-1.69421300
H	3.49197000	1.91930200	-1.91851700

C	3.15145700	-0.02702400	-1.02453400
C	4.07708800	-0.86379700	-1.90335300
H	3.68157600	0.23791300	-0.09677400
H	2.28395800	-0.64503700	-0.74664100
H	2.19225900	0.97778600	-2.66615600
C	5.63028100	-2.02823600	-0.29222200
C	5.99913500	-3.13359800	0.47146900
C	5.27092400	-4.31961600	0.38461600
H	3.54799100	-1.15896200	-2.82430500
C	4.17392000	-4.39007400	-0.47297400
C	3.80886000	-3.28327800	-1.23754400
H	6.19046000	-1.09386400	-0.20273700
C	4.53201500	-2.08788600	-1.15814000
H	6.85893200	-3.06649600	1.14166800
H	5.55957800	-5.18703500	0.98211600
H	3.59695900	-5.31457900	-0.55018500
H	4.95026800	-0.26218300	-2.20646700
H	2.93944200	-3.33816900	-1.89777400
C	2.50305500	5.46065500	-0.07763000
H	2.92249900	6.42378600	-0.40408900
C	2.62918800	4.33619500	-1.10716000
H	2.23073100	4.65169500	-2.08885900
C	0.50199700	4.06413500	0.02162200
H	0.08587400	3.63876800	0.94460100
H	-0.30590100	3.99054600	-0.72512000
C	0.98706200	5.52463200	0.16618100
H	0.74733800	5.95912000	1.14860900
H	0.51218100	6.16776900	-0.59094500
C	1.72362400	3.30412200	-0.47070200
H	3.66593300	3.99940600	-1.25159300
C	2.78075800	3.50185900	1.40342800
H	1.91812700	3.33571300	2.05517200
C	3.77947900	2.51648100	1.53827900
N	4.58938500	1.67165900	1.60066400
C	3.22044700	4.93341100	1.16814200
H	4.30919400	4.96614600	1.01014600
H	2.99804900	5.56440300	2.04517500

Int IV

E = -26393.8260

H_{cor} = 0.8704

$-TS_{cor}$ = -0.1073

G = -26393.0628

C	1.23662800	2.29879700	-0.50047400
O	0.41892900	1.31908700	-0.11869900
Sm	-0.84478700	-0.35966600	0.02771800
O	-2.12838800	1.11660900	-1.48423200
C	-1.48348400	1.85073600	-2.55846900
C	-2.55572300	2.76659800	-3.11363400
C	-3.38210900	3.06675700	-1.86765300
C	-3.39809600	1.72391100	-1.16516400
I	0.51702400	-1.73143700	-2.38917500
I	-2.37449300	0.64076300	2.51957500
O	-1.20028100	-2.72984800	1.00225200
C	-1.24325700	-3.94279400	0.22771200
C	-1.02186900	-5.05926900	1.22902800
C	-1.71988600	-4.50413600	2.46640500
C	-1.35401100	-3.03119200	2.40547900
O	1.03108300	-1.04997900	1.50943800
C	1.91762500	-2.18009800	1.34984900
C	3.18222900	-1.81499700	2.10578500
C	2.65023100	-0.89449600	3.19997600
C	1.58883700	-0.11014100	2.45494600
O	-3.01823900	-1.45390700	-0.77859900

C	-3.34839200	-1.68972300	-2.16462900
C	-4.65377300	-2.46511200	-2.14122300
C	-5.31177200	-1.94262700	-0.86807200
C	-4.12248000	-1.83077000	0.06536500
H	-2.23479200	-4.02066100	-0.25218700
H	-0.48234100	-3.86682200	-0.56062400
H	-0.39525500	-2.81983200	2.90692000
H	-2.11156200	-2.35289700	2.82042700
H	0.05496300	-5.19524000	1.41999900
H	-1.43019900	-6.01893100	0.88335900
H	-1.39760800	-4.97391200	3.40580600
H	-2.81083400	-4.63423800	2.37930100
H	1.42041100	-3.06446900	1.78209900
H	2.07265700	-2.34805100	0.27506300
H	2.01581900	0.73134500	1.88842600
H	0.76209700	0.25869300	3.07792200
H	3.87842400	-1.27570300	1.44665300
H	3.70476500	-2.70209000	2.48779800
H	3.42050300	-0.24105000	3.63229400
H	2.19584300	-1.47734800	4.01785700
H	-3.45441600	-0.71119400	-2.65957700
H	-2.50681000	-2.22197500	-2.62865900
H	-3.88948200	-2.80042100	0.53939600
H	-4.22074300	-1.07198100	0.85365000
H	-4.45950900	-3.54651900	-2.05452100
H	-5.25104700	-2.30001000	-3.04837400
H	-6.09502200	-2.60293600	-0.47103800
H	-5.75672500	-0.94970400	-1.04207500
H	-0.64654500	2.41883100	-2.12513800
H	-1.09135700	1.11164100	-3.27100500
H	-4.20032900	1.07073400	-1.55301400
H	-3.48275800	1.78226300	-0.07087200

H	-3.16430000	2.24757500	-3.87238900
H	-2.12268600	3.67246000	-3.55704000
H	-4.39441100	3.43337600	-2.08695000
H	-2.86401700	3.82074100	-1.25613400
C	2.36195400	1.89431600	-1.40080100
H	2.96741700	2.76871100	-1.69389600
C	3.25737400	0.82617700	-0.76956600
C	4.30323700	0.24888600	-1.72145100
H	3.75714200	1.23189700	0.12848400
H	2.60689500	0.00582500	-0.42936200
H	1.94305200	1.46839400	-2.33242800
C	6.12659500	-0.68801500	-0.24320100
C	6.74510100	-1.74945600	0.41491300
C	6.27575800	-3.05045200	0.23868900
H	3.79583400	-0.09313000	-2.63757800
C	5.18809500	-3.27858500	-0.60347700
C	4.57125900	-2.21458800	-1.25868600
H	6.50061800	0.32991300	-0.10213400
C	5.02998200	-0.90301500	-1.08624700
H	7.60161900	-1.56033800	1.06591900
H	6.76129500	-3.88406200	0.75035700
H	4.81695500	-4.29486900	-0.75498100
H	5.01893000	1.03379500	-2.01891300
H	3.71129500	-2.39511000	-1.90918800
C	1.87894000	5.21064600	1.73402500
H	2.47924700	5.73508800	2.49055200
C	2.39531100	3.84222400	1.28501000
H	3.38538600	3.87487300	0.80297700
C	0.01333900	3.77227700	1.18945500
H	-0.22592600	2.82951000	1.69672200
H	-0.87868300	4.04587900	0.60740300
C	0.45308500	4.86940800	2.18742600

H	0.45525100	4.48466400	3.21854700
H	-0.20254500	5.75315700	2.17289600
C	1.23993700	3.57093100	0.28273900
H	2.41357500	3.09648500	2.09391900
C	1.39543400	4.85150900	-0.60538300
H	2.21014400	4.68263200	-1.32603800
C	0.20473500	5.13989500	-1.38938800
N	-0.75492700	5.36031500	-2.00657500
C	1.79305600	5.96863500	0.40249100
H	1.05664000	6.78409900	0.43367100
H	2.76213100	6.40457100	0.12007700

Int IV_exo

E = -26393.8258

$\boldsymbol{H_{cor}}$ = 0.8704

$\boldsymbol{-TS_{cor}}$ = -0.1080

G = -26393.0634

C	1.17941900	2.31107200	-0.80799300
O	0.39560100	1.30755600	-0.40902700
Sm	-0.82605200	-0.34049200	0.02468400
O	-2.07442900	0.79699400	-1.81924600
C	-1.41731900	1.20472600	-3.03670300
C	-2.52506200	1.79674700	-3.88432000
C	-3.39531300	2.47484600	-2.82916100
C	-3.32620700	1.49551700	-1.66711500
I	0.40938000	-2.18420600	-2.13652900
I	-2.30521000	1.23793700	2.23585100
O	-1.28232300	-2.44006800	1.44728400
C	-1.39176800	-3.78152100	0.93734600
C	-1.10079500	-4.68066000	2.12301800
C	-1.69224300	-3.87230400	3.27367100
C	-1.31646500	-2.45155000	2.89030200
O	1.05791400	-0.87509300	1.52607200
C	1.94036600	-2.01879200	1.50974400
C	3.26041300	-1.51547600	2.06138700
C	2.81433000	-0.43301900	3.03947100
C	1.65204600	0.19789000	2.30076000
O	-3.09139800	-1.45525500	-0.51241800
C	-3.43514200	-2.03887200	-1.78591500
C	-4.69329100	-2.84697700	-1.52882100
C	-5.36918500	-2.03229000	-0.43062400
C	-4.18274600	-1.61031400	0.41556000
H	-2.41871100	-3.93327200	0.55990600
H	-0.69187700	-3.88213200	0.09639100
H	-0.31534600	-2.17265300	3.25625900
H	-2.02988100	-1.68412500	3.22056200
H	-0.01351300	-4.80181500	2.25381100
H	-1.54420300	-5.67953200	2.01006200
H	-1.29958300	-4.15391500	4.26029200
H	-2.78793100	-3.98890300	3.29855100
H	1.49822000	-2.80142900	2.15139700
H	1.99672300	-2.39026200	0.47726100
H	1.99005800	0.96733300	1.59760500
H	0.86702100	0.61457800	2.94755800
H	3.86885300	-1.08236600	1.25481800
H	3.84584400	-2.31952700	2.52714200
H	3.59506800	0.30945700	3.25289800
H	2.48083100	-0.87631700	3.99215300
H	-3.61236100	-1.22161000	-2.50506400
H	-2.57304300	-2.62319000	-2.13527100
H	-3.90859600	-2.38742700	1.14921100
H	-4.31056400	-0.66031700	0.95190500
H	-4.43679900	-3.85270400	-1.15798700
H	-5.30503500	-2.96525800	-2.43369600

H	-6.11396200	-2.59725100	0.14661900
H	-5.86968300	-1.14913300	-0.85993100
H	-0.64079700	1.94616100	-2.78573700
H	-0.93409800	0.31446500	-3.46111800
H	-4.13652100	0.74872600	-1.71027200
H	-3.34144100	1.97175700	-0.67619500
H	-3.08566100	0.99787900	-4.39651900
H	-2.14321000	2.48929500	-4.64689300
H	-4.42822900	2.65282800	-3.15867800
H	-2.96010300	3.44381400	-2.54038100
C	2.36492000	1.94738300	-1.64123800
H	2.96457300	2.84331900	-1.86626000
C	3.23456700	0.87044700	-0.99025000
C	4.31400300	0.29788300	-1.90625400
H	3.70114200	1.27367800	-0.07645800
H	2.57390800	0.04540100	-0.68010100
H	2.01724900	1.54583100	-2.61698200
C	6.13178500	-0.59579200	-0.39592800
C	6.73820700	-1.63524600	0.30705800
C	6.25294300	-2.93687700	0.18884900
H	3.84588000	-0.06294200	-2.83678000
C	5.15949900	-3.18846800	-0.63923400
C	4.55522900	-2.14670300	-1.34001700
H	6.51189800	0.42408900	-0.29289700
C	5.03241600	-0.83507900	-1.22822100
H	7.59544500	-1.42691900	0.95115600
H	6.72832700	-3.75283500	0.73739100
H	4.77225900	-4.20482600	-0.74234500
H	5.03034100	1.08770500	-2.18791100
H	3.68763300	-2.34662900	-1.97431300
C	0.75386600	5.92893300	-0.07159600
H	1.00467200	6.98268300	-0.25475100
C	1.51587400	4.89873200	-0.91315500
H	1.31607100	4.98722300	-1.99207900
C	-0.66184000	4.02324600	-0.45334400
H	-1.27642800	3.52171900	0.30717300
H	-1.00242700	3.66589400	-1.43460000
C	-0.70392100	5.56624300	-0.37227500
H	-1.39024200	5.93921700	0.40296800
H	-1.02287600	6.00766500	-1.32937000
C	0.83893000	3.66646900	-0.29318200
H	2.60431500	4.92213600	-0.74846800
C	1.14736900	3.92266100	1.23346900
H	0.40752400	3.39667000	1.85618100
C	2.46912300	3.43825200	1.60511300
N	3.53399500	3.06728000	1.88835100
C	1.06868900	5.47400200	1.36021000
H	2.02949000	5.88093100	1.70522100
H	0.29686800	5.78489400	2.07862400

Product_endo

E = -26393.8420

H_{cor} = 0.8713

$-TS_{cor}$ = -0.1119

G = -26393.0826

C	1.15223400	2.50064100	-0.18787900
O	0.42411300	1.55612500	0.09052800
Sm	-0.84119000	-0.57164100	0.03894500
O	-2.16411500	1.10462800	-1.41792900
C	-1.68783400	1.78851900	-2.59011500
C	-2.87559500	2.57493500	-3.11285400
C	-3.61028000	2.91706000	-1.82004100
C	-3.43154200	1.64723900	-1.00754600
I	0.80981000	-1.44561300	-2.58426900

I	-2.38040500	0.34200400	2.70360700
O	-0.95353200	-3.03004000	0.86813000
C	-0.91897500	-4.16697500	-0.00766700
C	-0.74975400	-5.36875900	0.90494000
C	-1.50929600	-4.91924100	2.15036700
C	-1.13207800	-3.45009100	2.23061800
O	1.25162900	-1.07975700	1.47810500
C	2.16152400	-2.17087100	1.25072800
C	3.35656100	-1.89277300	2.14334000
C	2.70203200	-1.17926700	3.32330300
C	1.66229700	-0.32009300	2.62585200
O	-2.96995900	-1.72809800	-0.87573400
C	-3.31253100	-1.81922000	-2.26851200
C	-4.58697700	-2.64511900	-2.31568600
C	-5.24831500	-2.27444800	-0.99064200
C	-4.05033400	-2.20684300	-0.06095700
H	-1.87136200	-4.21012300	-0.56510900
H	-0.10462500	-4.01753000	-0.73147500
H	-0.17922000	-3.30405300	2.76980700
H	-1.88887900	-2.80106400	2.69382500
H	0.31556900	-5.52472400	1.14115000
H	-1.13833800	-6.29447800	0.45839900
H	-1.23724300	-5.47318400	3.05942900
H	-2.59530200	-5.02946100	1.99687000
H	1.65352400	-3.11044400	1.52901800
H	2.39524100	-2.20347100	0.17754800
H	2.09938700	0.63232400	2.27715100
H	0.76544300	-0.09914700	3.22295700
H	4.07030500	-1.22981200	1.63007800
H	3.89291300	-2.81008100	2.42133400
H	3.40424300	-0.58378300	3.92329100
H	2.21123700	-1.90508800	3.99228600
H	-3.47179600	-0.79774800	-2.65317800
H	-2.45868300	-2.25875800	-2.80404200
H	-3.78591900	-3.20776700	0.32609800
H	-4.16390900	-1.52181400	0.79208100
H	-4.34925400	-3.72122900	-2.33572900
H	-5.20216400	-2.41682400	-3.19697300
H	-6.00312900	-2.99855700	-0.65418700
H	-5.73130800	-1.28649500	-1.06458500
H	-0.86999200	2.46711900	-2.29596500
H	-1.28641900	1.03474500	-3.28220000
H	-4.21716600	0.90467600	-1.23764900
H	-3.40603500	1.79604300	0.08200000
H	-3.50327900	1.94359400	-3.76324500
H	-2.56359900	3.46481400	-3.67512800
H	-4.66840200	3.17384600	-1.96795400
H	-3.11527400	3.76906700	-1.32905900
C	2.35648200	2.27269600	-1.05985000
H	2.95586400	3.18775400	-1.17886100
C	3.21229800	1.12196700	-0.53376400
C	4.32580700	0.72497800	-1.49905600
H	3.64793300	1.39795700	0.44290100
H	2.55717500	0.25487100	-0.36515900
H	1.96918800	1.99104500	-2.05647100
C	6.18895900	-0.23402200	-0.08730100
C	6.88064000	-1.31330000	0.45953800
C	6.50924500	-2.61694600	0.13380700
H	3.87099400	0.45125100	-2.46412600
C	5.44641700	-2.83001400	-0.74307300
C	4.75557300	-1.74920100	-1.28759000
H	6.48786700	0.78679500	0.16765500
C	5.11784300	-0.43657200	-0.96474200
H	7.71676800	-1.13560200	1.13944200

H	7.05167100	-3.46445400	0.55799700
H	5.15107000	-3.84752400	-1.00868200
H	4.99322600	1.58456100	-1.68031800
H	3.91288600	-1.91797700	-1.96404600
C	1.15399100	5.60534000	1.80344000
H	1.61540500	6.22556700	2.58369300
C	1.86394800	4.28039300	1.50449900
H	2.89306500	4.39994200	1.13138900
C	-0.48078100	3.94759800	1.15326200
H	-0.68460200	3.00621600	1.68115100
H	-1.31541800	4.10193500	0.45478800
C	-0.26902500	5.13169800	2.12716000
H	-0.33998400	4.79518500	3.17181200
H	-1.00698800	5.93603700	1.99279500
C	0.85895500	3.85140500	0.40805200
H	1.86226800	3.58035400	2.35272900
C	0.99160600	5.06732100	-0.55415400
H	1.90012600	4.96340000	-1.16637500
C	-0.12261700	5.19325100	-1.48337200
N	-1.01287300	5.29012700	-2.22261300
C	1.14506900	6.27531700	0.42425300
H	0.32513500	6.99839900	0.31552900
H	2.08684000	6.80333200	0.21916100

Product_exo

E = -26393.8424

$\boldsymbol{H}_{\mathbf{cor}}$ = 0.8713

$\boldsymbol{-TS}_{\mathbf{cor}}$ = -0.1124

G = -26393.0836

C	0.79962500	2.68539100	-0.71041000
O	0.29831100	1.57390500	-0.60364900
Sm	-0.72035700	-0.61313000	-0.00806900
O	-2.21587400	0.35609100	-1.93182700
C	-1.95553500	0.38477300	-3.33240800
C	-2.72149800	1.60243300	-3.84204600
C	-3.87982400	1.75477700	-2.83412400
C	-3.59373700	0.68417600	-1.77599600
I	0.91733200	-2.18614400	-2.27770700
I	-2.43707900	1.02137900	2.19541400
O	-0.65652600	-2.69191100	1.55849800
C	-0.49589600	-4.04659200	1.11096200
C	-0.03175800	-4.81883300	2.33312300
C	-0.74661400	-4.07368400	3.45732600
C	-0.63075000	-2.63192800	2.99340300
O	1.39205200	-0.52416800	1.49029800
C	2.45179300	-1.49225700	1.37370200
C	3.59151700	-0.95065200	2.21614700
C	2.84282800	-0.20358500	3.31673600
C	1.68800400	0.40994400	2.54506400
O	-2.78905000	-2.15879600	-0.42008200
C	-3.01404000	-2.93151400	-1.60911400
C	-3.99719300	-4.01457200	-1.20231300
C	-4.83148700	-3.29129300	-0.14847300
C	-3.77486700	-2.47416900	0.57517500
H	-1.47225500	-4.41679900	0.74982100
H	0.20966800	-4.04715300	0.26716000
H	0.32622500	-2.18084700	3.30688400
H	-1.44756900	-1.97411200	3.32402800
H	1.06086300	-4.73275900	2.44952800
H	-0.28498300	-5.88656900	2.27782200
H	-0.30008100	-4.23292500	4.44850500
H	-1.80454100	-4.37931600	3.50688100
H	2.08165400	-2.45885200	1.76004700
H	2.69328800	-1.61431700	0.30927400

H	1.98095900	1.37325600	2.09836100
H	0.77132300	0.56386900	3.13299700
H	4.20995500	-0.26001200	1.62228600
H	4.24879600	-1.74810100	2.58878000
H	3.44910300	0.55932600	3.82406800
H	2.47035200	-0.90838000	4.07884400
H	-3.44509300	-2.27224500	-2.38380700
H	-2.04153500	-3.29649000	-1.97023600
H	-3.28716400	-3.05635800	1.37631400
H	-4.13869800	-1.53247300	1.01052400
H	-3.46477600	-4.86847500	-0.75231100
H	-4.58440600	-4.39045600	-2.05152100
H	-5.37596100	-3.96615600	0.52638000
H	-5.56633300	-2.62778100	-0.63272900
H	-0.86747400	0.41826000	-3.46920000
H	-2.32773300	-0.54917000	-3.79271400
H	-4.20388700	-0.22120400	-1.95105000
H	-3.73792600	1.01086600	-0.73682100
H	-3.06871200	1.46003000	-4.87477600
H	-2.07608400	2.49347100	-3.83364000
H	-4.86292100	1.59804600	-3.29957900
H	-3.88538100	2.75844800	-2.38657400
C	2.22849400	2.83568700	-1.14373000
H	2.68183800	3.68376800	-0.60569100
C	3.01854400	1.55358100	-0.95034700
C	4.45220500	1.61746700	-1.46666200
H	3.03534700	1.32199500	0.12585200
H	2.48311500	0.71867700	-1.42833700
H	2.20147000	3.14164600	-2.20852700
C	6.31616000	0.48626900	-0.18733700
C	6.99064000	-0.65549000	0.24480900
C	6.57884300	-1.91427800	-0.18680100
H	4.44513400	1.71334900	-2.56656600
C	5.49435400	-2.02098100	-1.05791300
C	4.82585600	-0.87914600	-1.49117200
H	6.63704000	1.47111800	0.16262300
C	5.22613900	0.39151700	-1.05762200
H	7.83941000	-0.55911100	0.92543500
H	7.10253300	-2.81043700	0.15264300
H	5.15718600	-3.00091700	-1.40258500
H	4.95784100	2.51500800	-1.07442500
H	3.96582900	-0.98676800	-2.15780000
C	-0.85947500	5.97814700	-0.22032000
H	-0.93311900	7.06216900	-0.37864700
C	0.32272000	5.27970400	-0.90489400
H	0.28427900	5.33986200	-2.00332400
C	-1.51424600	3.73131100	-0.80199500
H	-2.04466600	3.03865400	-0.13387800
H	-1.55564700	3.30489000	-1.81305300
C	-2.05349800	5.17888500	-0.75850300
H	-2.93980700	5.28208400	-0.11551500
H	-2.33268500	5.53125000	-1.76325500
C	-0.04054600	3.88666600	-0.37394200
H	1.30219400	5.64164100	-0.55765800
C	-0.07161700	4.14763800	1.17068000
H	-0.71205800	3.39318900	1.66028900
C	1.25659100	4.05640100	1.76391200
N	2.32942200	3.97820300	2.20396000
C	-0.64732000	5.59409800	1.25111900
H	0.07113100	6.26679600	1.73949000
H	-1.57998500	5.61537600	1.83133700

10. References

[1] M. Szostak, M. Spain, D. J. Procter, *J. Org. Chem.* **2012**, *77*, 3049–3059.

[2] A. Dahlén, G. Hilmersson, *Eur. J. Inorg. Chem.* **2004**, *2004*, 3020–3024.

[3] B. E. Love, E. G. Jones, *J. Org. Chem.* **1999**, *64*, 3755–3756.

[4] K. A. Spence, M. Hoffmann, N. K. Garg, *Org. Lett.* **2023**, *25*, 5044–5048.

[5] H. Wu, Q. Wang, J. Zhu, *Angew. Chem. Int. Ed.* **2018**, *57*, 2721-2725.

[6] R. Guo, Y.-C. Chang, L. Herter, C. Salome, S. E. Braley, T. C. Fessard, M. K. Brown, *J. Am. Chem. Soc.* **2022**, *144*, 7988–7994.

[7] N. J. Baxter, L. J. M. Rigoreau, A. P. Laws, M. I. Page, *J. Am. Chem. Soc.* **2000**, *122*, 3375–3385.

[8] S. Agasti, F. Beltran, E. Pye, N. Kaltsoyannis, G. Crisenza, D. J. Procter, *Nat. Chem.* **2023**, *15*, 535–541

[9] A. K. K. Fung, M. J. Sowden, M. L. Coote, M. S. Sherburn, *Org. Lett.* **2023**, *25*, 8145–8149.

[10] X. Ouyang, F. W. Fowler, J. W. Lauher, *J. Am. Chem. Soc.* **2003**, *125*, 12400–12401.

[11] D.-S. Ji, R. Zhang, X.-Y. Han, H.-L. Chai, Y. Gu, X.-Q. Hu, P.-F. Xu, *Org. Chem. Front.* **2024**, *11*, 2911–2916.

[12] Gaussian 16, Revision C.01, M. J. Frisch, G. W. Trucks, H. B. Schlegel, G. E. Scuseria, M. A. Robb, J. R. Cheeseman, G. Scalmani, V. Barone, G. A. Petersson, H. Nakatsuji, X. Li, M. Caricato, A. V. Marenich, J. Bloino, B. G. Janesko, R. Gomperts, B. Mennucci, H. P. Hratchian, J. V. Ortiz, A. F. Izmaylov, J. L. Sonnenberg, D. Williams-Young, F. Ding, F. Lipparini, F. Egidi, J. Goings, B. Peng, A. Petrone, T. Henderson, D. Ranasinghe, V. G. Zakrzewski, J. Gao, N. Rega, G. Zheng, W. Liang, M. Hada, M. Ehara, K. Toyota, R. Fukuda, J. Hasegawa, M. Ishida, T. Nakajima, Y. Honda, O. Kitao, H. Nakai, T. Vreven, K. Throssell, J. A .Montgomery, Jr., J. E. Peralta, F. Ogliaro, M. J. Bearpark, J. J. Heyd, E. N. Brothers, K. N. Kudin, V. N. Staroverov, T. A. Keith, R. Kobayashi, J. Normand, K. Raghavachari, A. P. Rendell, J. C. Burant, S. S. Iyengar, J. Tomasi, M. Cossi, J. M. Millam, M. Klene, C. Adamo, R. Cammi, J. W. Ochterski, R. L. Martin, K. Morokuma, O. Farkas, J. B. Foresman, D. J. Fox, Gaussian, Inc., Wallingford CT, 2016.

[13] C. Adamo, V. Barone, *J. Chem. Phys.* **1999**, *110*, 6158–6169.

[14] T. H. J. Dunning, *J. Chem. Phys.* **1989**, *90*, 1007–1023.

[15] a) For Sm, see: X. Cao, M. Dolg, *J. Mol. Struct. (THEOCHEM)* **2002**, *581*, 139–147; b) for I, see: J. M. Martin, A. Sundermann, *J. Chem. Phys.* **2001**, *114*, 3408–3420.

[16] S. Grimme, J. Antony, S. Ehrlich, H. Krieg, *J. Chem. Phys.* **2010**, *132*, 154104.

[17] E. R. Johnson, A. D. Becke, *J. Chem. Phys.* **2006**, *124*, 174104.

[18] L. Visscher, K. G. Dyall, *At. Data Nucl. Data Tables* **1997**, *67*, 207–224.

[19] a) For Sm, see: D. A. Pantazis, F. Neese, *J. Chem. Theory Comput.* **2009**, *5*, 2229–2238; b) For I, see: C. T. Campos, F. E. Jorge, *Mol. Phys.* **2013**, *111*, 167–173.

[20] J. Tomasi, B. Mennucci, R. Cammi, *Chem. Rev.* **2005**, *105*, 2999–3094.

[21] S. Grimme, *Chem. Eur. J.* **2012**, *18*, 9955–9964.

[22] G. Luchini, J. V. Alegre-Requena, I. Funes-Ardoiz, R. S. Paton, *F1000Research*, **2020**, *9*, 291.

^{1}H, $^{13}C\{H\}$, and ^{19}F NMR Spectra

^{1}H NMR (400 MHz, Chloroform-*d*) of **SI-4**:

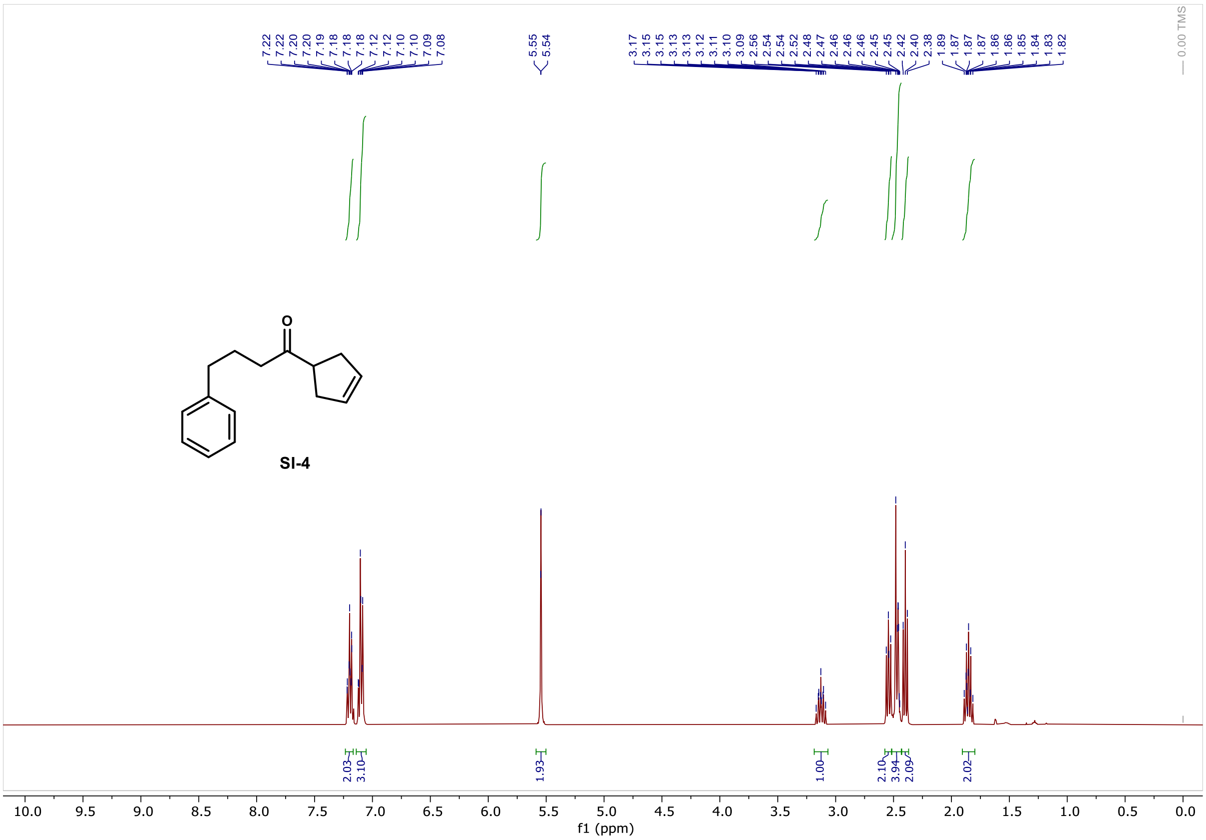

^{13}C NMR (101 MHz, Chloroform-*d*) of **SI-4**:

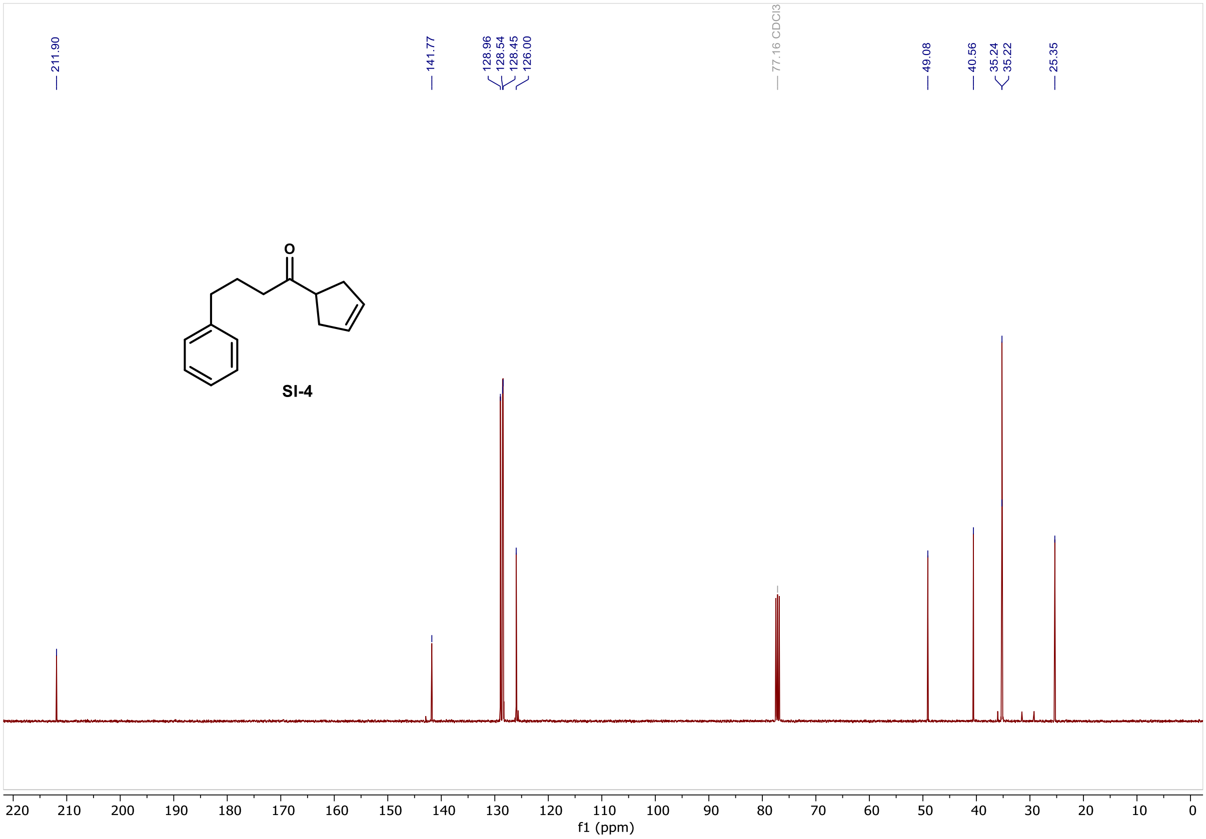

^{1}H NMR (400 MHz, Chloroform-*d*) of **SI-5 – diastereoisomer 1**:

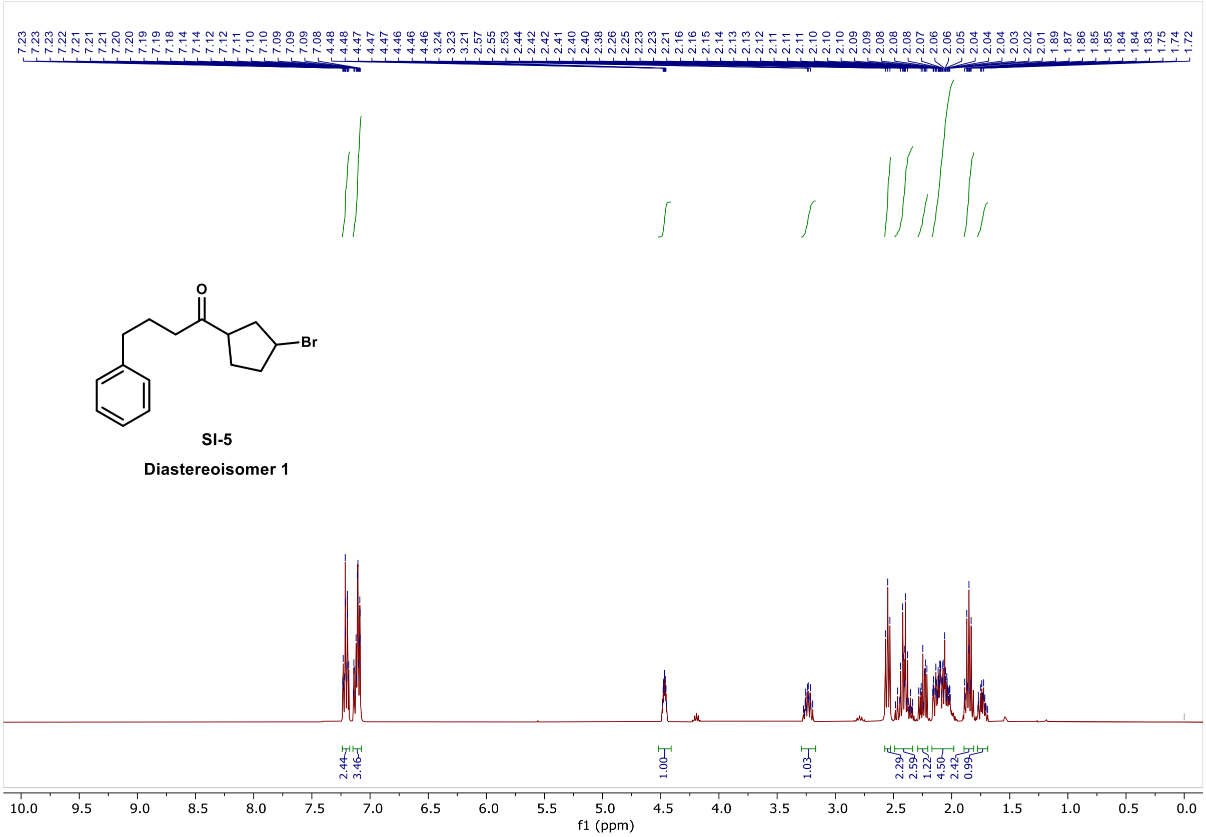

^{13}C NMR (101 MHz, Chloroform-*d*) of **SI-5 – diastereoisomer 1**:

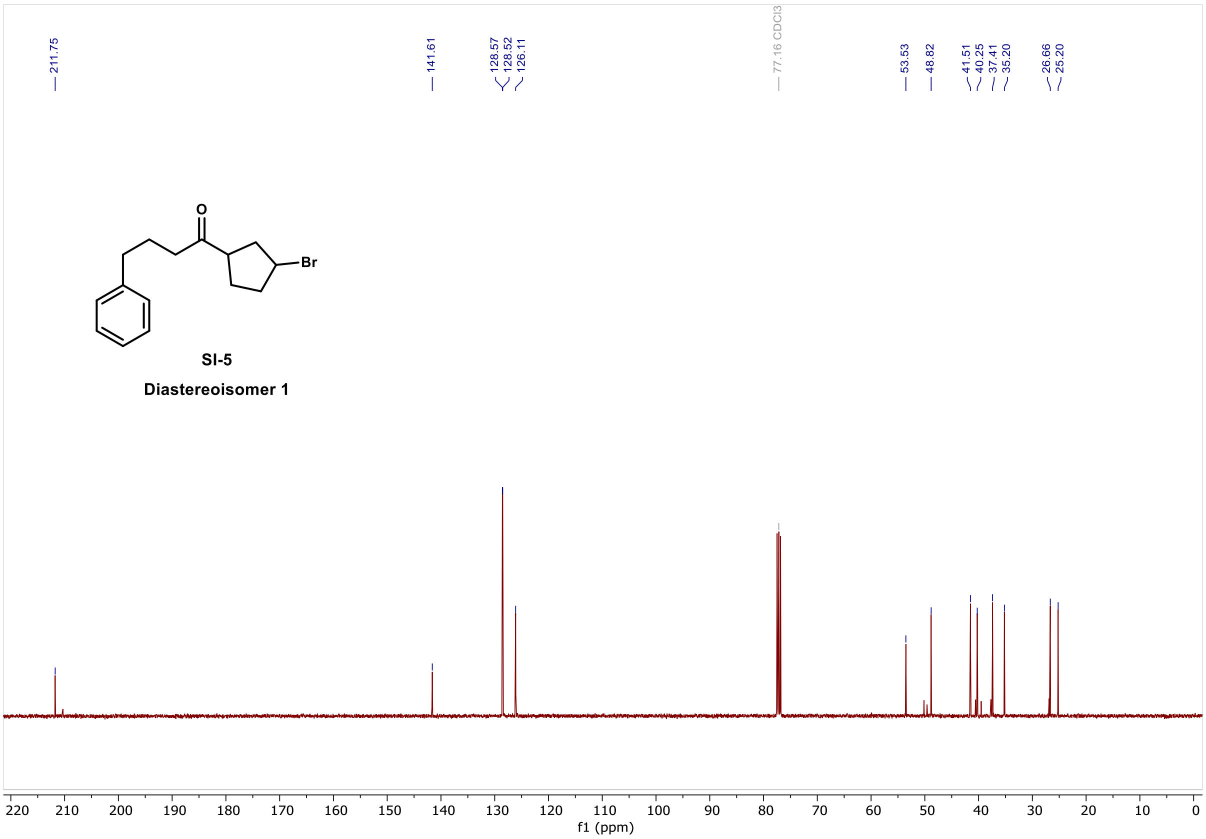

^{1}H NMR (400 MHz, Chloroform-*d*) of **SI-5 – diastereoisomer 2**:

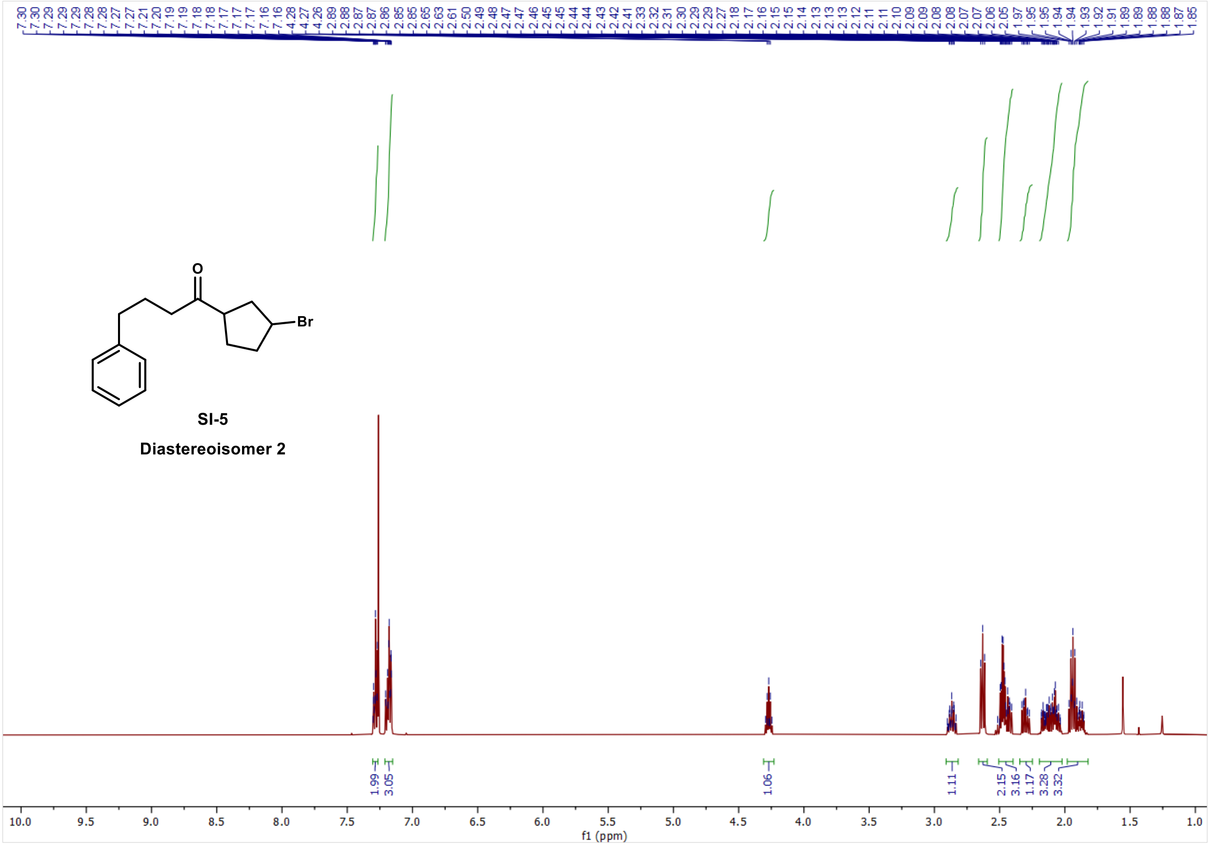

^{13}C NMR (101 MHz, Chloroform-*d*) of **SI-5 – diastereoisomer 2**:

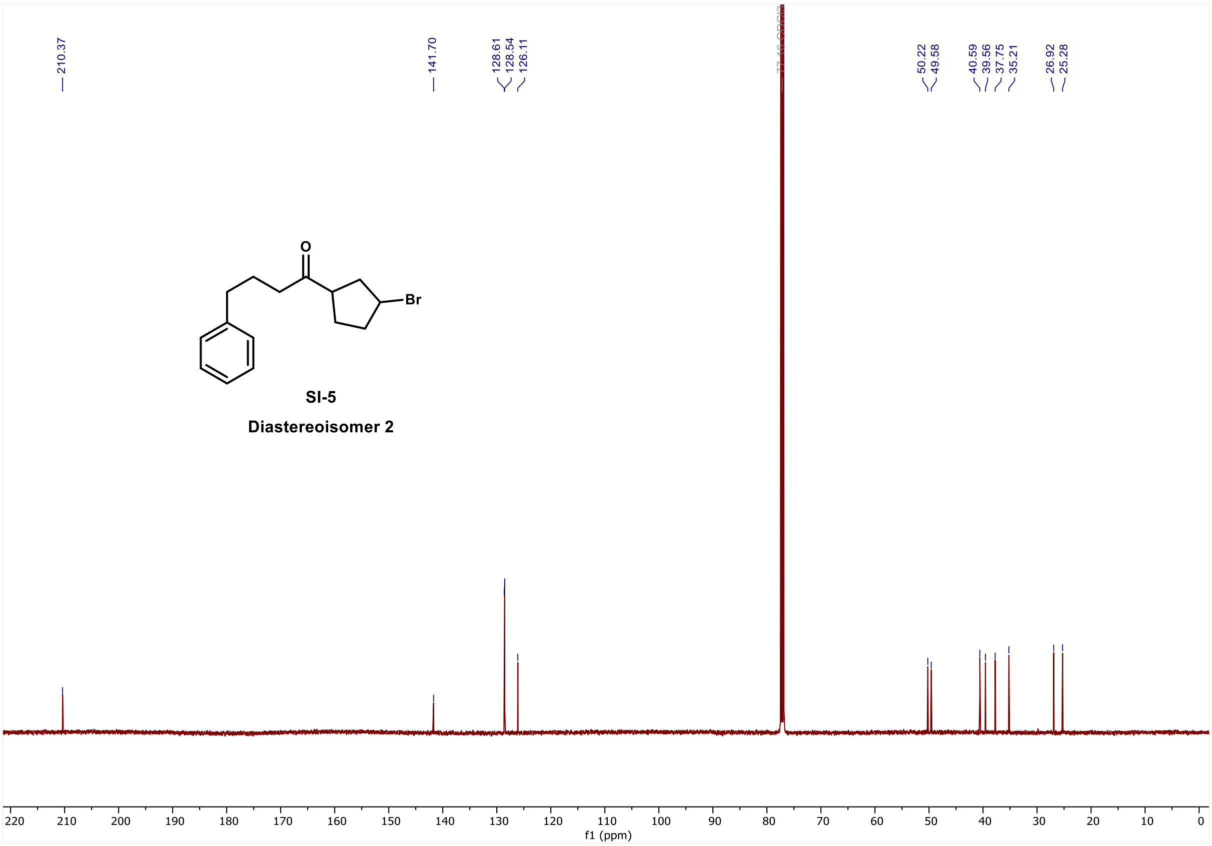

^{1}H NMR (500 MHz, Chloroform-*d*) of **1a**:

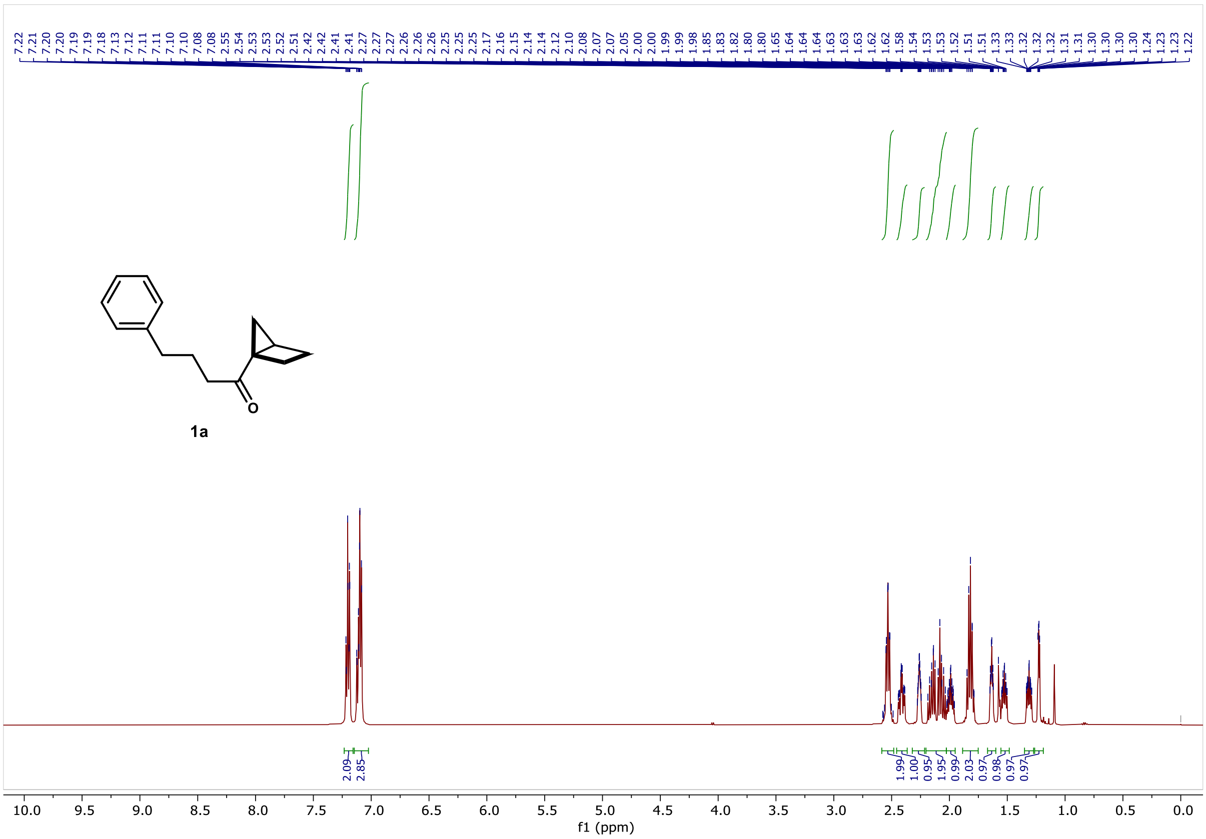

^{13}C NMR (126 MHz, Chloroform-*d*) of **1a**:

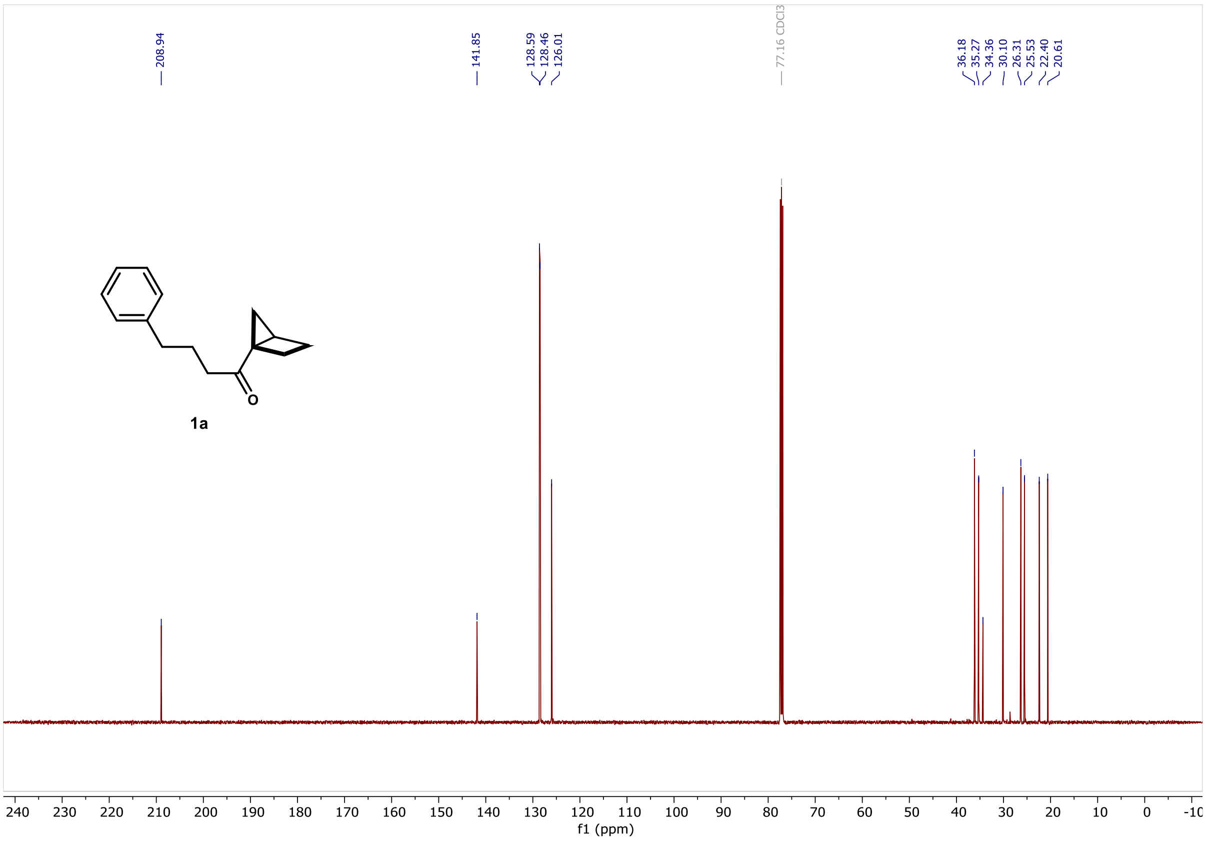

^{1}H-COSY NMR (500 MHz, Chloroform-*d*) of **1a**:

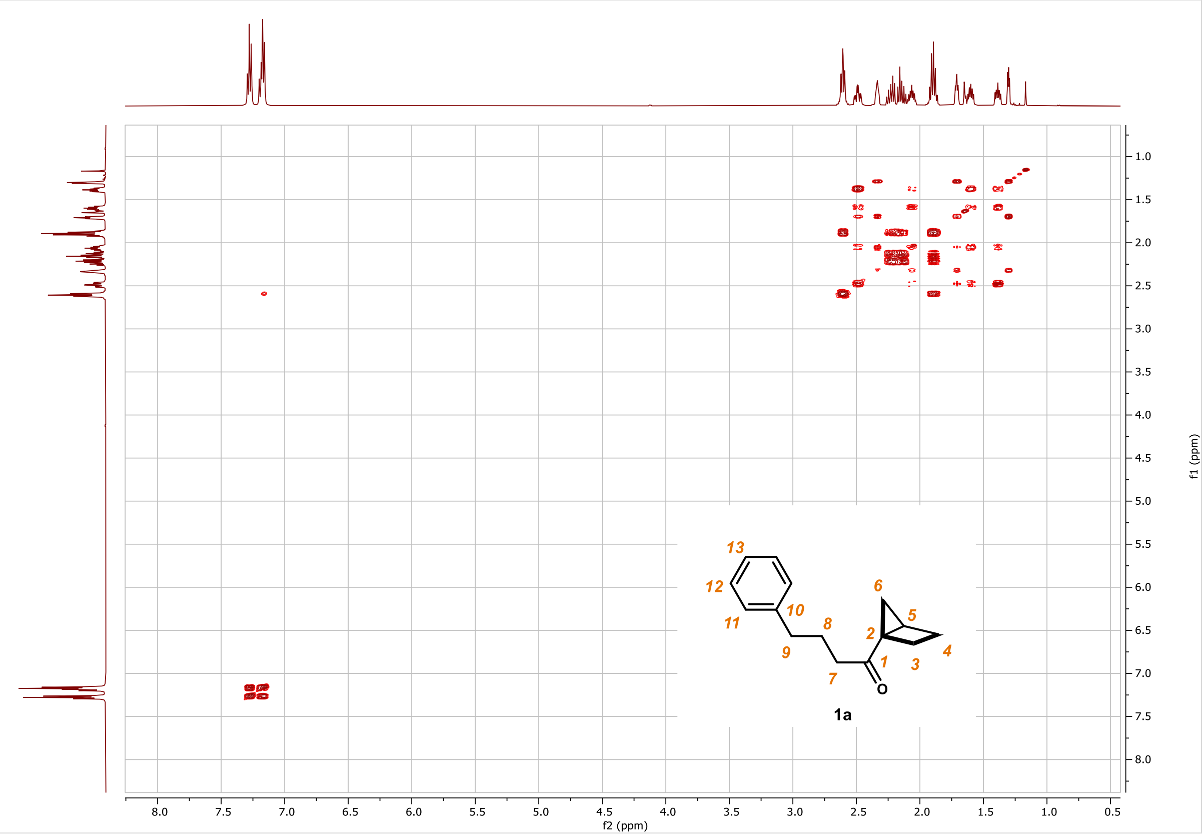

^{1}H-^{13}C HSQC NMR (500 MHz, Chloroform-*d*) of **1a**:

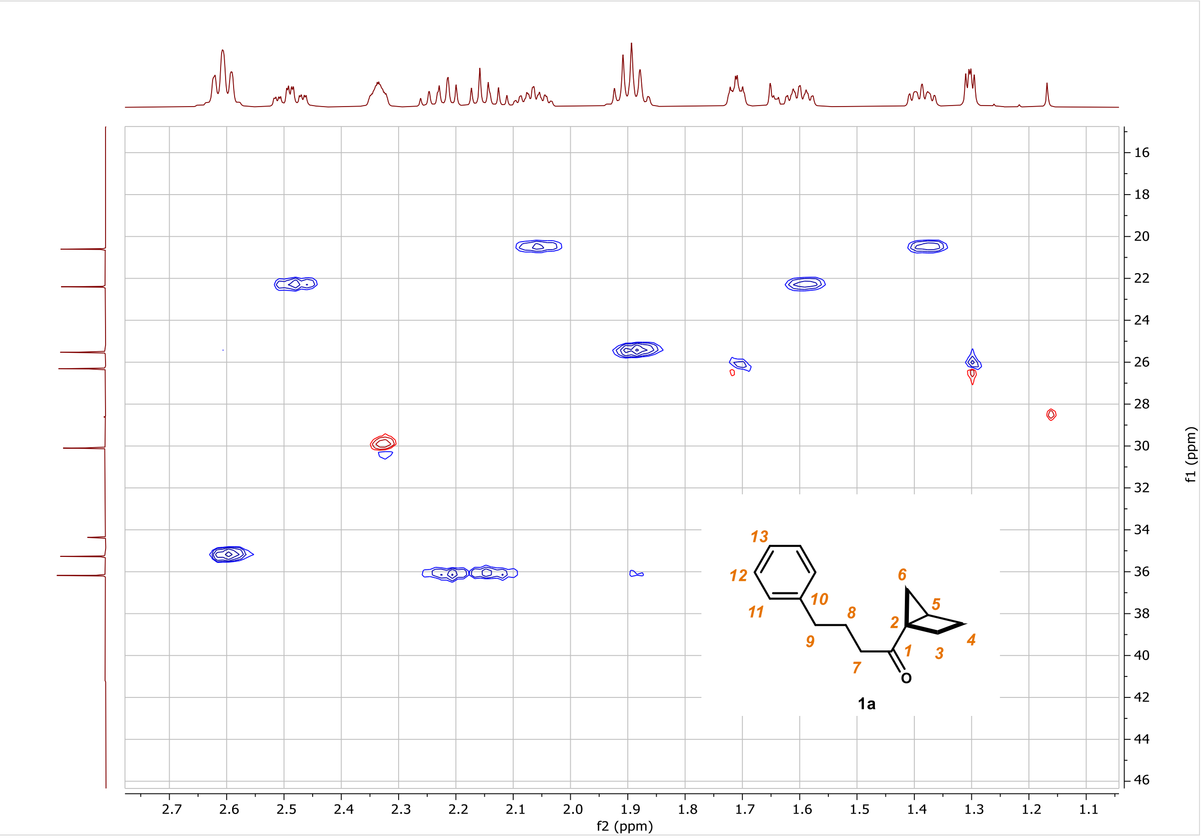

^{1}H-^{13}C HMBC NMR (500 MHz, Chloroform-*d*) of **1a**:

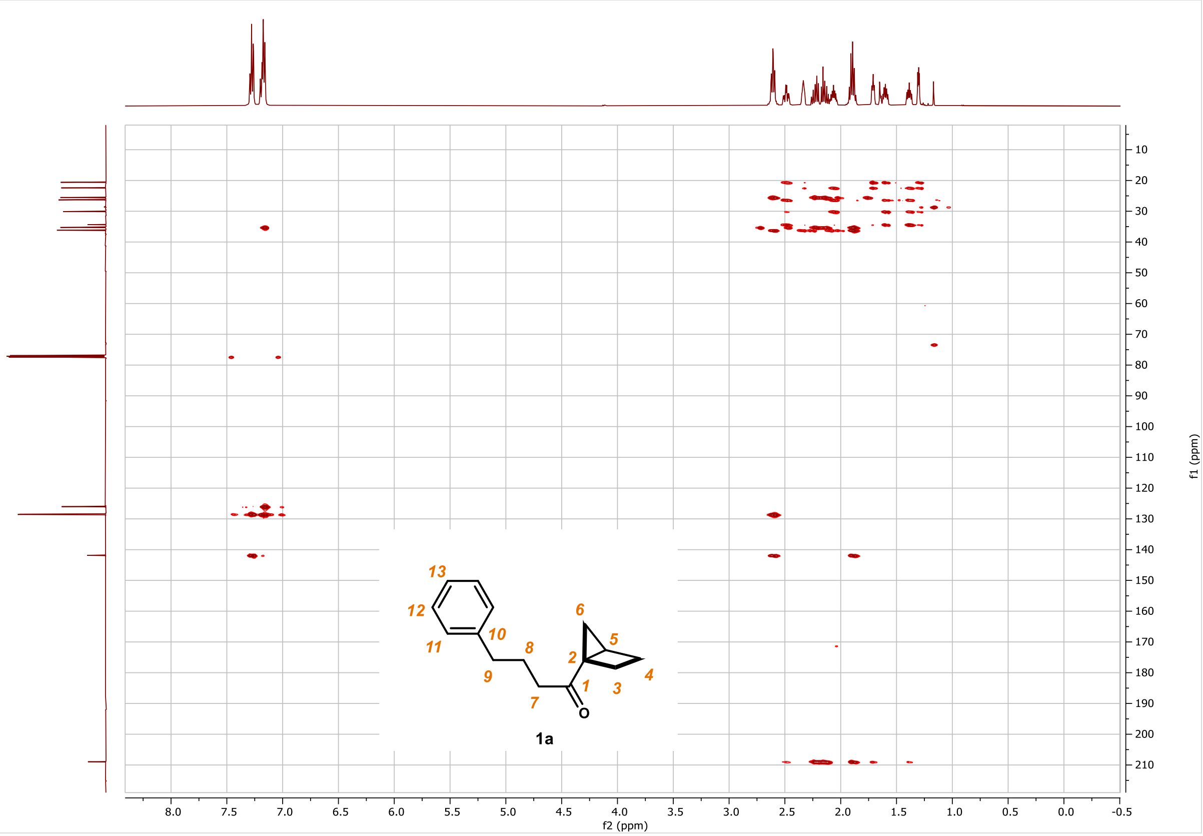

^{1}H-NOESY NMR (500 MHz, Chloroform-*d*) of **1a**:

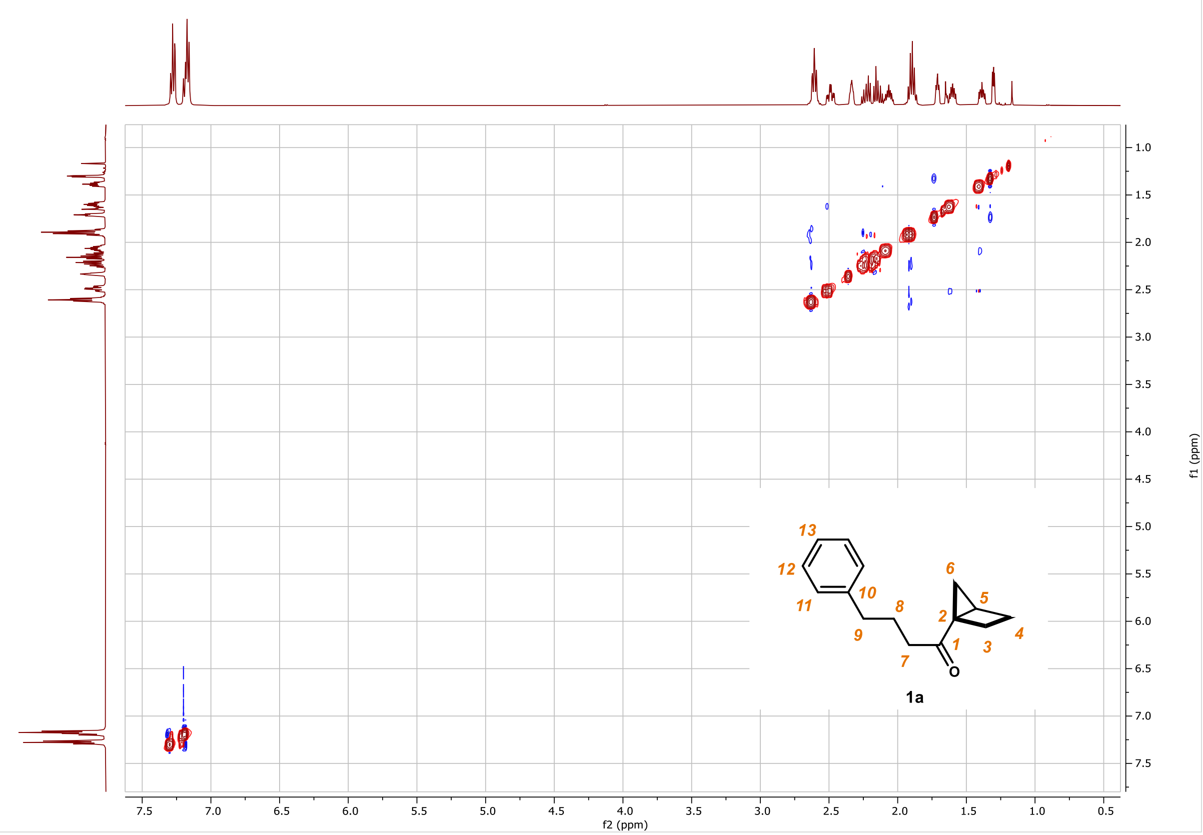

^{1}H NMR (500 MHz, Chloroform-*d*) of **SI-6**:

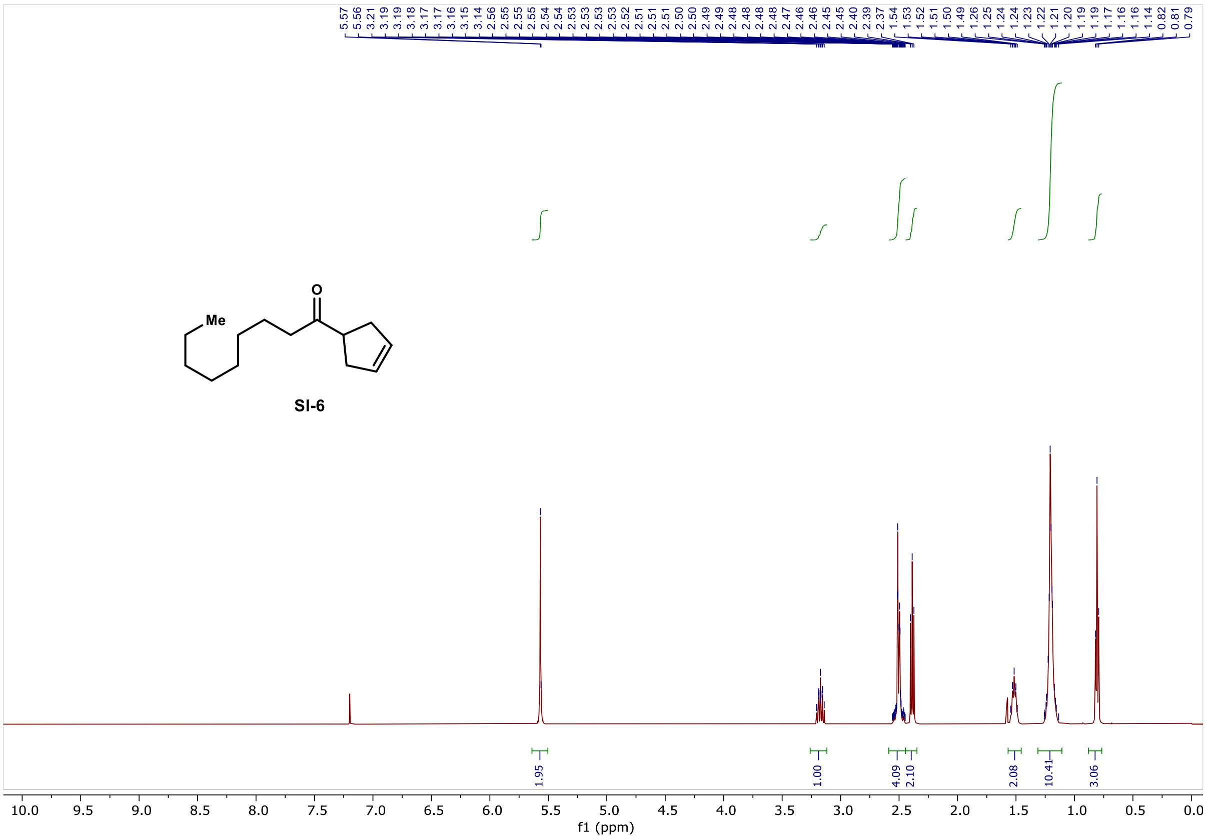

^{13}C NMR (126 MHz, Chloroform-*d*) of **SI-6**:

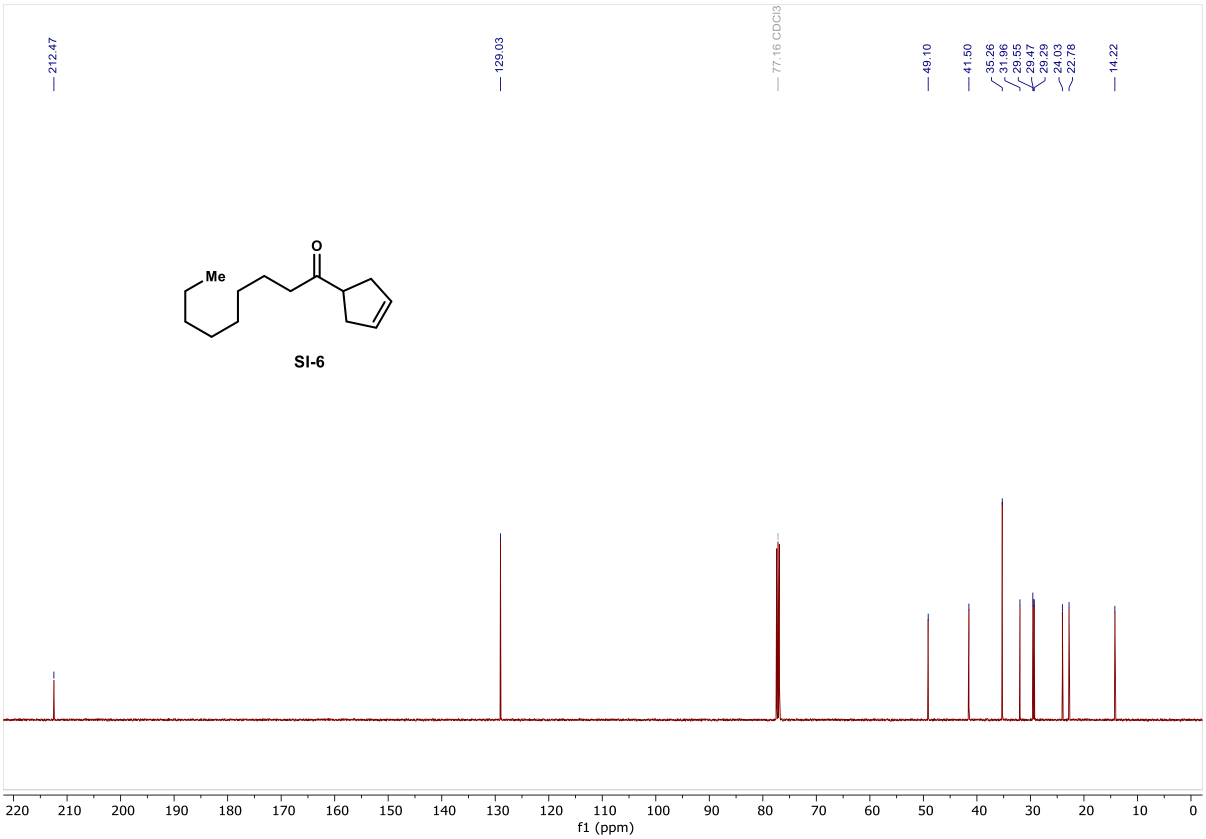

^{1}H NMR (400 MHz, Chloroform-*d*) of **SI-7 – mixture of diastereoisomers**:

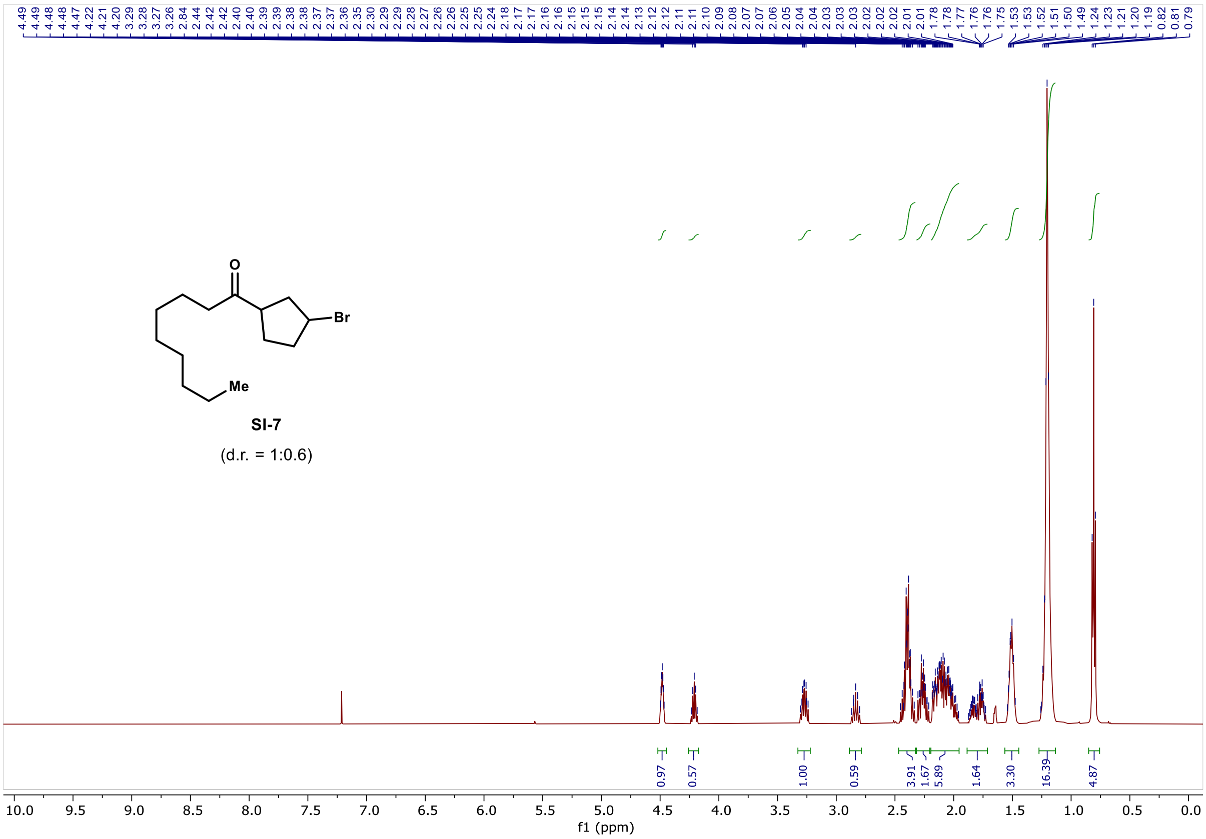

^{13}C NMR (101 MHz, Chloroform-*d*) of **SI-7 – mixture of diastereoisomers**:

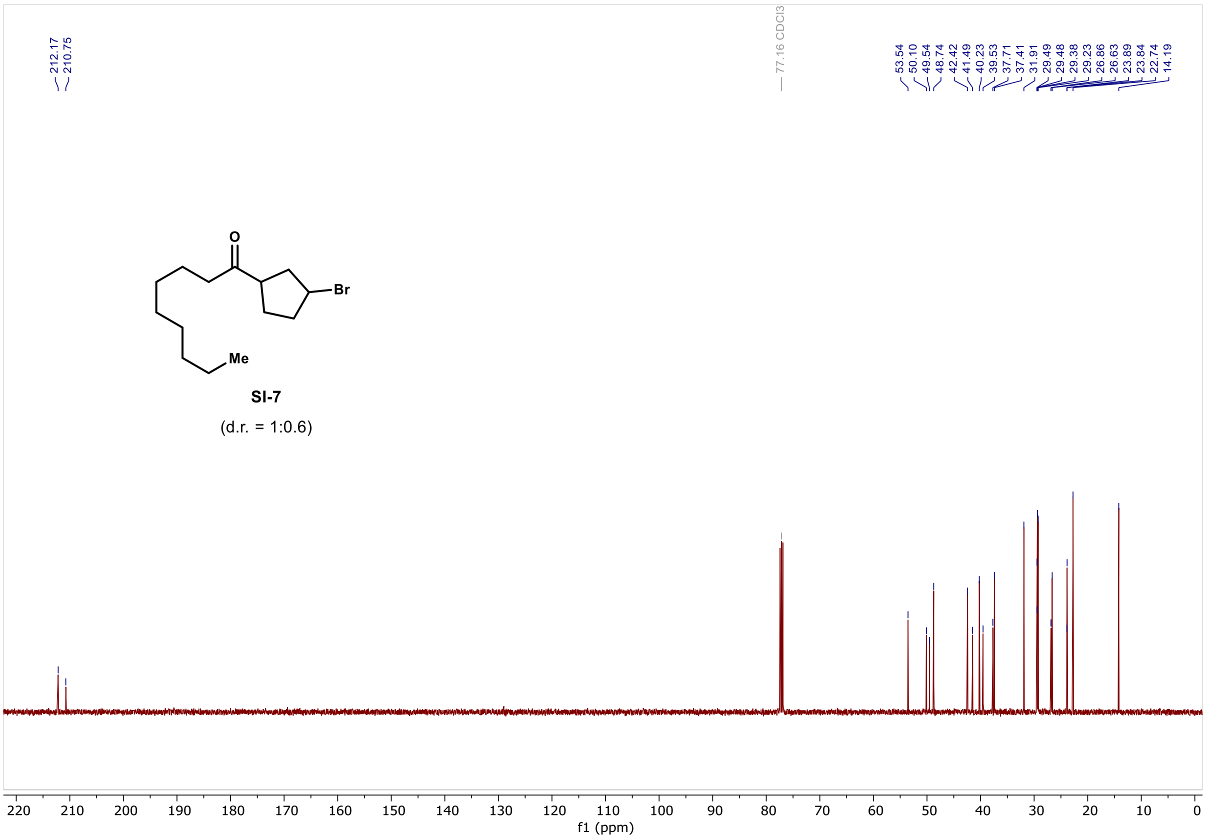

^{1}H NMR (500 MHz, Chloroform-*d*) of **1b**:

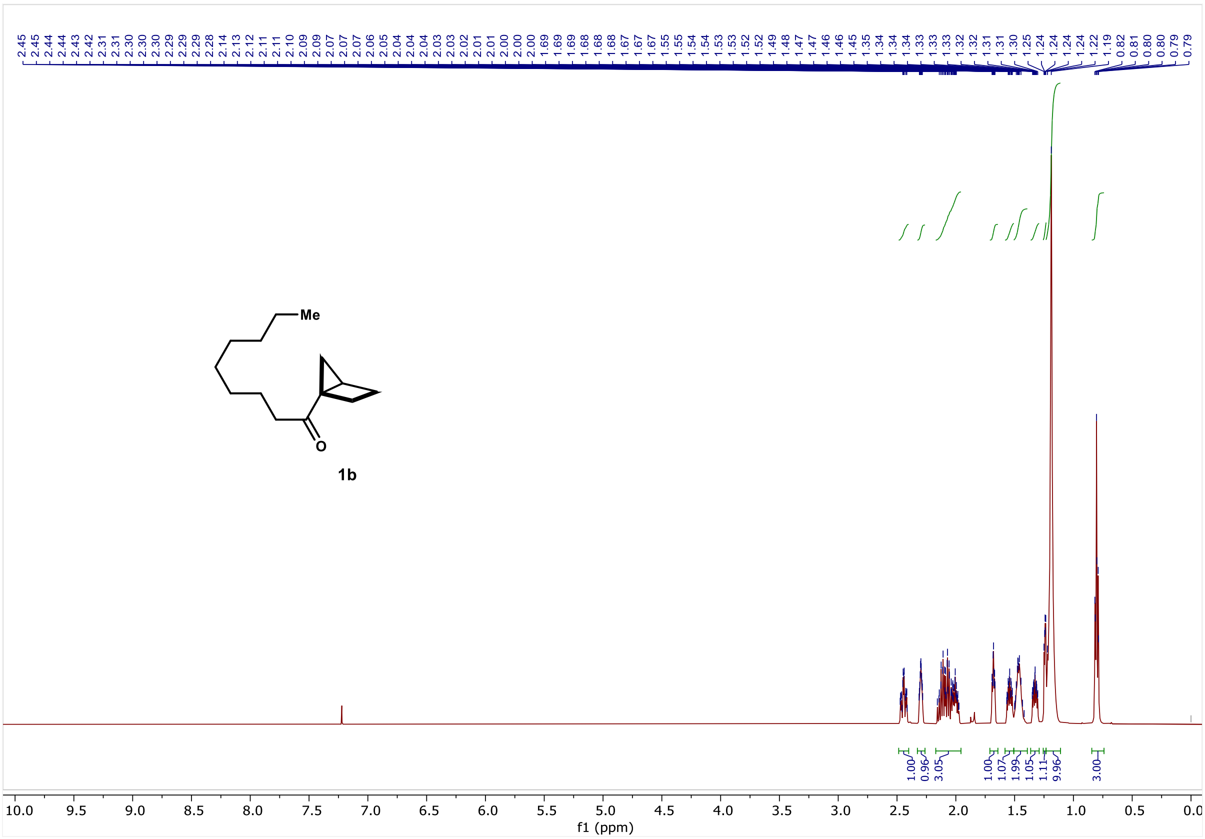

^{13}C NMR (126 MHz, Chloroform-*d*) of **1b**:

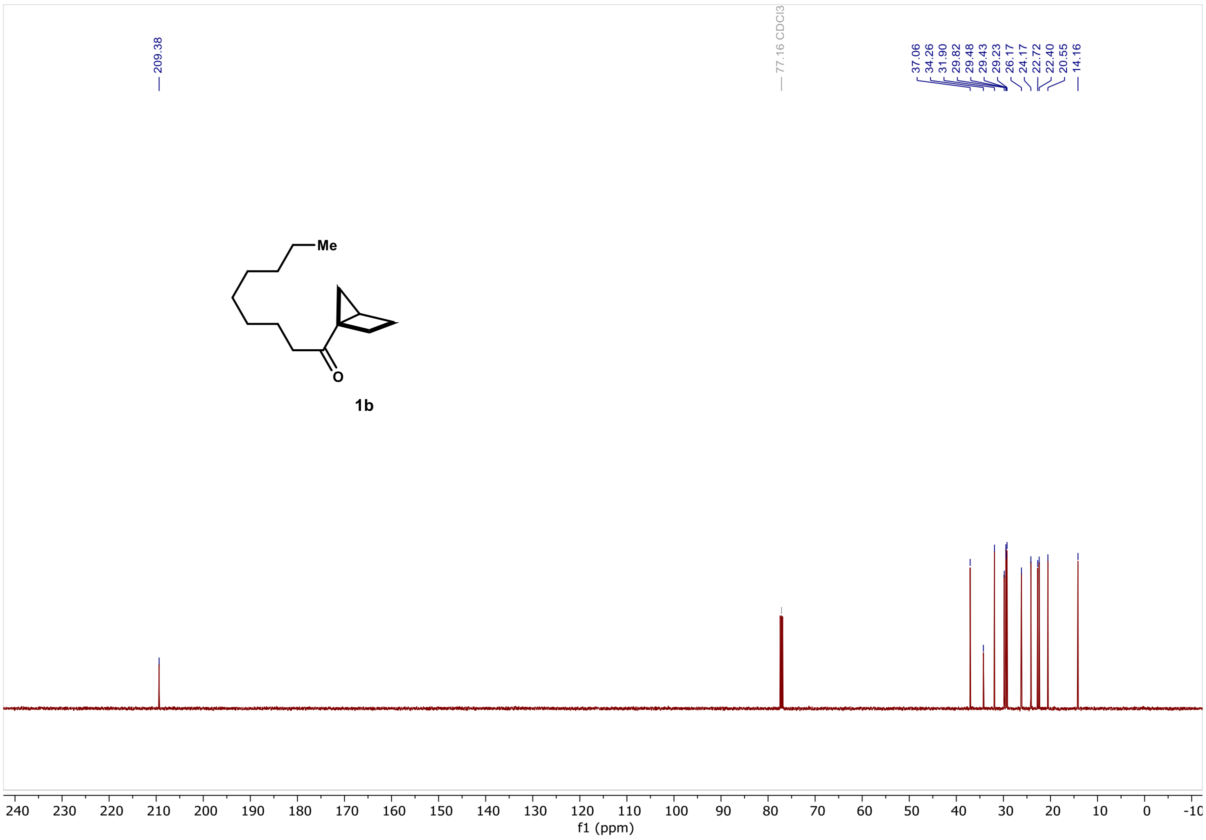

^{1}H NMR (500 MHz, Chloroform-*d*) of **SI-8**:

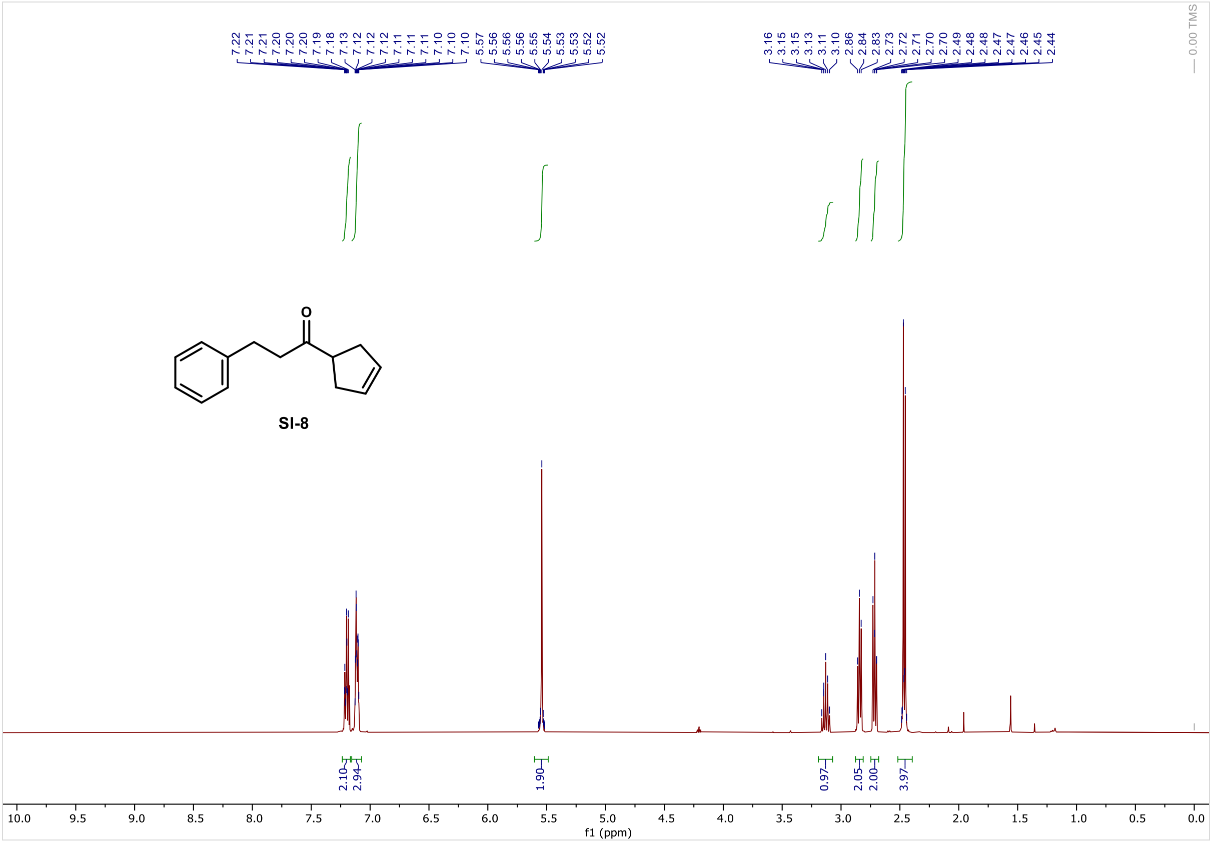

^{13}C NMR (126 MHz, Chloroform-*d*) of **SI-8**:

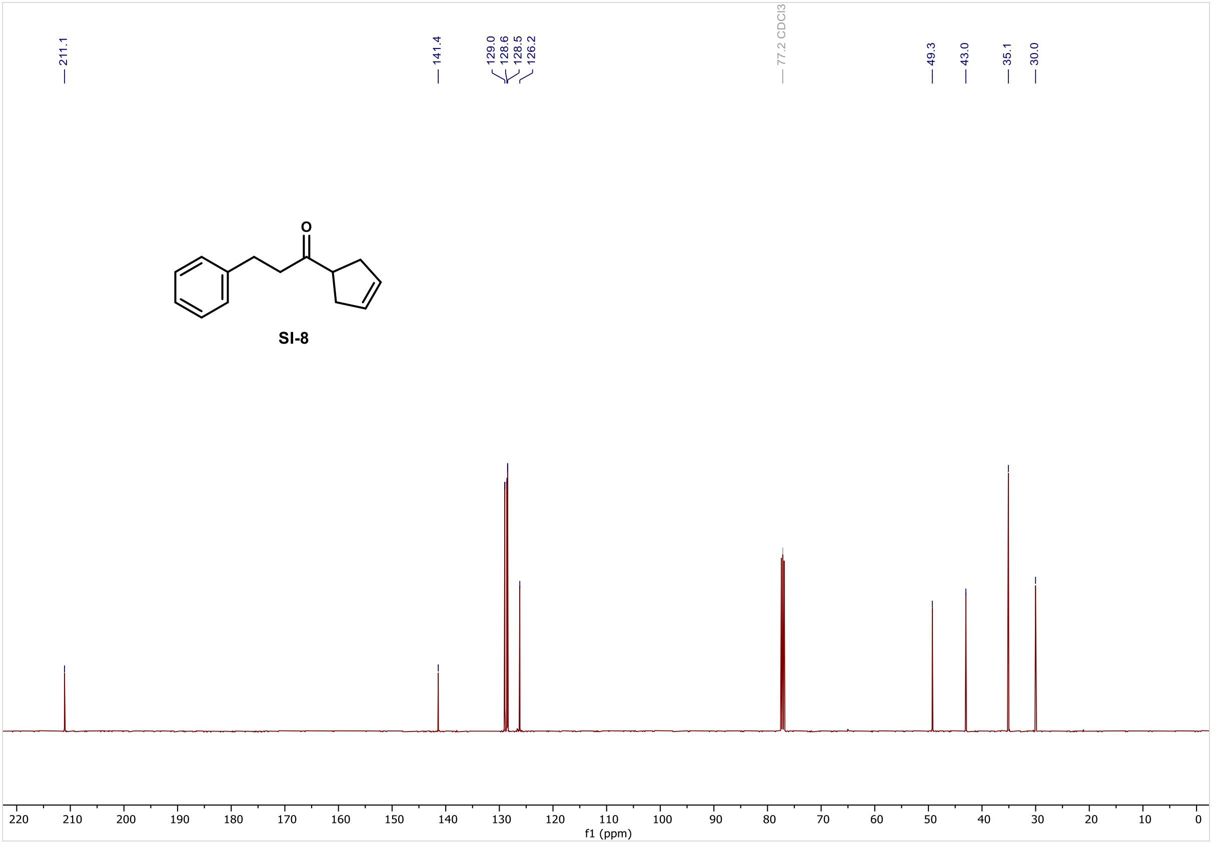

^{1}H NMR (500 MHz, Chloroform-*d*) of of **SI-9 – diastereoisomer 1**:

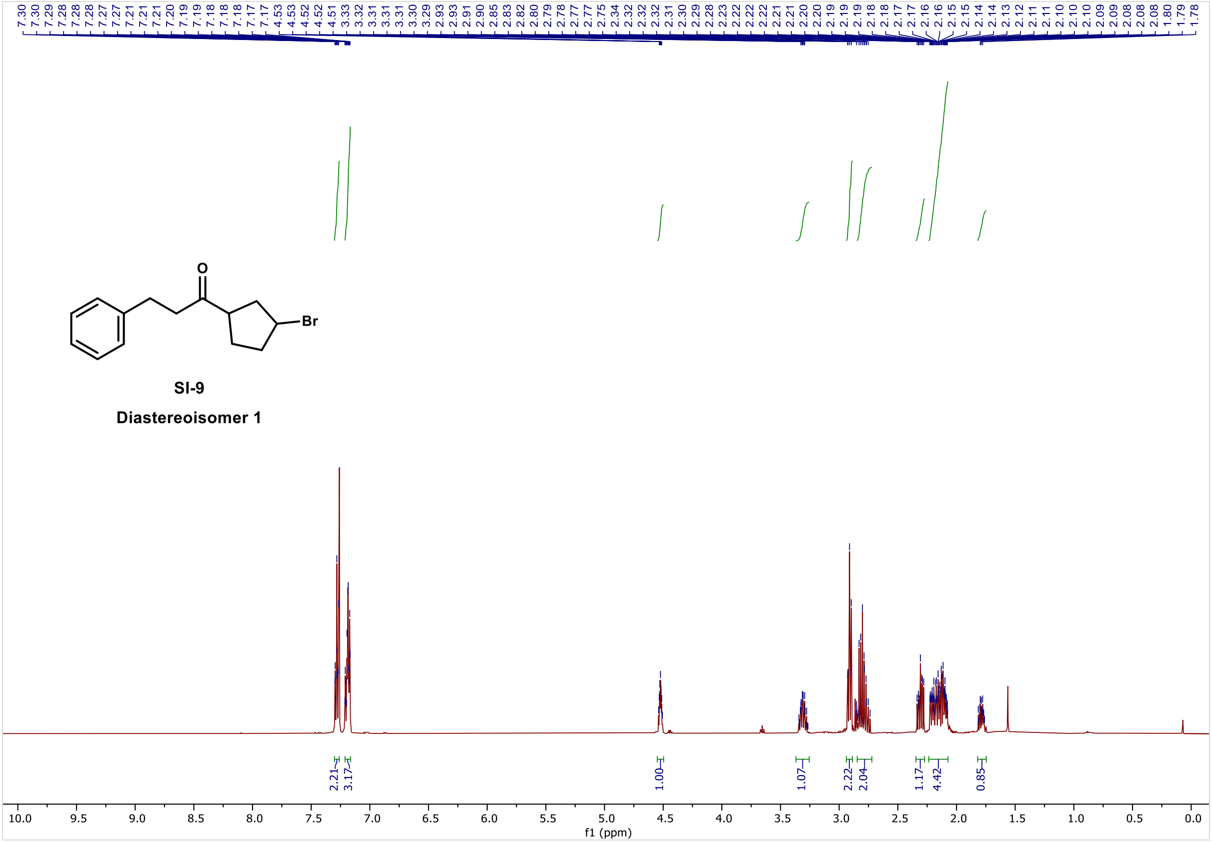

^{13}C NMR (126 MHz, Chloroform-*d*) of **SI-9 – diastereoisomer 1**:

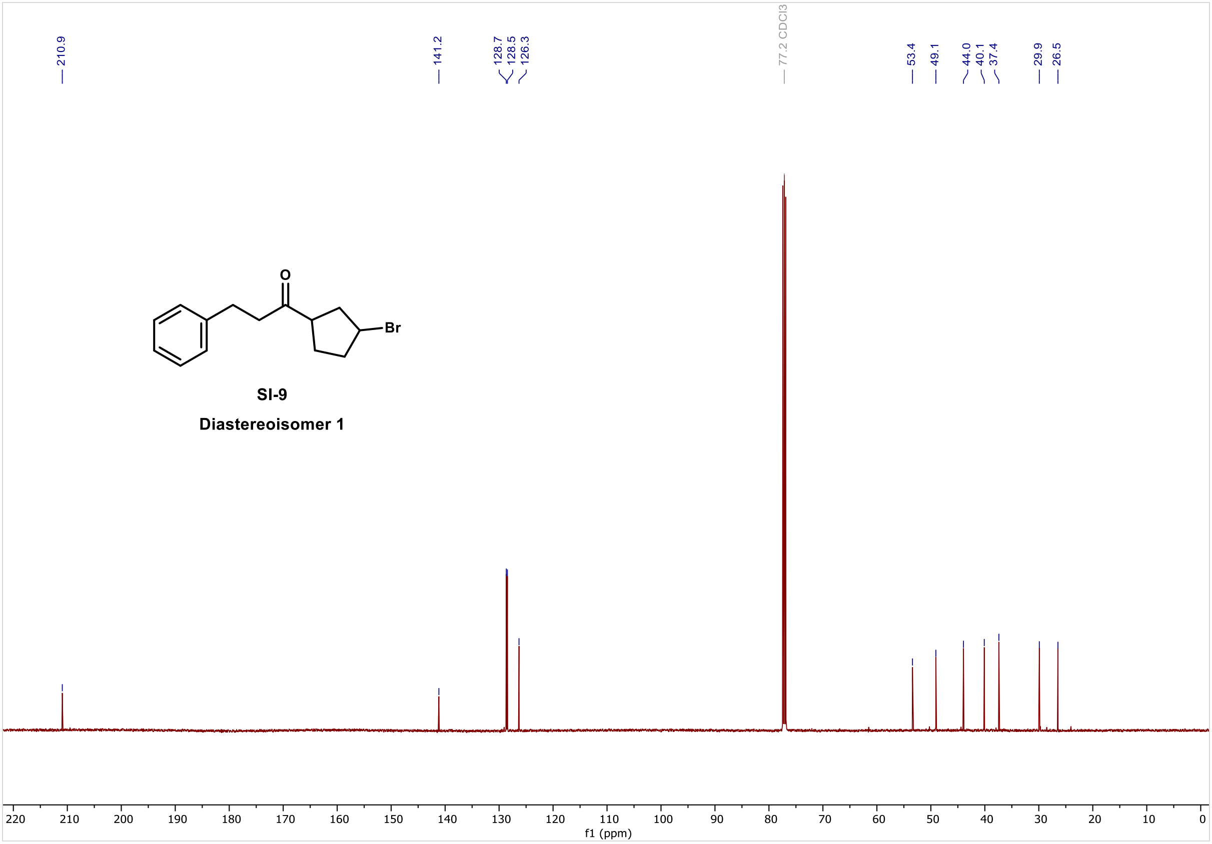

^{1}H NMR (500 MHz, Chloroform-*d*) of **SI-9 – diastereoisomer 2**:

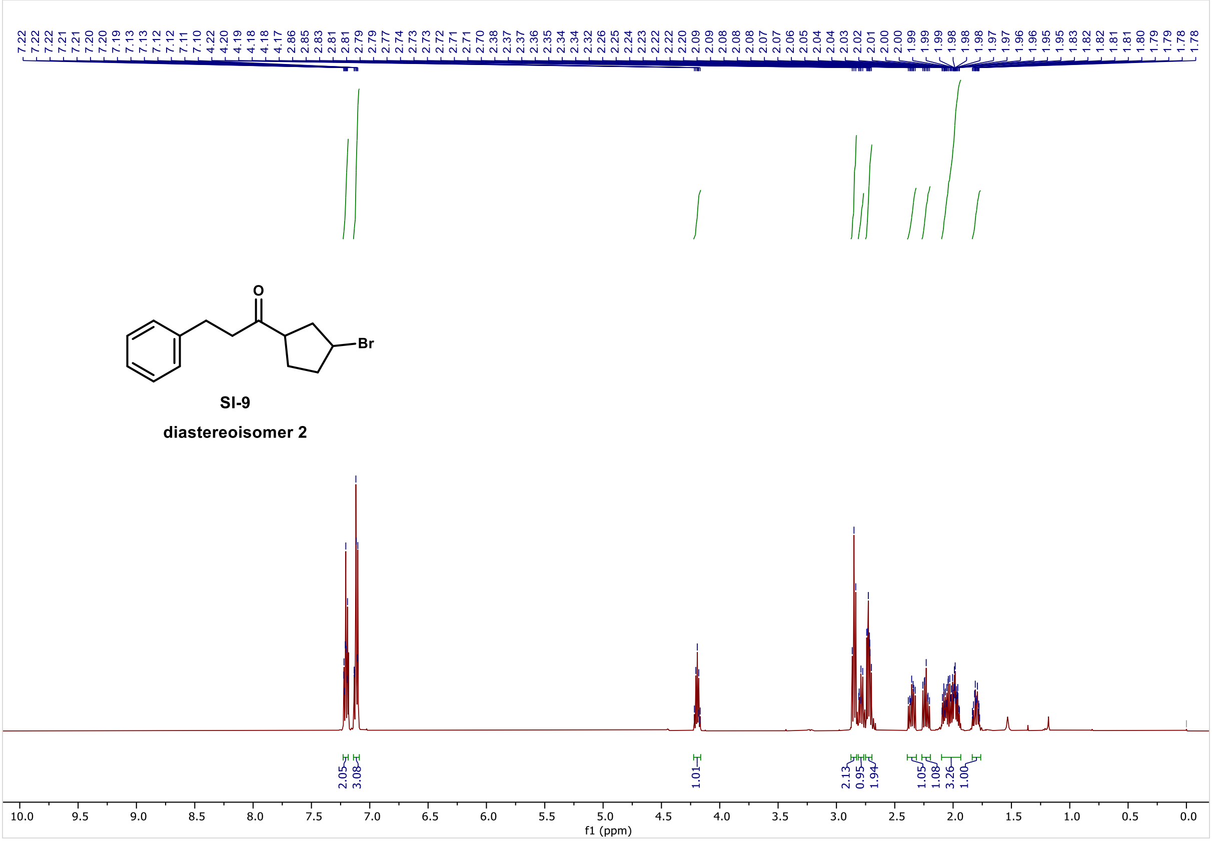

^{13}C NMR (126 MHz, Chloroform-*d*) of **SI-9 – diastereoisomer 2**:

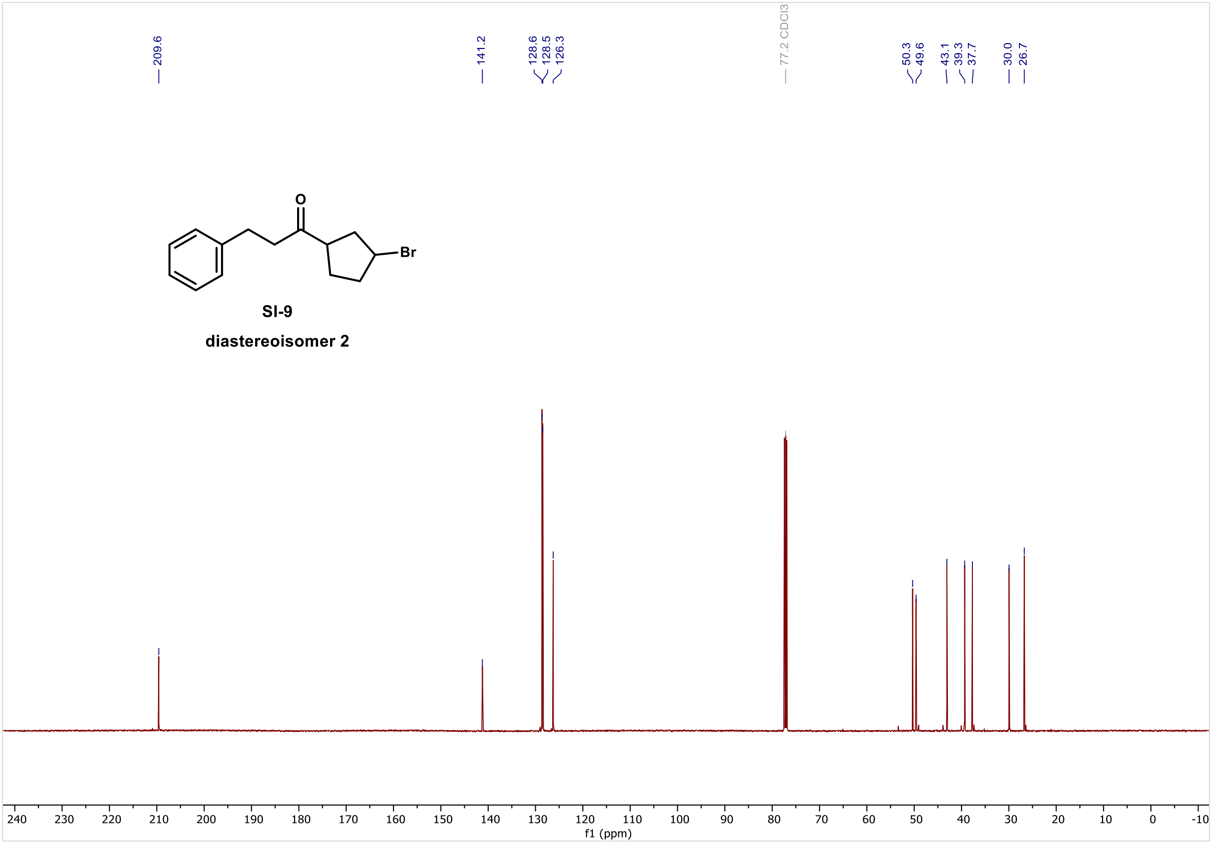

^{1}H NMR (500 MHz, Chloroform-*d*) of **1d**:

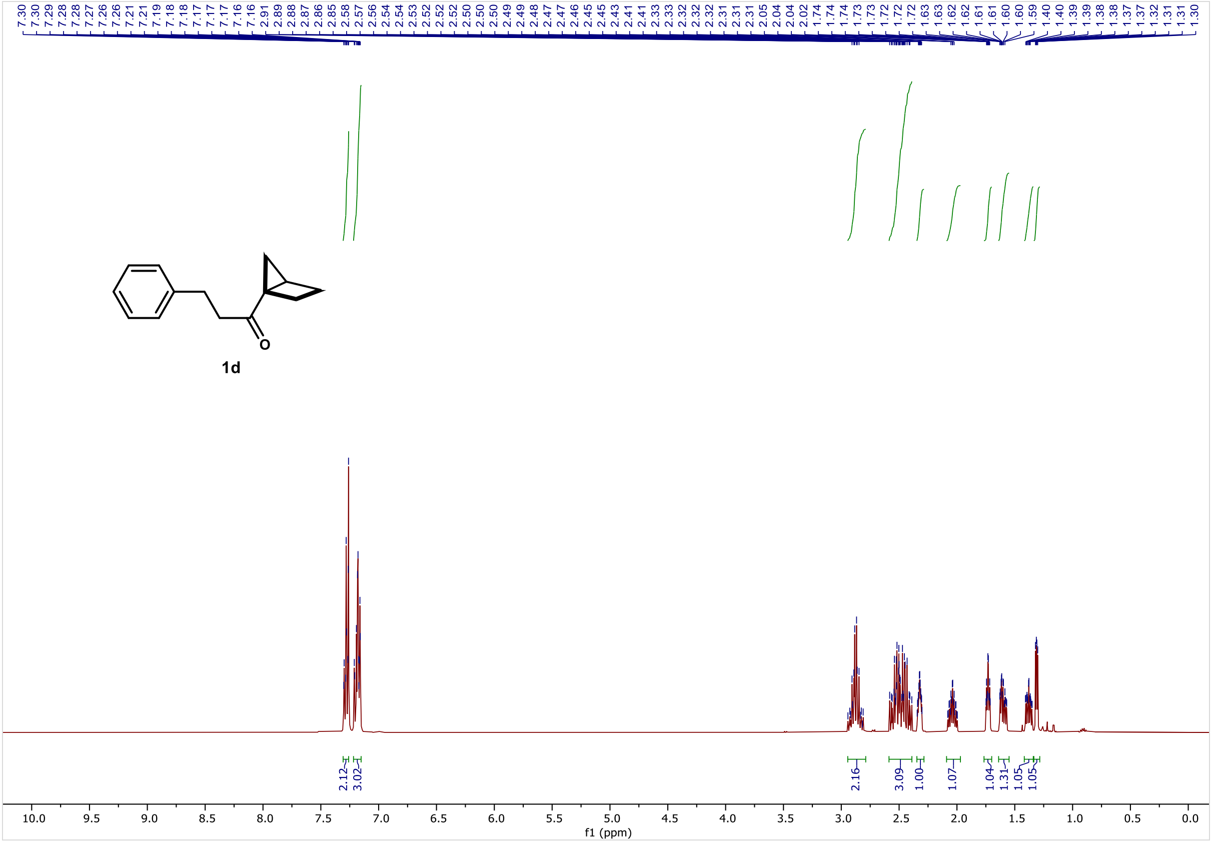

^{13}C NMR (126 MHz, Chloroform-*d*) of **1d**:

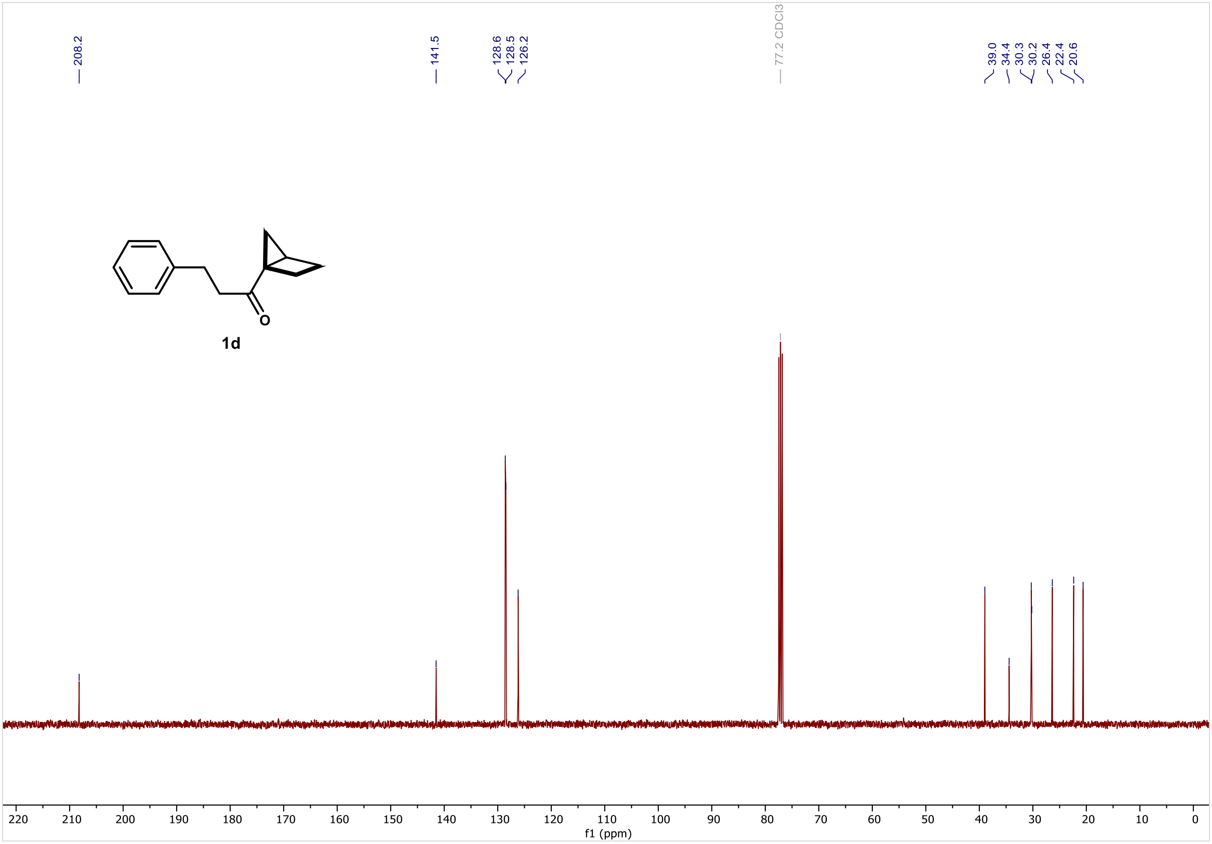

^{1}H NMR (400 MHz, Chloroform-*d*) of **SI-10**:

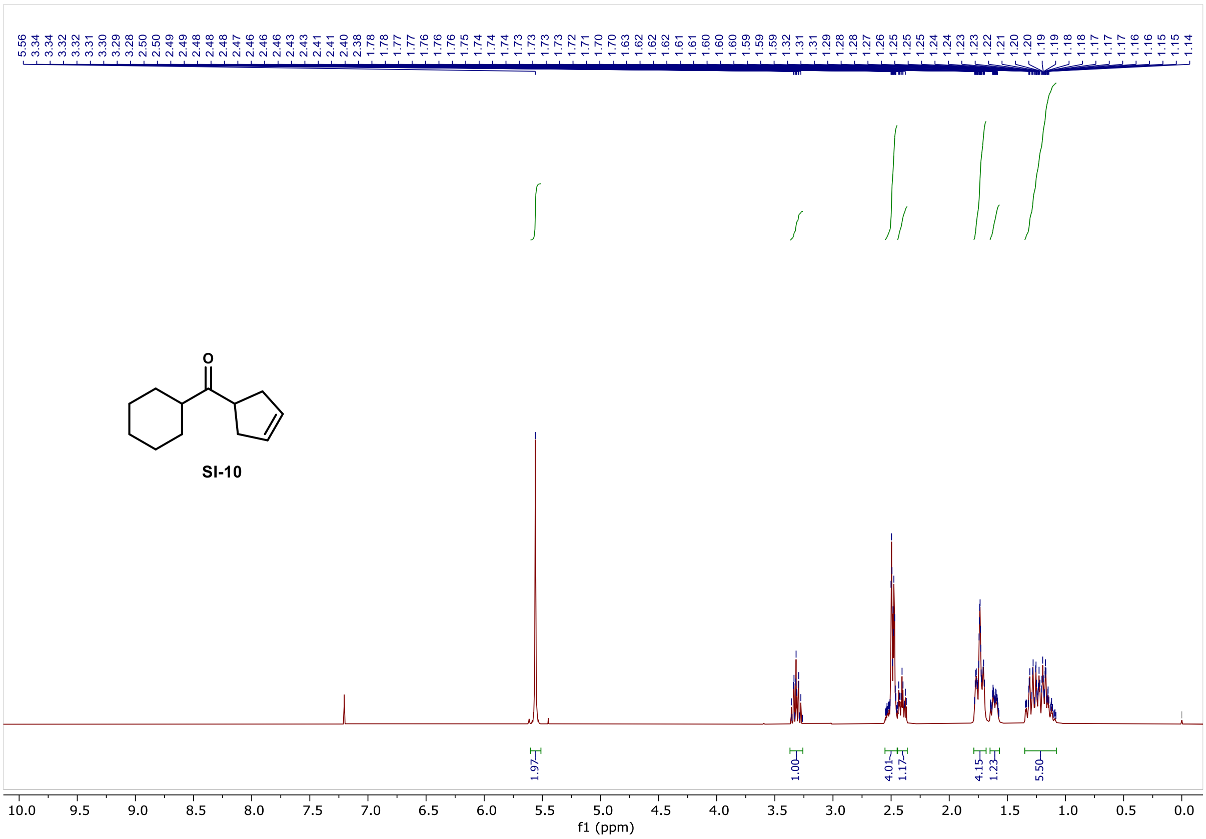

^{13}C NMR (101 MHz, Chloroform-*d*) of **SI-10**:

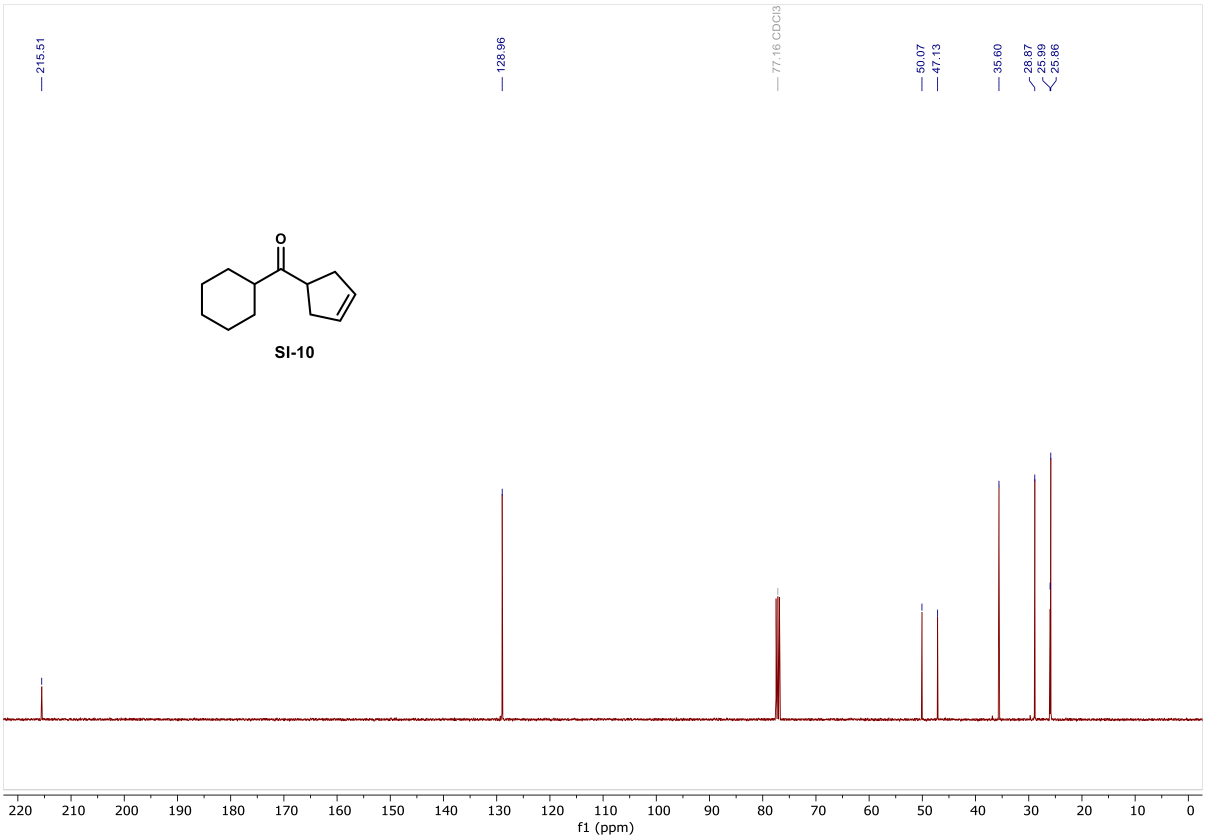

^{1}H NMR (400 MHz, Chloroform-*d*) of **SI-11 – diastereoisomer 1**:

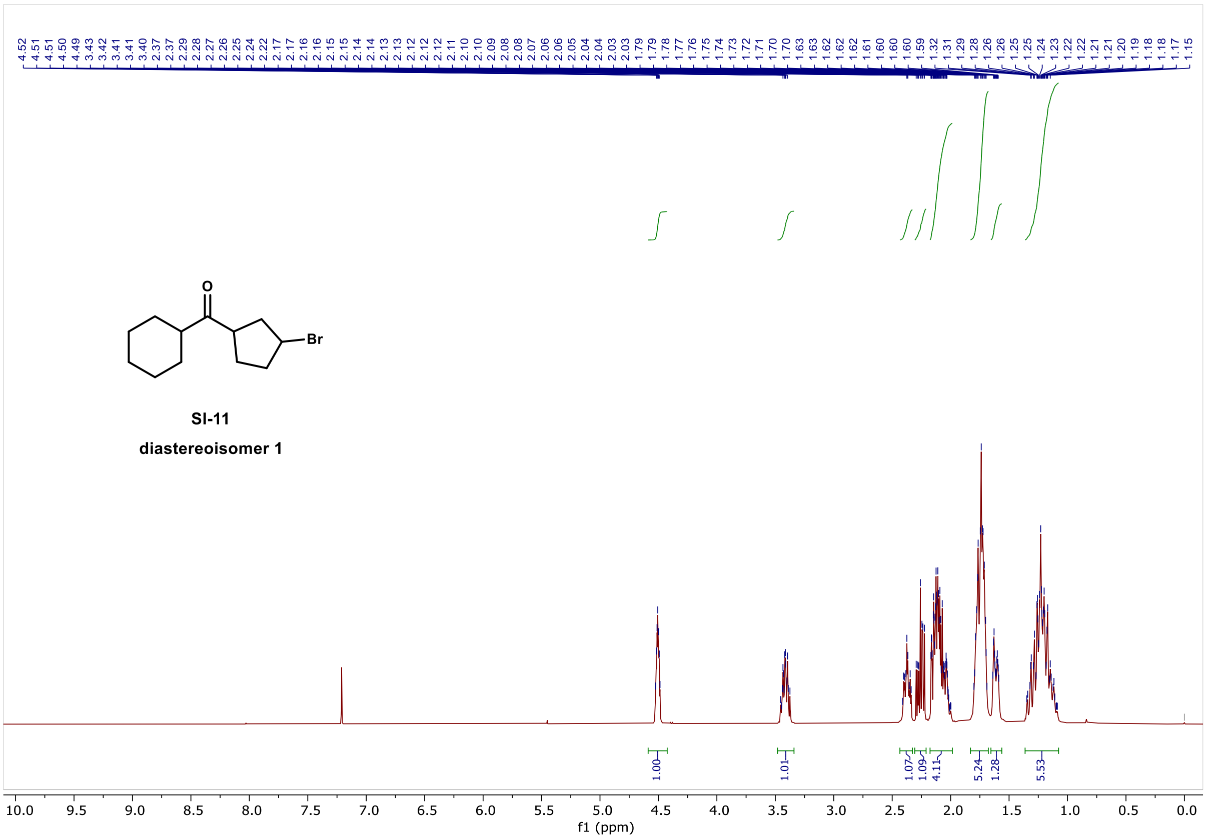

^{13}C NMR (101 MHz, Chloroform-*d*) of **SI-11 – diastereoisomer 1**:

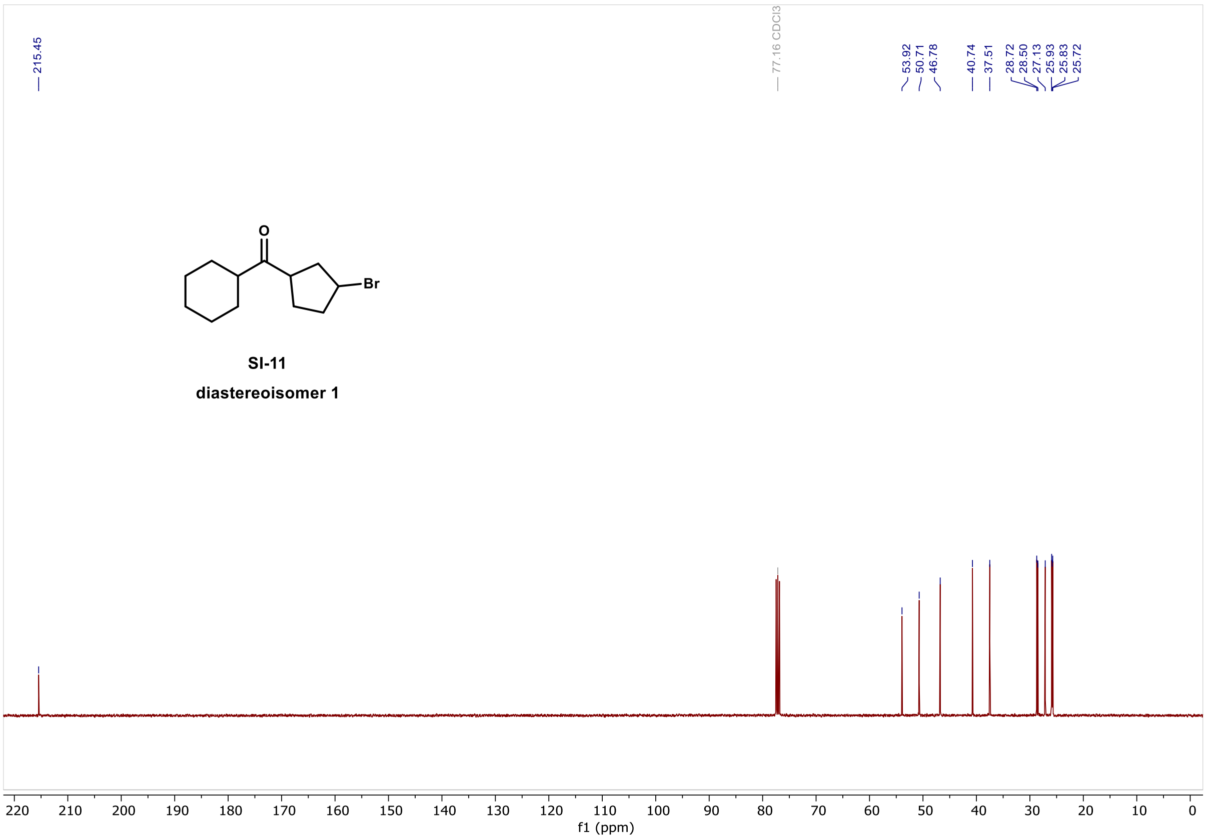

^{1}H NMR (400 MHz, Chloroform-*d*) of **SI-11 – diastereoisomer 2**:

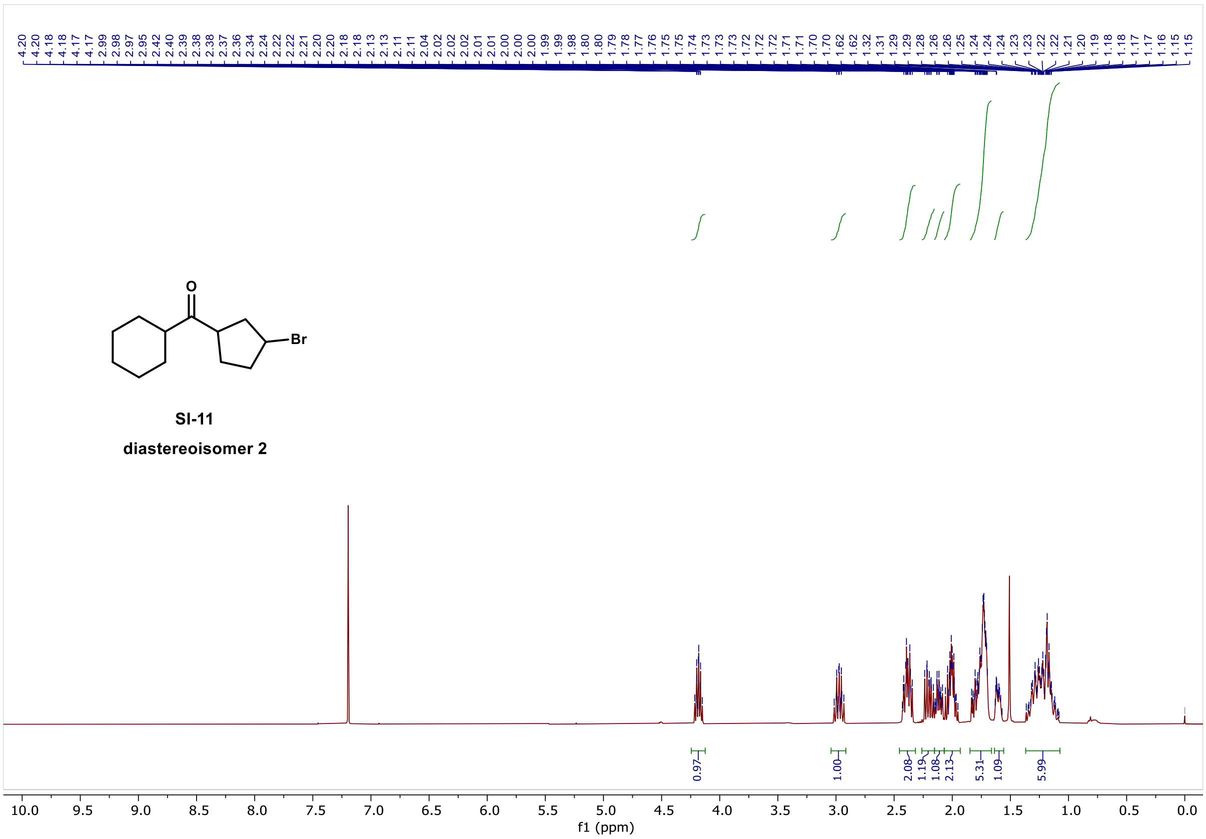

^{13}C NMR (101 MHz, Chloroform-*d*) of **SI-11 – diastereoisomer 2**:

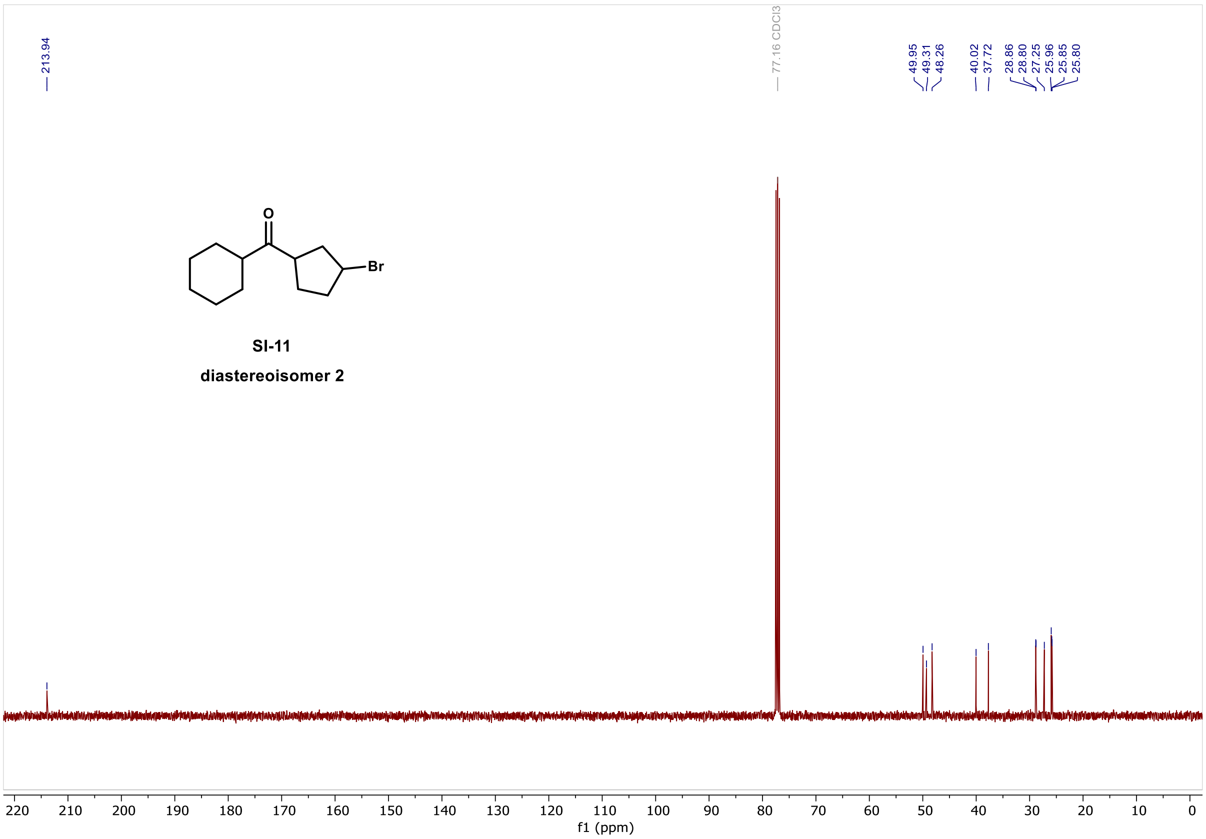

^{1}H NMR (400 MHz, Chloroform-*d*) of **1j**:

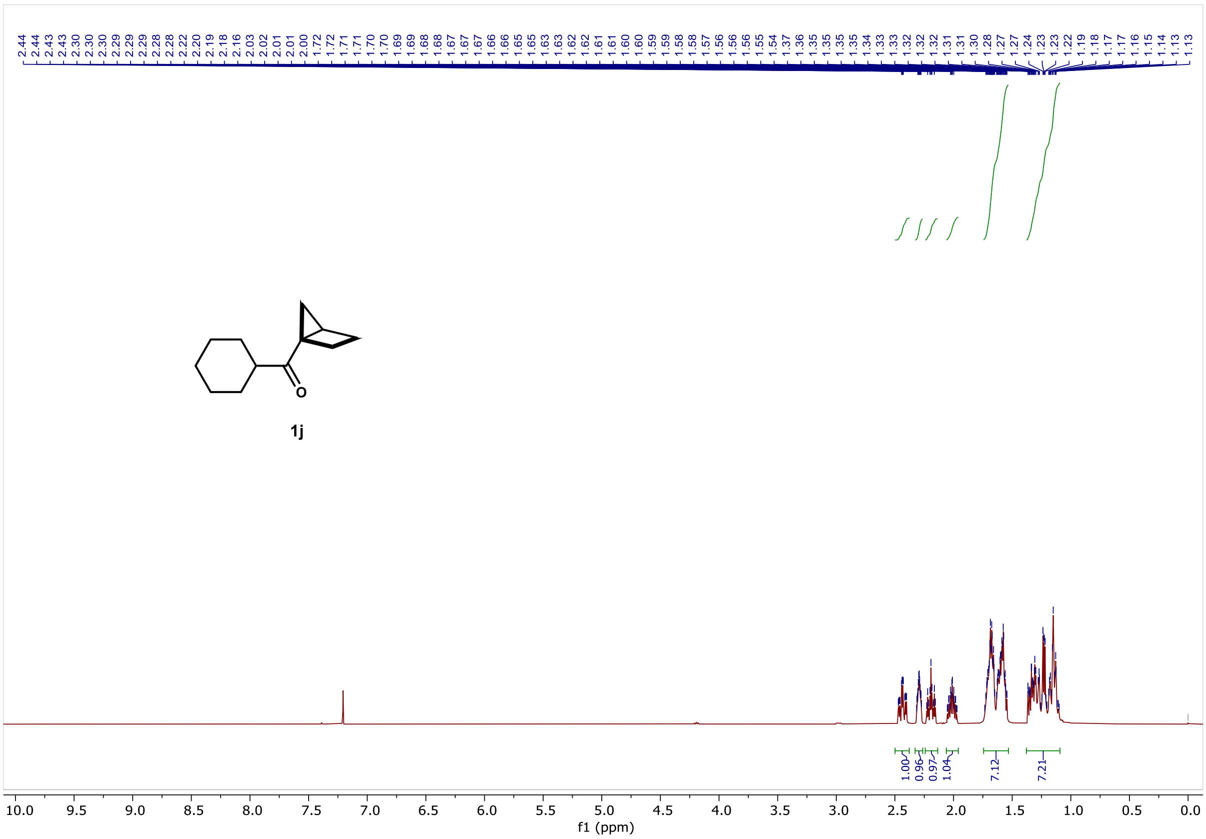

^{13}C NMR (101 MHz, Chloroform-*d*) of **1j**:

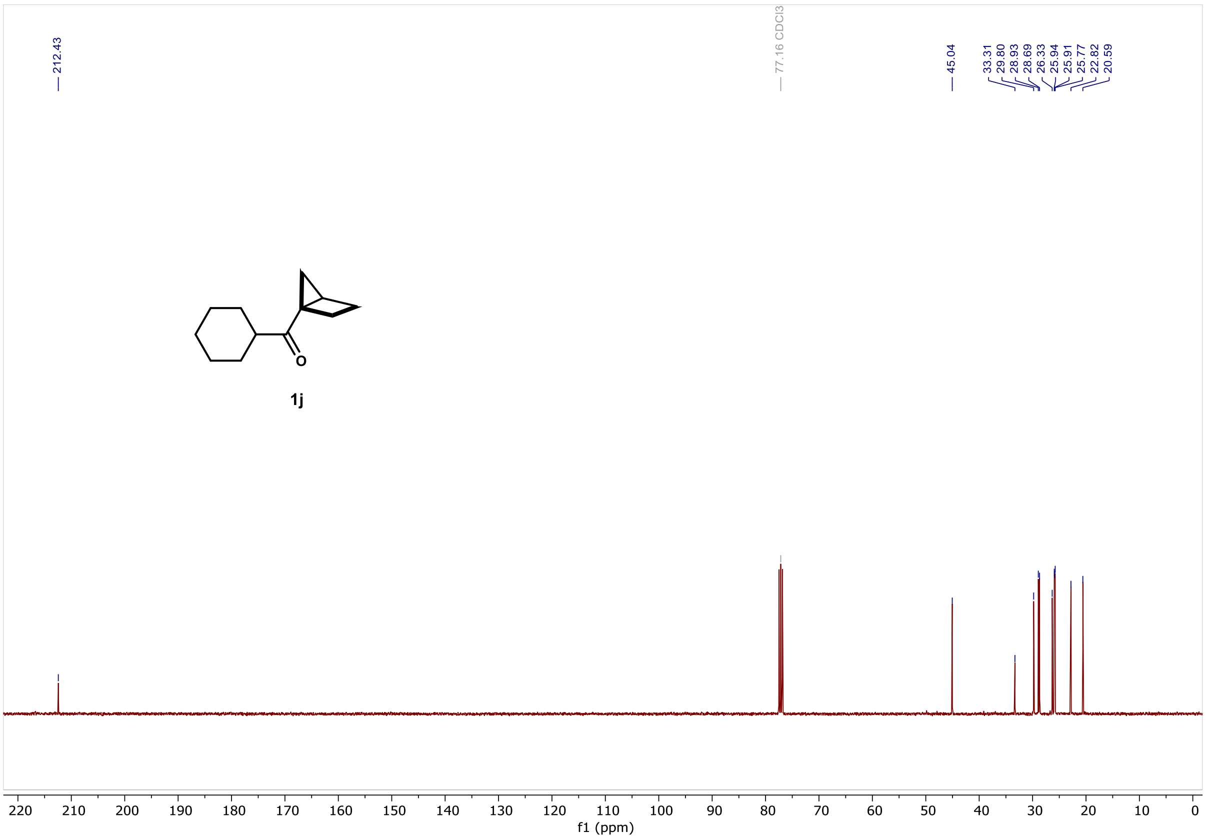

^{1}H NMR (400 MHz, Chloroform-*d*) of **SI-12**:

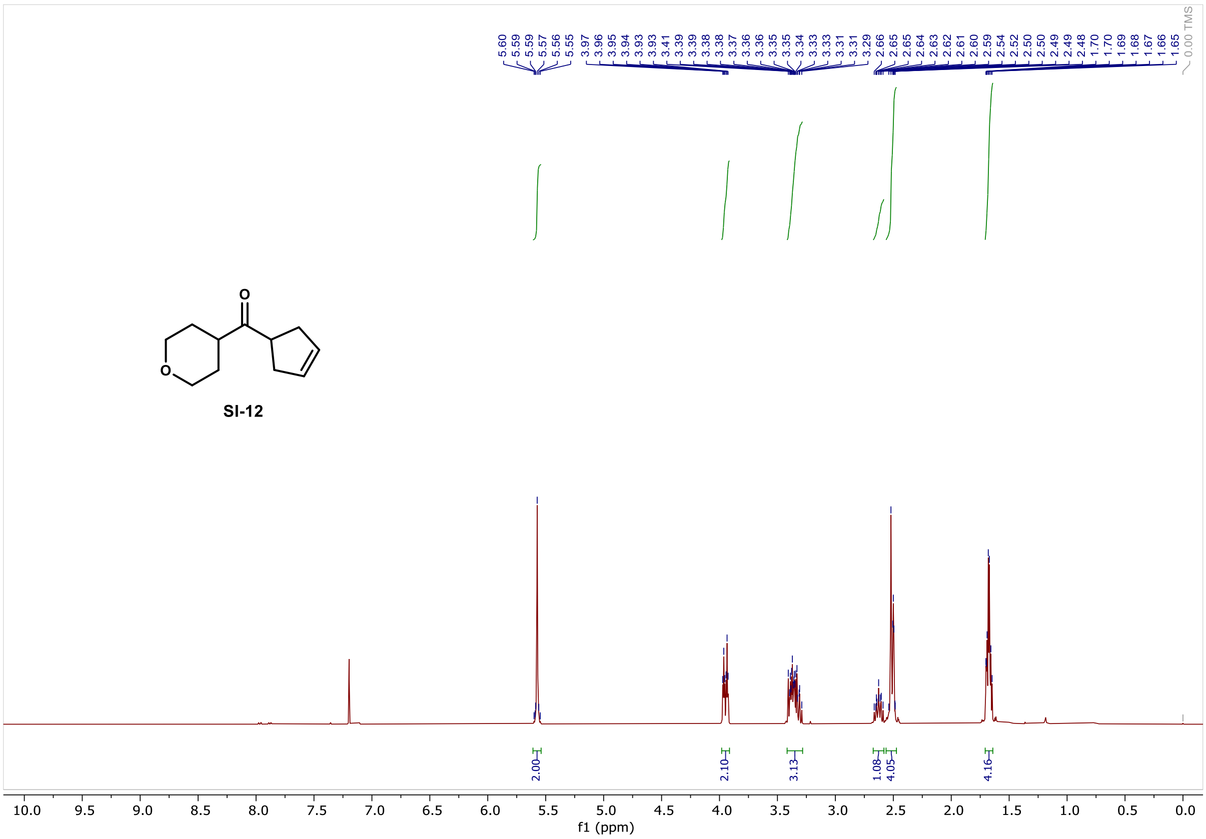

^{13}C NMR (101 MHz, Chloroform-*d*) of **SI-12**:

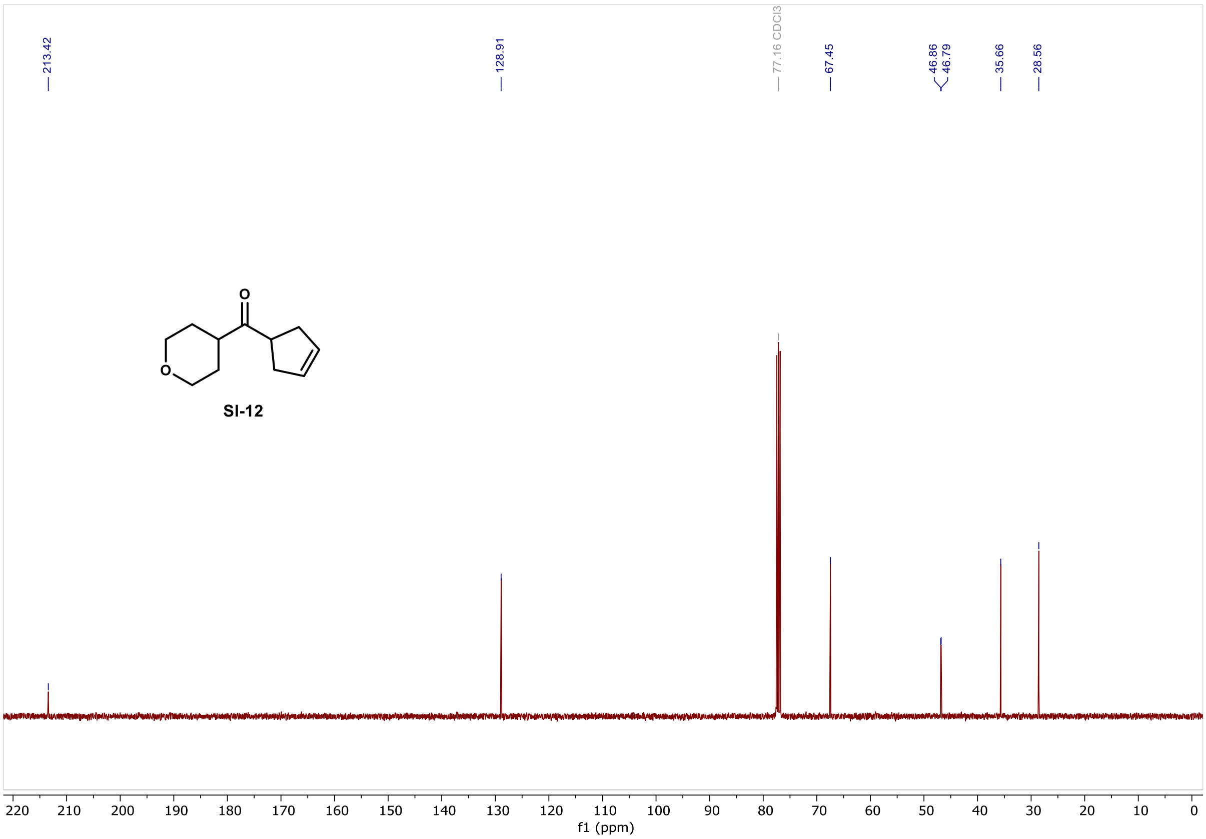

^{1}H NMR (400 MHz, Chloroform-*d*) of **SI-13 – diastereoisomer 1**:

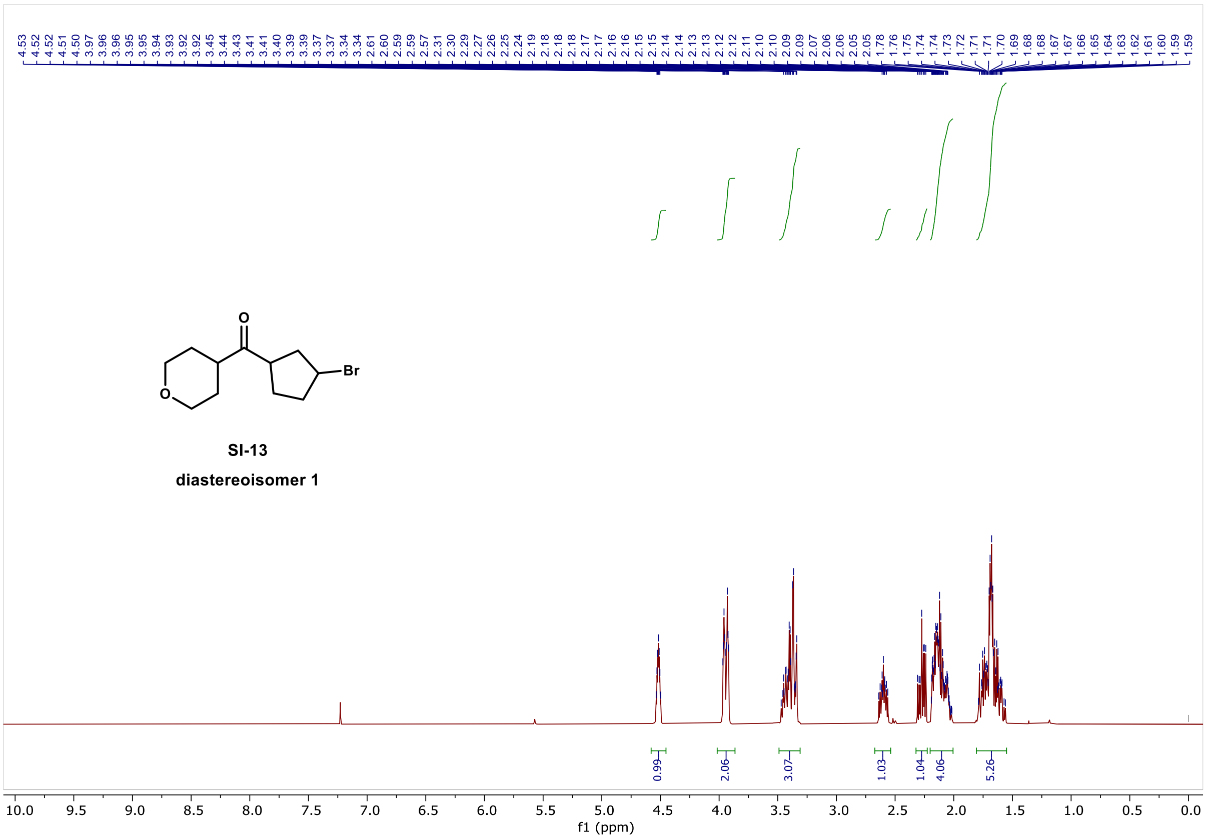

^{13}C NMR (101 MHz, Chloroform-*d*) of **SI-13 – diastereoisomer 1**:

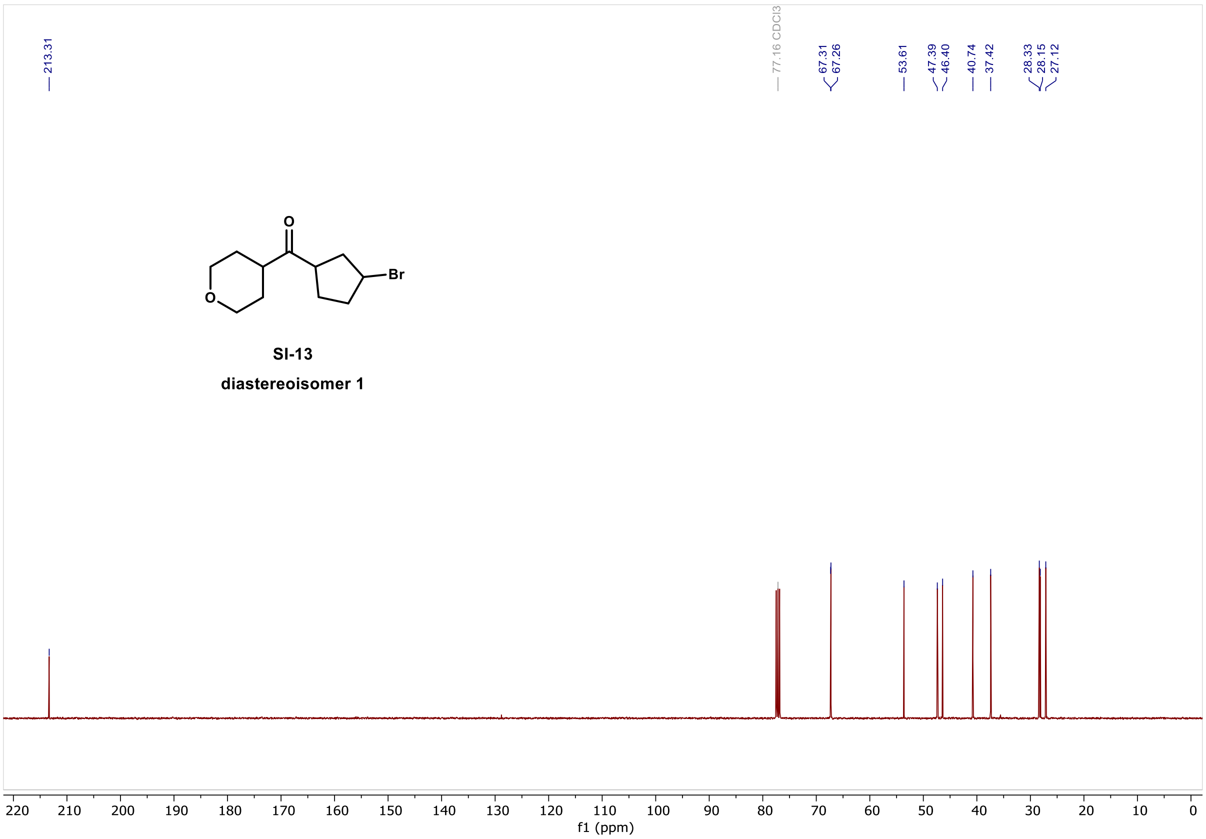

^{1}H NMR (400 MHz, Chloroform-*d*) of **SI-13 – diastereoisomer 2**:

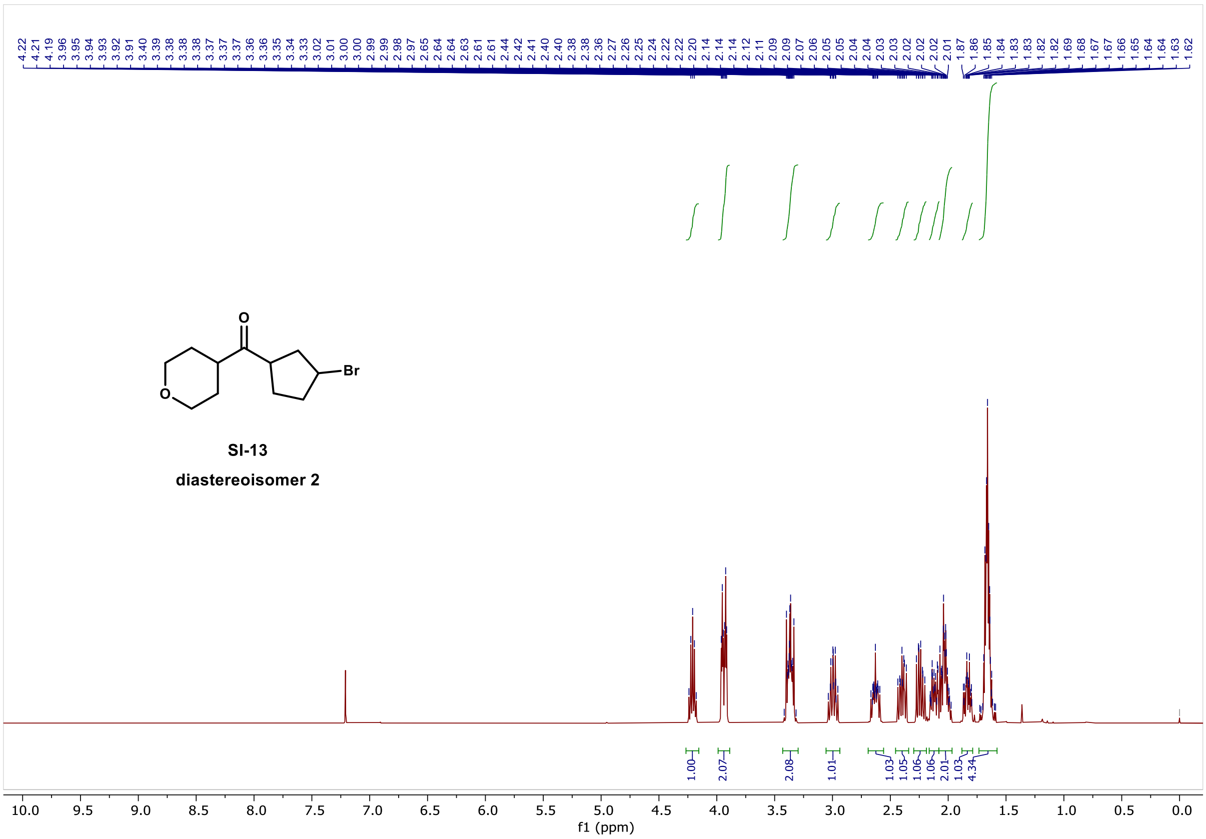

^{13}C NMR (101 MHz, Chloroform-*d*) of **SI-13 – diastereoisomer 2**:

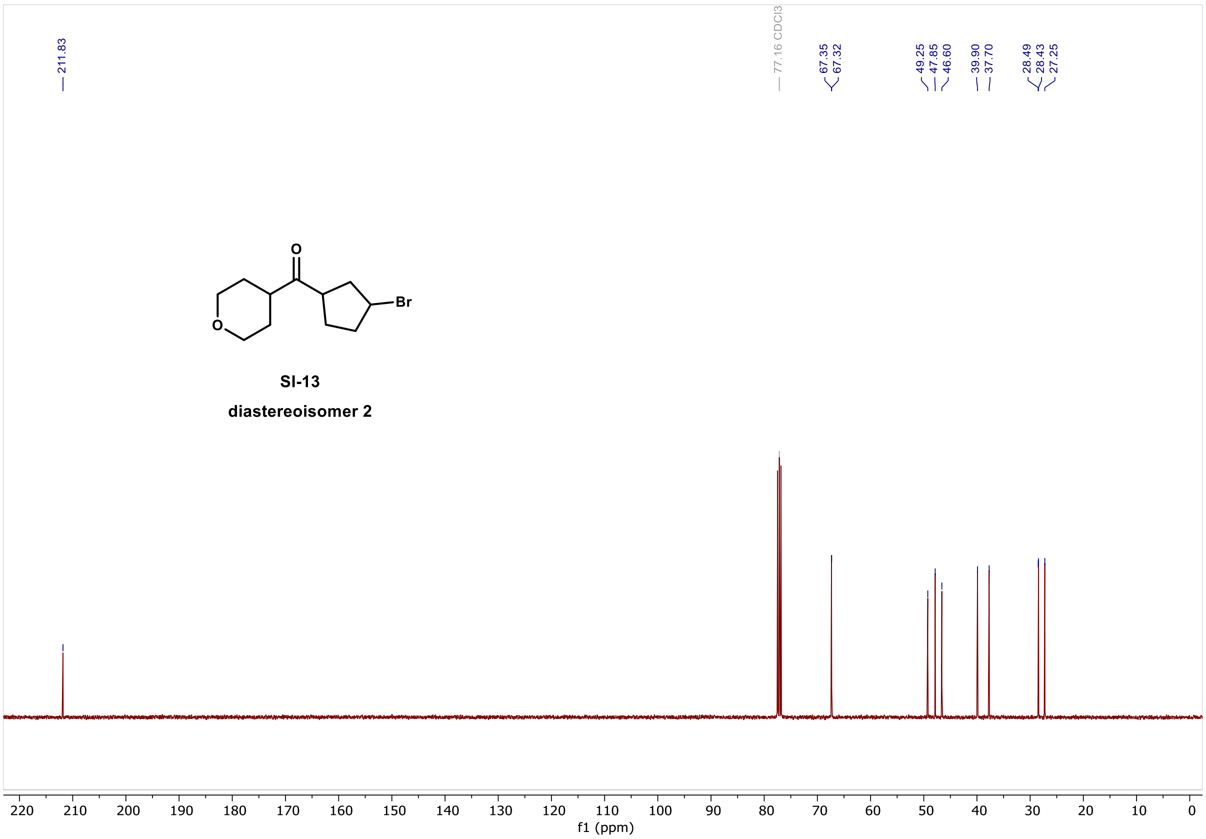

^{1}H NMR (400 MHz, Chloroform-*d*) of **1l**:

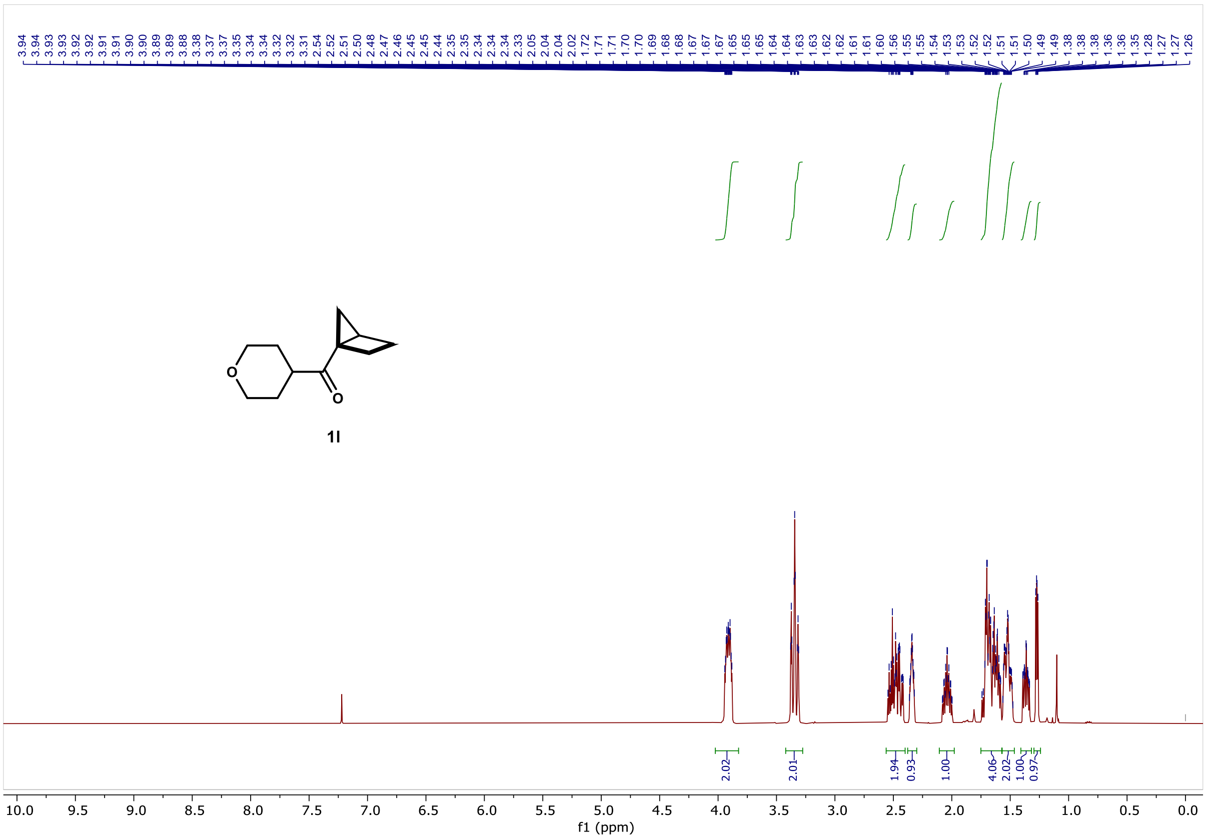

^{13}C NMR (101 MHz, Chloroform-*d*) of **1l**:

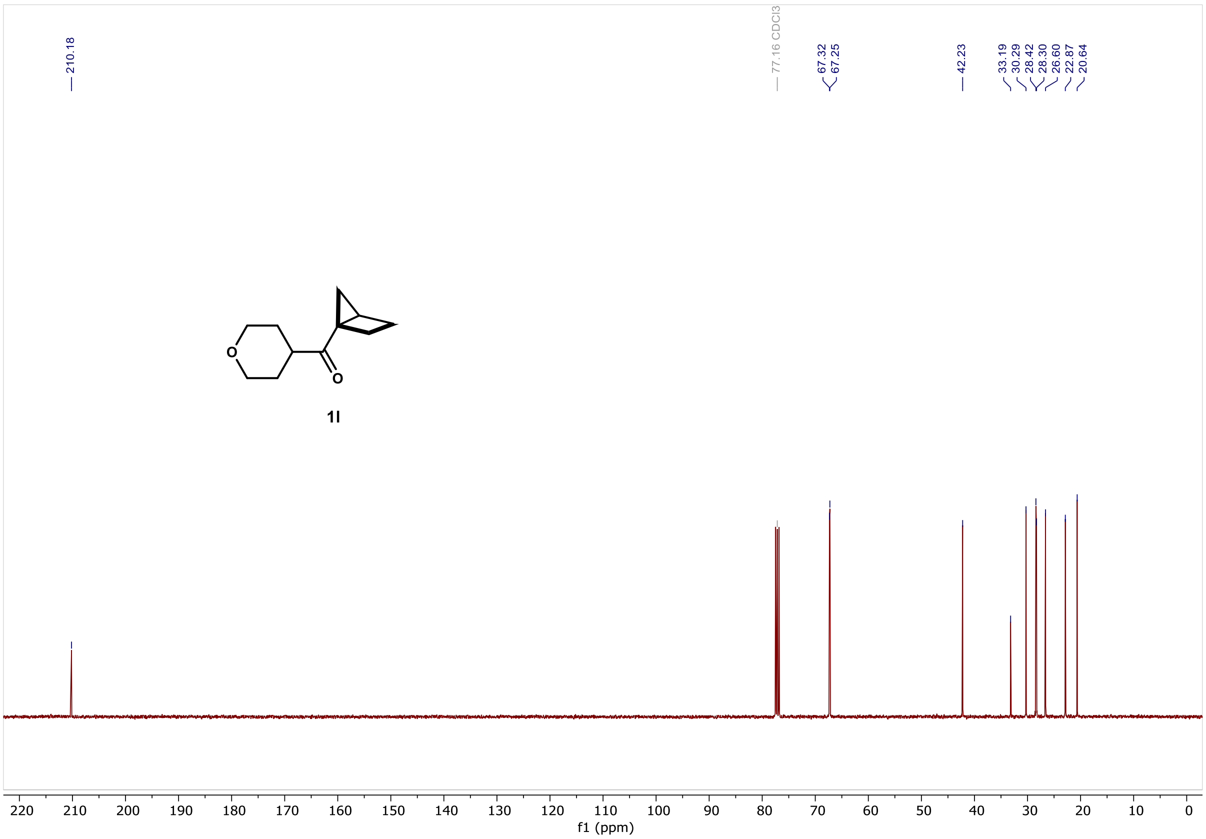

^{1}H NMR (500 MHz, Chloroform-*d*) of **SI-14**:

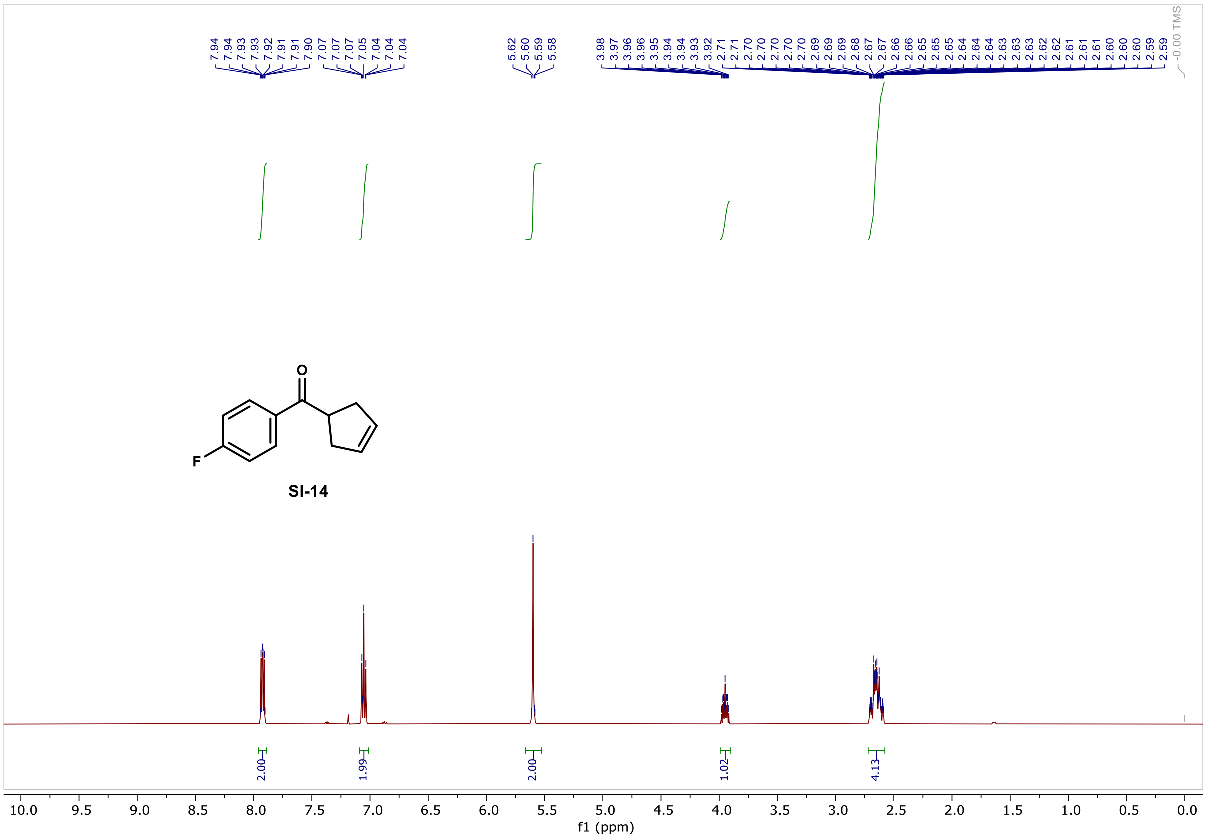

^{13}C NMR (126 MHz, Chloroform-*d*) of **SI-14**:

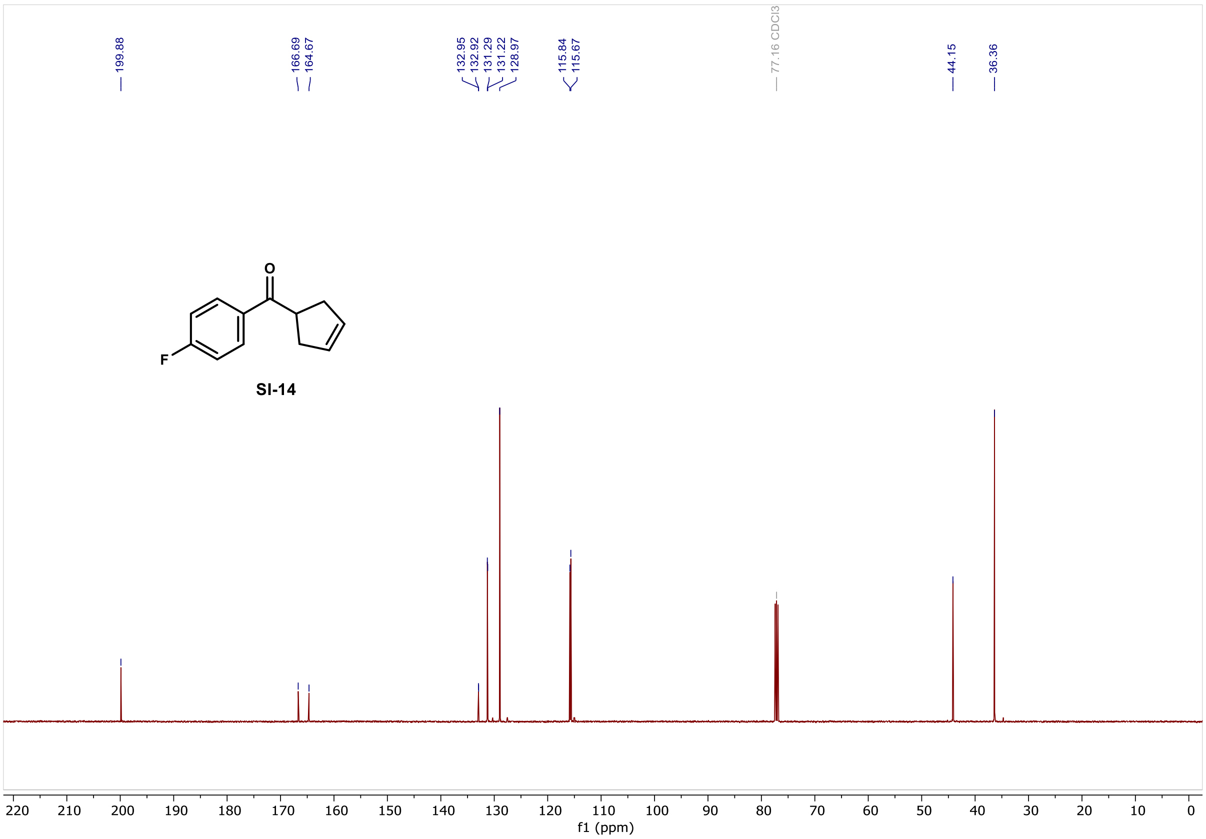

^{19}F NMR (376 MHz, Chloroform-*d*) of **SI-14**:

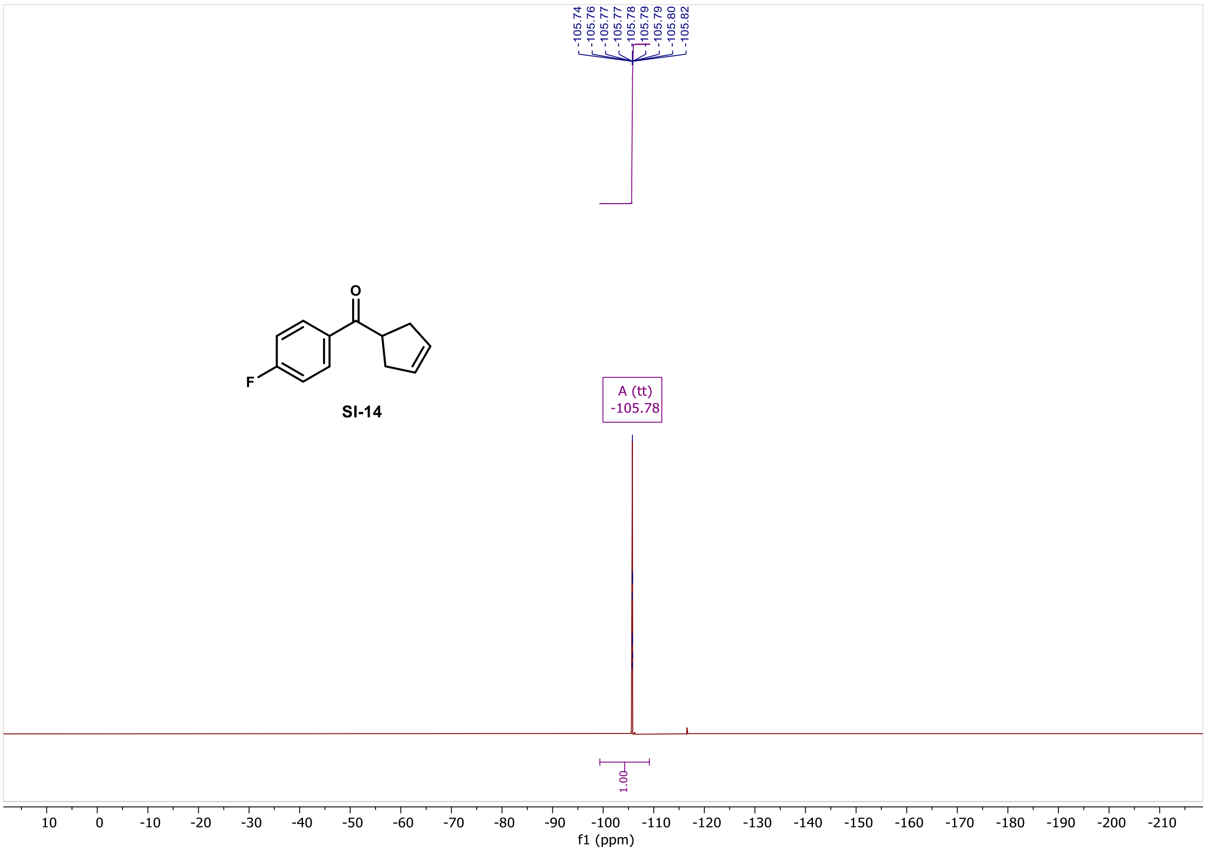

^{1}H NMR (500 MHz, Chloroform-*d*) of **SI-15 – diastereoisomer 1**:

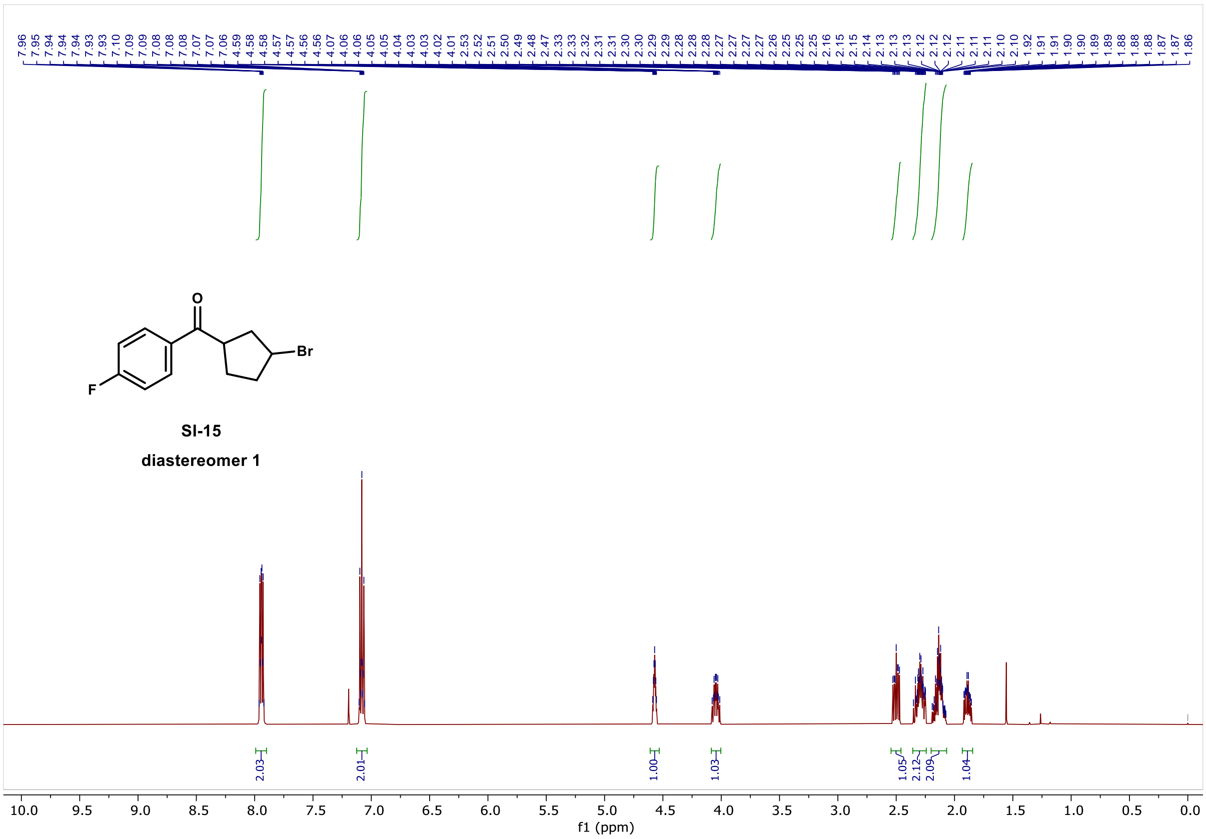

^{13}C NMR (126 MHz, Chloroform-*d*) of **SI-15 – diastereoisomer 1**:

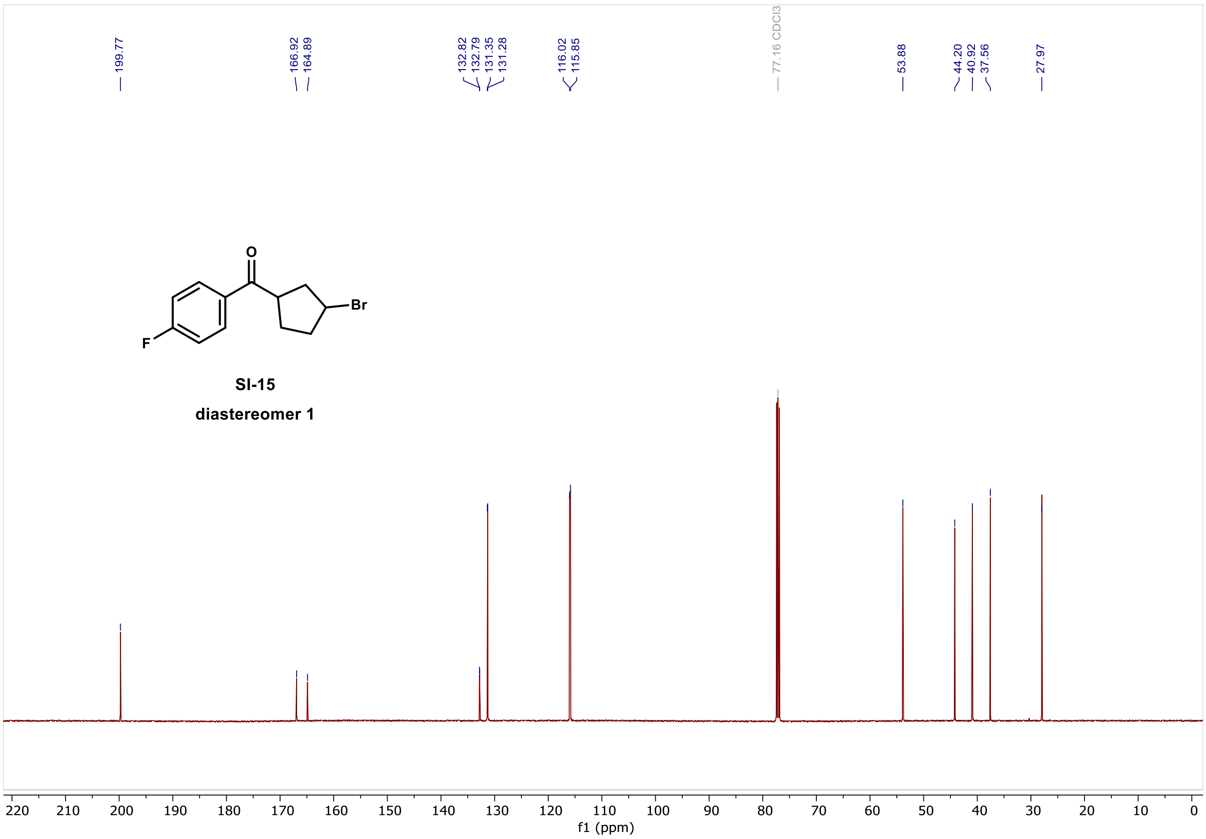

^{19}F NMR (376 MHz, Chloroform-*d*) of **SI-15 – diastereoisomer 1**:

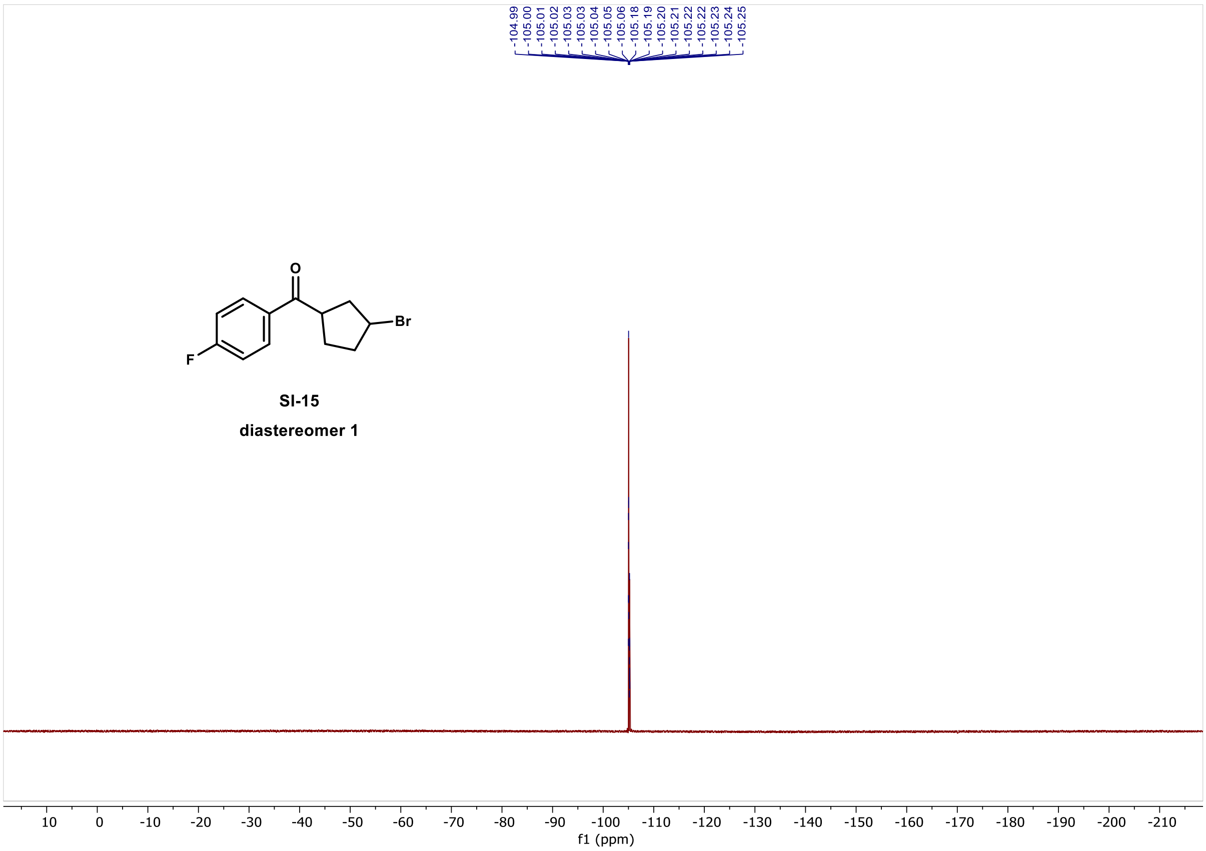

^{1}H NMR (500 MHz, Chloroform-*d*) of **SI-15 – diastereoisomer 2**:

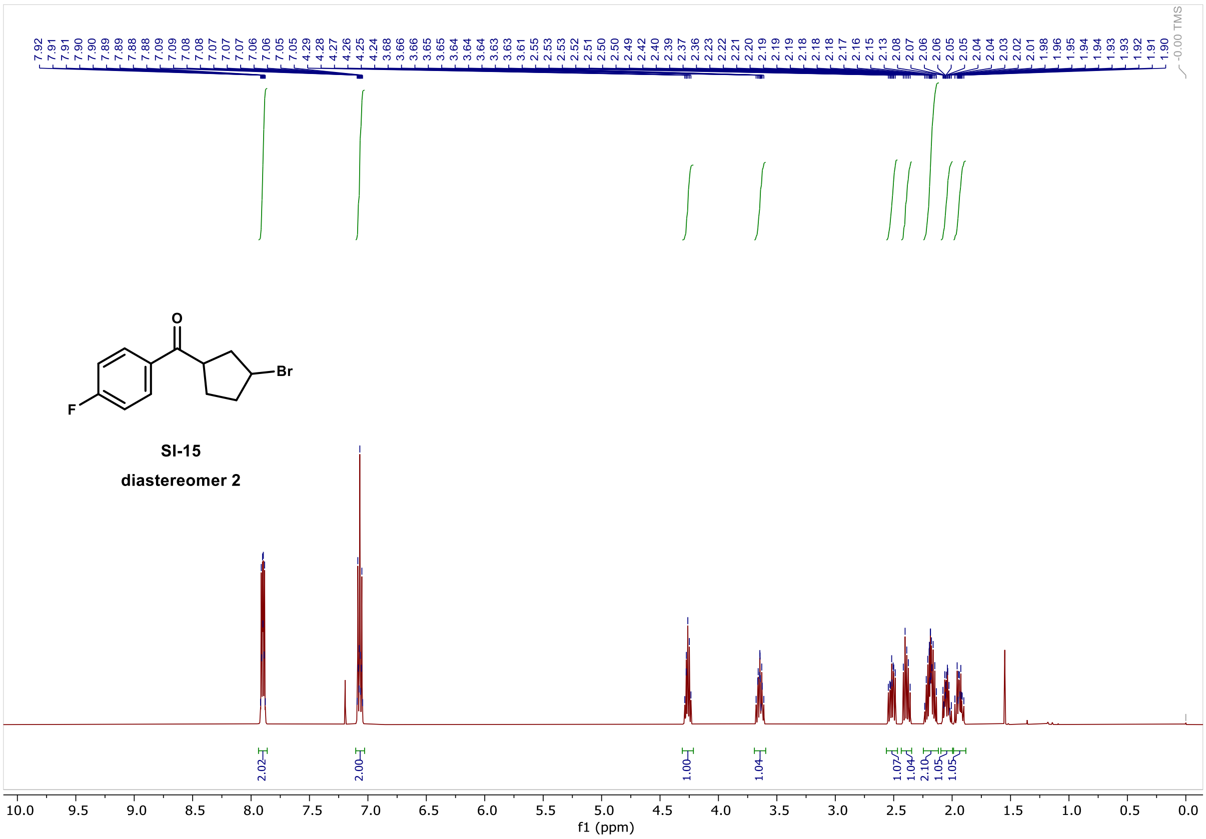

^{13}C NMR (126 MHz, Chloroform-*d*) of **SI-15 – diastereoisomer 2**:

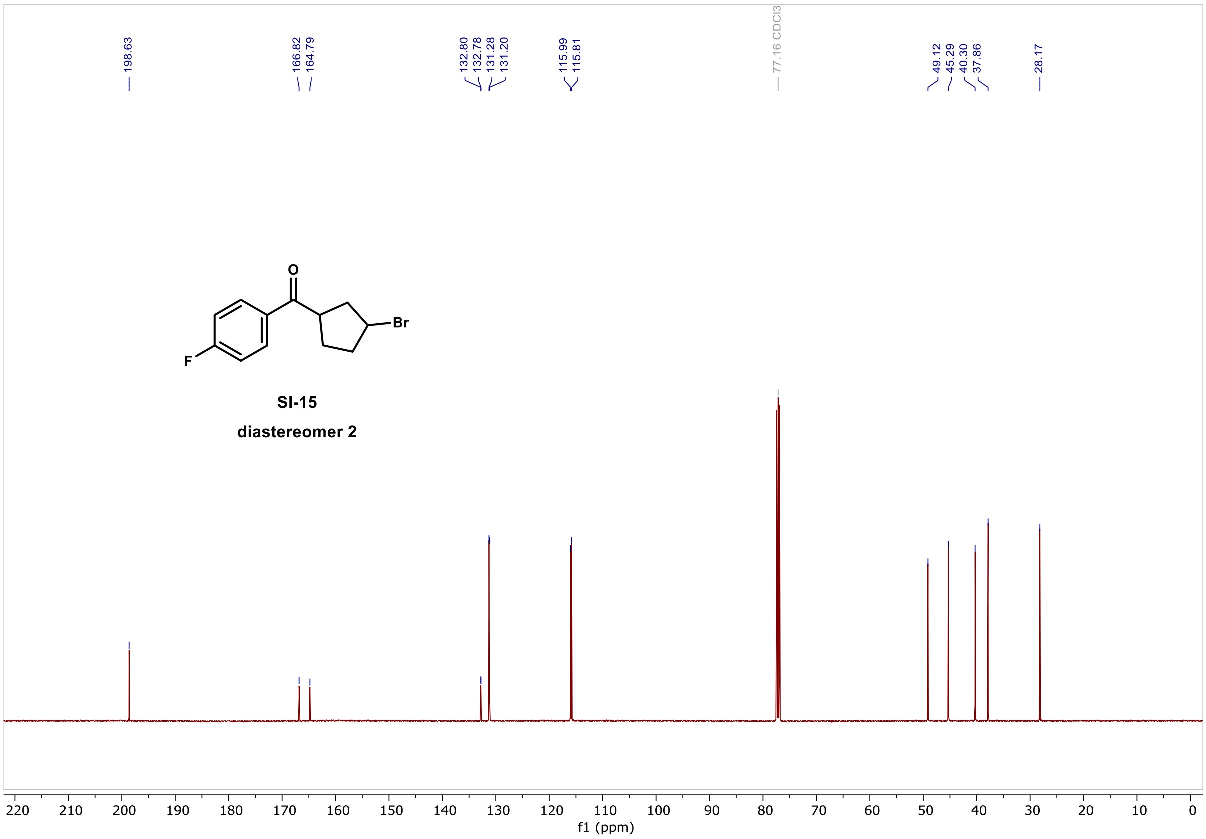

^{19}F NMR (471 MHz, Chloroform-*d*) of **SI-15 – diastereoisomer 2**:

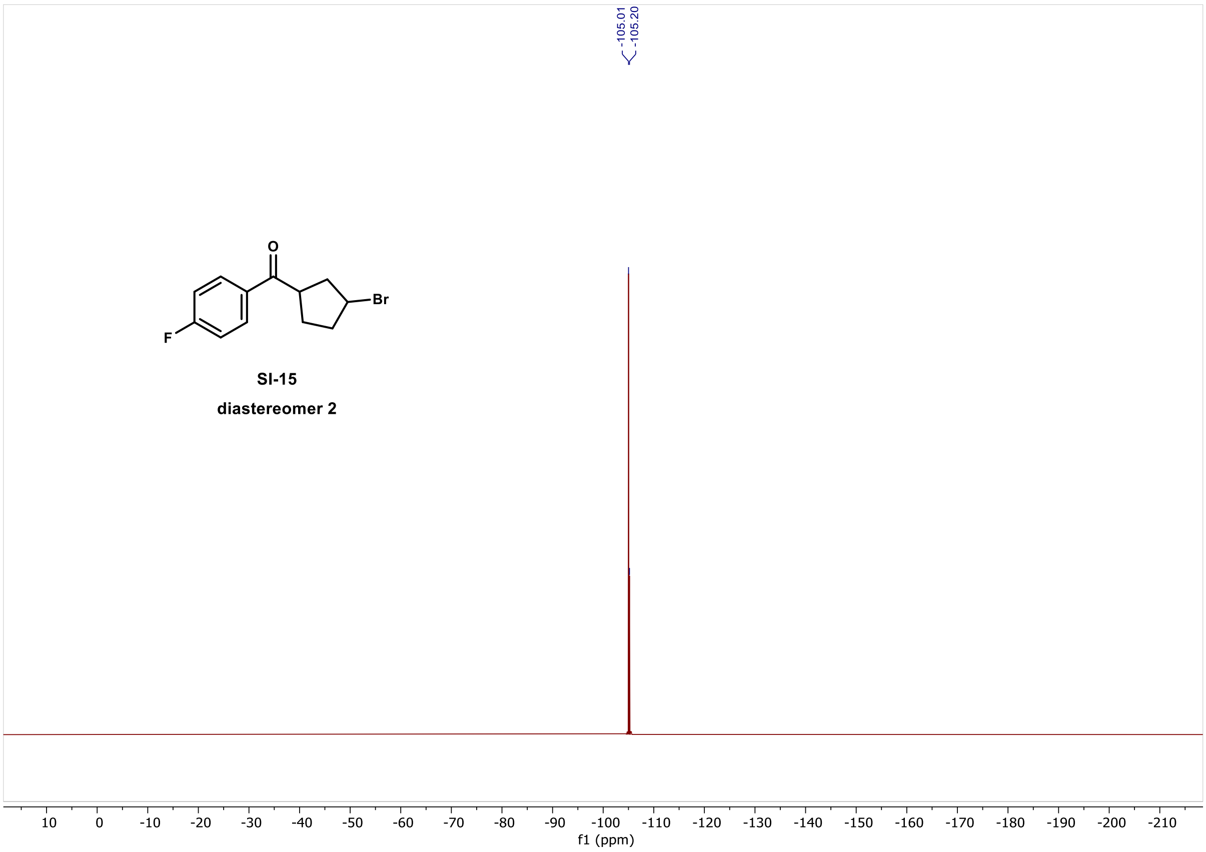

^{1}H NMR (400 MHz, Chloroform-*d*) of **1o**:

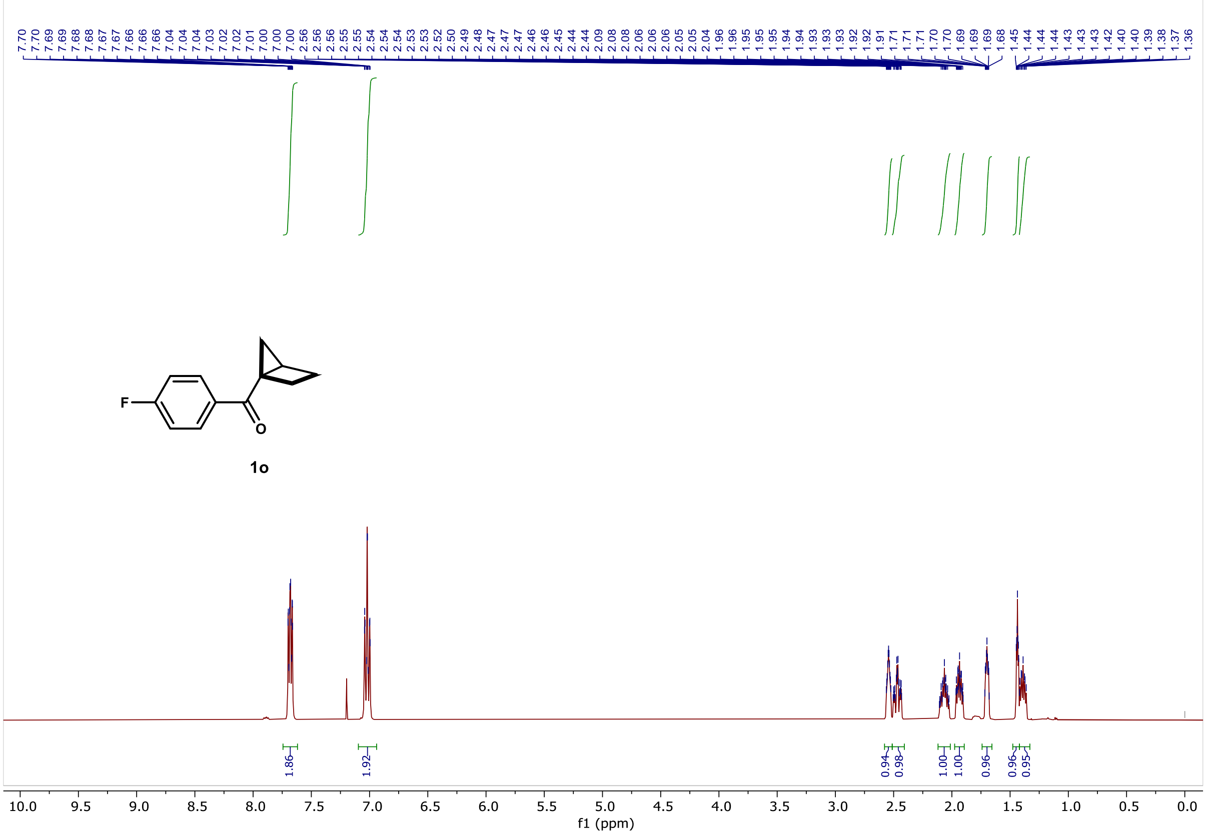

^{13}C NMR (101 MHz, Chloroform-*d*) of **1o**:

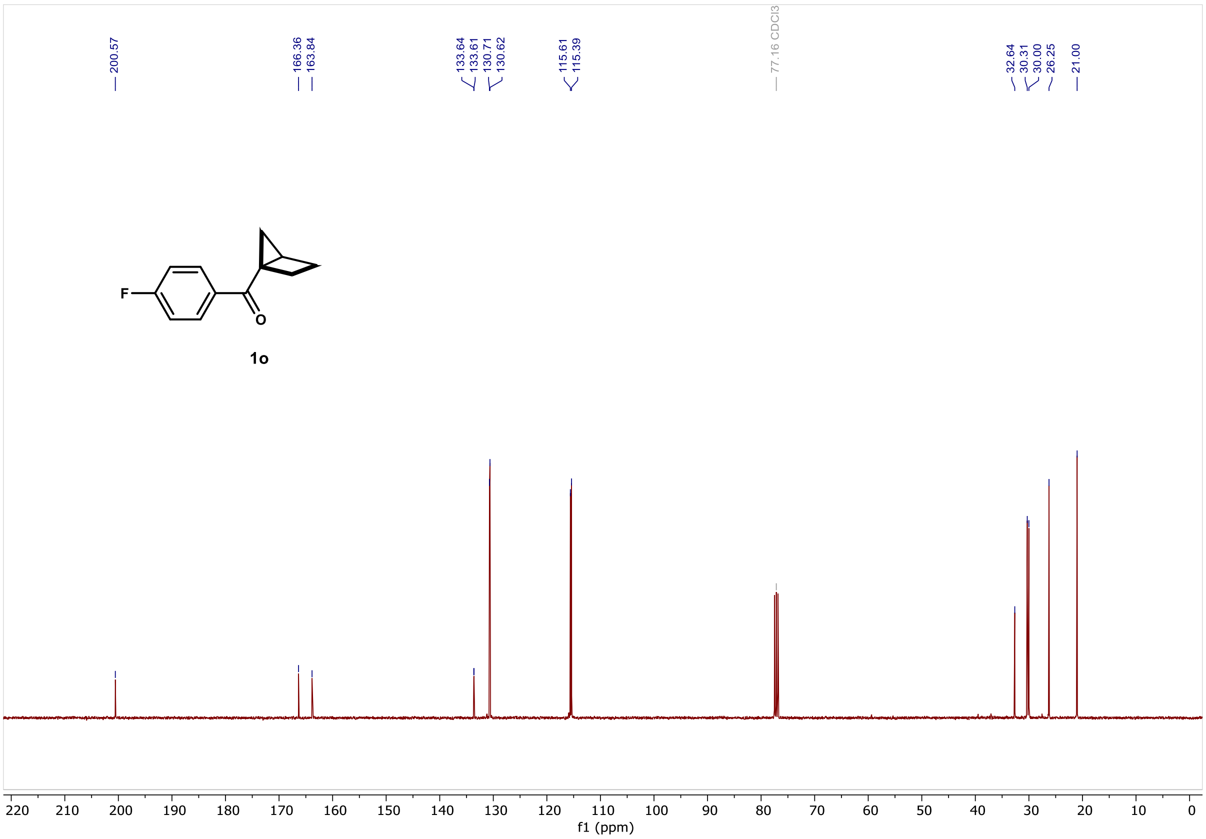

^{19}F NMR (376 MHz, Chloroform-*d*) of **1o**:

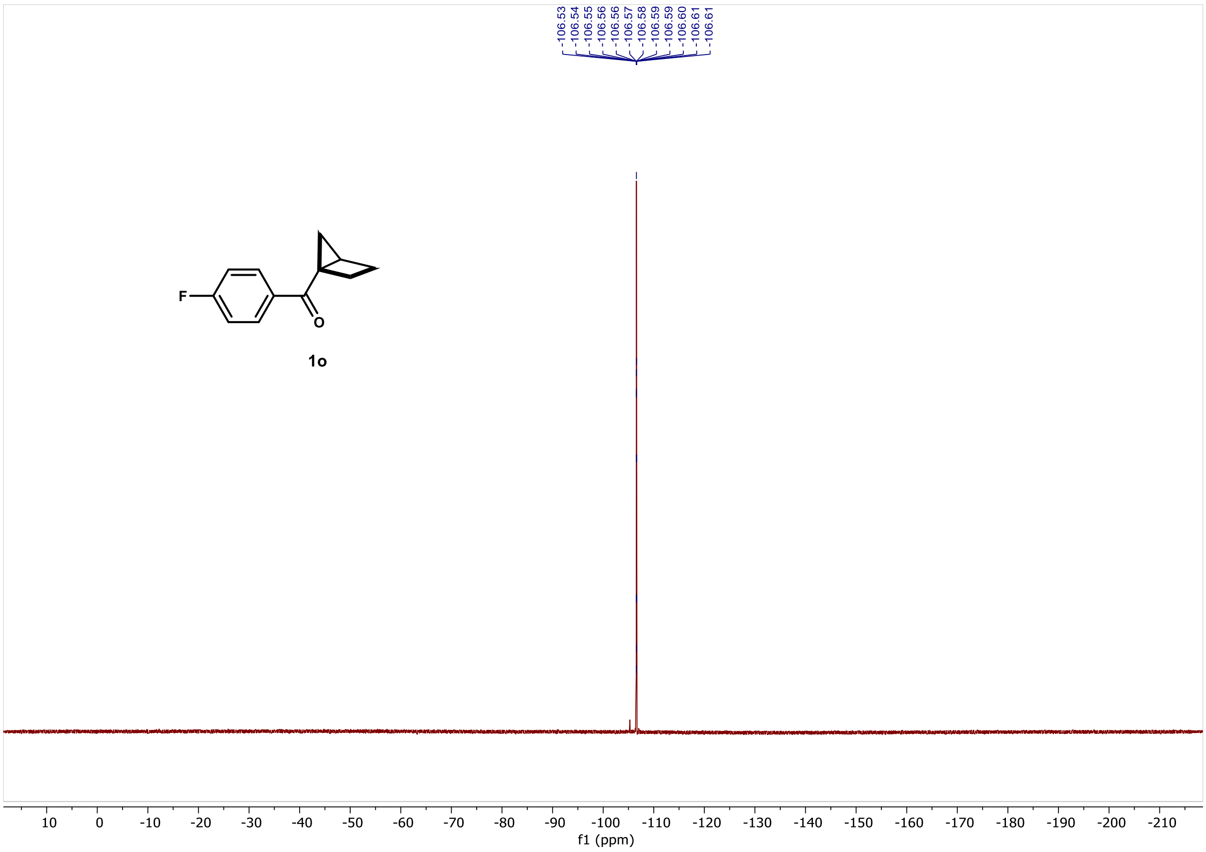

^{1}H NMR (400 MHz, Chloroform-*d*) of **SI-16**:

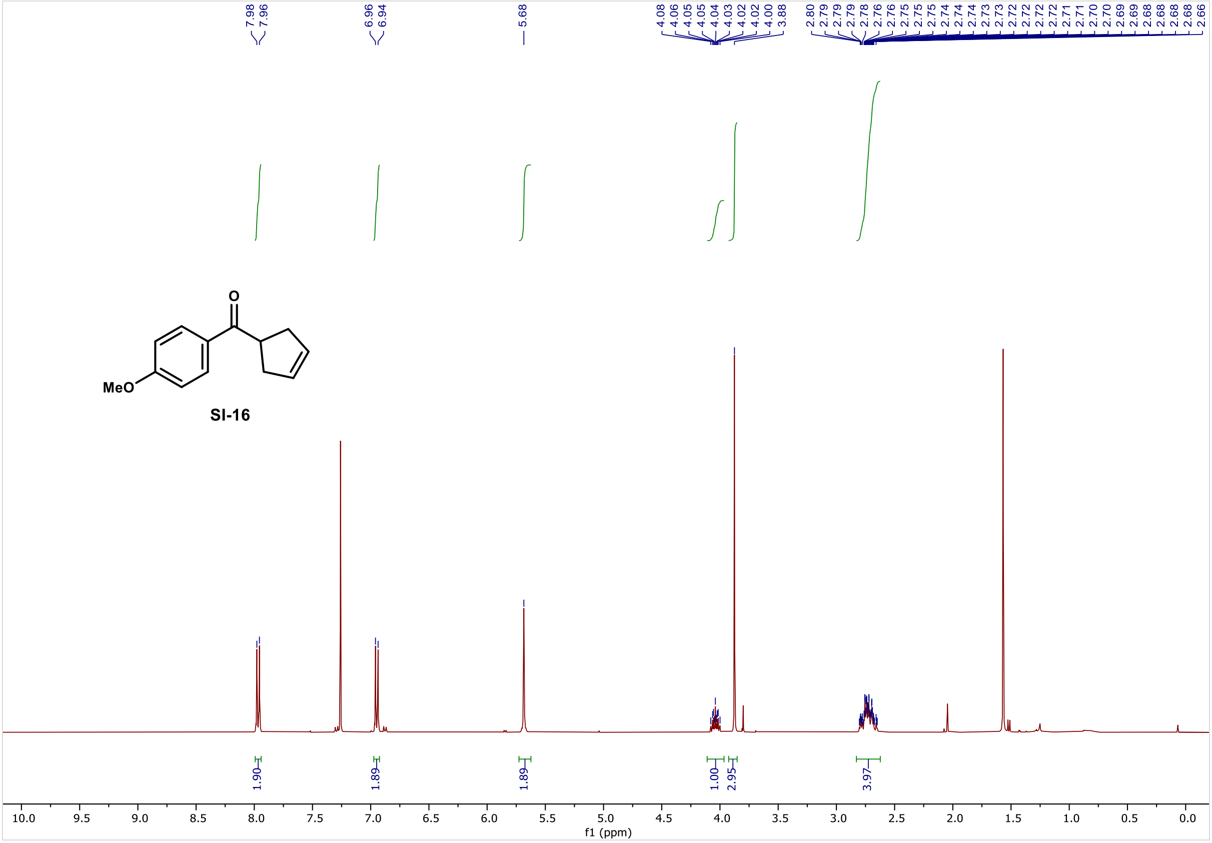

^{13}C NMR (101 MHz, Chloroform-*d*) of **SI-16:**

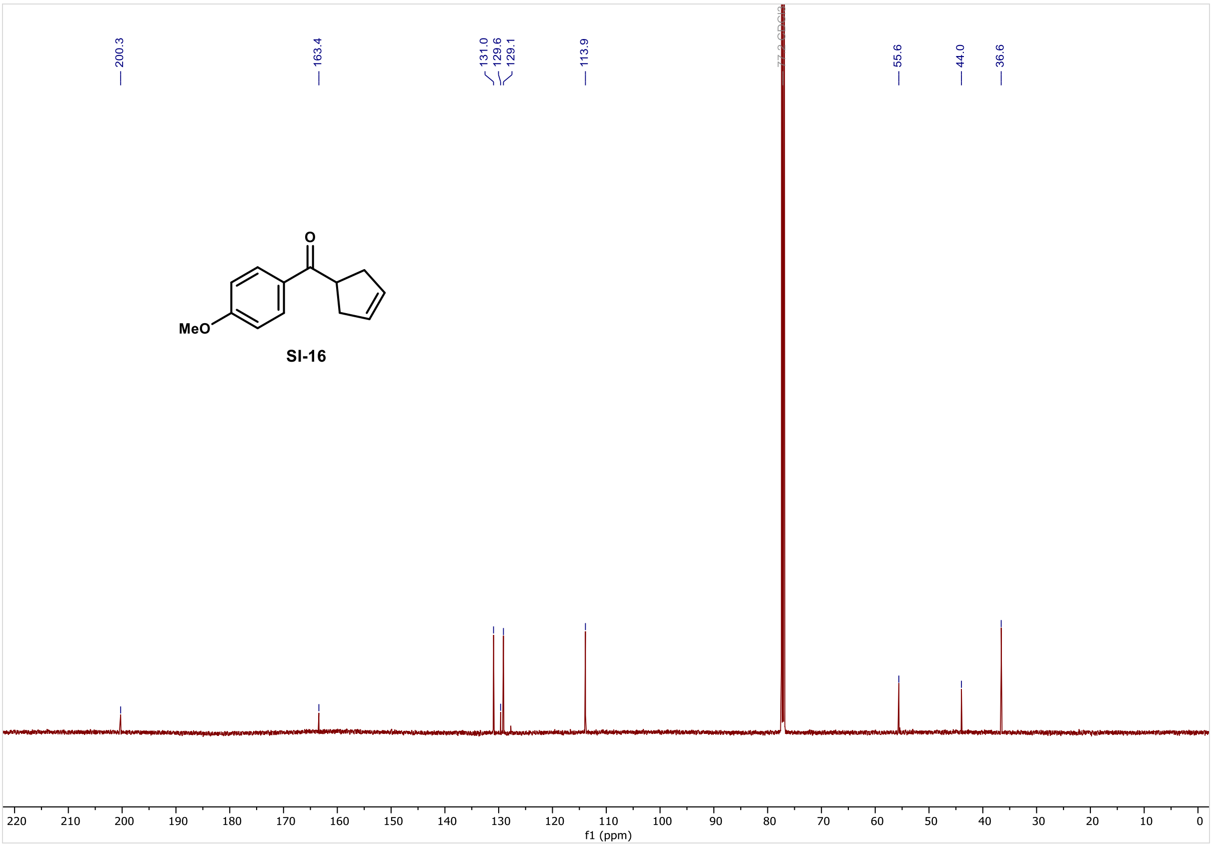

^{1}H NMR (500 MHz, Chloroform-*d*) of **SI-17 – major diastereoisomer**:

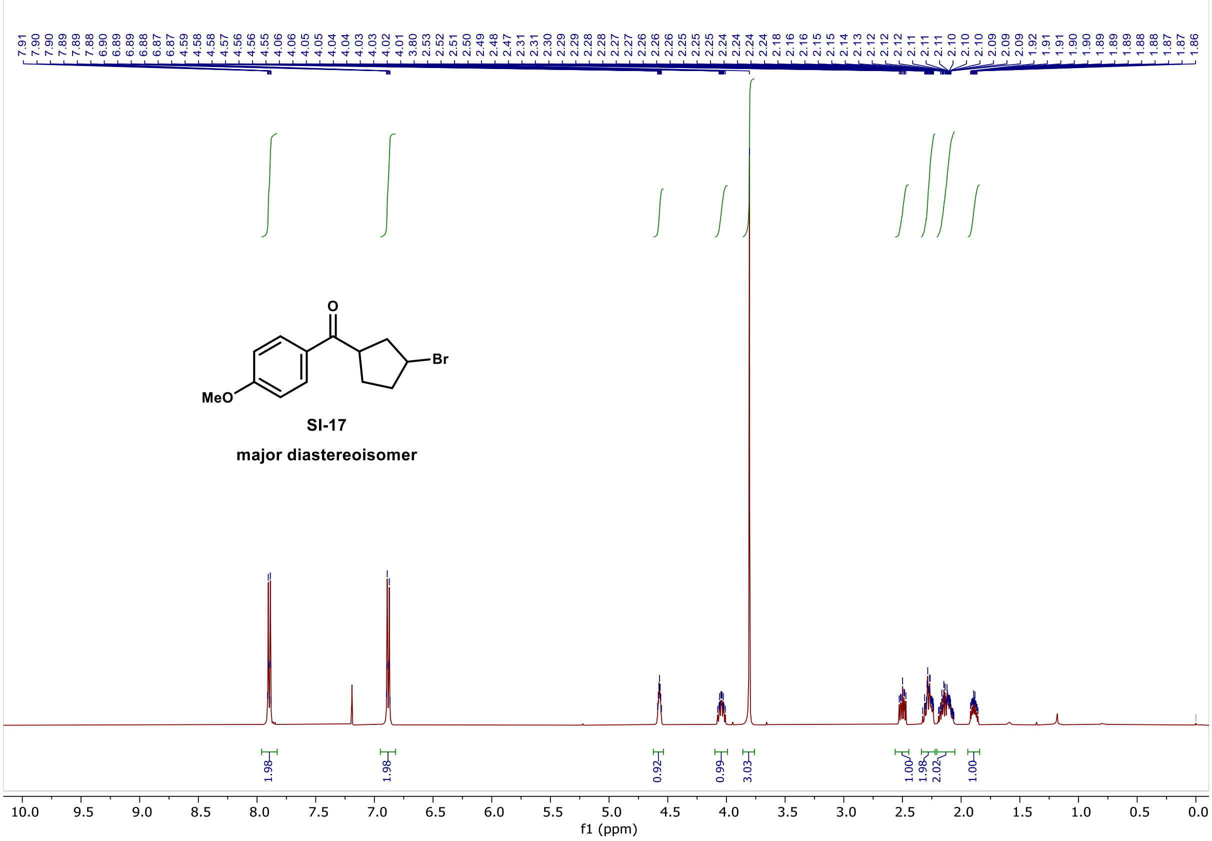

^{13}C NMR (126 MHz, Chloroform-*d*) of **SI-17 – major diastereoisomer**:

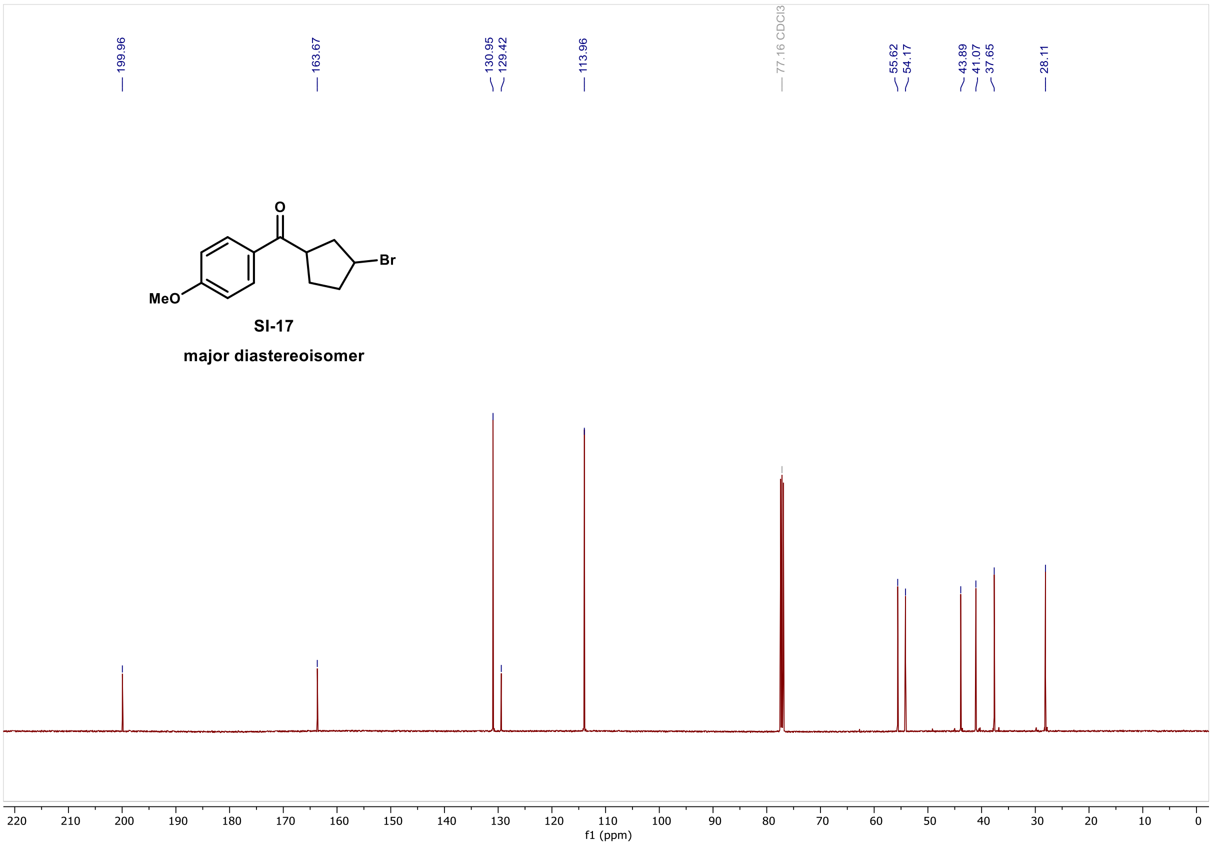

^{1}H NMR (500 MHz, Chloroform-*d*) of **SI-17 – minor diastereoisomer**:

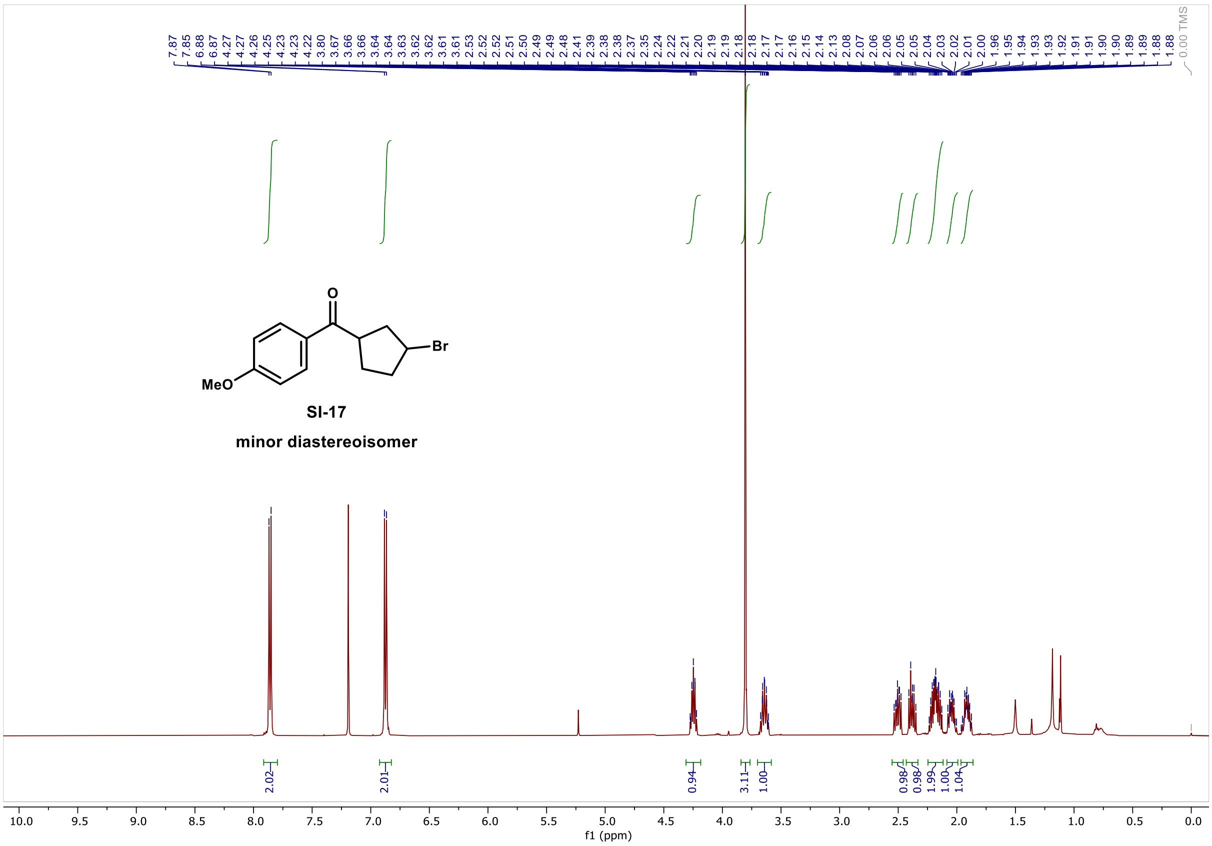

^{13}C NMR (126 MHz, Chloroform-*d*) of **SI-17 – minor diastereoisomer**:

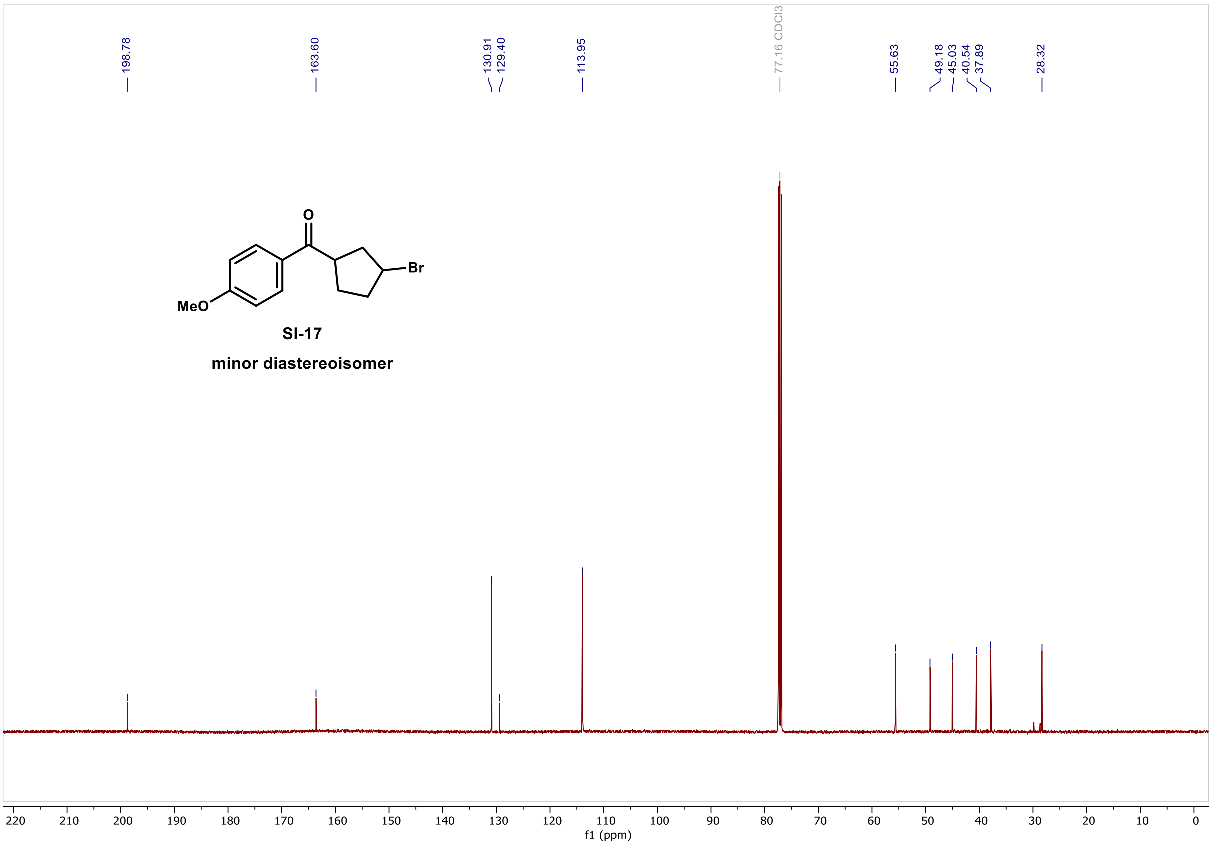

^{1}H NMR (400 MHz, Chloroform-*d*) of **1p**:

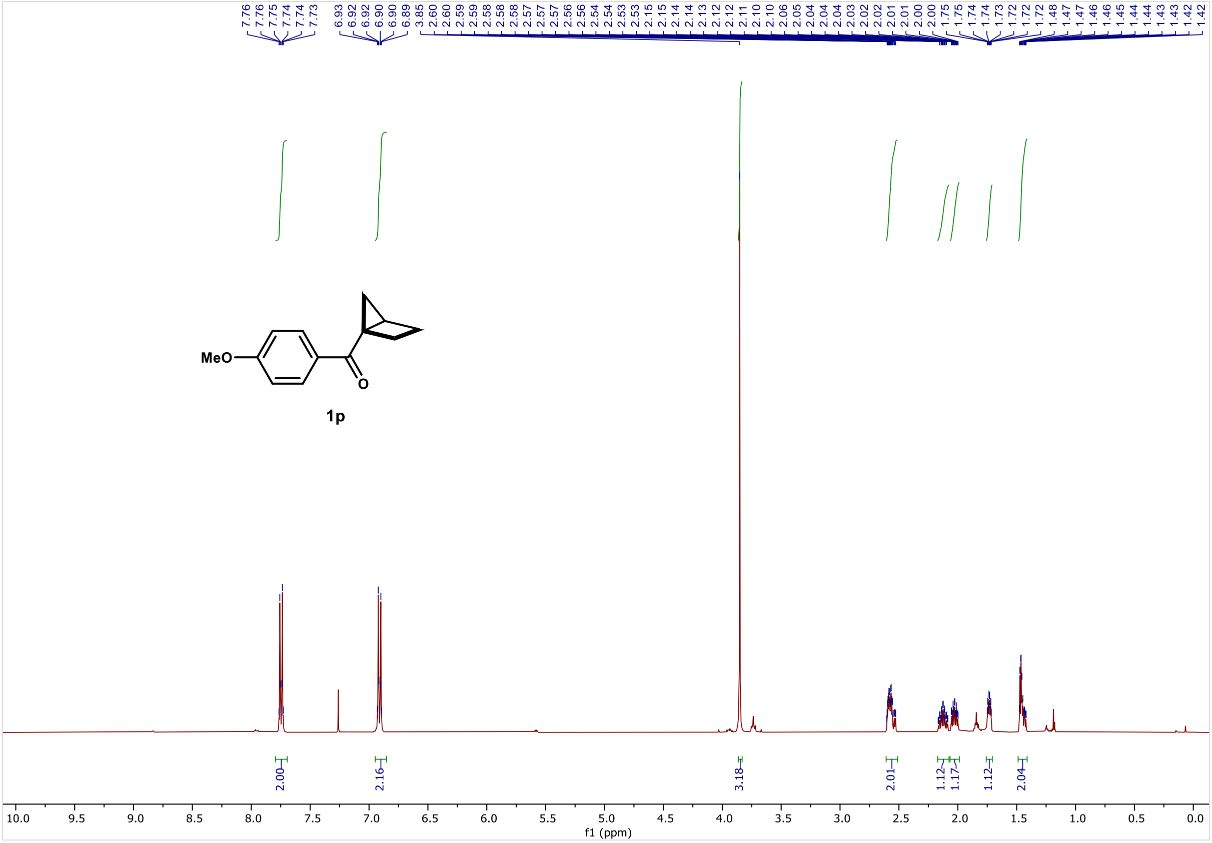

^{13}C NMR (101 MHz, Chloroform-*d*) of **1p**:

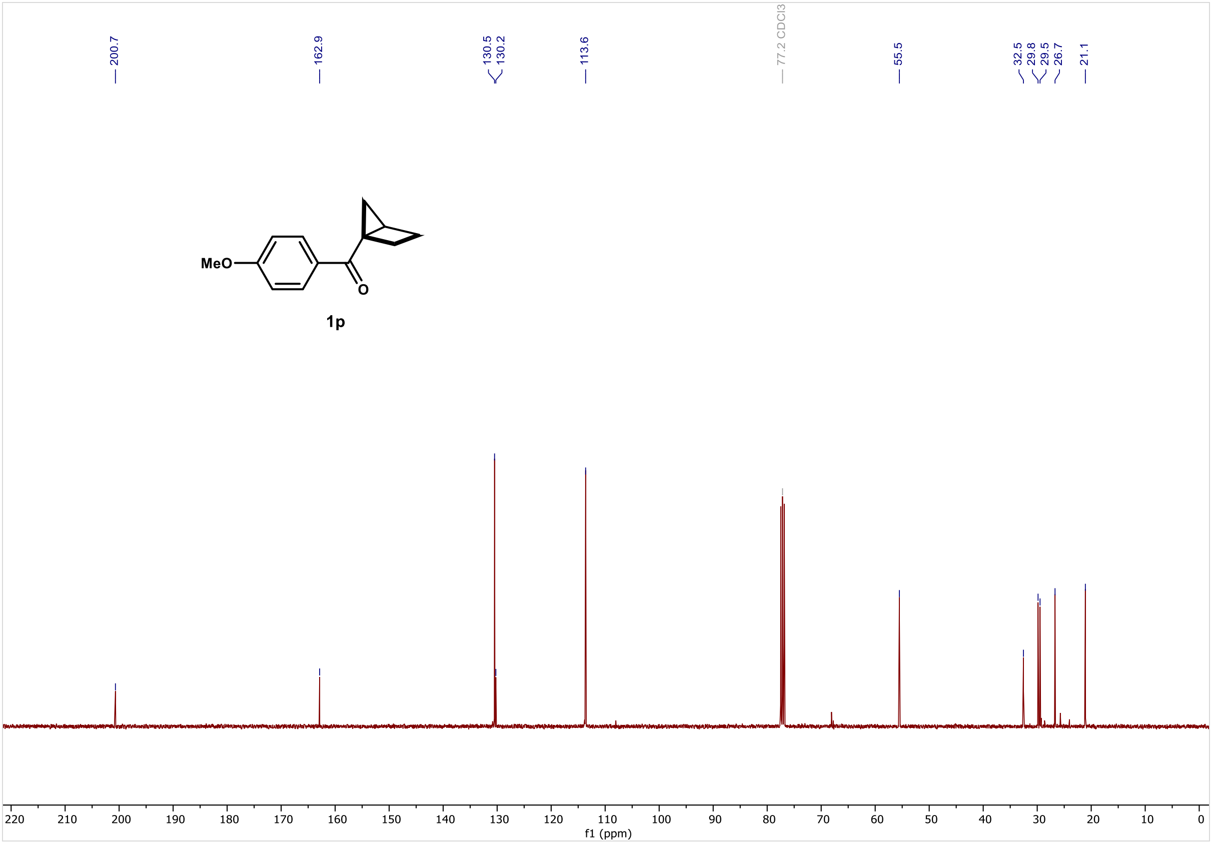

^{1}H NMR (400 MHz, Chloroform-*d*) of **SI-18**:

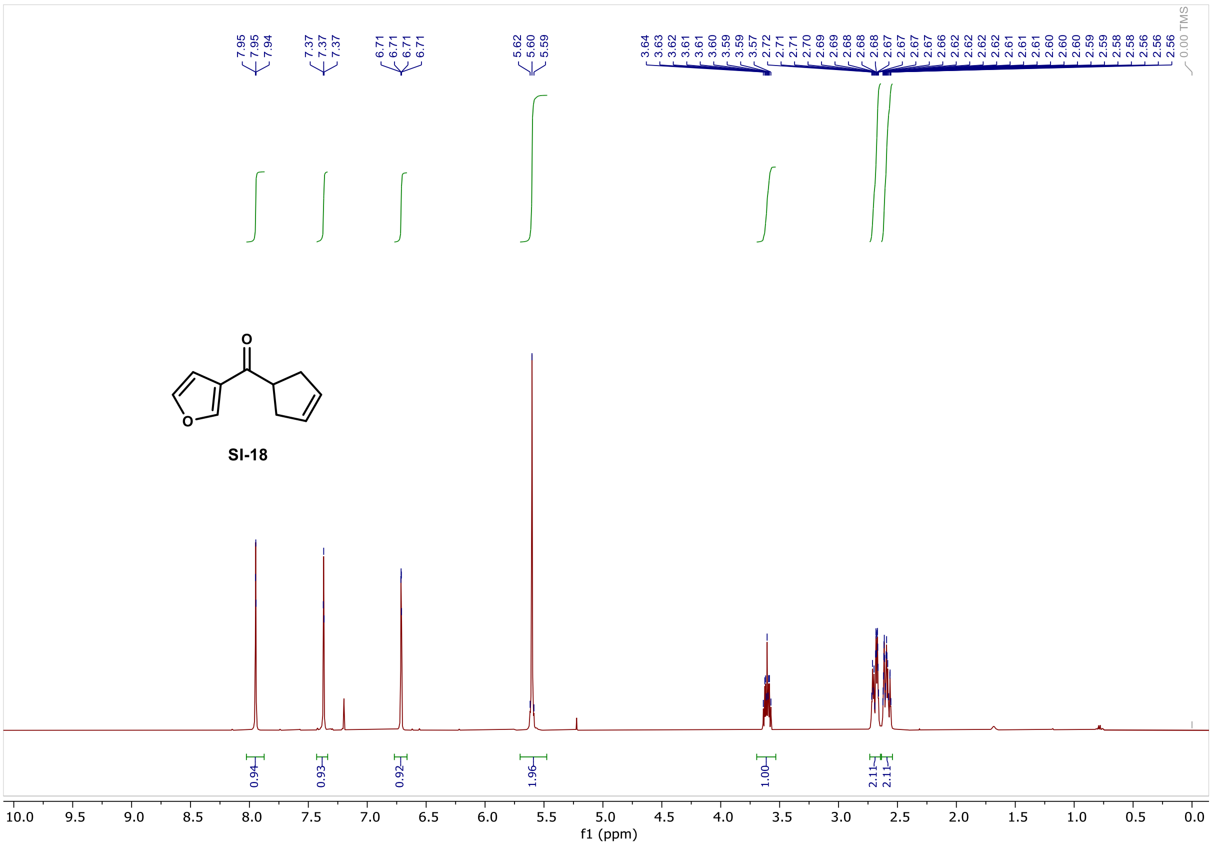

^{13}C NMR (101 MHz, Chloroform-*d*) of **SI-18**:

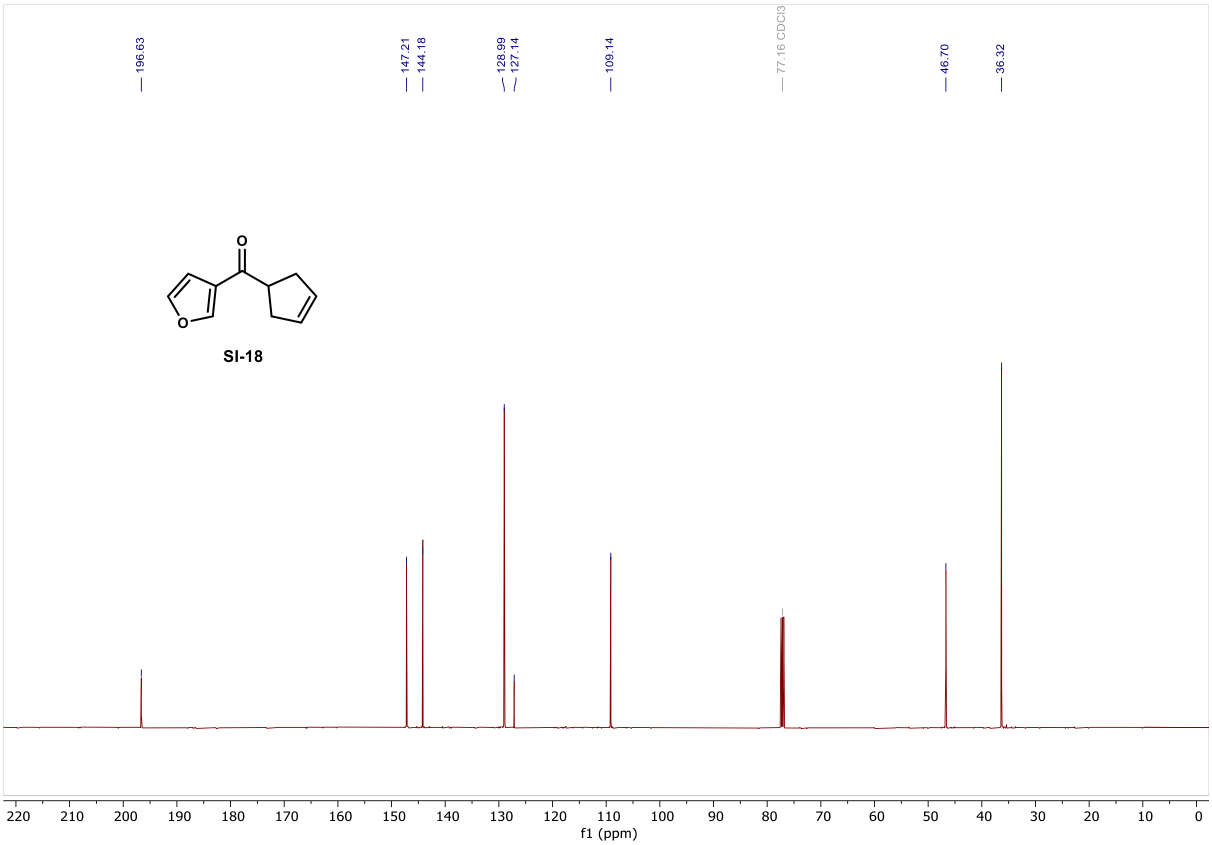

^{1}H NMR (400 MHz, Chloroform-*d*) of **SI-19 – major diastereoisomer**:

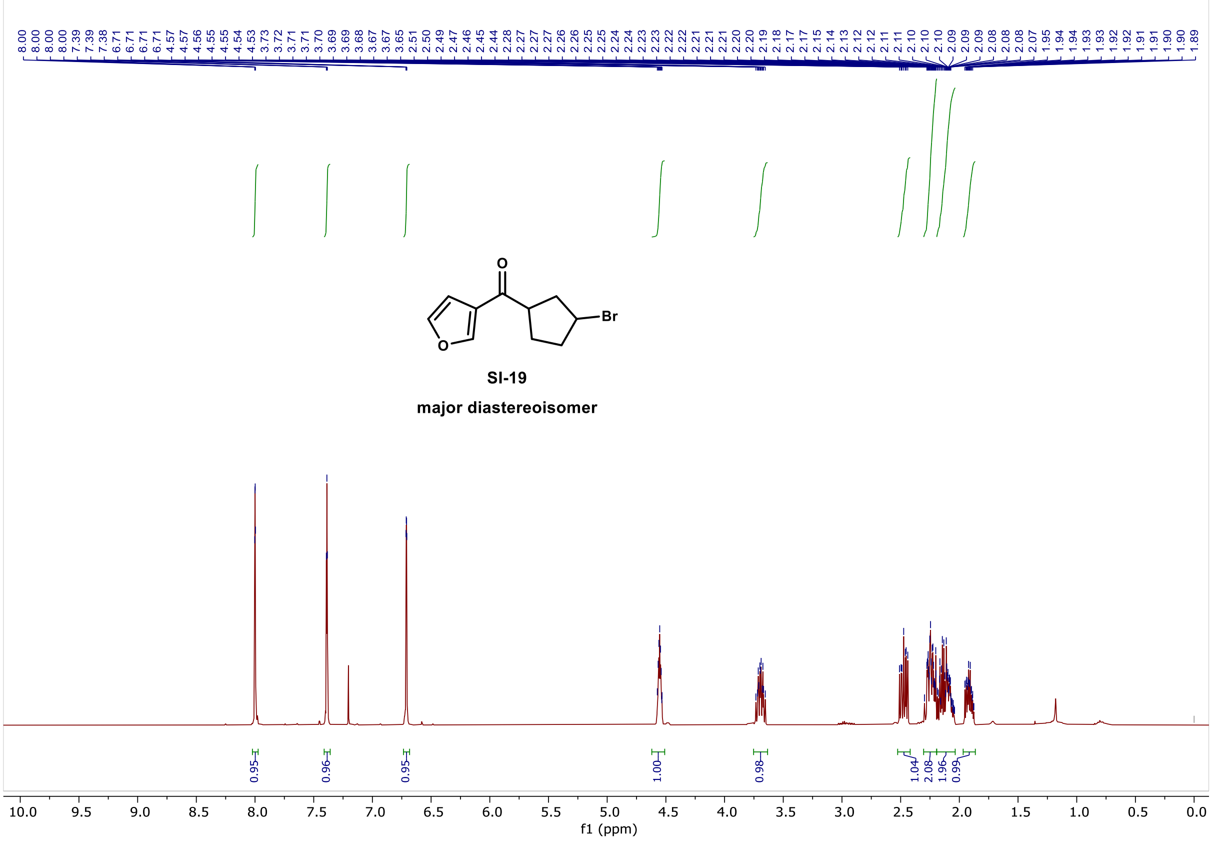

^{13}C NMR (101 MHz, Chloroform-*d*) of **SI-19 – major diastereoisomer**:

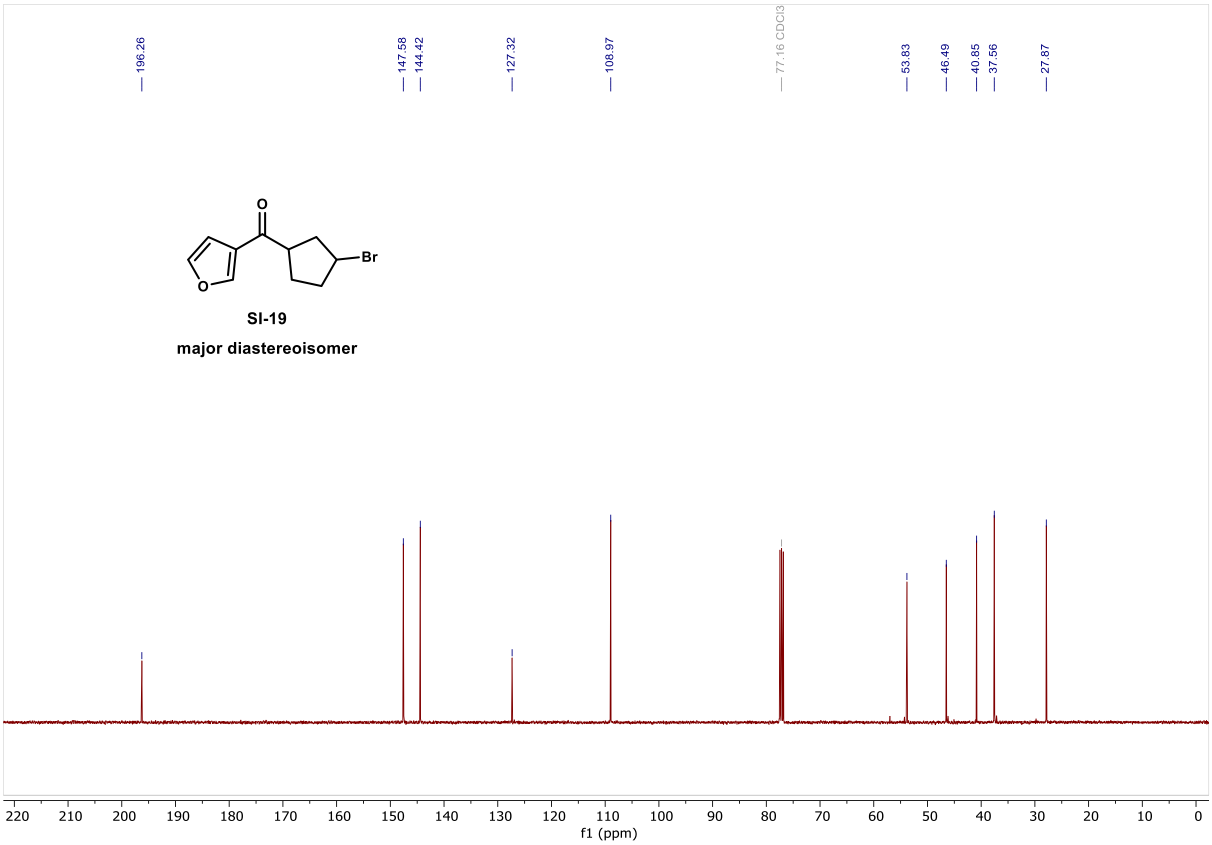

^{1}H NMR (400 MHz, Chloroform-*d*) of **SI-19 – minor diastereoisomer**:

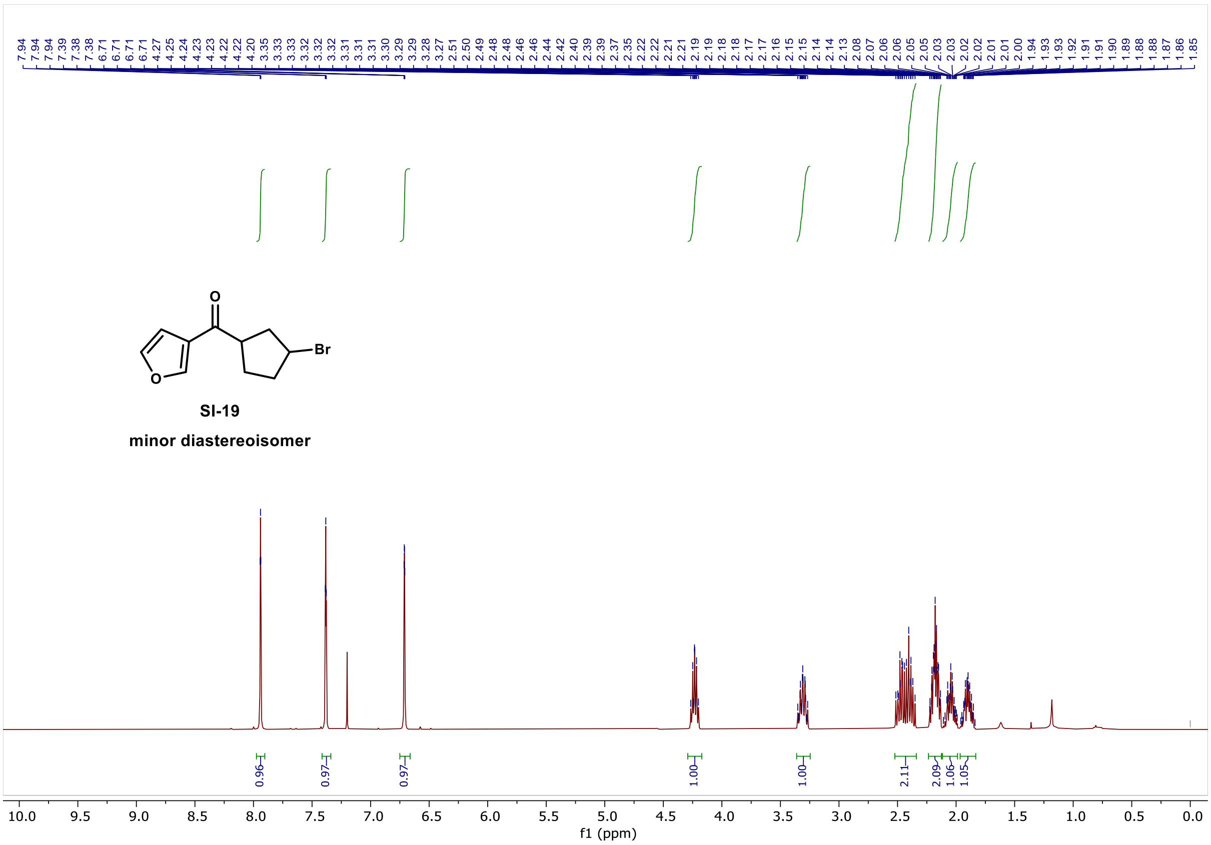

^{13}C NMR (101 MHz, Chloroform-*d*) of **SI-19 – minor diastereoisomer**:

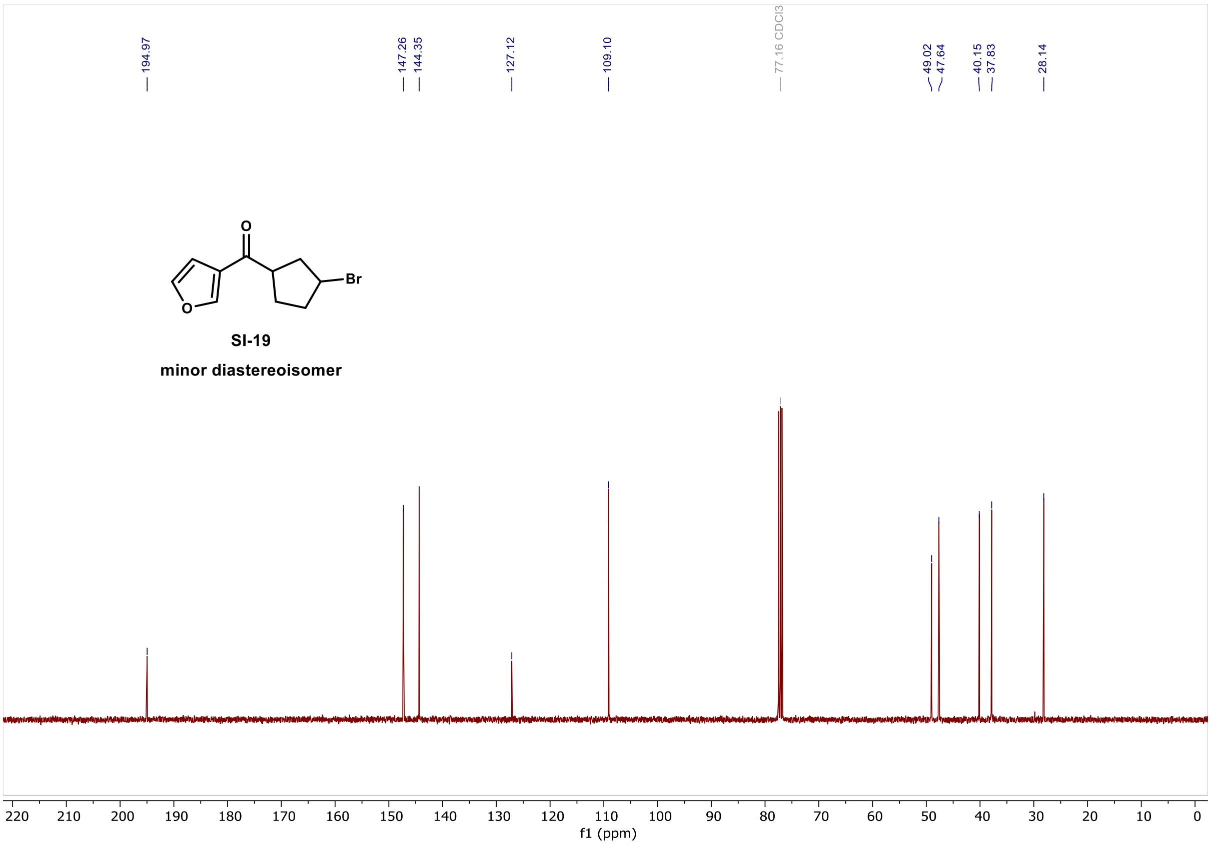

^{1}H NMR (400 MHz, Chloroform-*d*) of **1q**:

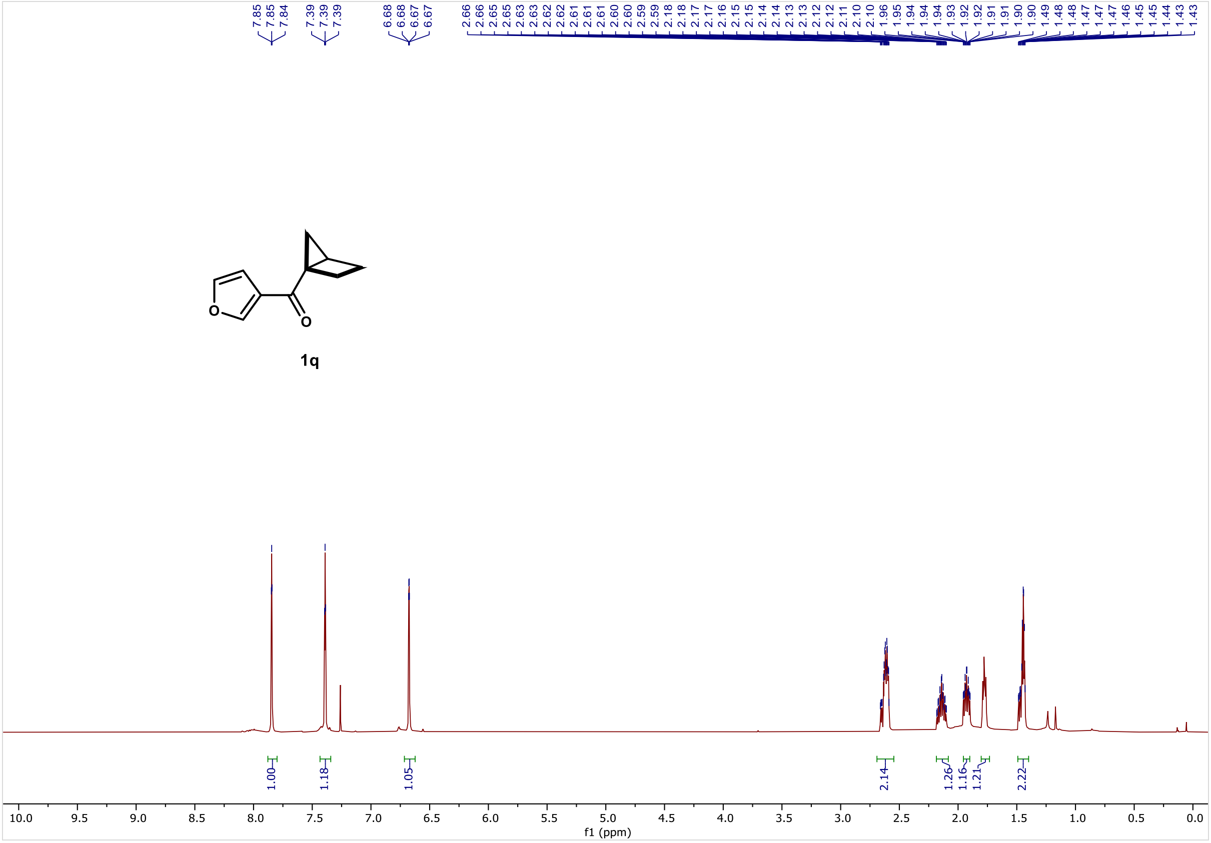

^{13}C NMR (101 MHz, Chloroform-*d*) of **1q**:

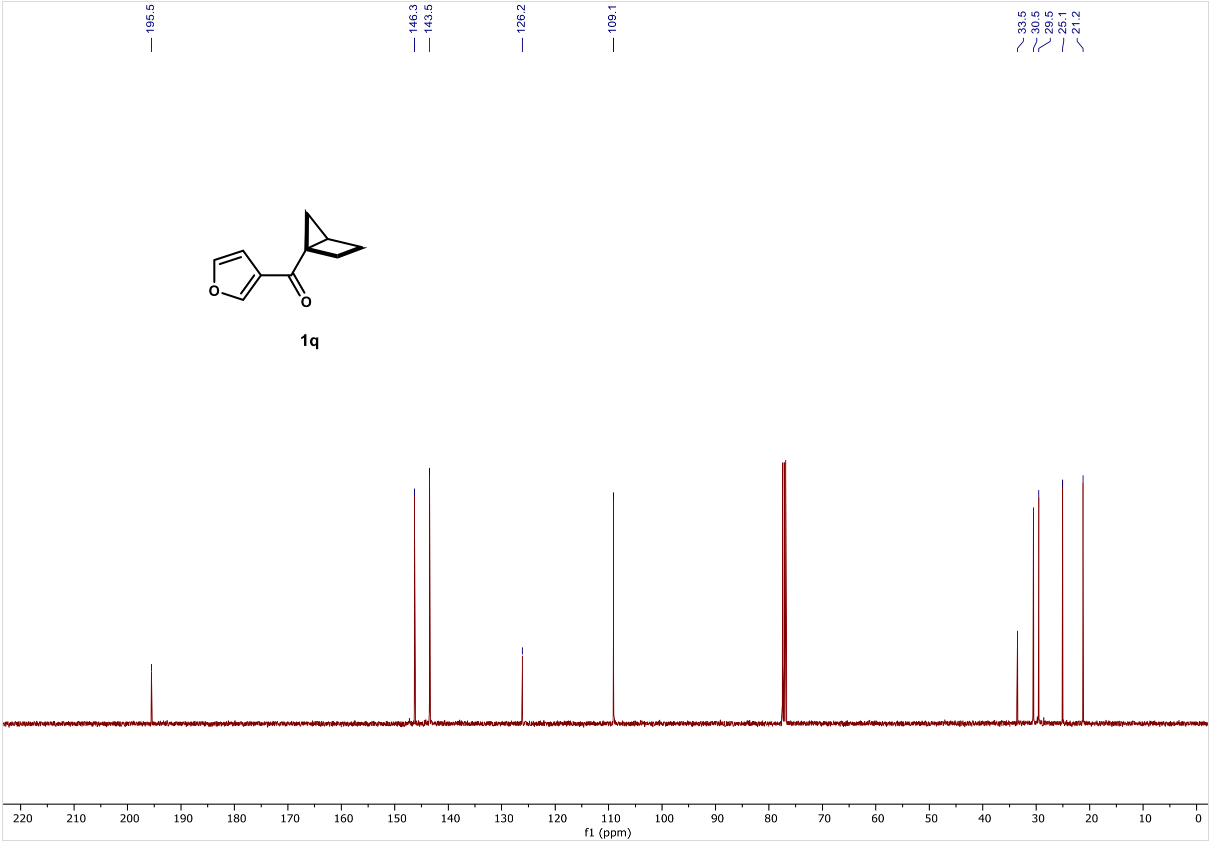

^{1}H NMR (500 MHz, Chloroform-*d*) of **SI-20**:

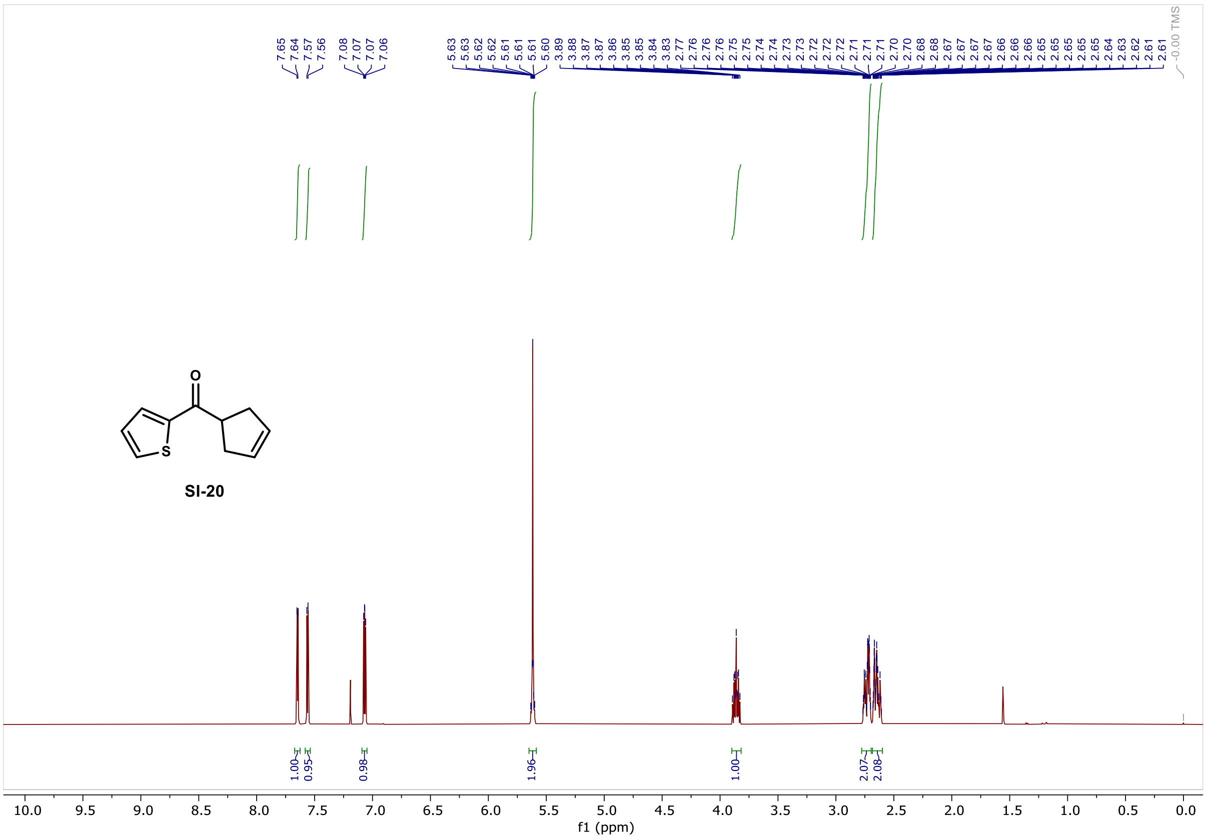

^{13}C NMR (126 MHz, Chloroform-*d*) of **SI-20**:

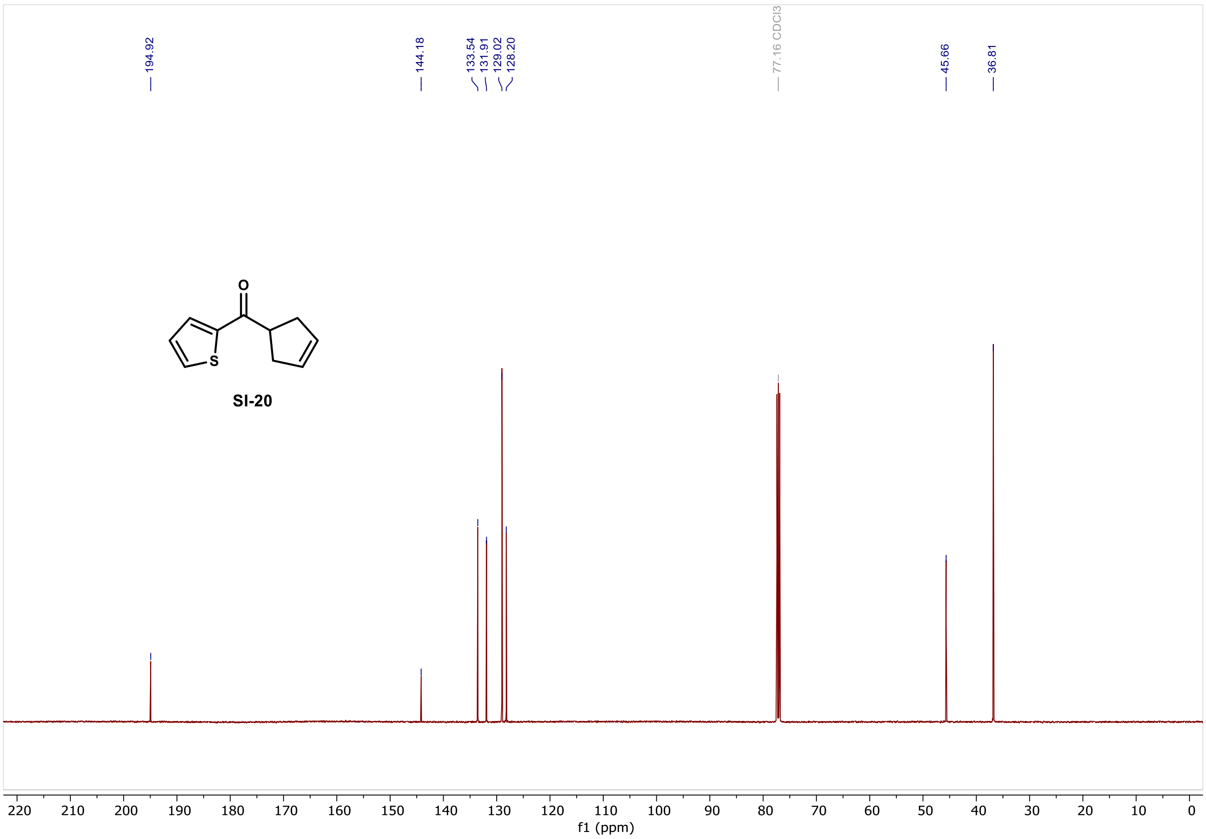

^{1}H NMR (500 MHz, Chloroform-*d*) of **SI-21 – diastereoisomer 1**:

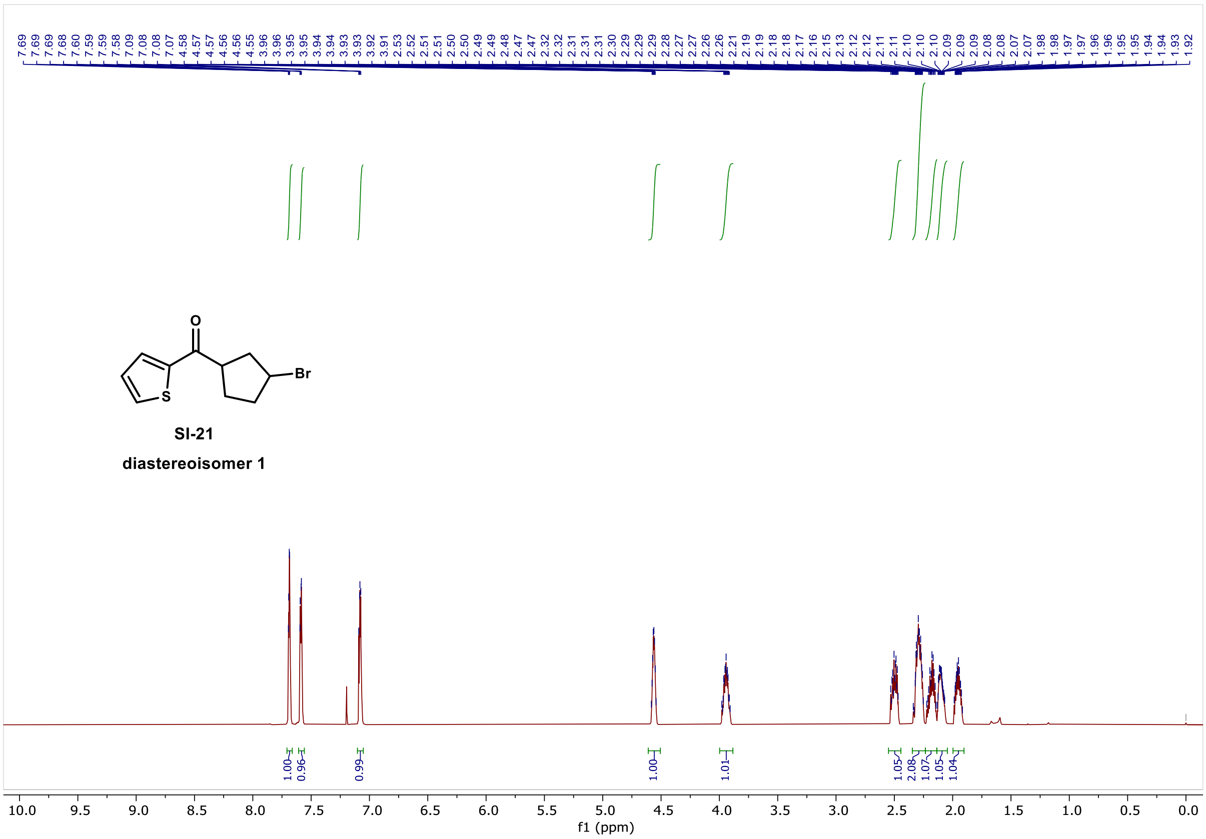

^{13}C NMR (126 MHz, Chloroform-*d*) of **SI-21 – diastereoisomer 1**:

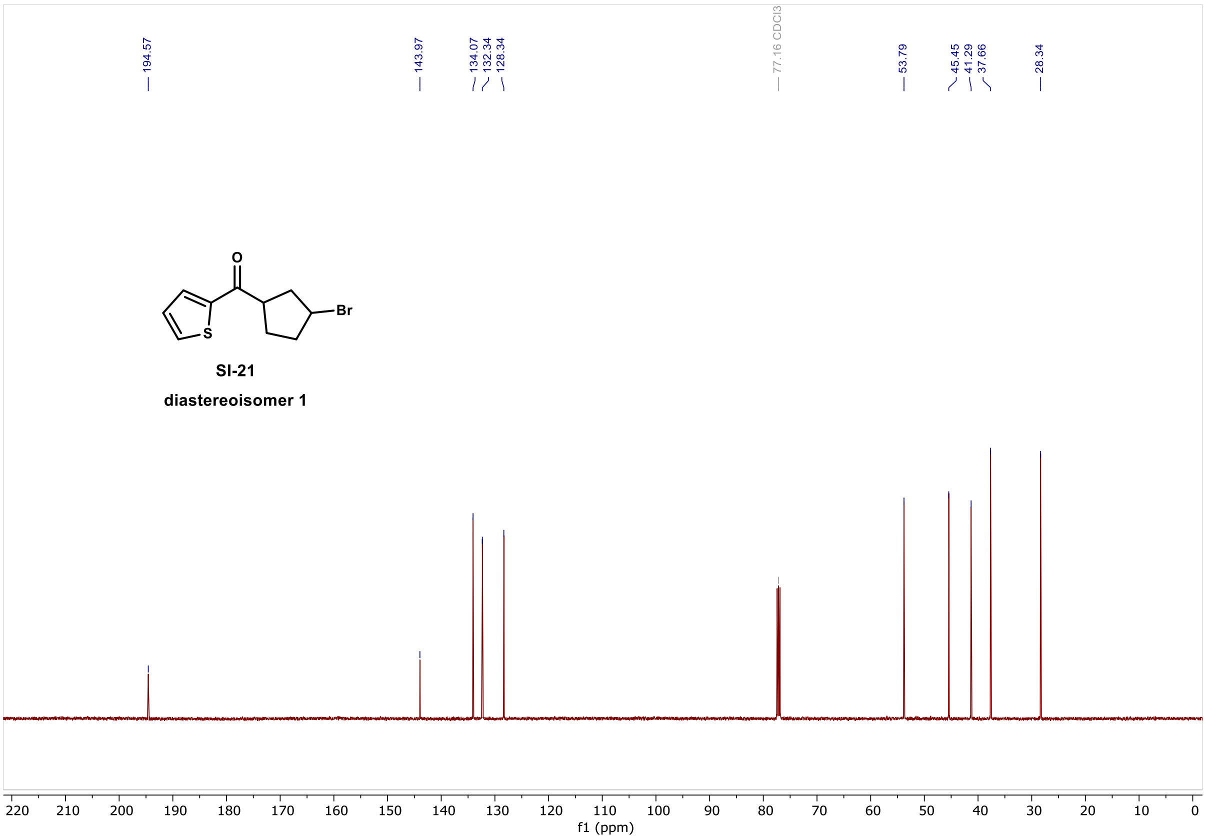

^{1}H NMR (500 MHz, Chloroform-*d*) of **SI-21 – diastereoisomer 2**:

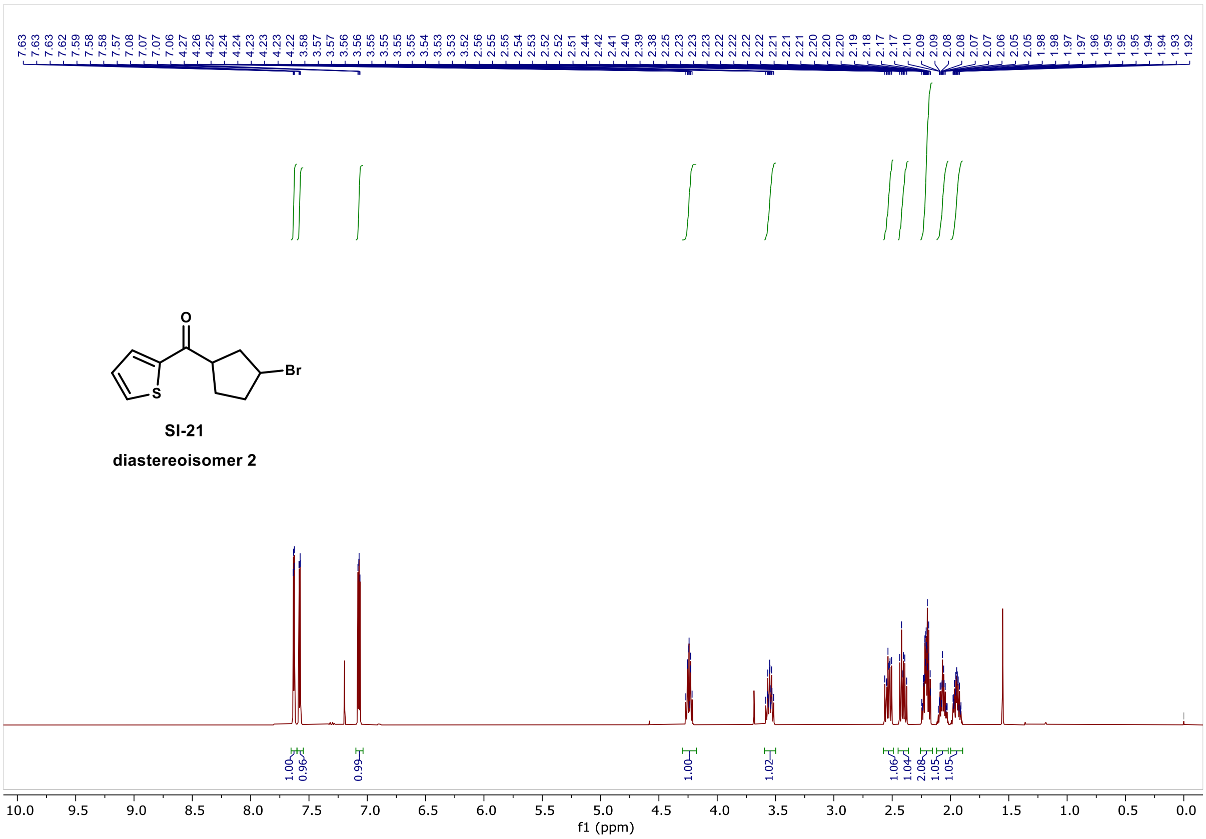

^{13}C NMR (126 MHz, Chloroform-*d*) of **SI-21 – diastereoisomer 2**:

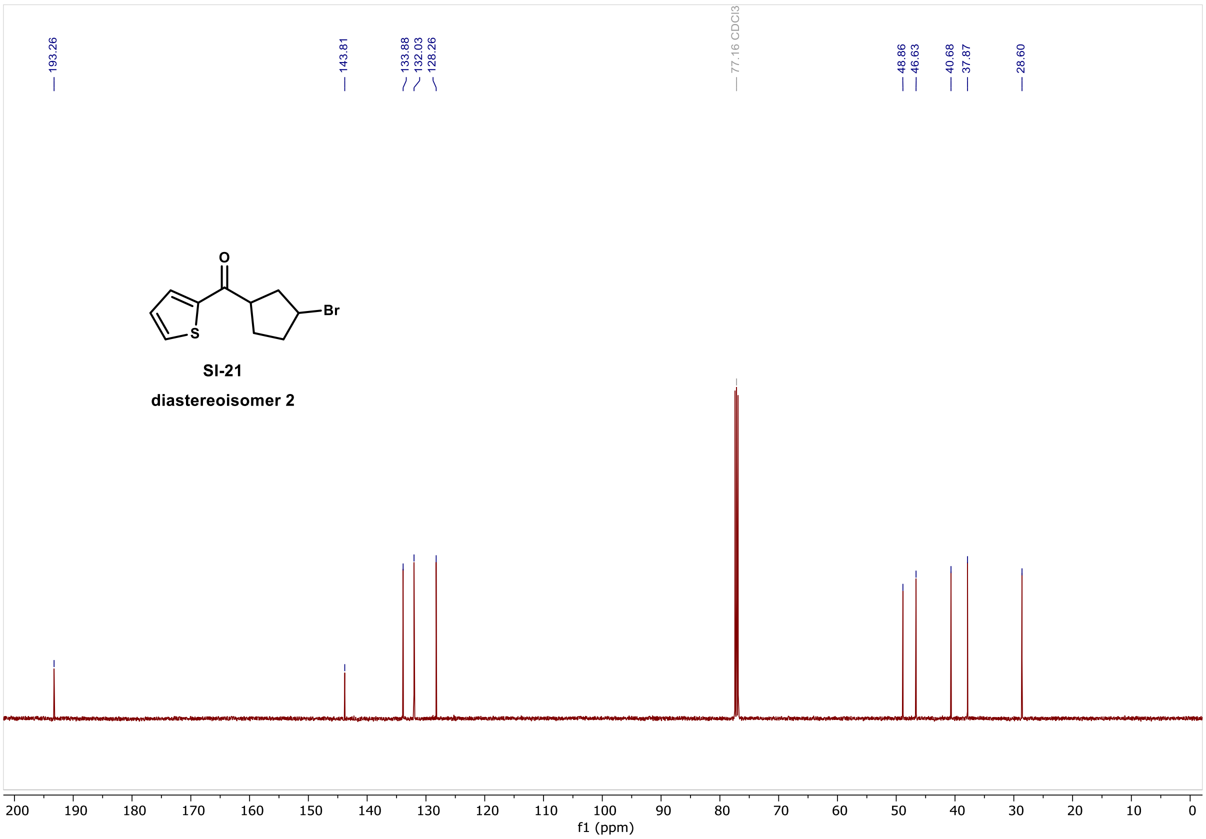

^1H NMR (400 MHz, Chloroform-*d*) of **1r**:

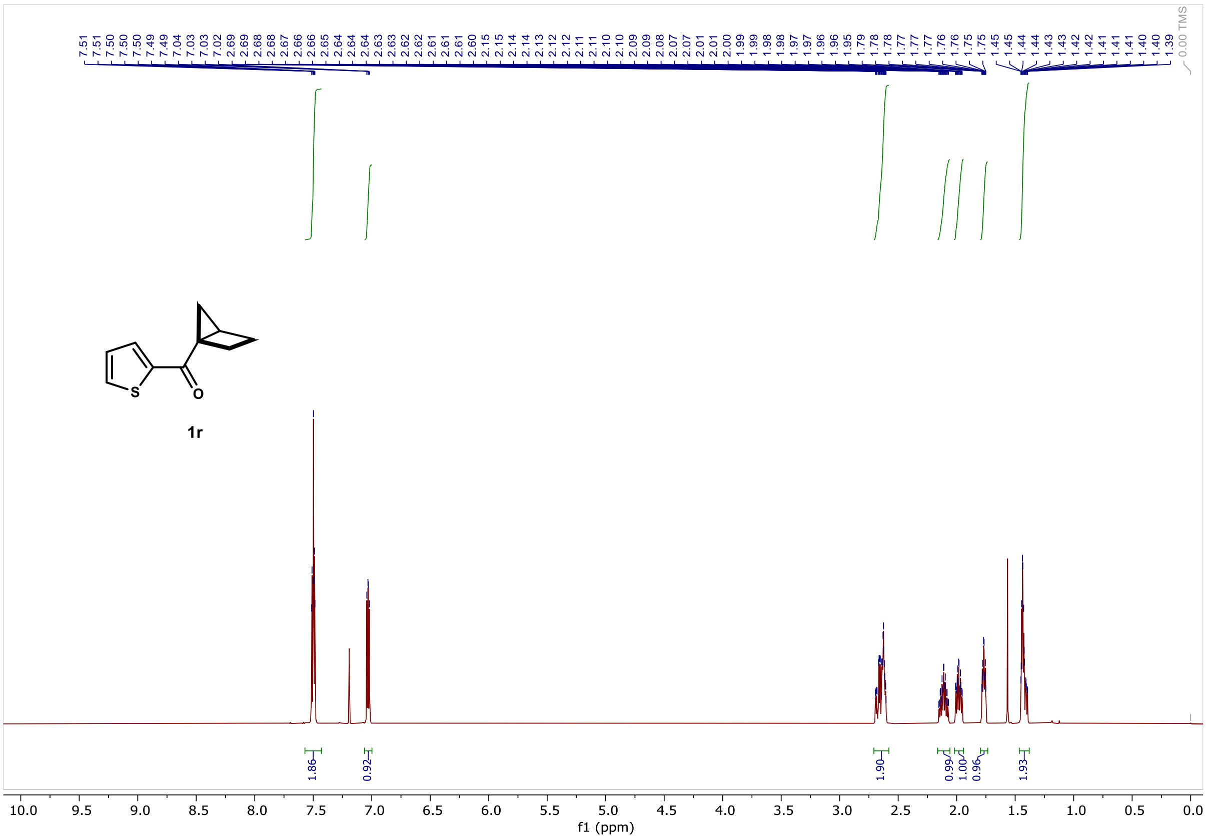

^{13}C NMR (101 MHz, Chloroform-*d*) of **1r**:

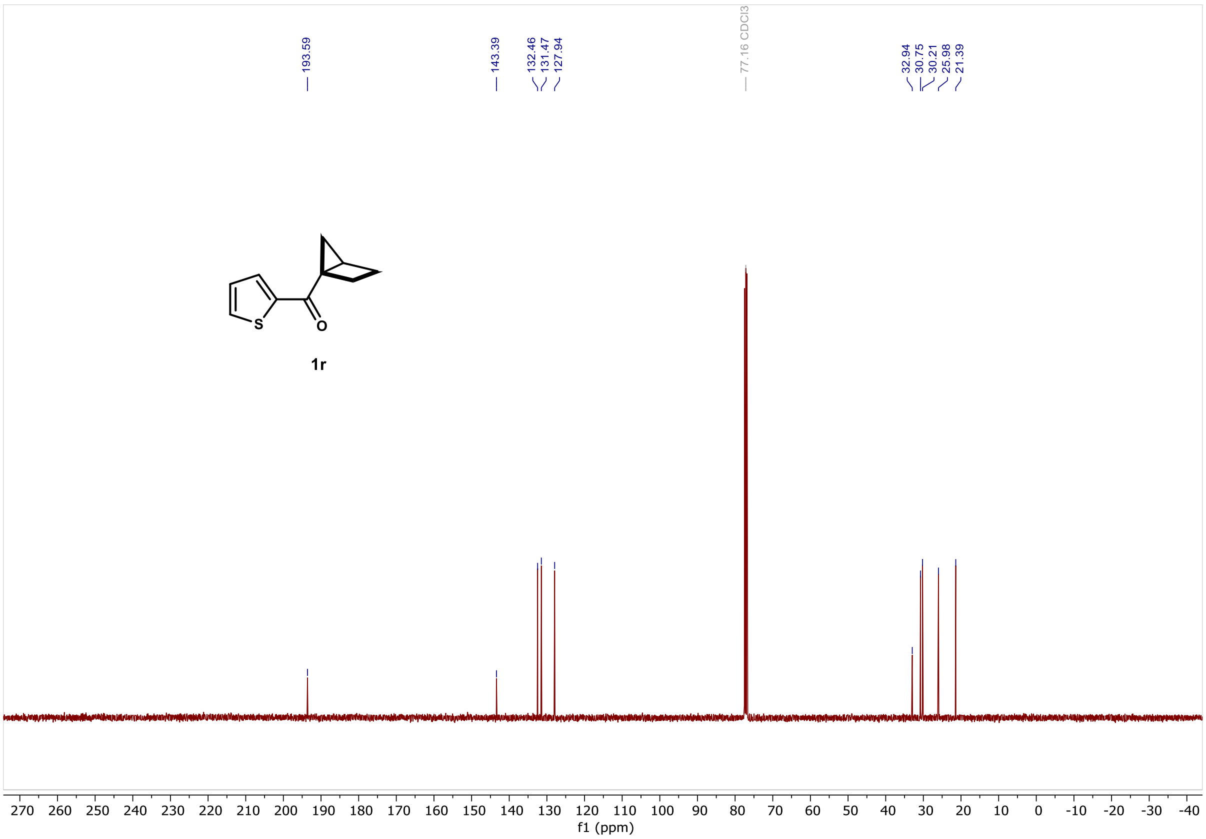

^{1}H NMR (500 MHz, Chloroform-*d*) of **SI-22**:

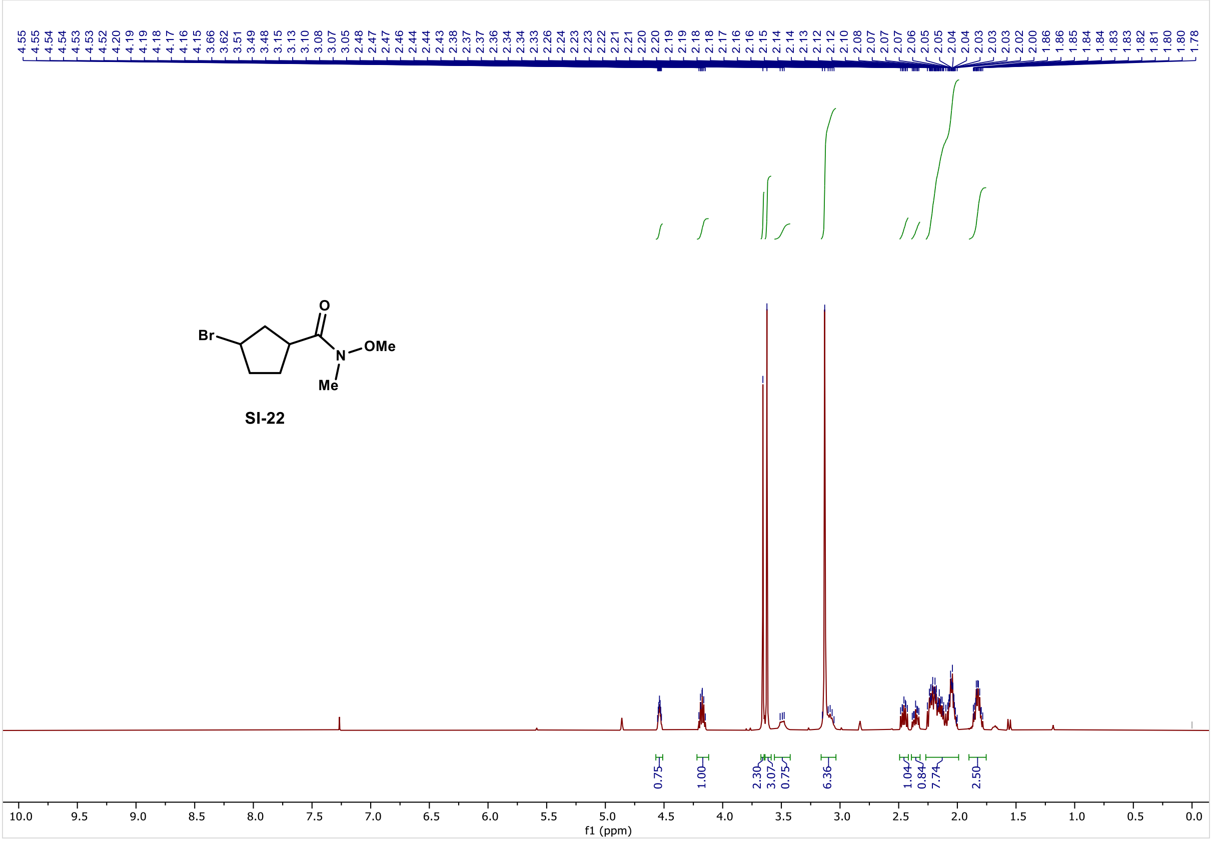

^{13}C NMR (101 MHz, Chloroform-*d*) of **SI-22**:

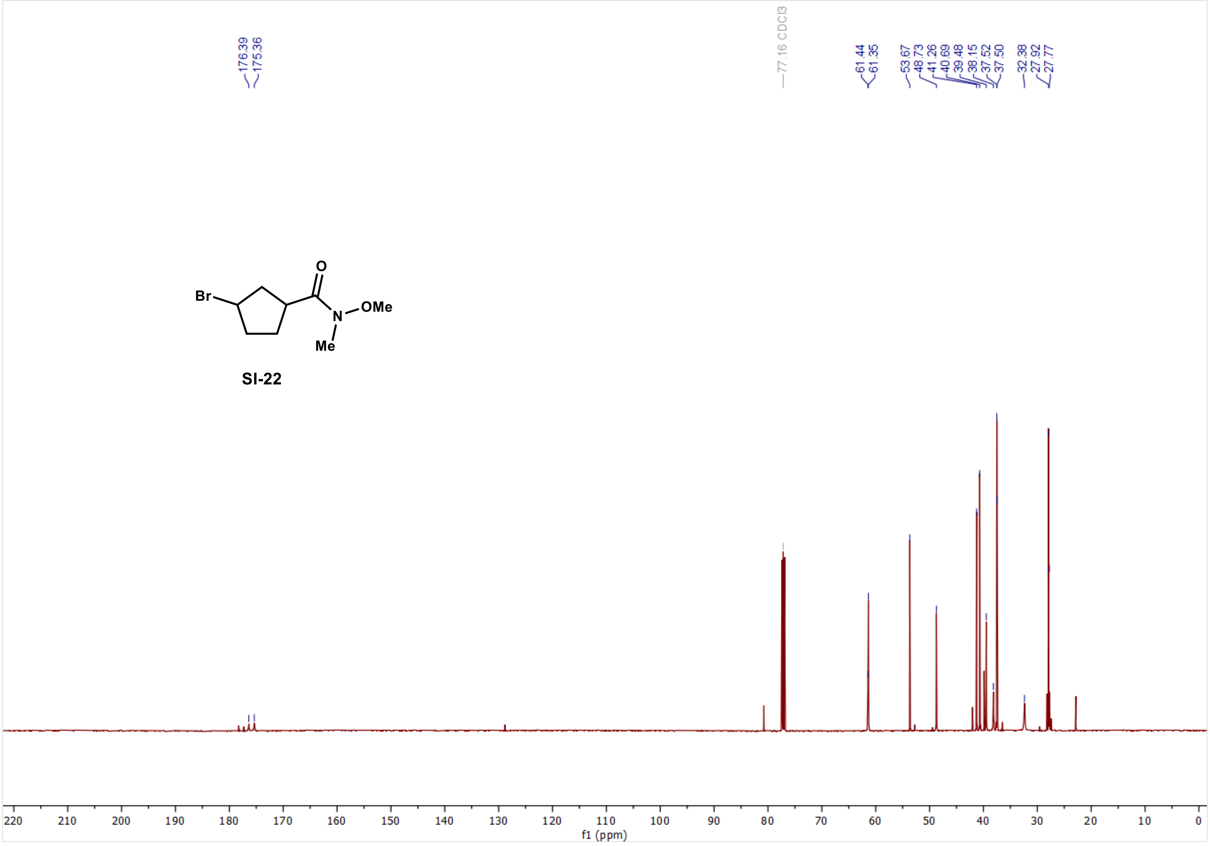

^{1}H NMR (400 MHz, Chloroform-*d*) of **SI-23 – minor diastereoisomer**:

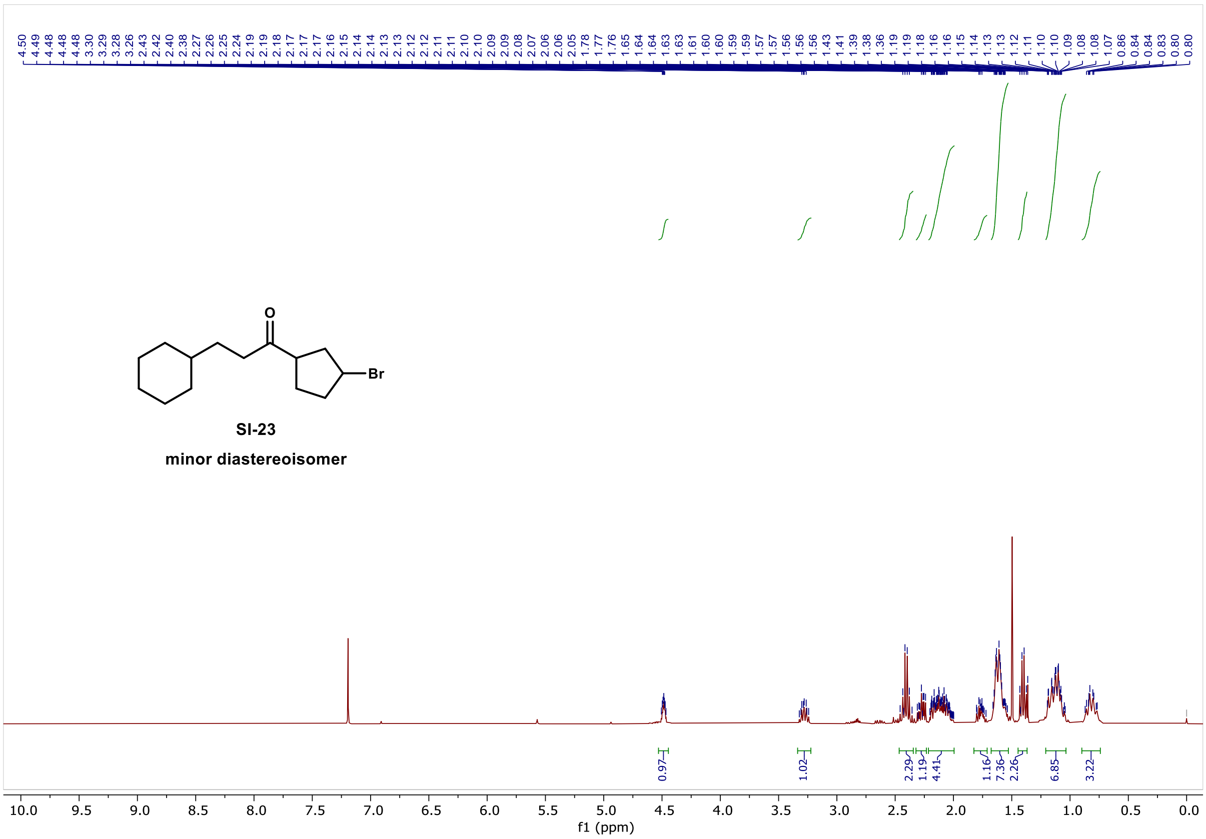

^{13}C NMR (101 MHz, Chloroform-*d*) of **SI-23 – minor diastereoisomer**:

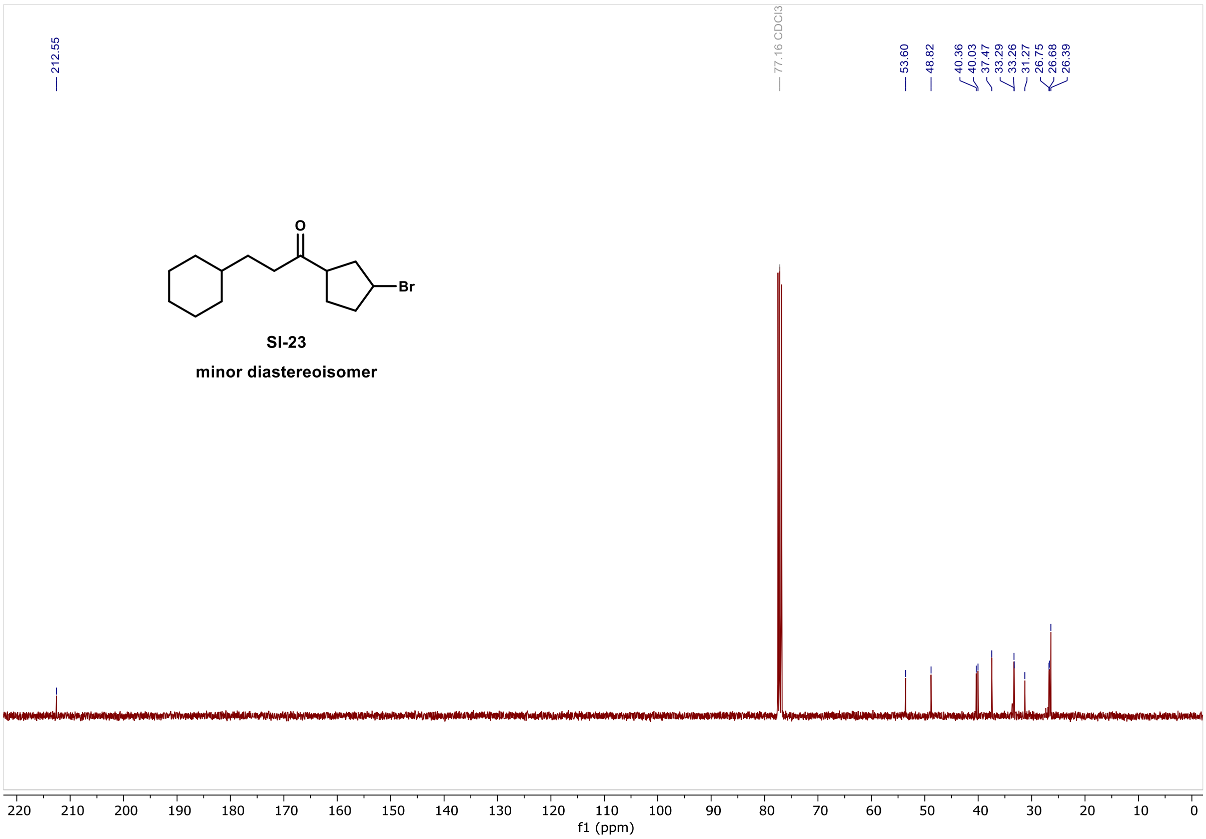

^{1}H NMR (400 MHz, Chloroform-*d*) of **SI-23 – major diastereoisomer**:

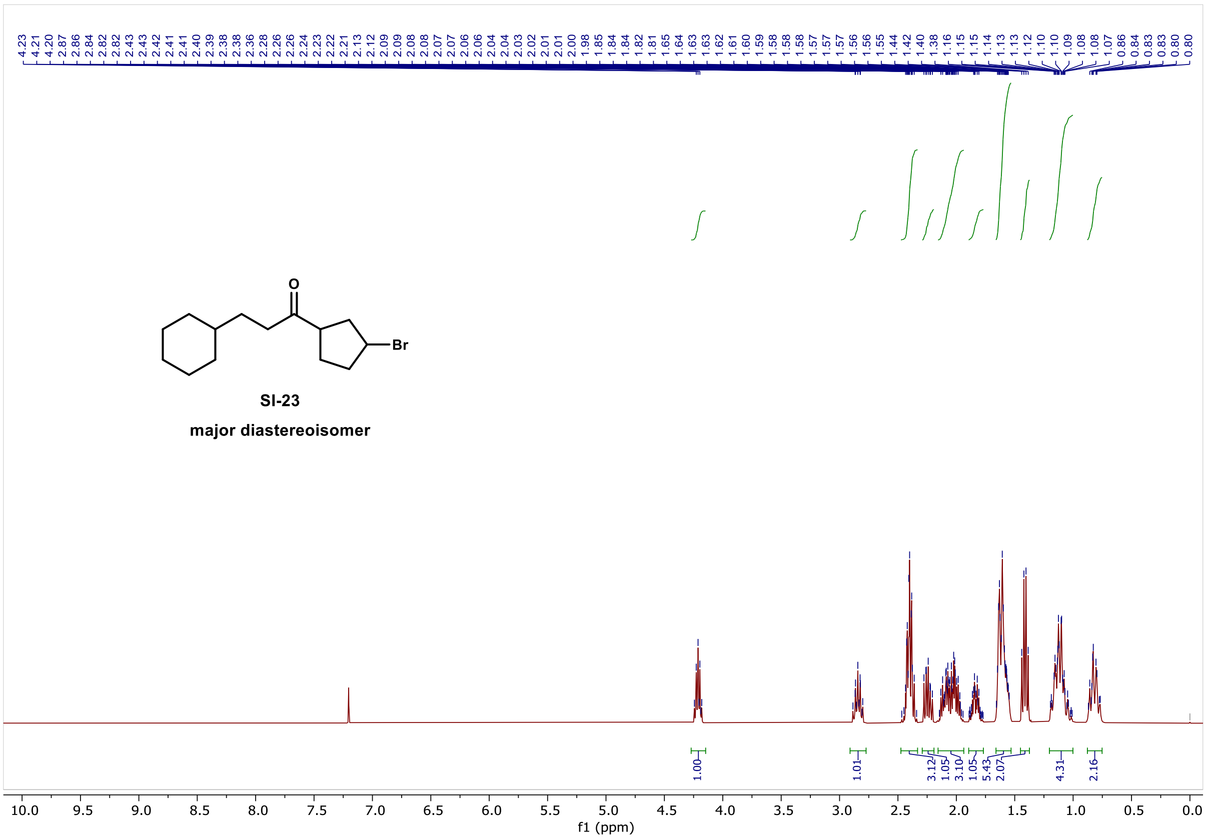

^{13}C NMR (101 MHz, Chloroform-*d*) of **SI-23 – major diastereoisomer**:

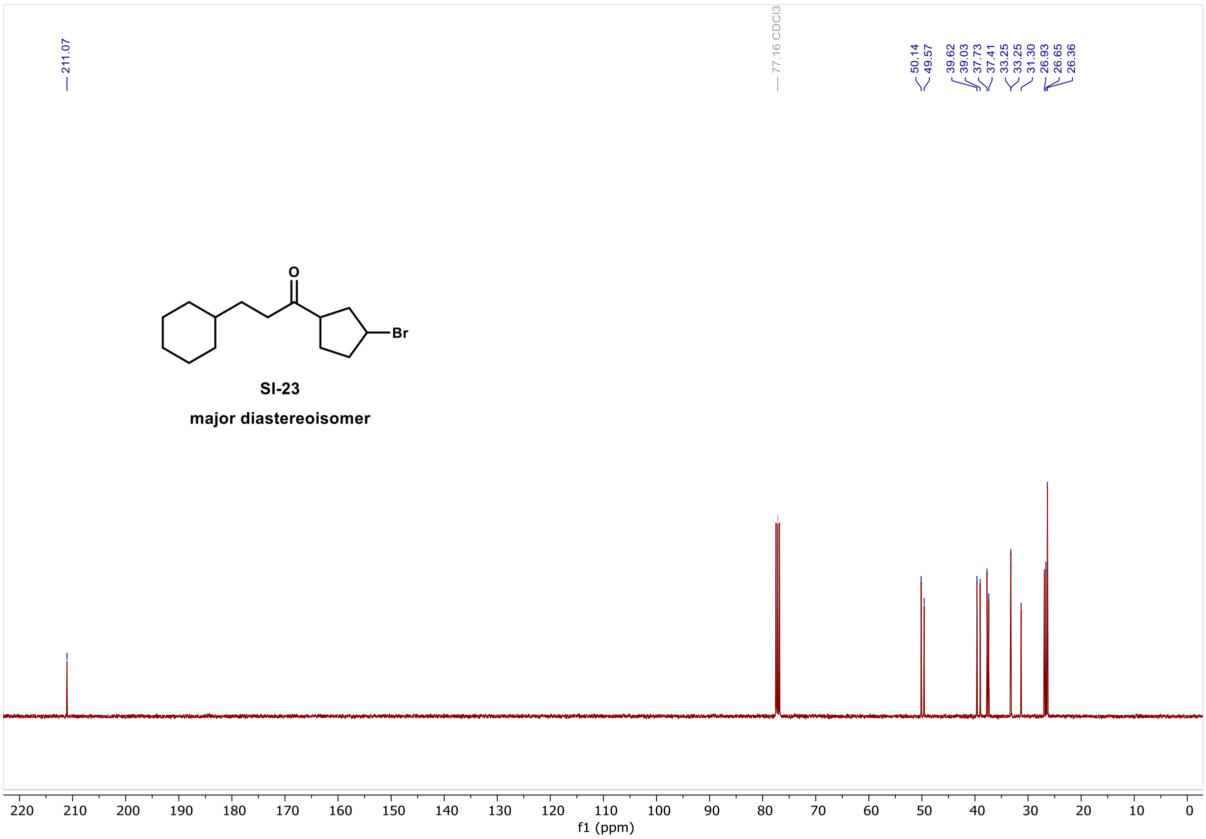

^{1}H NMR (500 MHz, Chloroform-*d*) of **1c**:

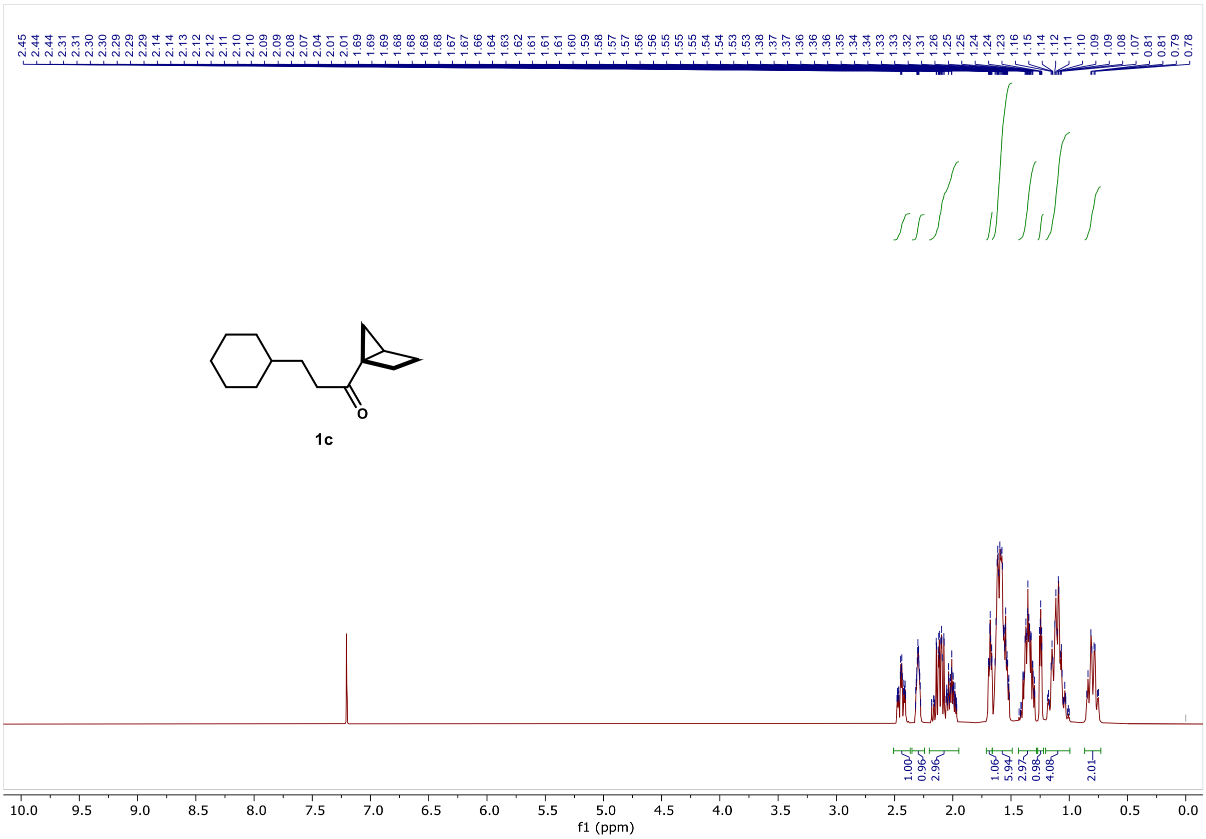

^{13}C NMR (126 MHz, Chloroform-*d*) of **1c**:

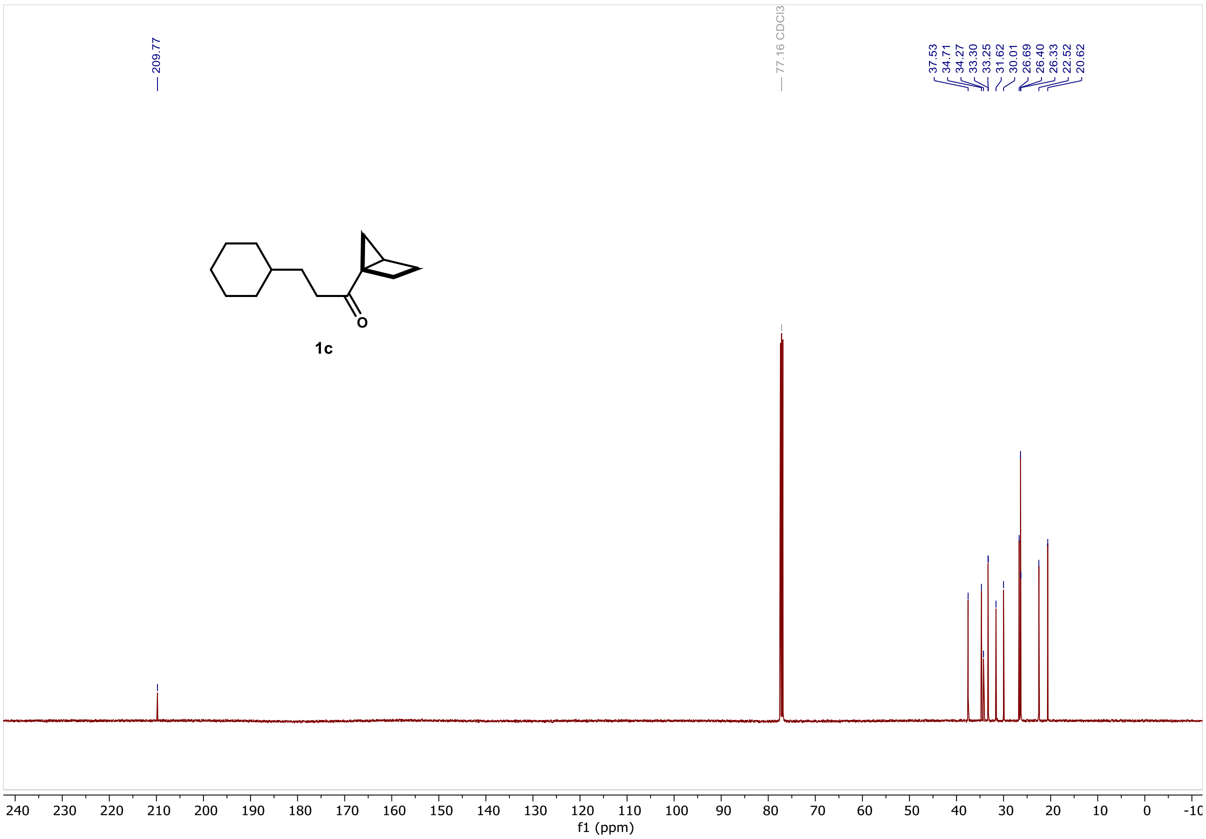

^{1}H NMR (400 MHz, Chloroform-*d*) of **SI-24 – major diastereoisomer**:

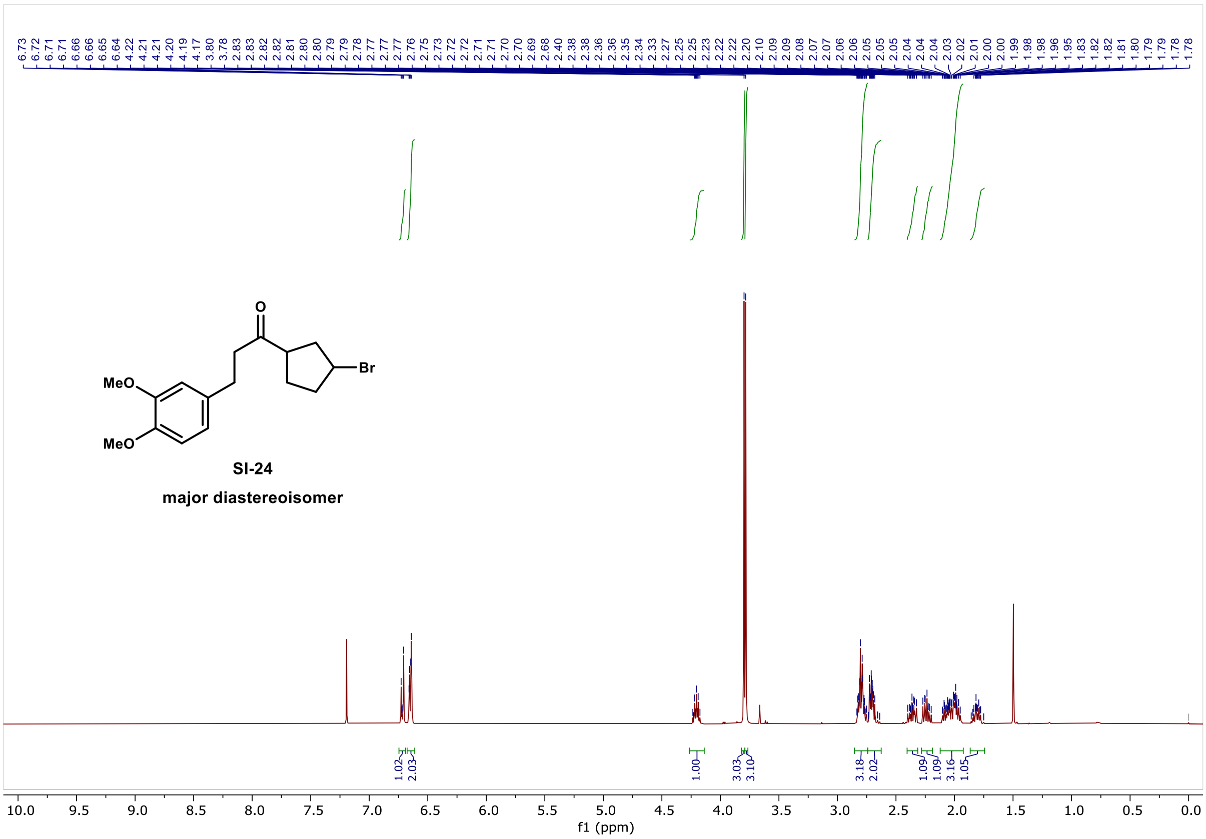

^{13}C NMR (101 MHz, Chloroform-*d*) of **SI-24 – major diastereoisomer**:

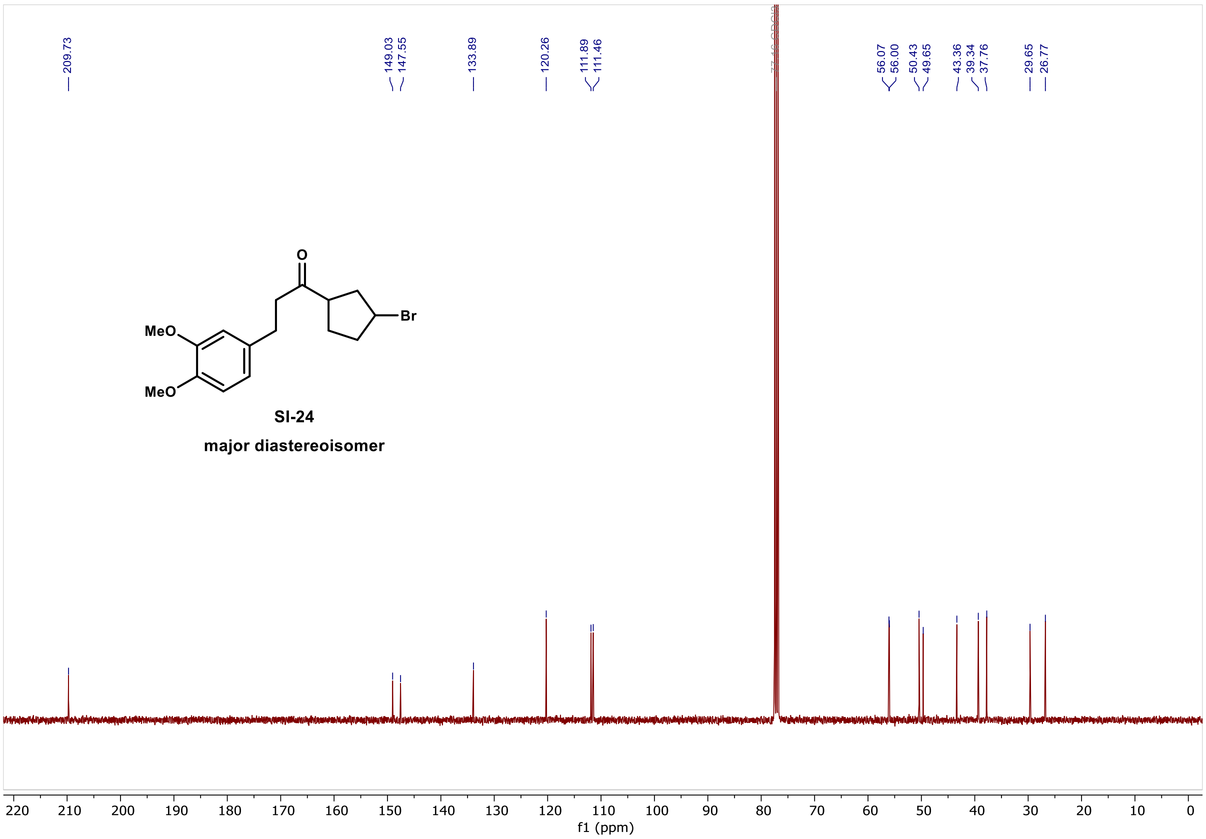

^{1}H NMR (500 MHz, Chloroform-*d*) of **1e**:

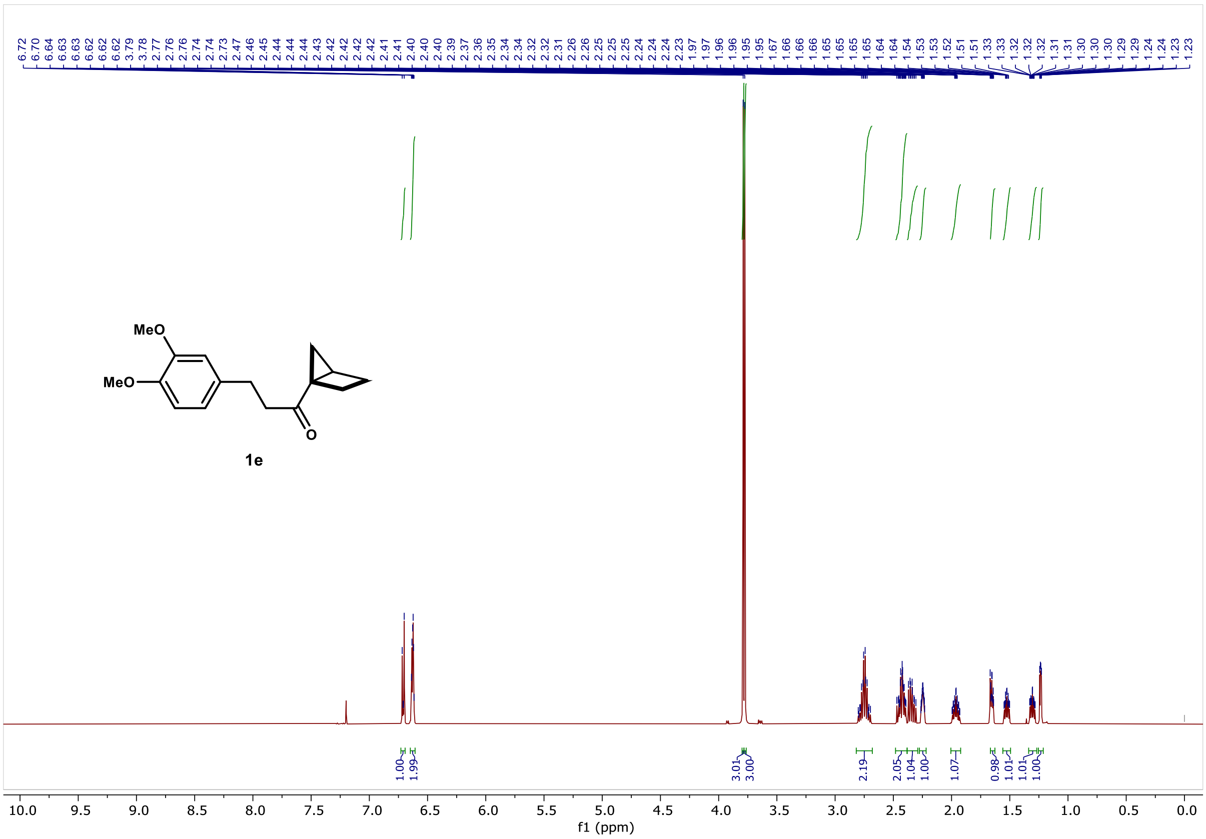

^{13}C NMR (126 MHz, Chloroform-*d*) of **1e**:

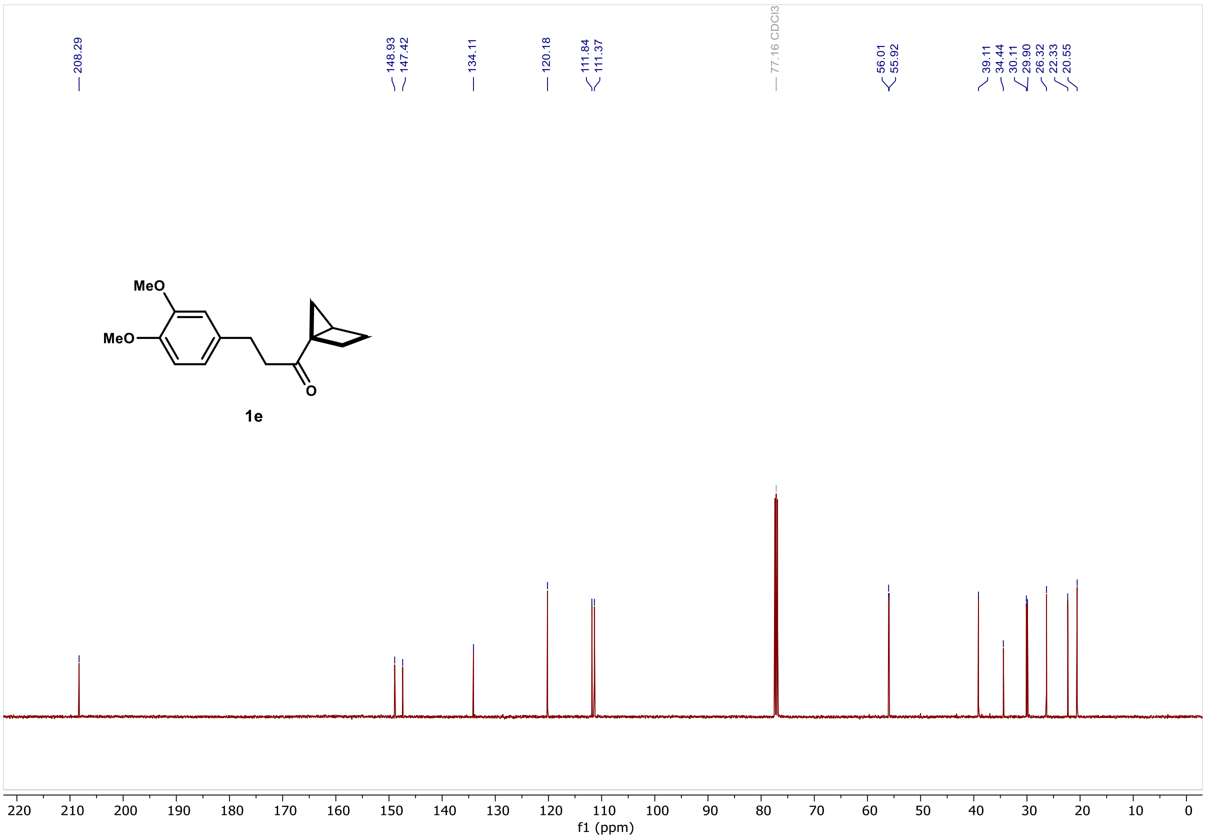

^{1}H NMR (400 MHz, Chloroform-*d*) of **SI-25 – minor diastereoisomer**:

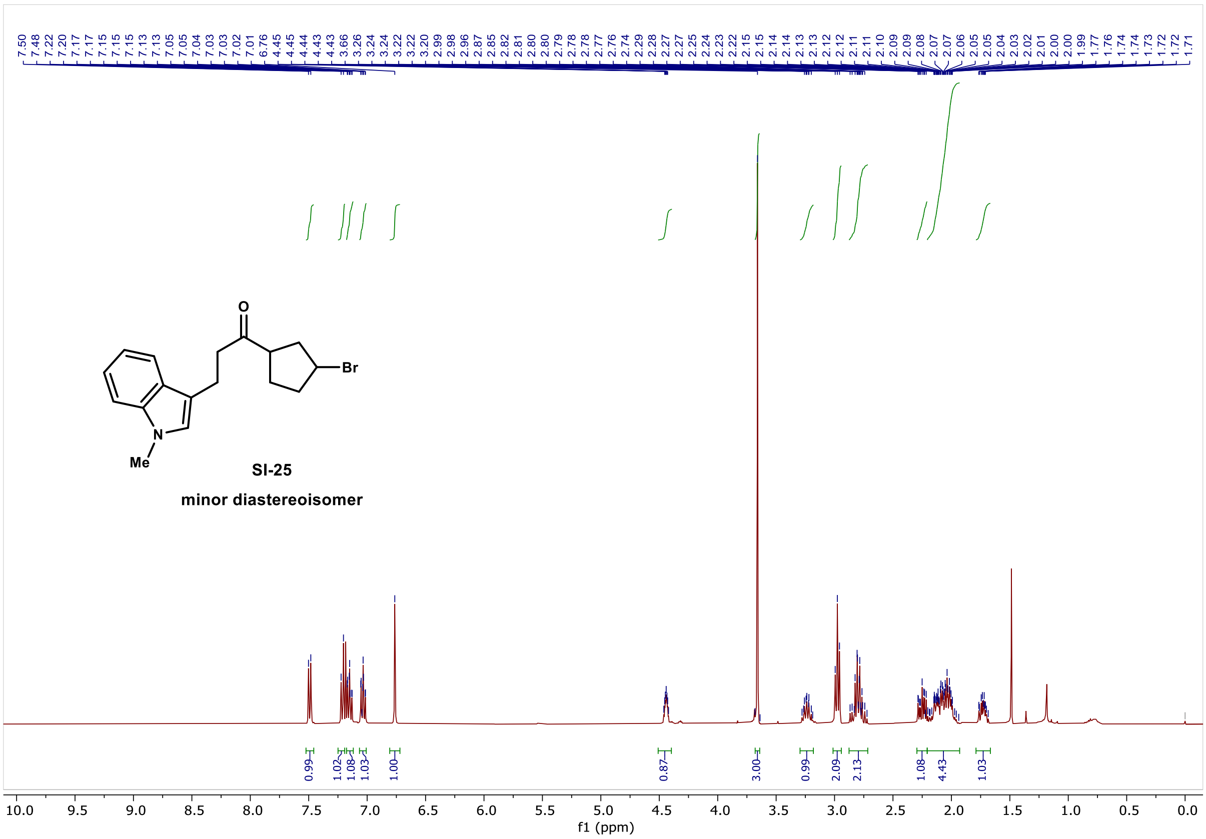

^{13}C NMR (101 MHz, Chloroform-*d*) of **SI-25 – minor diastereoisomer**:

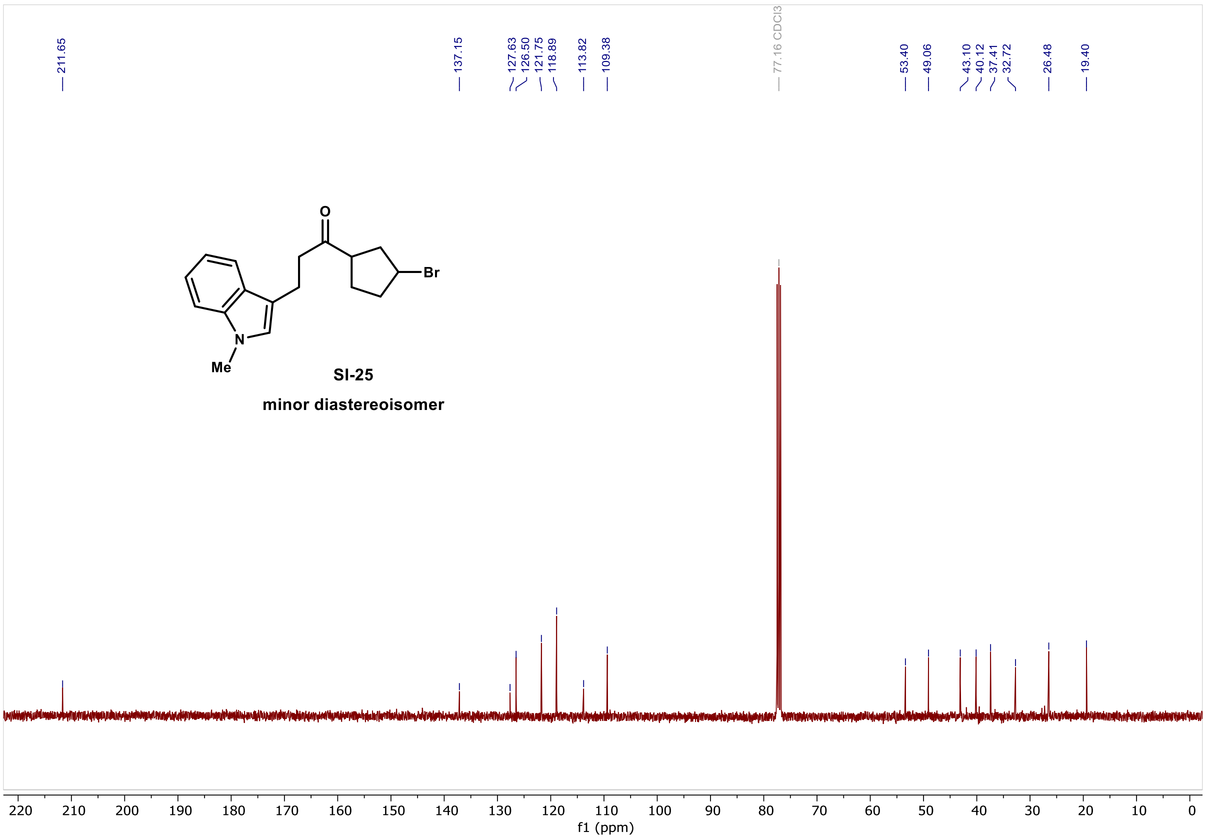

^{1}H NMR (400 MHz, Chloroform-*d*) of **SI-25 – major diastereoisomer**:

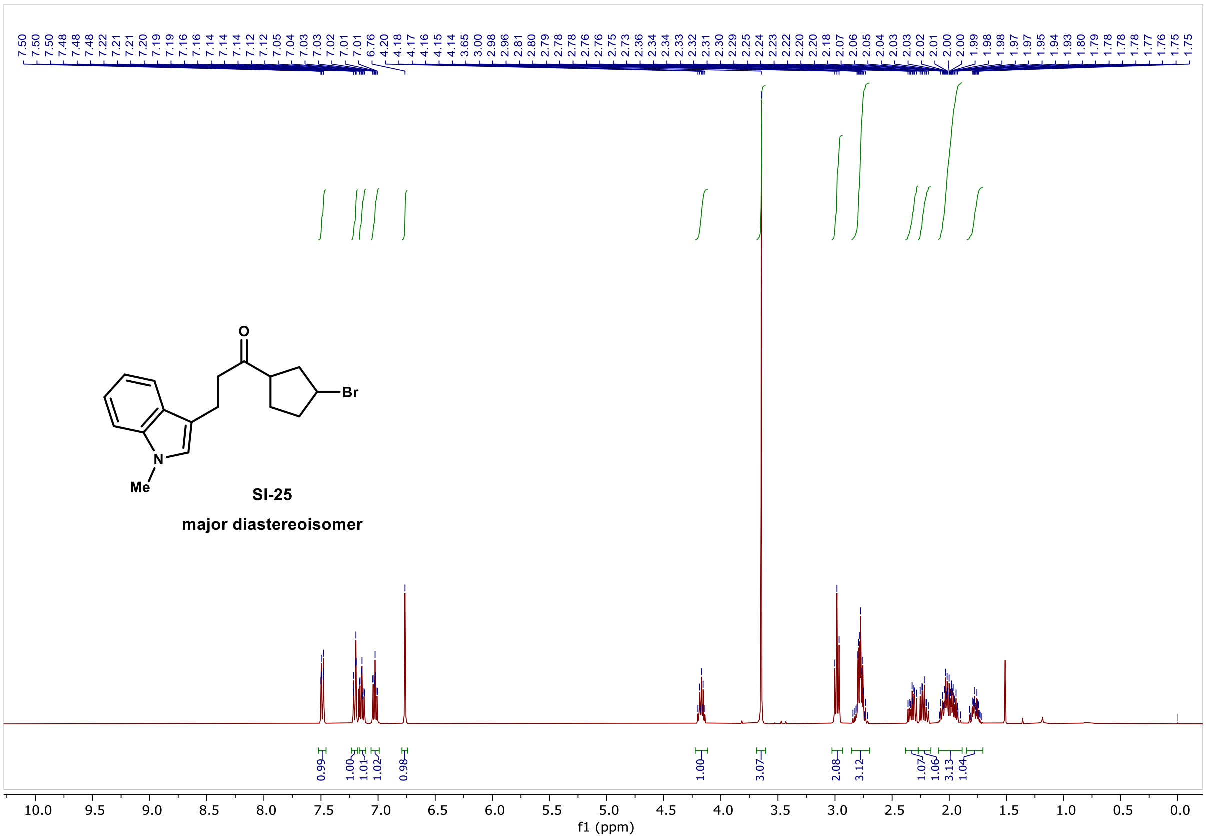

^{13}C NMR (101 MHz, Chloroform-*d*) of **SI-25 – major diastereoisomer**:

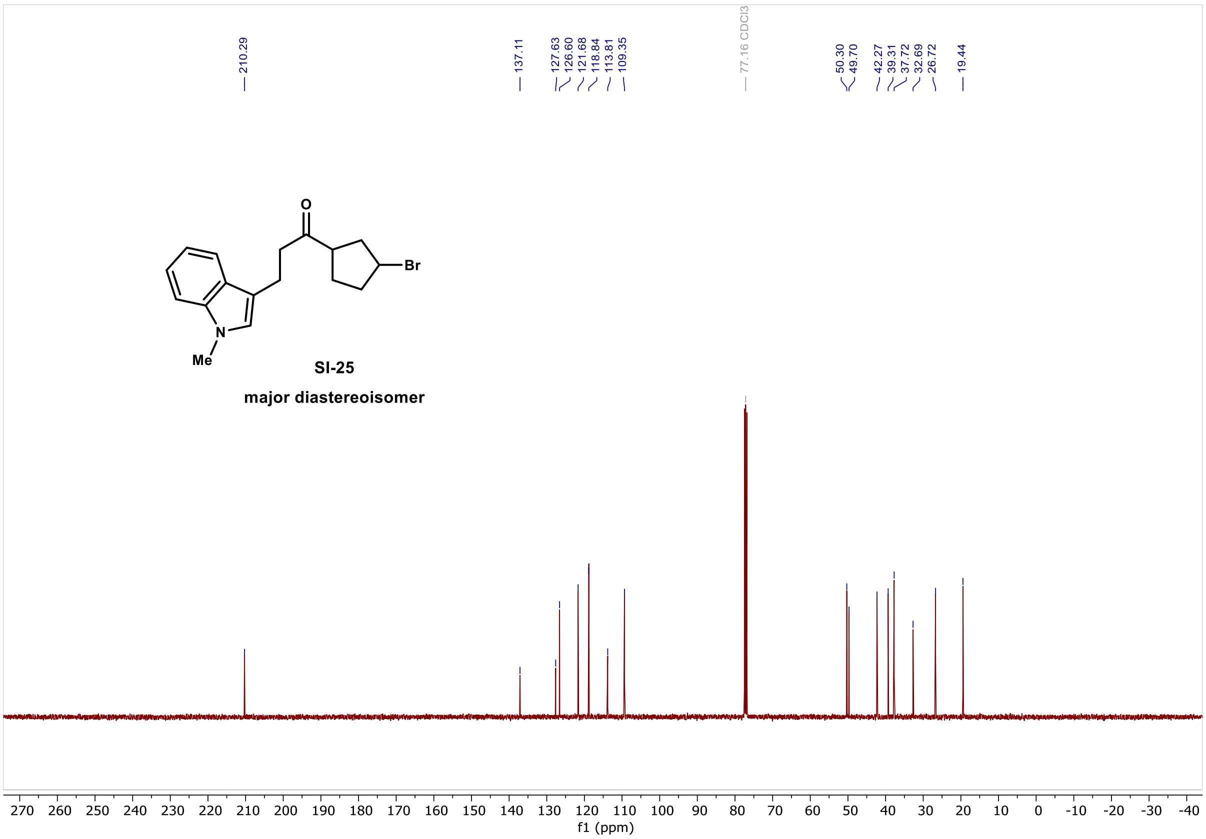

^{1}H NMR (500 MHz, Chloroform-*d*) of **1f**:

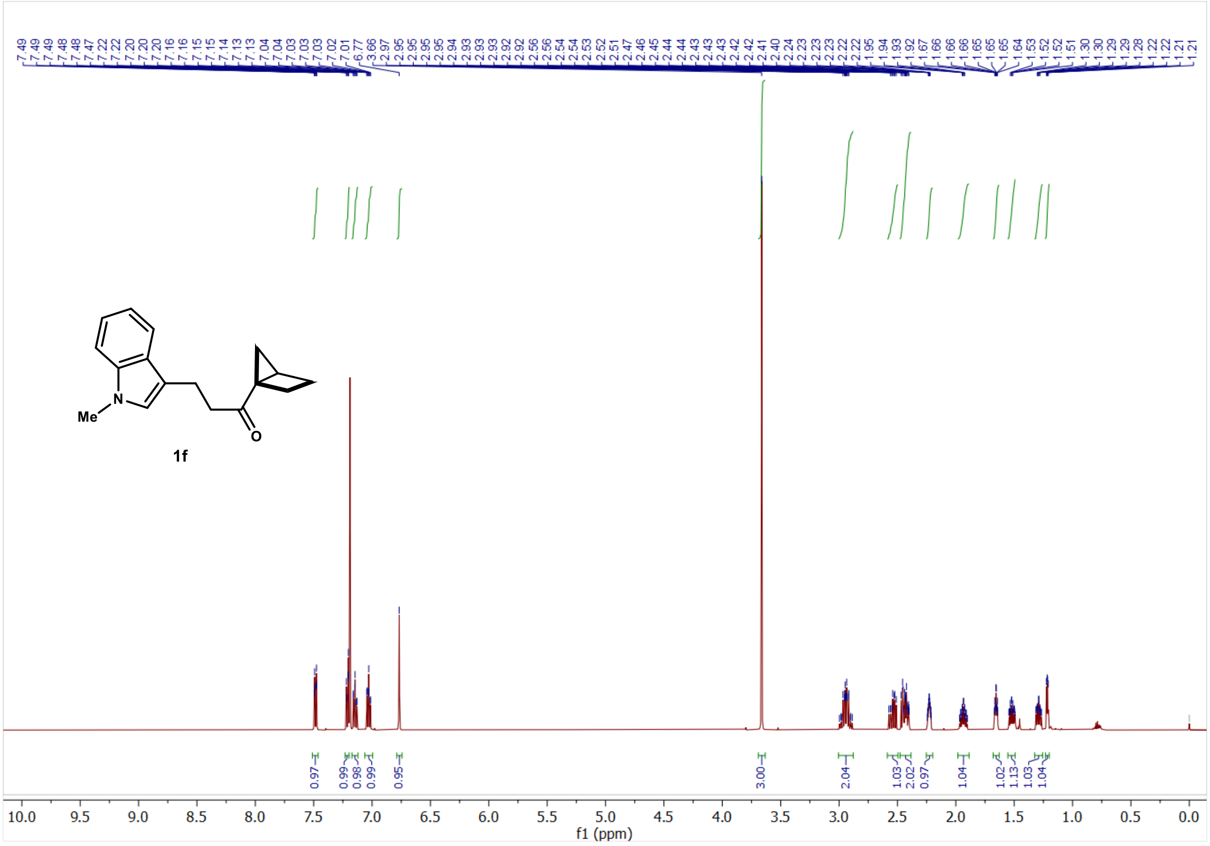

^{13}C NMR (126 MHz, Chloroform-*d*) of **1f**:

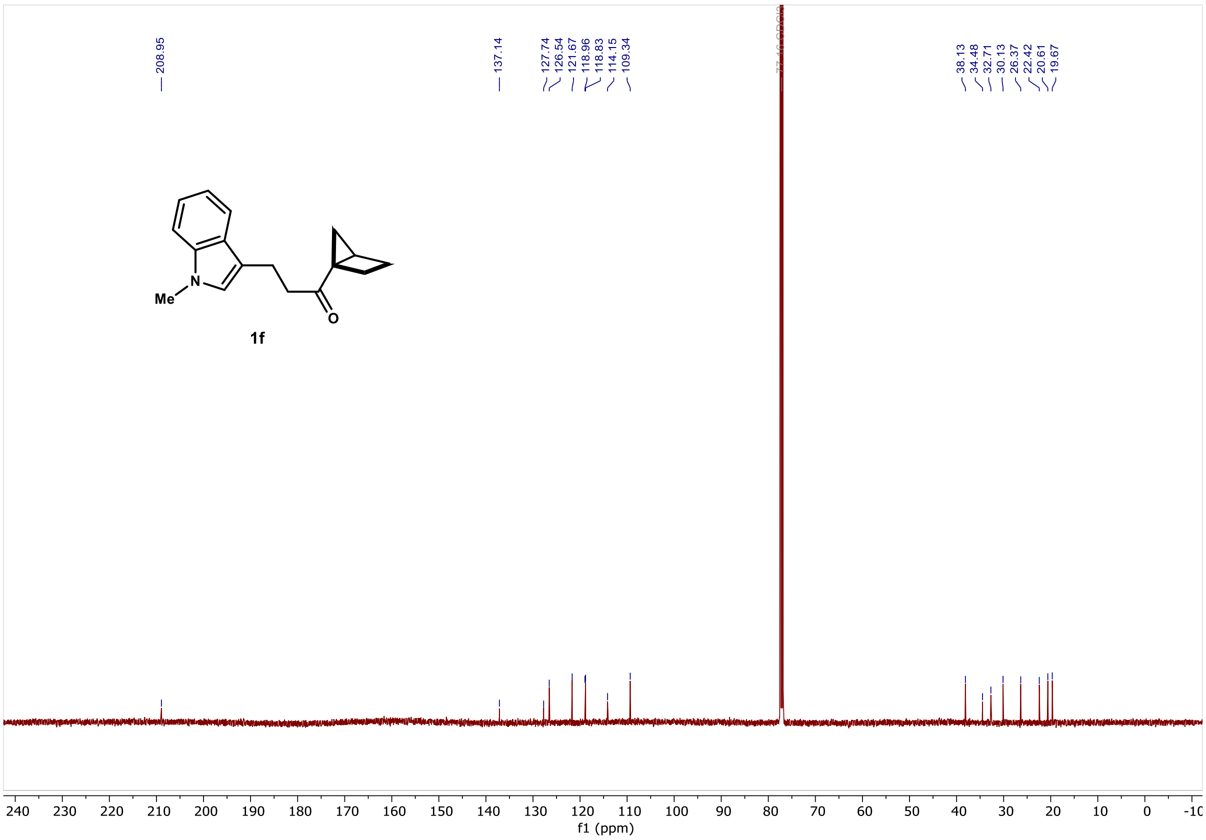

^{1}H NMR (400 MHz, Chloroform-*d*) of **SI-26 – minor diastereoisomer**:

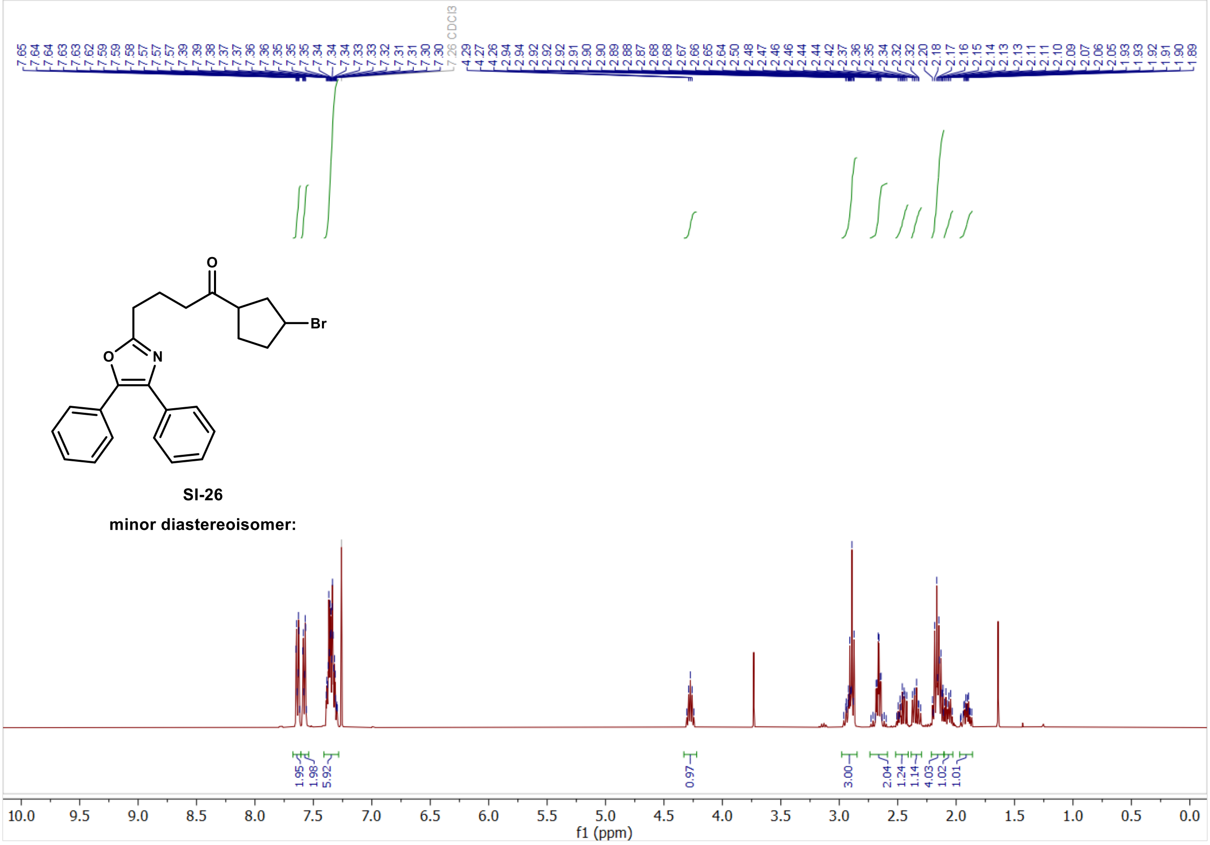

^{13}C NMR (101 MHz, Chloroform-*d*) of **SI-26 – minor diastereoisomer**:

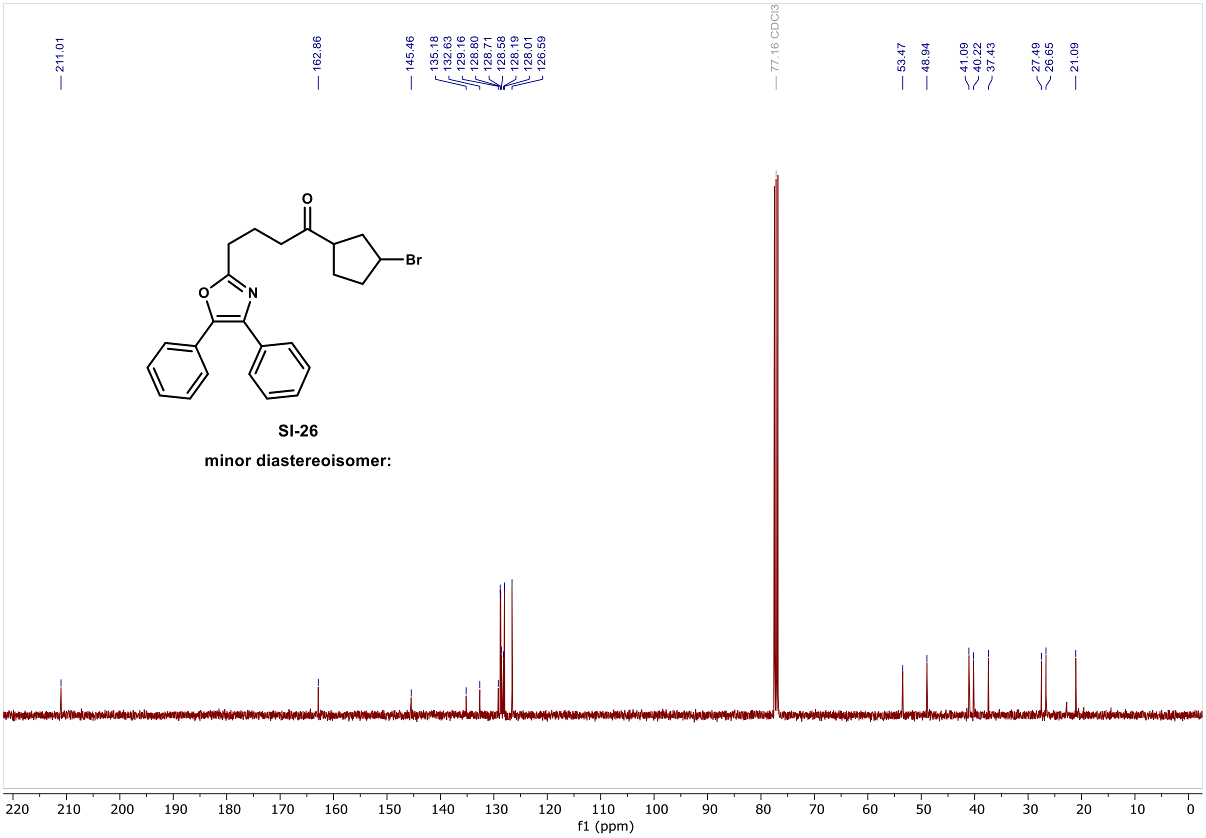

^{1}H NMR (400 MHz, Chloroform-*d*) of **SI-26 – major diastereoisomer**:

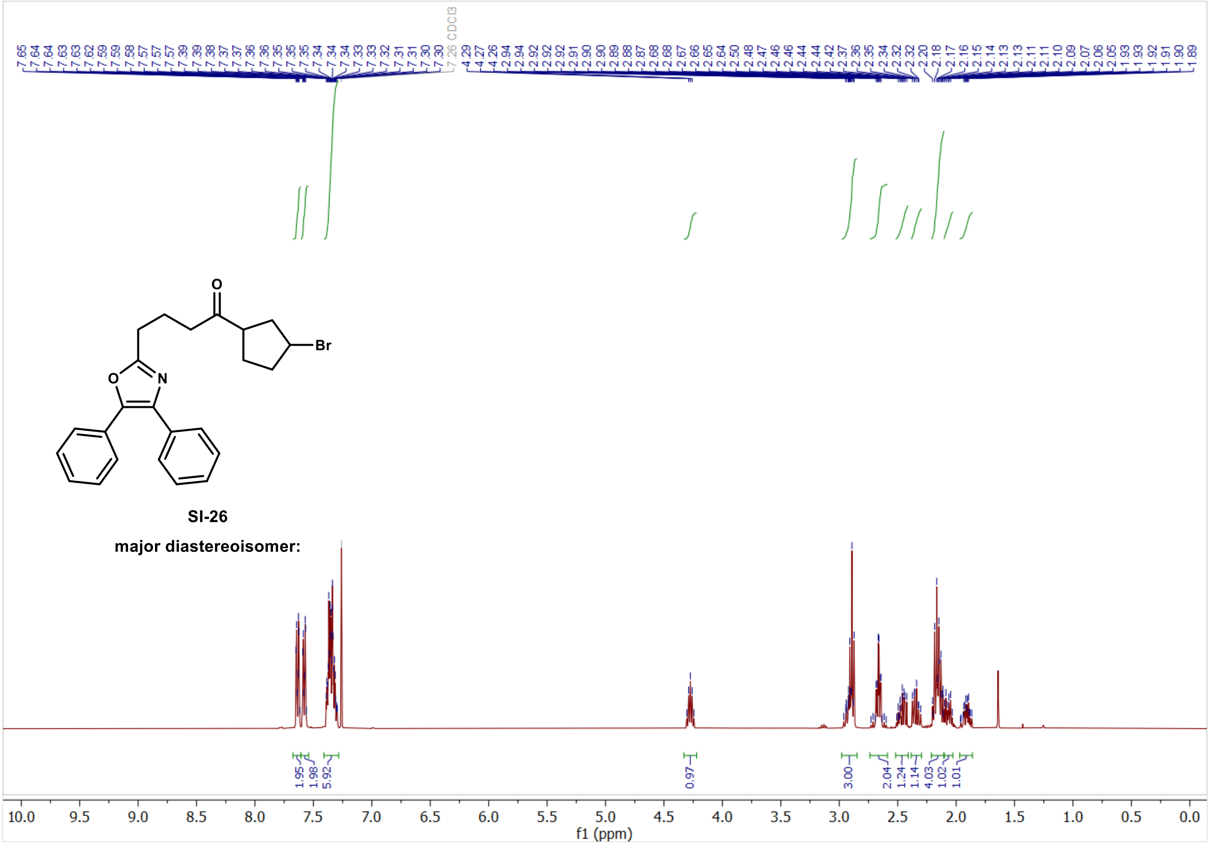

^{13}C NMR (101 MHz, Chloroform-*d*) of **SI-26 – major diastereoisomer**:

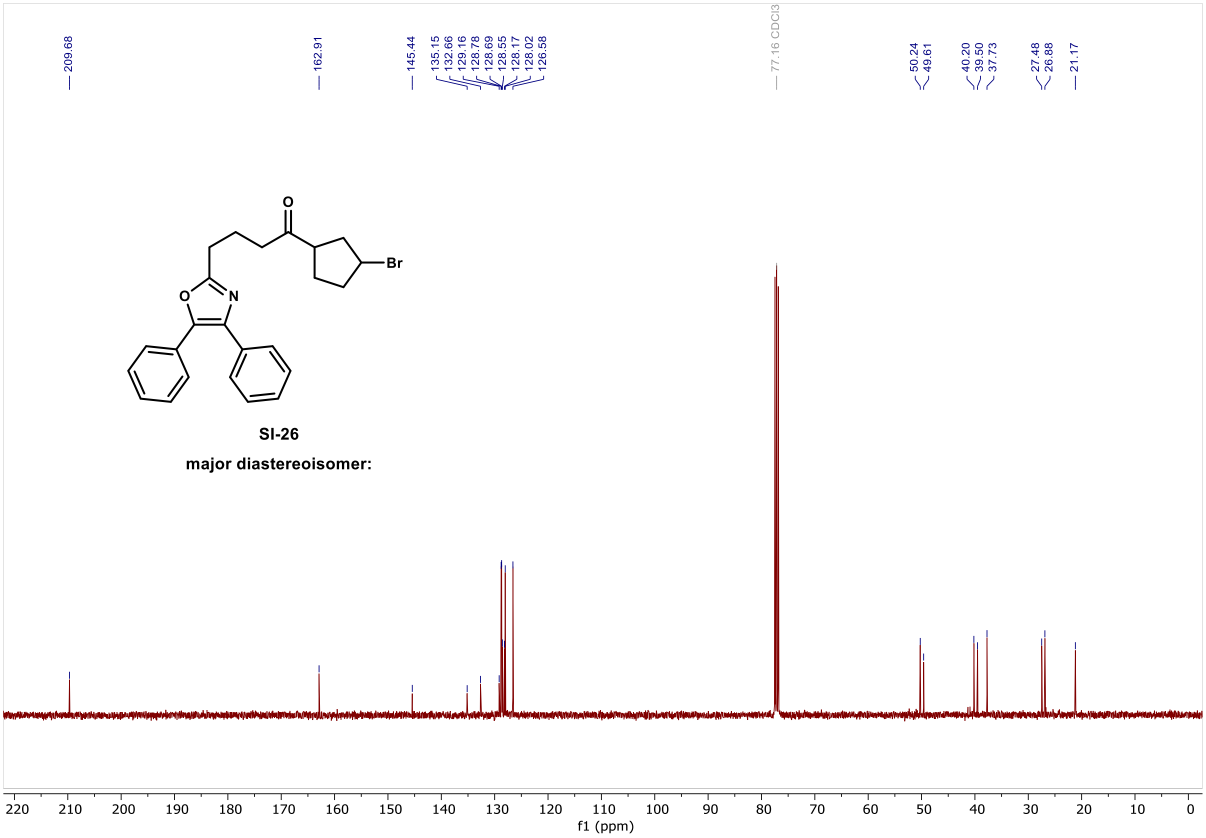

^{1}H NMR (400 MHz, Chloroform-*d*) of **1g**:

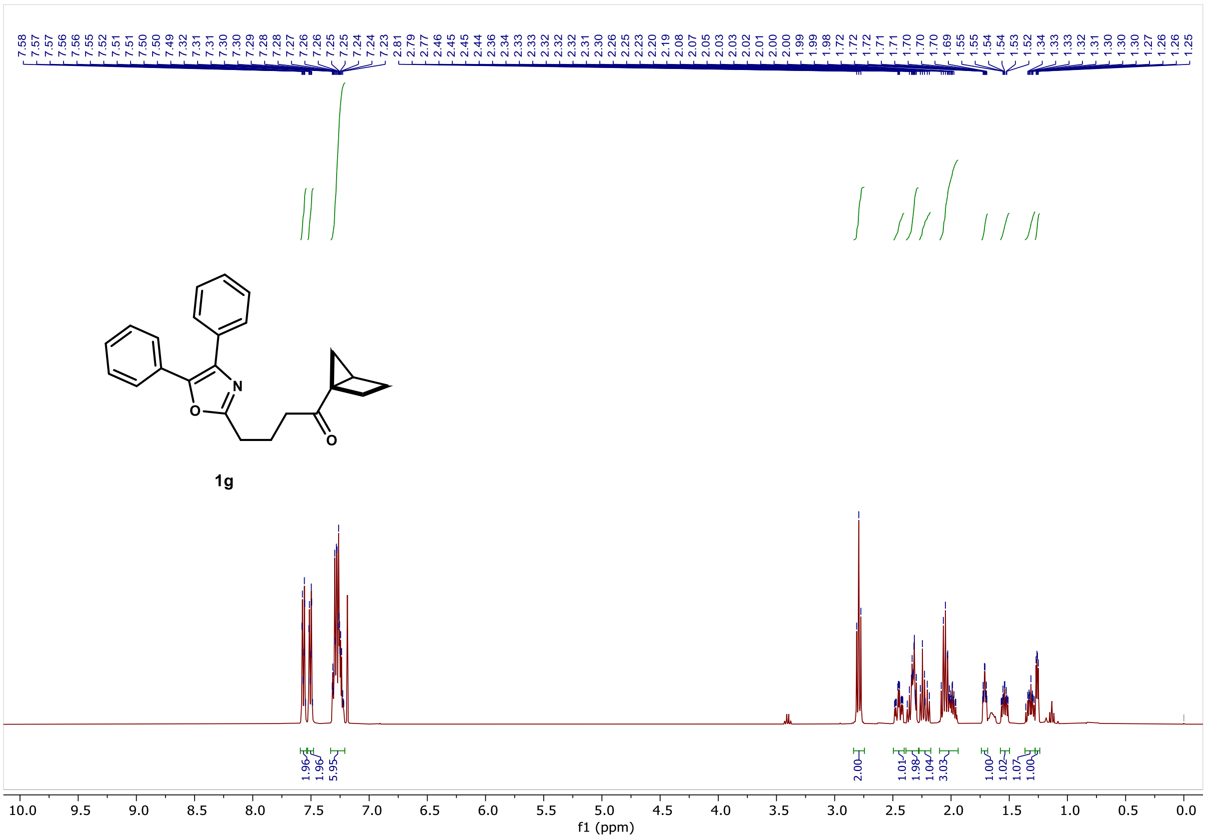

^{13}C NMR (101 MHz, Chloroform-*d*) of **1g**:

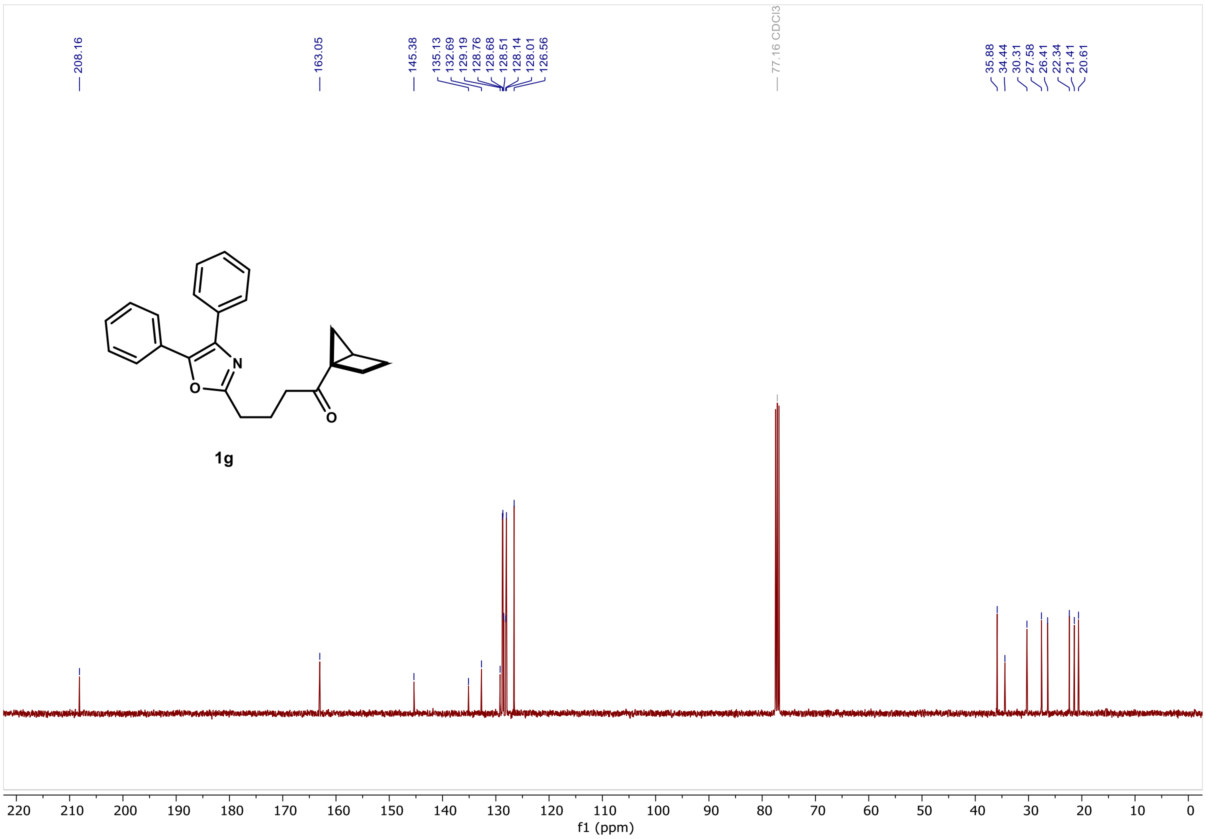

^{1}H NMR (400 MHz, Chloroform-*d*) of **SI-27 – mixture of diastereoisomers**:

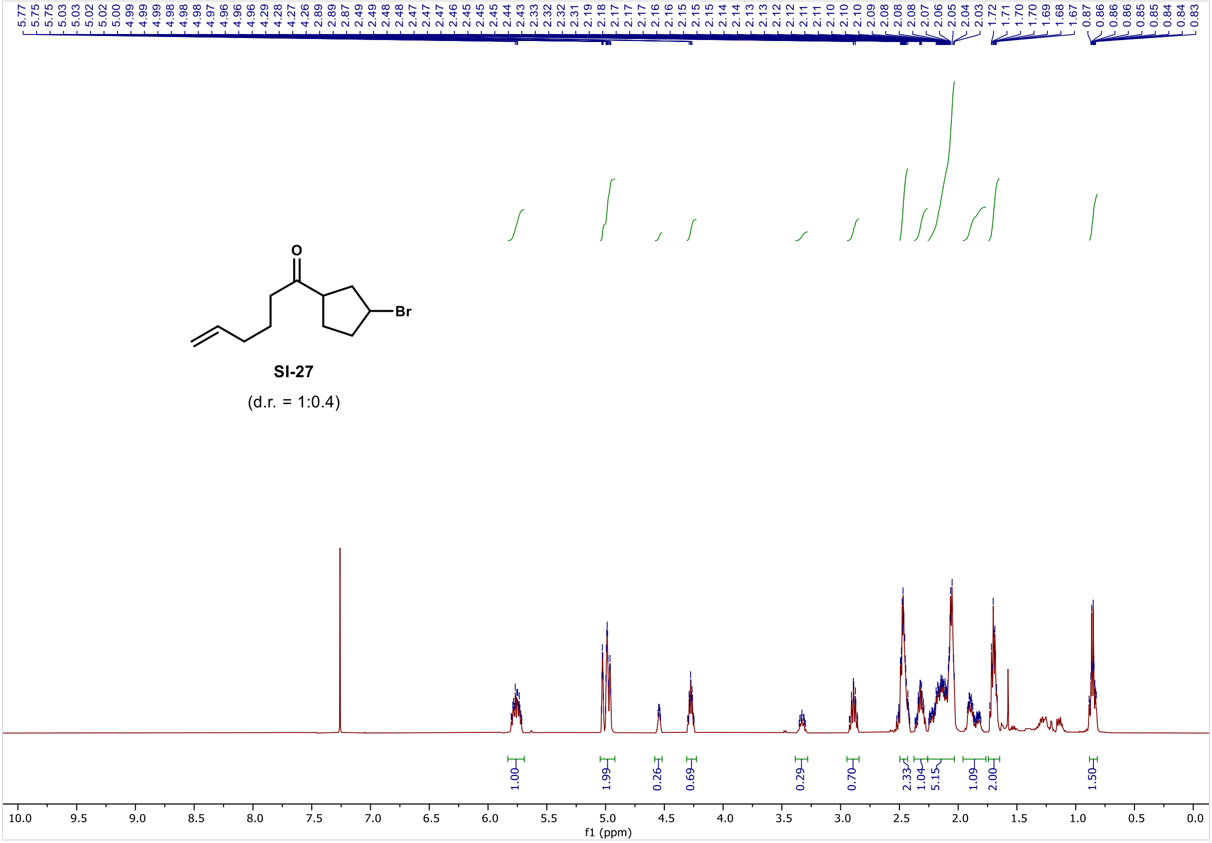

SI-27

(d.r. = 1:0.4)

^{13}C NMR (101 MHz, Chloroform-*d*) of **SI-27 – mixture of diastereoisomers**:

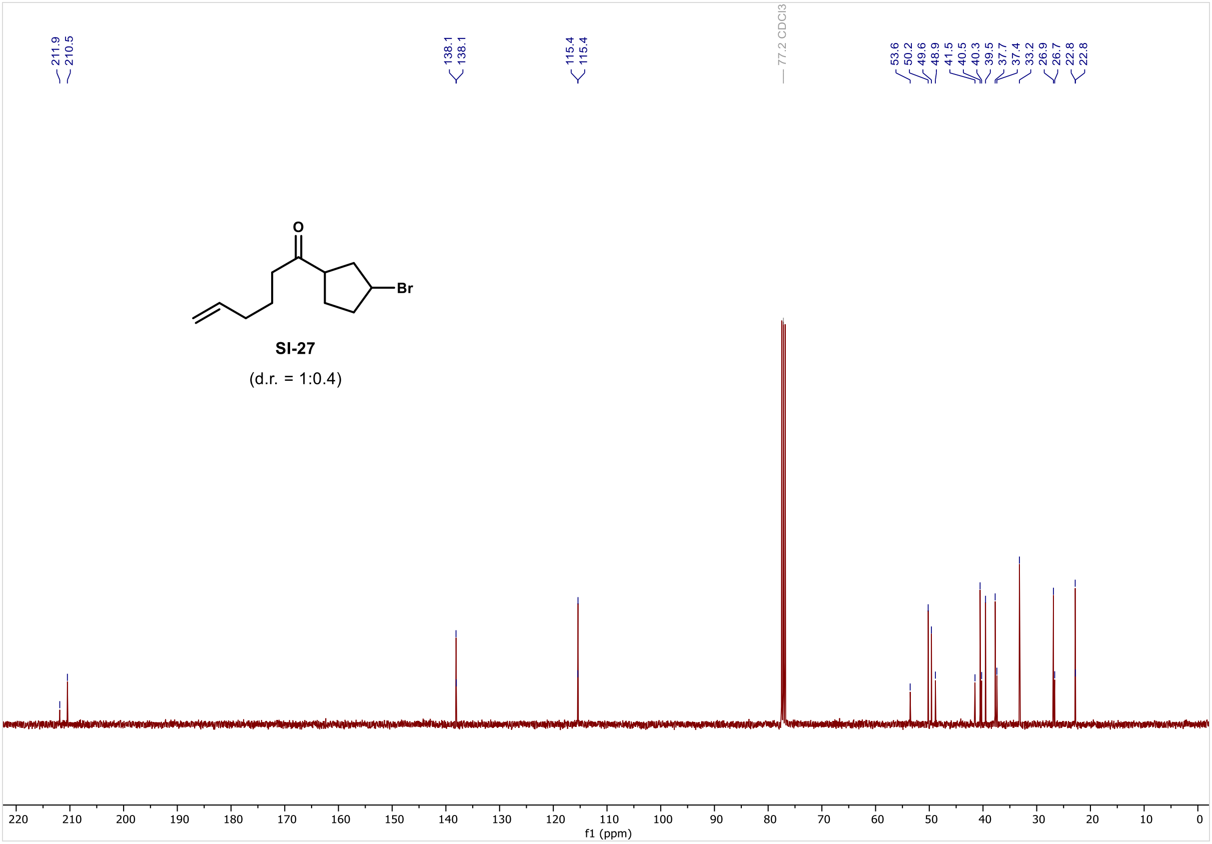

SI-27

(d.r. = 1:0.4)

^{1}H NMR (500 MHz, Chloroform-*d*) of **1h**:

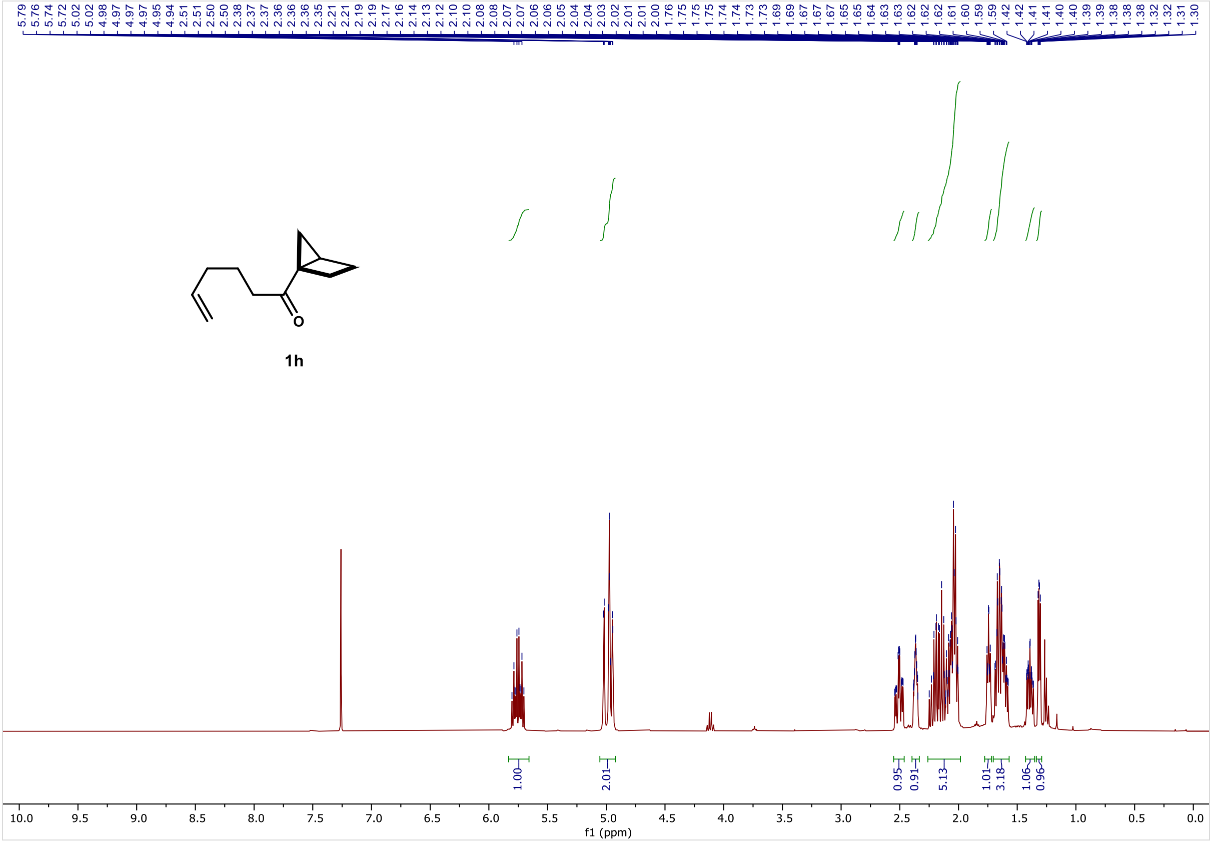

^{13}C NMR (126 MHz, Chloroform-*d*) of **1h**:

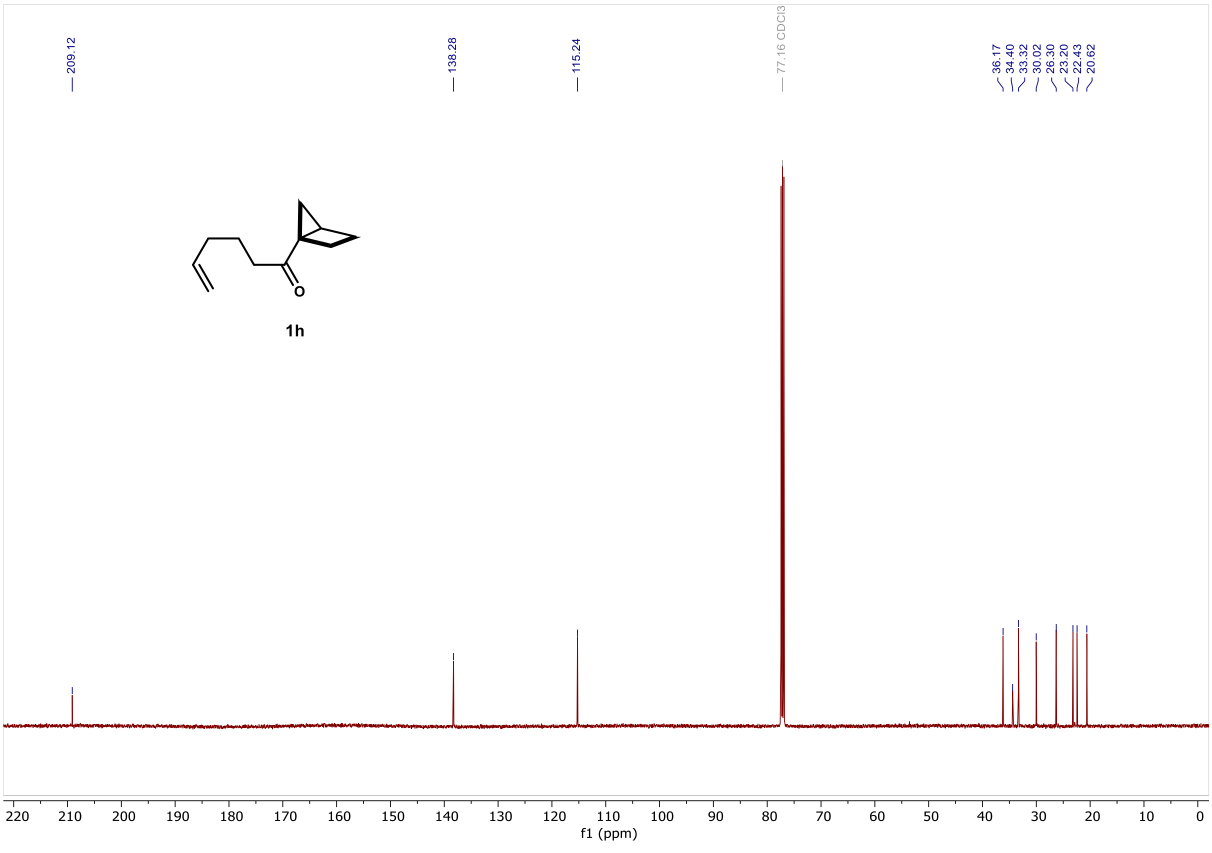

^{1}H NMR (400 MHz, Chloroform-*d*) of **SI-28 – minor diastereoisomer**:

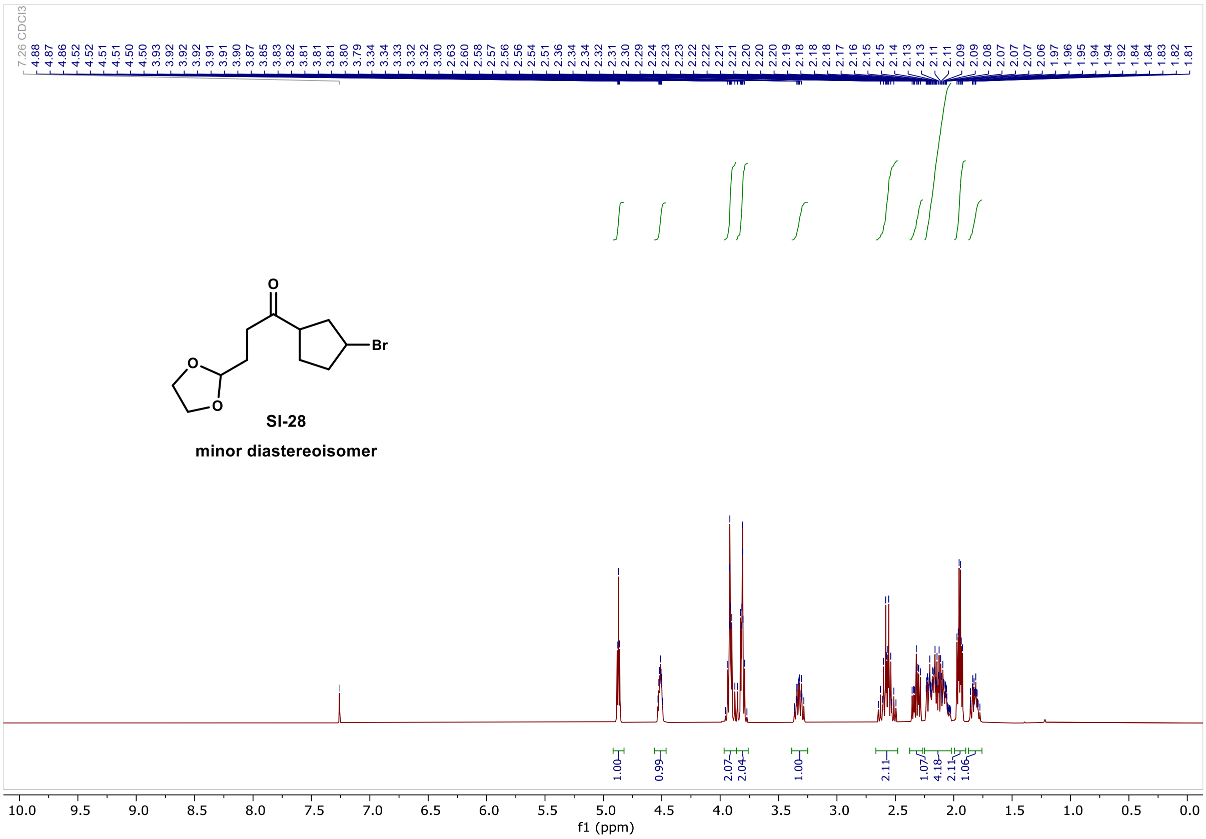

^{13}C NMR (101 MHz, Chloroform-*d*) of **SI-28 – minor diastereoisomer**:

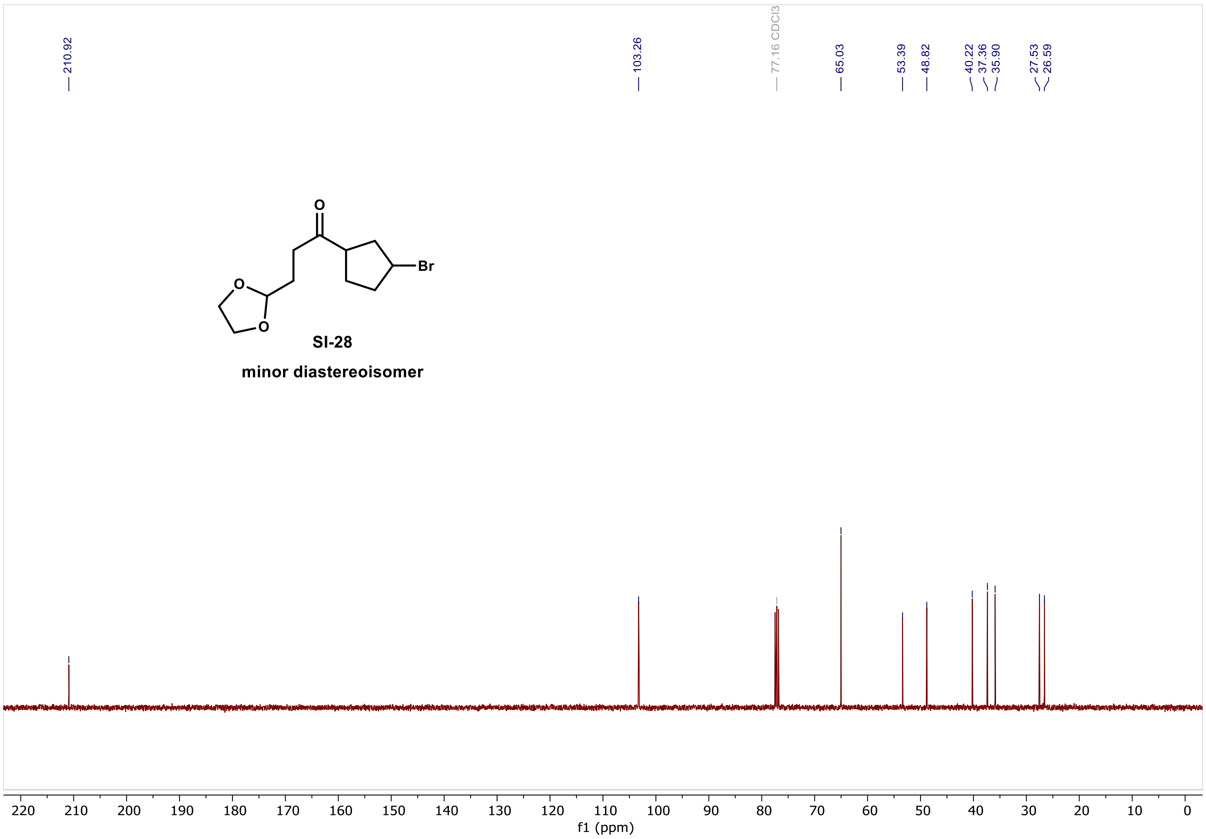

^{1}H NMR (400 MHz, Chloroform-*d*) of **SI-28 – major diastereoisomer**:

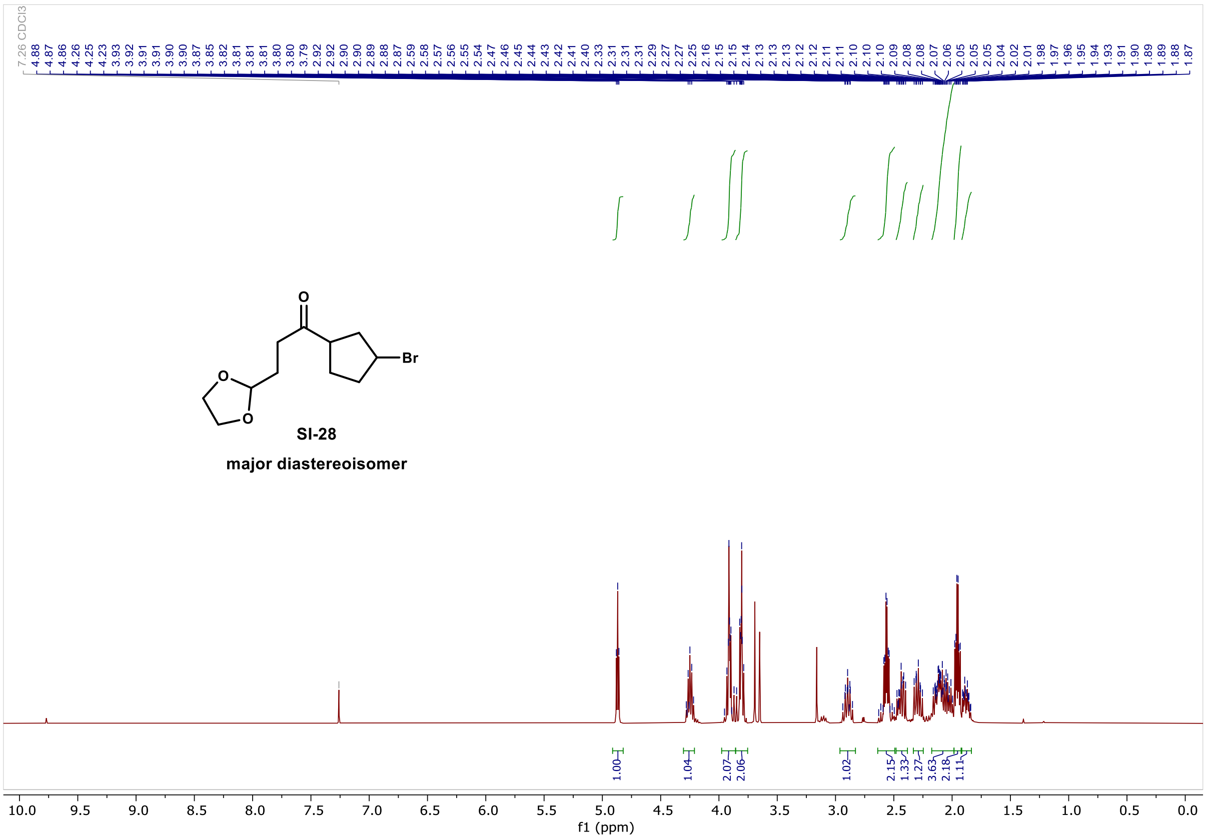

^{13}C NMR (101 MHz, Chloroform-*d*) of **SI-28 – major diastereoisomer**:

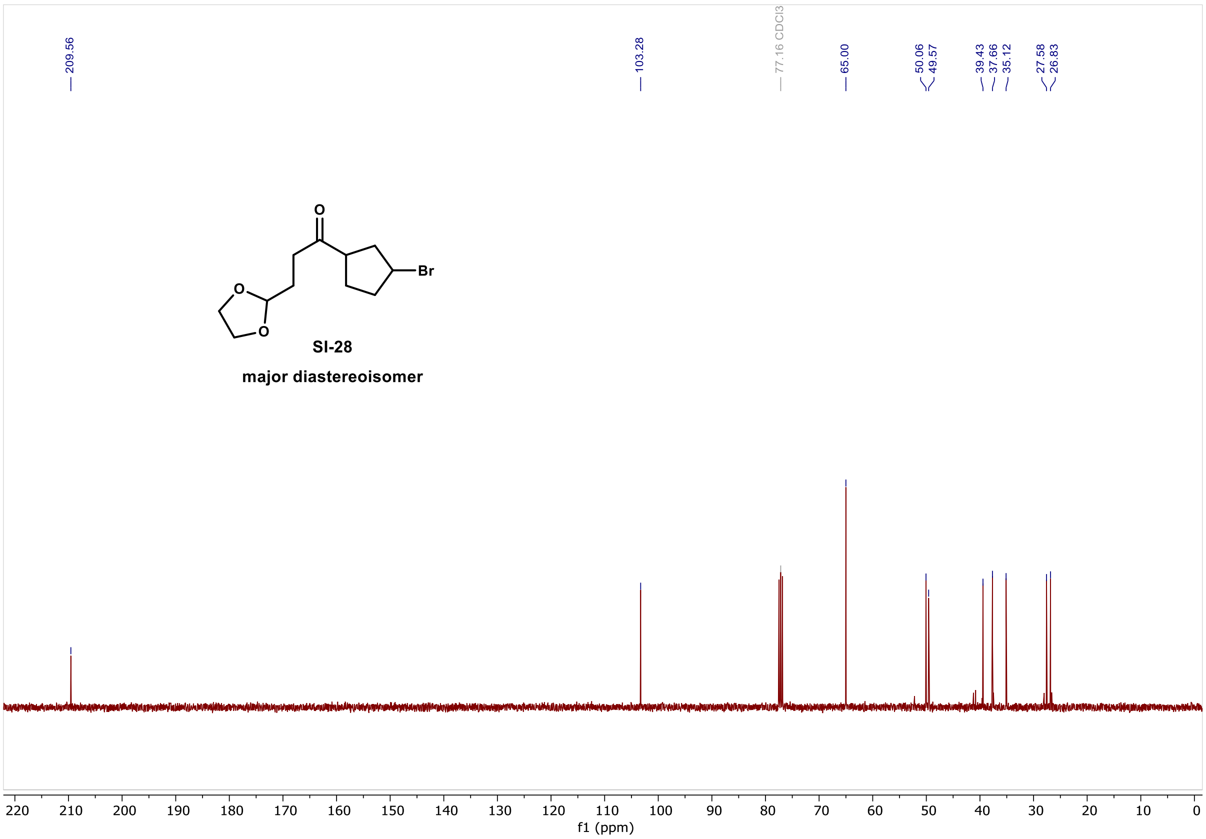

^{1}H NMR (400 MHz, Chloroform-*d*) of **1i**:

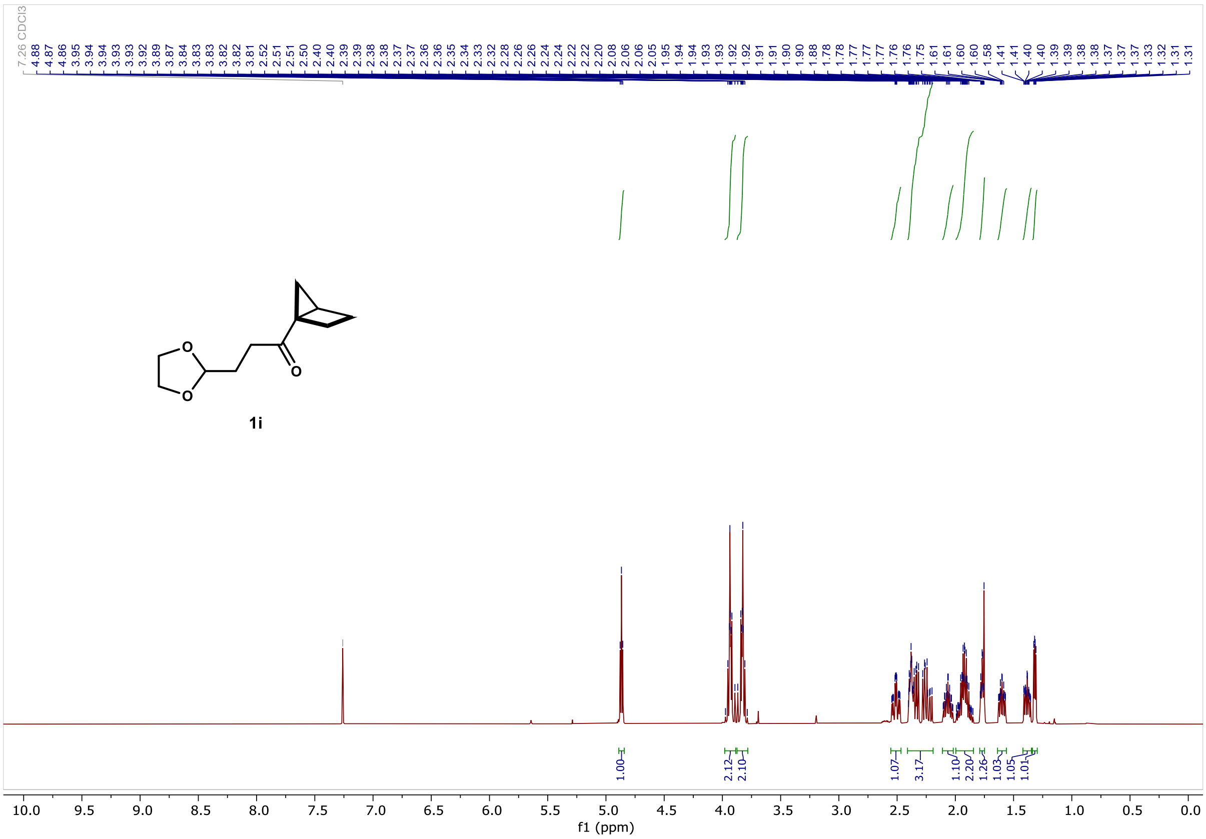

^{13}C NMR (101 MHz, Chloroform-*d*) of **1i**:

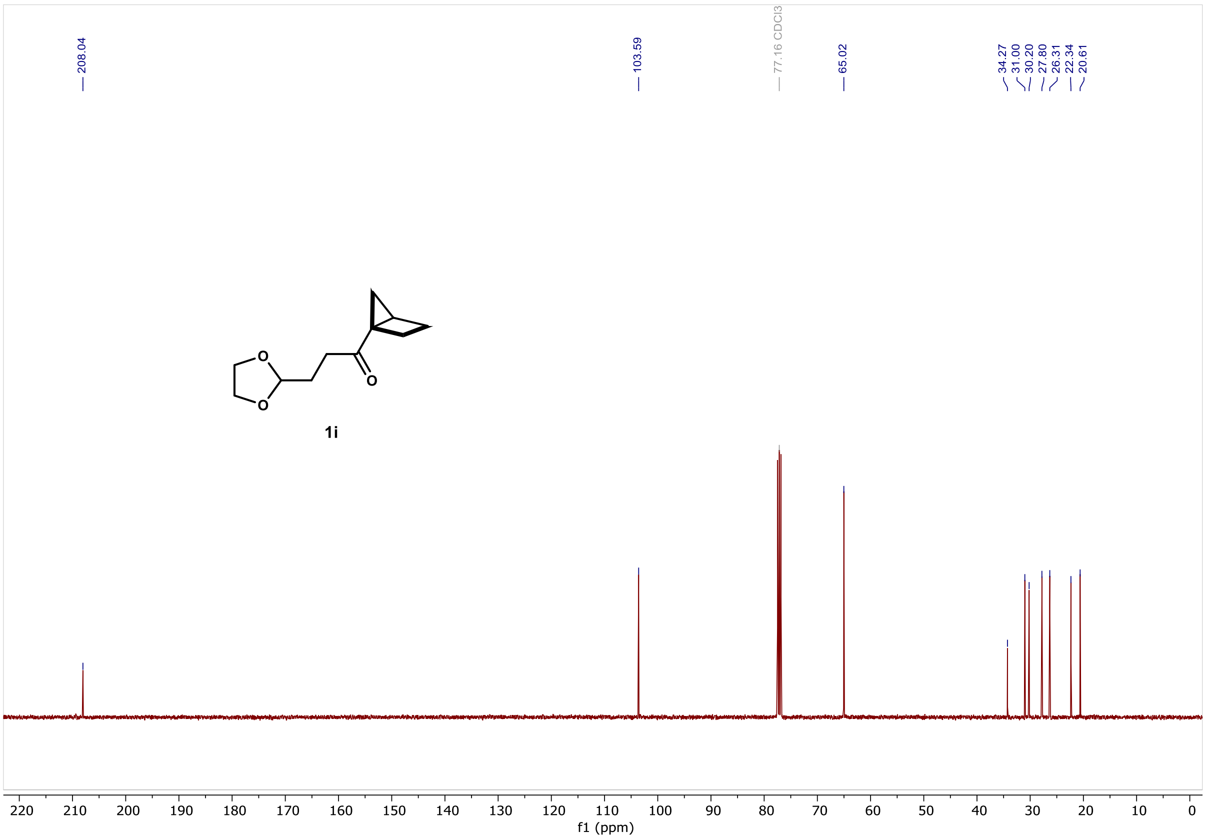

^{1}H NMR (400 MHz, Chloroform-*d*) of **SI-29 – mixture of diastereoisomers**:

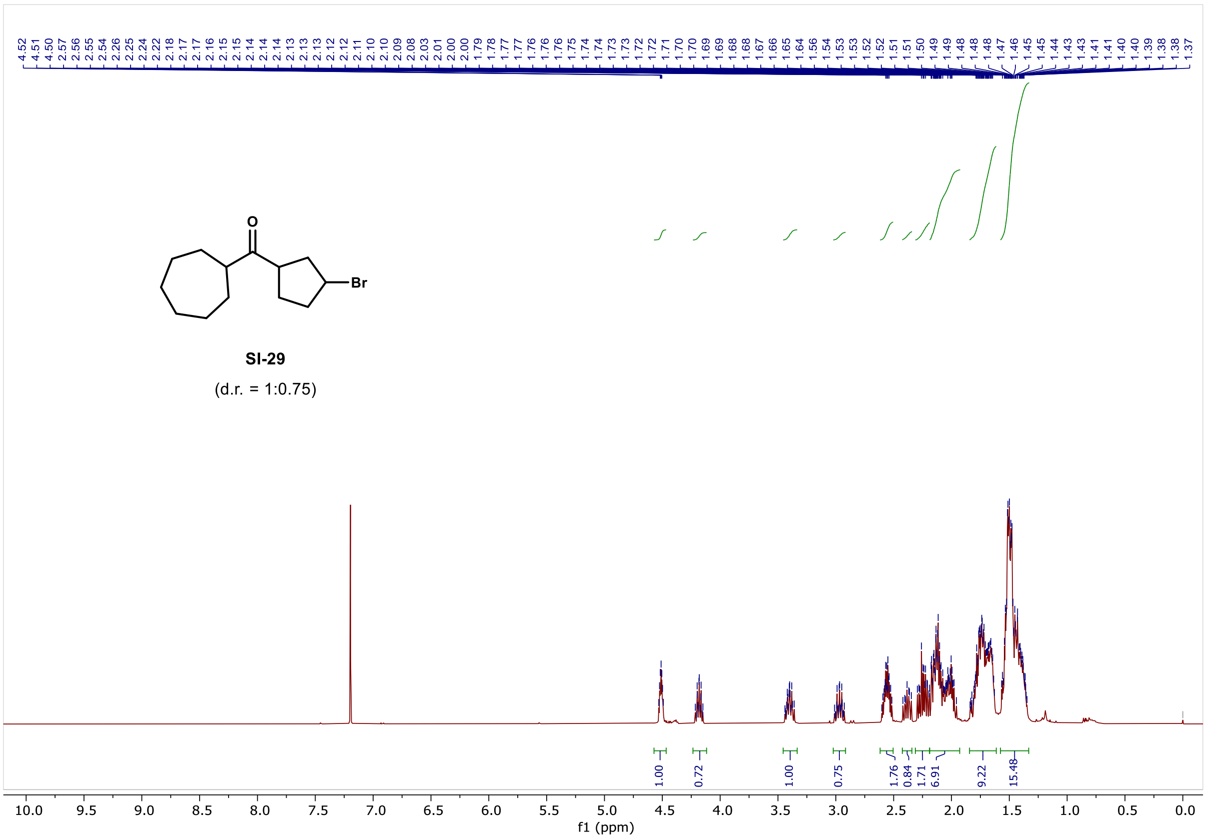

^{13}C NMR (101 MHz, Chloroform-*d*) of **SI-29 – mixture of diastereoisomers**:

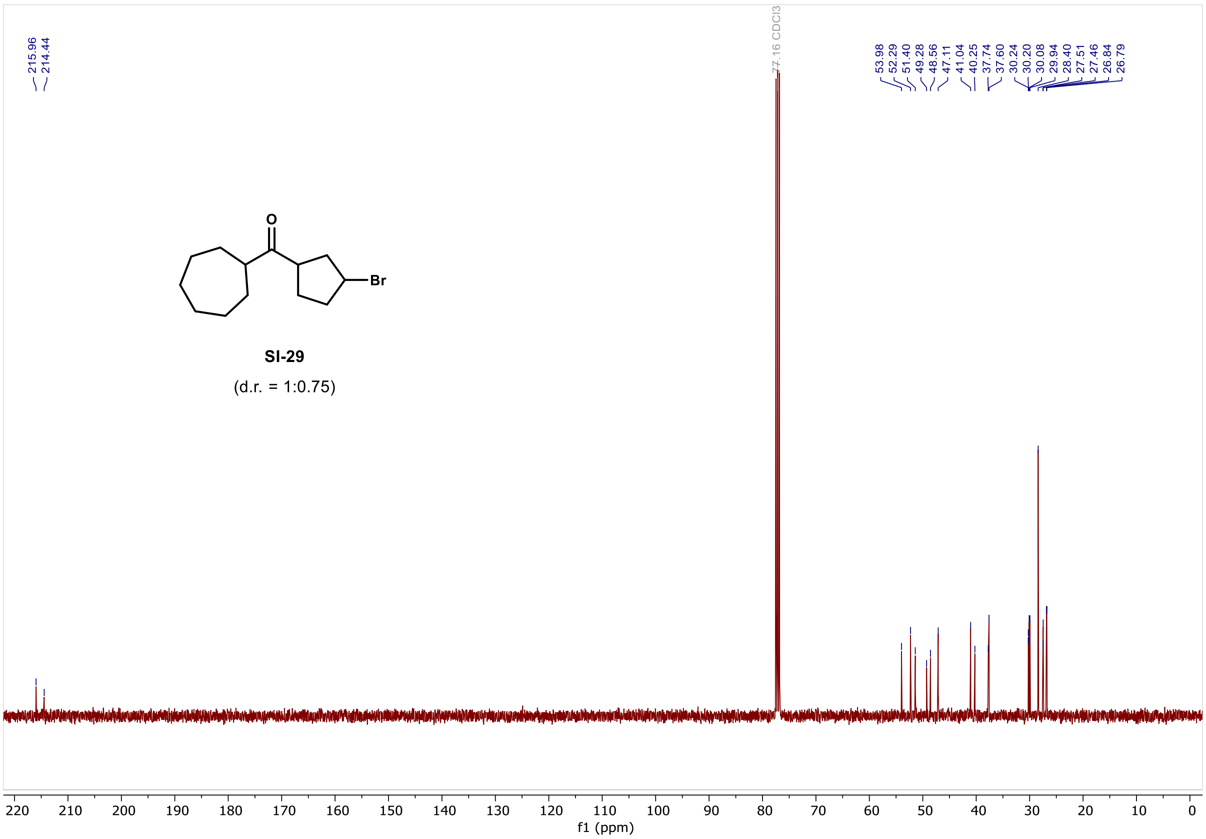

^{1}H NMR (400 MHz, Chloroform-*d*) of **1k**:

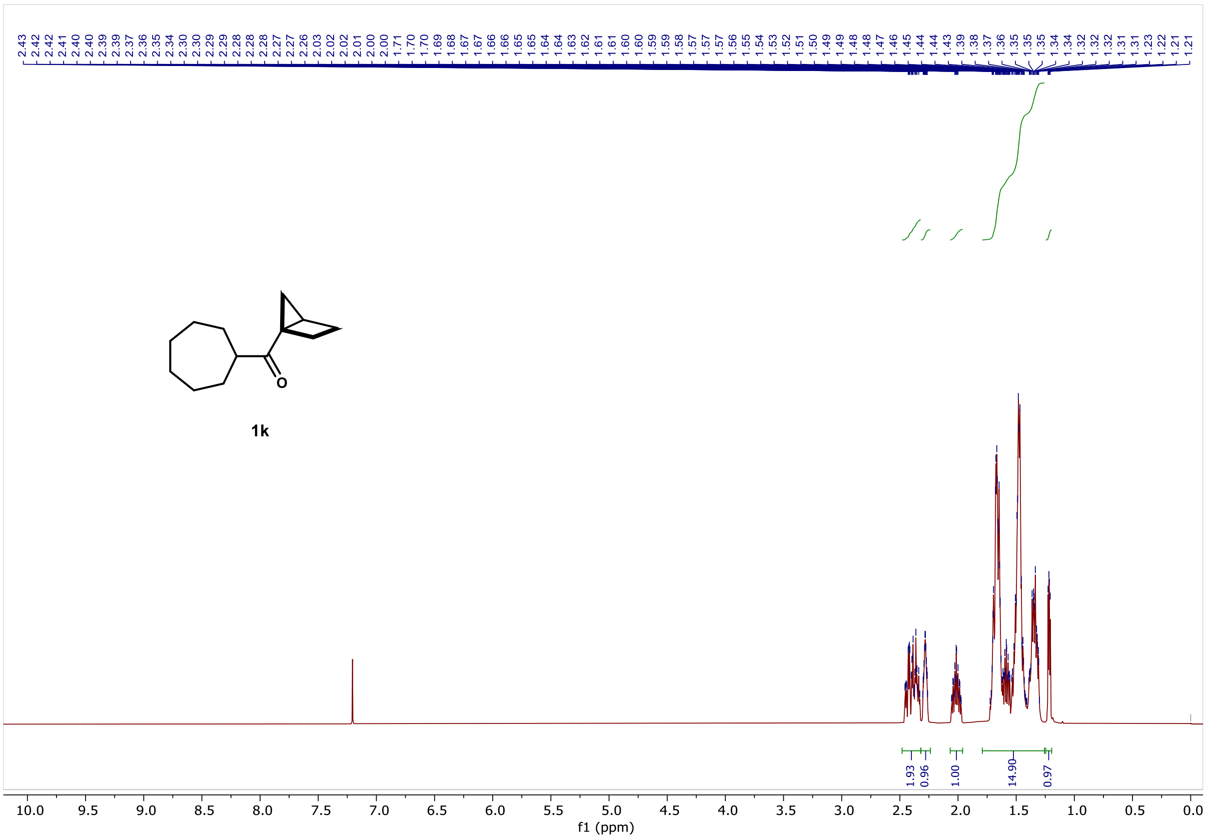

^{13}C NMR (101 MHz, Chloroform-*d*) of **1k**:

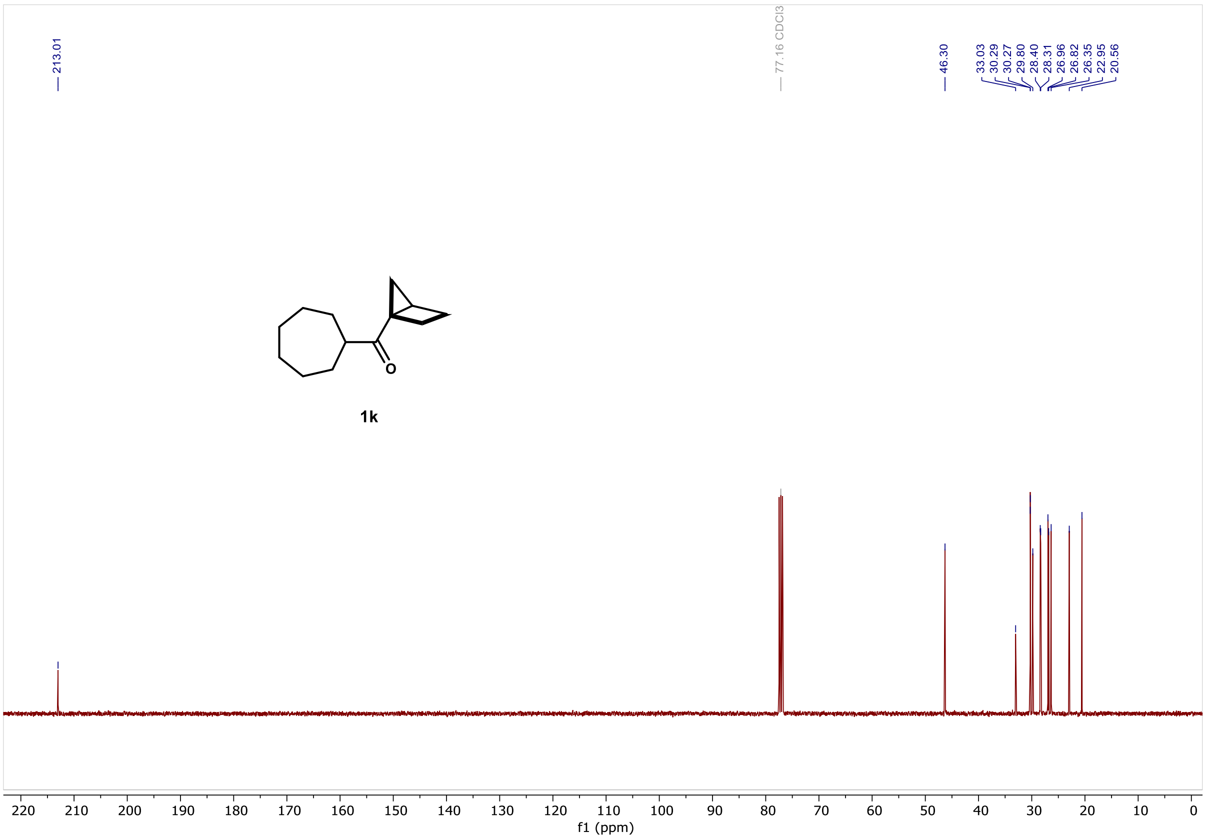

^{1}H NMR (400 MHz, Chloroform-*d*) of **SI-30 – mixture of diastereoisomers**:

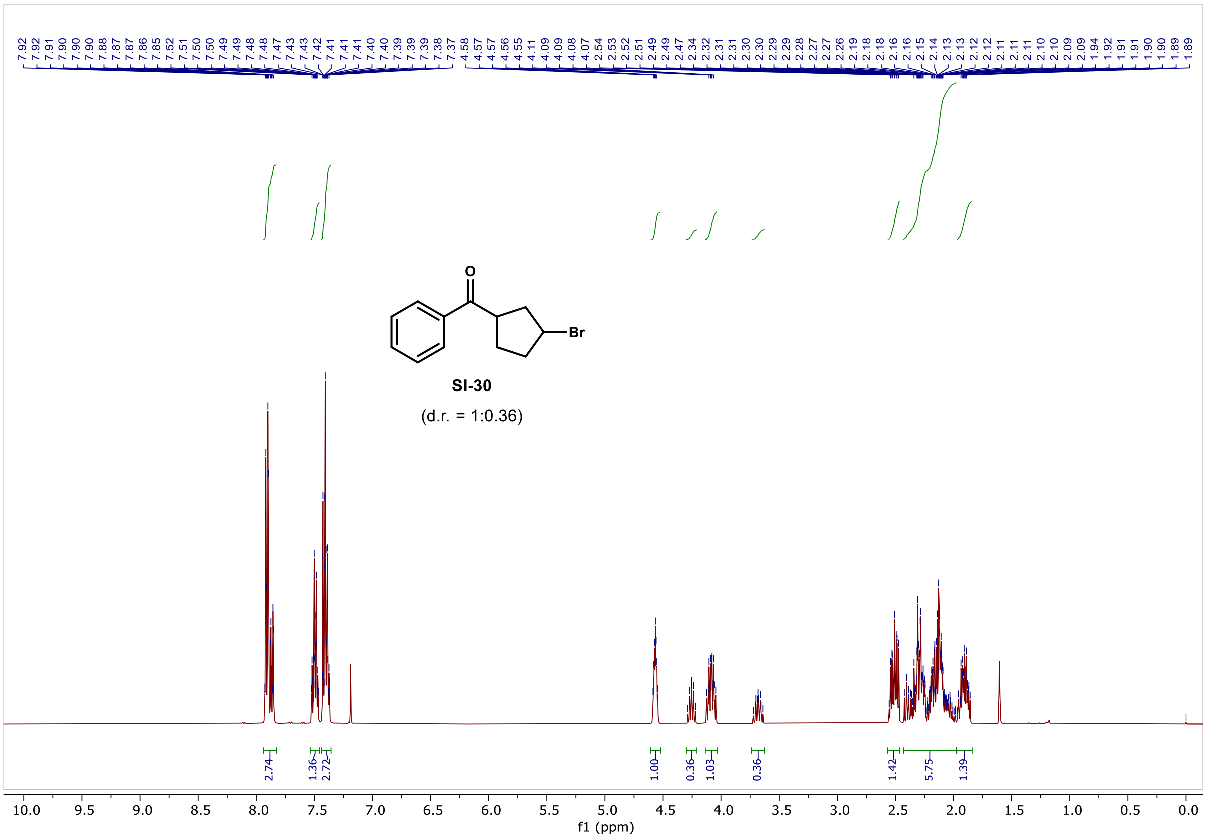

^{13}C NMR (101 MHz, Chloroform-*d*) of **SI-30 – mixture of diastereoisomers**:

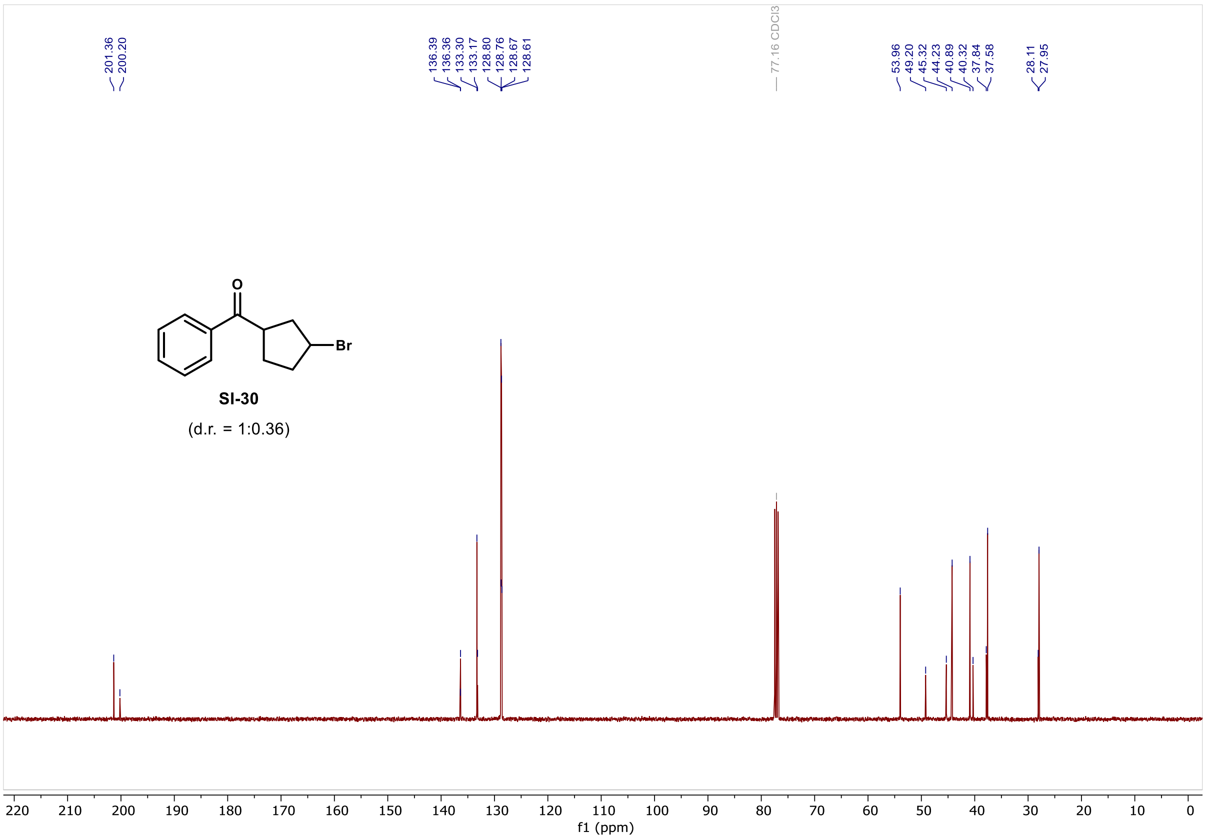

^{1}H NMR (400 MHz, Chloroform-*d*) of **1n**:

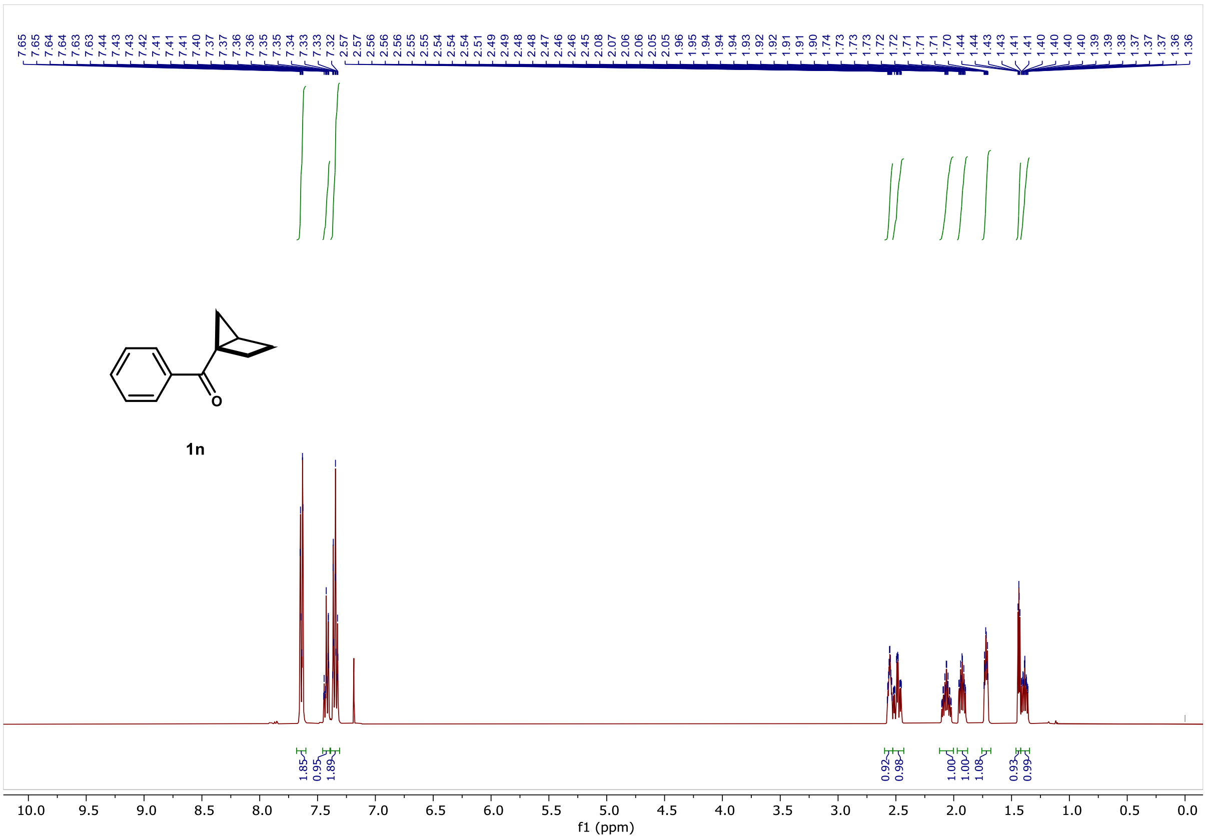

^{13}C NMR (101 MHz, Chloroform-*d*) of **1n**:

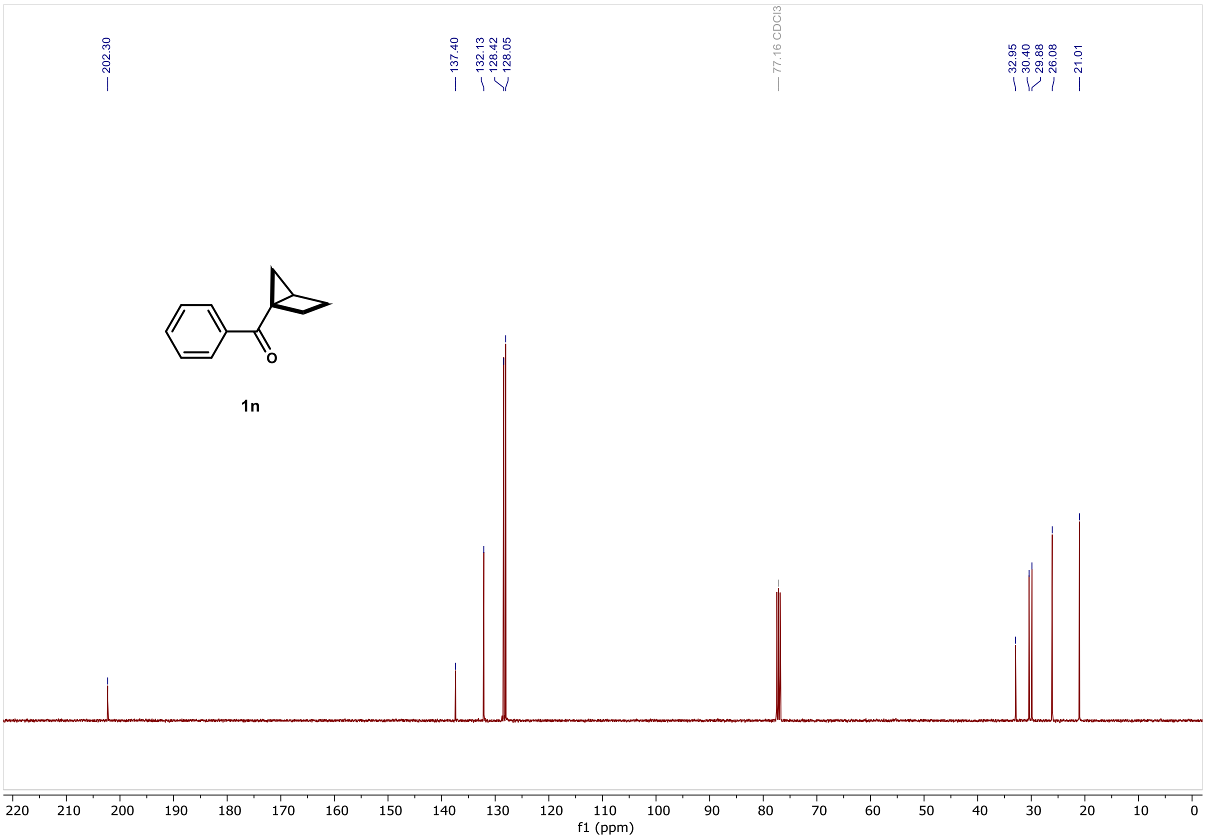

^{1}H NMR (400 MHz, Chloroform-*d*) of **SI-34 – mixture of diastereoisomers**:

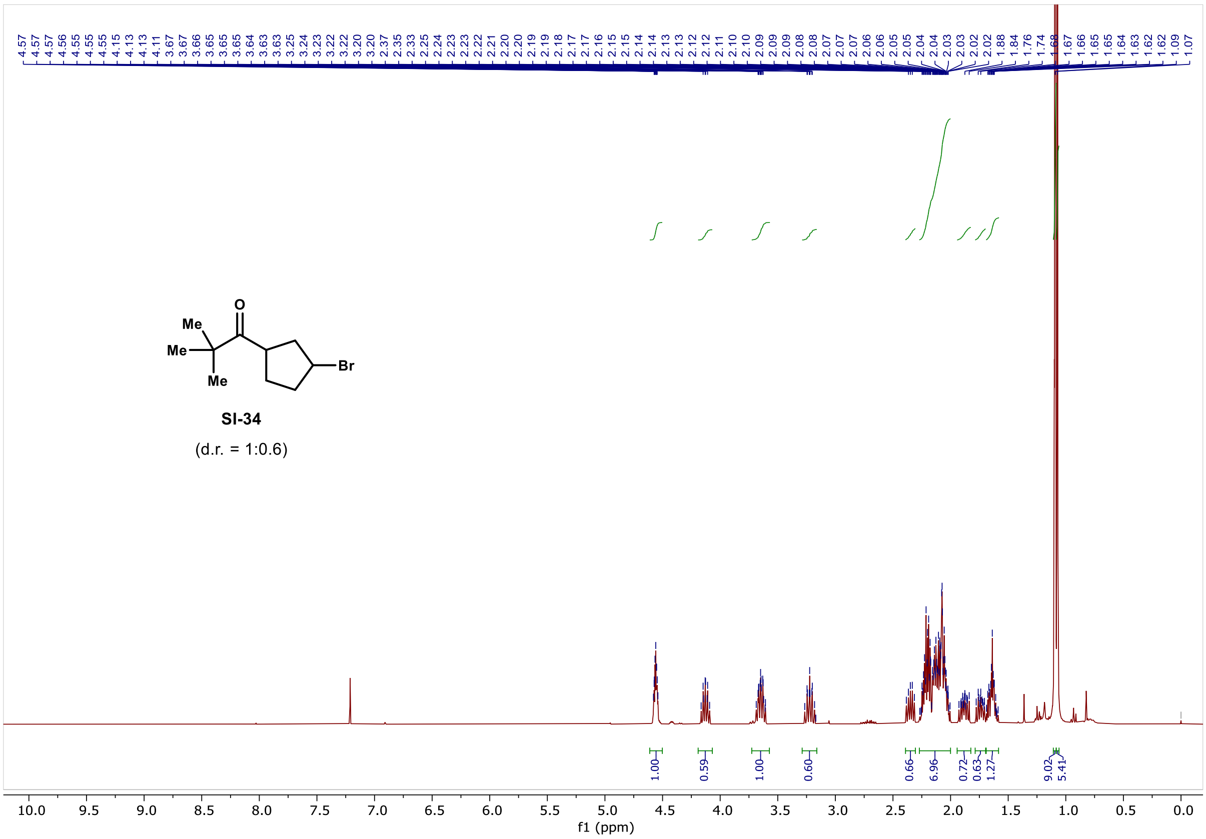

^{13}C NMR (101 MHz, Chloroform-*d*) of **SI-34 – mixture of diastereoisomers**:

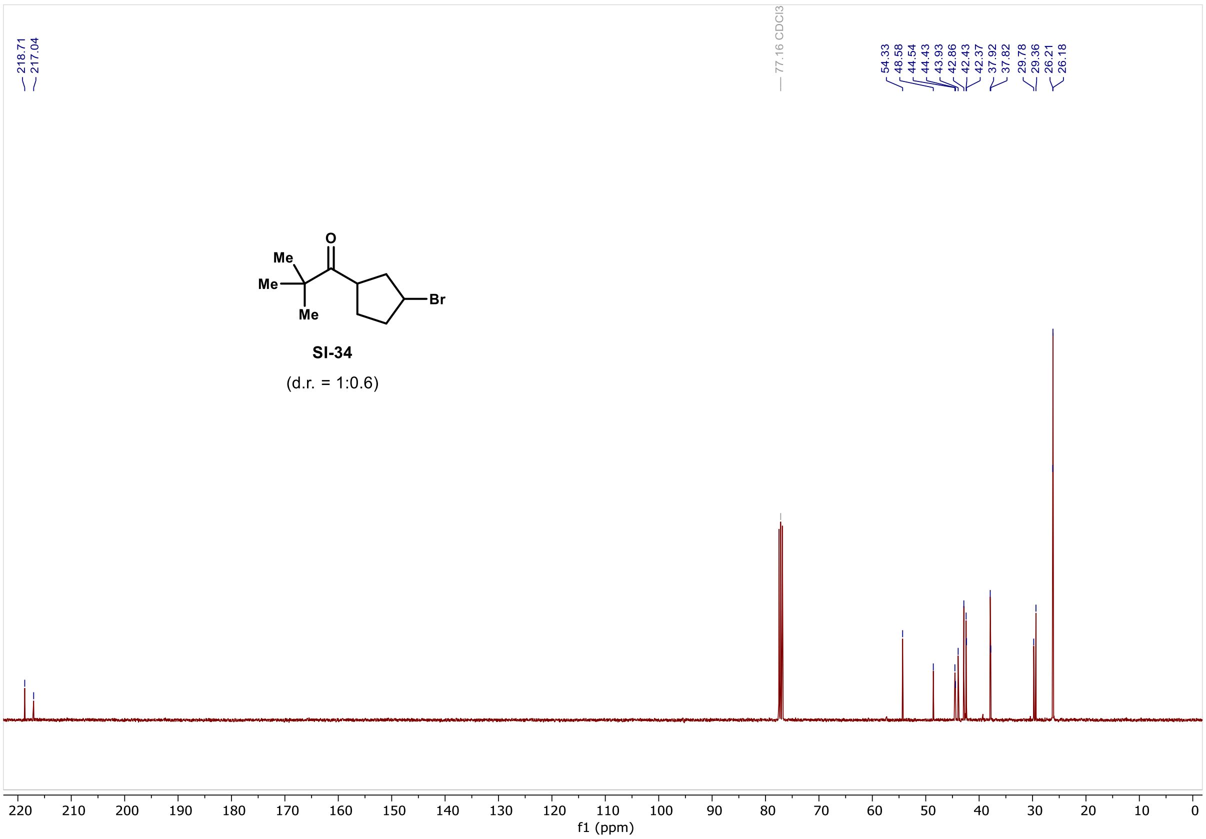

^{1}H NMR (400 MHz, Chloroform-*d*) of **1m**:

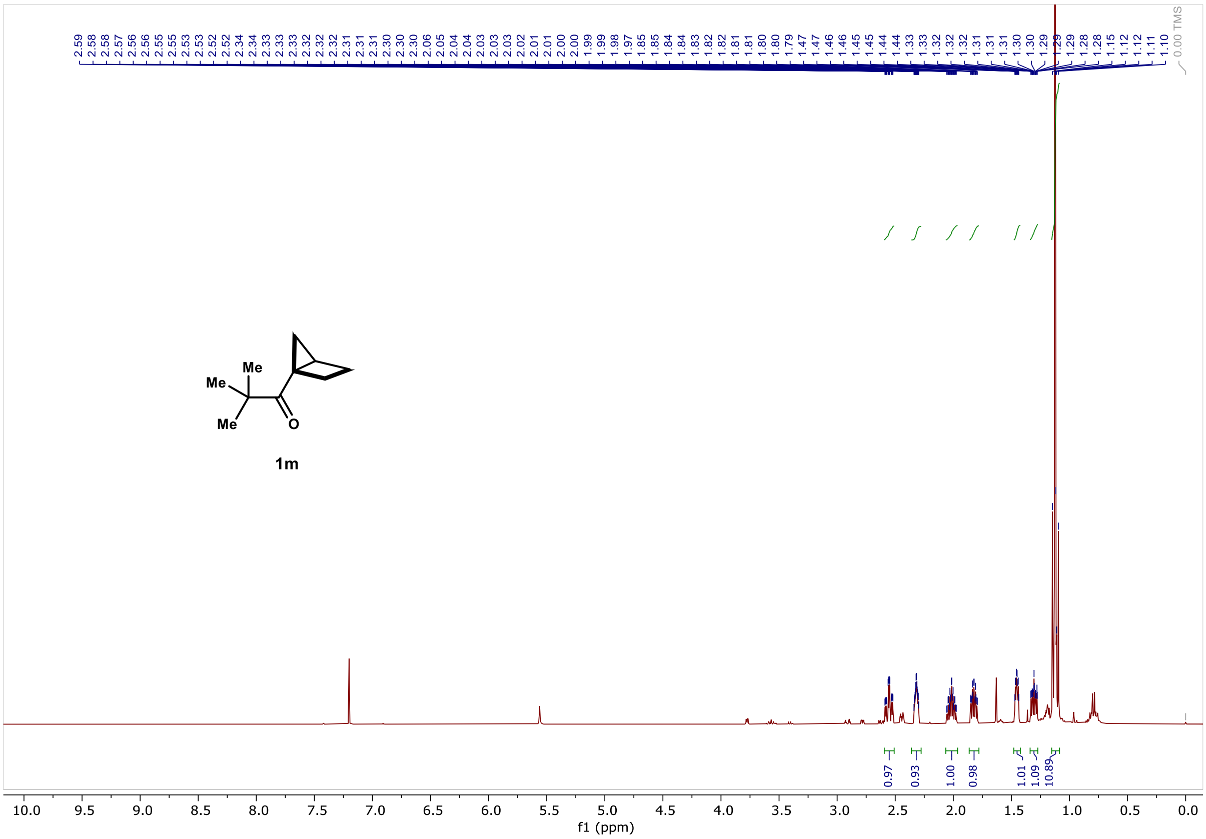

^{13}C NMR (101 MHz, Chloroform-*d*) of **1m**:

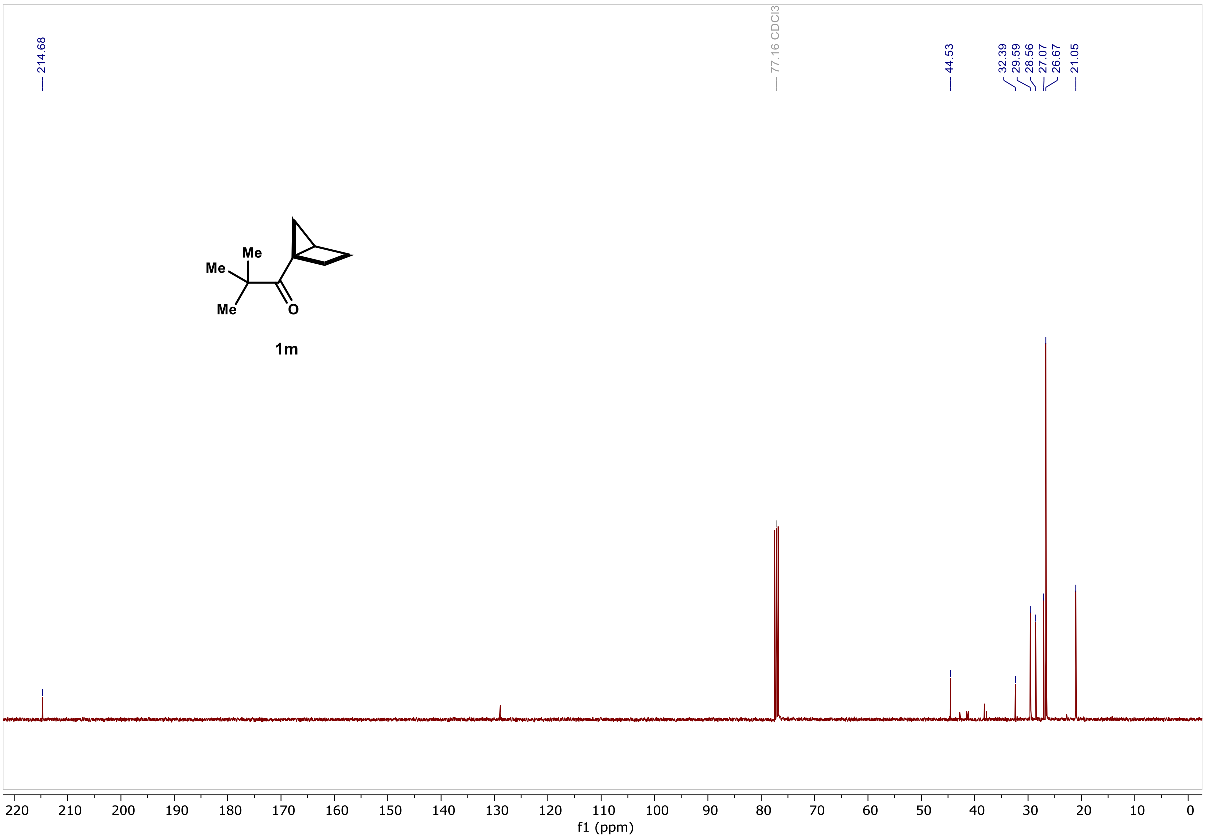

^{1}H NMR (400 MHz, Benzene-*d6*) of **SI-38**:

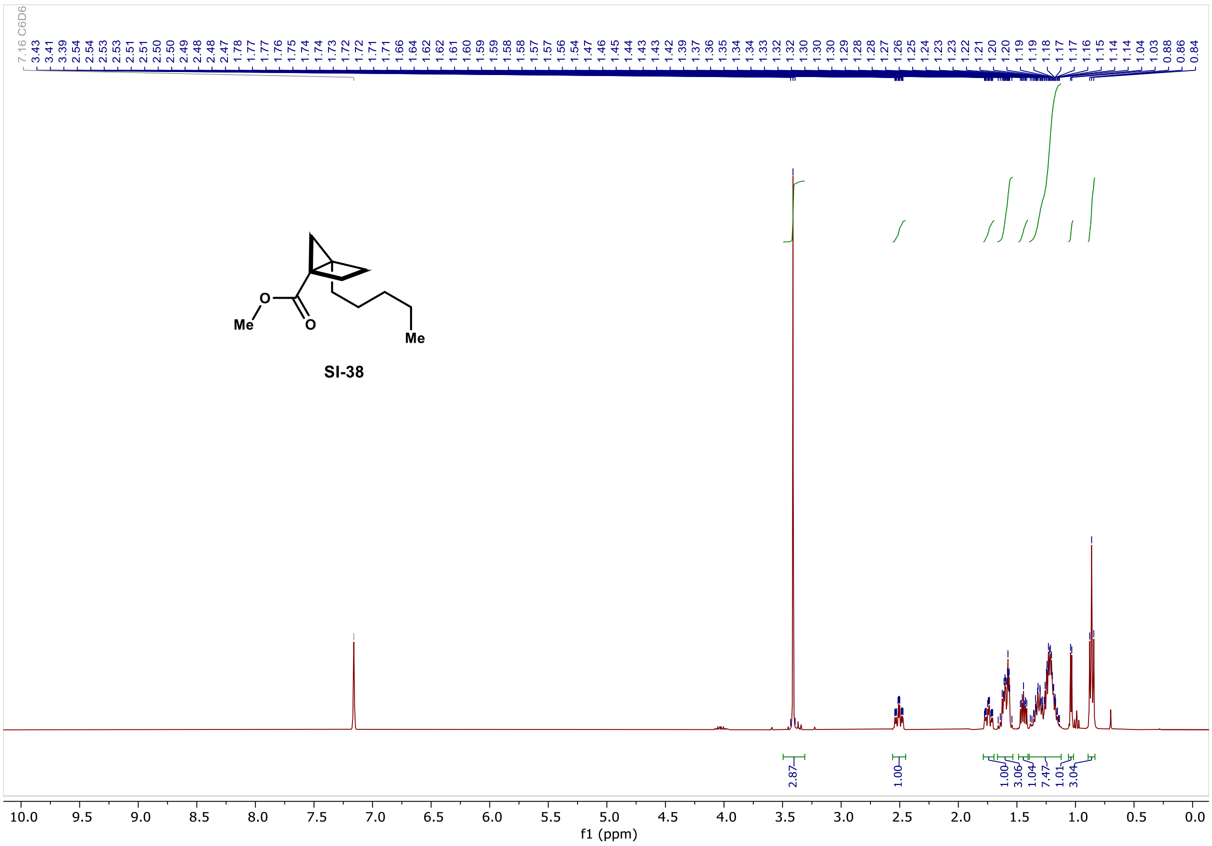

^{13}C NMR (101 MHz, Benzene-*d6*) of **SI-38**:

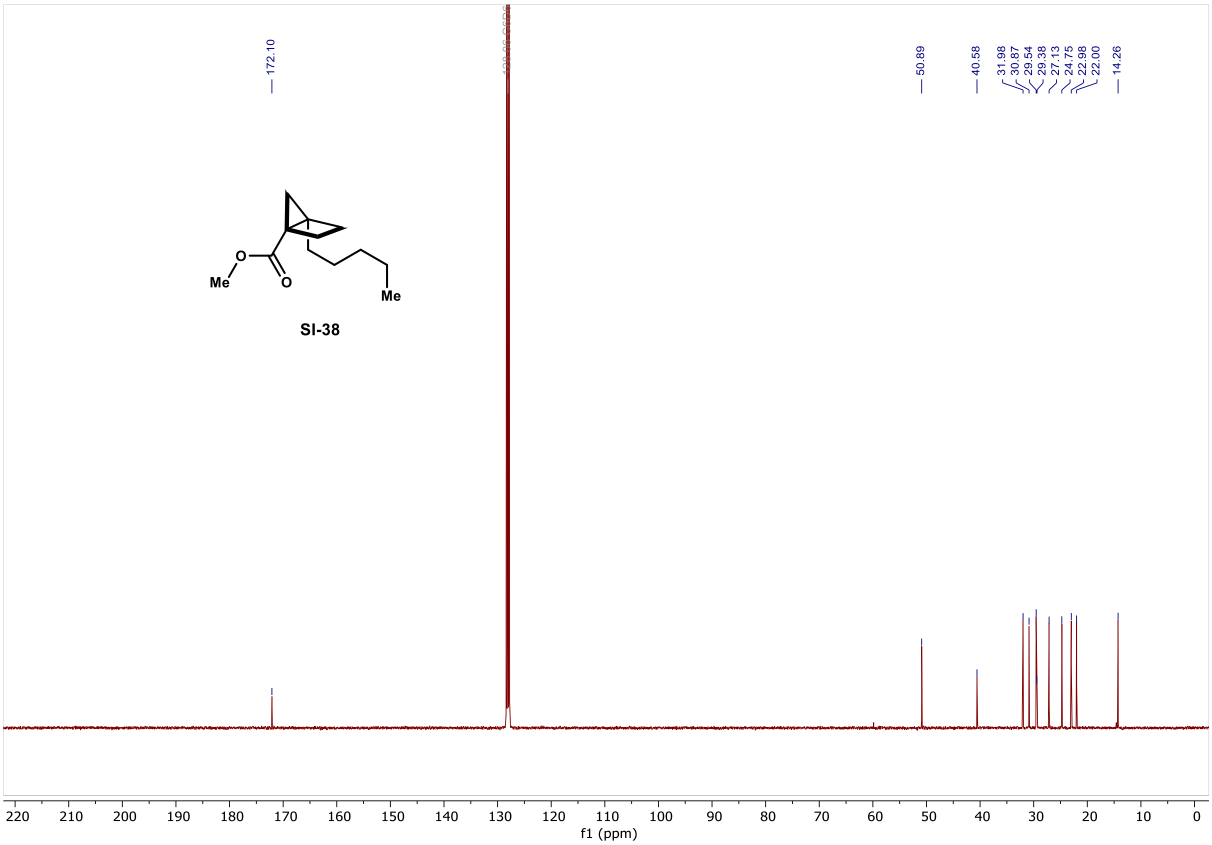

^{1}H NMR (400 MHz, Benzene-*d6*) of **1s**:

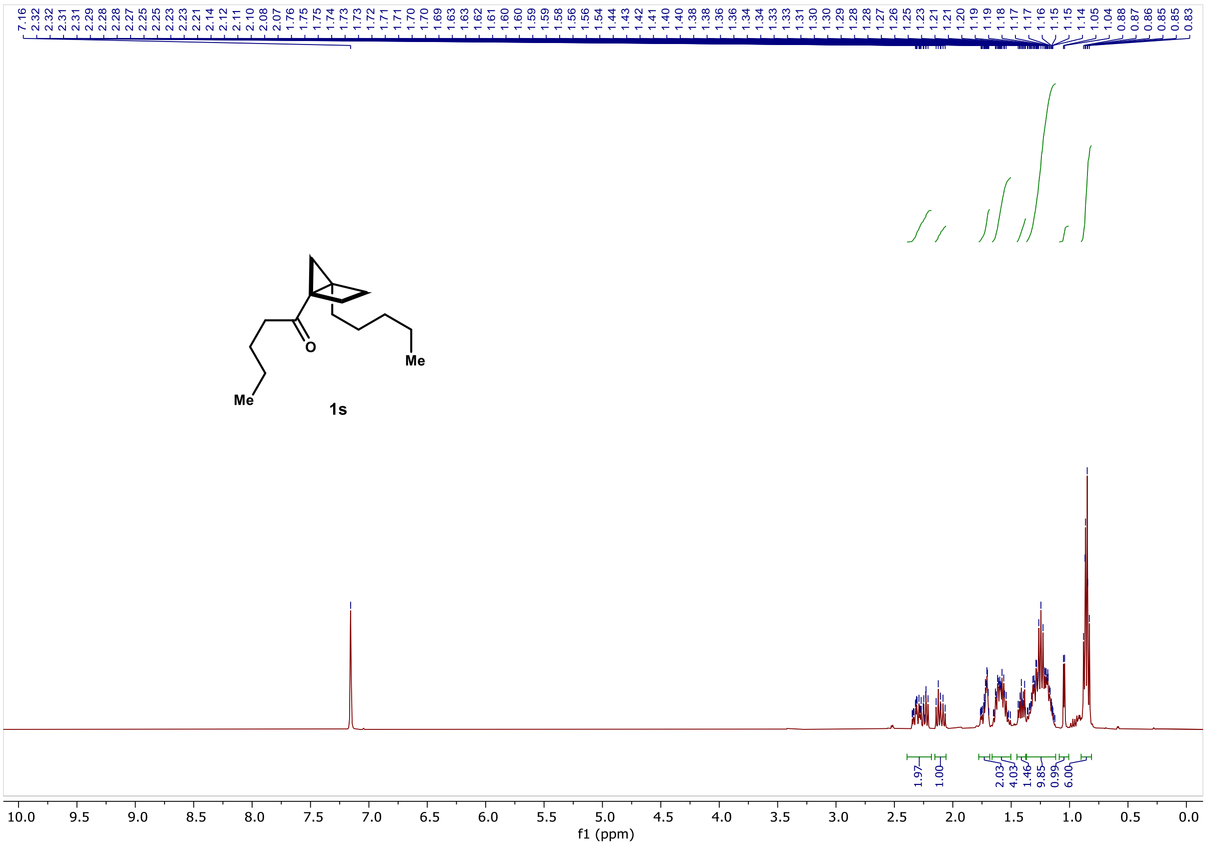

^{13}C NMR (101 MHz, Benzene-*d6*) of **1s**:

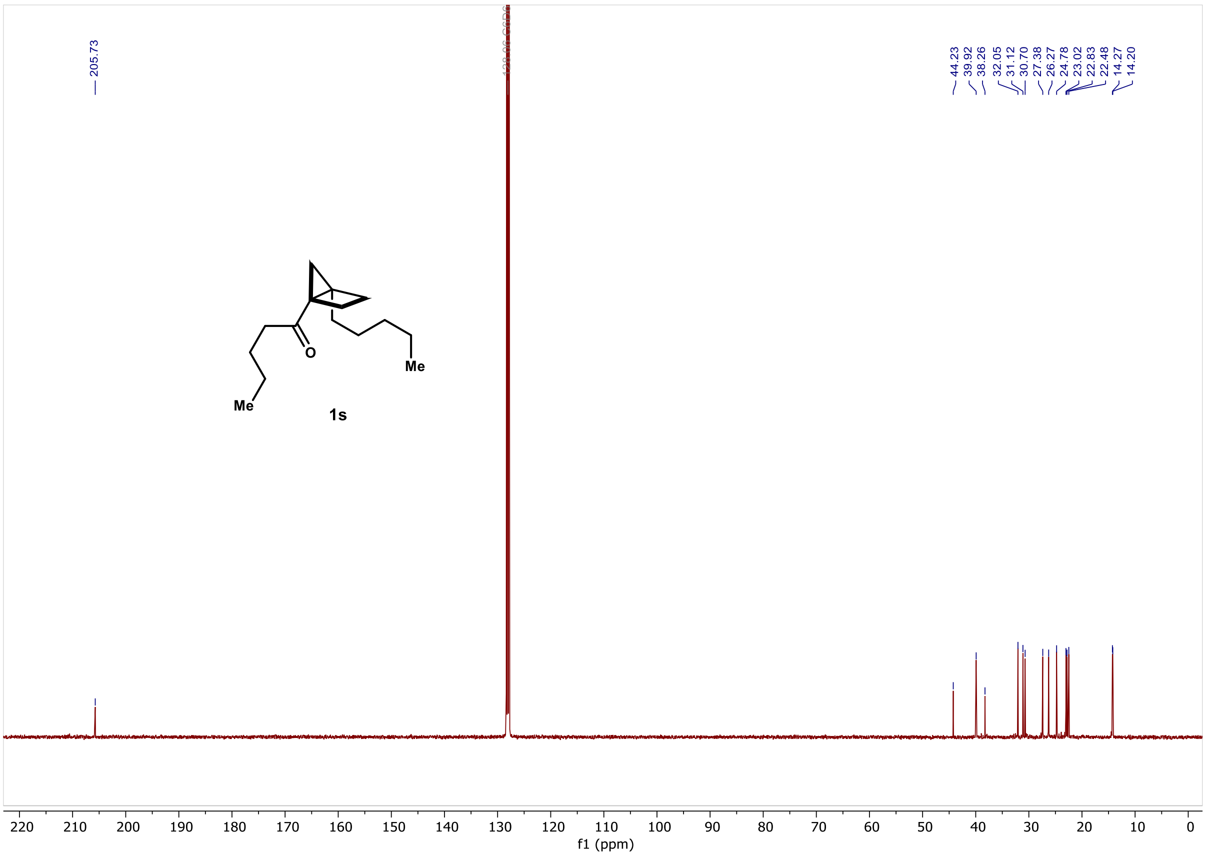

^{1}H NMR (500 MHz, Chloroform-*d*) of **SI-41**:

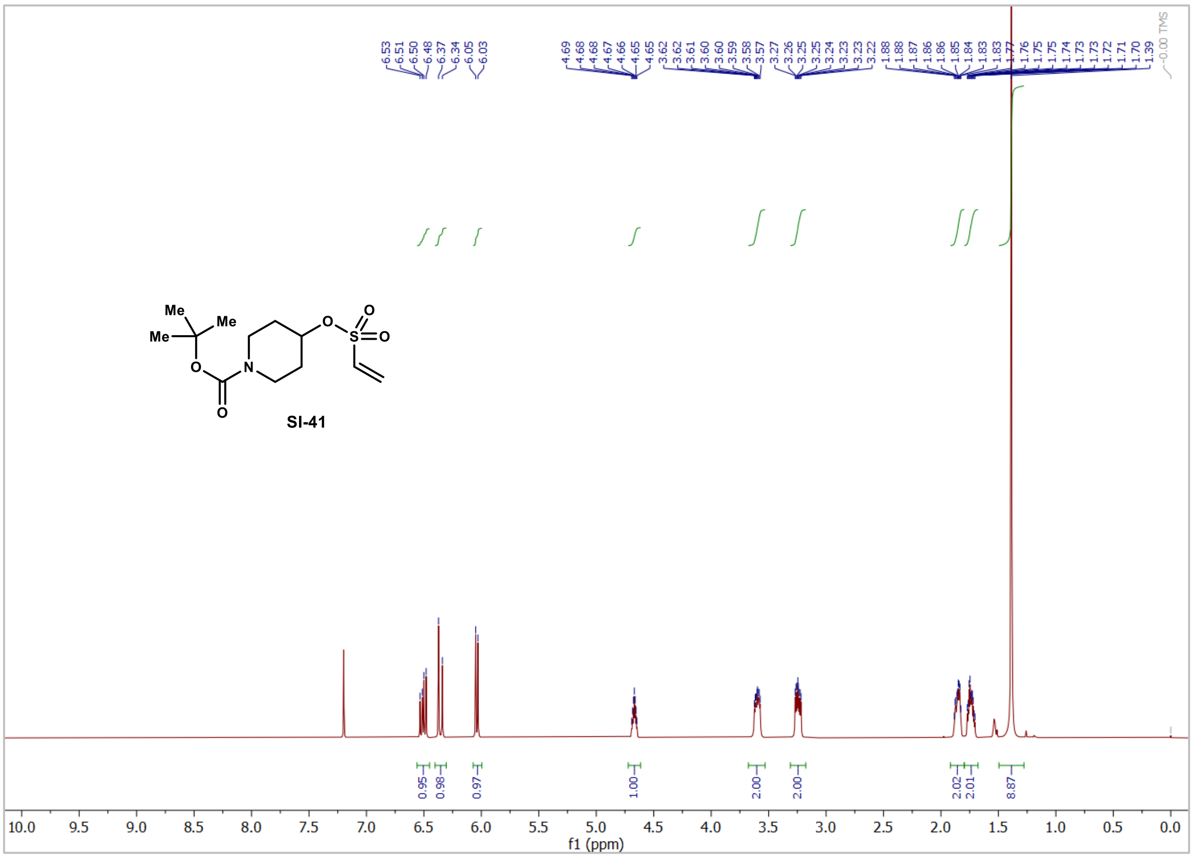

^{13}C NMR (126 MHz, Chloroform-*d*) of **SI-41**:

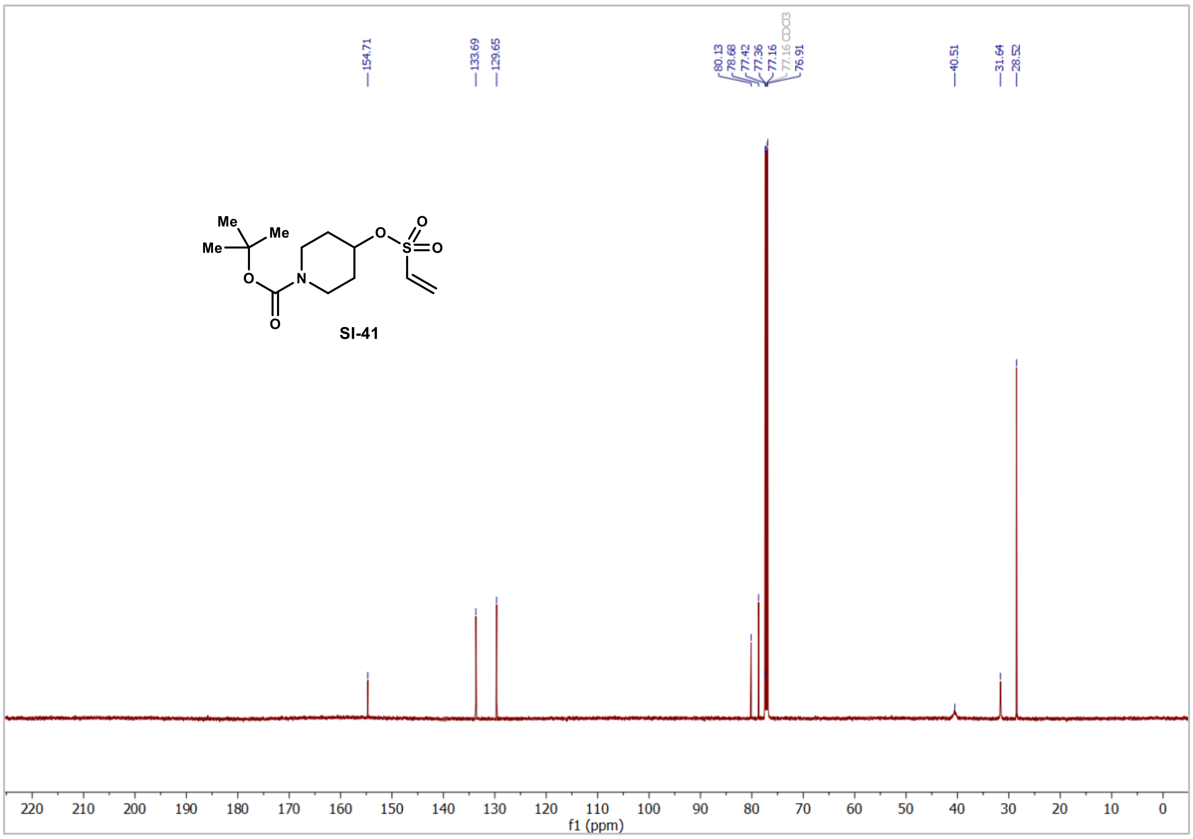

^{1}H NMR (400 MHz, Chloroform-*d*) of **SI-42**:

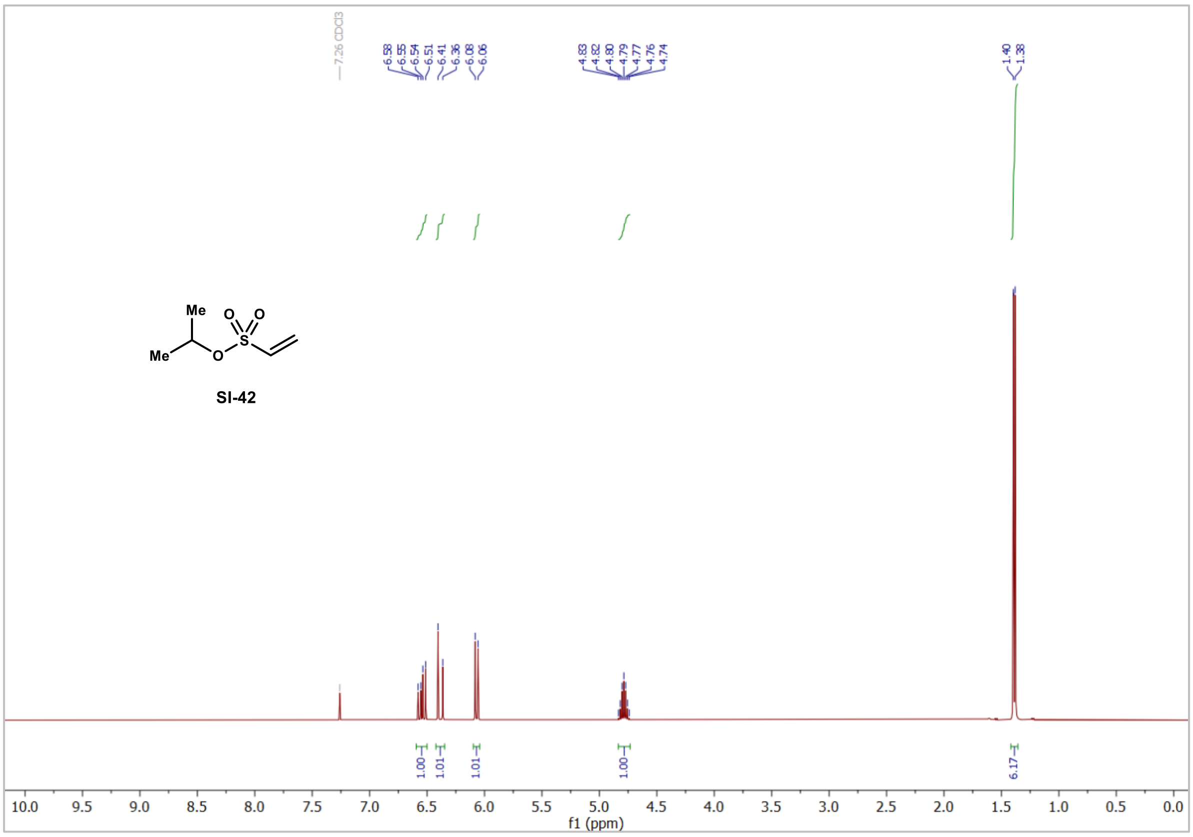

^{13}C NMR (101 MHz, Chloroform-*d*) of **SI-42**:

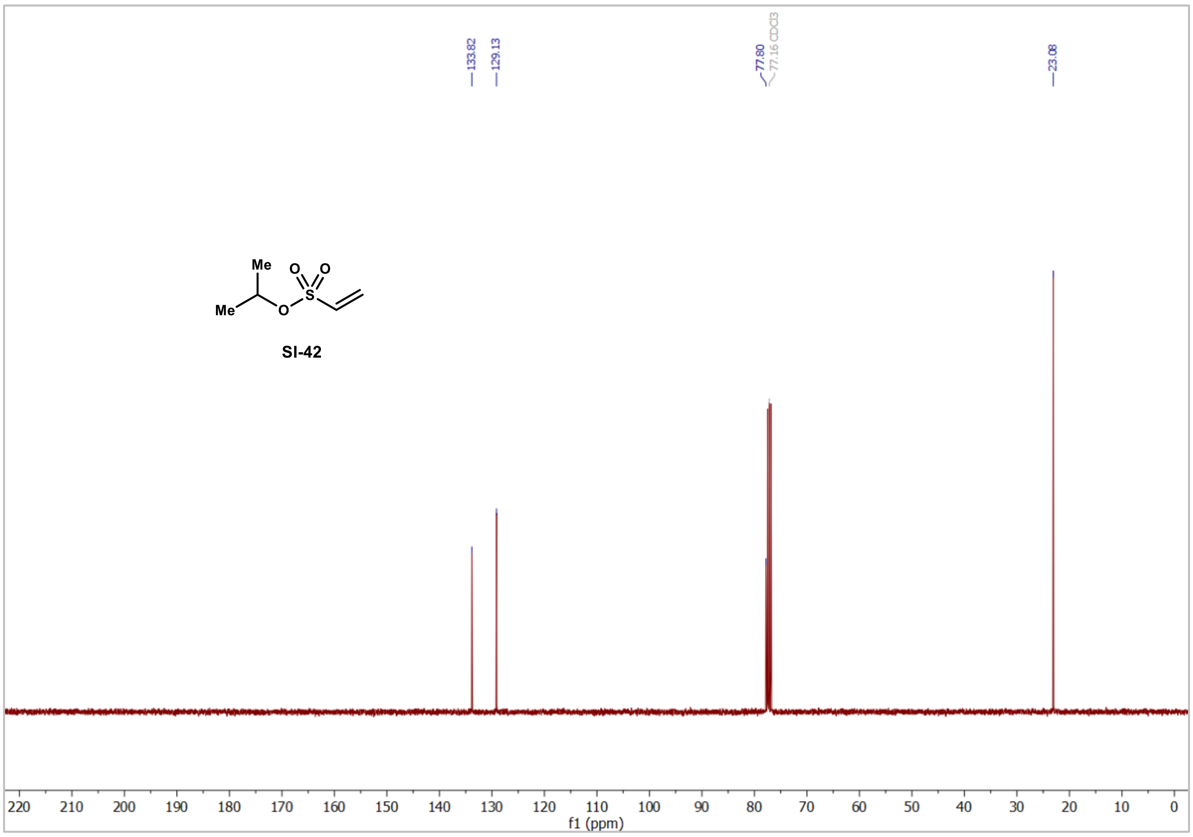

^{1}H NMR (400 MHz, Chloroform-*d*) of **2a-*endo***:

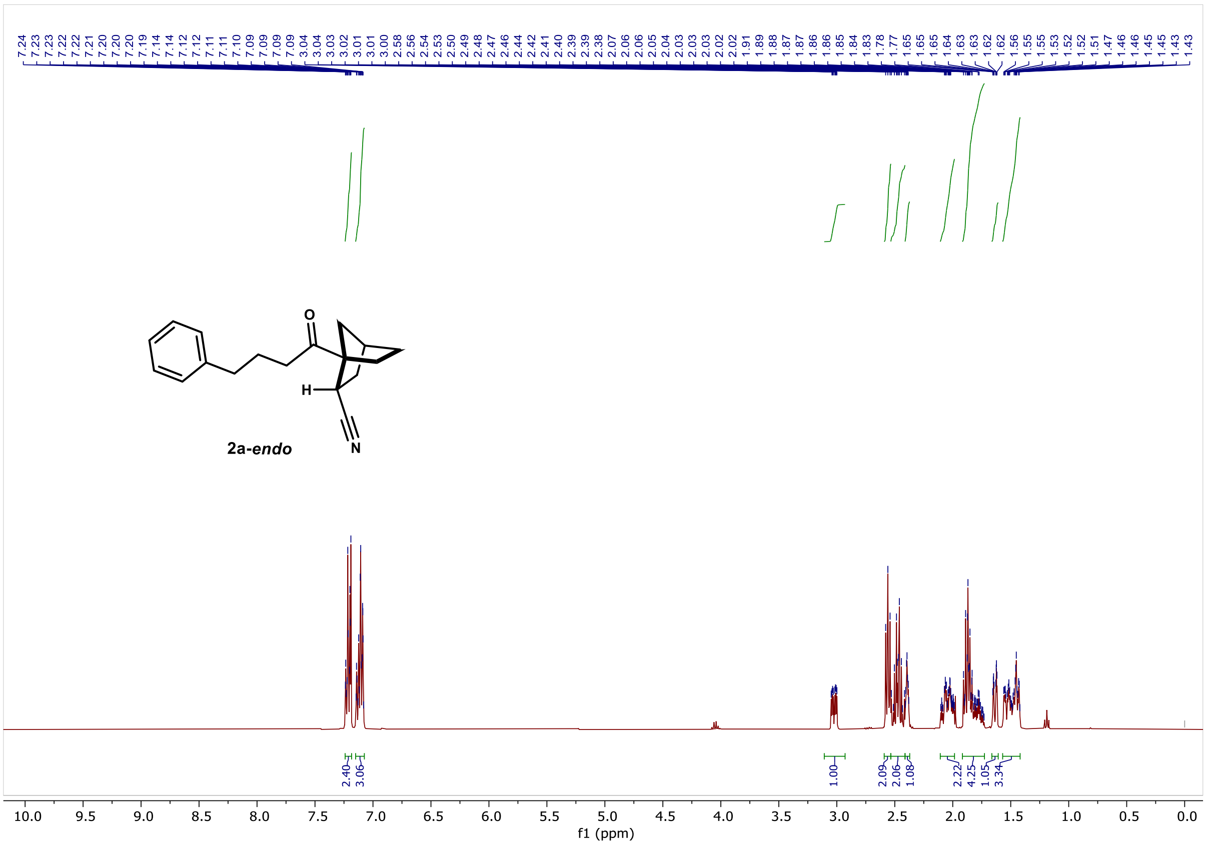

^{13}C NMR (101 MHz, Chloroform-*d*) of **2a-*endo***:

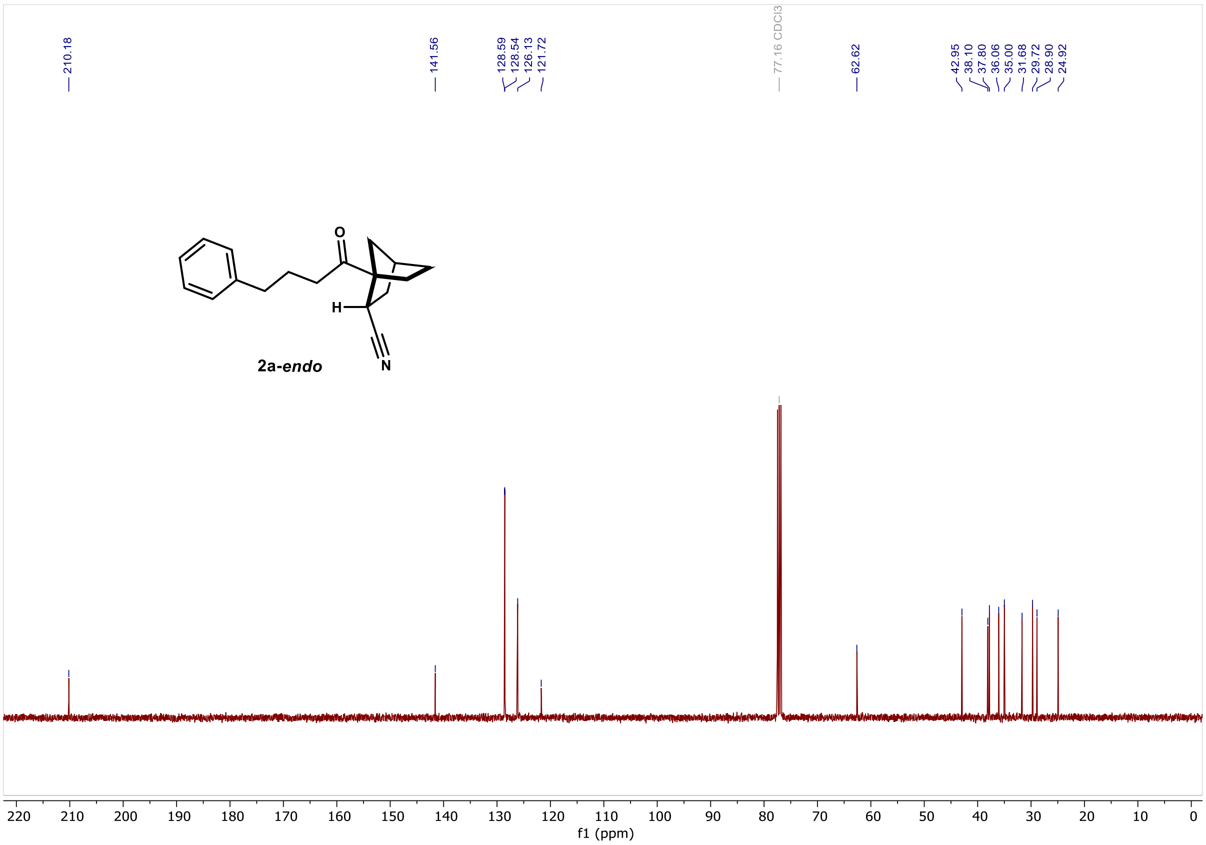

^{1}H NMR (400 MHz, Chloroform-*d*) of **2a-*exo***:

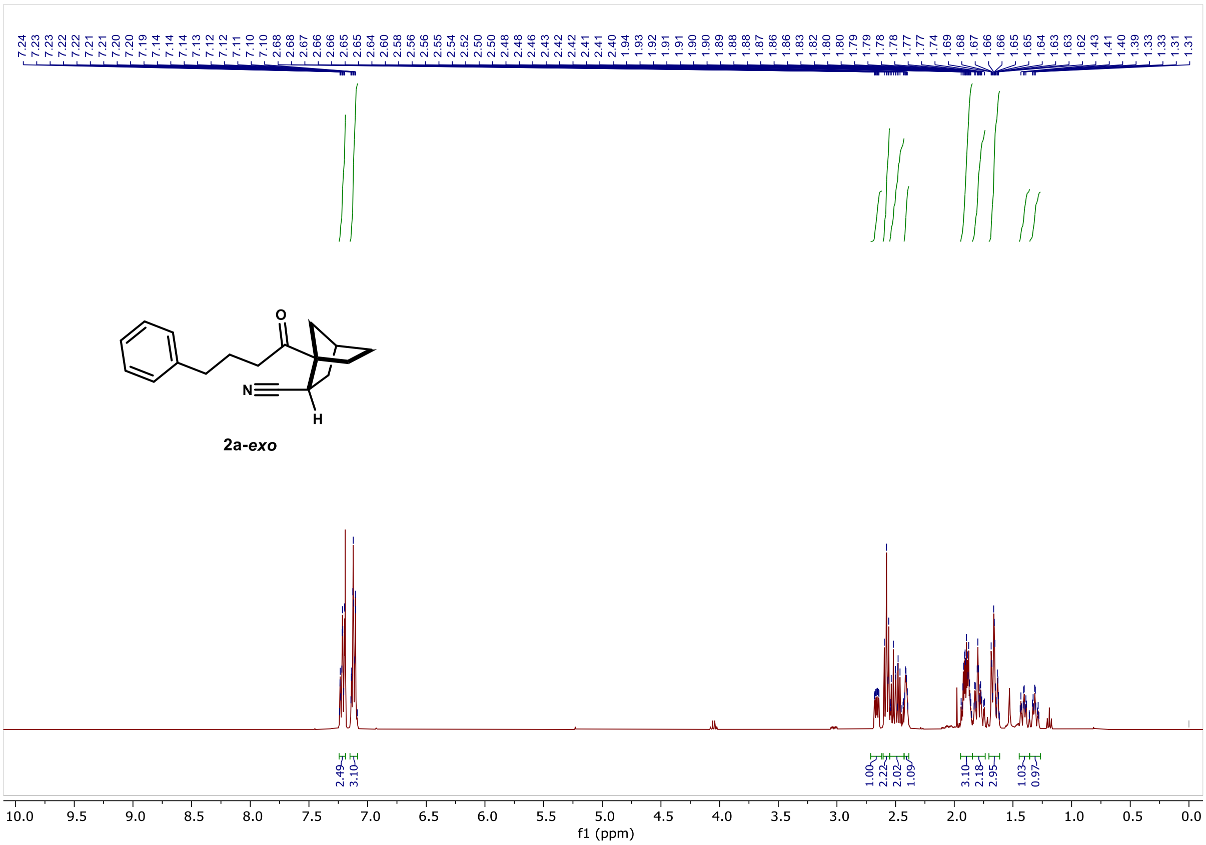

^{13}C NMR (101 MHz, Chloroform-*d*) of **2a-*exo***:

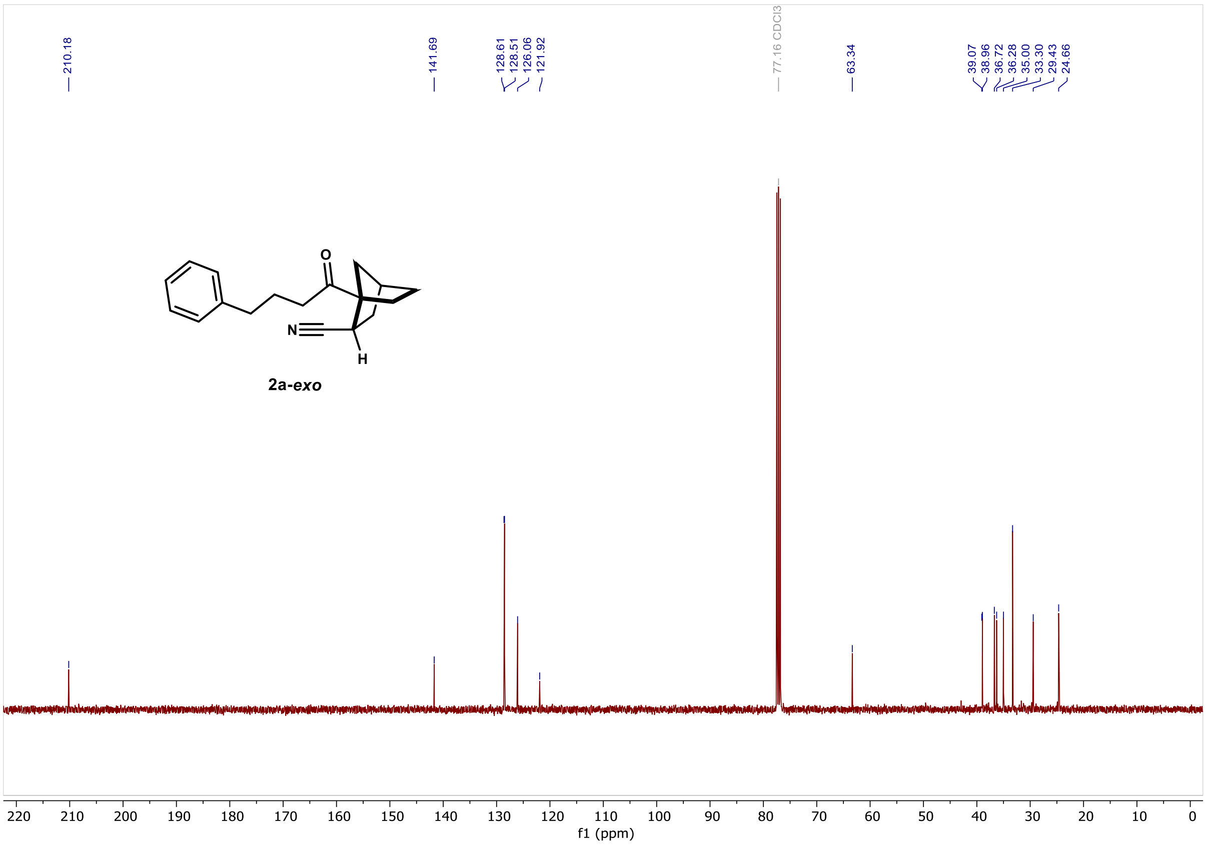

^1H NMR (400 MHz, Chloroform-*d*) of **2b-*endo***:

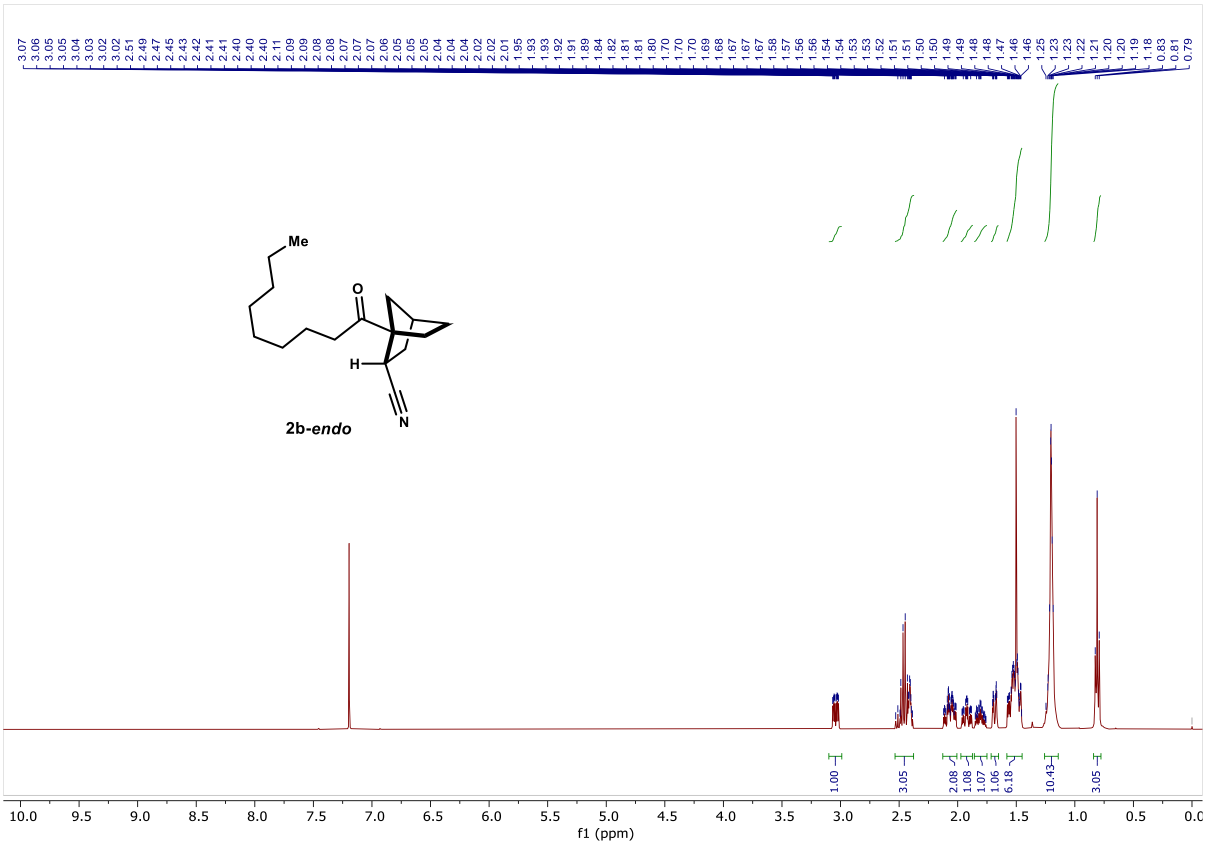

^{13}C NMR (101 MHz, Chloroform-*d*) of **2b-*endo***:

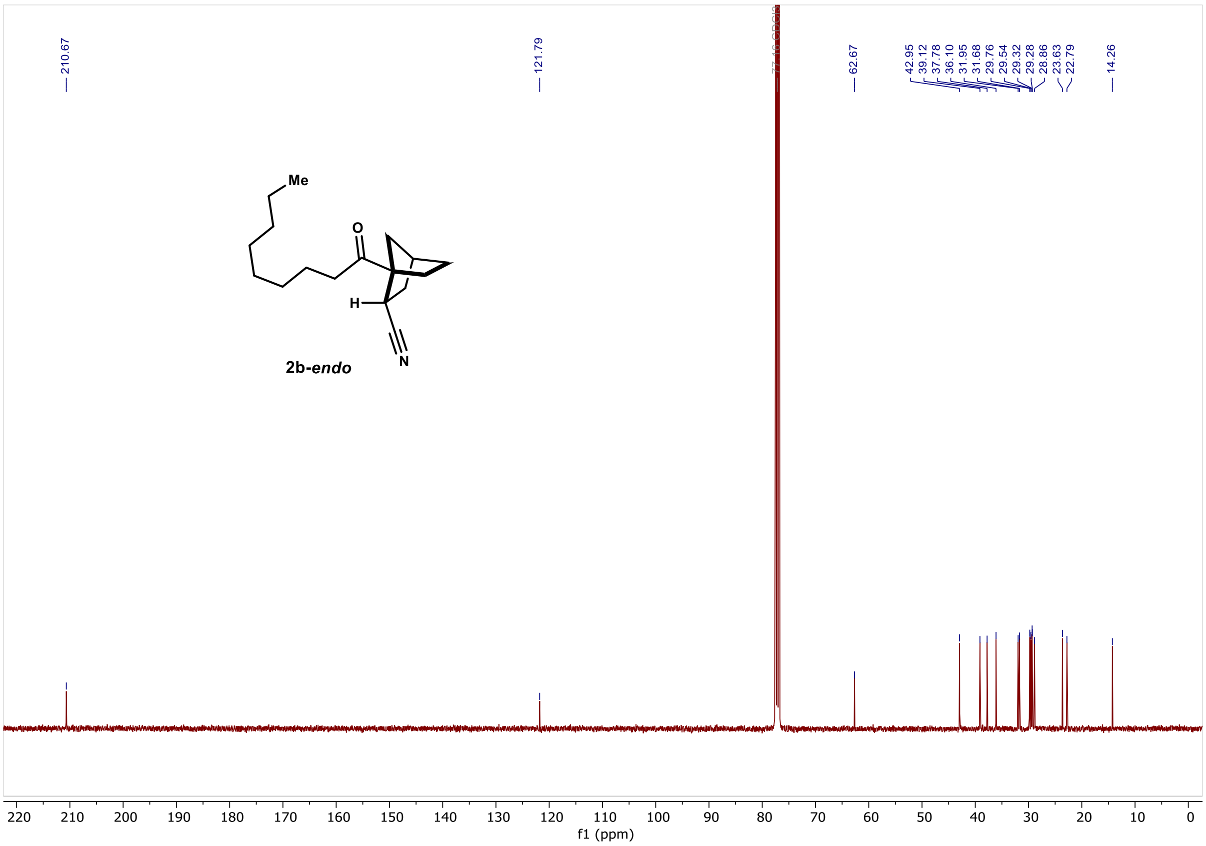

^1H NMR (400 MHz, Chloroform-*d*) of **2b-*exo***:

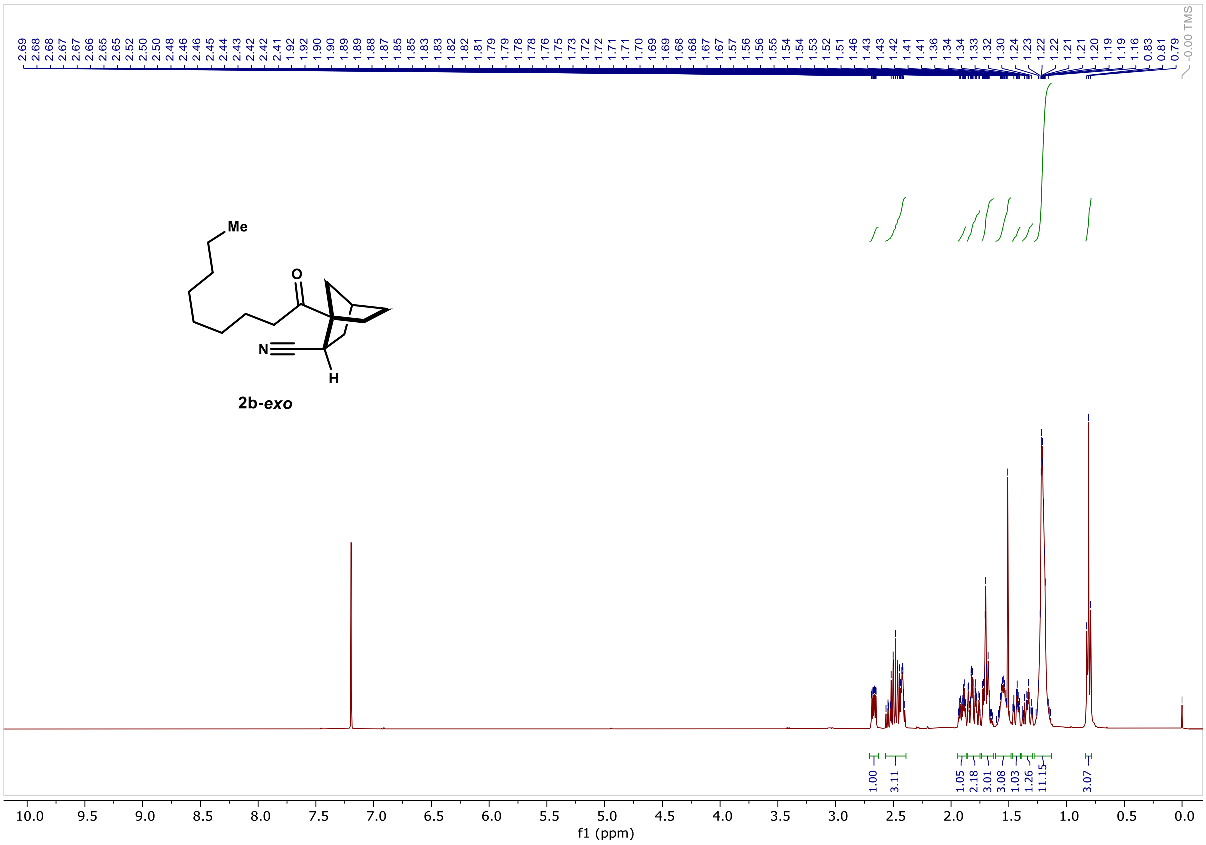

^{13}C NMR (101 MHz, Chloroform-*d*) of **2b-*exo***:

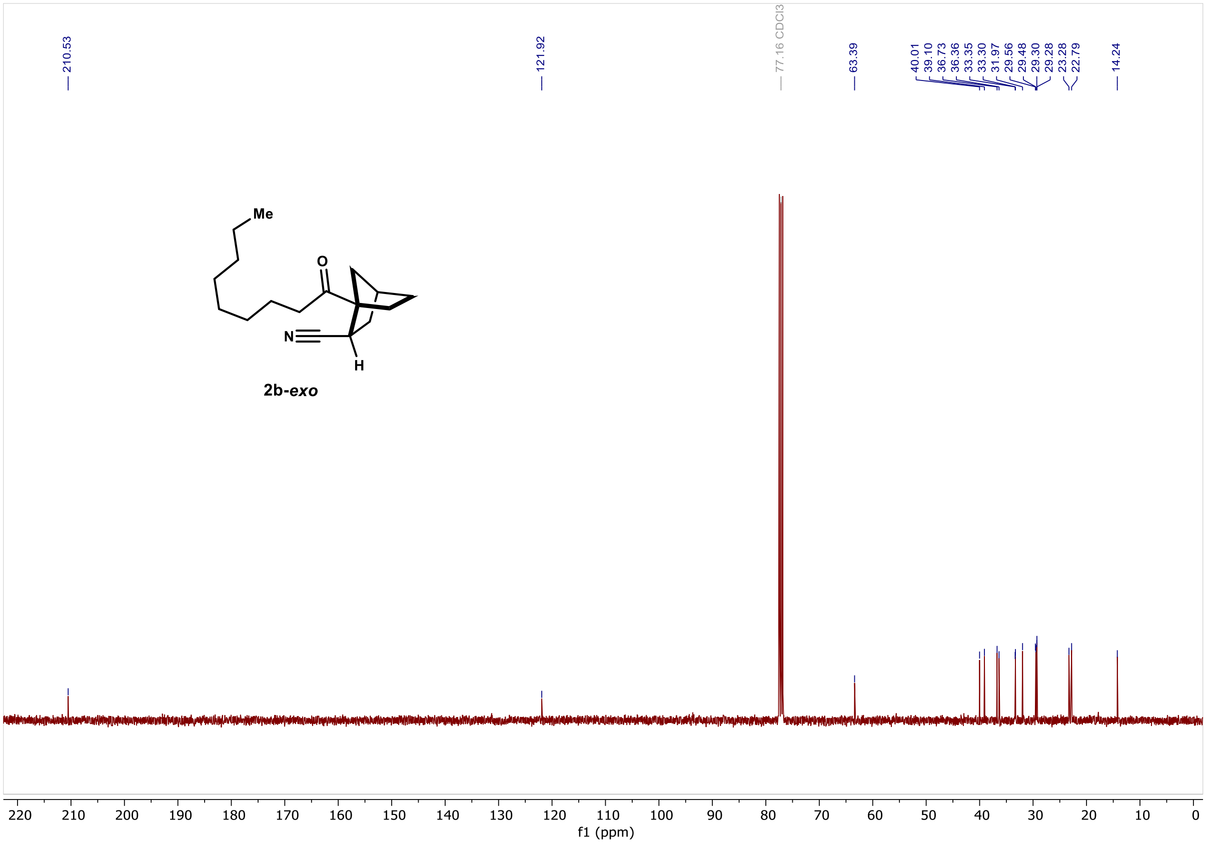

^{1}H NMR (400 MHz, Chloroform-*d*) of **2c-*endo***:

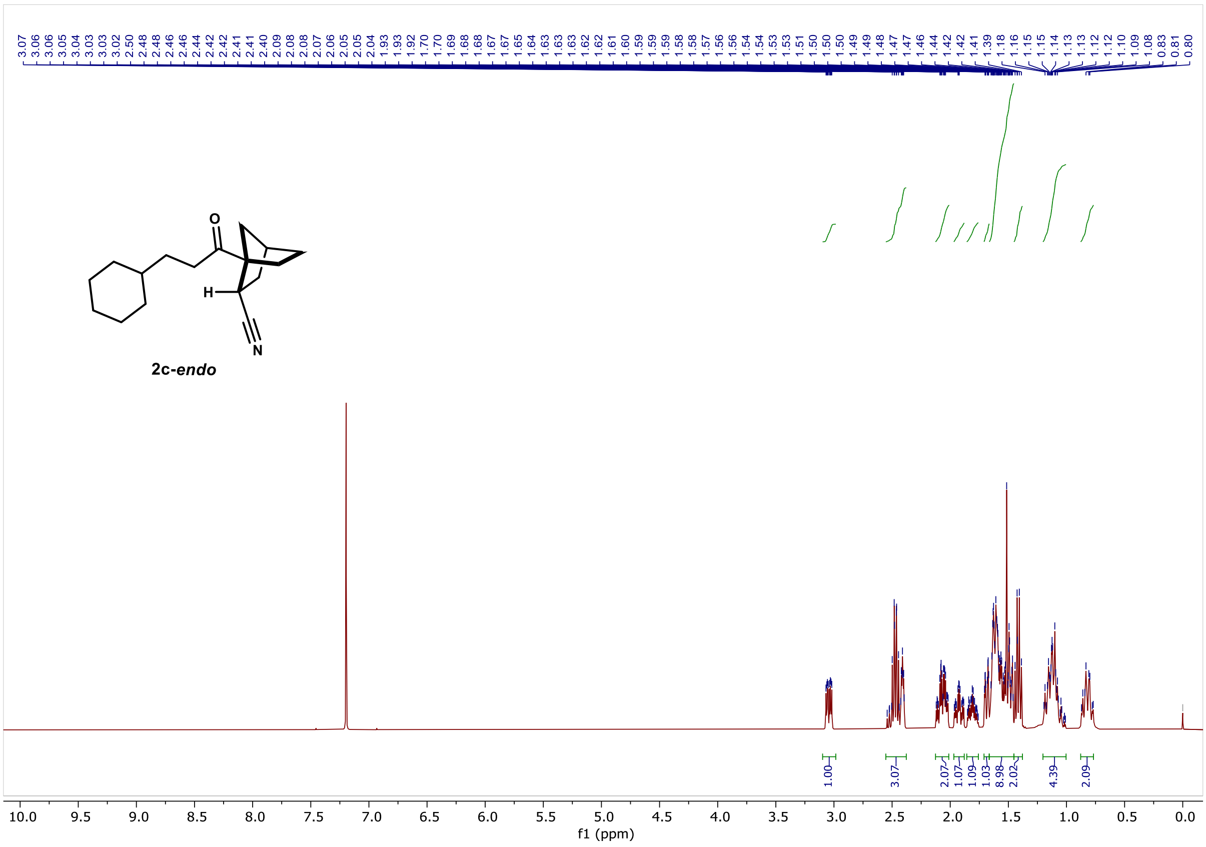

^{13}C NMR (101 MHz, Chloroform-*d*) of **2c-*endo***:

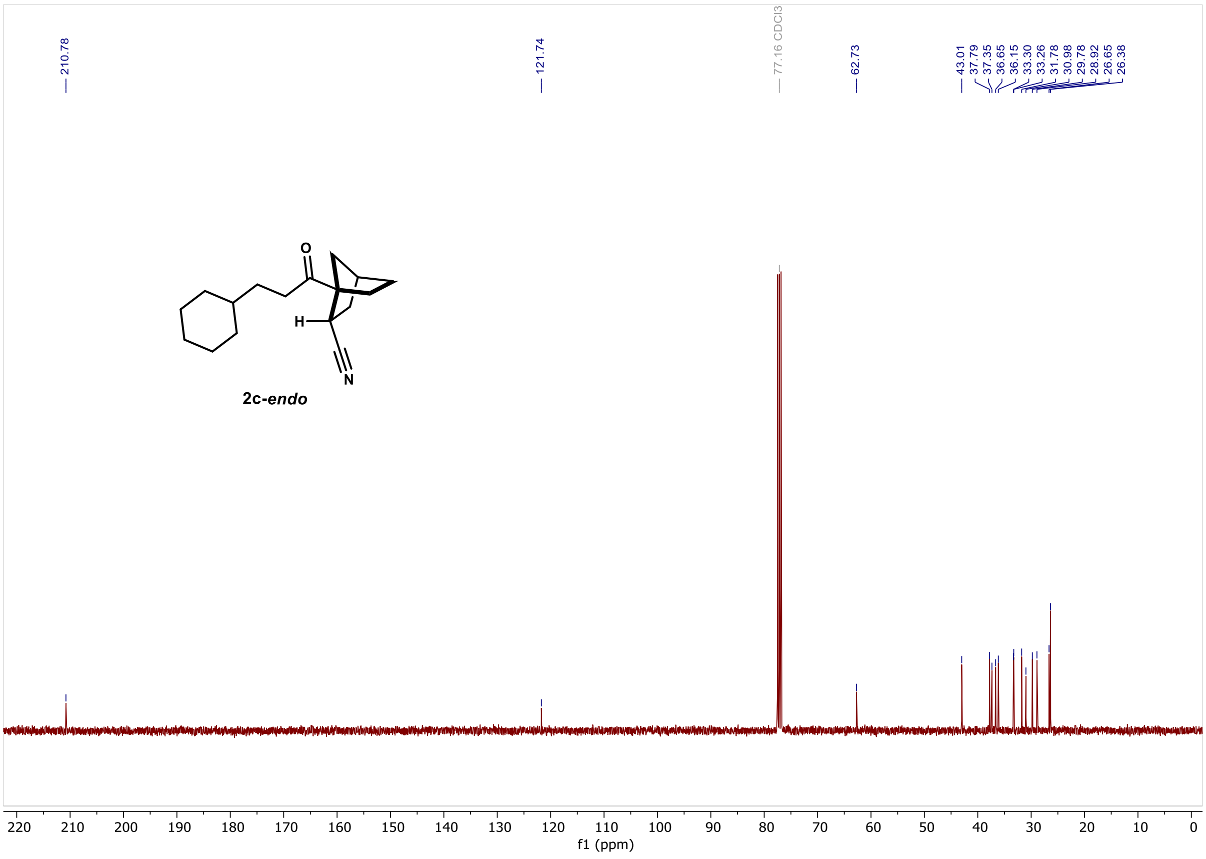

^{1}H NMR (400 MHz, Chloroform-*d*) of **2c-*exo***:

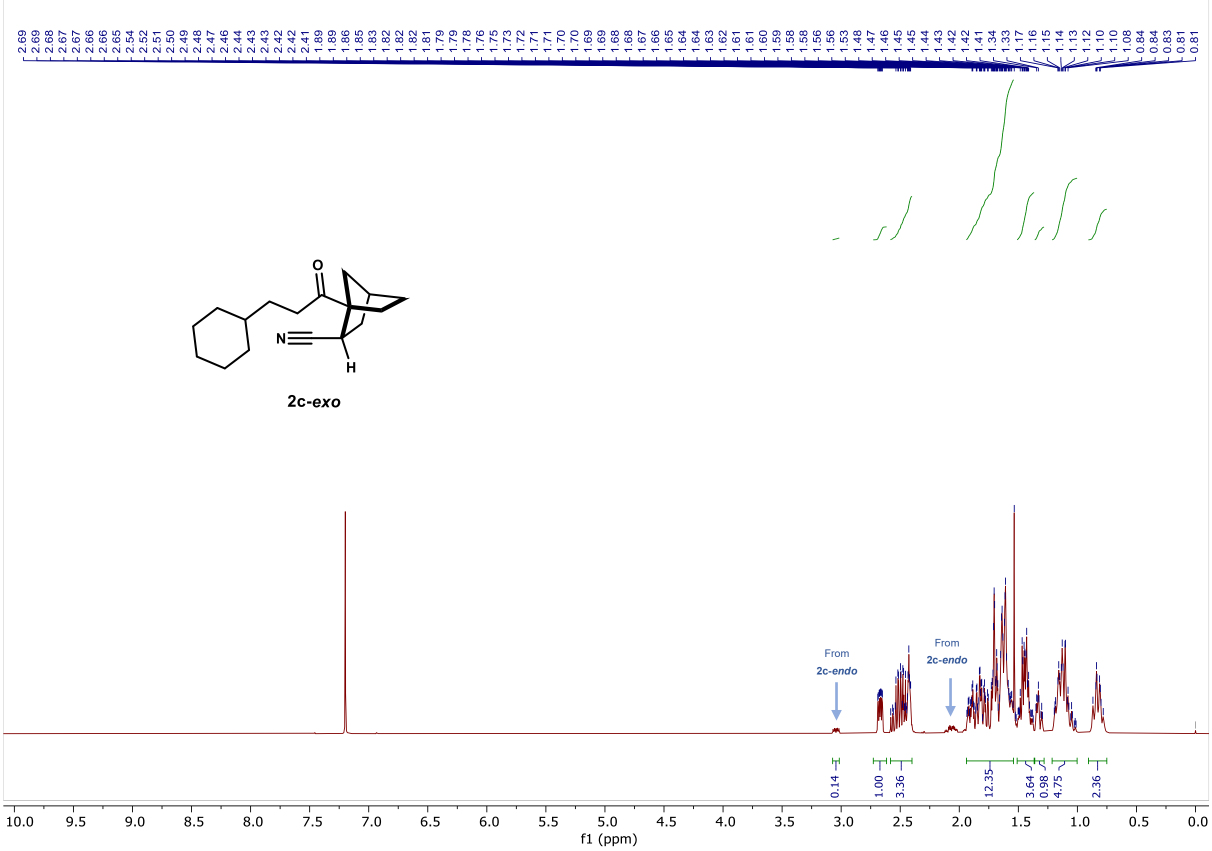

^{13}C NMR (126 MHz, Chloroform-*d*) of **2c-*exo***:

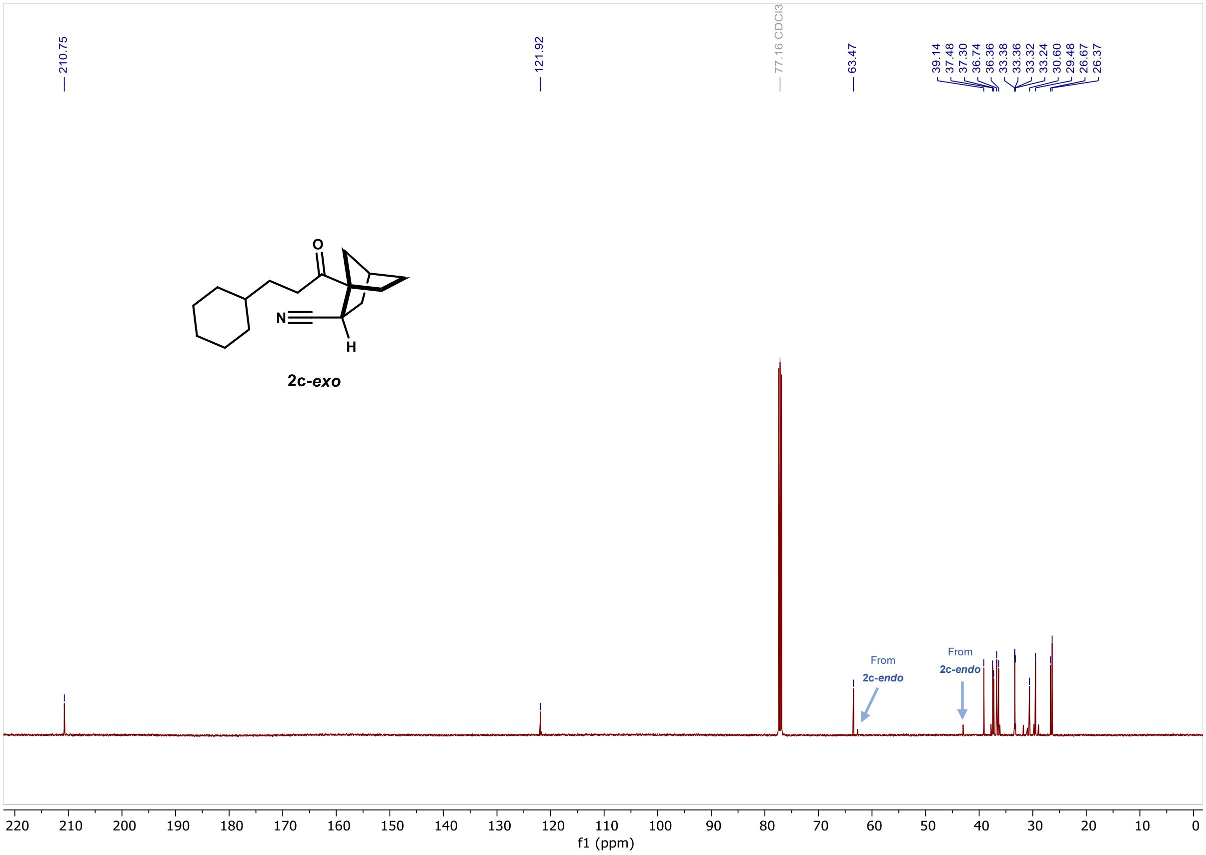

^{1}H NMR (500 MHz, Chloroform-*d*) of **2d-*endo* and 2d-*exo***:

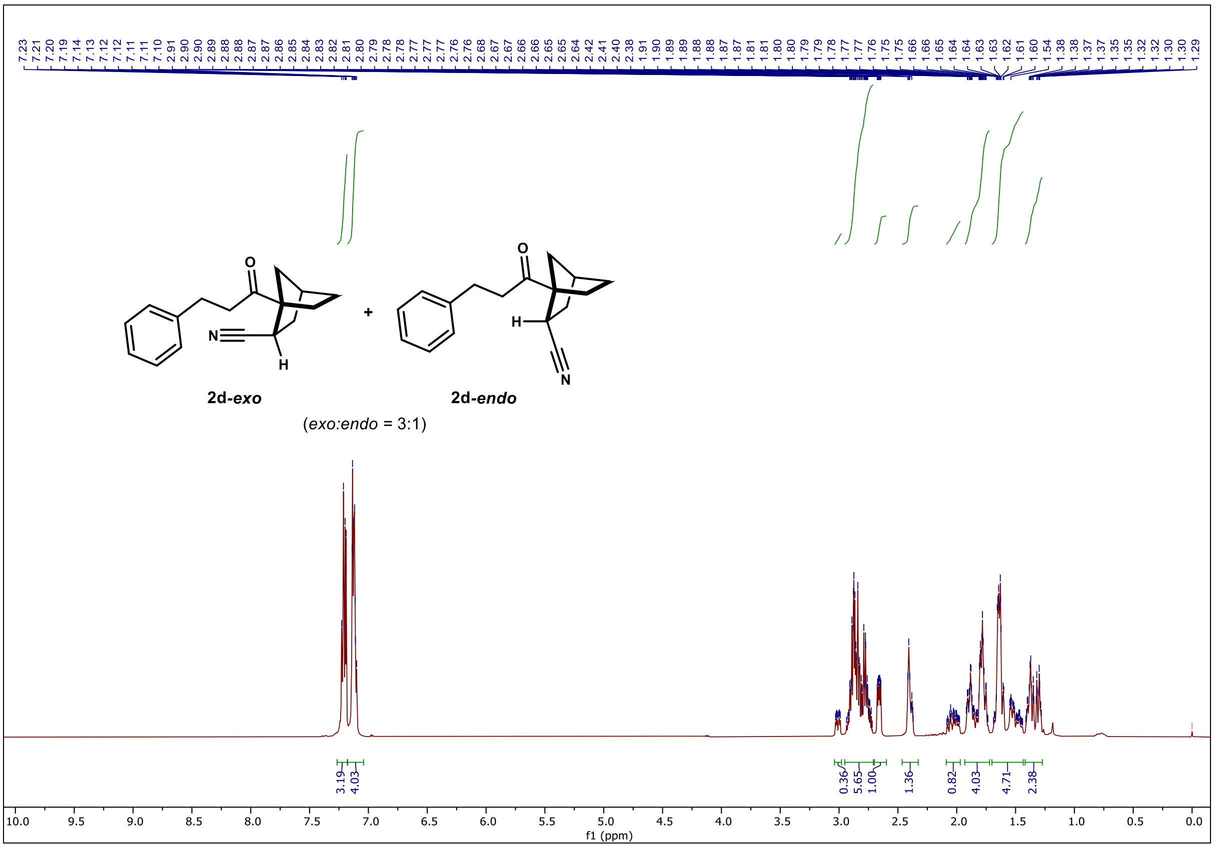

^{13}C NMR (126 MHz, Chloroform-*d*) of **2d-*endo* and 2d-*exo***:

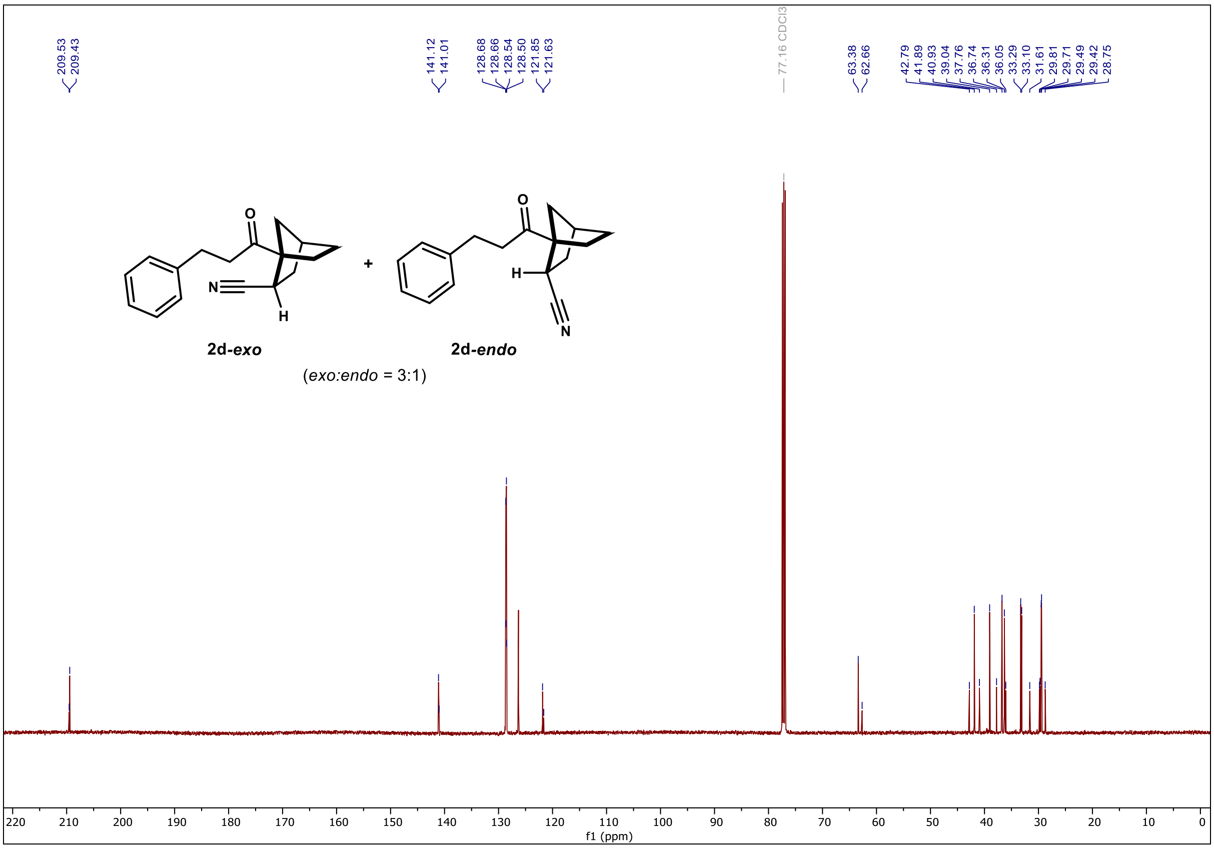

^{1}H NMR (400 MHz, Chloroform-*d*) of **2e-*endo* and 2e-*exo***:

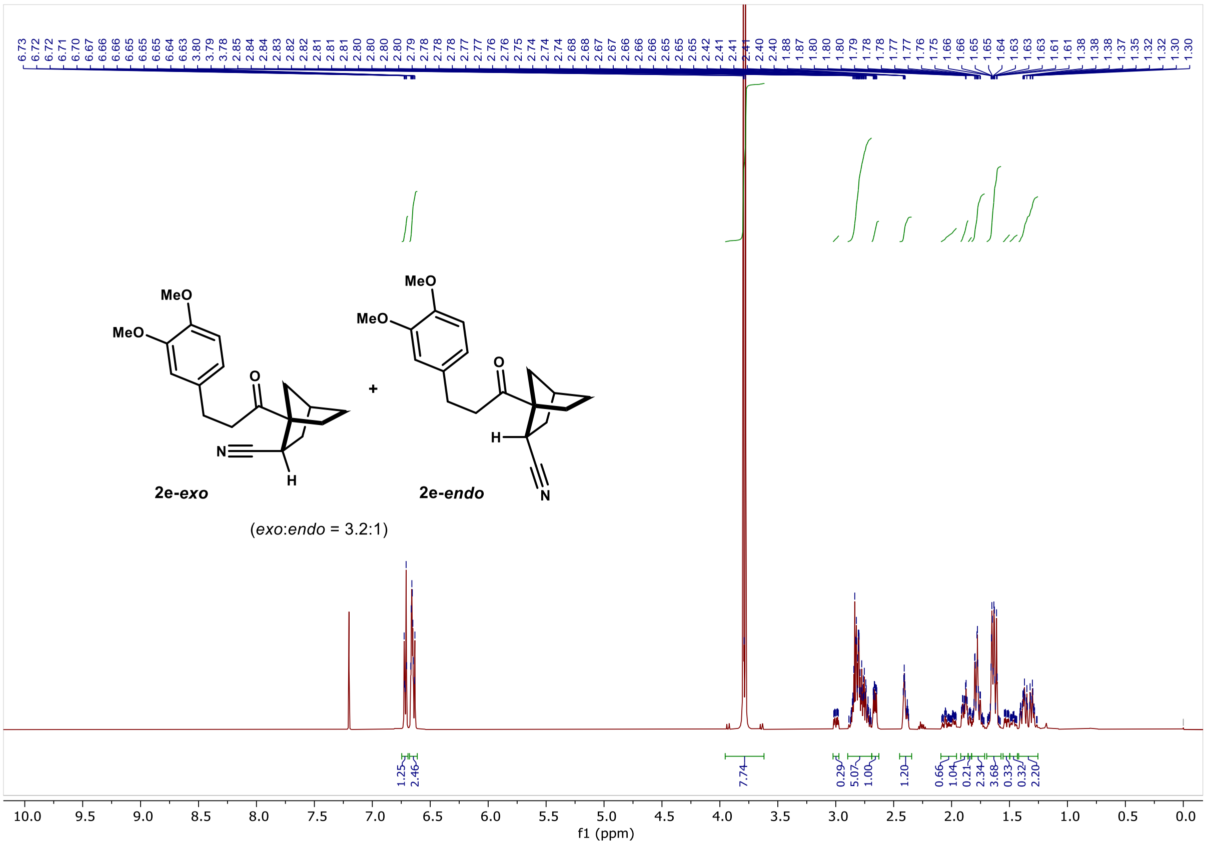

^{13}C NMR (101 MHz, Chloroform-*d*) of **2e-*endo* and 2e-*exo***:

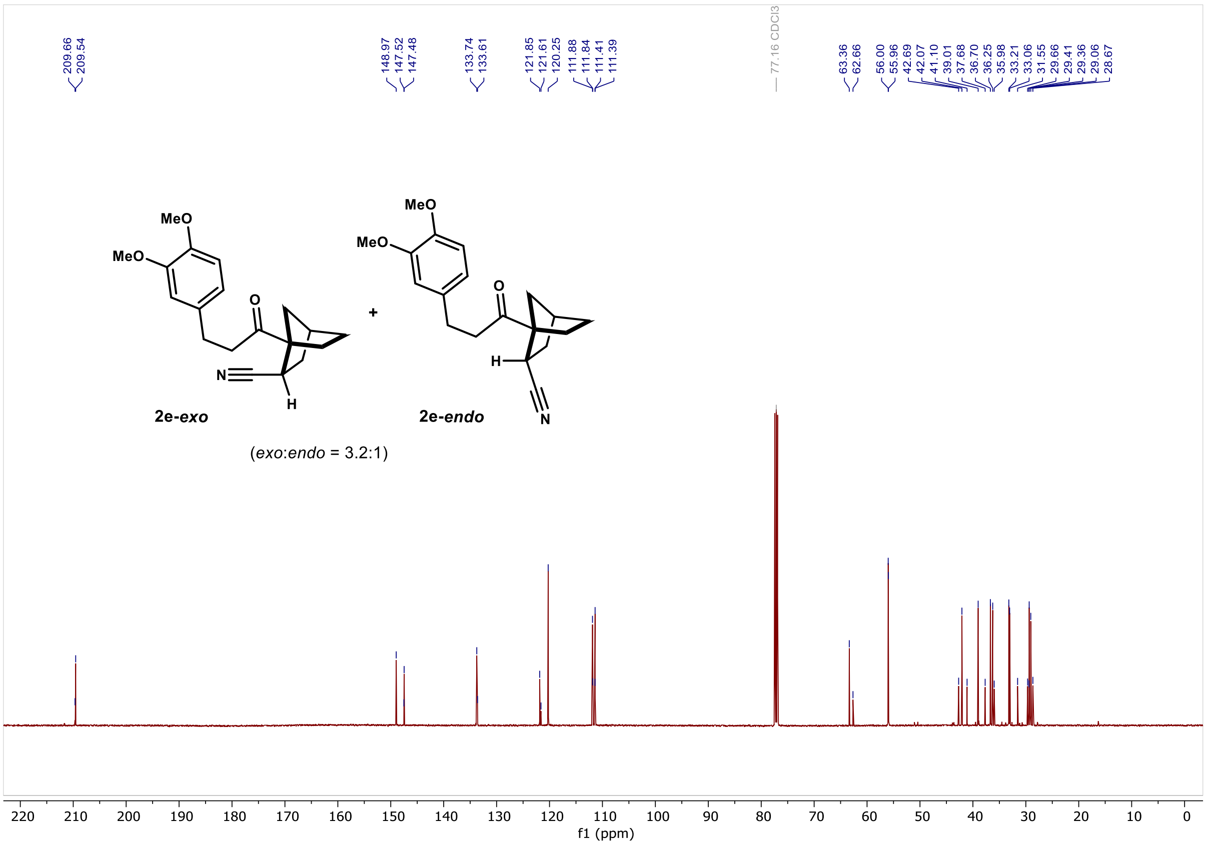

^{1}H NMR (400 MHz, Chloroform-*d*) of **2f-*endo* and 2f-*exo***:

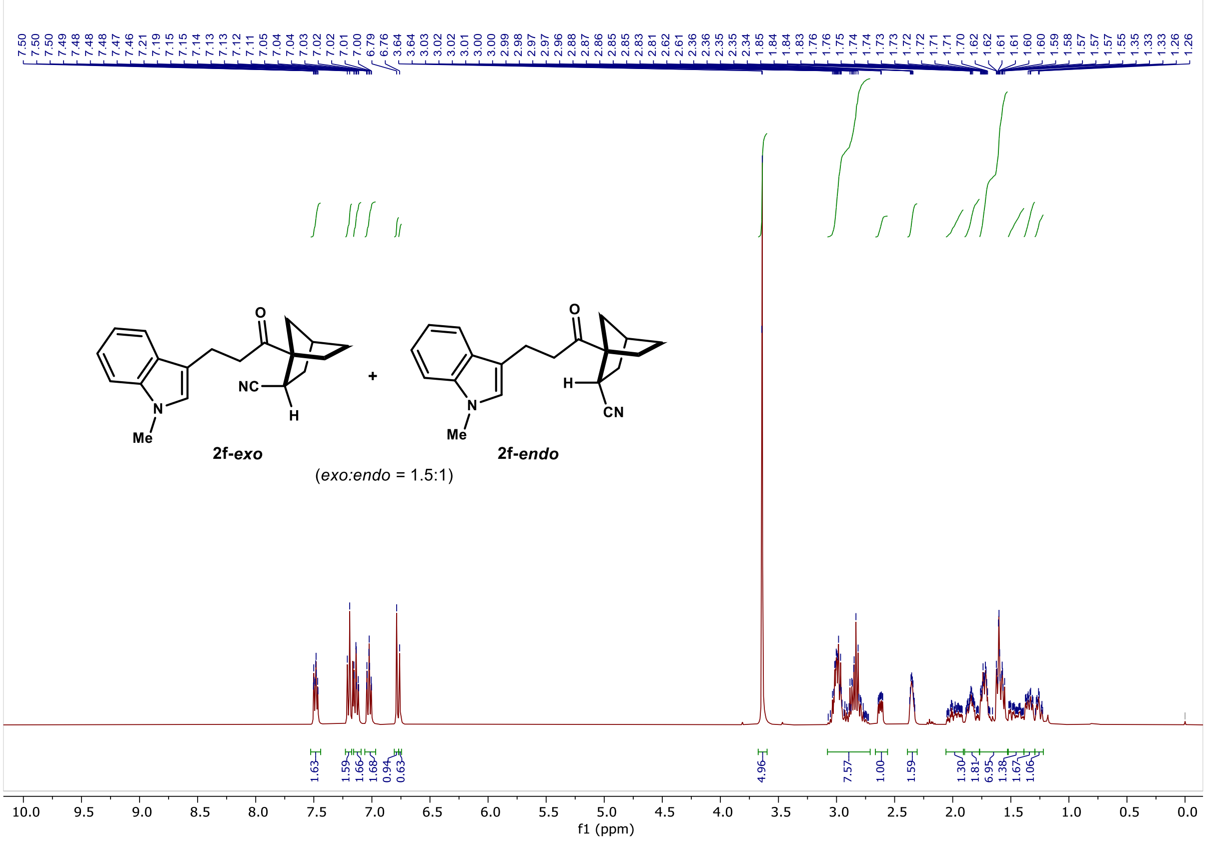

^{13}C NMR (101 MHz, Chloroform-*d*) of **2f-*endo* and 2f-*exo***:

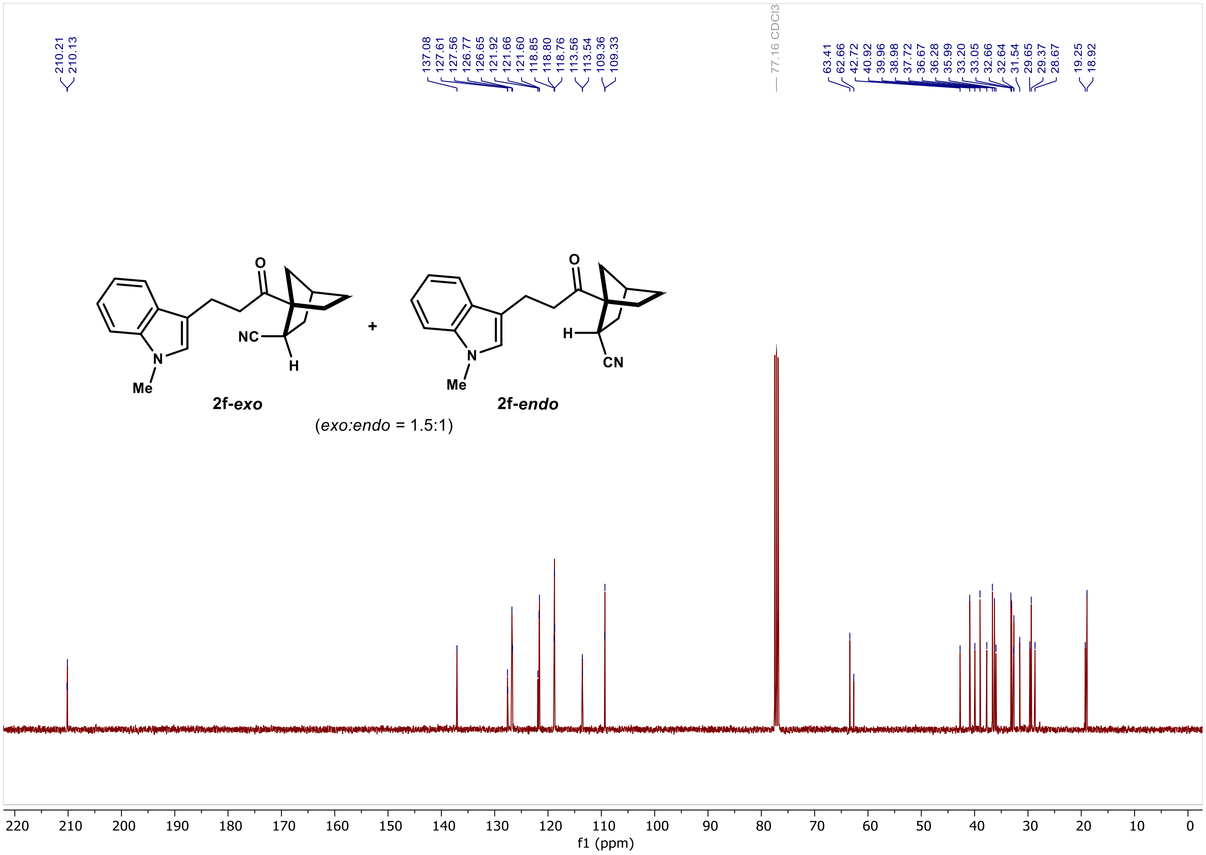

^{1}H NMR (400 MHz, Chloroform-*d*) of **2g-*endo* and 2g-*exo***:

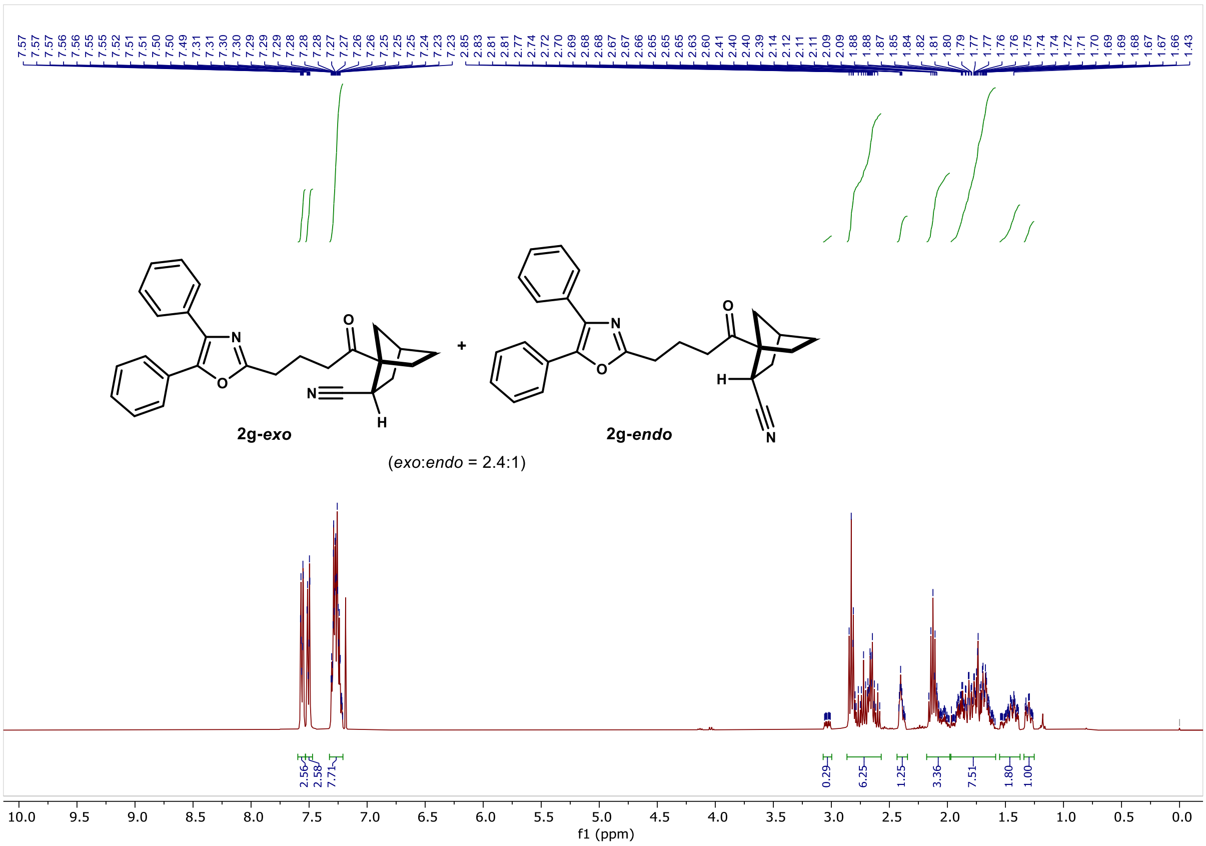

^{13}C NMR (101 MHz, Chloroform-*d*) of **2g-*endo* and 2g-*exo***:

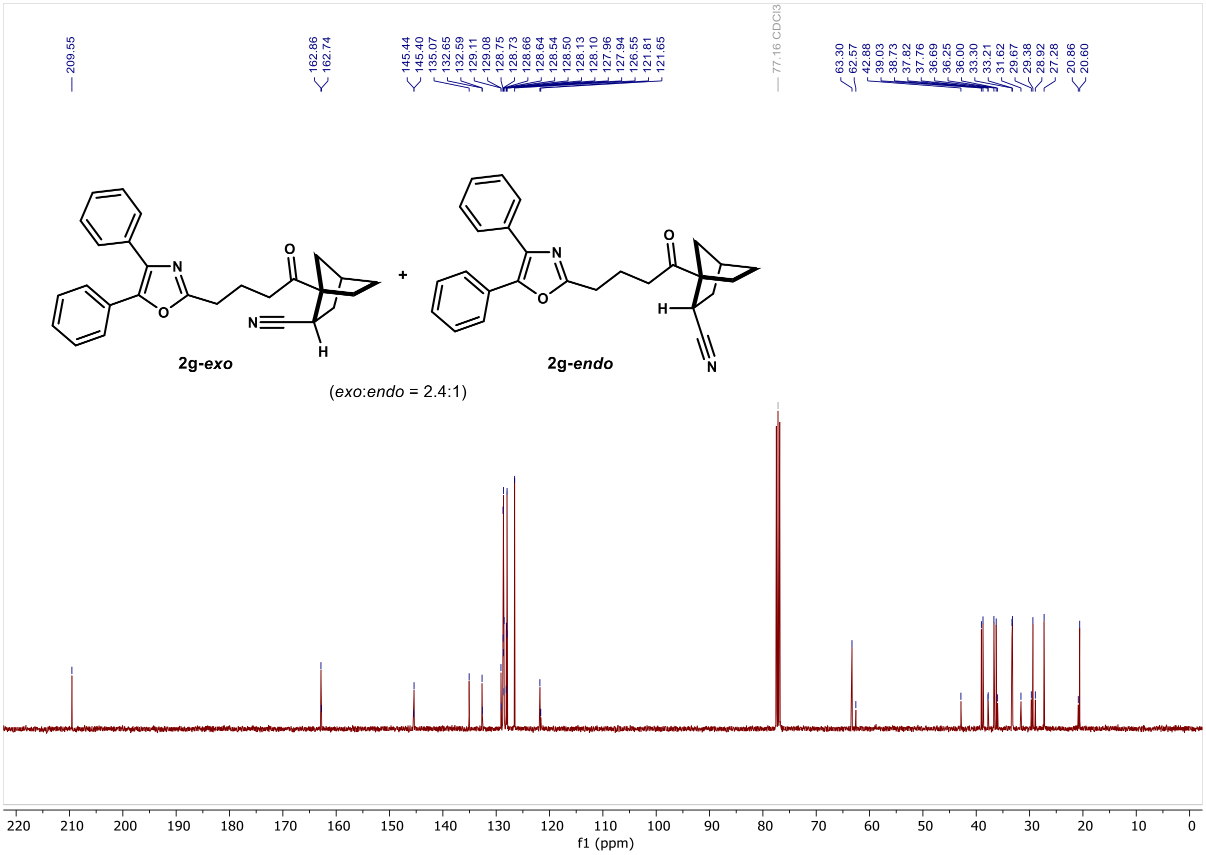

^{1}H NMR (400 MHz, Chloroform-*d*) of **2h-*endo* and 2h-*exo***:

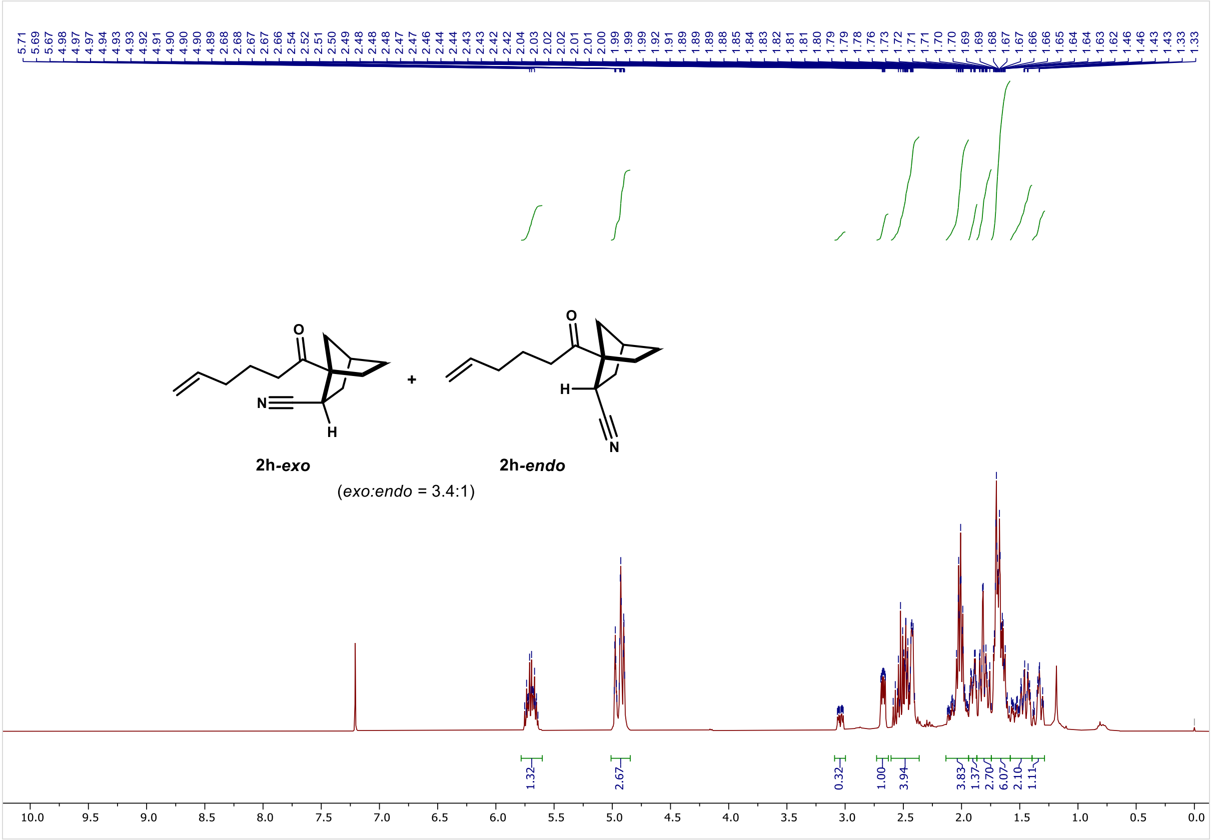

^{13}C NMR (101 MHz, Chloroform-*d*) of **2h-*endo* and 2h-*exo***:

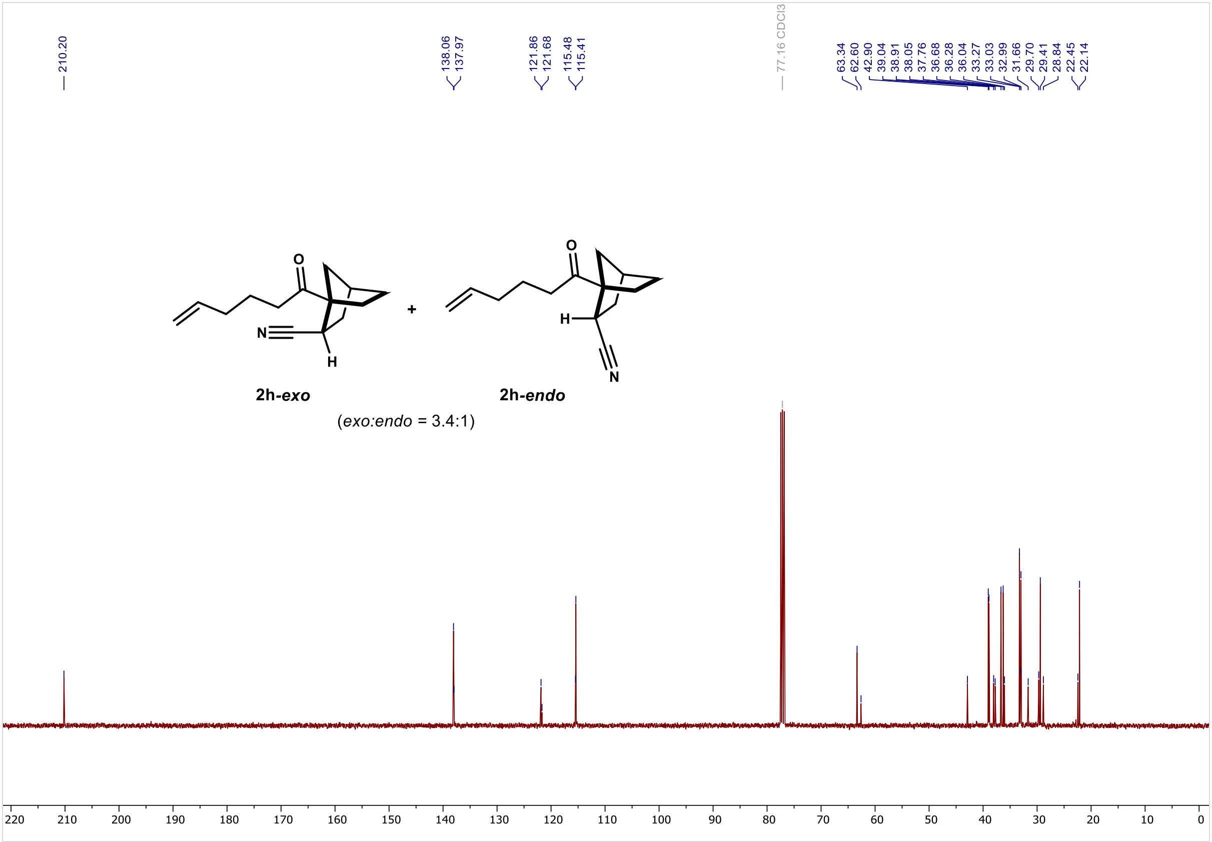

^{1}H NMR (500 MHz, Chloroform-*d*) of **2i-*endo* and 2i-*exo***:

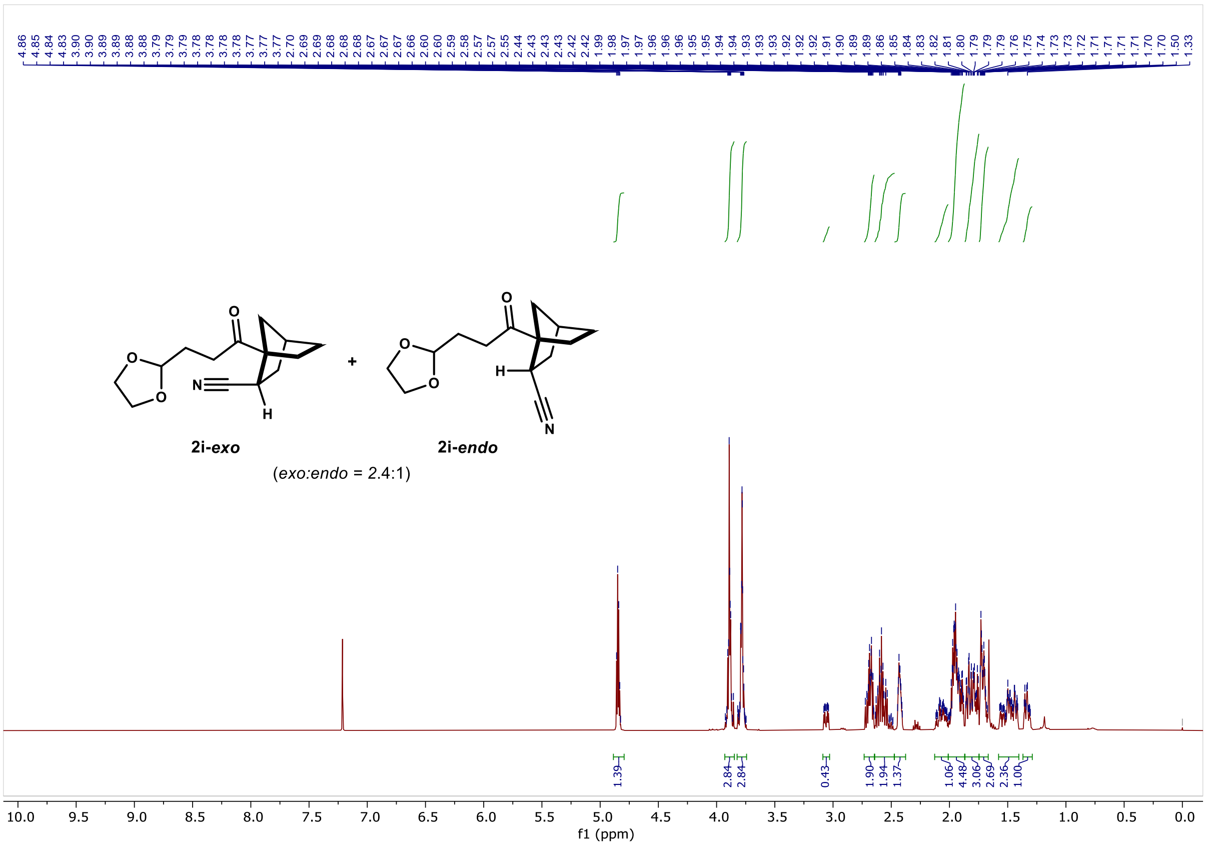

^{13}C NMR (126 MHz, Chloroform-*d*) of **2i-*endo* and 2i-*exo***:

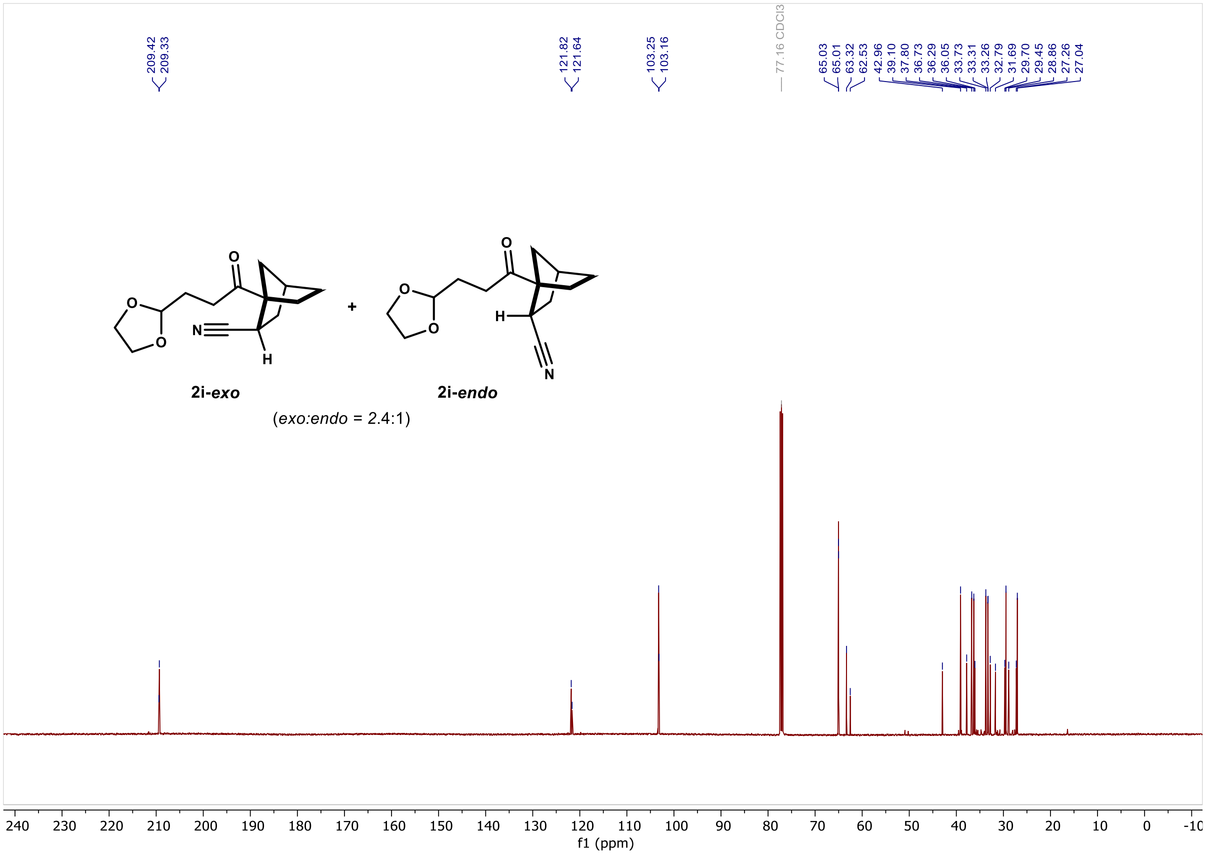

^{1}H NMR (500 MHz, Chloroform-*d*) of **2j-*endo***:

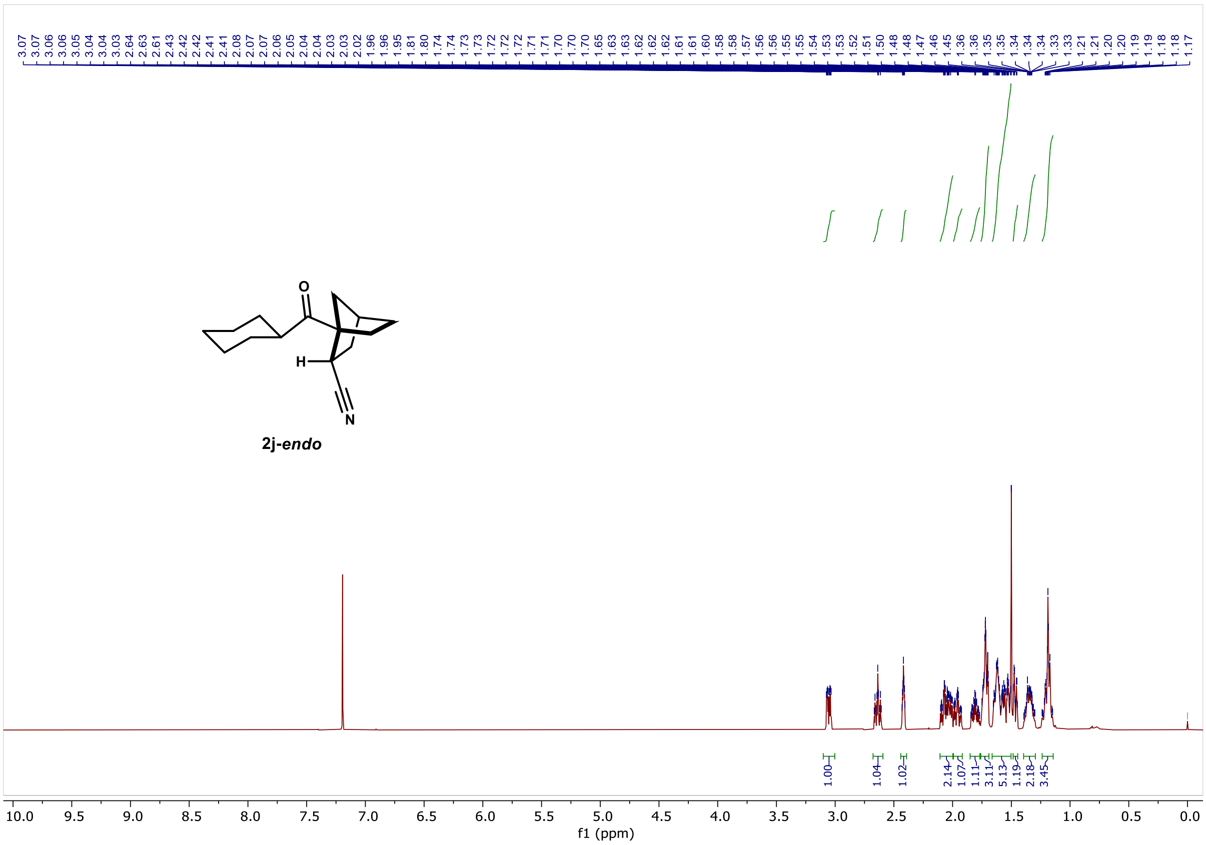

^{13}C NMR (101 MHz, Chloroform-*d*) of **2j-*endo***:

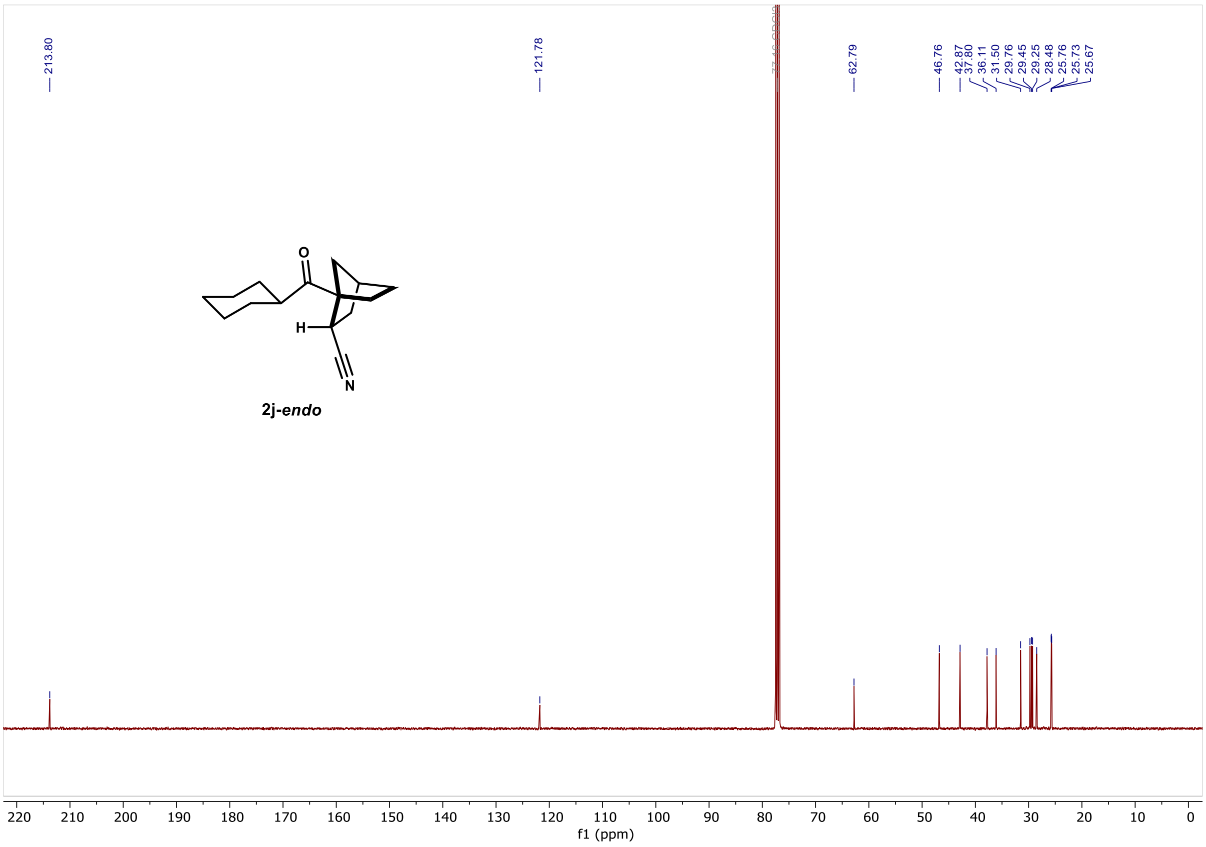

^{1}H NMR (400 MHz, Chloroform-*d*) of **2j-*exo***:

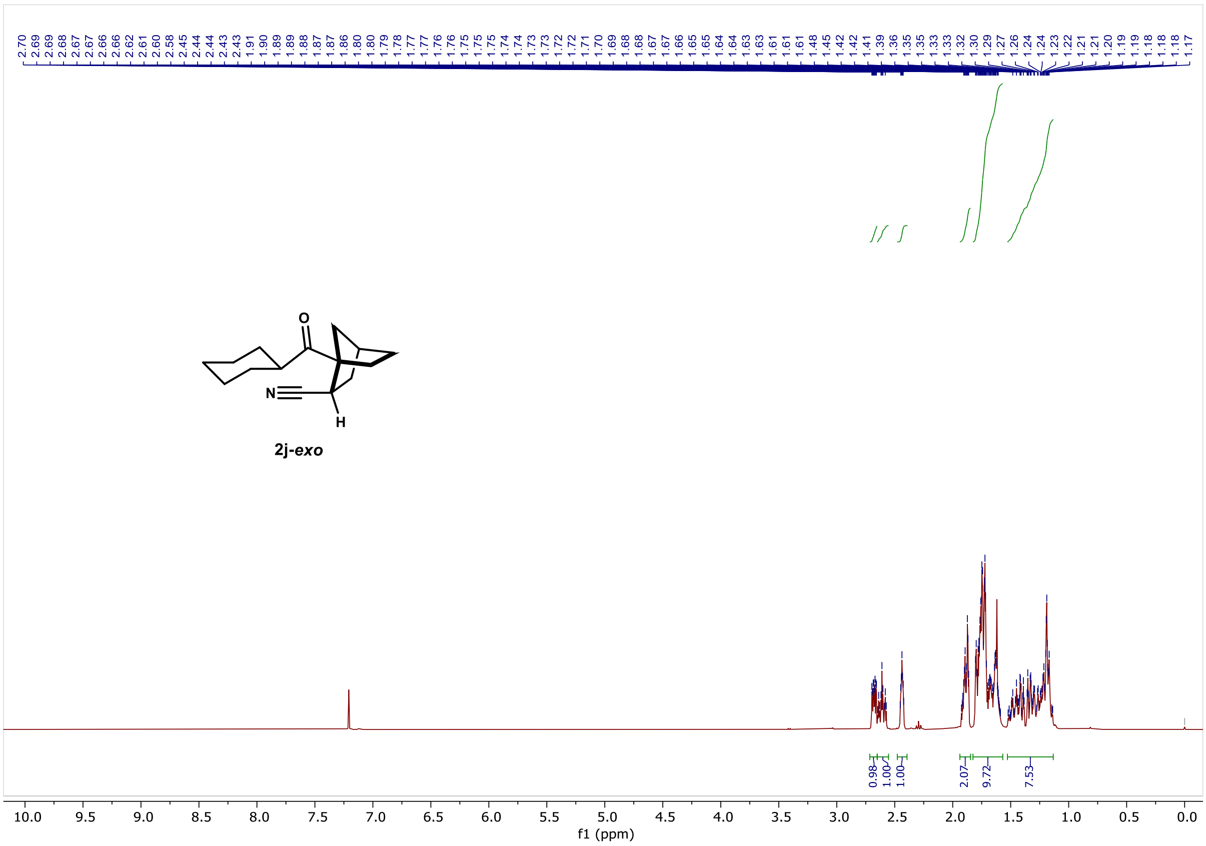

^{13}C NMR (126 MHz, Chloroform-*d*) of **2j-*exo***:

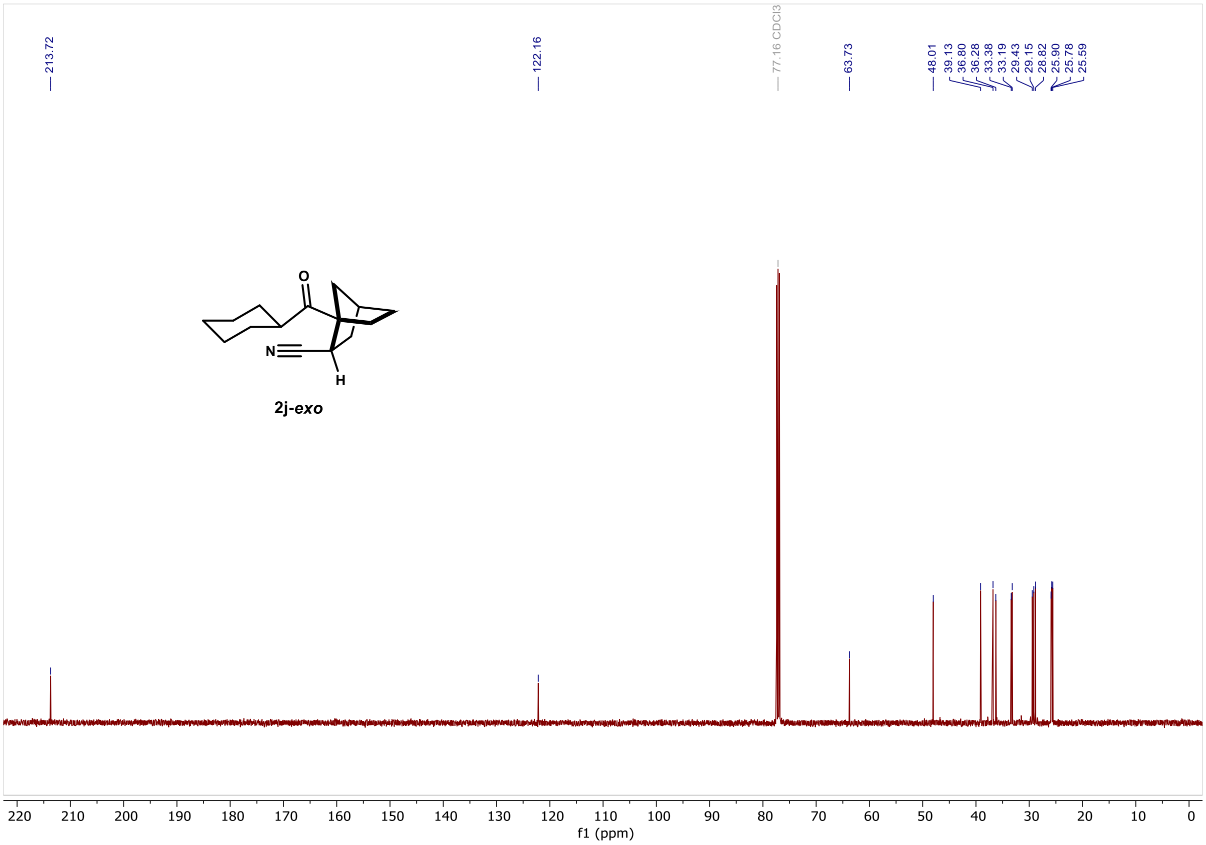

^{1}H NMR (400 MHz, Chloroform-*d*) of **2k-*endo* and 2k-*exo***:

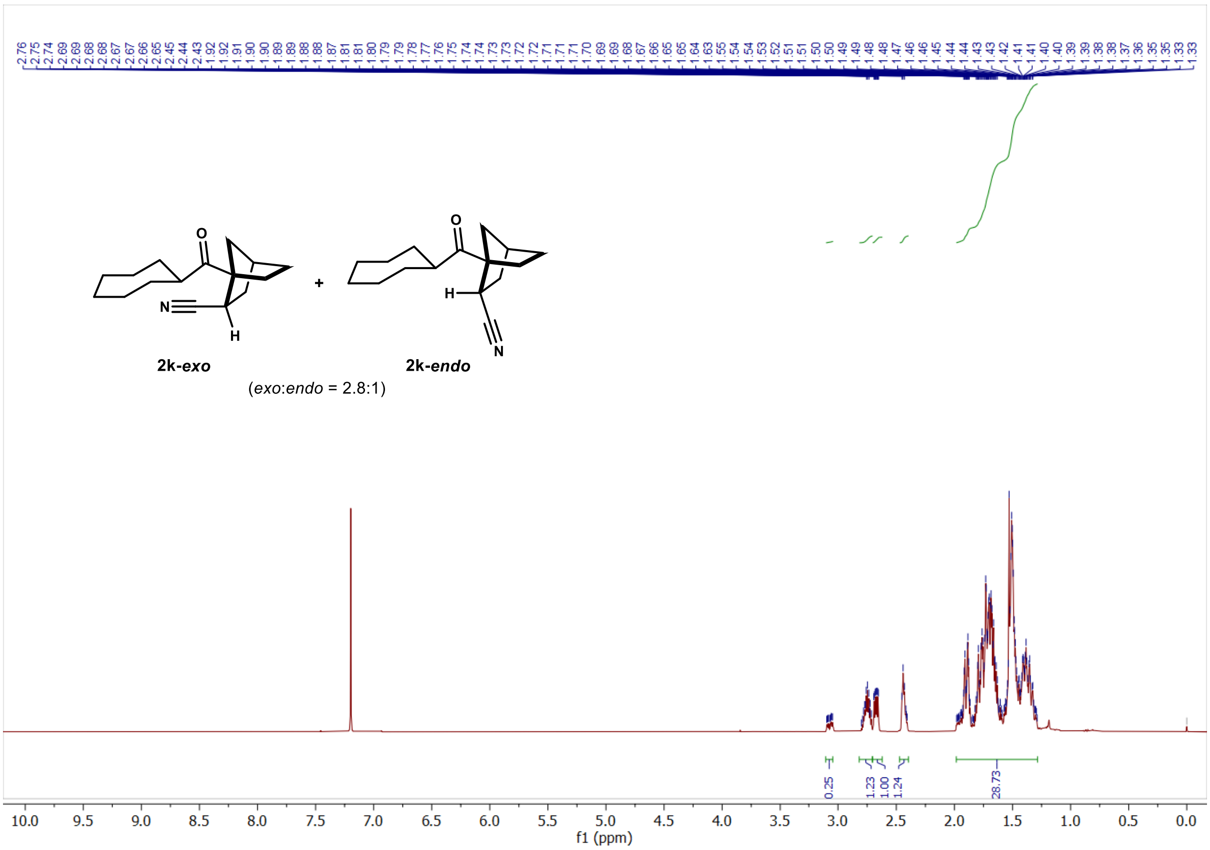

^{13}C NMR (101 MHz, Chloroform-*d*) of **2k-*endo* and 2k-*exo***:

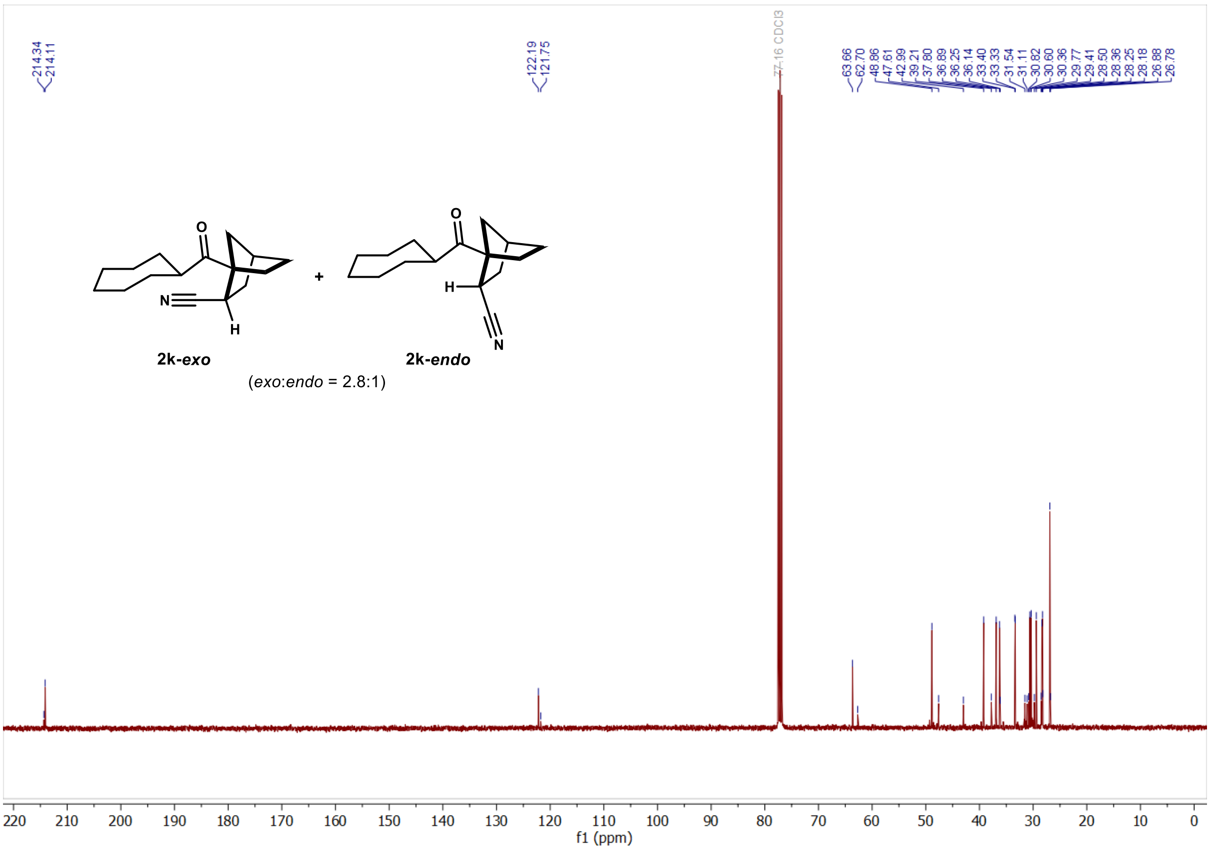

^{1}H NMR (400 MHz, Chloroform-*d*) of **2l-*endo* and 2l-*exo***:

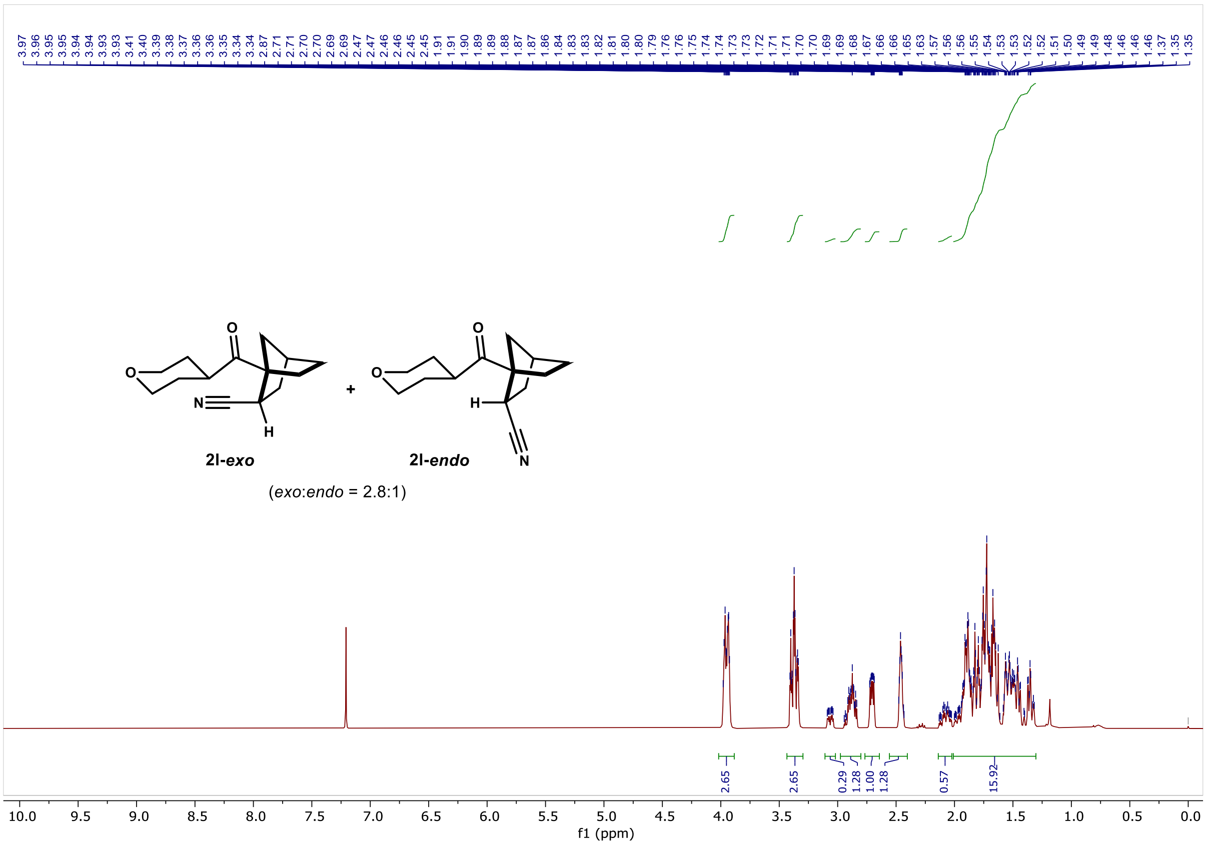

^{13}C NMR (101 MHz, Chloroform-*d*) of **2l-*endo* and 2l-*exo***:

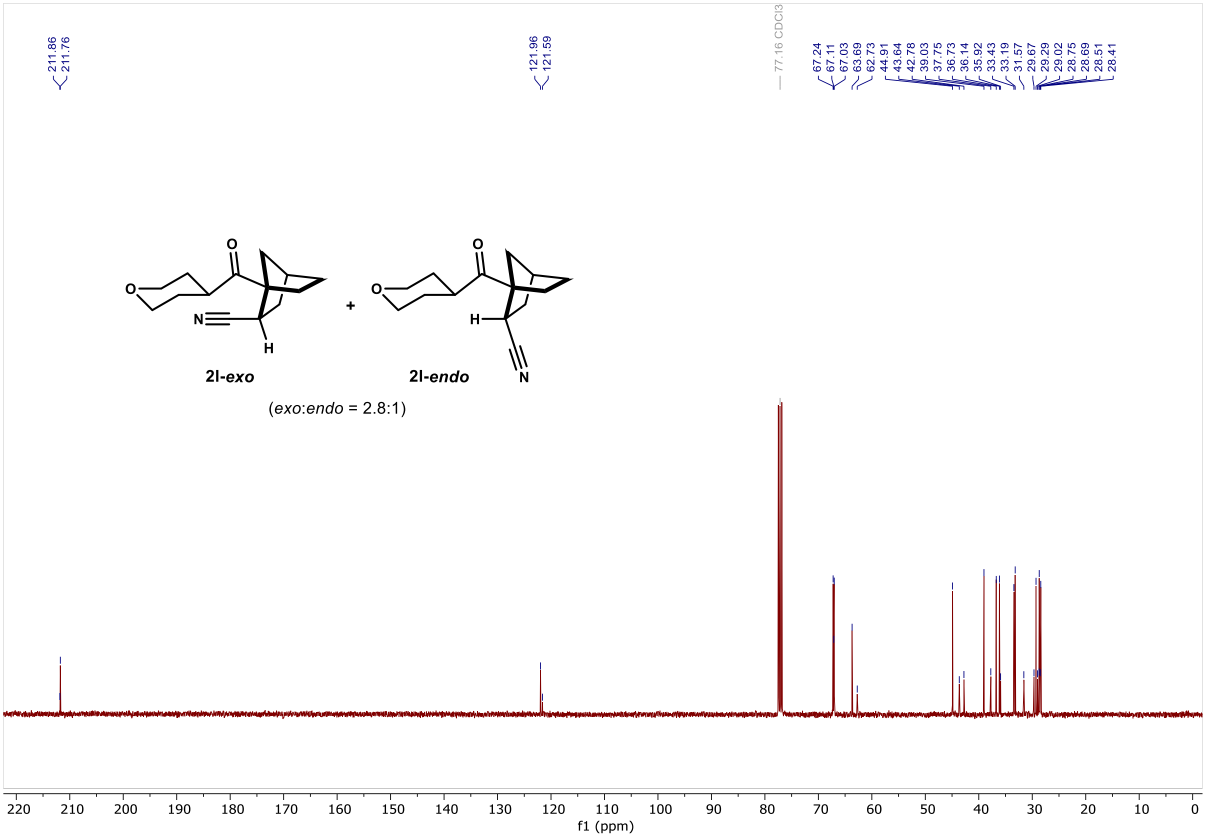

^{1}H NMR (400 MHz, Chloroform-*d*) of **2m-*endo* and 2m-*exo***:

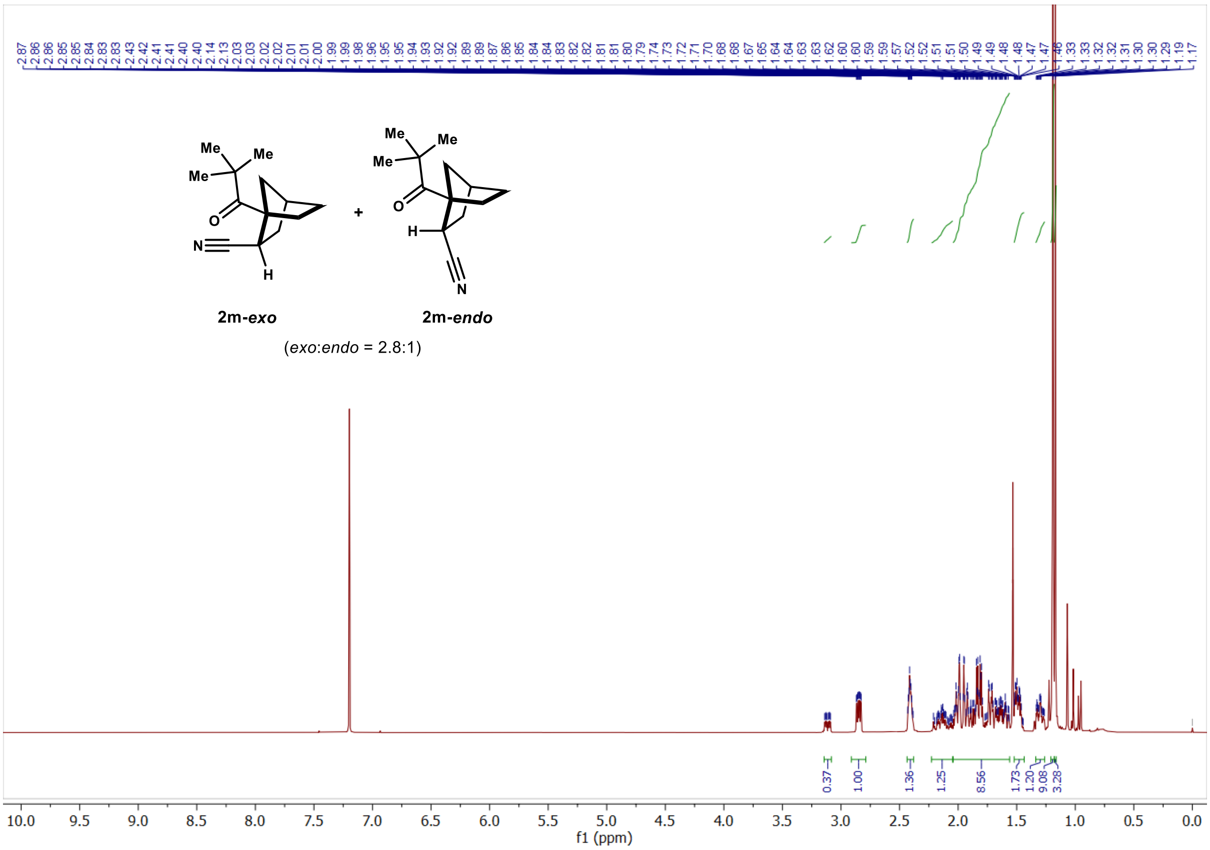

^{13}C NMR (101 MHz, Chloroform-*d*) of **2m-*endo* and 2m-*exo***:

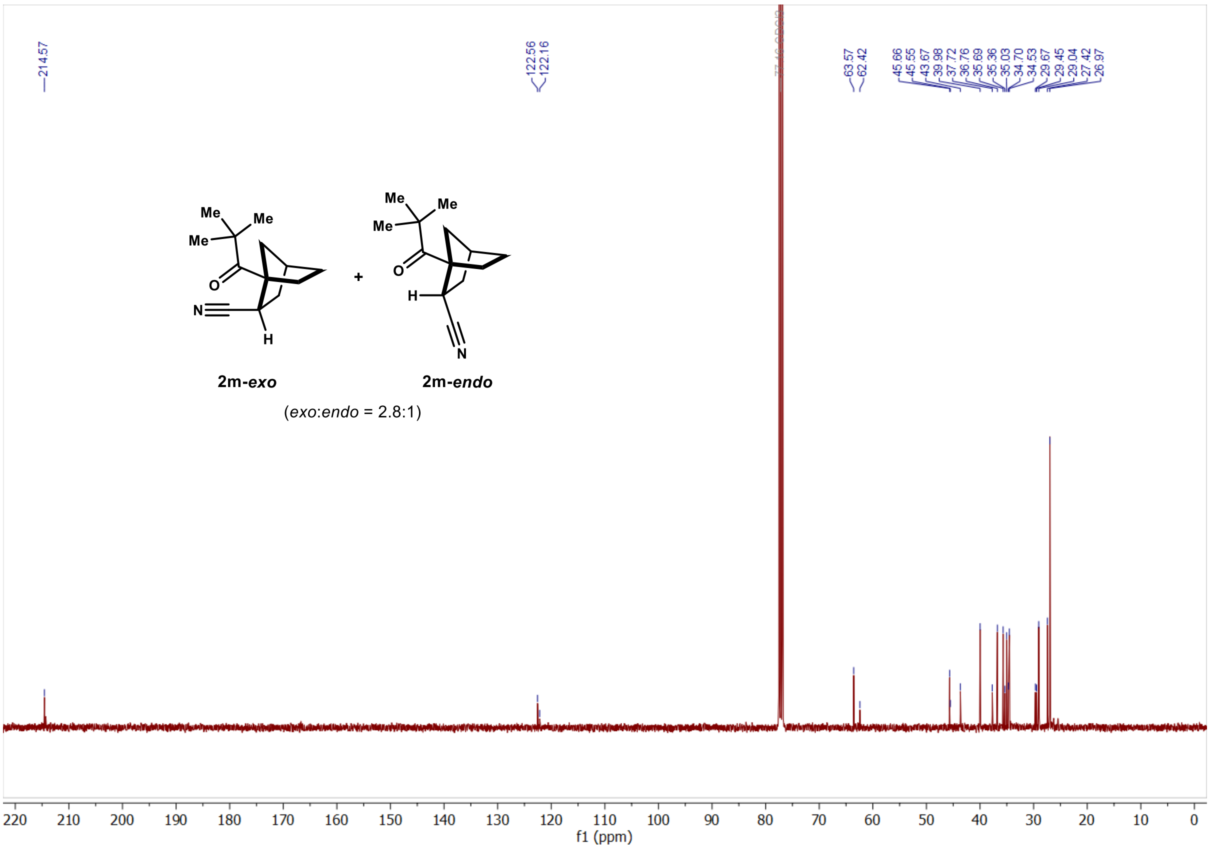

^{1}H NMR (400 MHz, Chloroform-*d*) of **2n-*endo***:

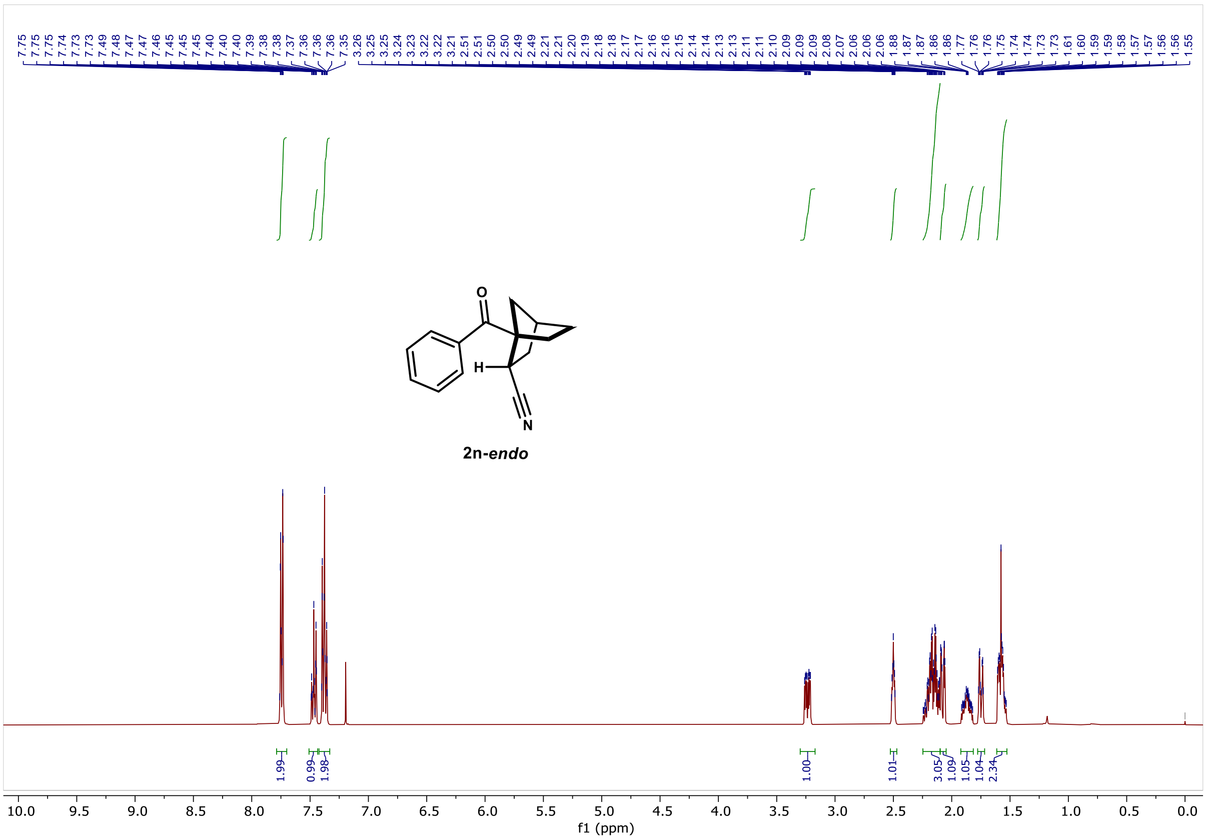

^{13}C NMR (101 MHz, Chloroform-*d*) of **2n-*endo***:

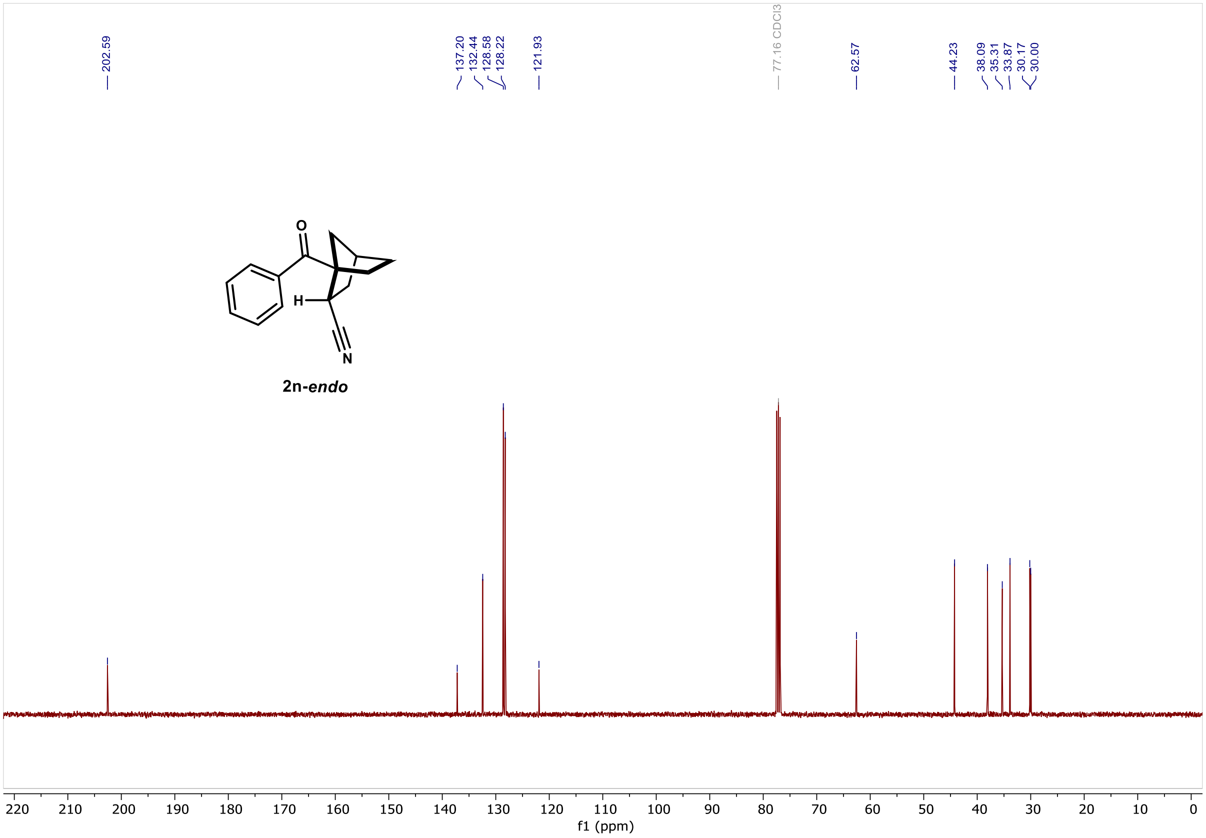

^{1}H NMR (400 MHz, Chloroform-*d*) of **2n-*exo***:

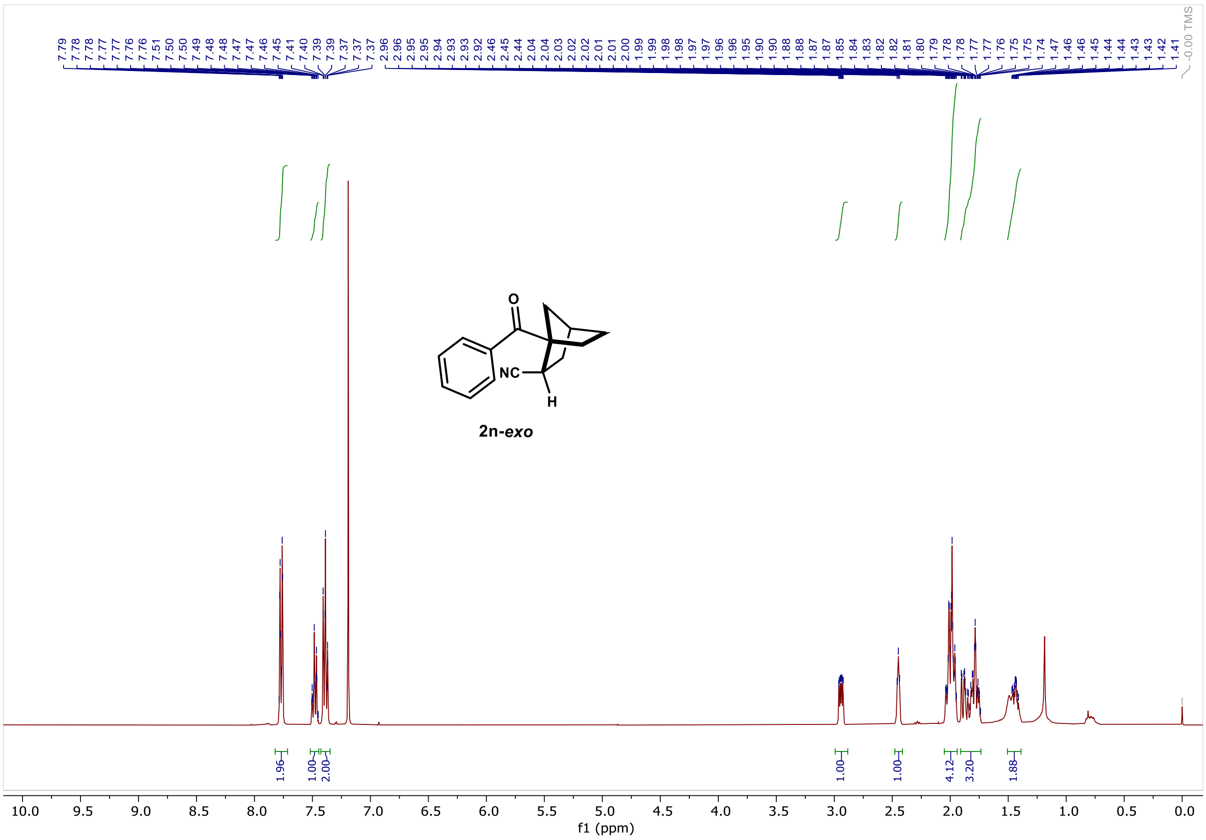

^{13}C NMR (101 MHz, Chloroform-*d*) of **2n-*exo***:

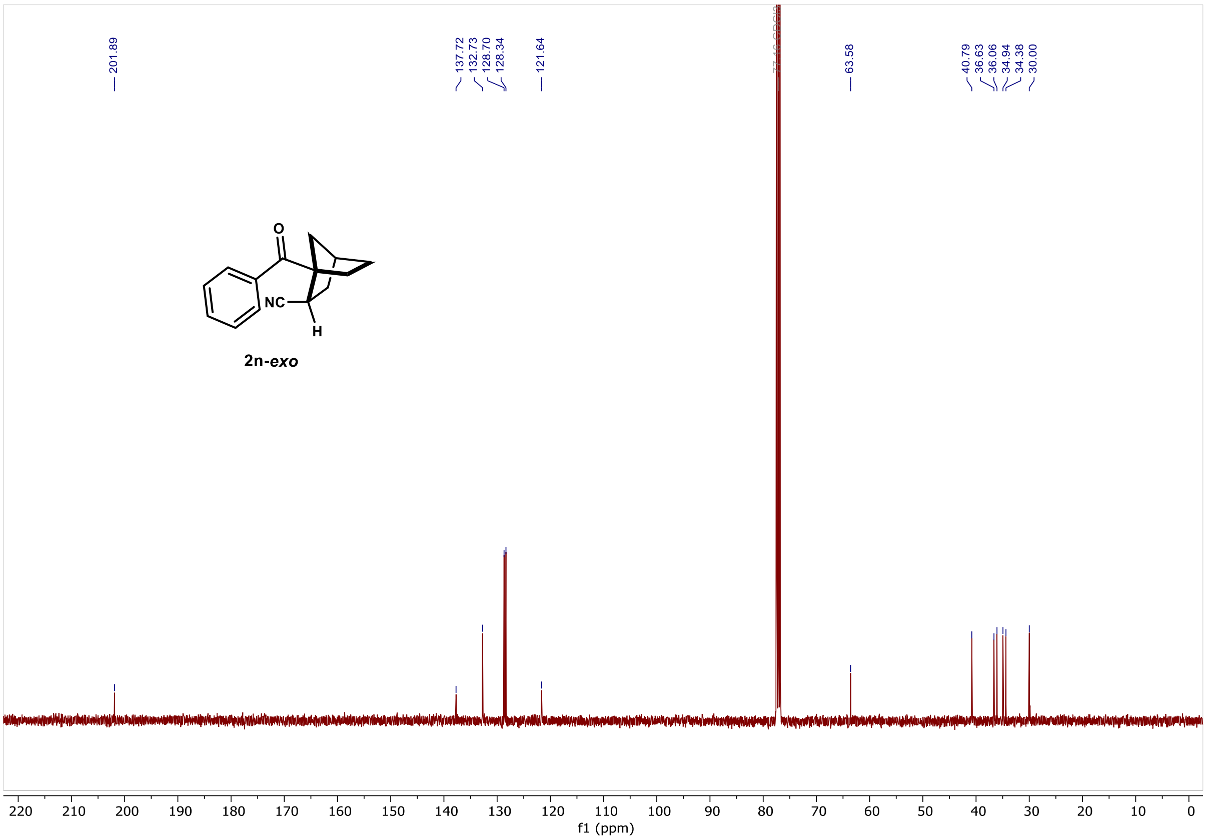

^{1}H NMR (500 MHz, Chloroform-*d*) of **2o-*endo***:

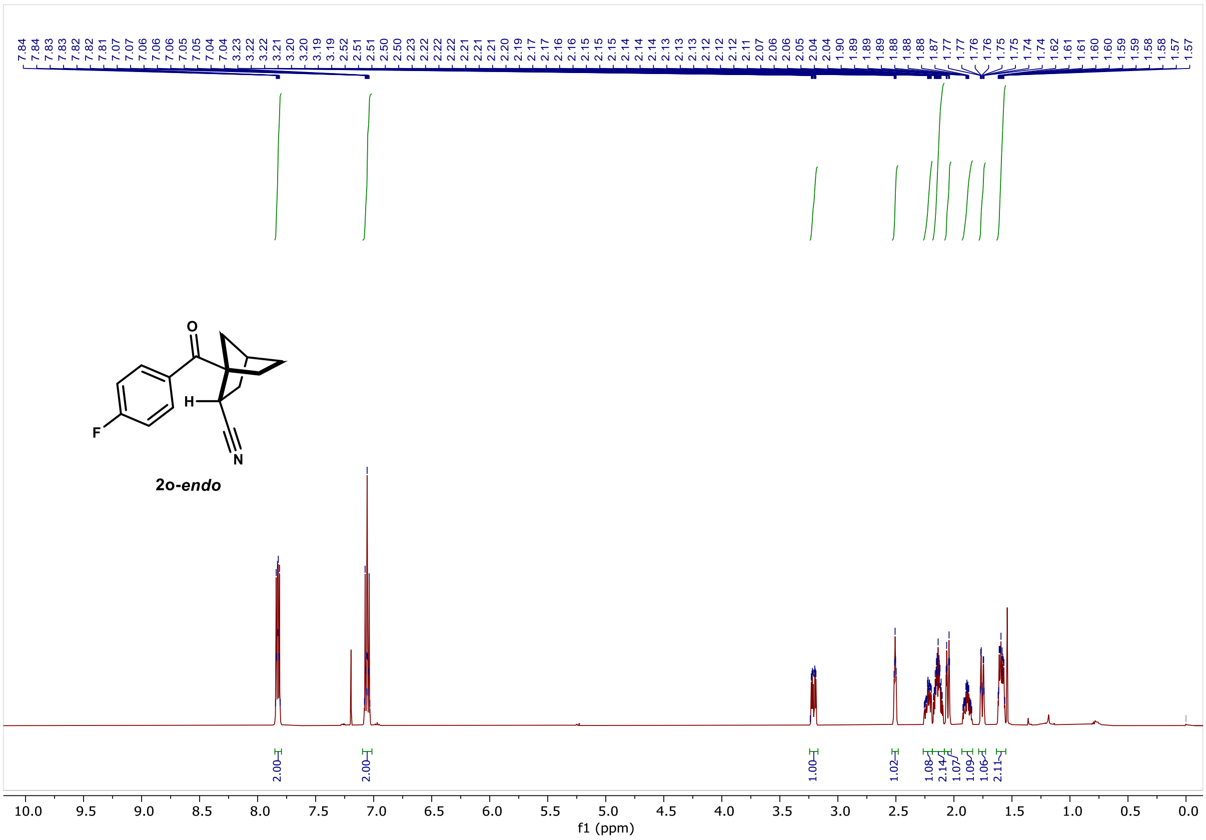

^{13}C NMR (126 MHz, Chloroform-*d*) of **2o-*endo***:

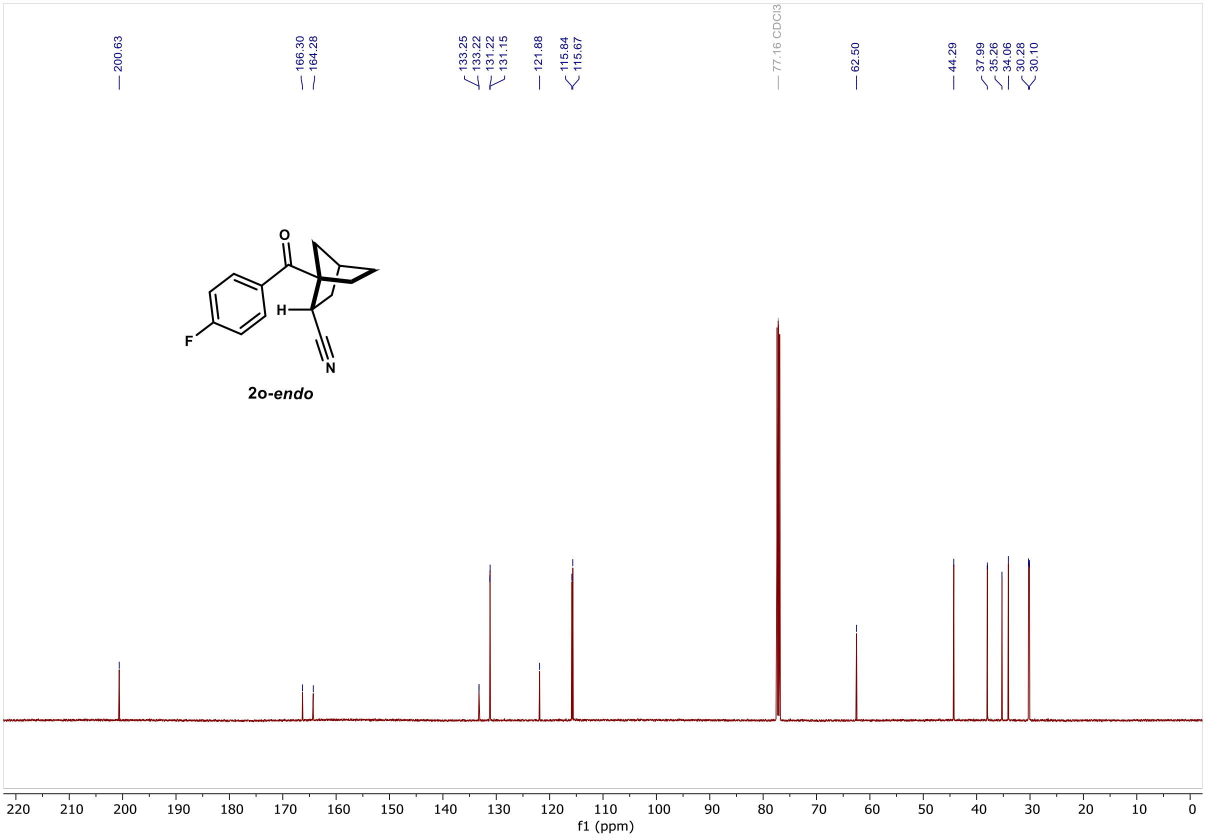

^{1}H NMR (400 MHz, Chloroform-*d*) of **2o-*exo***:

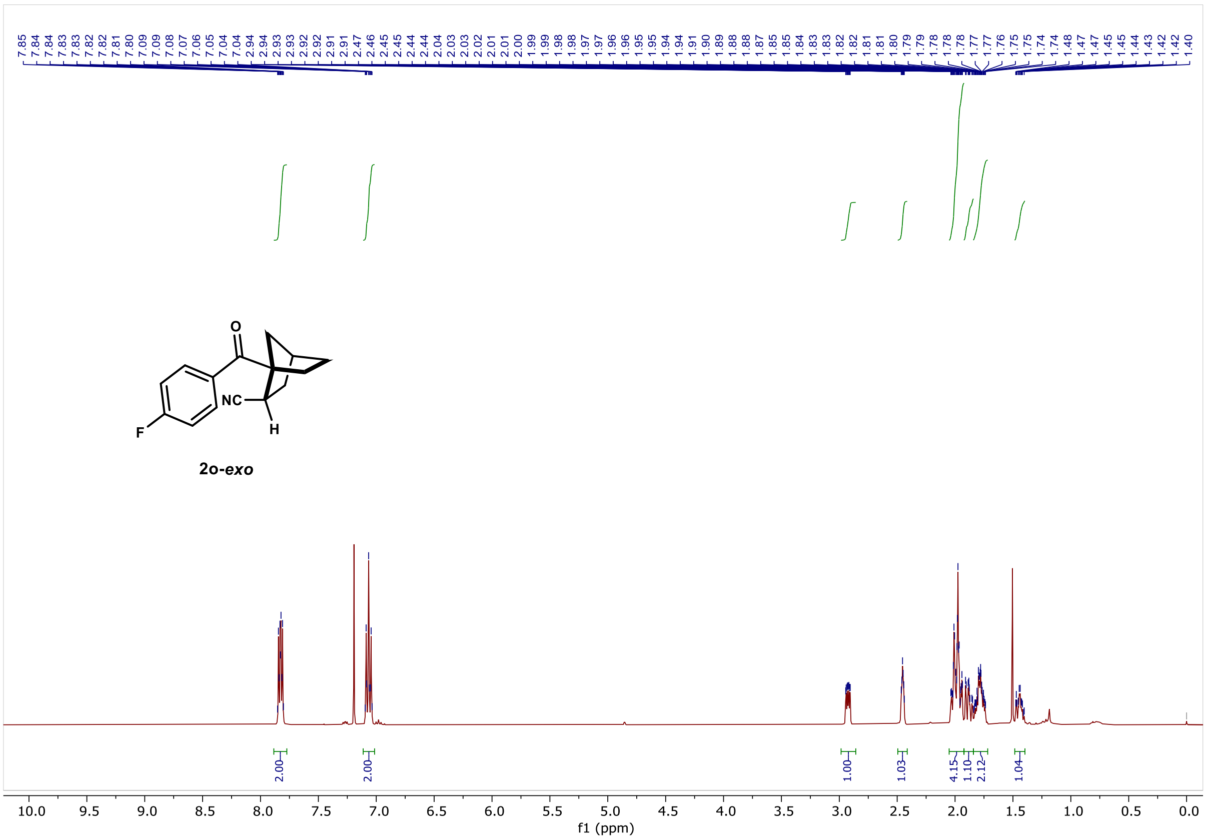

^{13}C NMR (101 MHz, Chloroform-*d*) of **2o-*exo***:

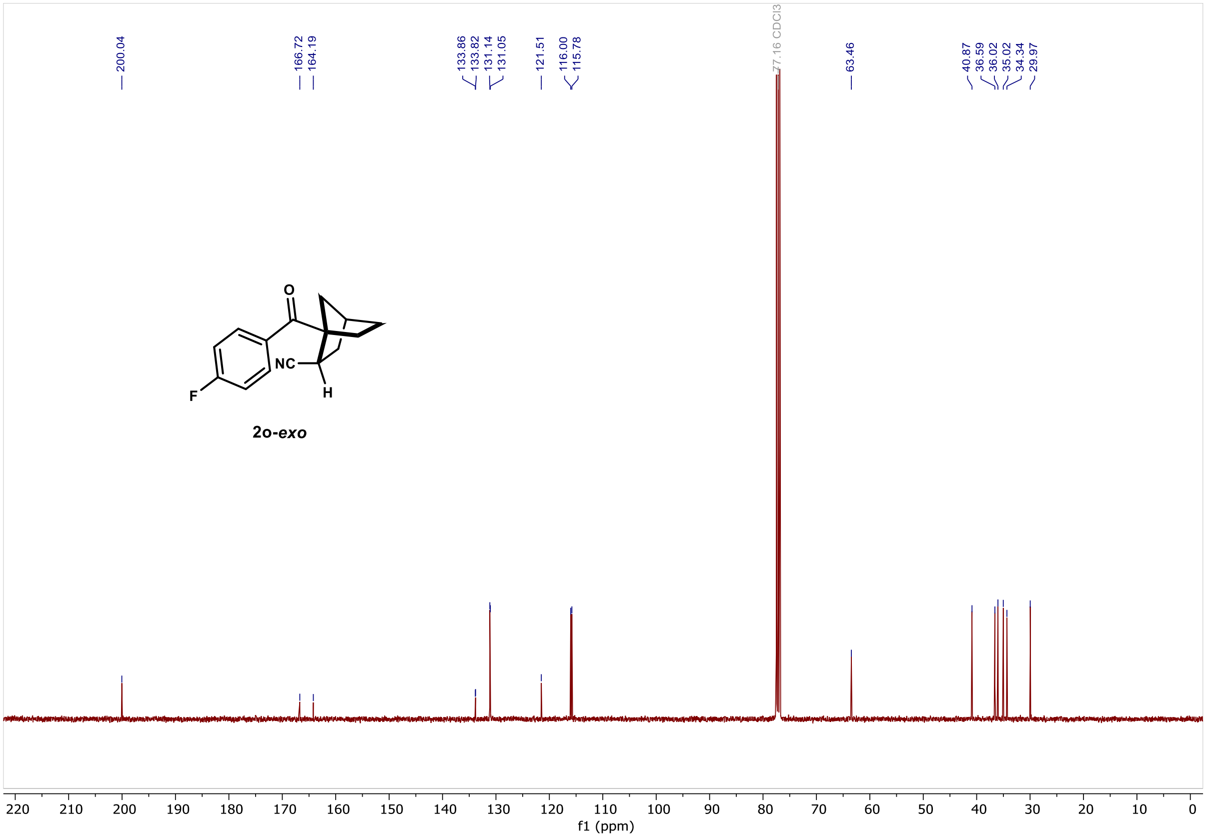

^{1}H NMR (500 MHz, Chloroform-*d*) of **2p-*endo***:

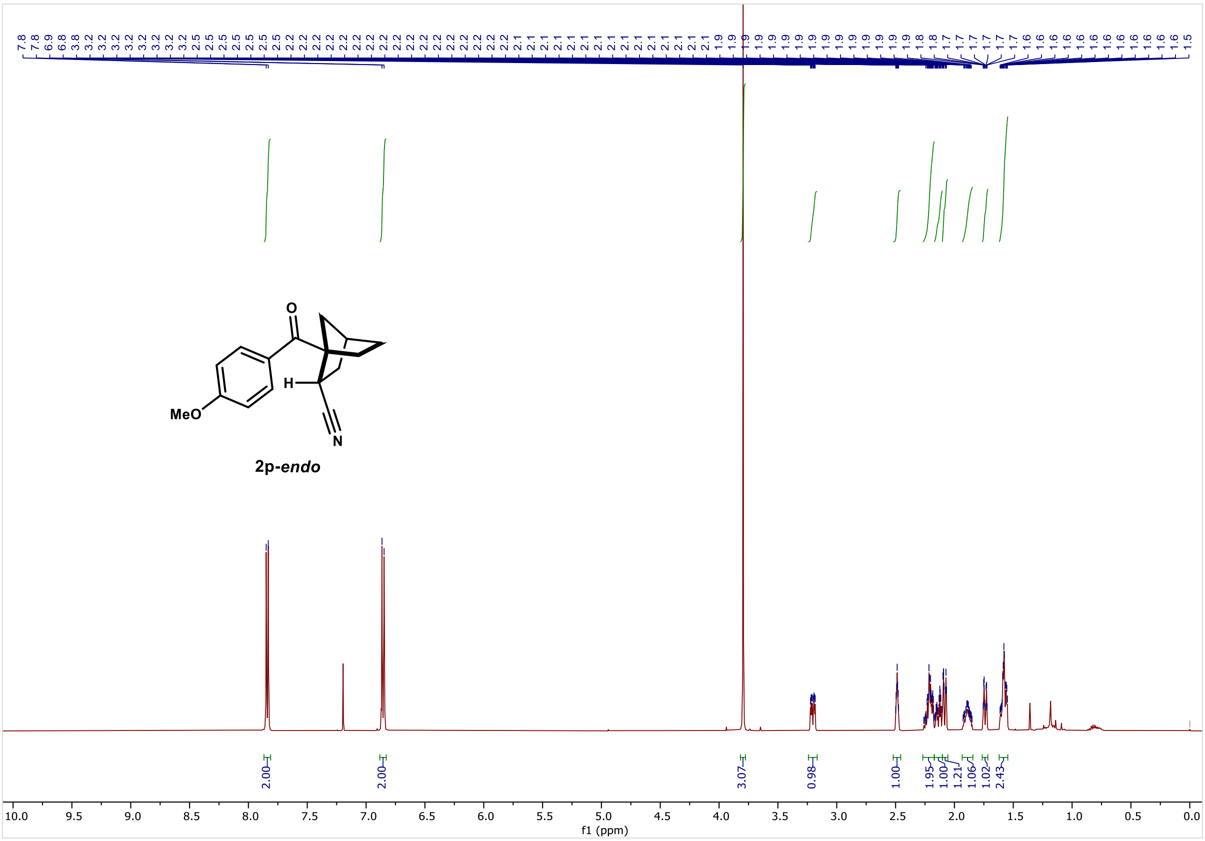

^{13}C NMR (126 MHz, Chloroform-*d*) of **2p-*endo***:

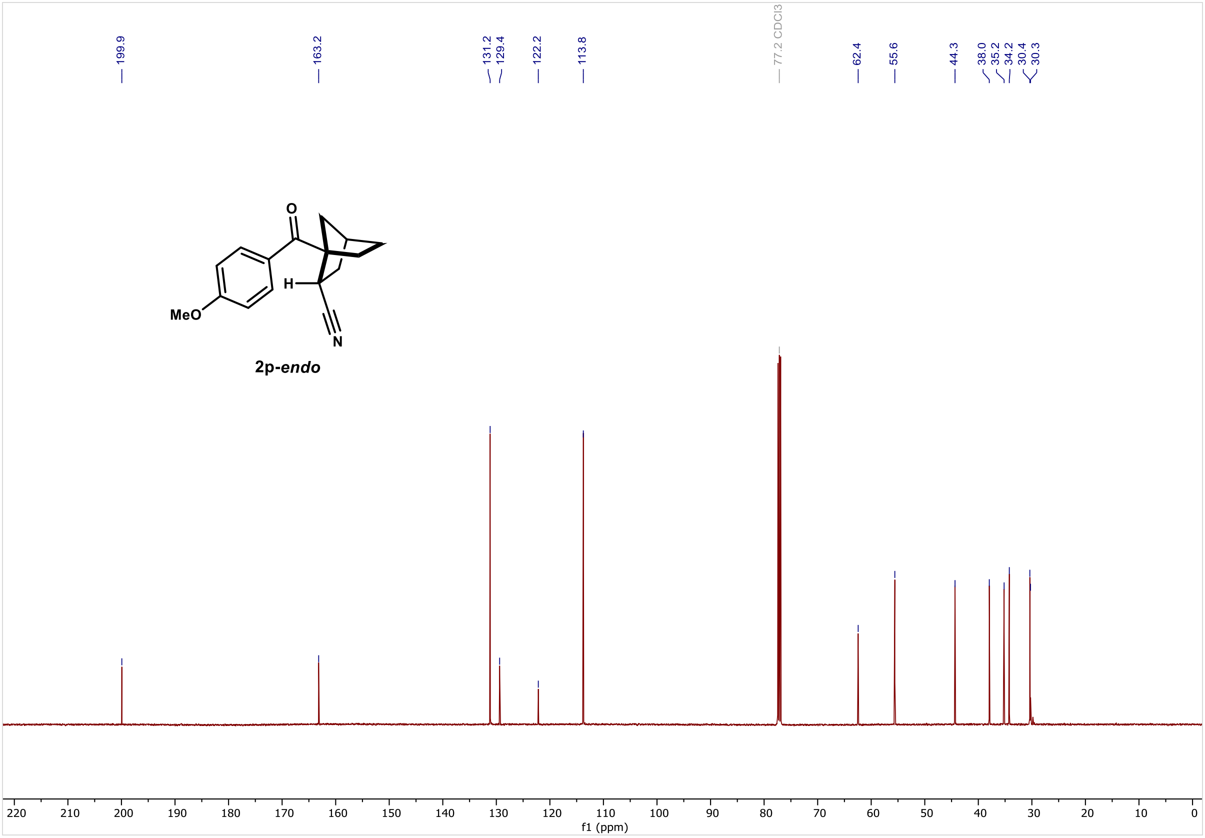

^{1}H NMR (500 MHz, Chloroform-*d*) of **2p-*exo***:

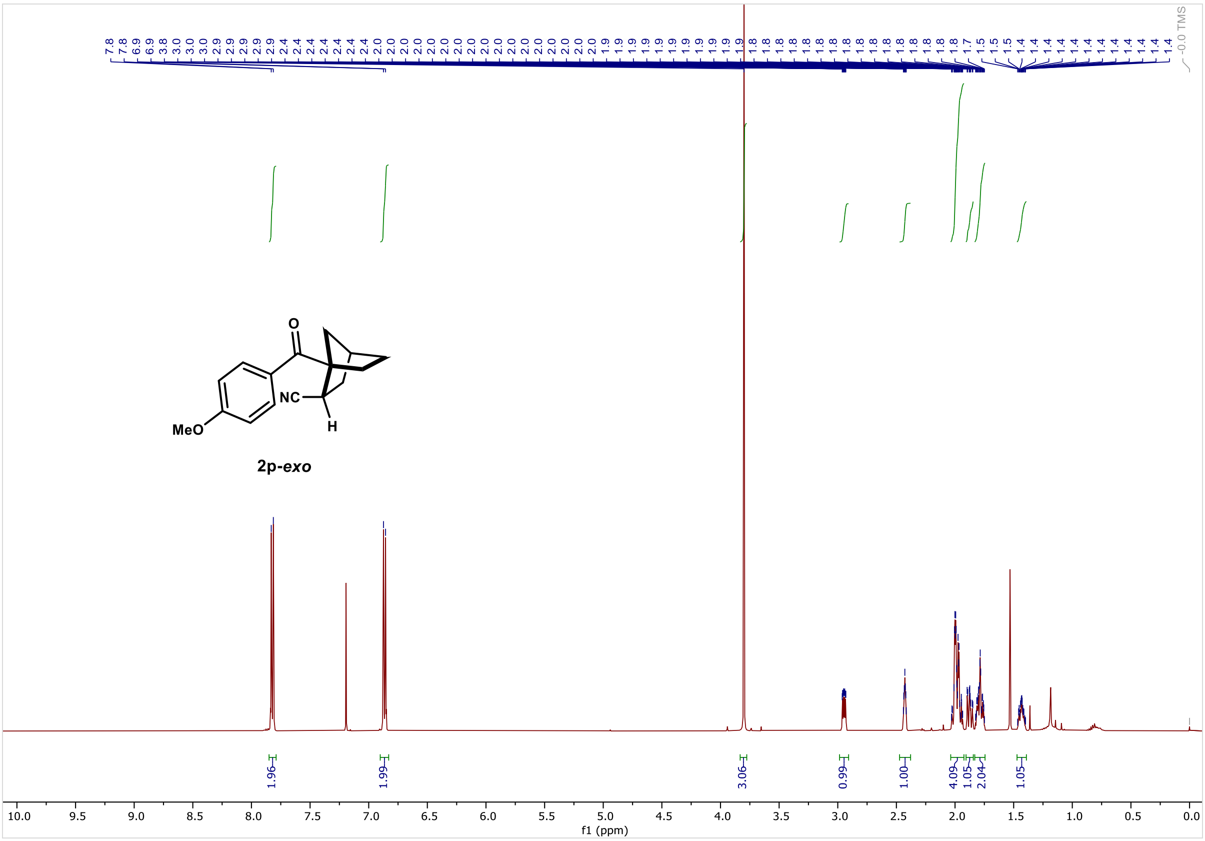

^{13}C NMR (126 MHz, Chloroform-*d*) of **2p-*exo***:

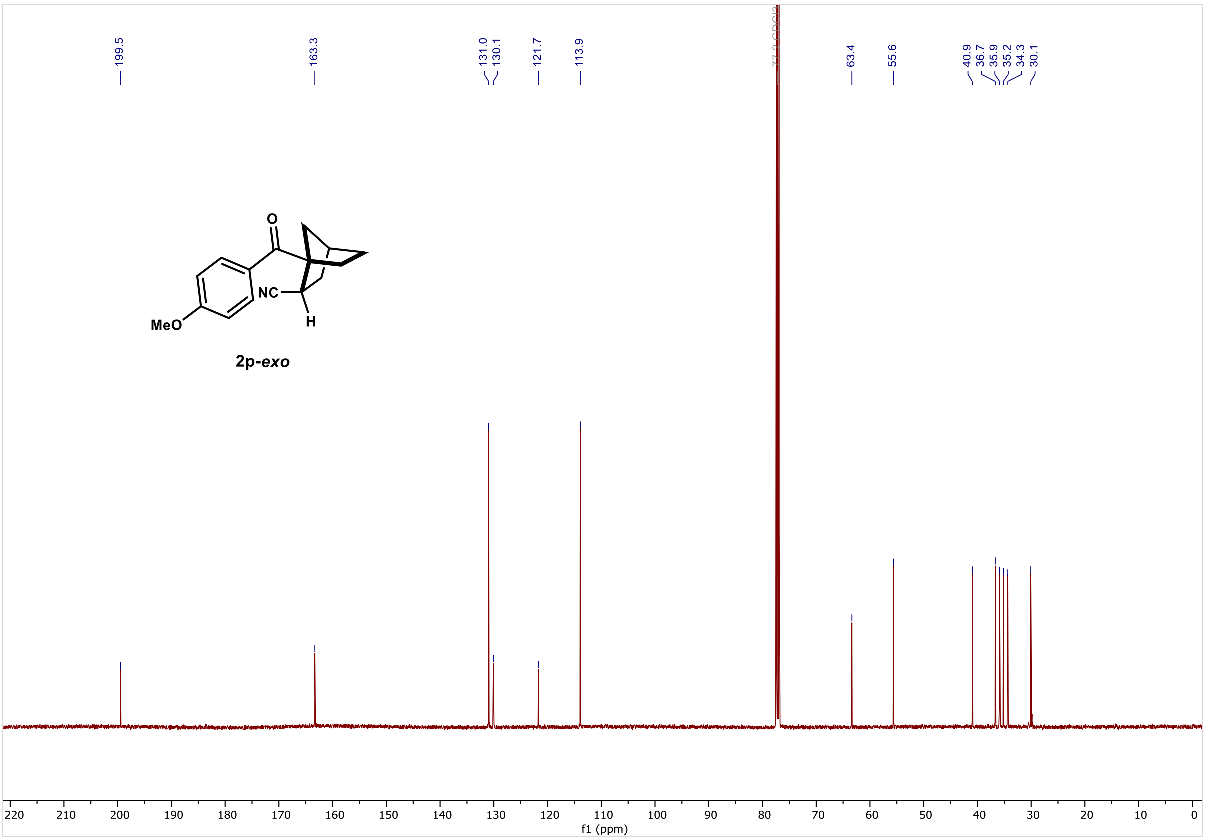

^{1}H NMR (500 MHz, Chloroform-*d*) of **2q-*endo***:

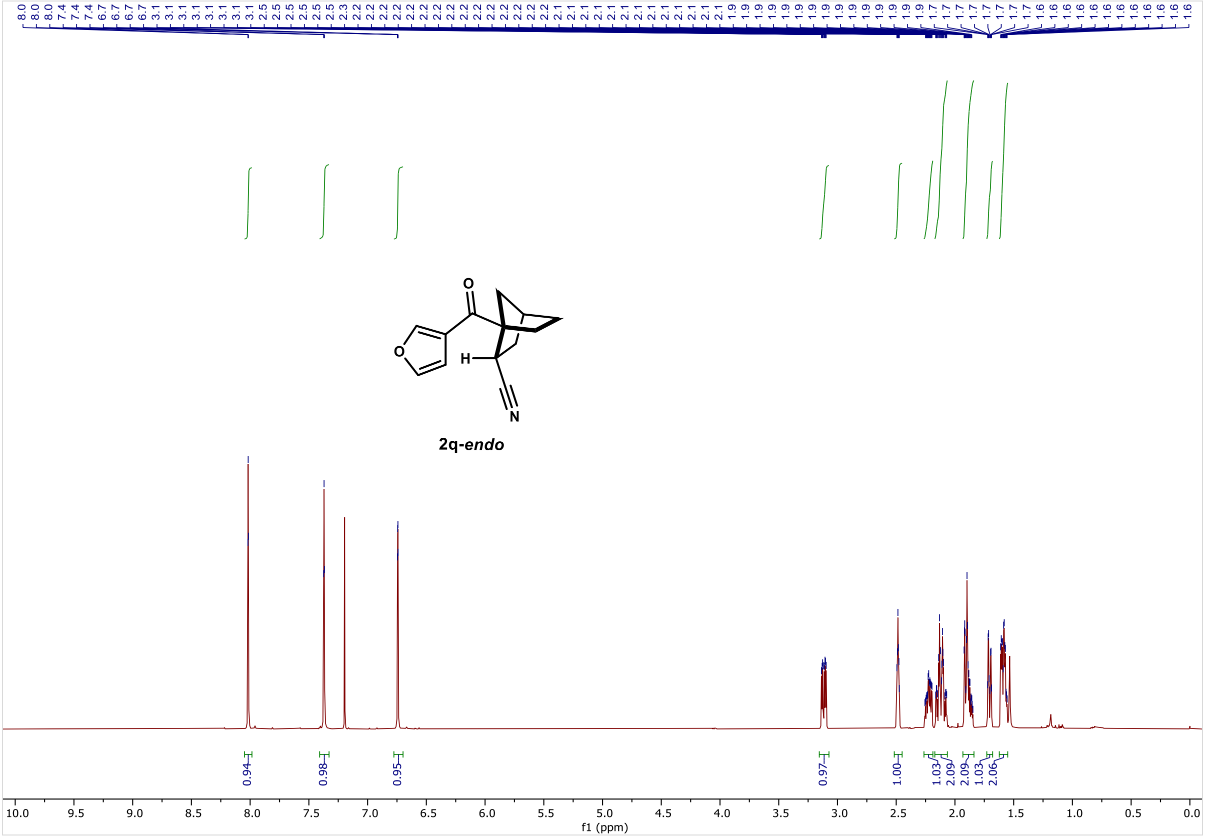

^{13}C NMR (126 MHz, Chloroform-*d*) of **2q-*endo***:

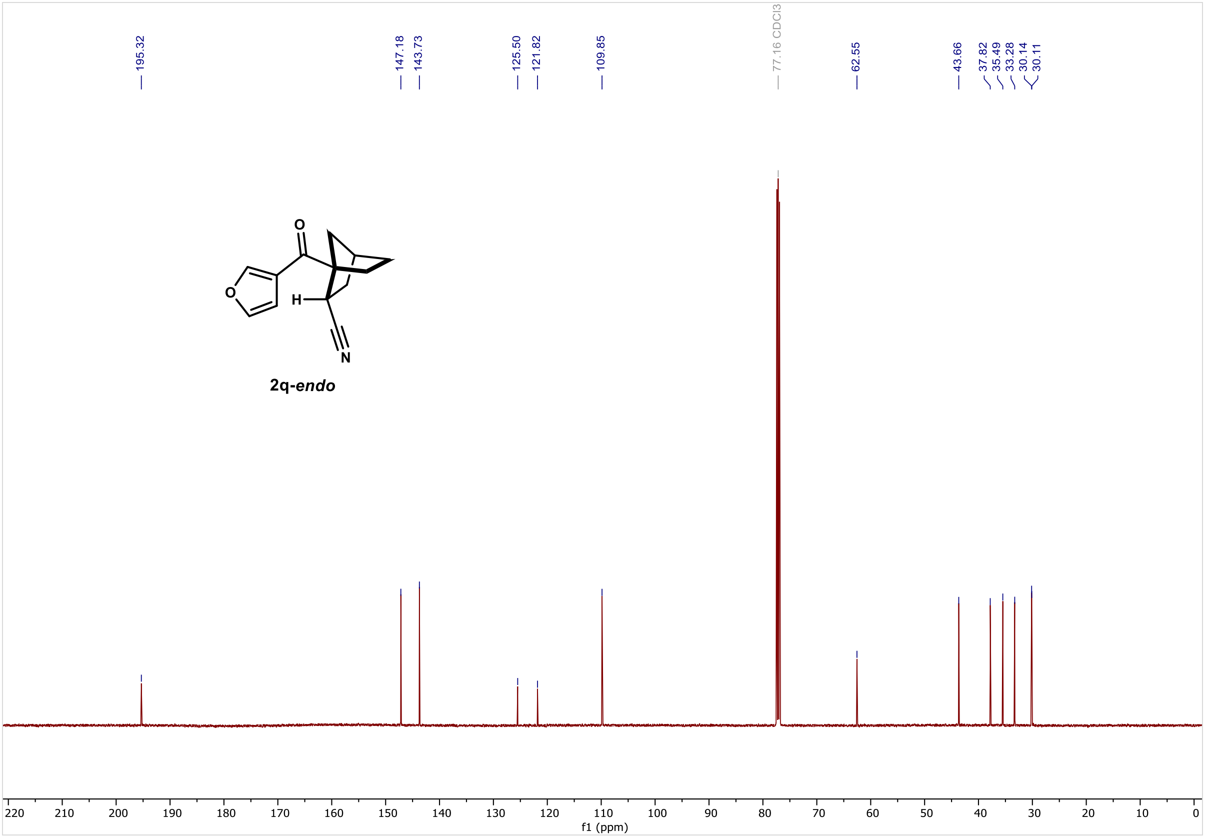

^{1}H NMR (500 MHz, Chloroform-*d*) of **2q-*exo***:

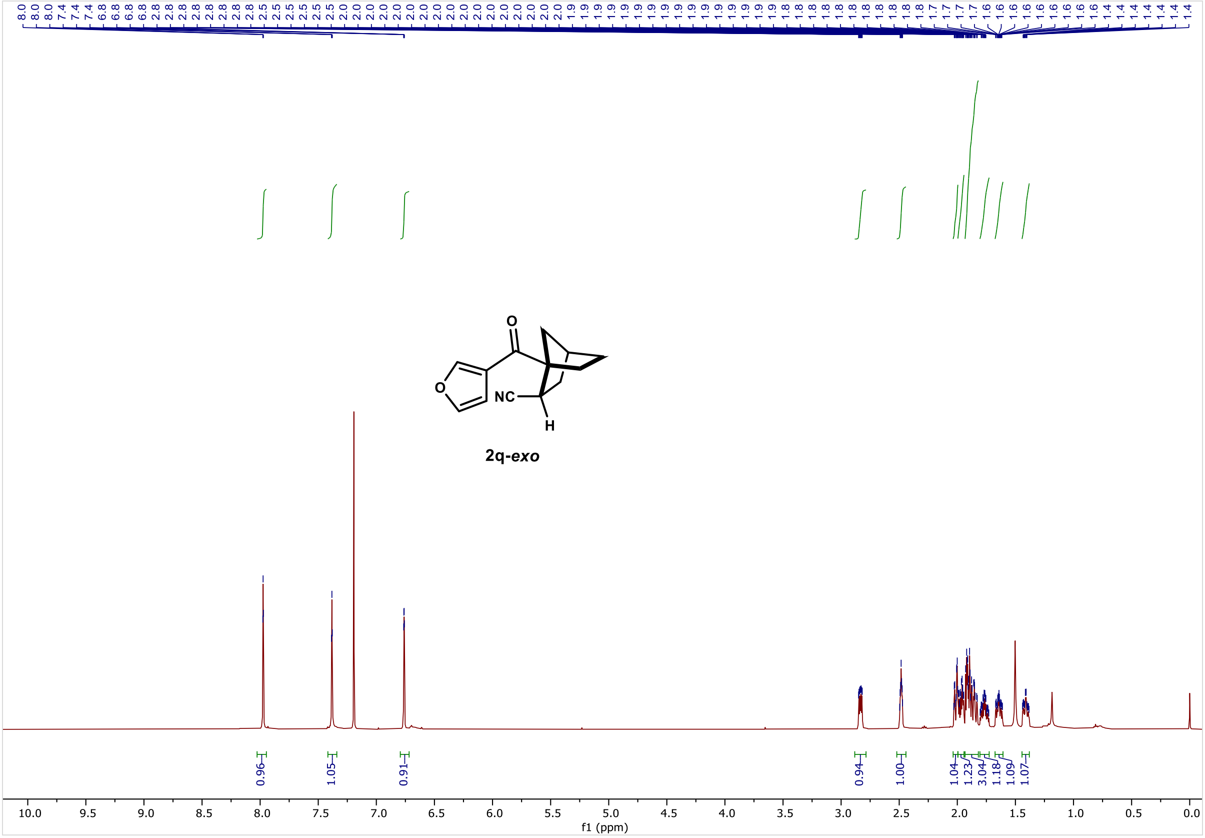

^{13}C NMR (126 MHz, Chloroform-*d*) of **2q-*exo***:

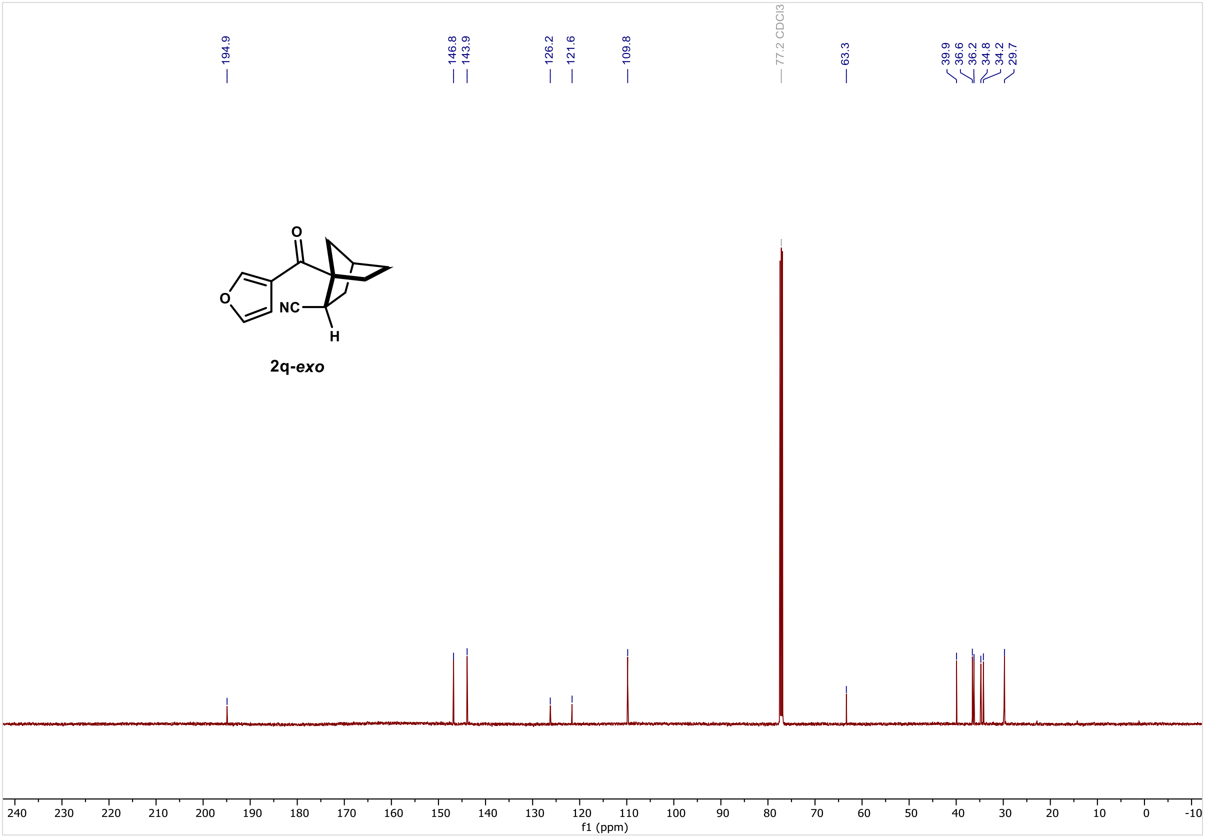

^{1}H NMR (400 MHz, Chloroform-*d*) of **2r-*endo***:

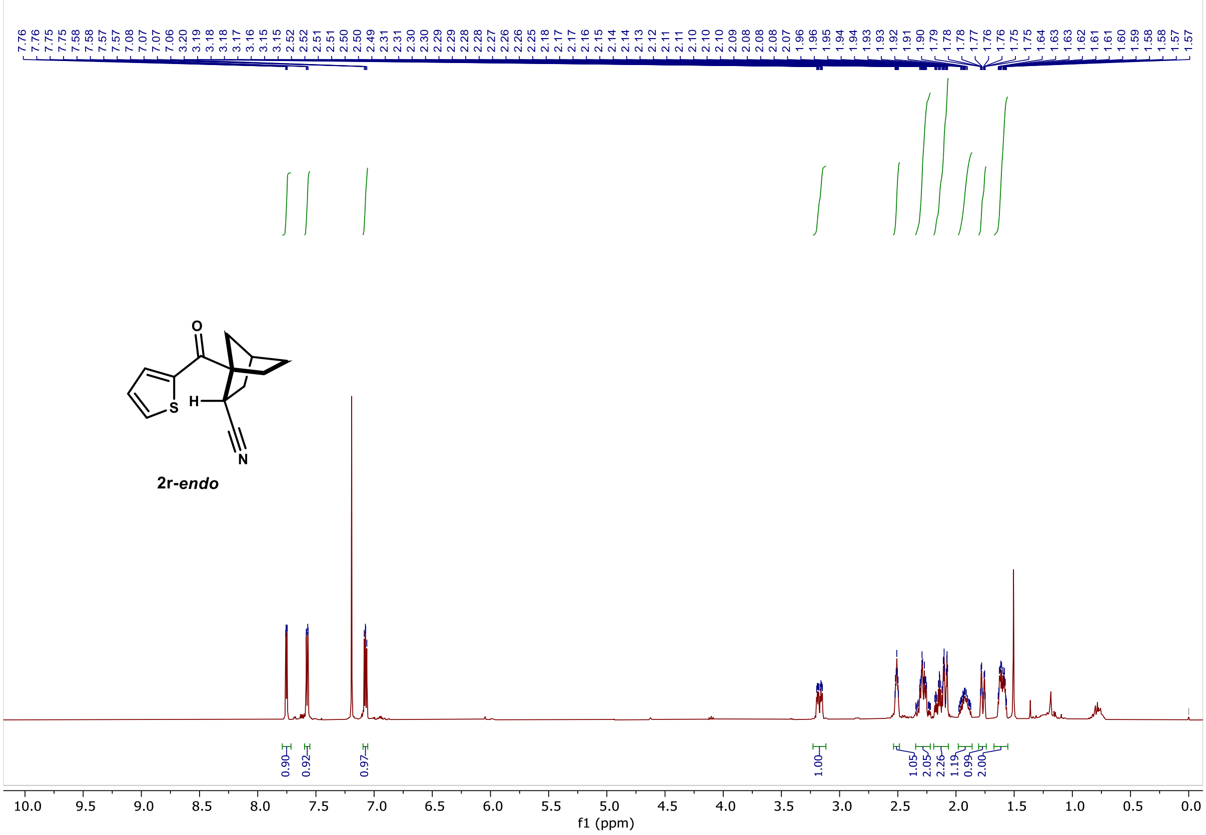

^{13}C NMR (101 MHz, Chloroform-*d*) of **2r-*endo***:

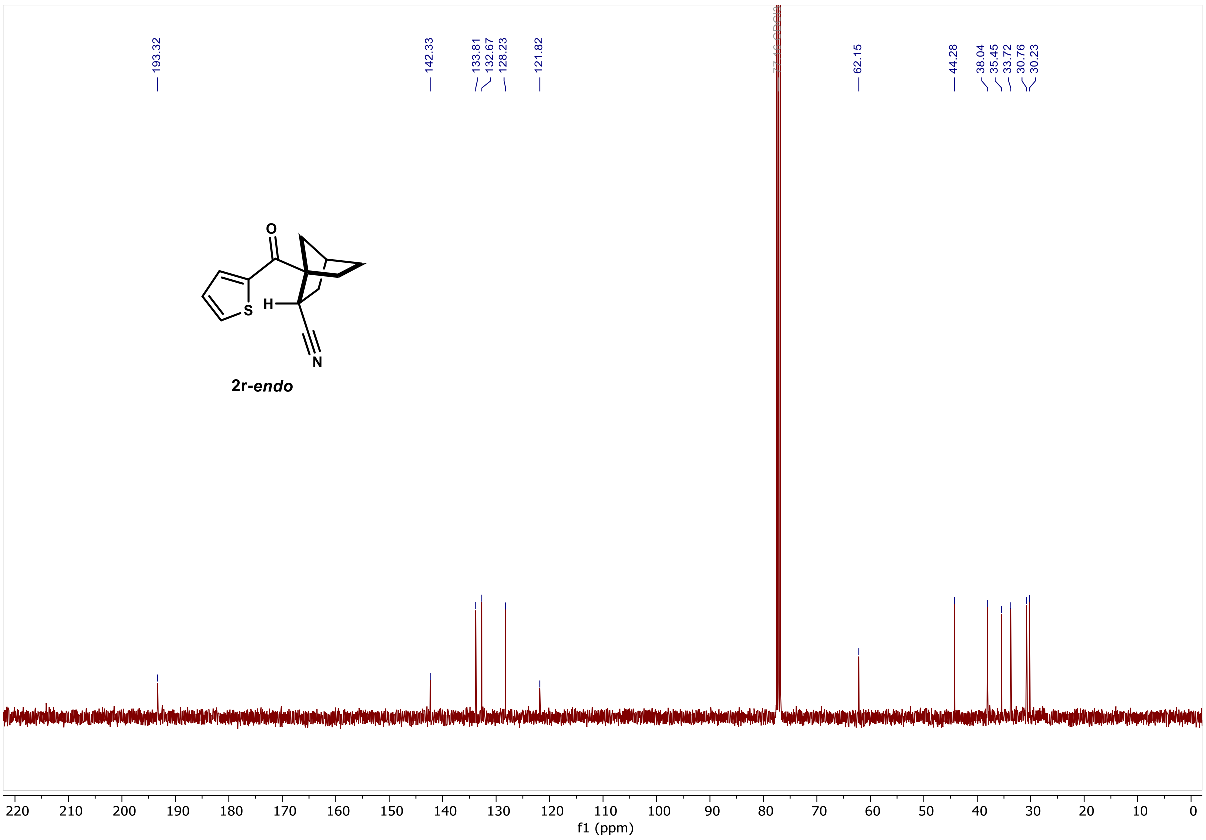

^{1}H NMR (400 MHz, Chloroform-*d*) of **2r-*exo***:

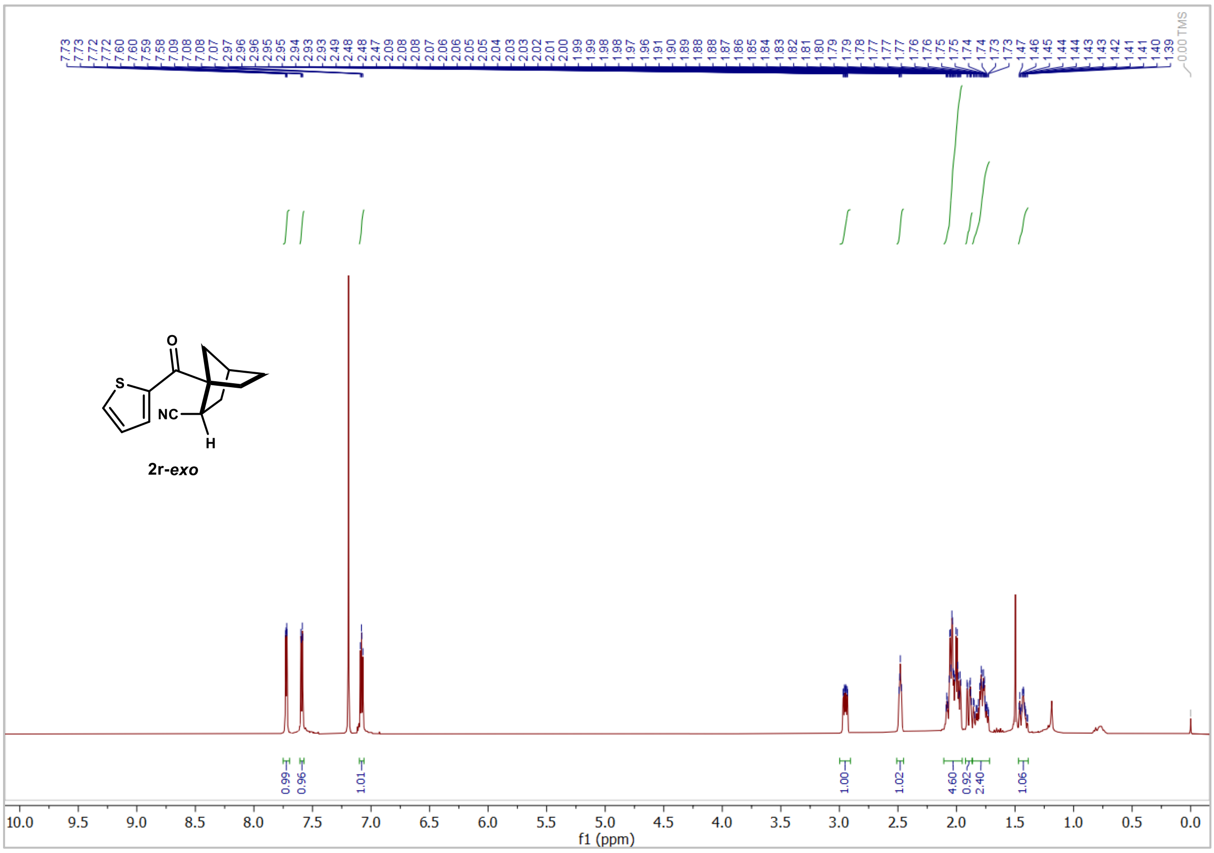

^{13}C NMR (101 MHz, Chloroform-*d*) of **2r-*exo***:

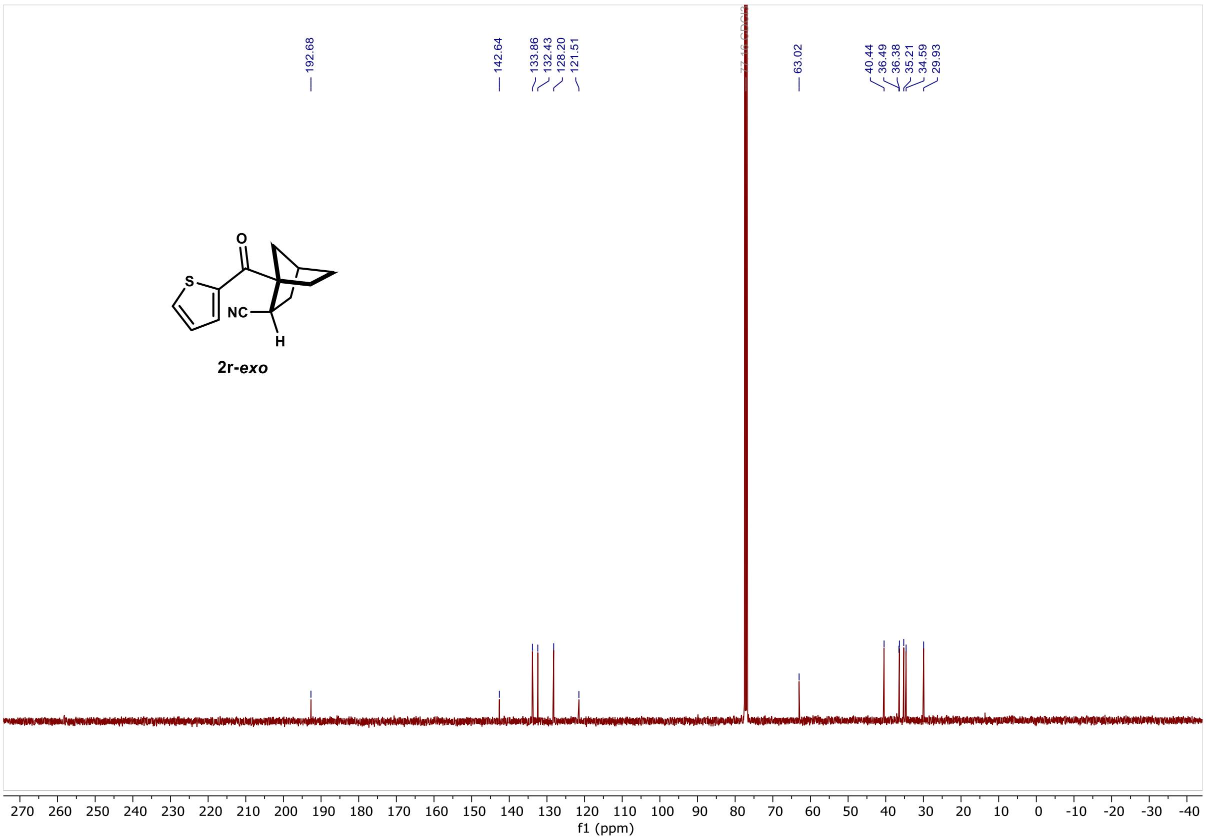

^{1}H NMR (500 MHz, Chloroform-*d*) of **2s-*endo* and 2s-*exo***:

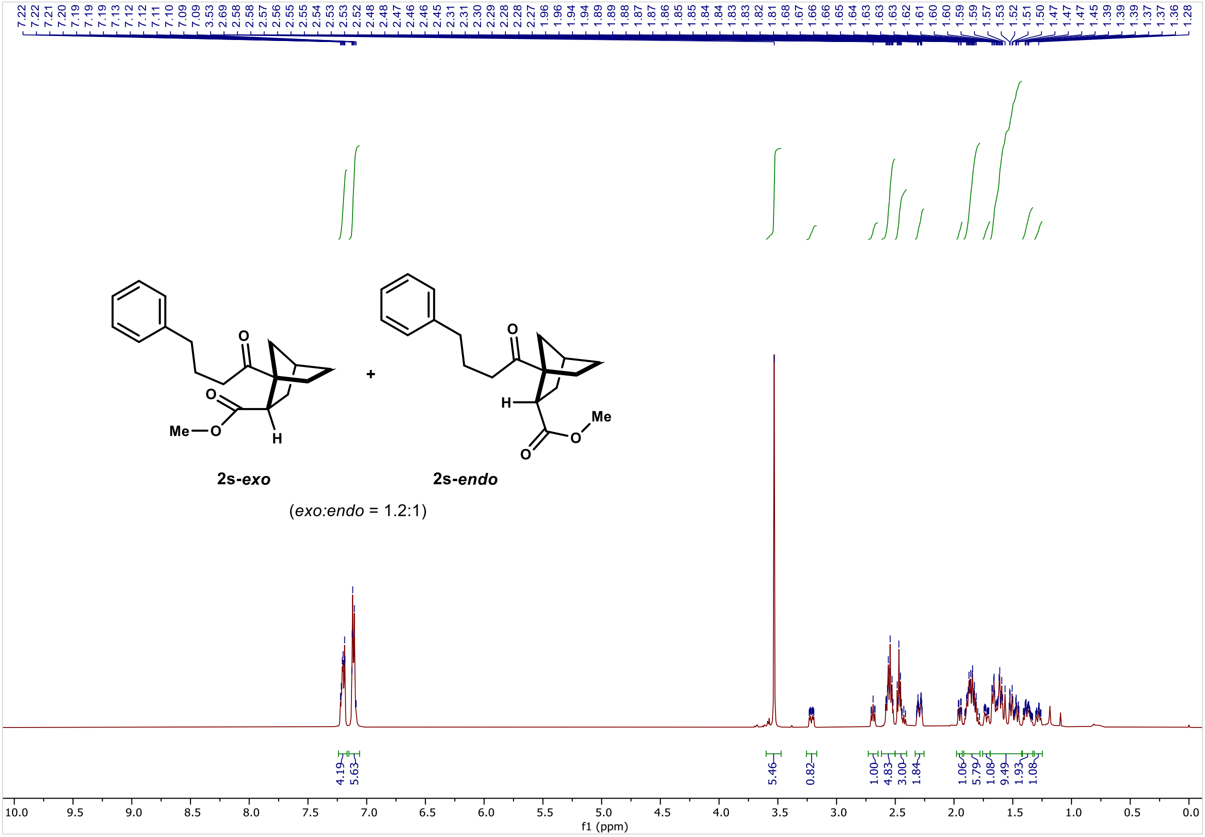

^{13}C NMR (101 MHz, Chloroform-*d*) of **2s-*endo* and 2s-*exo***:

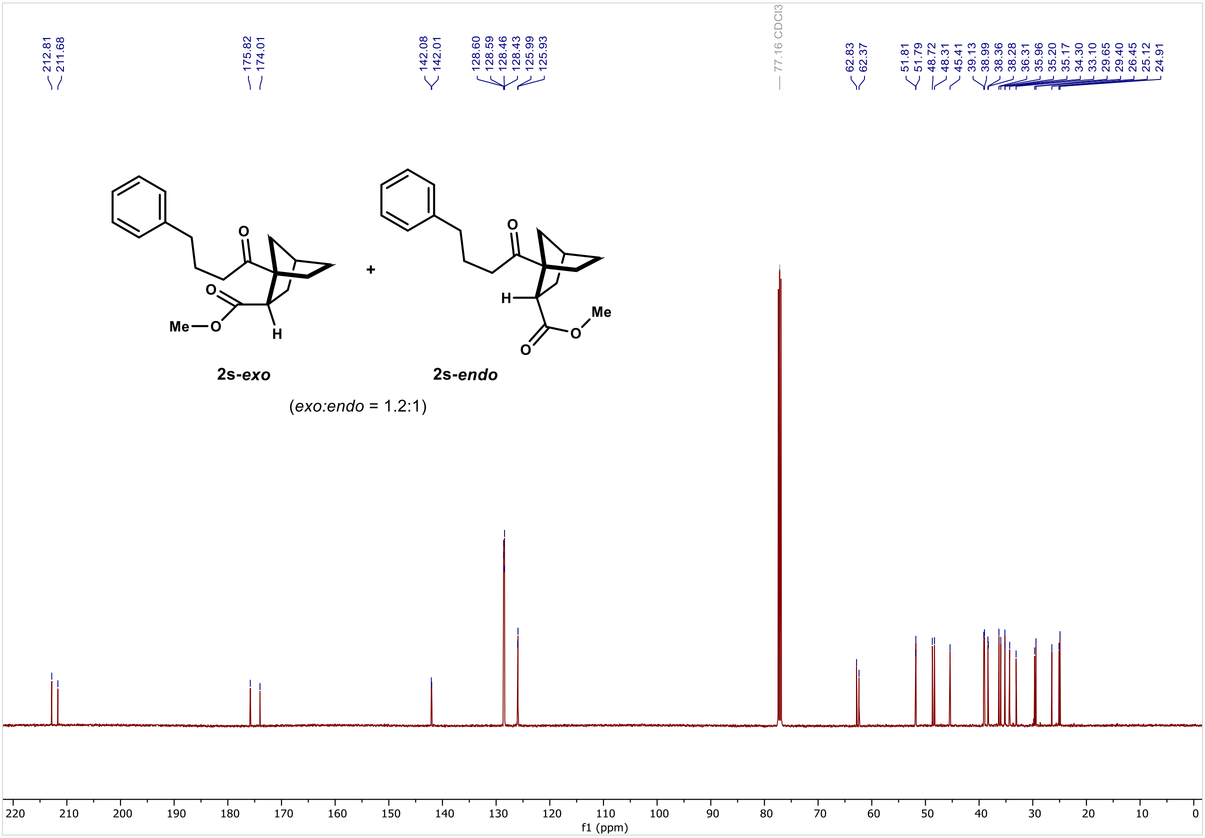

^{1}H NMR (400 MHz, Chloroform-*d*) of **2t-*endo* and 2t-*exo***:

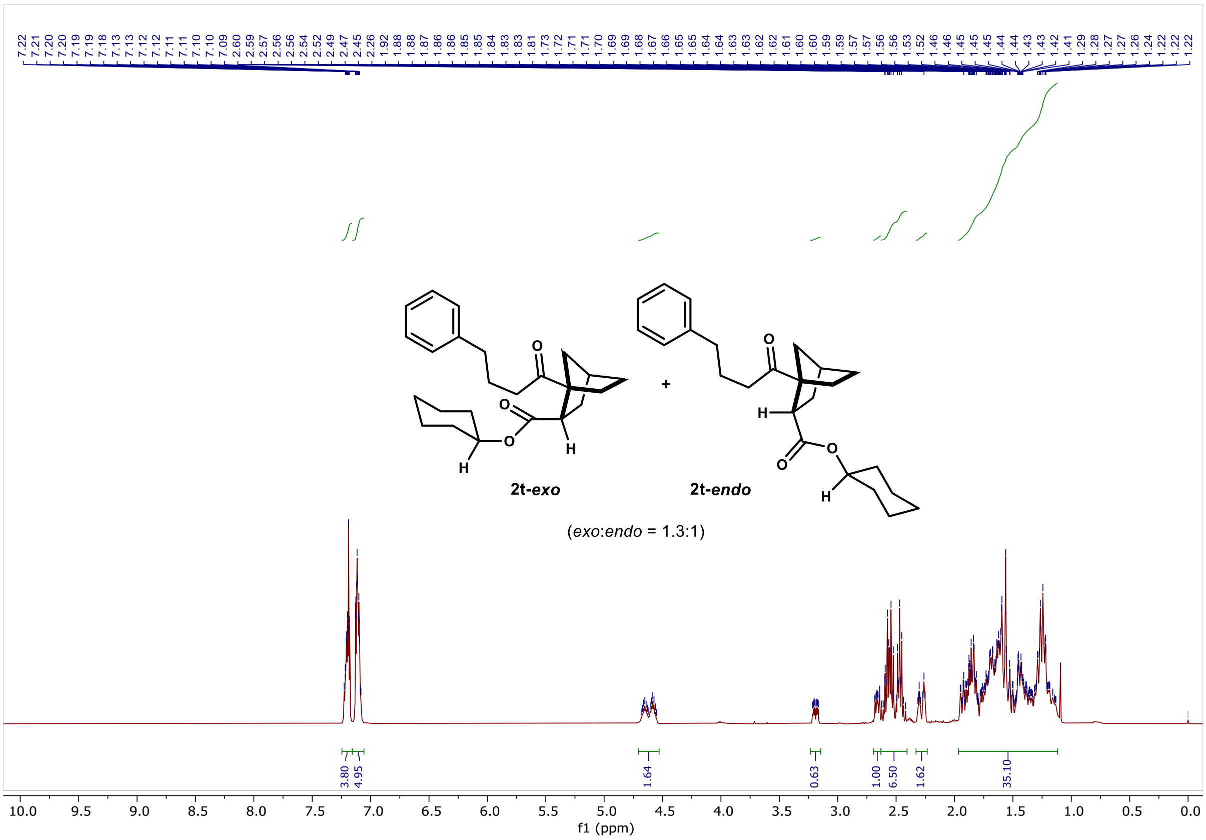

^{13}C NMR (101 MHz, Chloroform-*d*) of **2t-*endo* and 2t-*exo***:

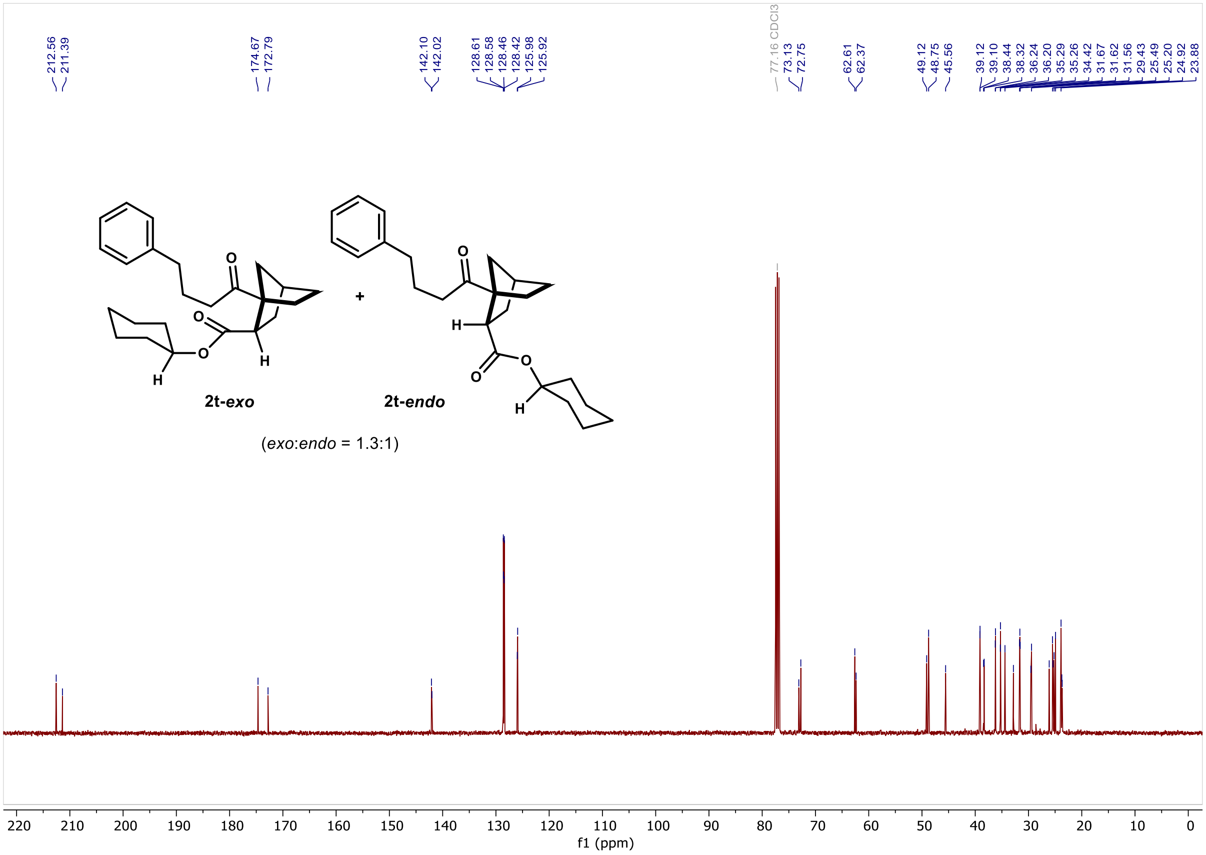

^{1}H NMR (400 MHz, Chloroform-*d*) of **2u-*endo* and 2u-*exo***:

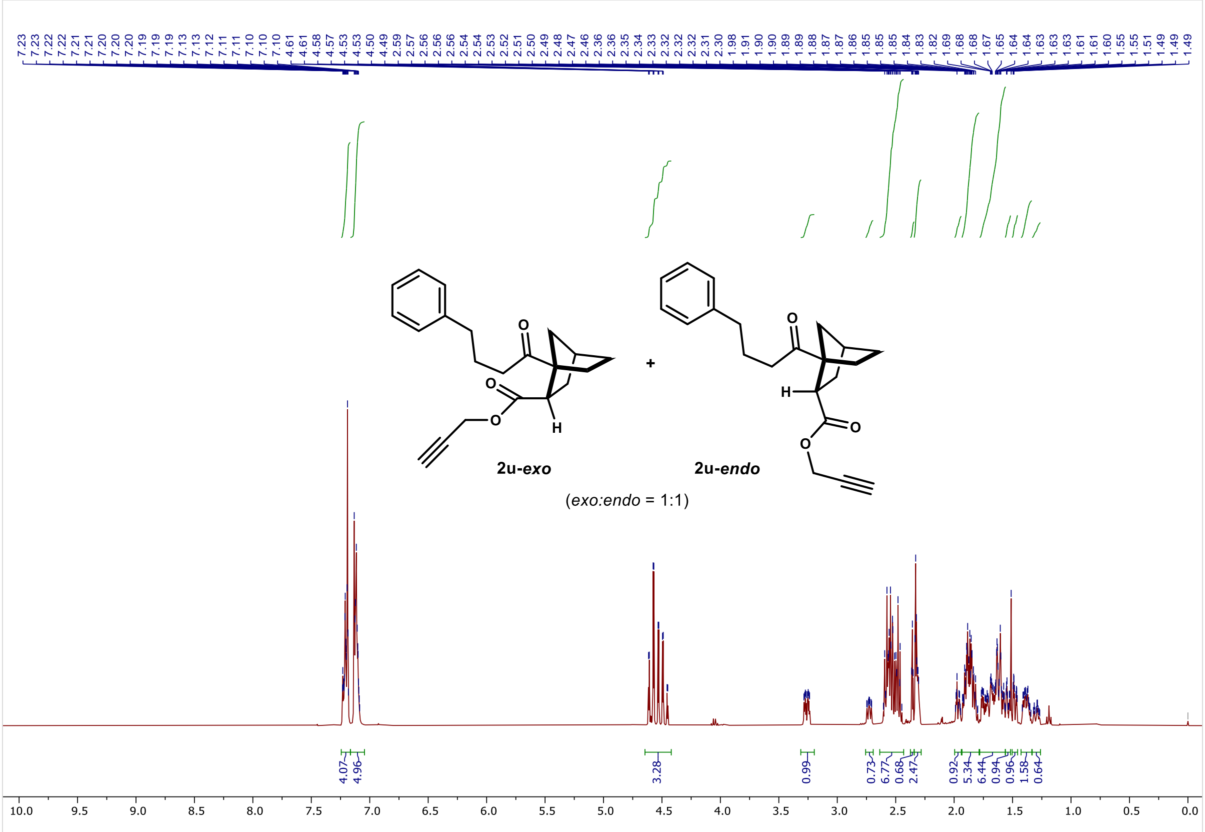

^{13}C NMR (126 MHz, Chloroform-*d*) of **2u-*endo* and 2u-*exo***:

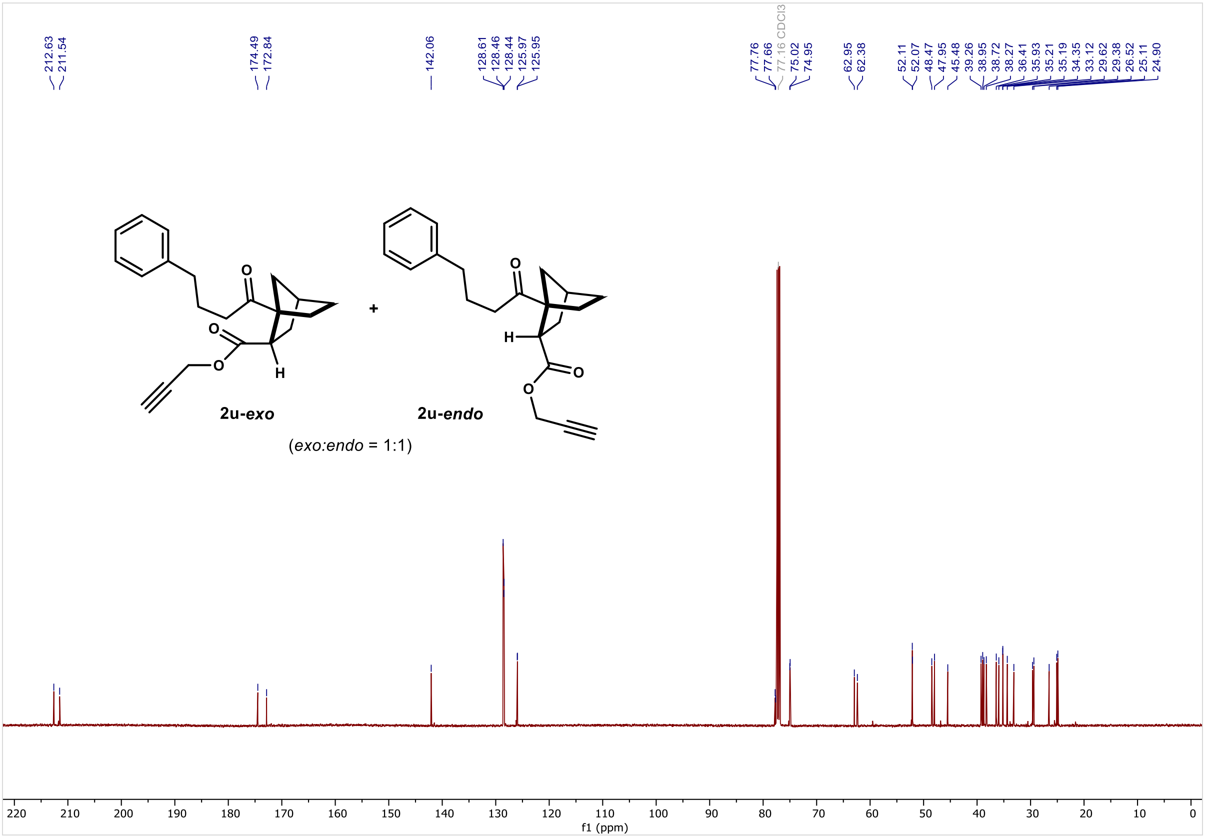

^{1}H NMR (500 MHz, Chloroform-*d*) of **2v-*endo* and 2v-*exo***:

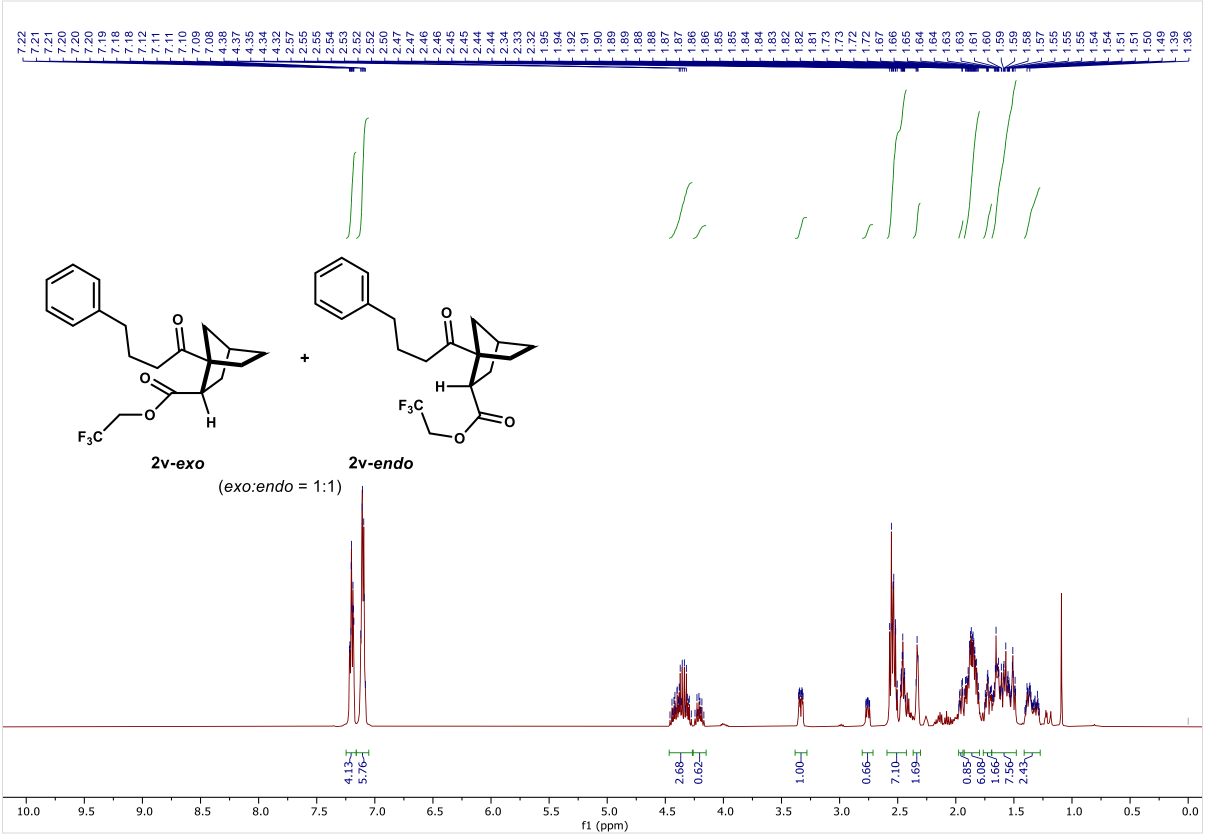

^{13}C NMR (126 MHz, Chloroform-*d*) of **2v-*endo* and 2v-*exo***:

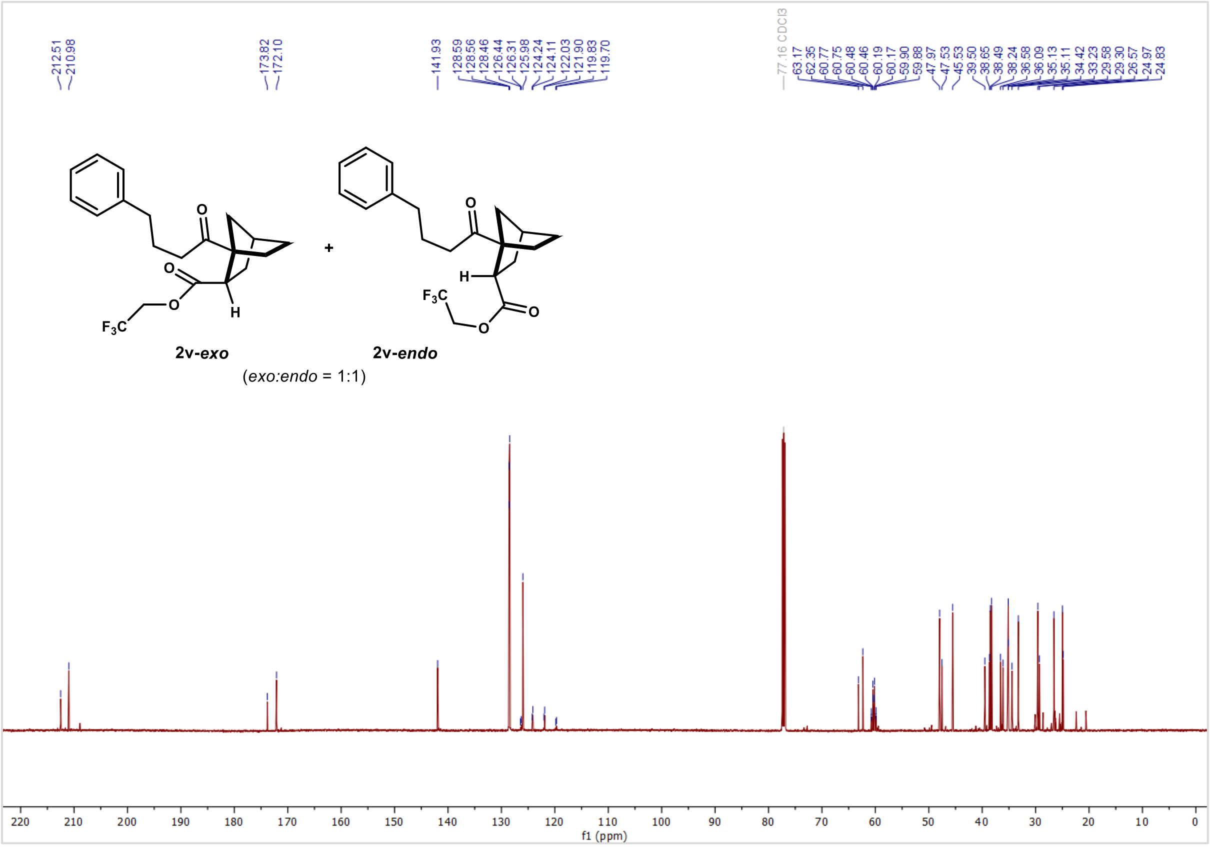

^{19}F NMR (376 MHz, Chloroform-*d*) of **2v-*endo* and 2v-*exo***:

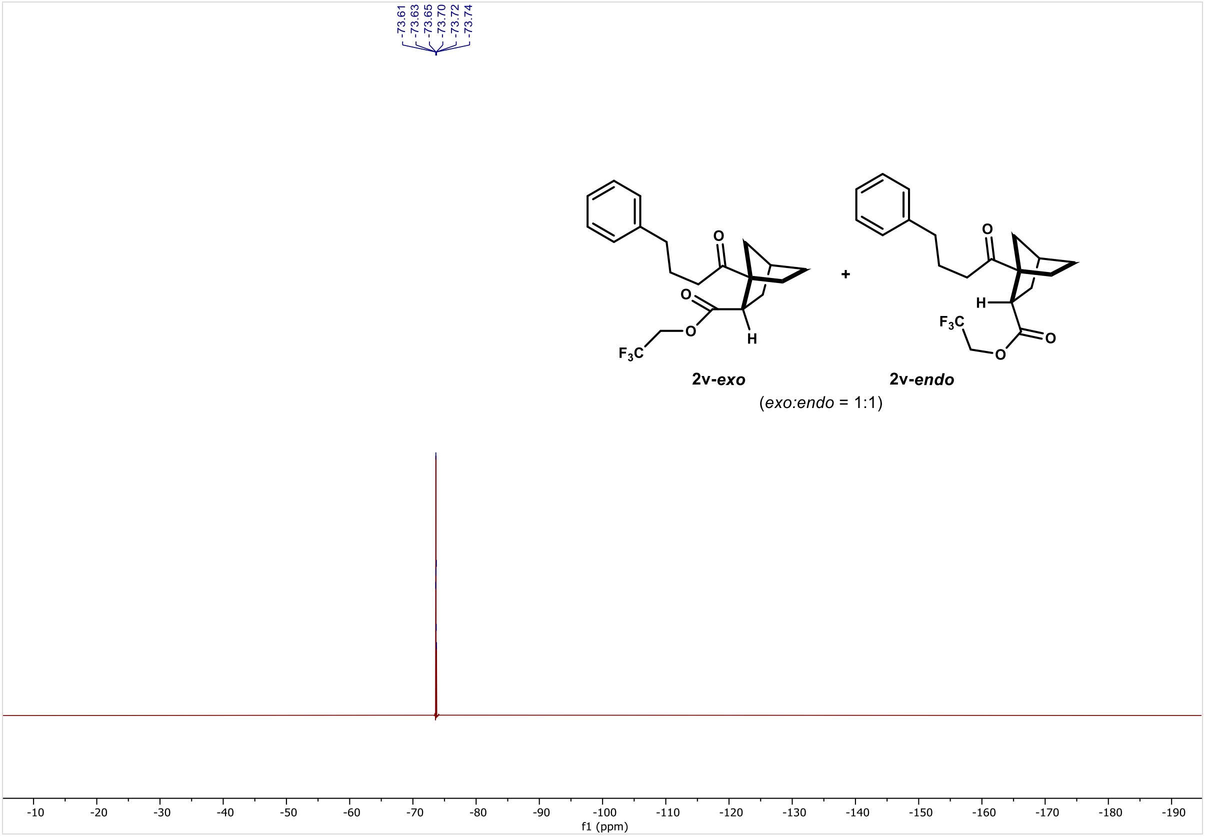

^{1}H NMR (400 MHz, Chloroform-*d*) of **2w-*endo***:

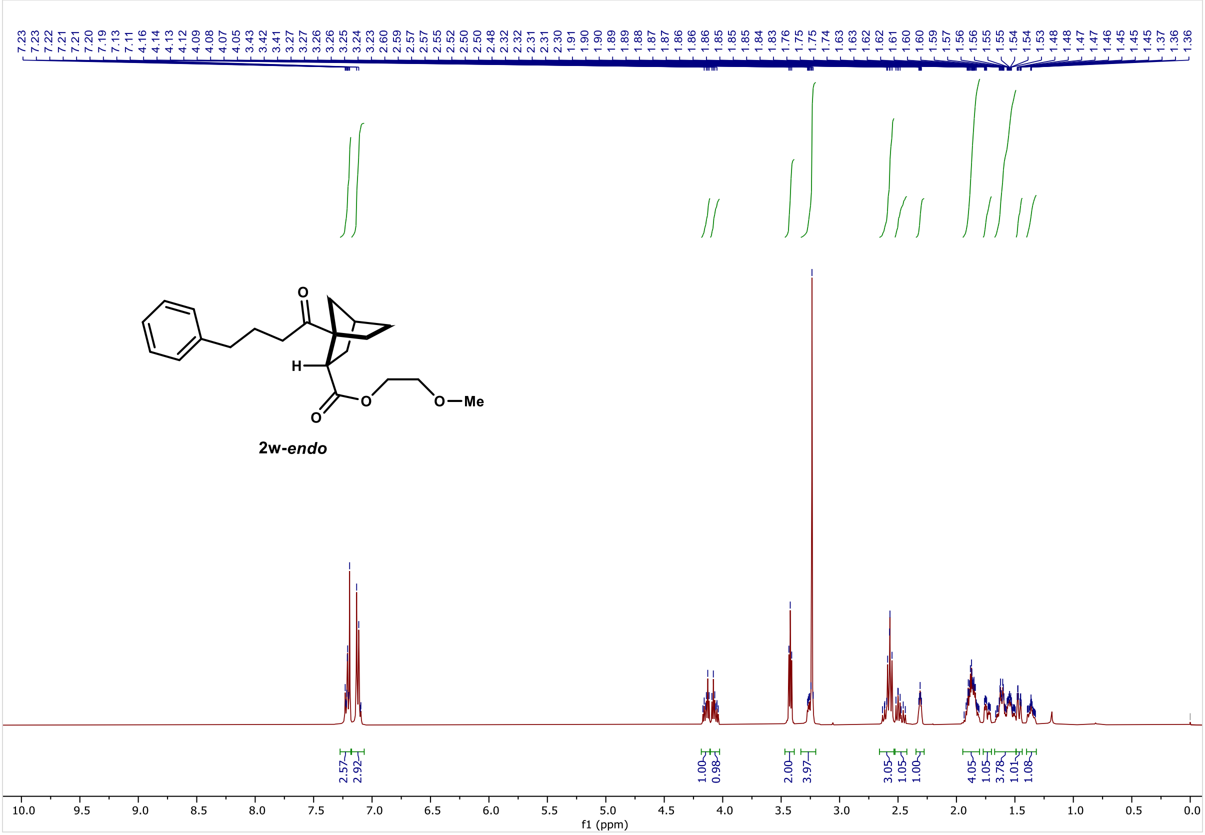

^{13}C NMR (126 MHz, Chloroform-*d*) of **2w-*endo***:

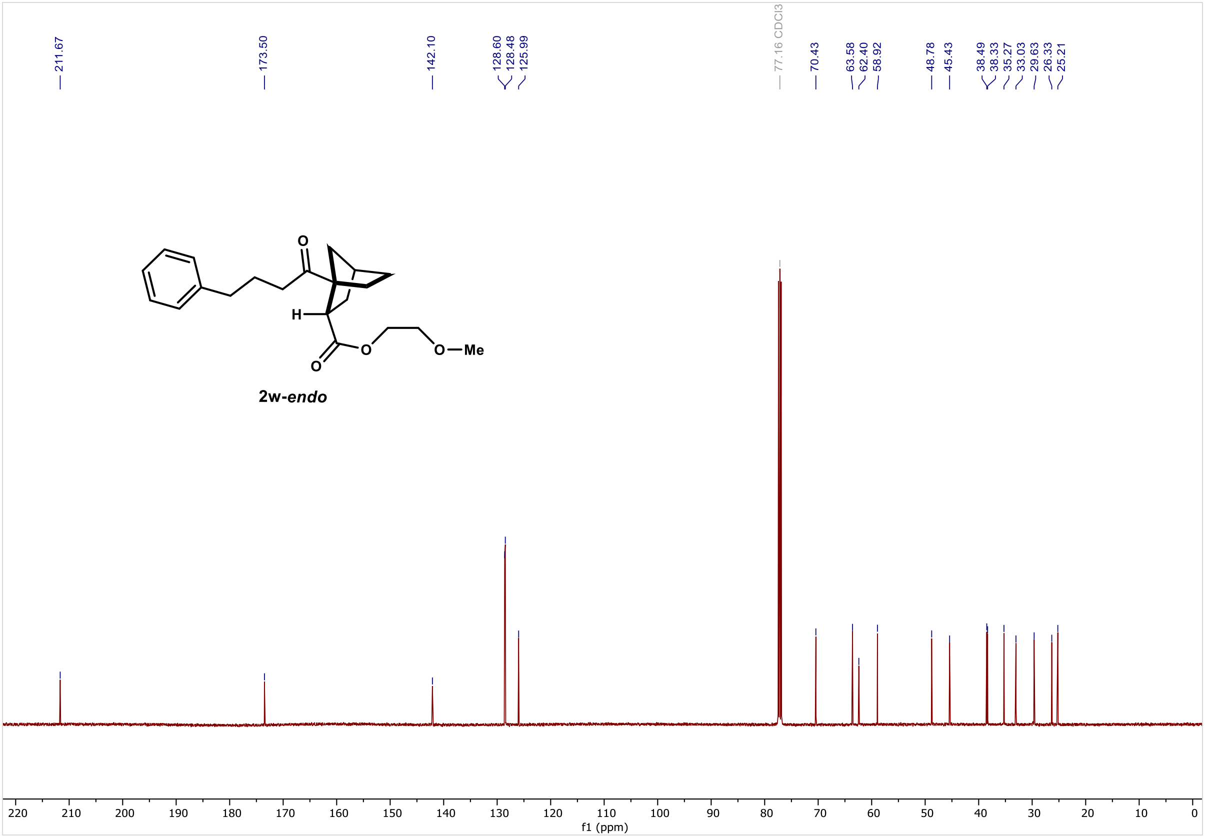

^{1}H NMR (400 MHz, Chloroform-*d*) of **2w-*exo***:

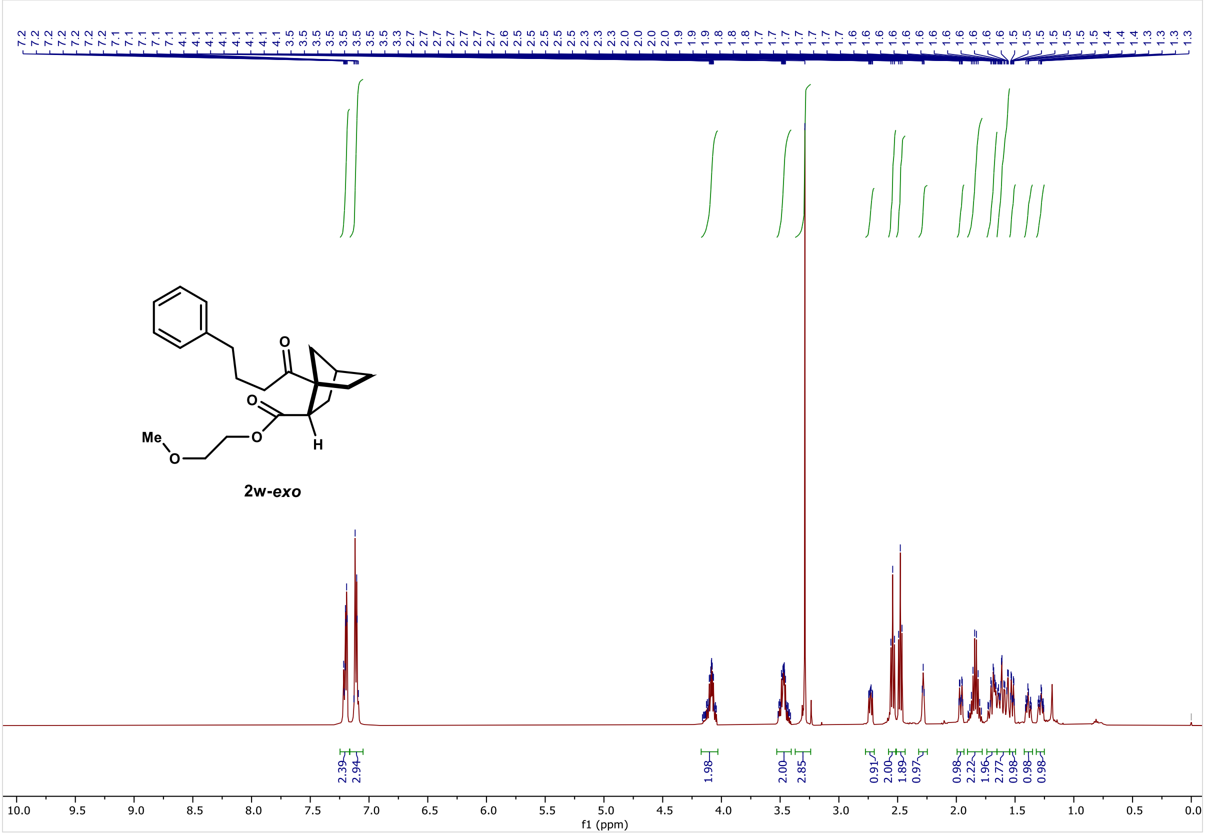

^{13}C NMR (126 MHz, Chloroform-*d*) of **2w-*exo***:

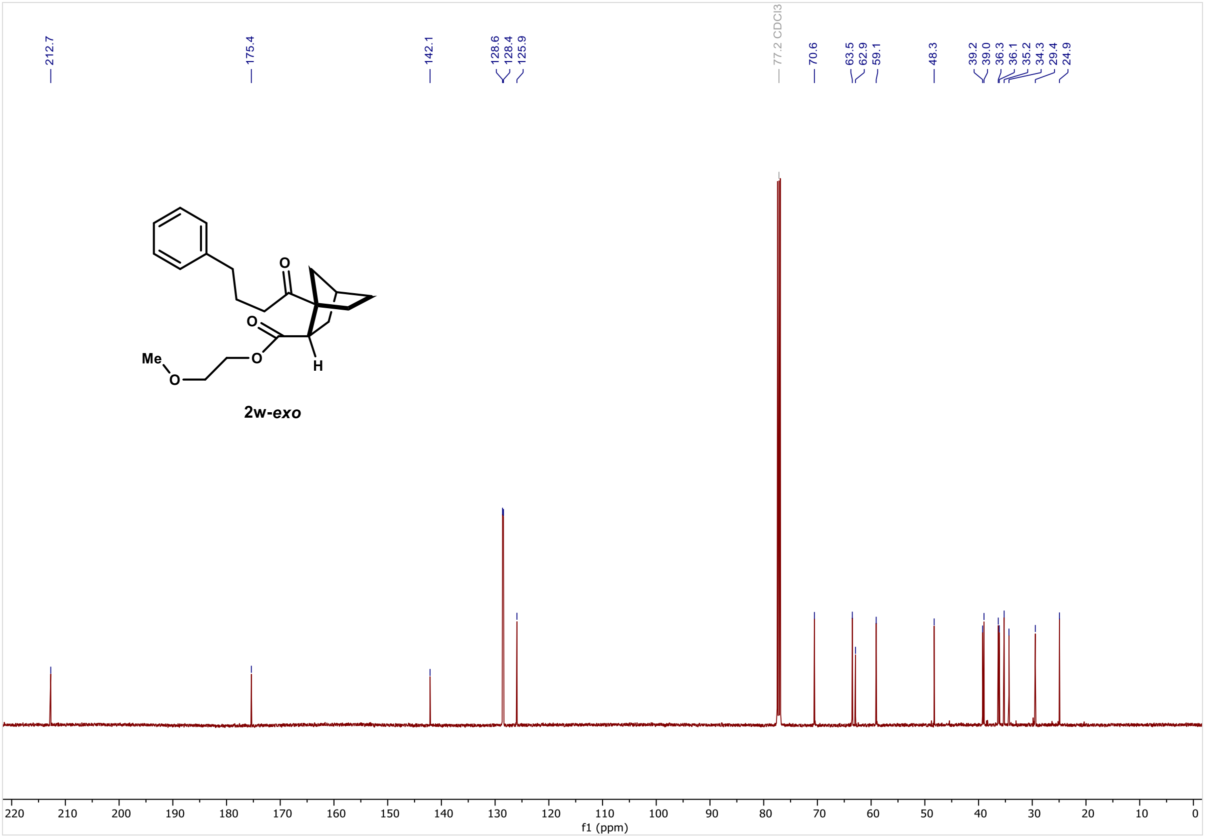

^{1}H NMR (400 MHz, Chloroform-*d*) of **2x-*endo* and 2x-*exo***:

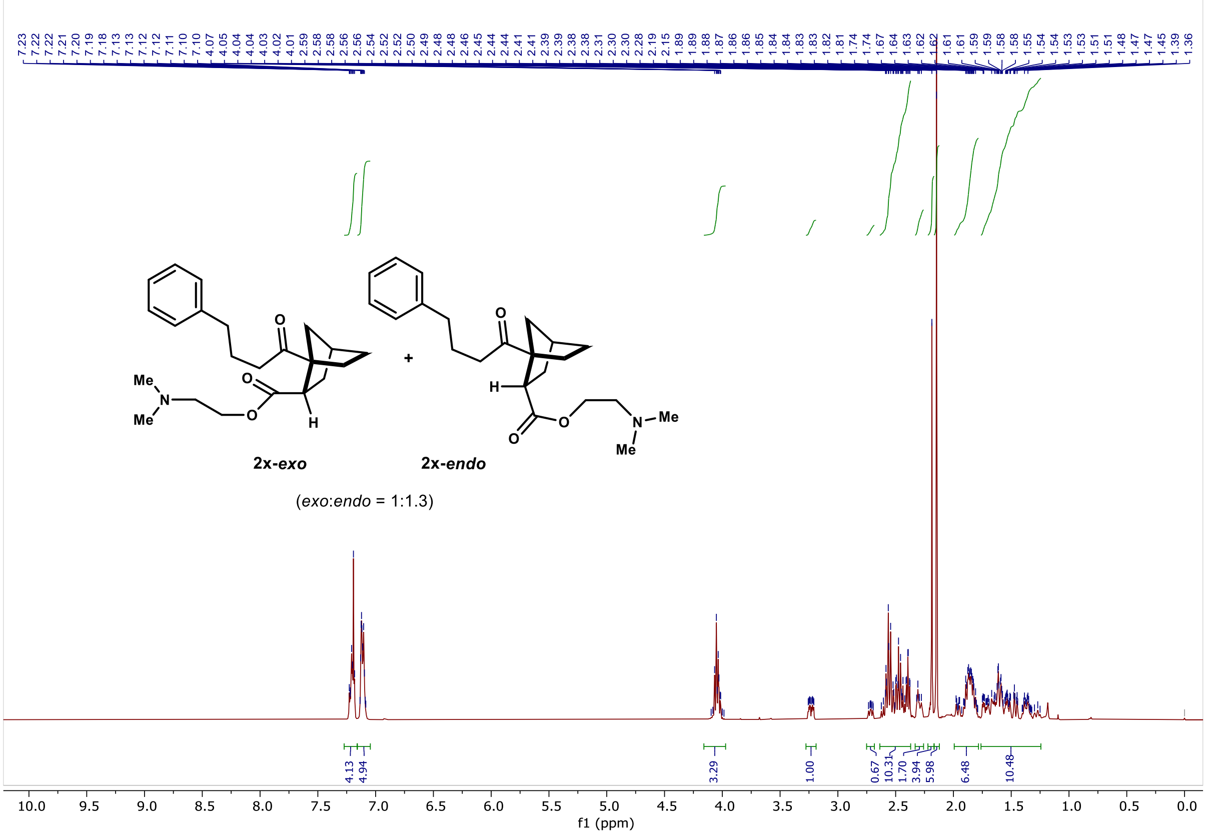

^{13}C NMR (101 MHz, Chloroform-*d*) of **2x-*endo* and 2x-*exo***:

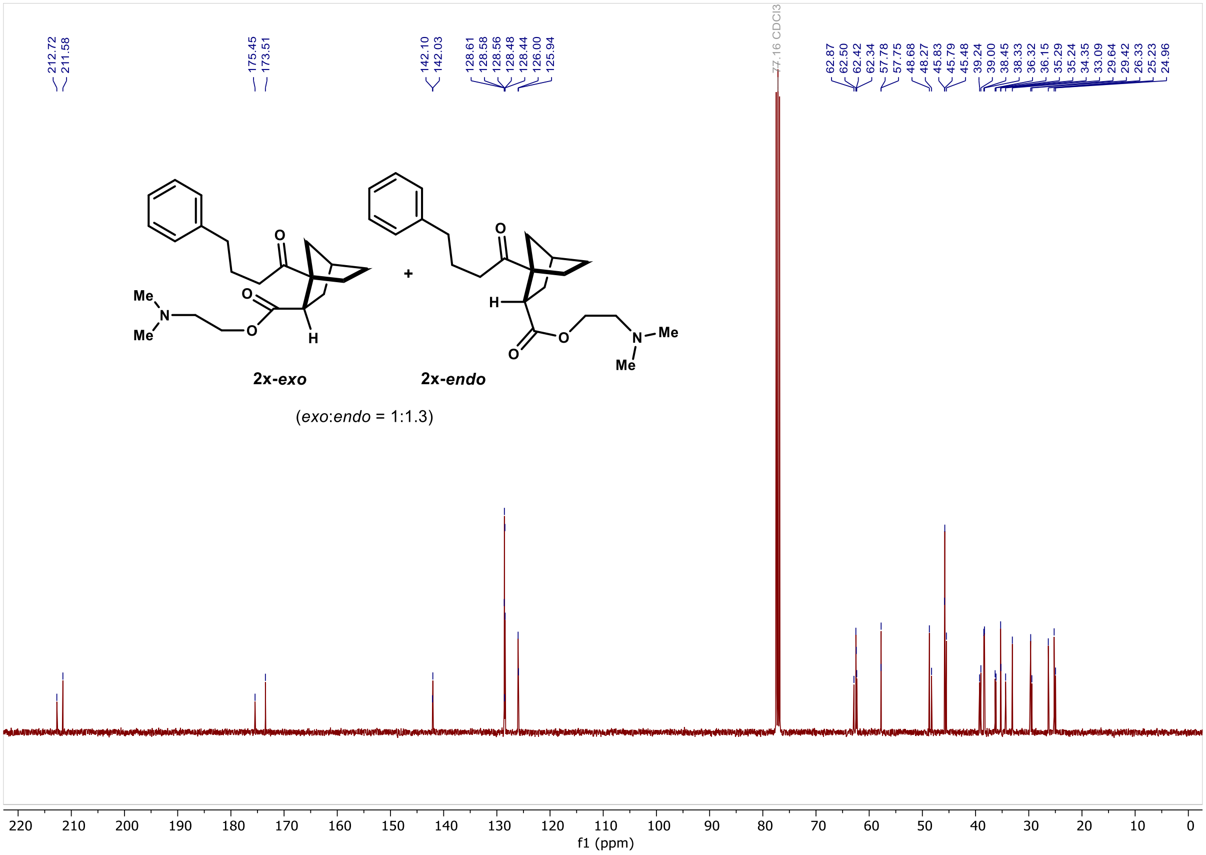

^{1}H NMR (400 MHz, Chloroform-*d*) of **2y-*endo* and 2y-*exo***:

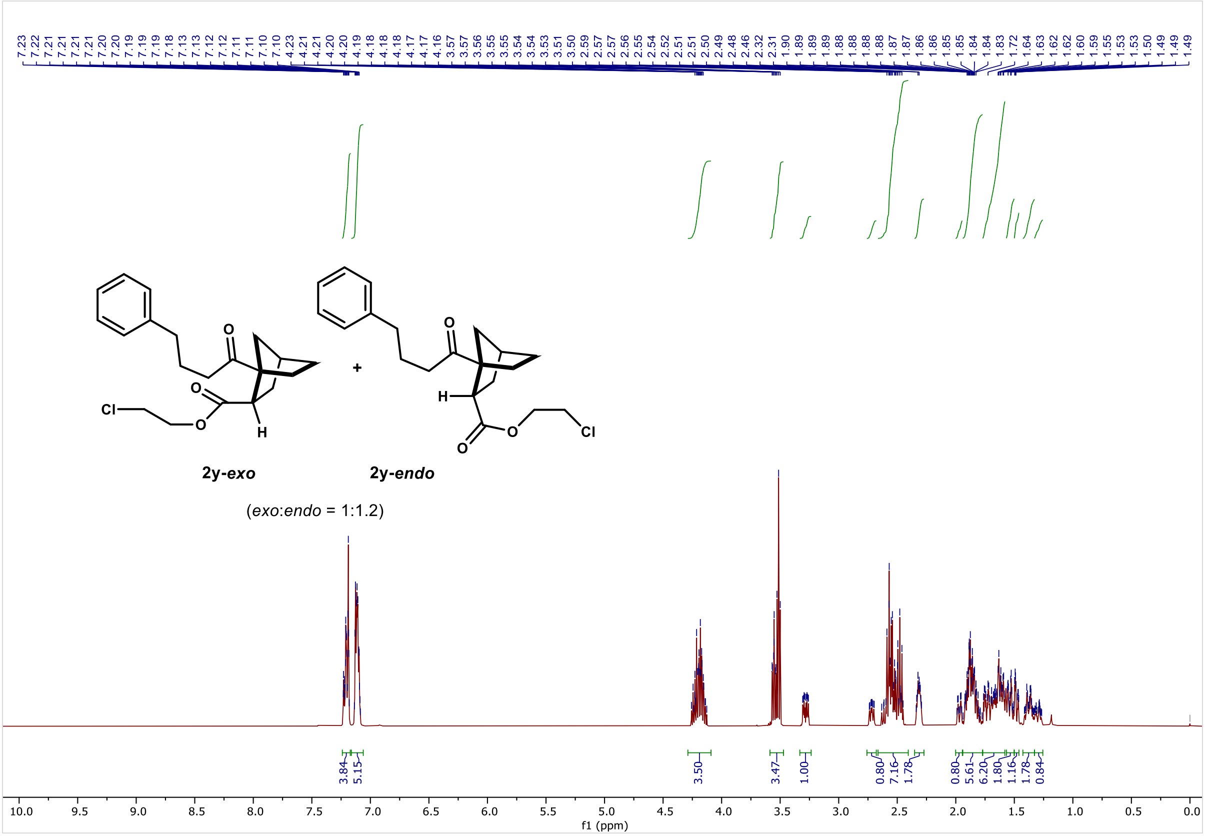

^{13}C NMR (101 MHz, Chloroform-*d*) of **2y-*endo* and 2y-*exo***:

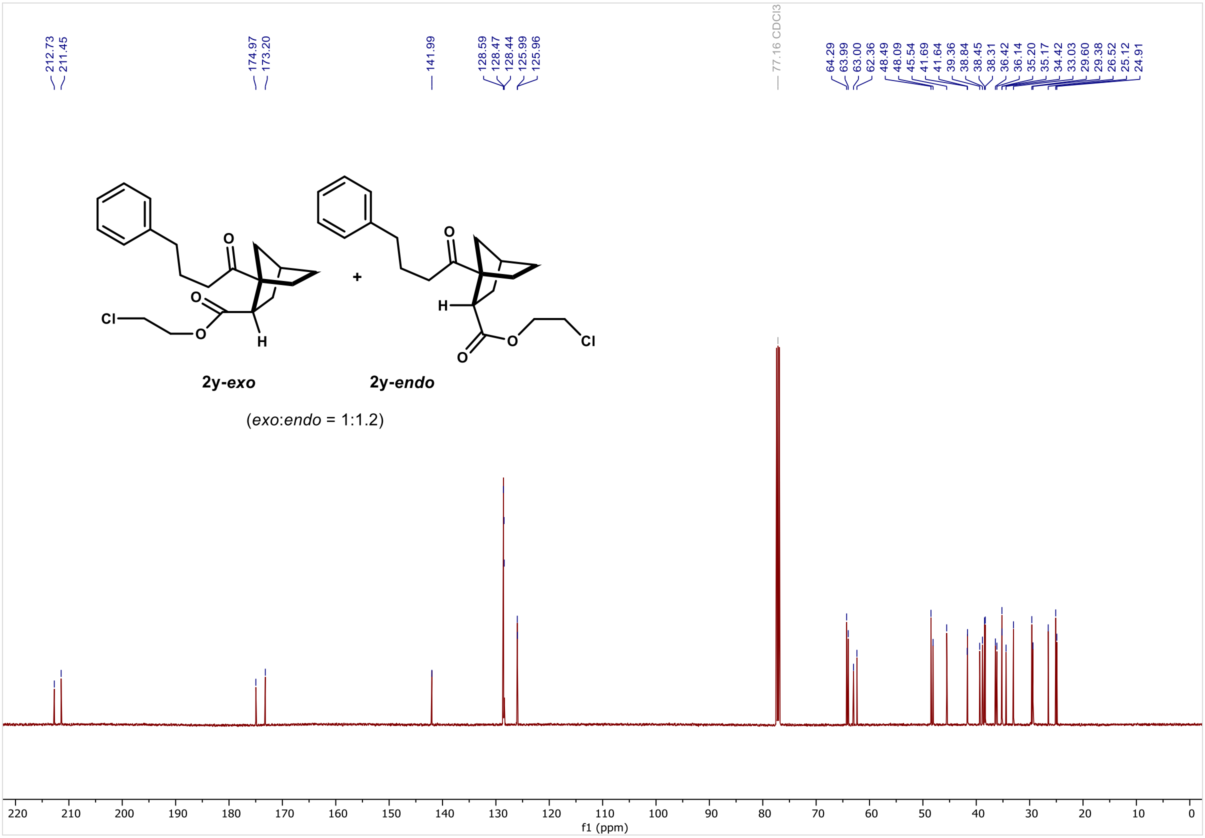

^{1}H NMR (400 MHz, Chloroform-*d*) of **2z-*endo* and 2z-*exo***:

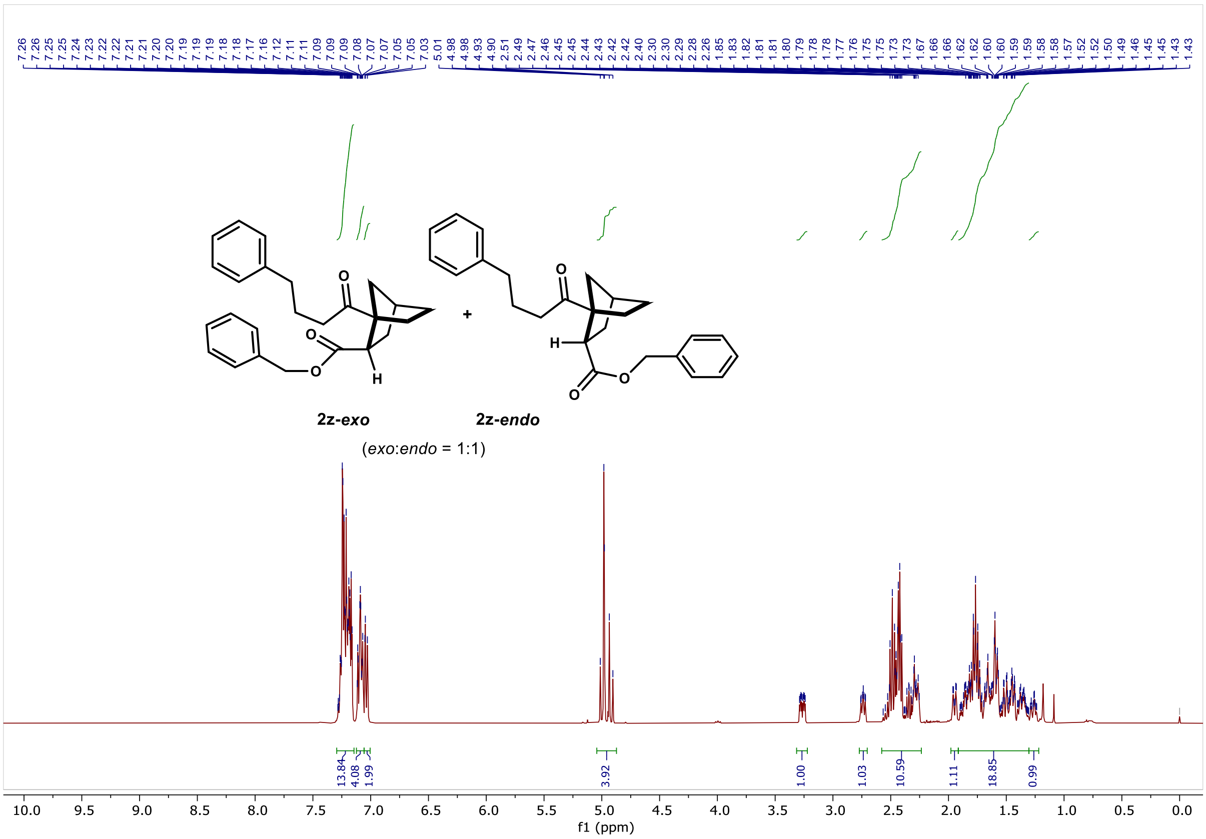

^{13}C NMR (101 MHz, Chloroform-*d*) of **2z-*endo* and 2z-*exo***:

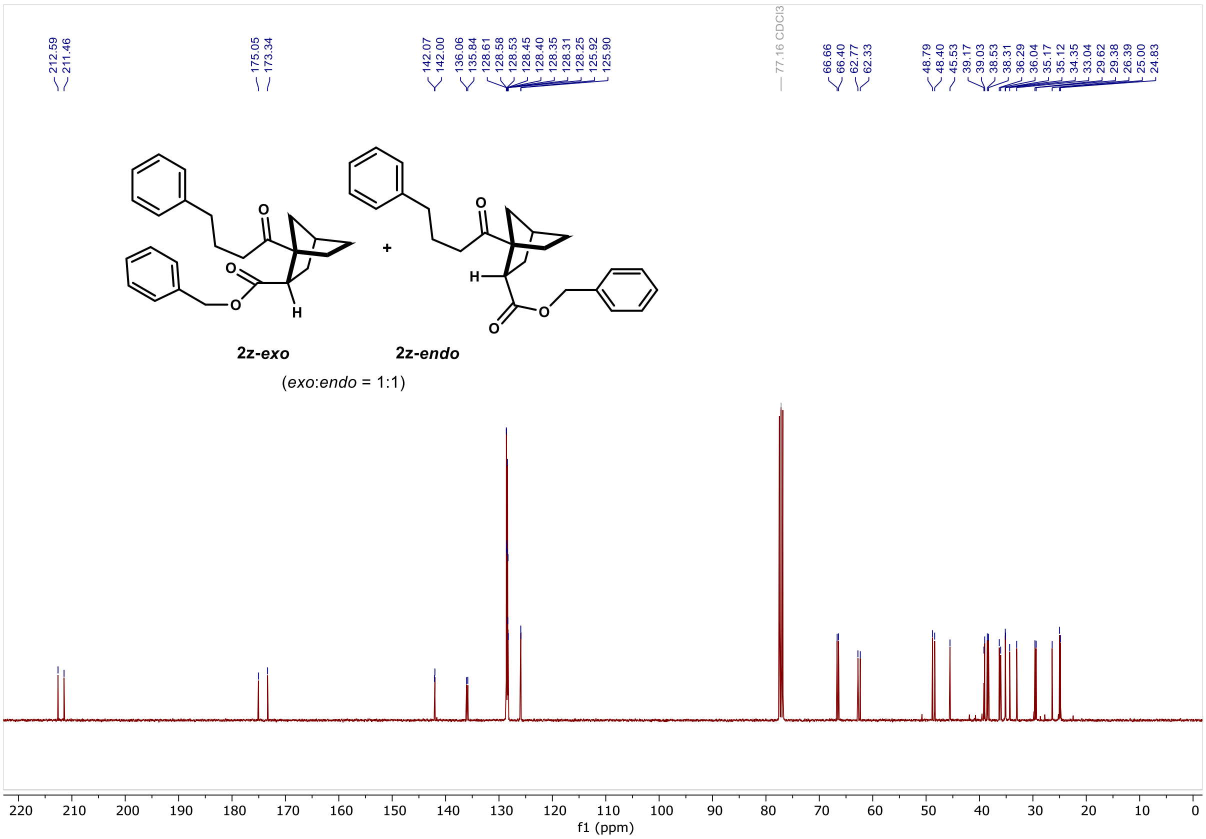

^{1}H NMR (400 MHz, Chloroform-*d*) of **2aa-*endo***

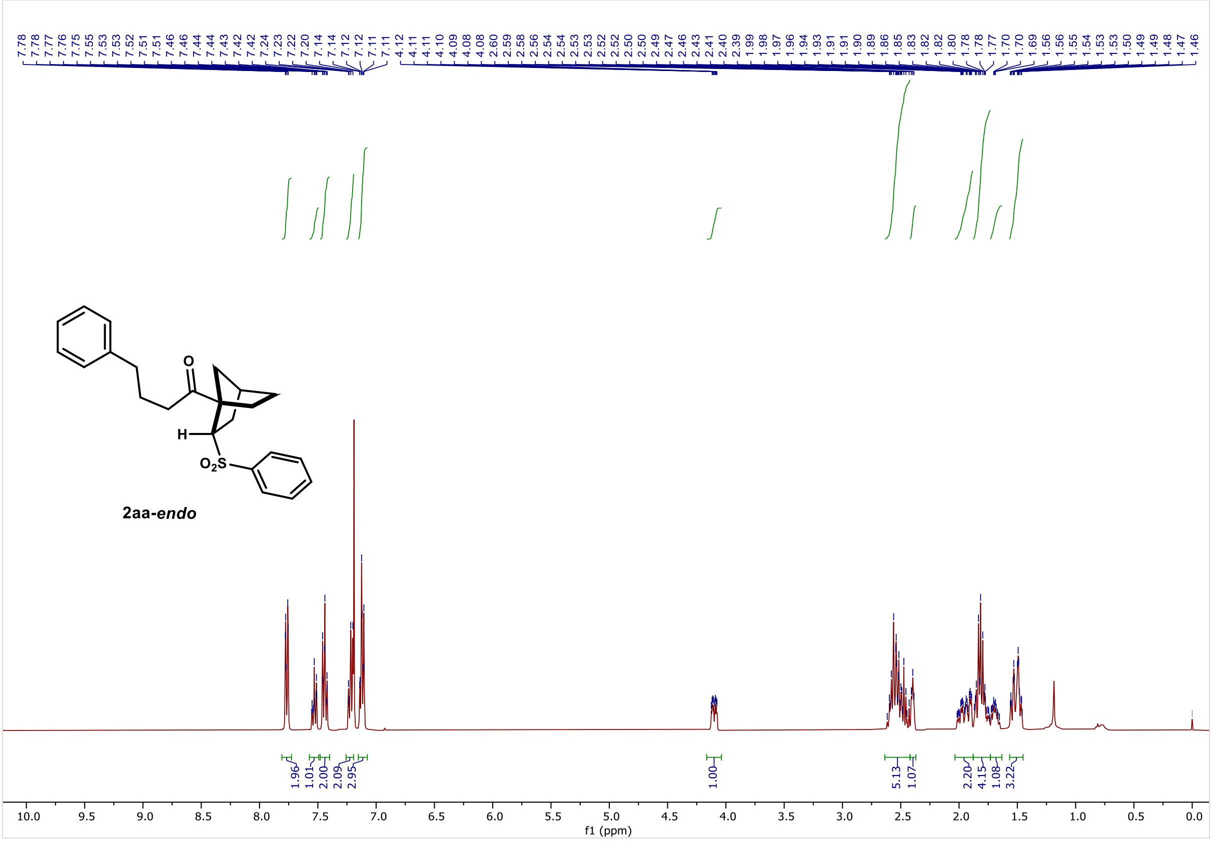

^{13}C NMR (101 MHz, Chloroform-*d*) of **2aa-*endo***

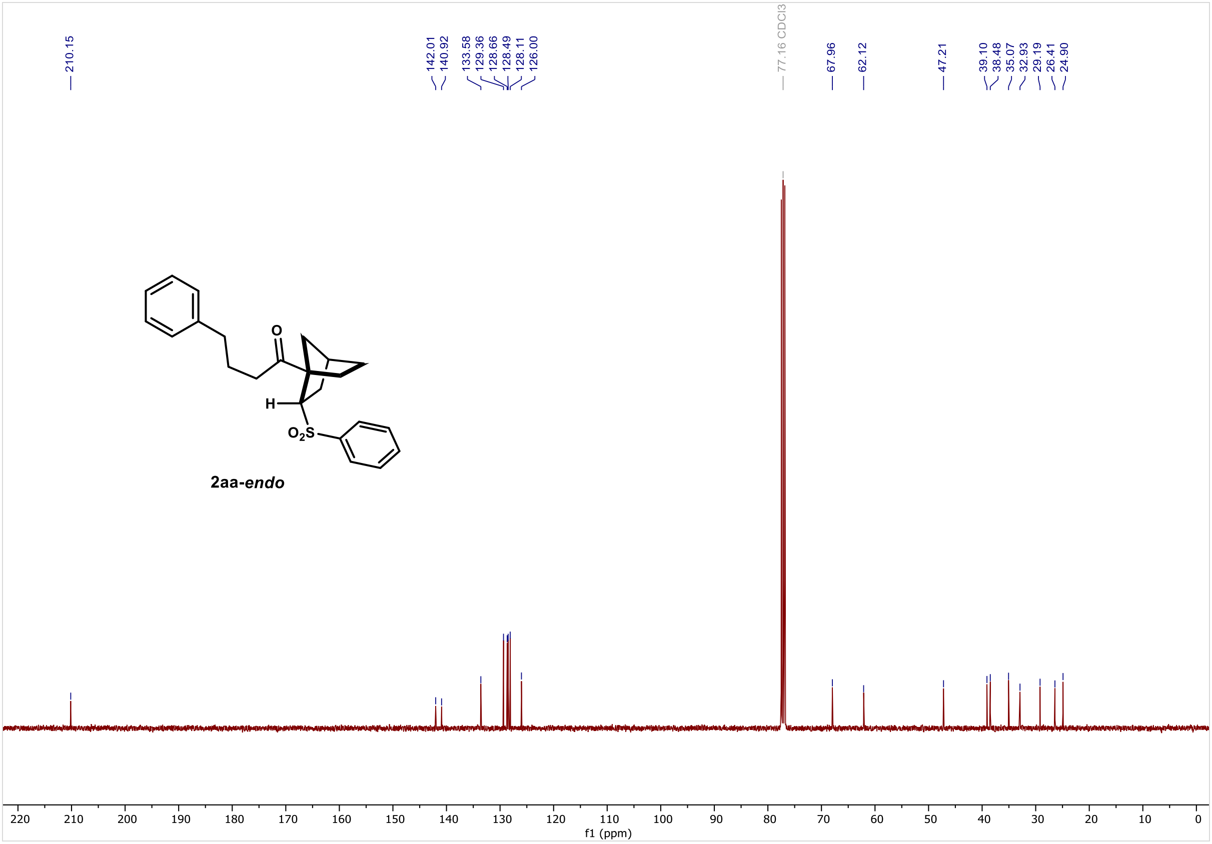

^{1}H NMR (400 MHz, Chloroform-*d*) of **2aa-*exo***

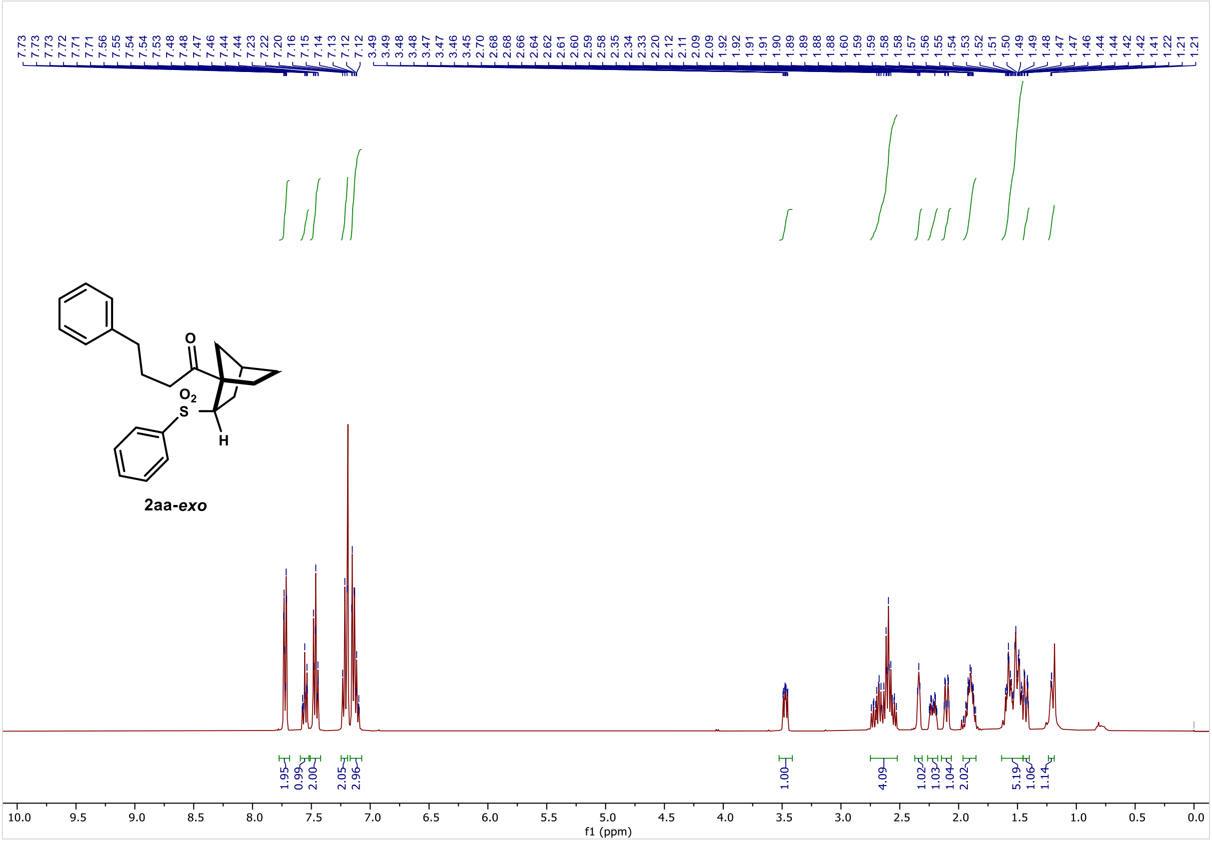

^{13}C NMR (101 MHz, Chloroform-*d*) of **2aa-*exo***

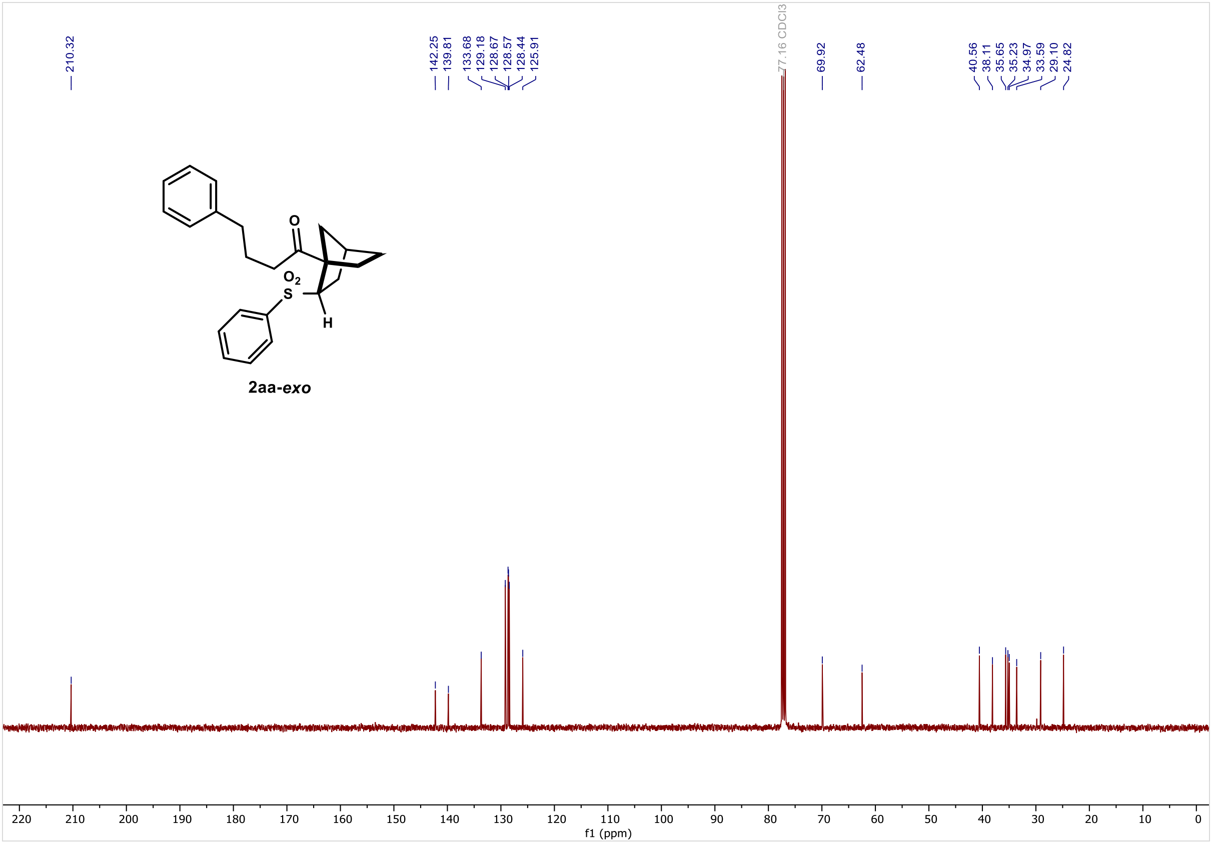

^{1}H NMR (400 MHz, Chloroform-*d*) of **2ab-*endo***:

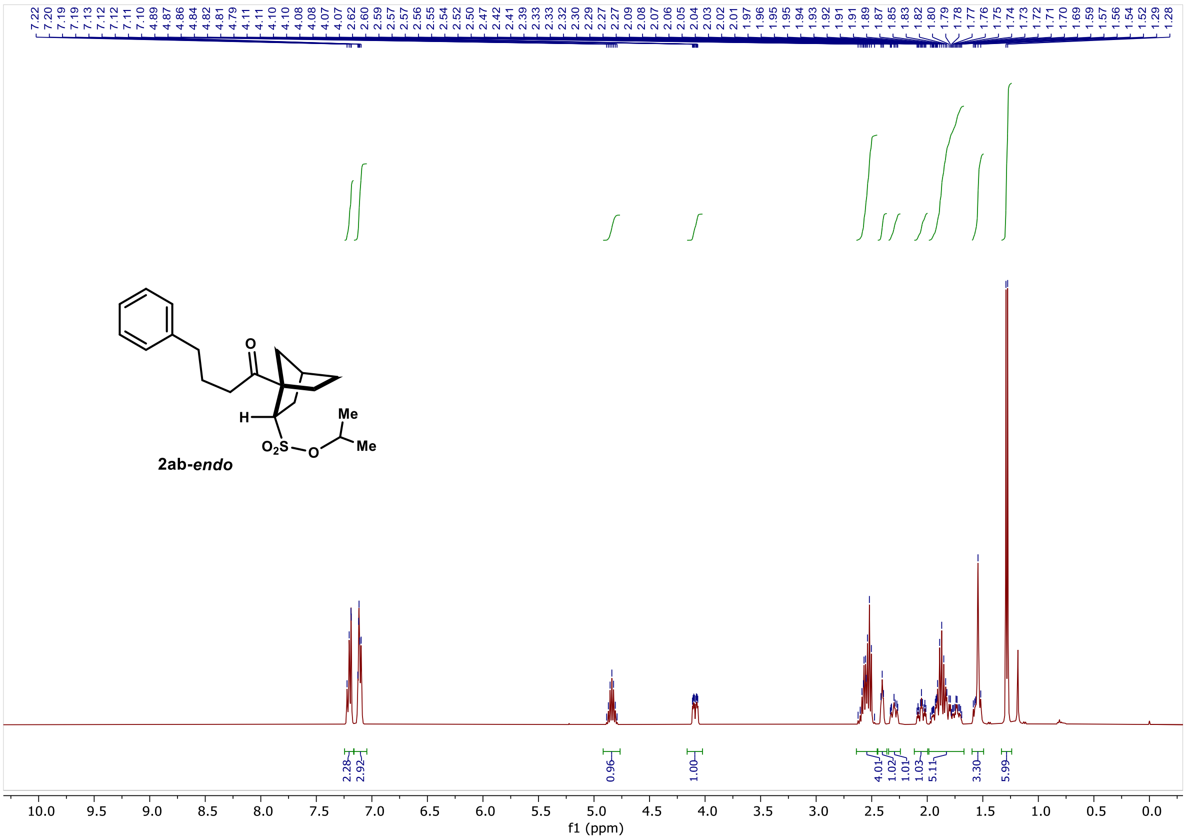

^{13}C NMR (101 MHz, Chloroform-*d*) of **2ab-*endo***:

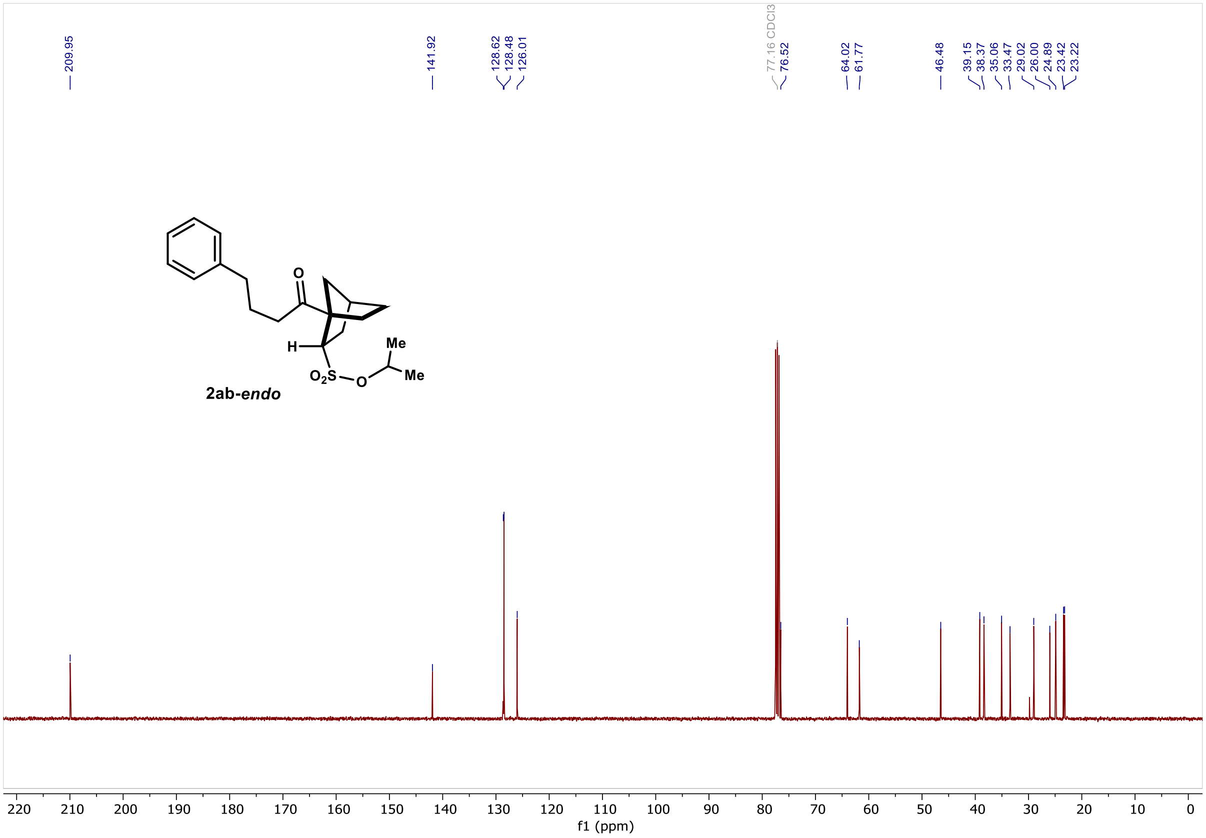

^{1}H NMR (400 MHz, Chloroform-*d*) of **2ab-*exo***:

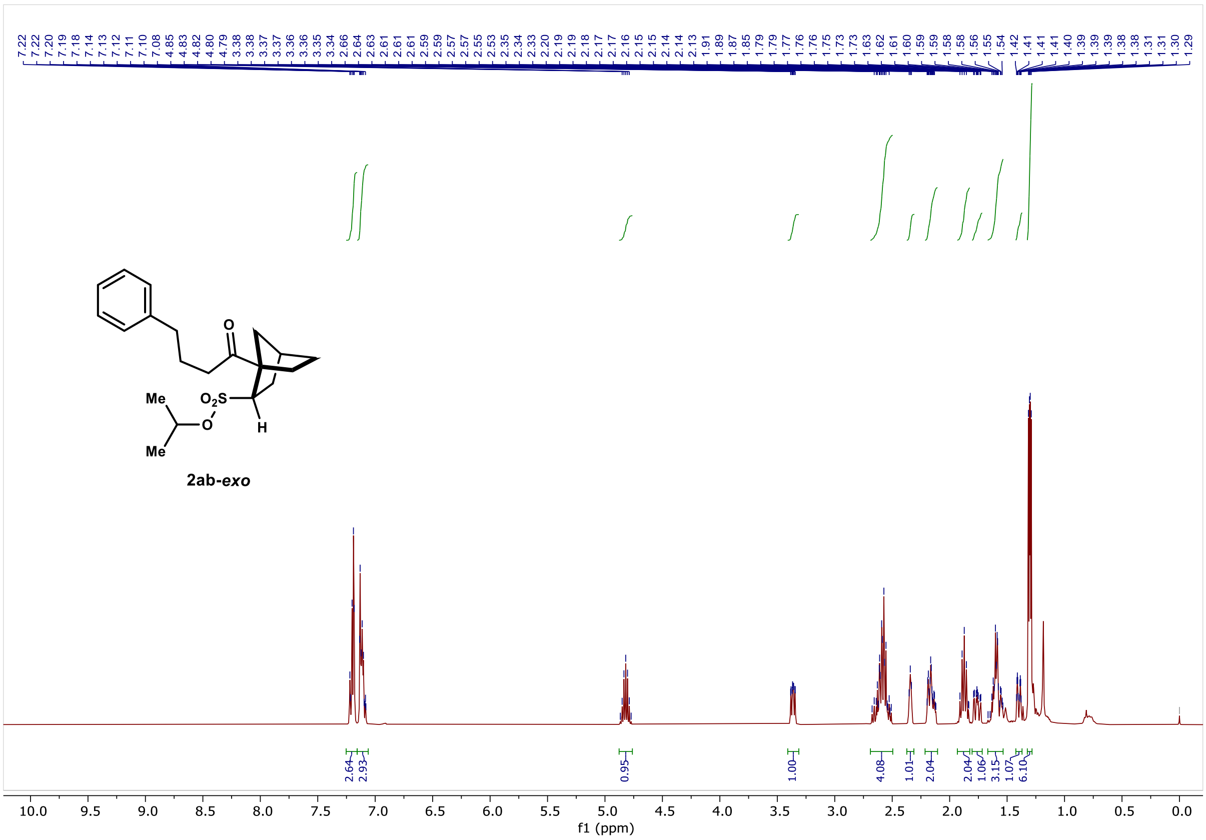

^{13}C NMR (101 MHz, Chloroform-*d*) of **2ab-*exo***:

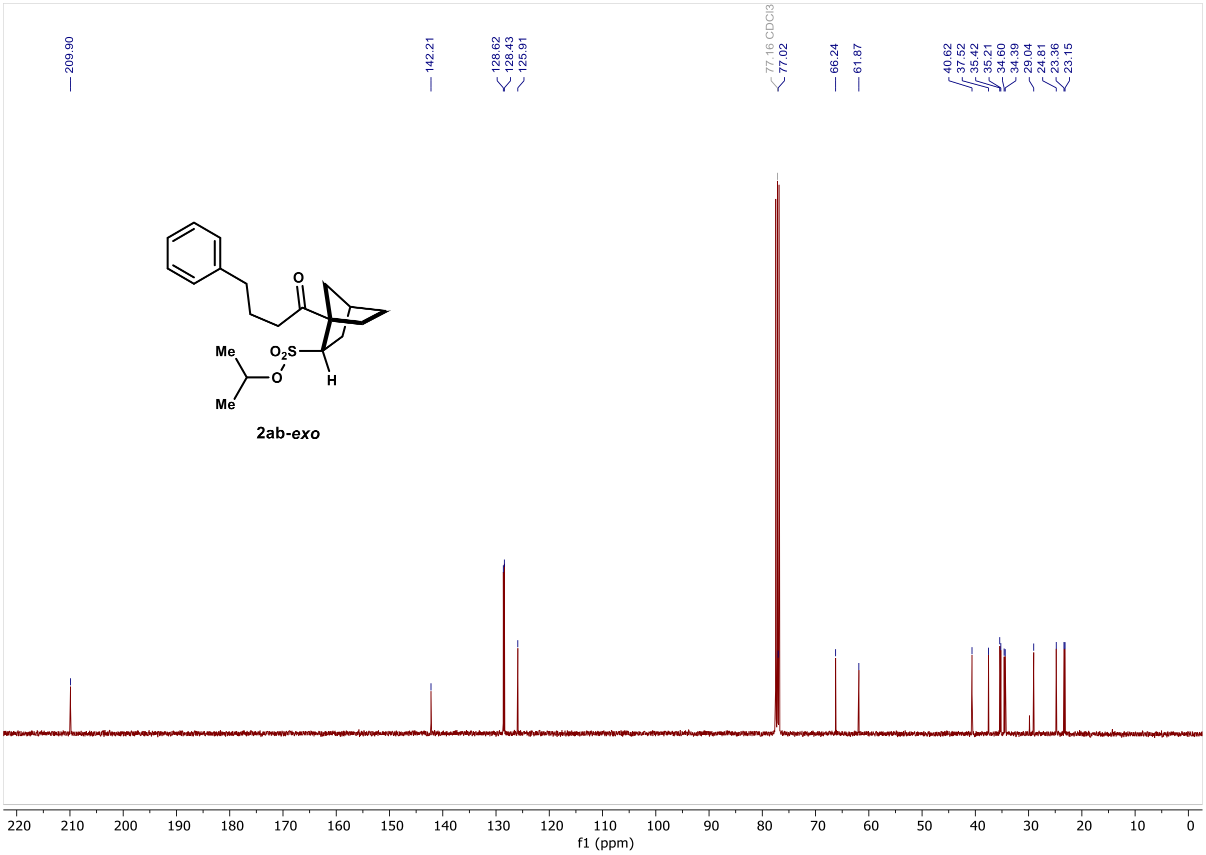

^{1}H NMR (400 MHz, Chloroform-*d*) of **2ac-*endo***:

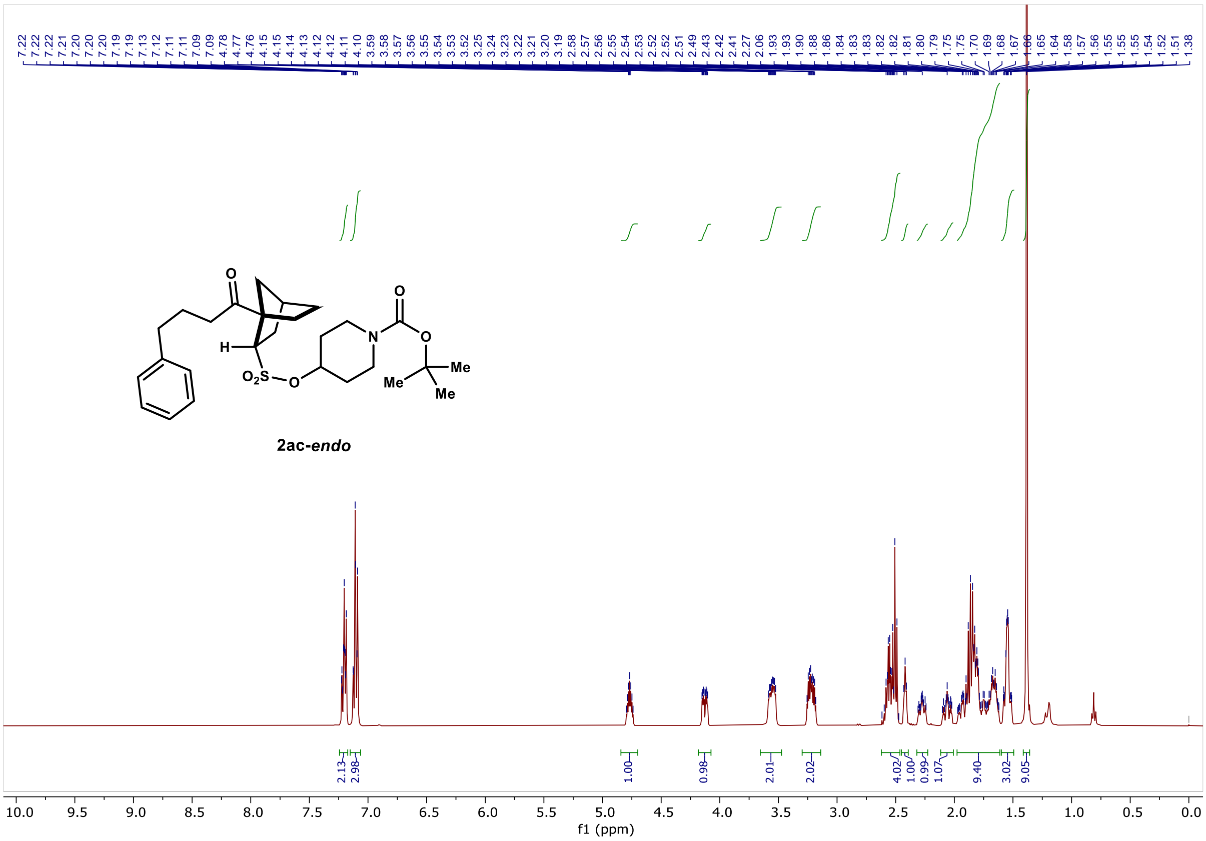

^{13}C NMR (126 MHz, Chloroform-*d*) of **2ac-*endo***:

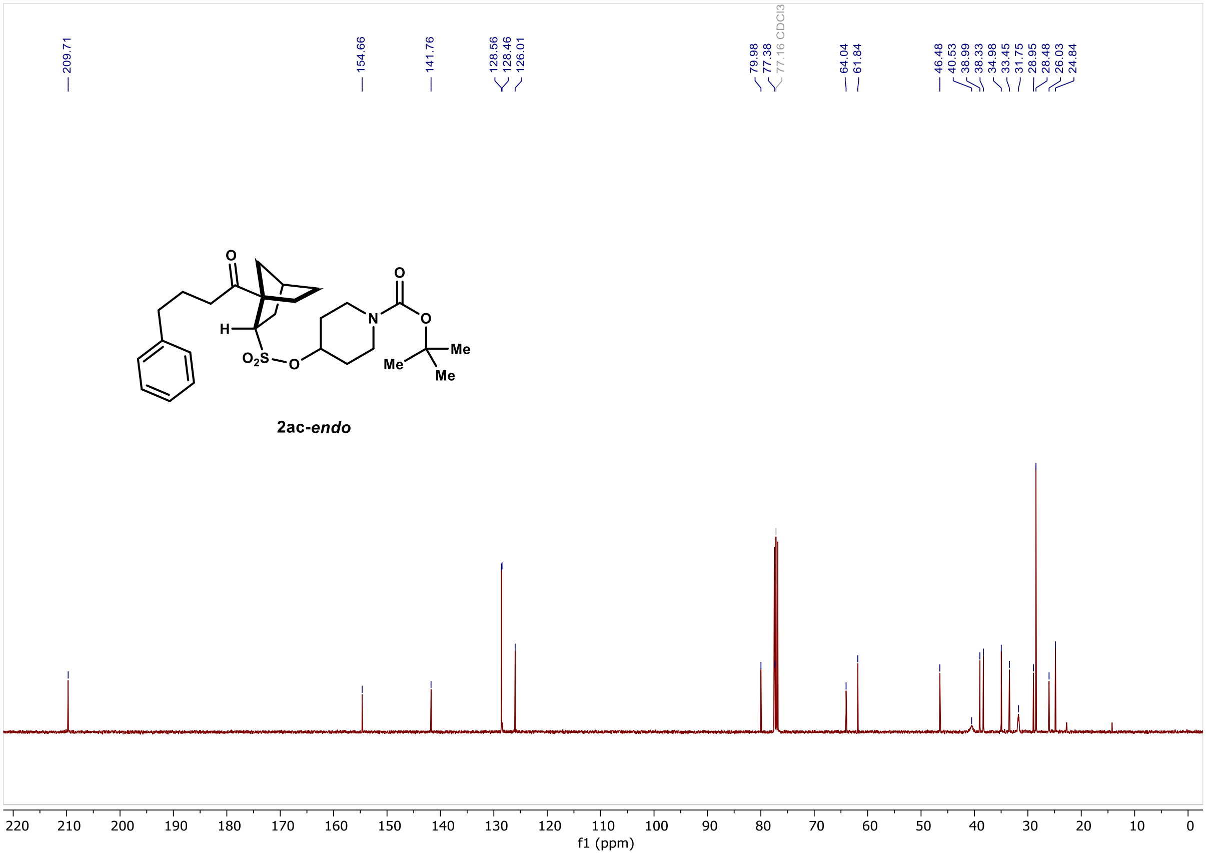

^{1}H NMR (400 MHz, Chloroform-*d*) of **2ac-*exo***:

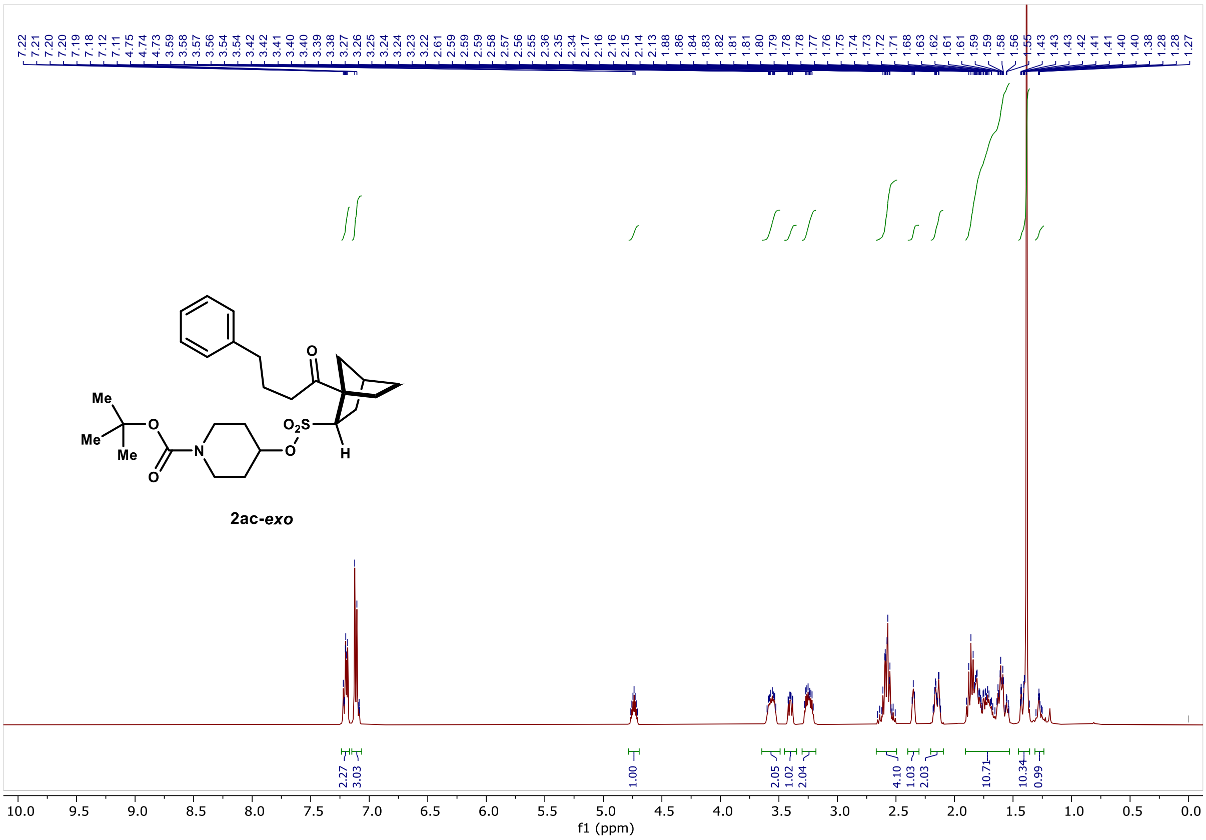

^{13}C NMR (101 MHz, Chloroform-*d*) of **2ac-*exo***:

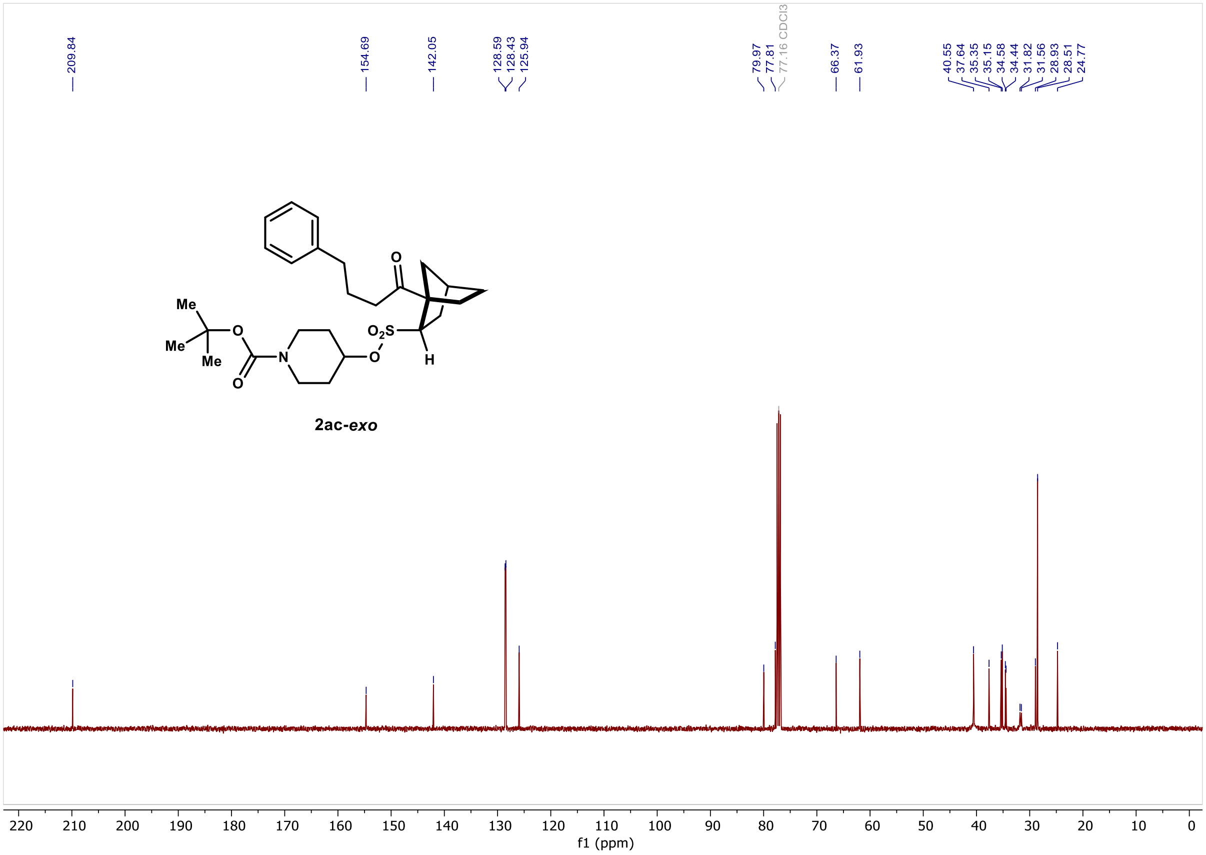

^{1}H NMR (400 MHz, Chloroform-*d*) of **2ad-*endo***:

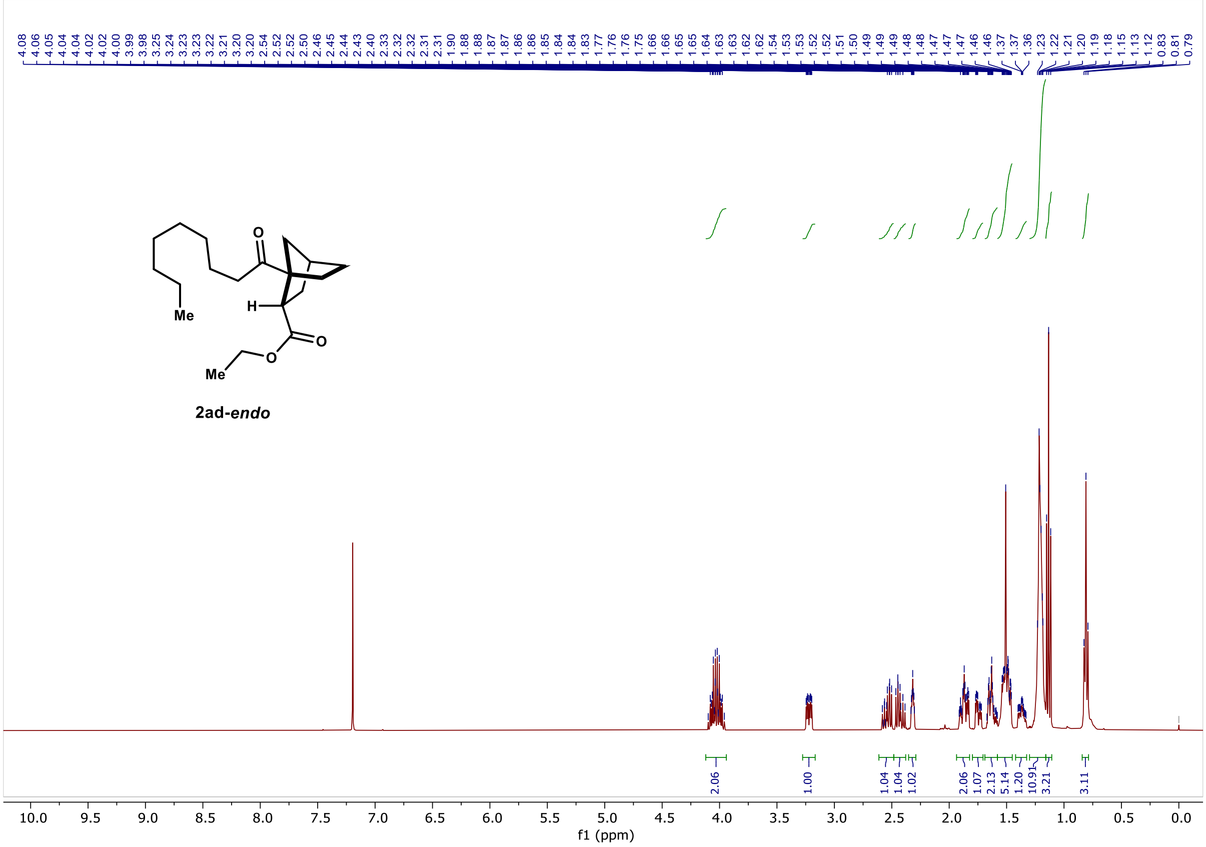

^{13}C NMR (101 MHz, Chloroform-*d*) of **2ad-*endo***:

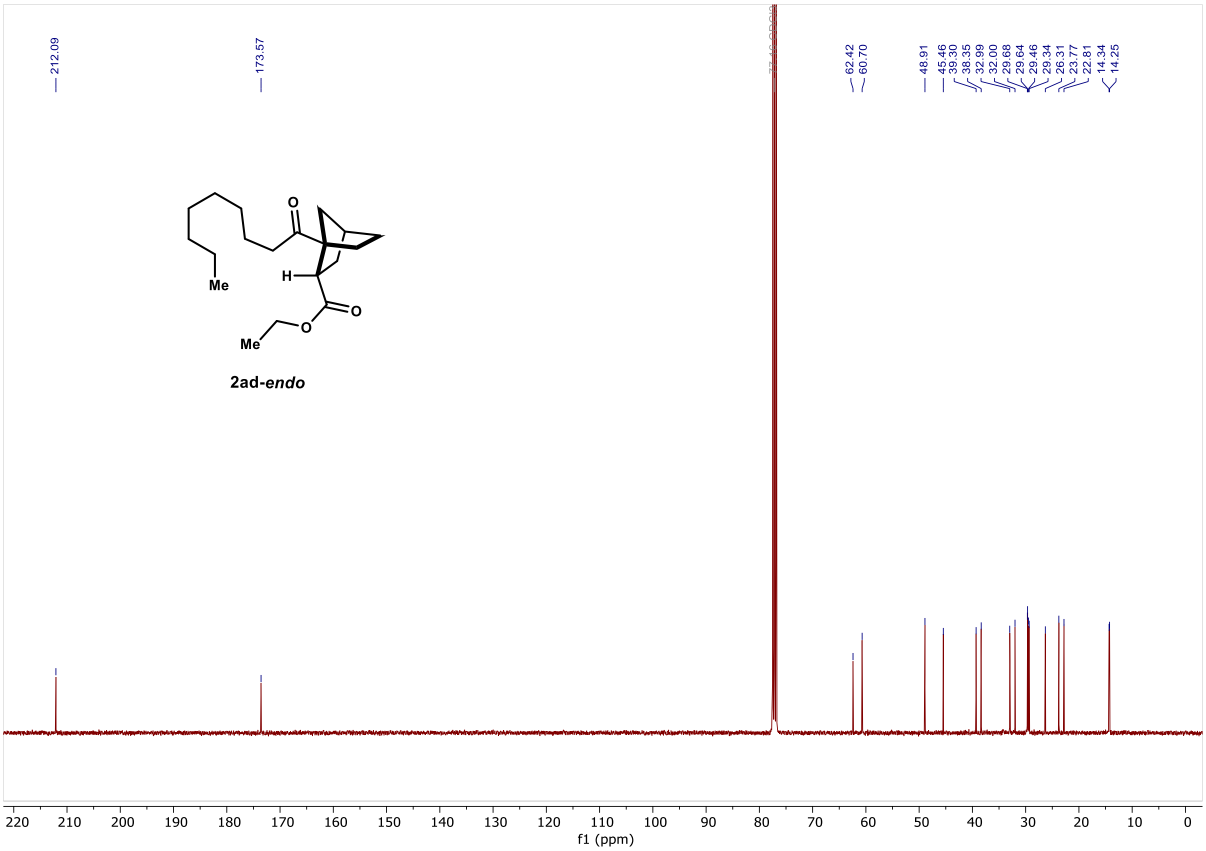

^{1}H NMR (400 MHz, Chloroform-*d*) of **2ad-*exo***:

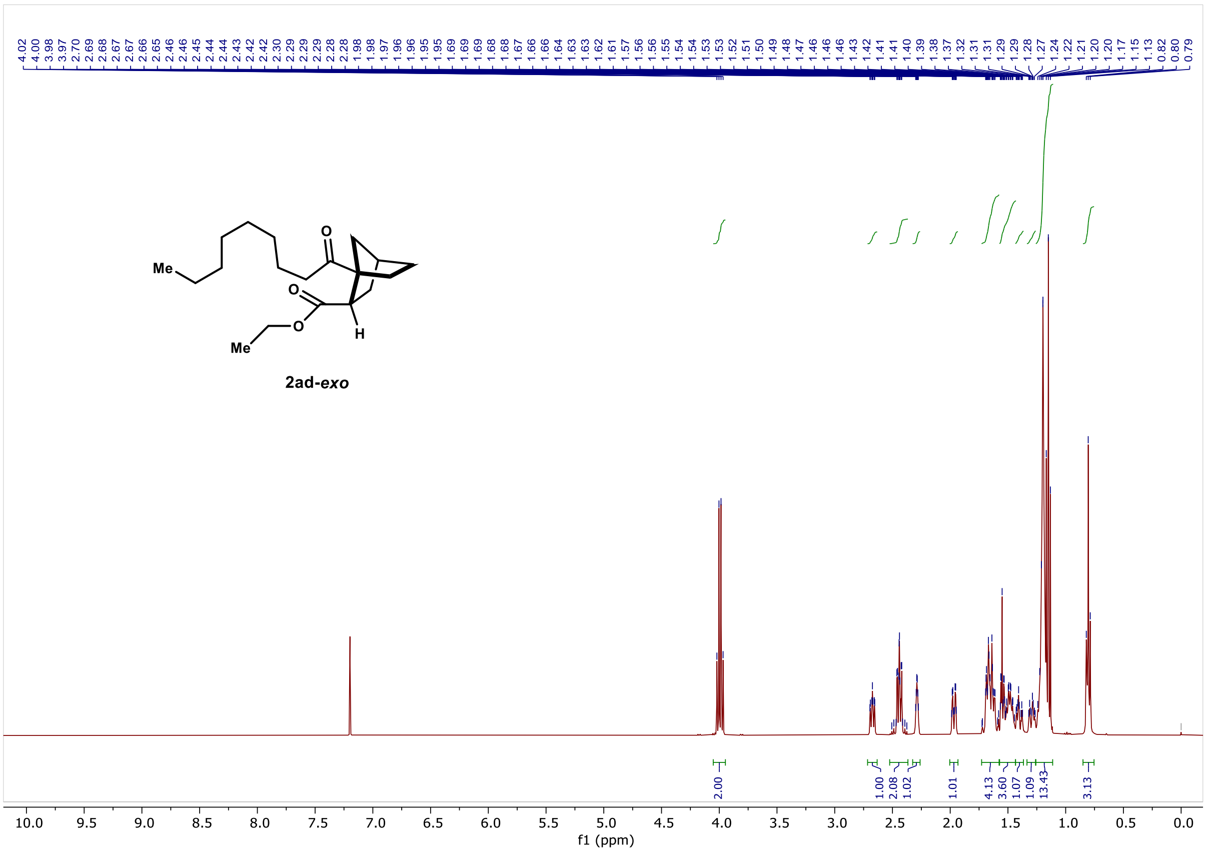

^{13}C NMR (101 MHz, Chloroform-*d*) of **2ad-*exo***:

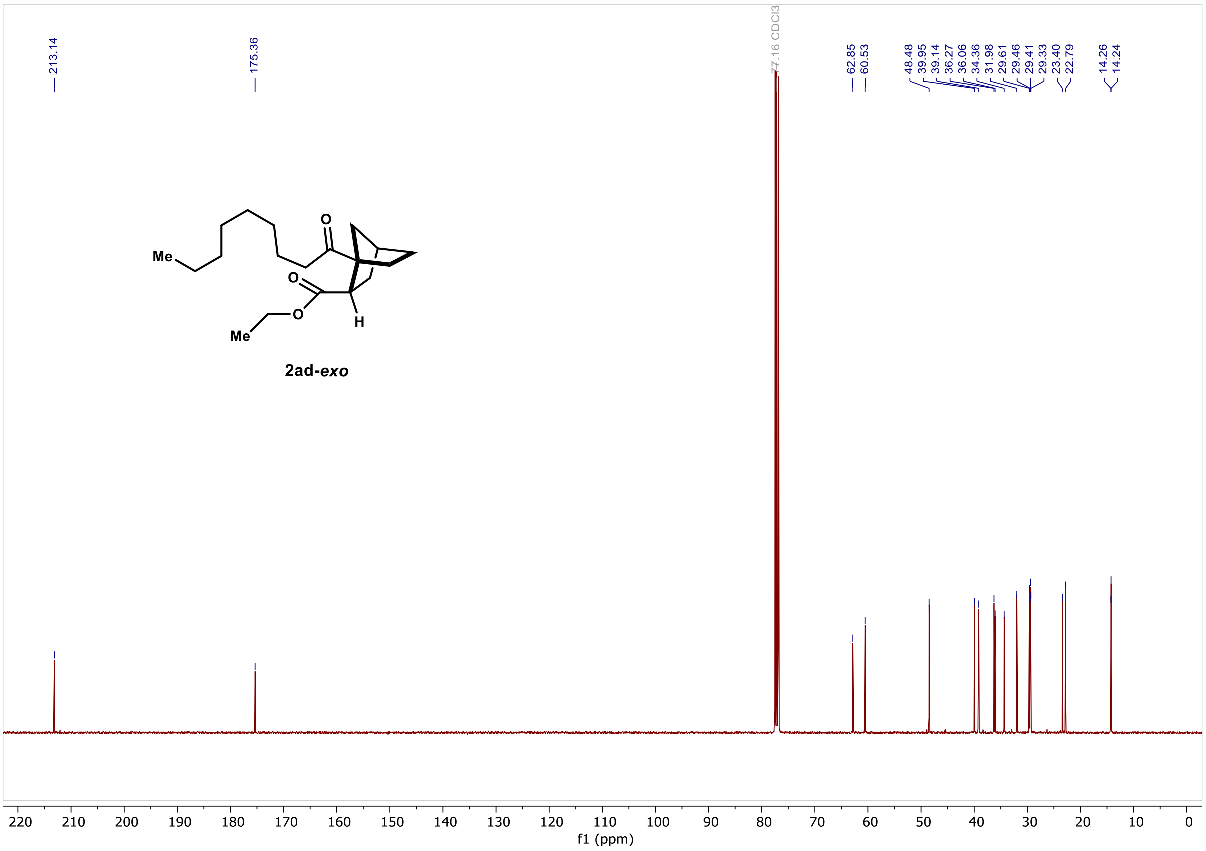

^{1}H NMR (500 MHz, Chloroform-*d*) of **2ae-*endo***:

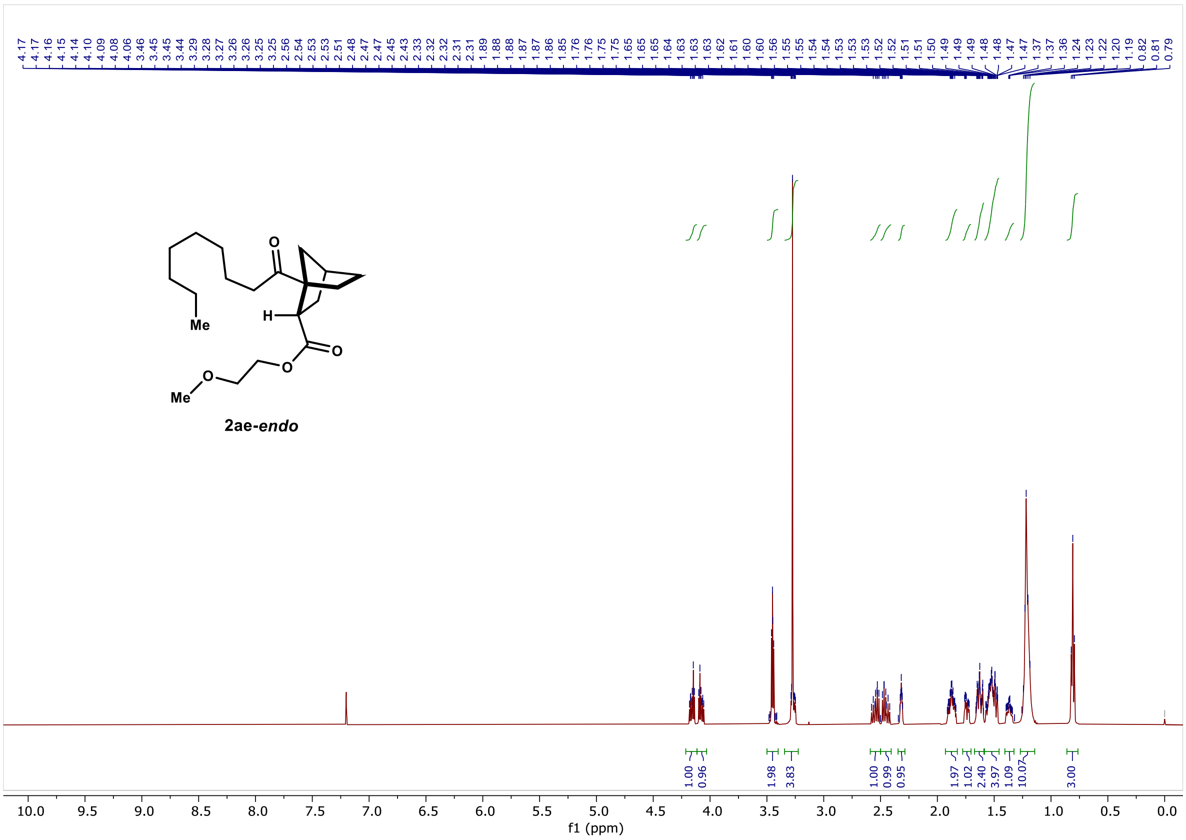

^{13}C NMR (126 MHz, Chloroform-*d*) of **2ae-*endo***:

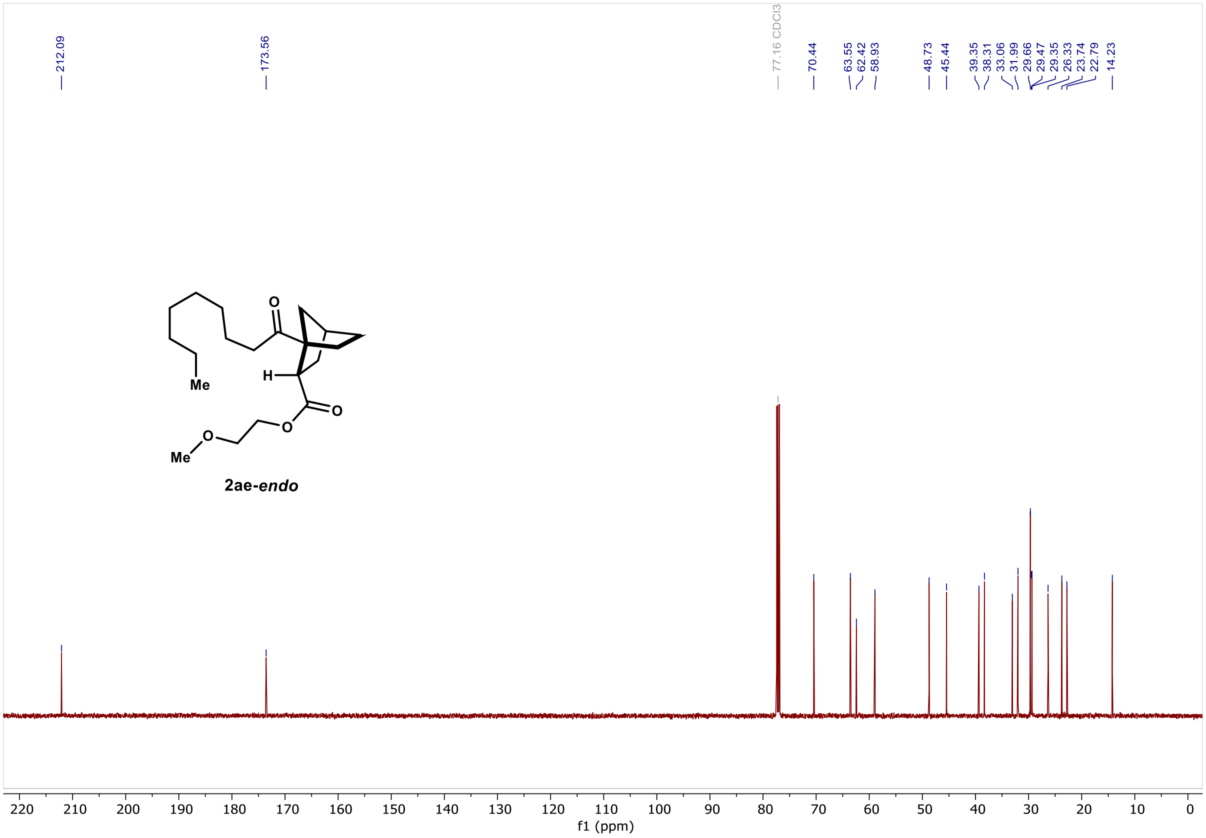

^{1}H NMR (500 MHz, Chloroform-*d*) of **2ae-*exo***:

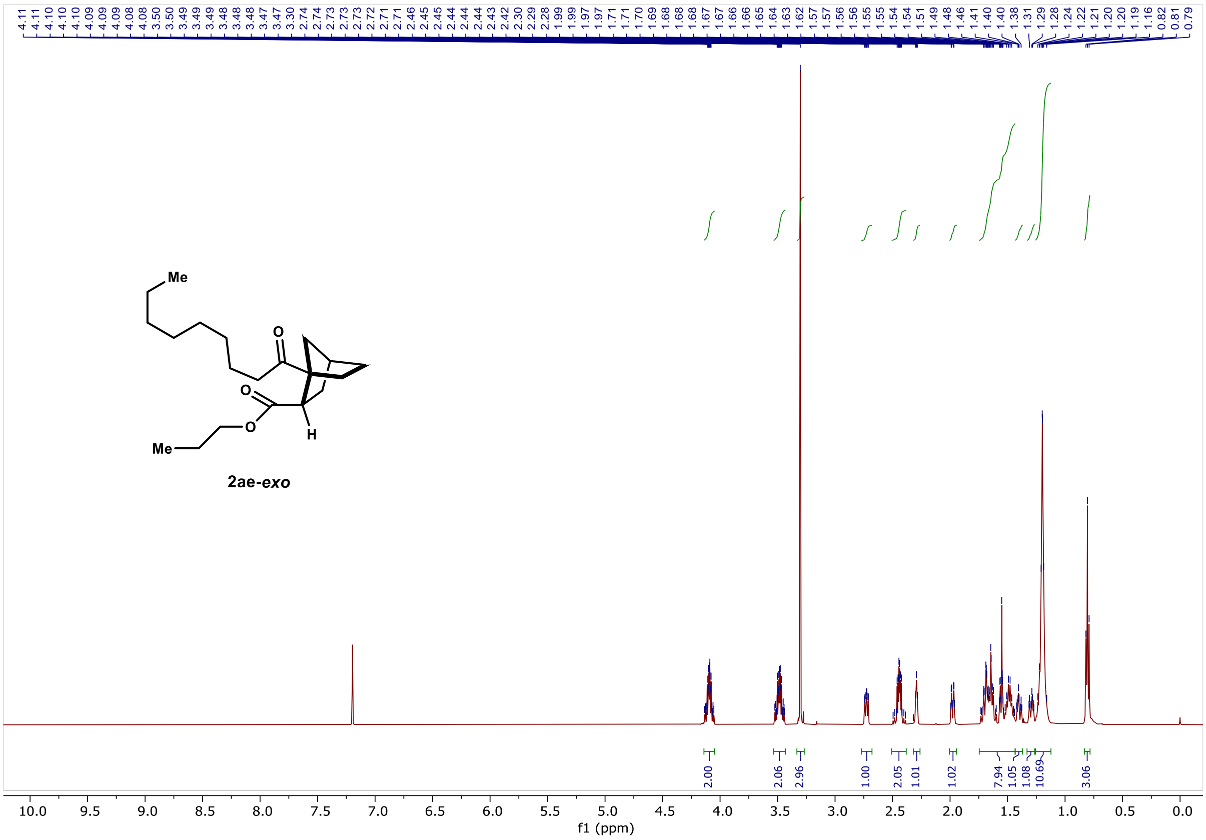

^{13}C NMR (126 MHz, Chloroform-*d*) of **2ae-*exo***:

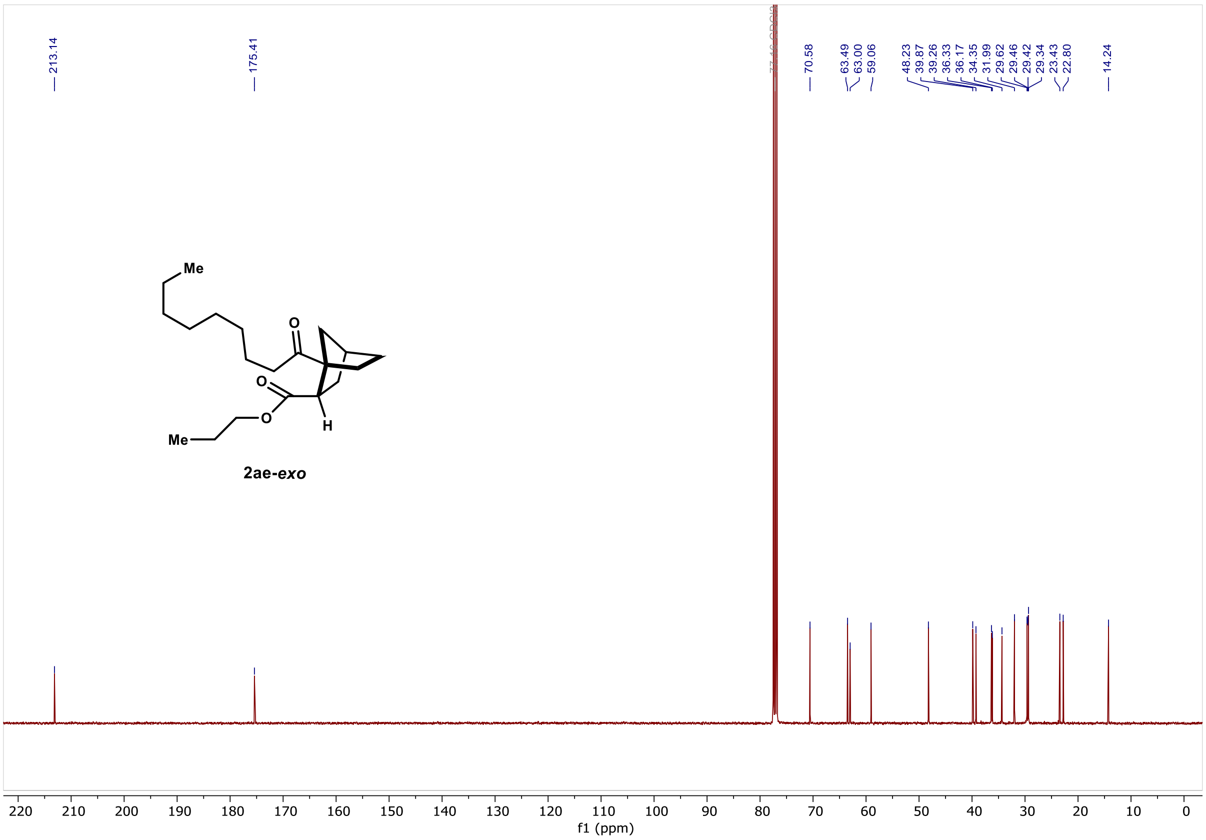

^{1}H NMR (500 MHz, Chloroform-*d*) of **2af-*endo***:

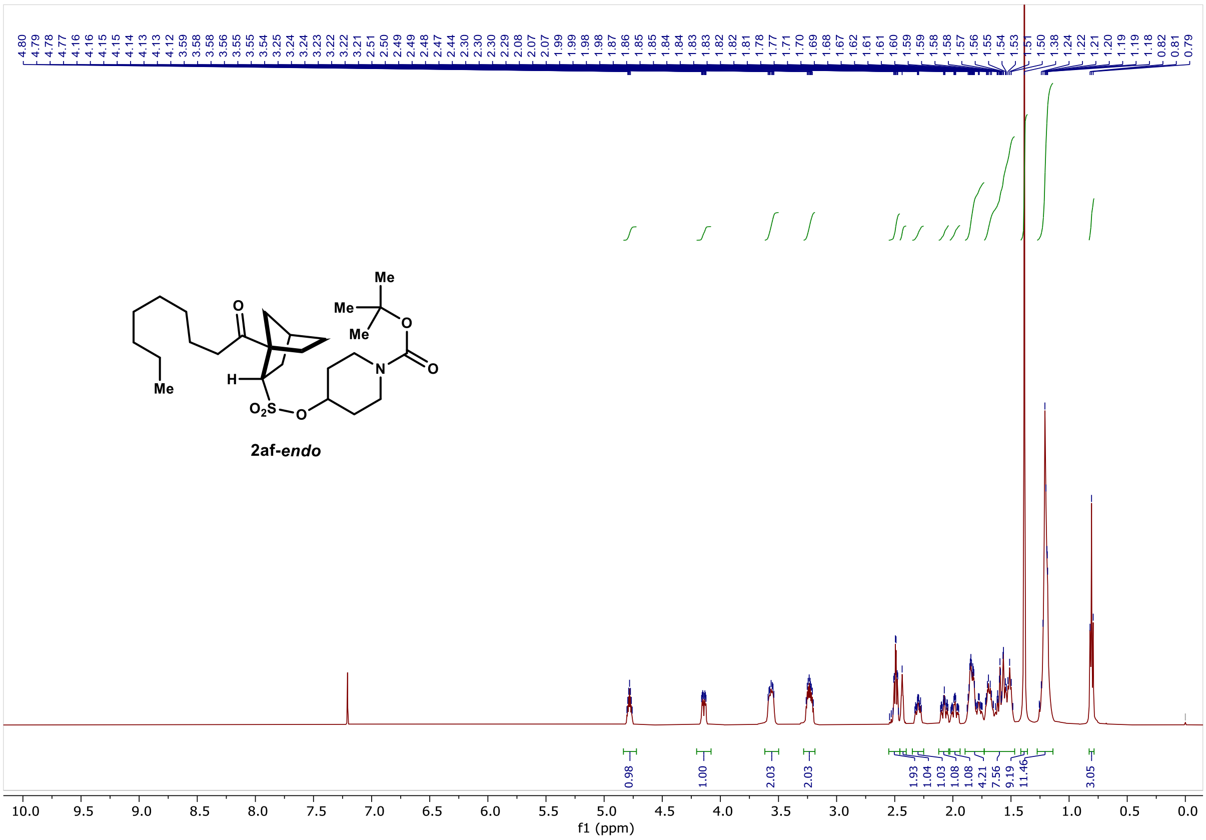

^{13}C NMR (126 MHz, Chloroform-*d*) of **2af-*endo***:

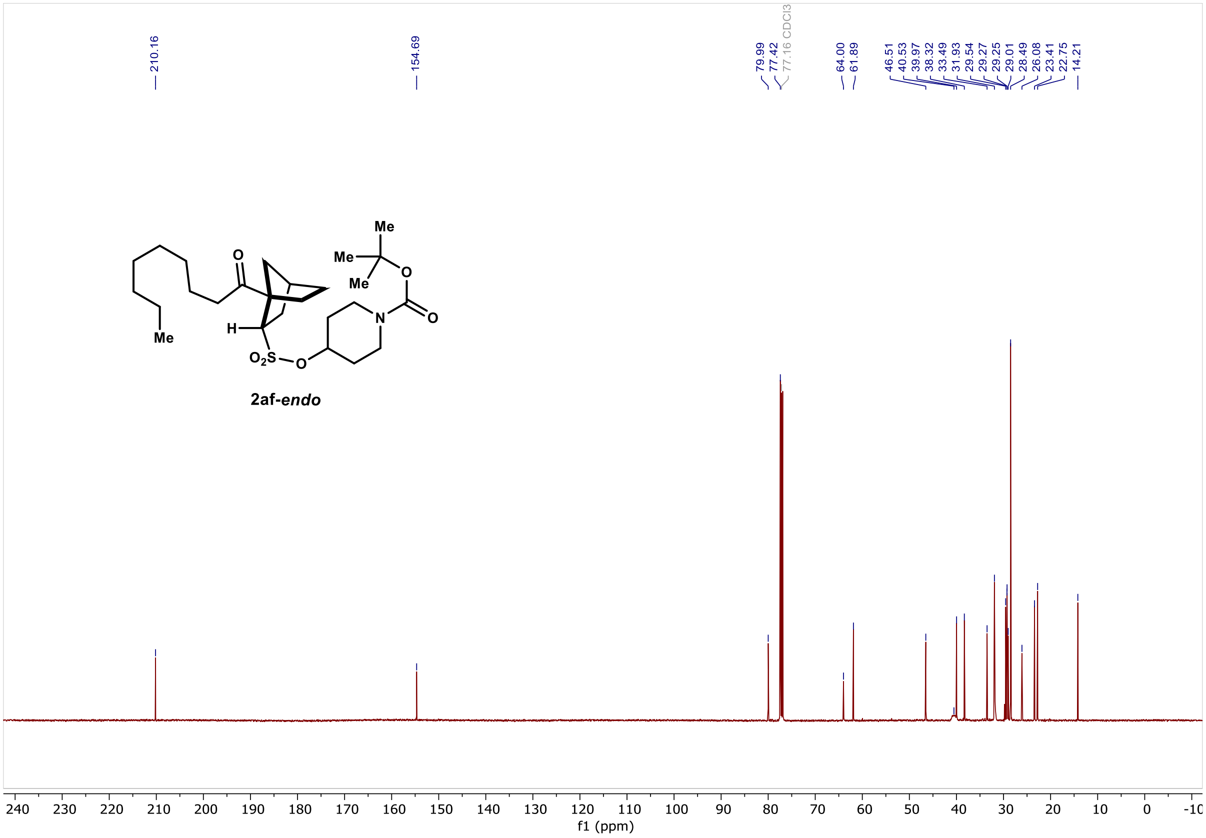

^{1}H NMR (500 MHz, Chloroform-*d*) of **2af-*exo***:

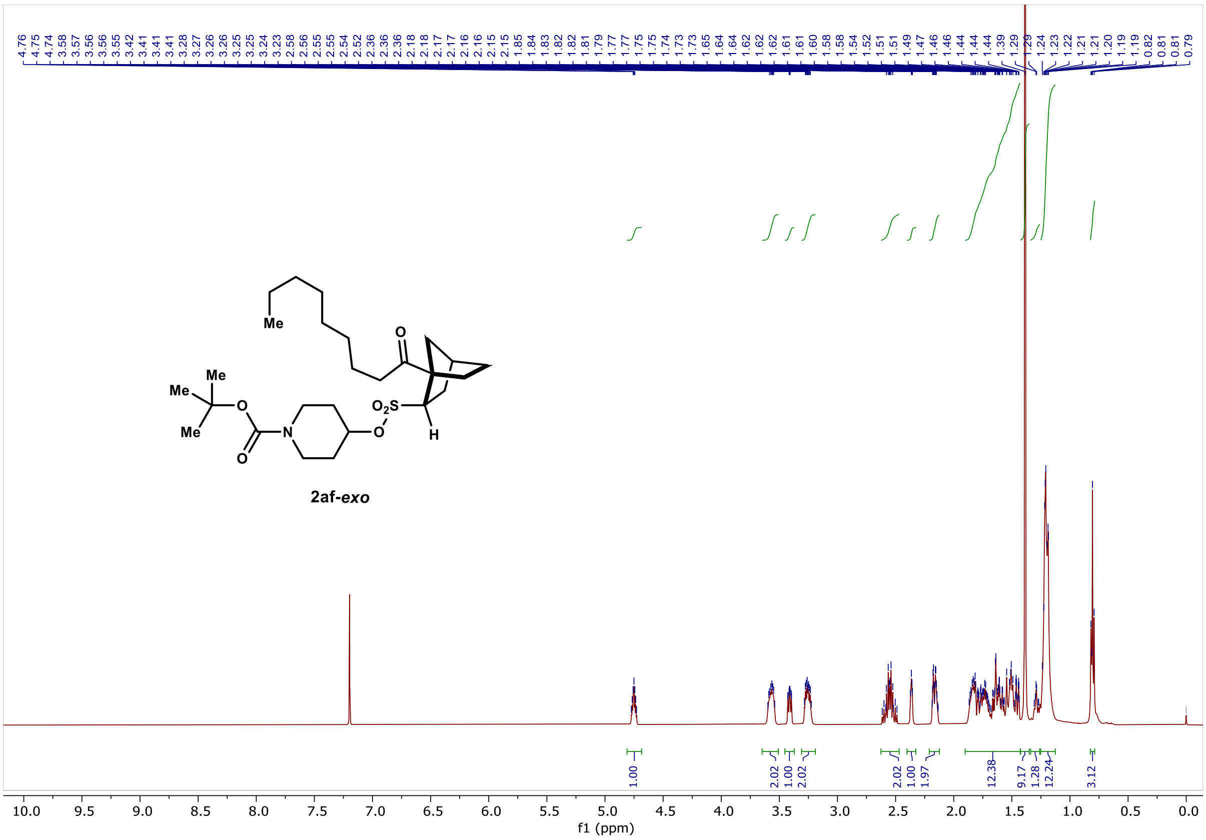

^{13}C NMR (126 MHz, Chloroform-*d*) of **2af-*exo***:

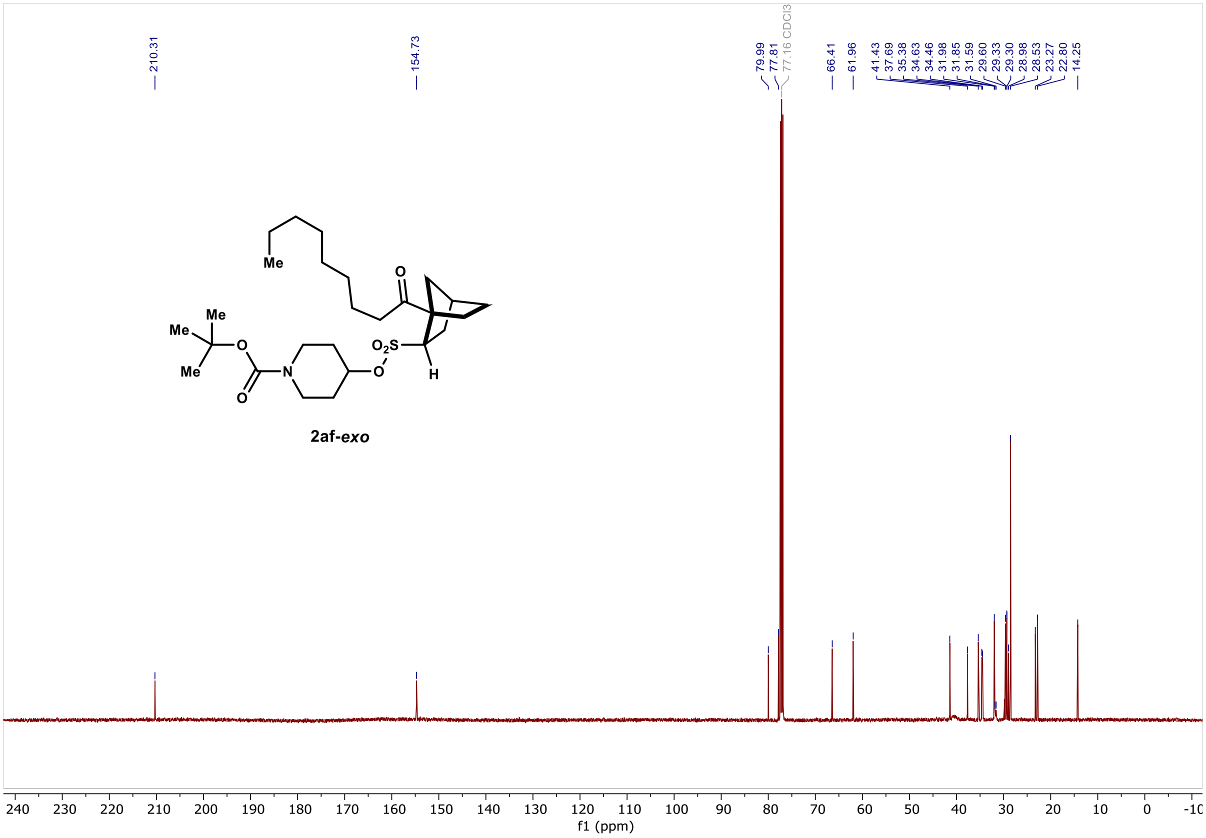

^{1}H NMR (400 MHz, Chloroform-*d*) of **2ag-*endo***:

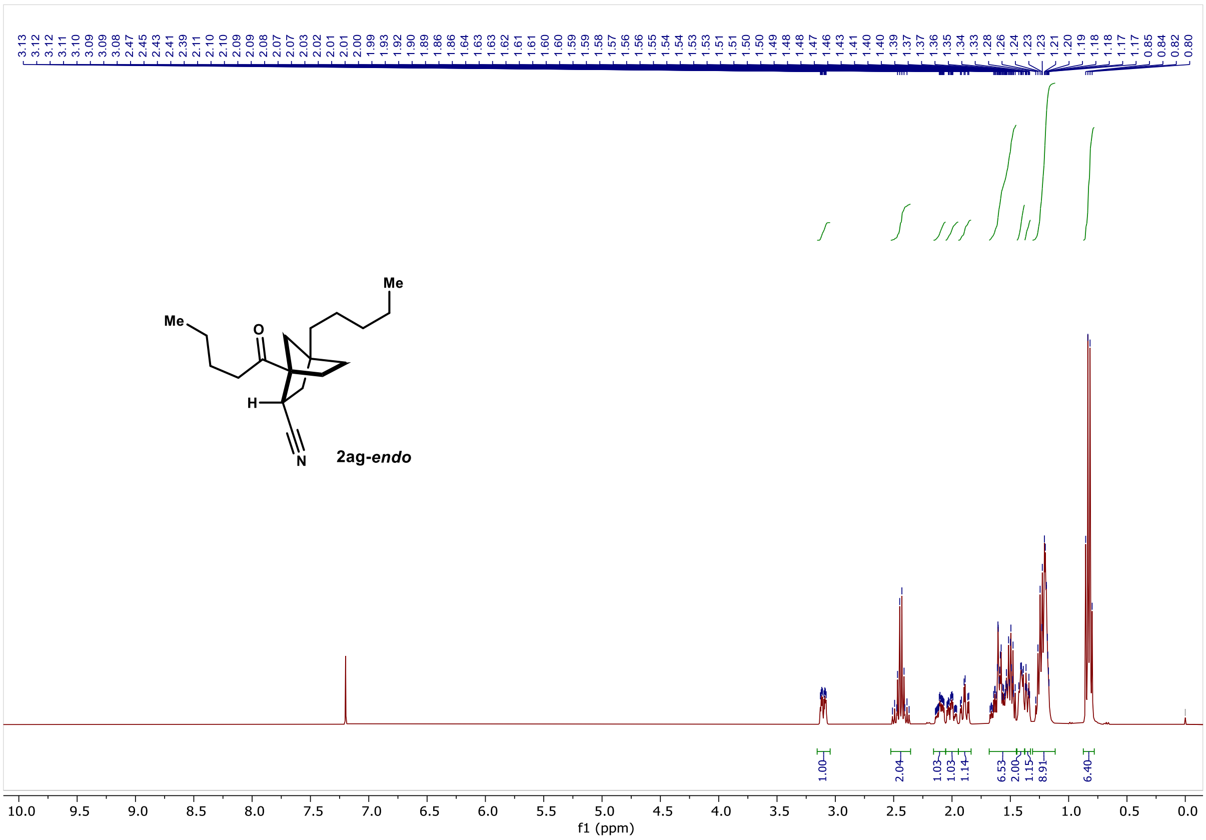

^{13}C NMR (101 MHz, Chloroform-*d*) of **2ag-*endo***:

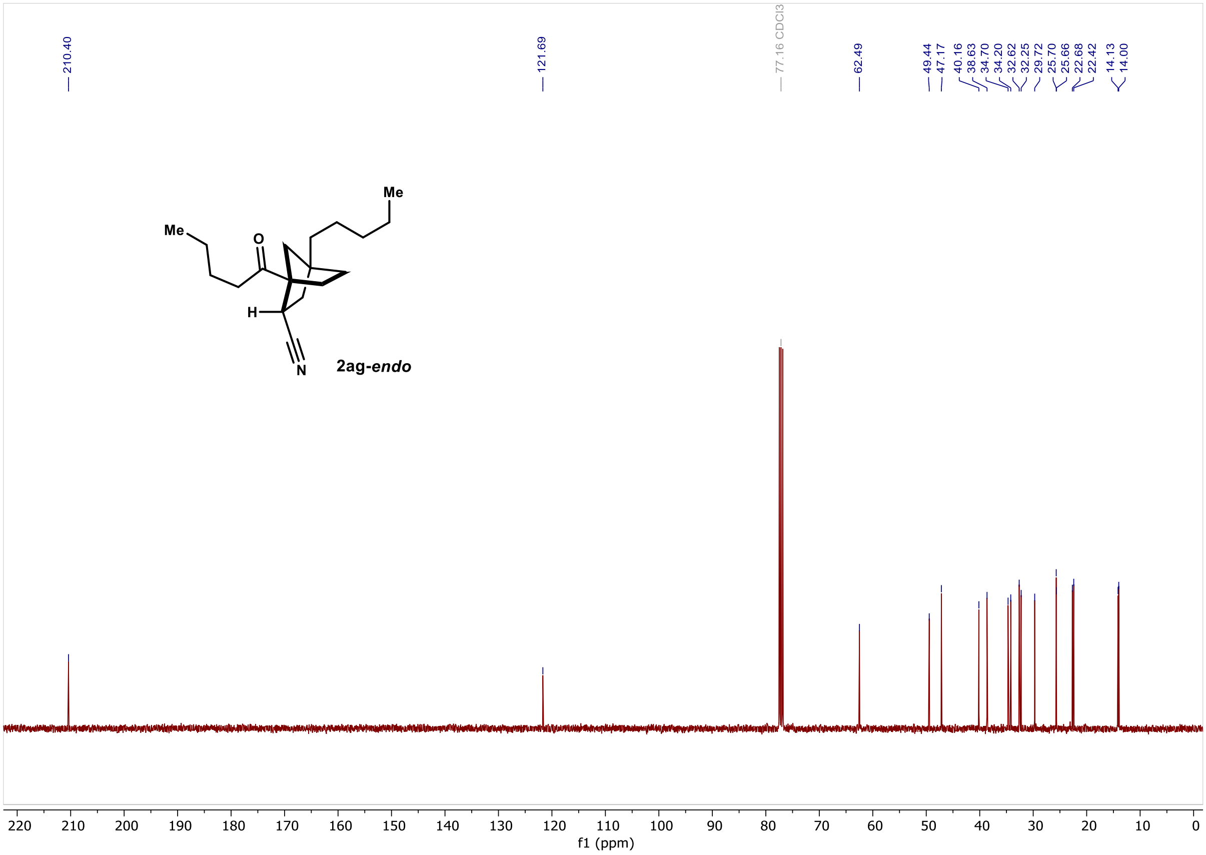

^{1}H NMR (400 MHz, Chloroform-*d*) of **2ag-*exo***:

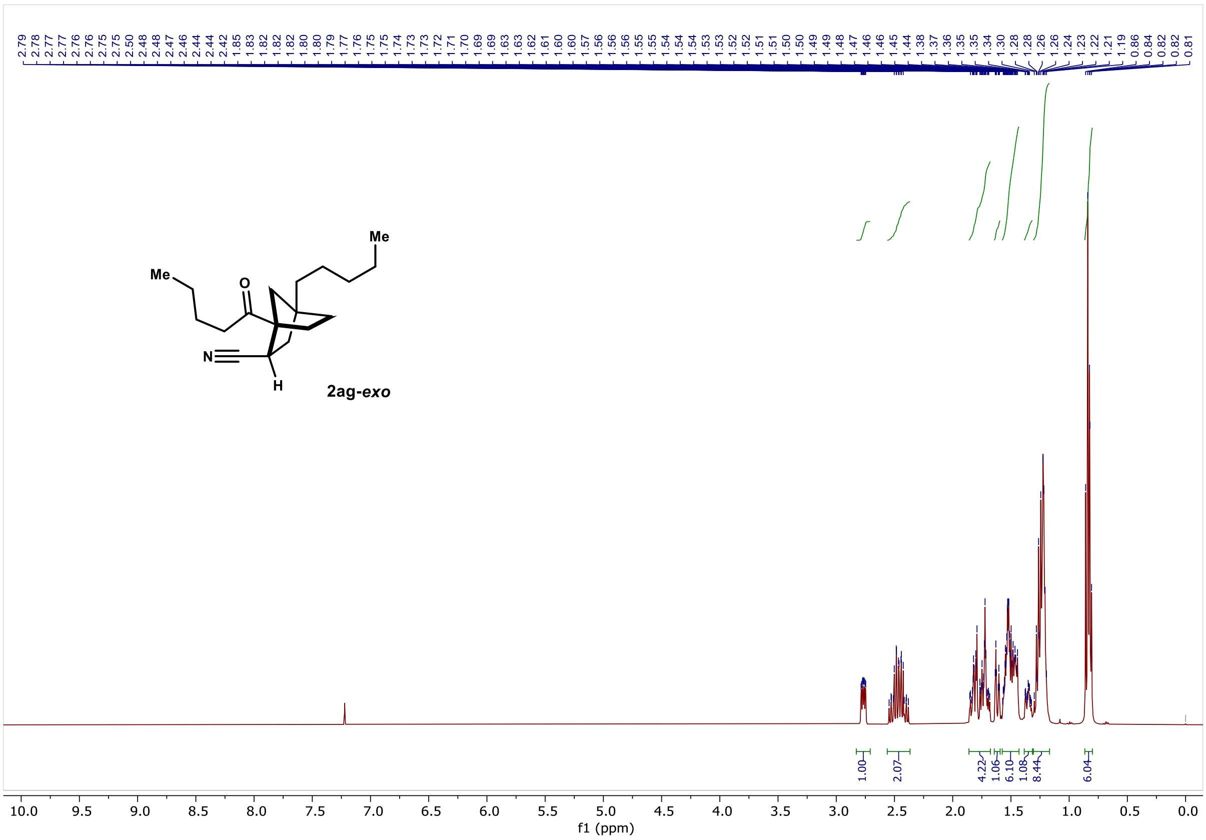

^{13}C NMR (101 MHz, Chloroform-*d*) of **2ag-*exo***:

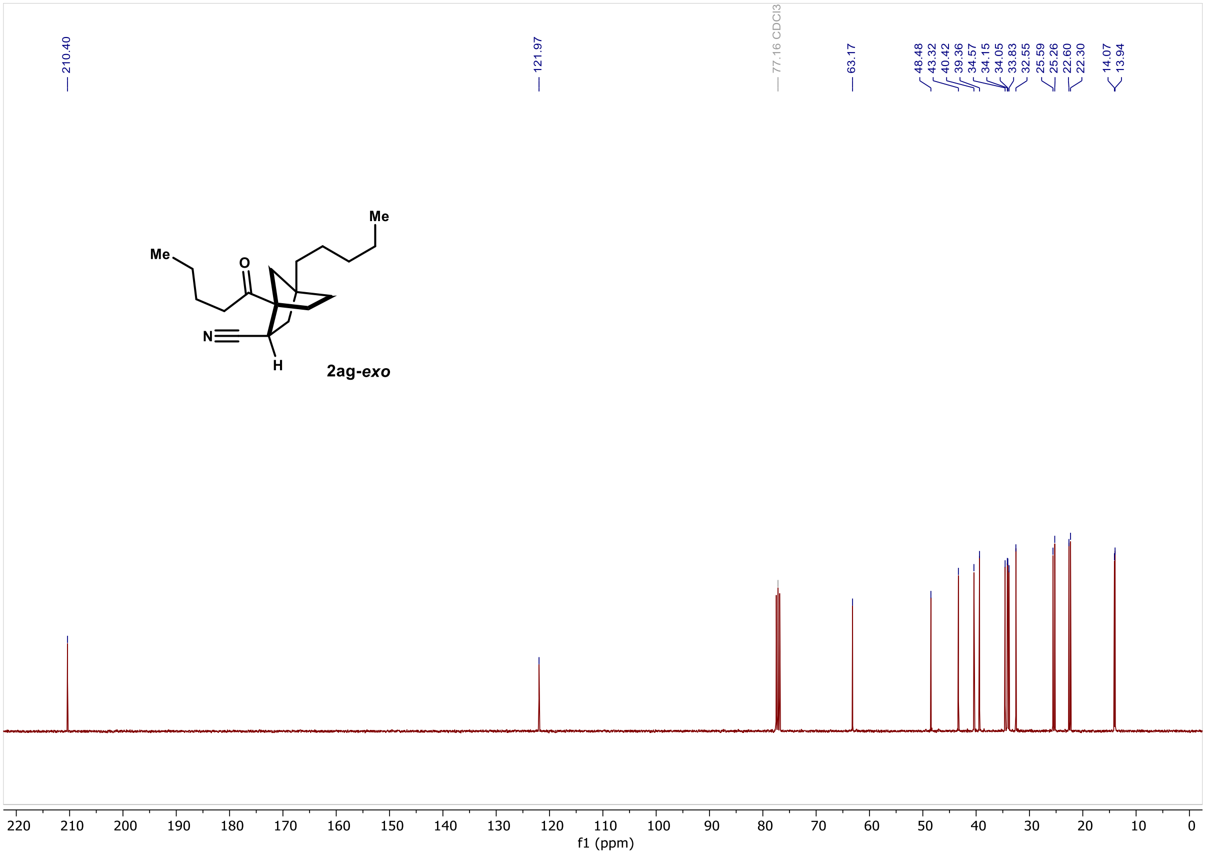

^{1}H NMR (400 MHz, Chloroform-*d*) of **2ah-*endo***:

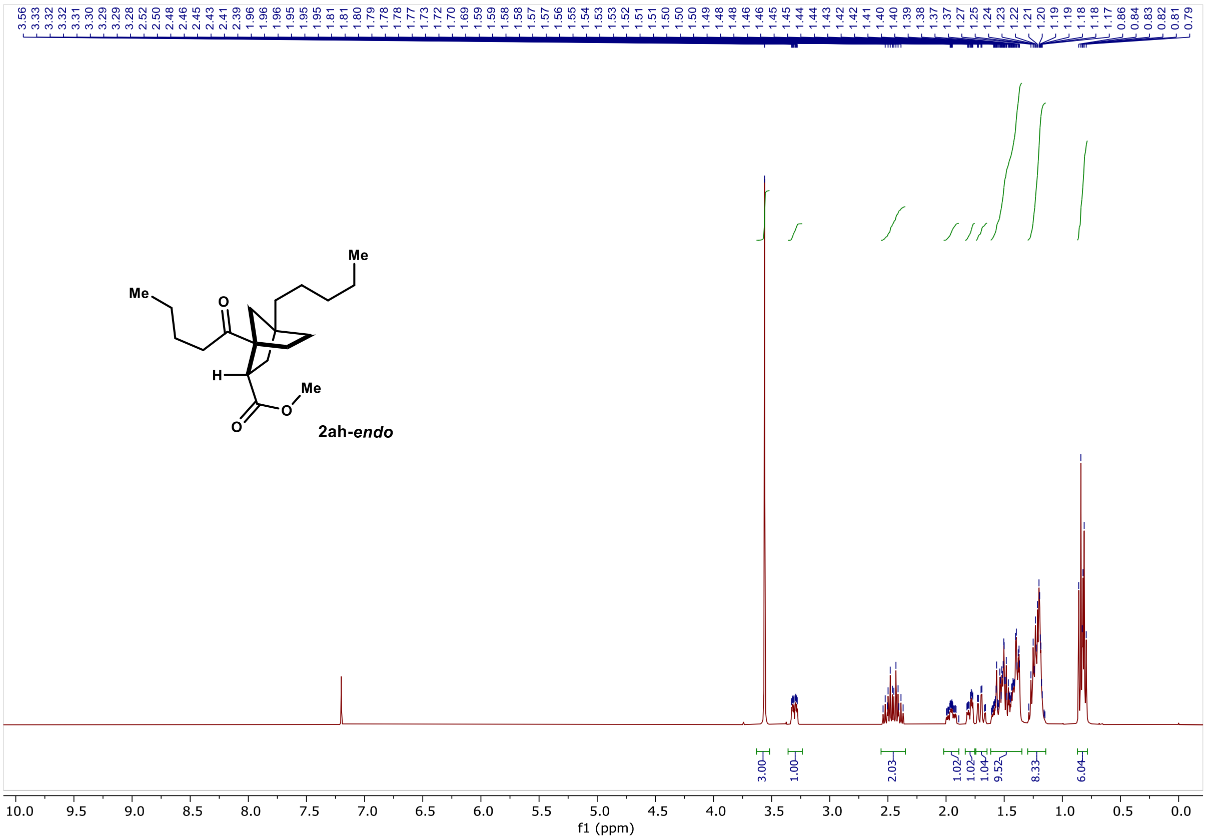

^{13}C NMR (101 MHz, Chloroform-*d*) of **2ah-*endo***:

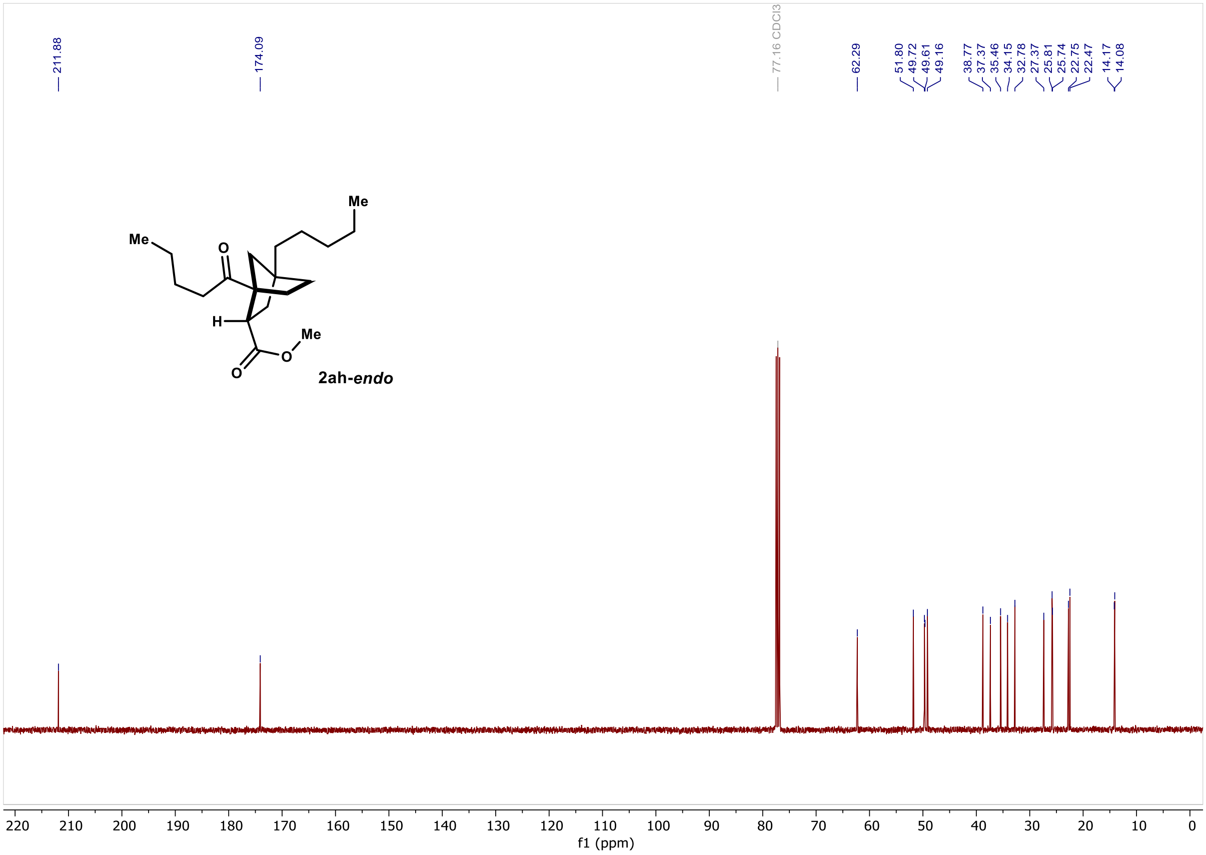

[1] H NMR (400 MHz, Chloroform-*d*) of **2ah-*exo***:

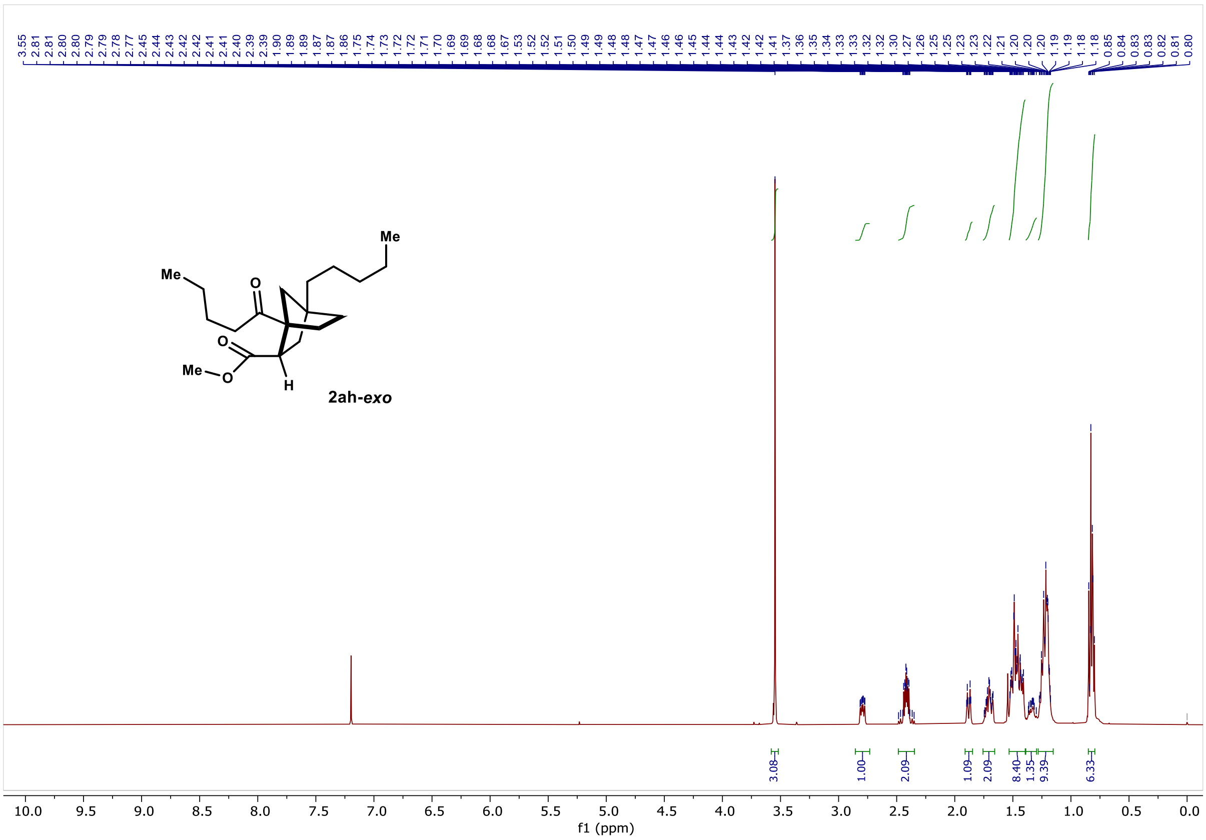

[13] C NMR (101 MHz, Chloroform-*d*) of **2ah-*exo***:

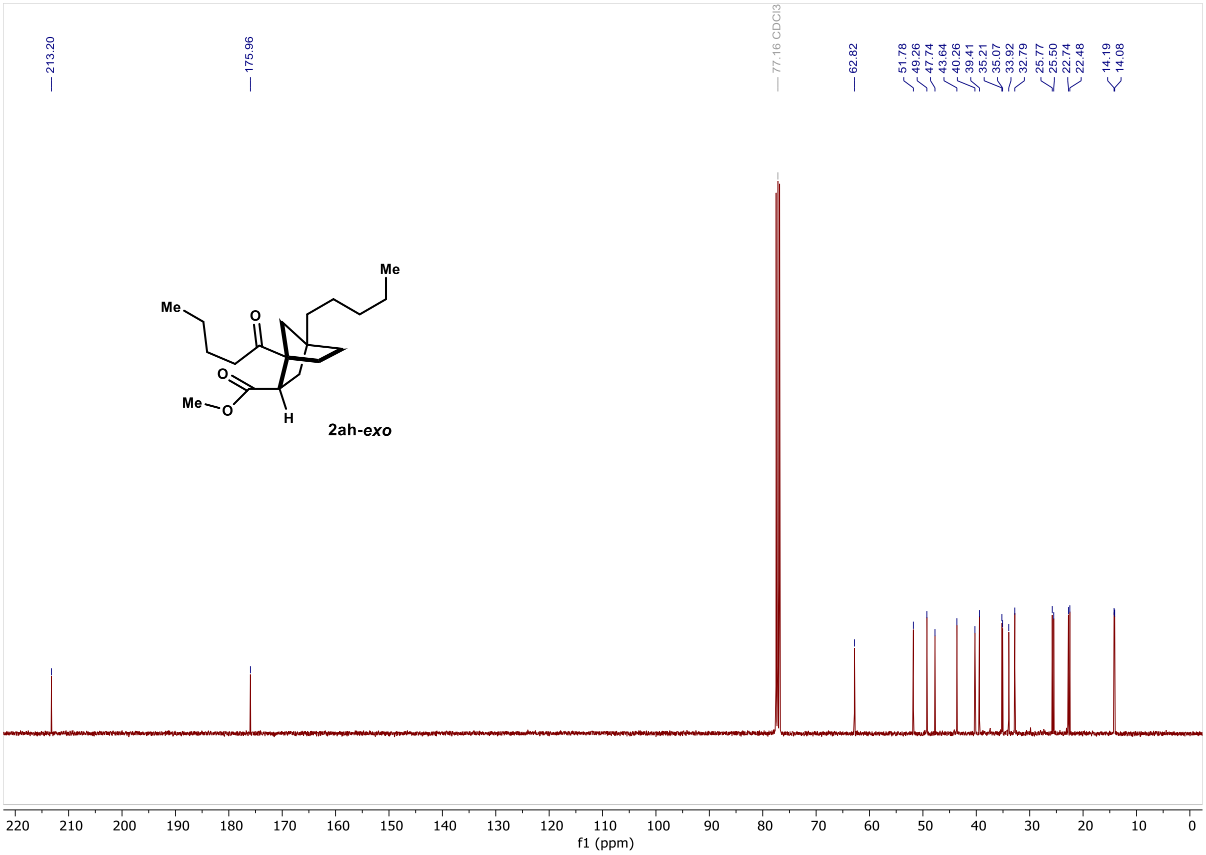

^{1}H NMR (400 MHz, Chloroform-*d*) of **2ai-*endo***:

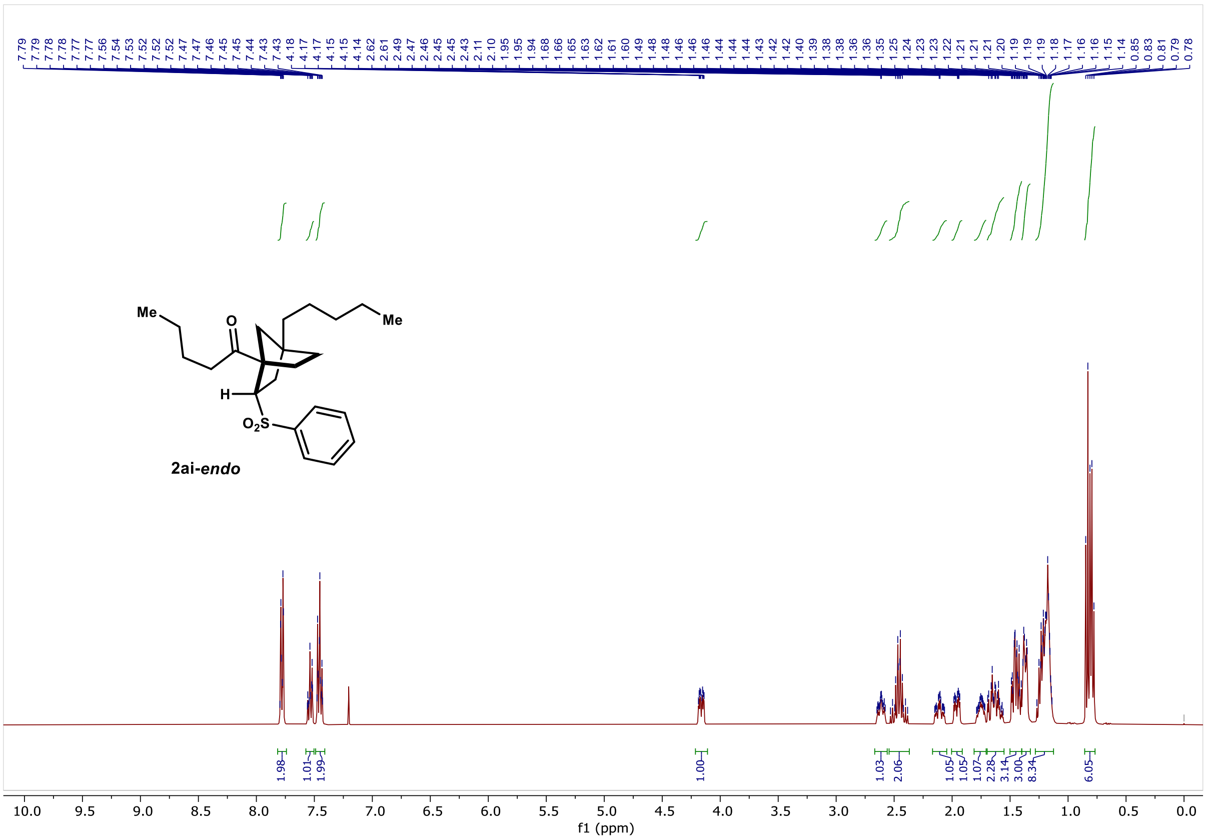

^{13}C NMR (101 MHz, Chloroform-*d*) of **2ai-*endo***:

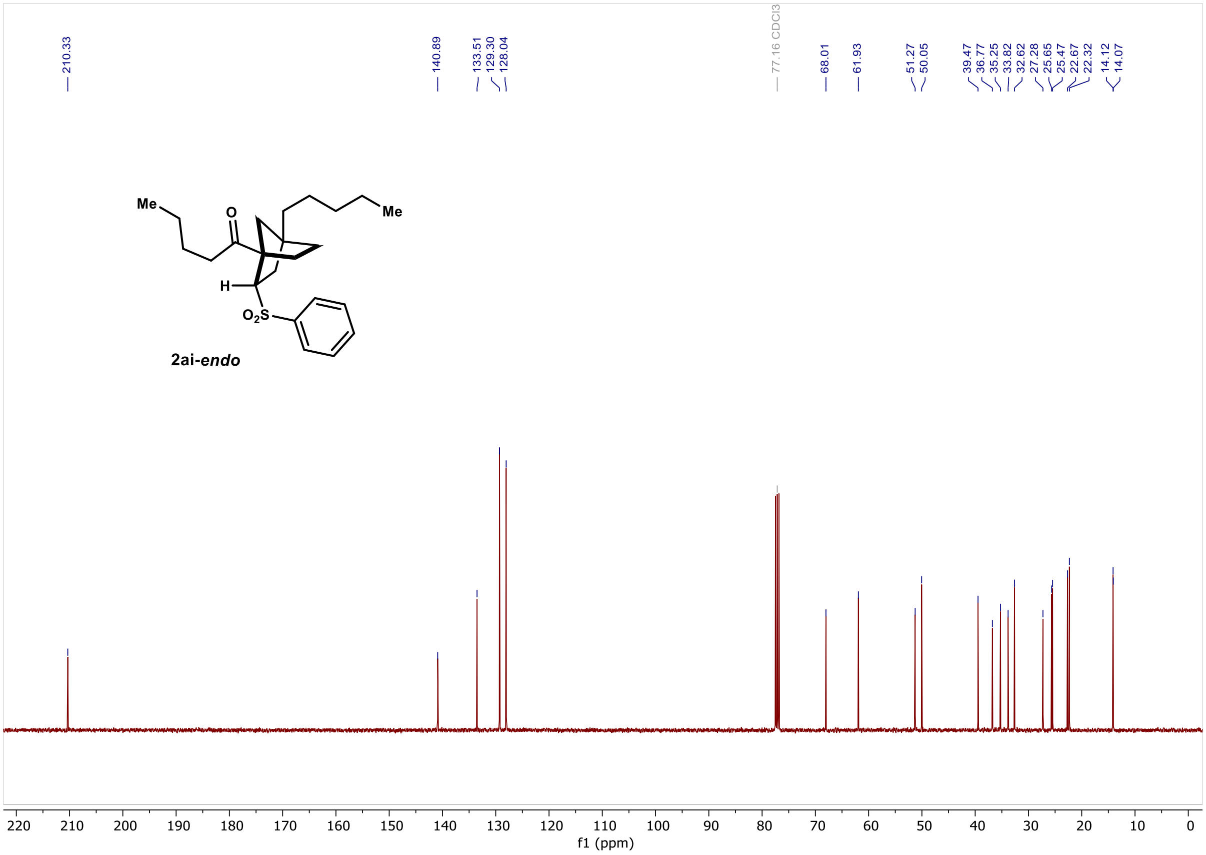

^{1}H NMR (400 MHz, Chloroform-*d*) of **2ai-*exo***:

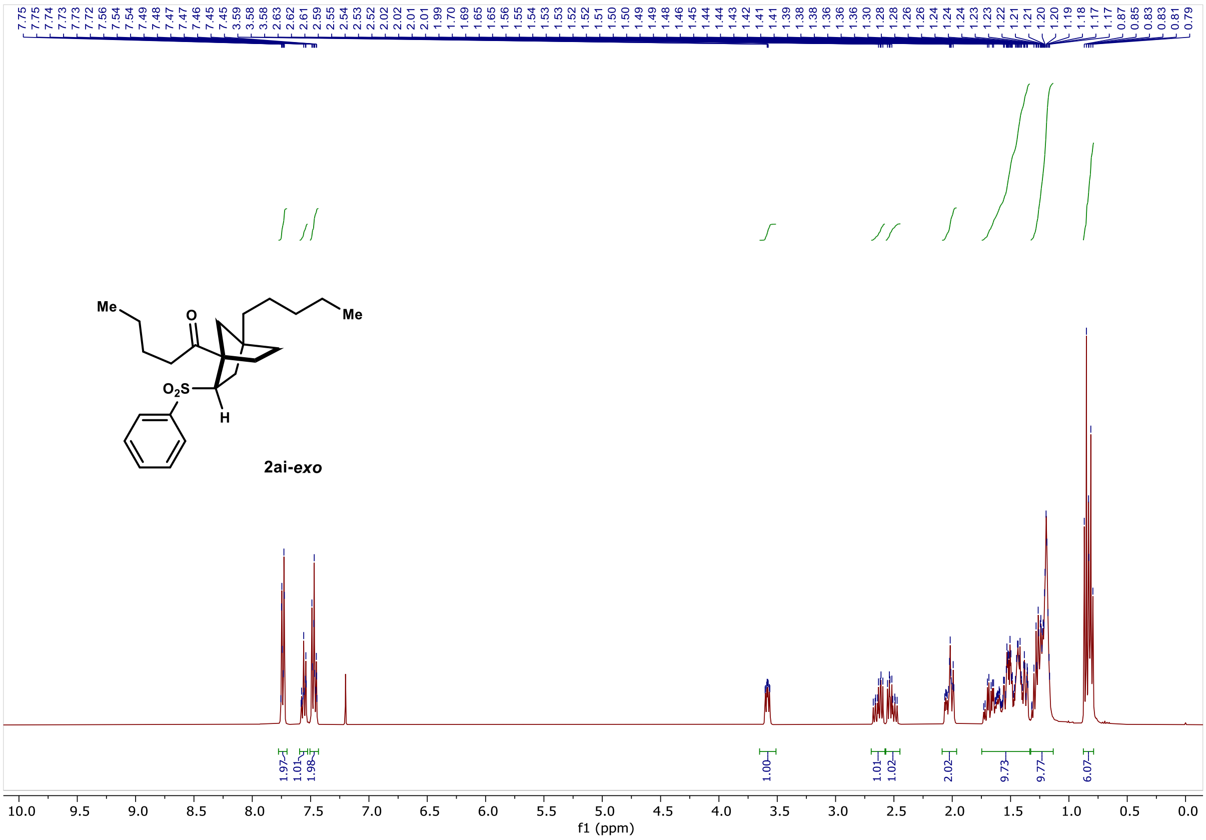

^{13}C NMR (101 MHz, Chloroform-*d*) of **2ai-*exo***:

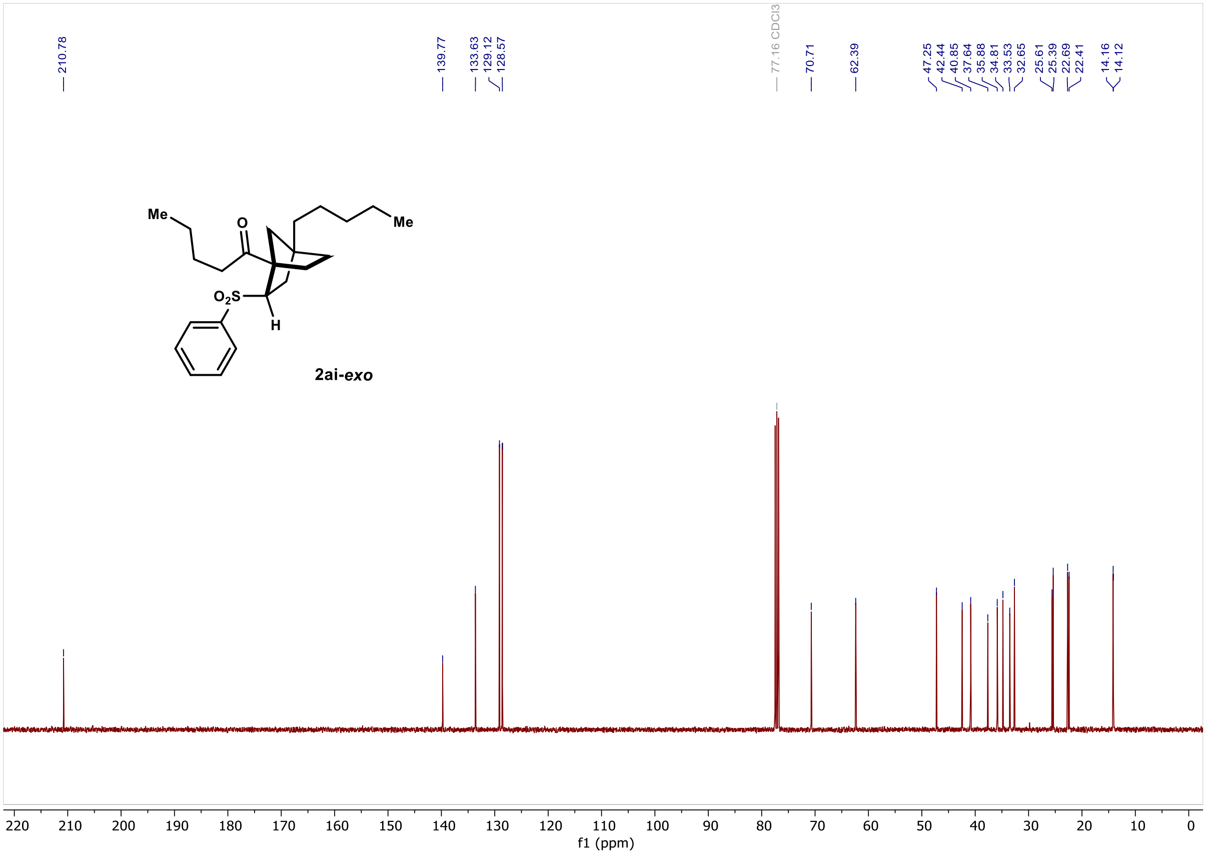

^{1}H NMR (400 MHz, Chloroform-*d*) of **2aj-*endo***:

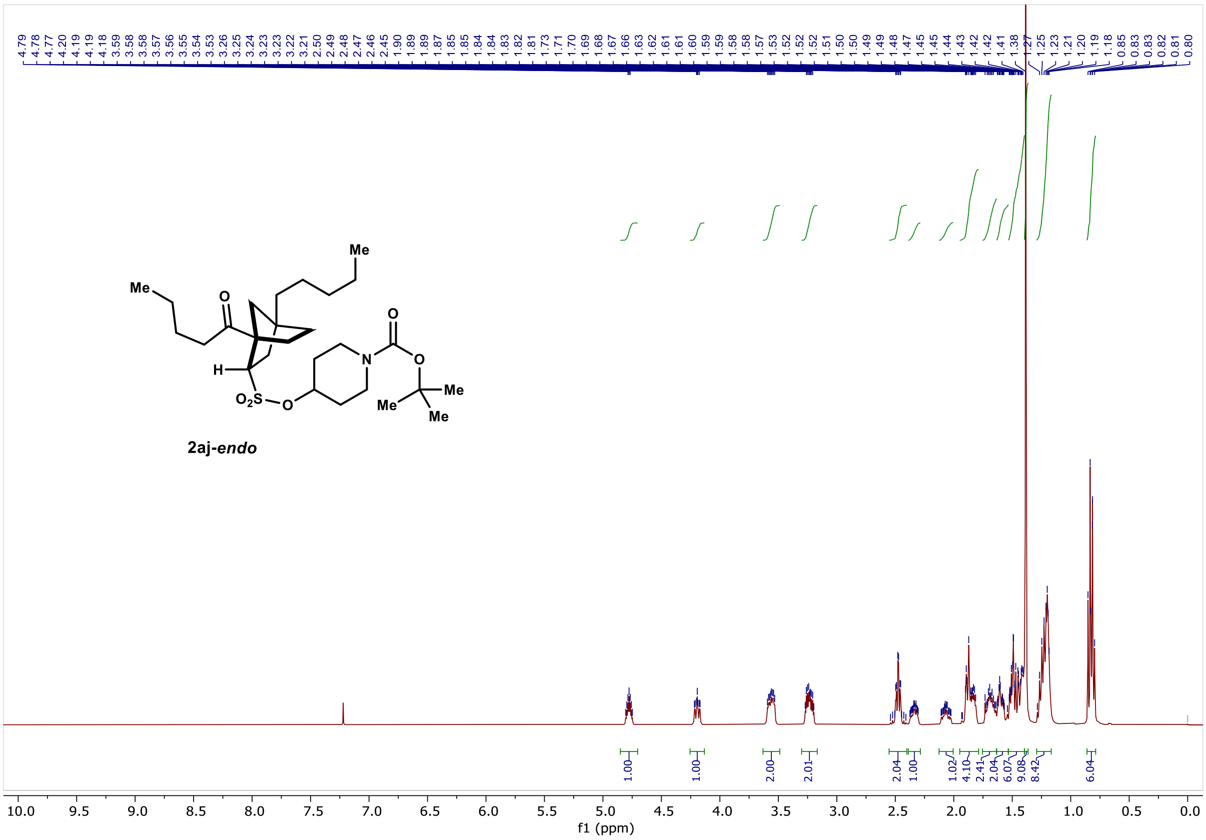

^{13}C NMR (101 MHz, Chloroform-*d*) of **2aj-*endo***:

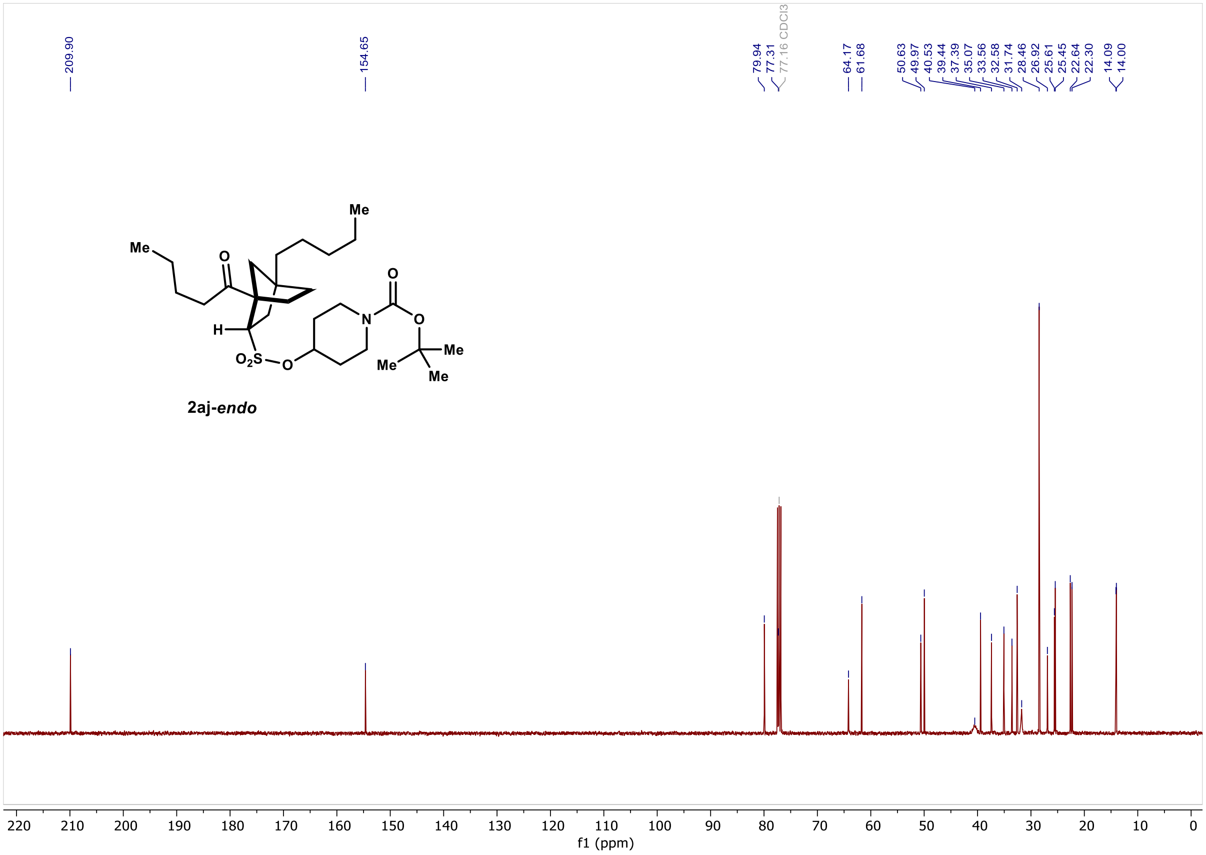

^{1}H NMR (400 MHz, Chloroform-*d*) of **2aj-*exo***:

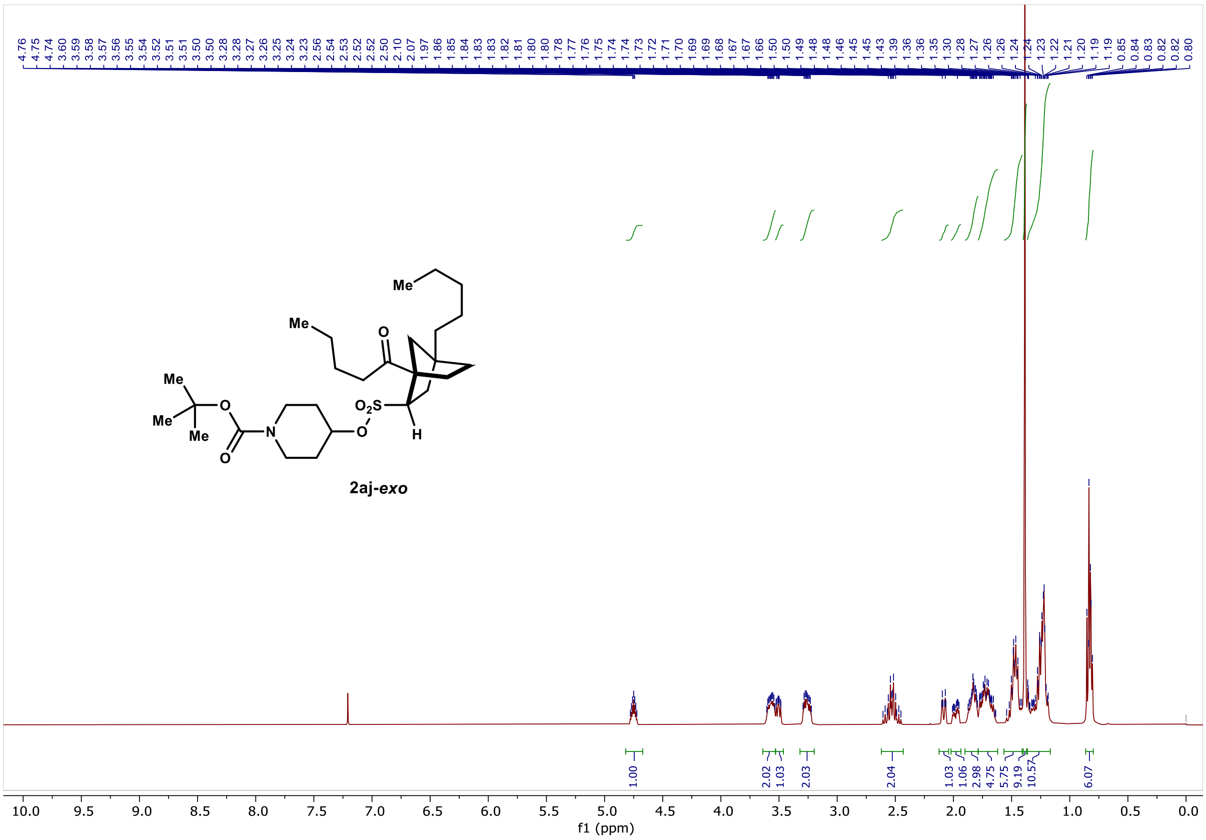

^{13}C NMR (101 MHz, Chloroform-*d*) of **2aj-*exo***:

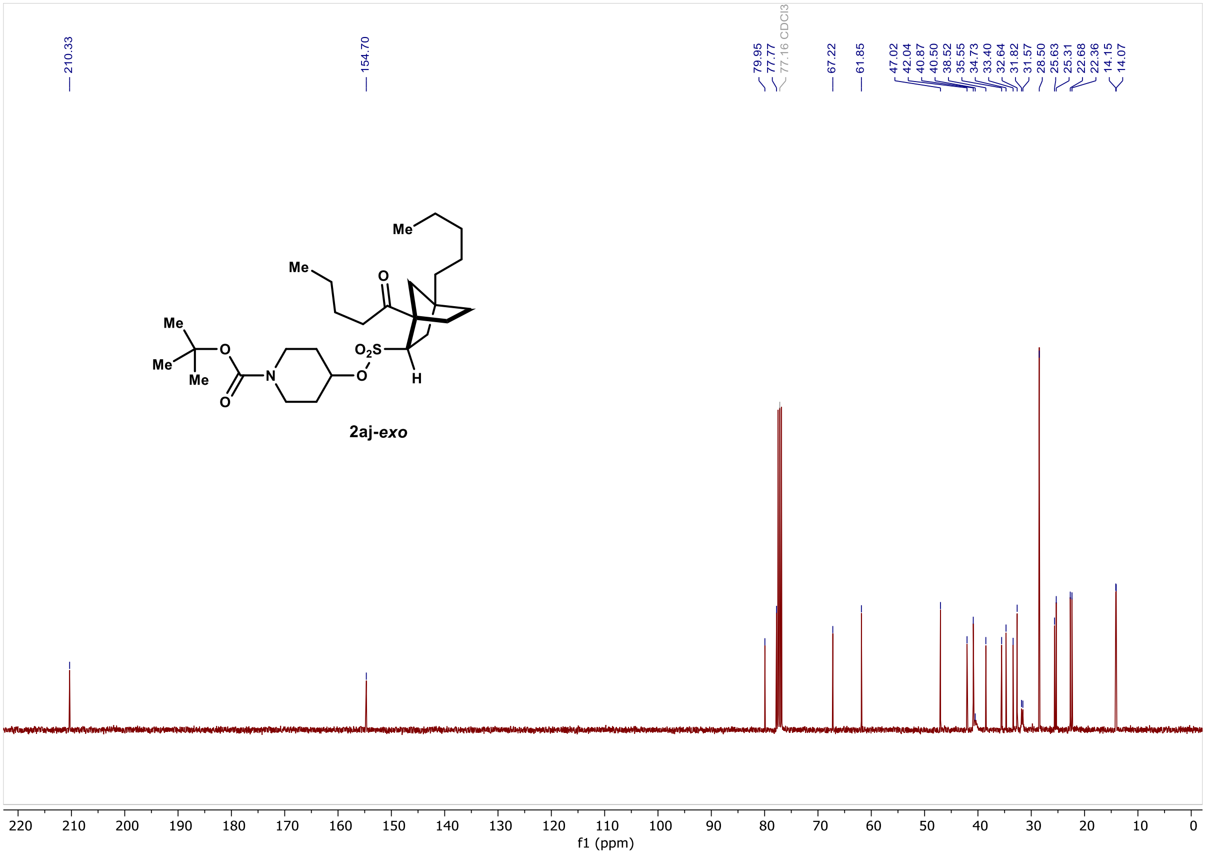

^{1}H NMR (400 MHz, Chloroform-*d*) of **4**:

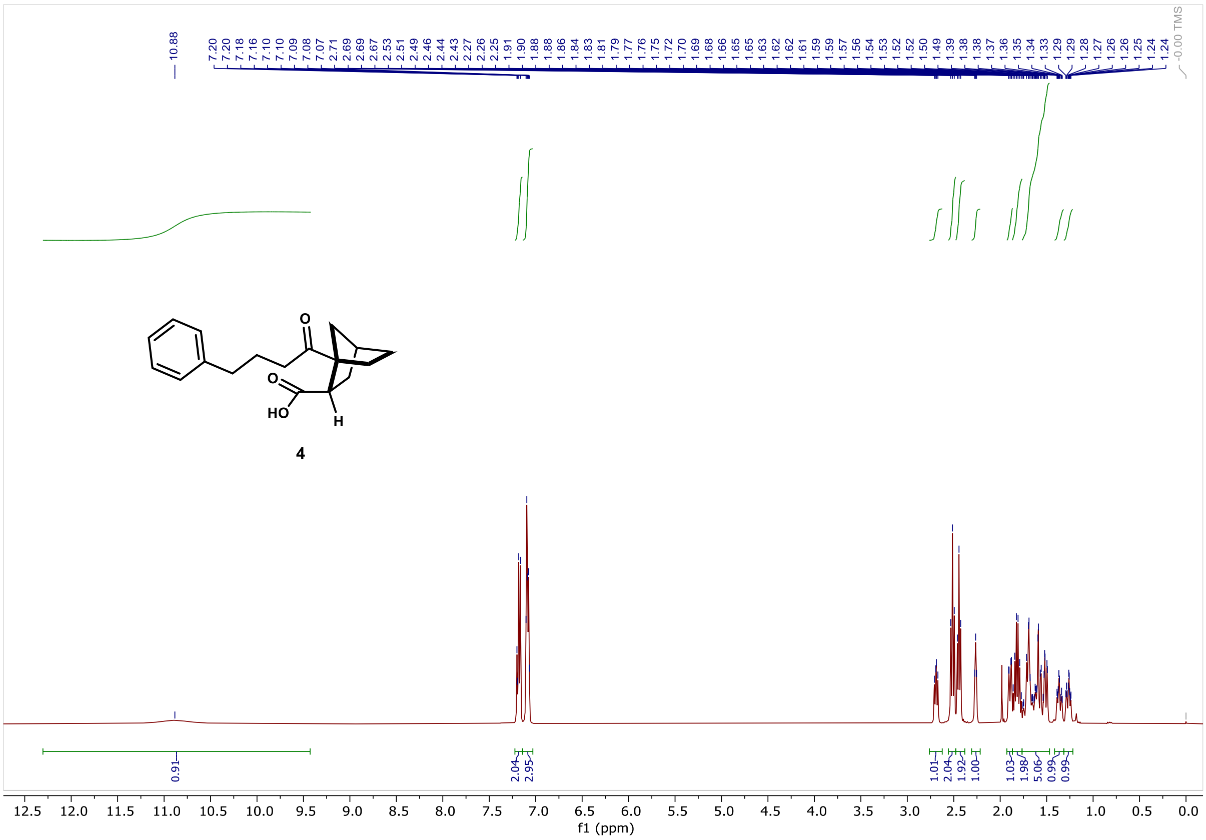

^{13}C NMR (101 MHz, Chloroform-*d*) of **4**:

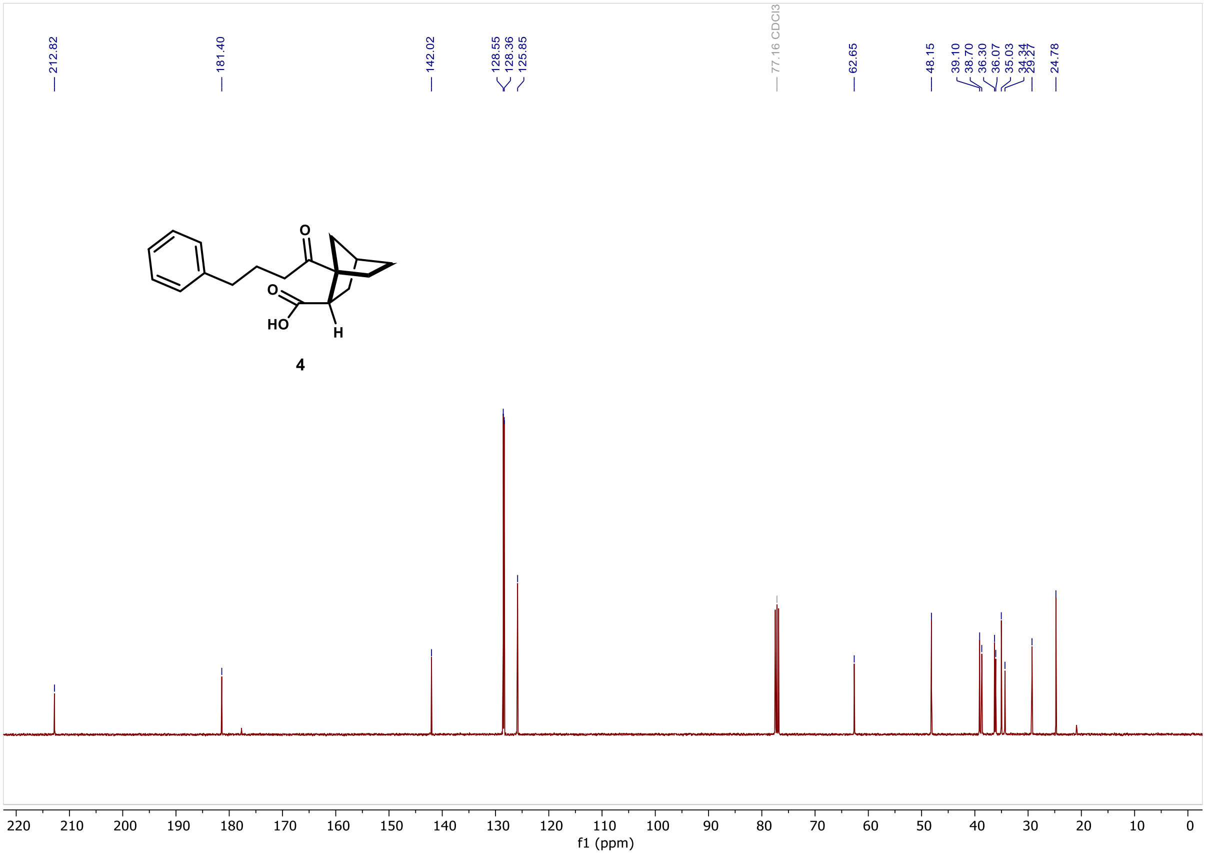

^{1}H NMR (400 MHz, Chloroform-*d*) of **2s-*exo***:

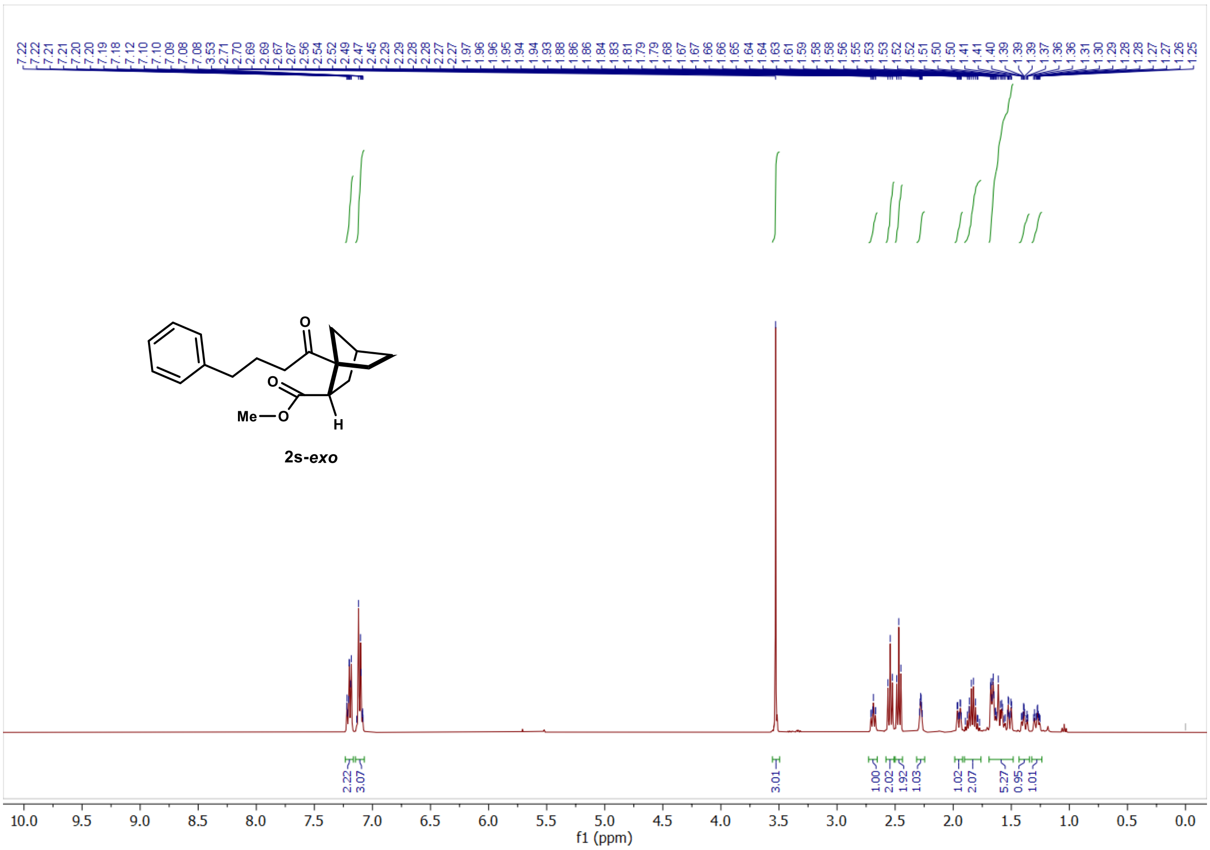

^{13}C NMR (101 MHz, Chloroform-*d*) of **2s-*exo***:

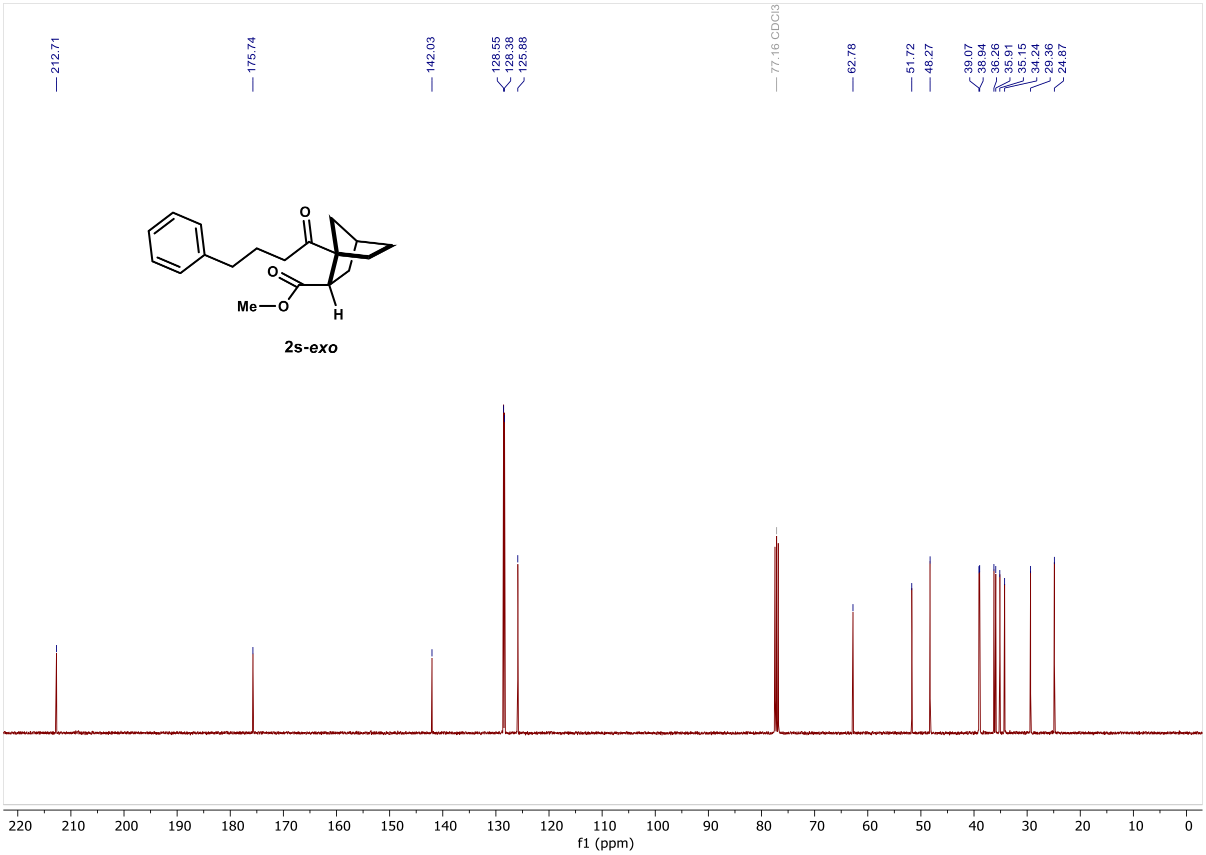

^{1}H NMR (400 MHz, Chloroform-*d*) of **5**:

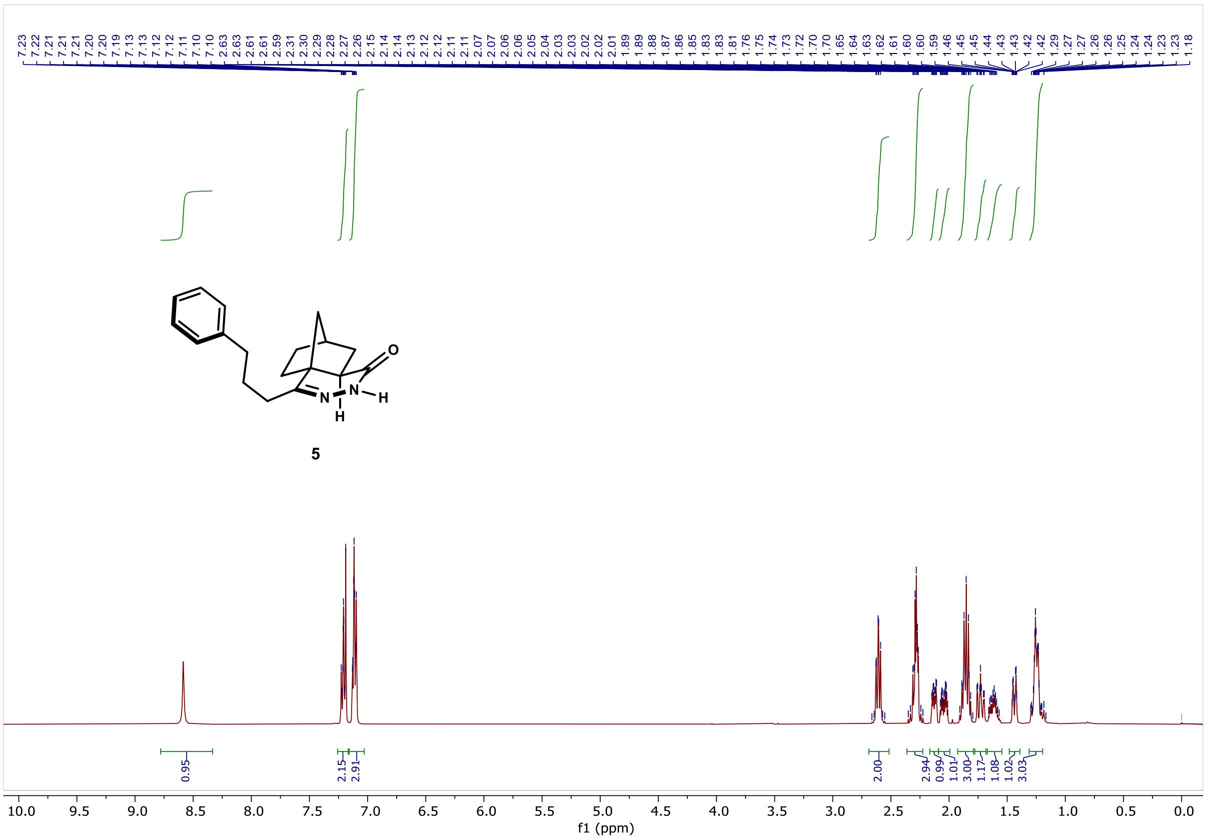

^{13}C NMR (126 MHz, Chloroform-*d*) of **5**:

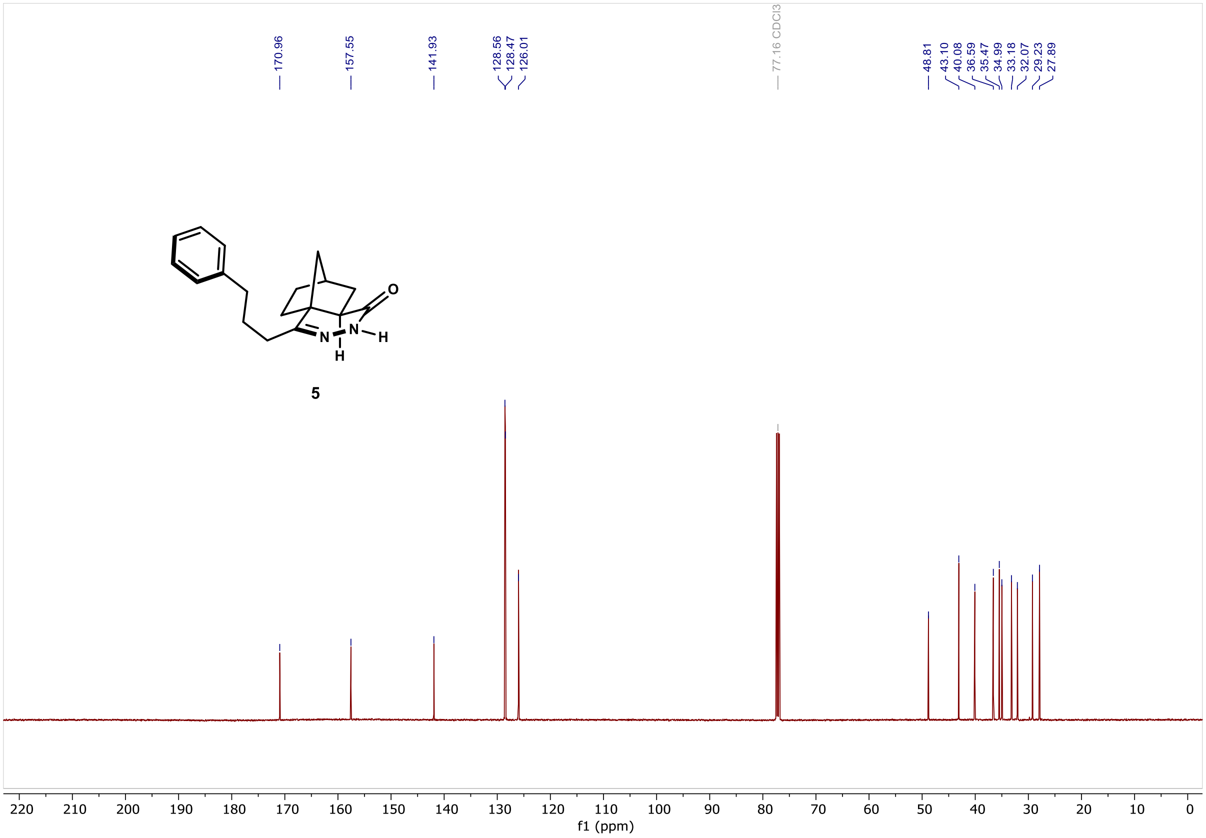

^{1}H NMR (400 MHz, Chloroform-*d*) of **6**:

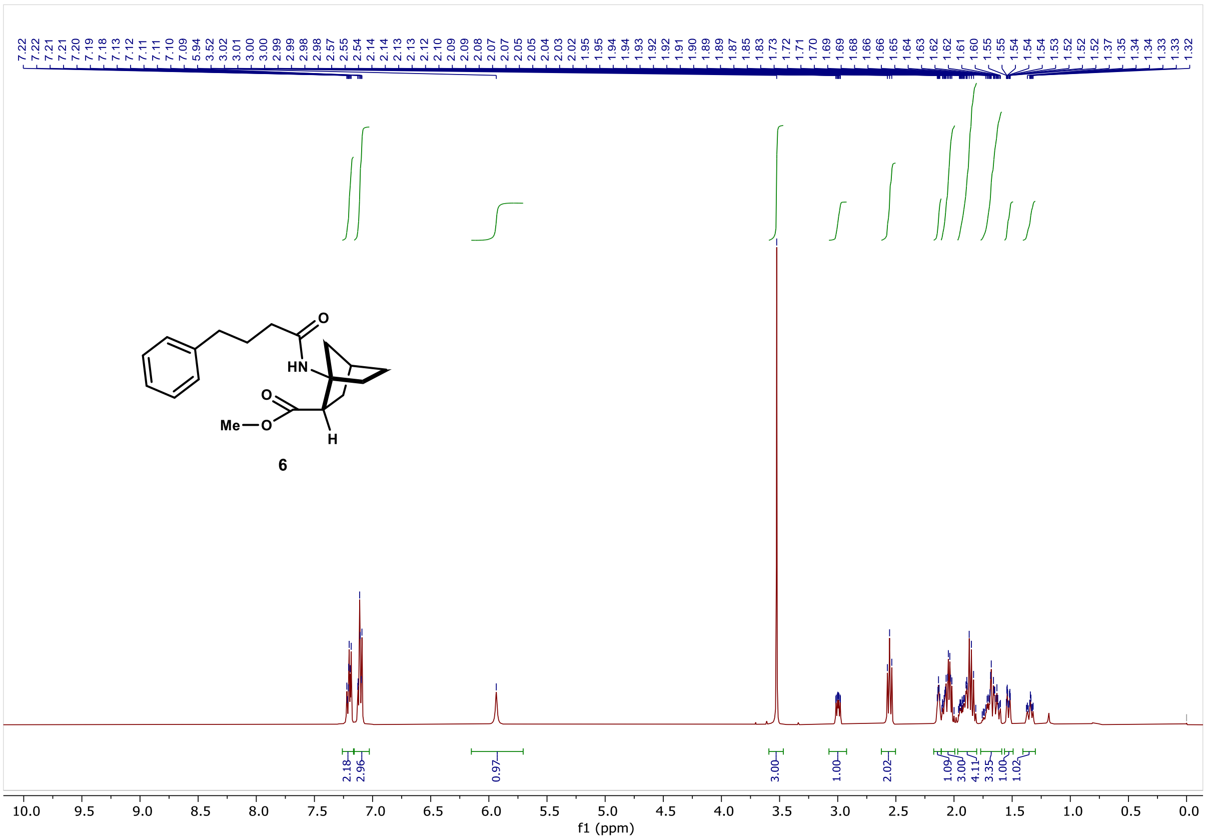

^{13}C NMR (101 MHz, Chloroform-*d*) of **6**:

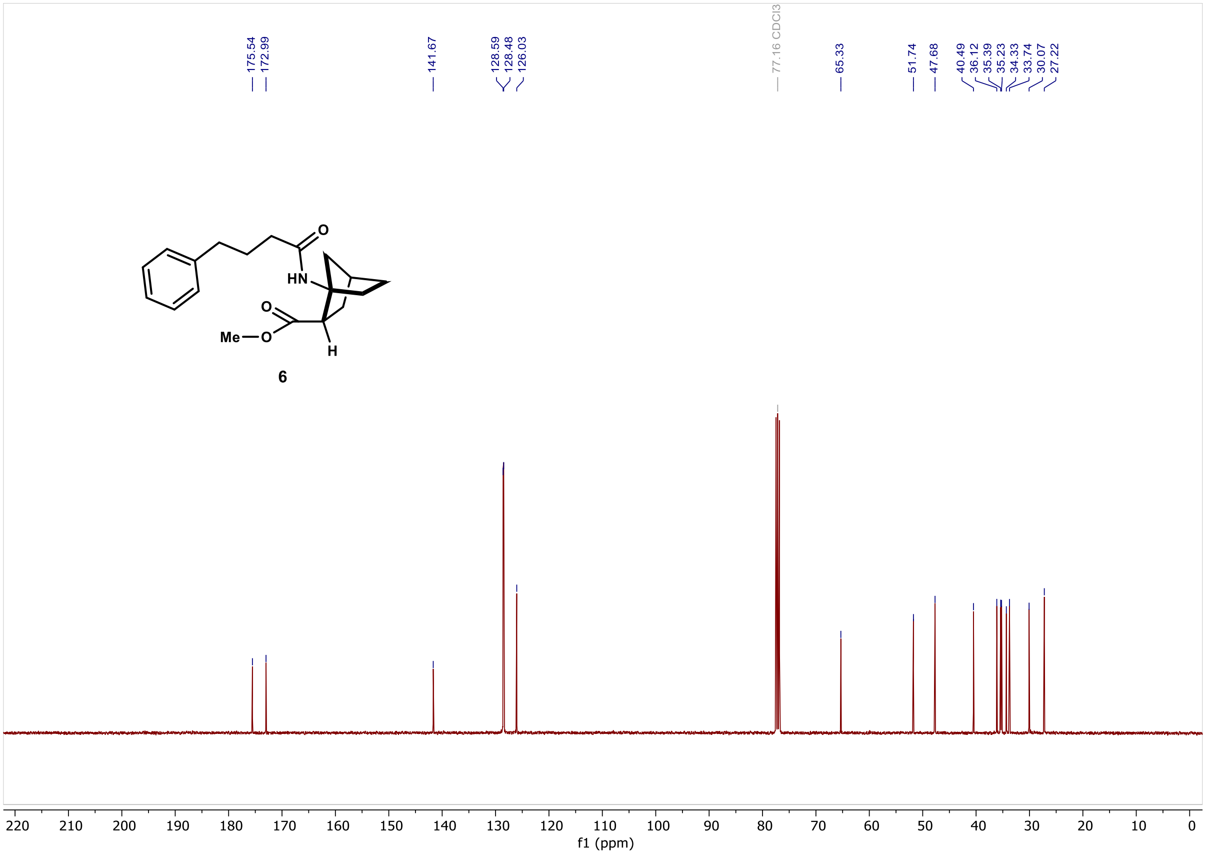

^{1}H NMR (400 MHz, Chloroform-*d*) of **7**:

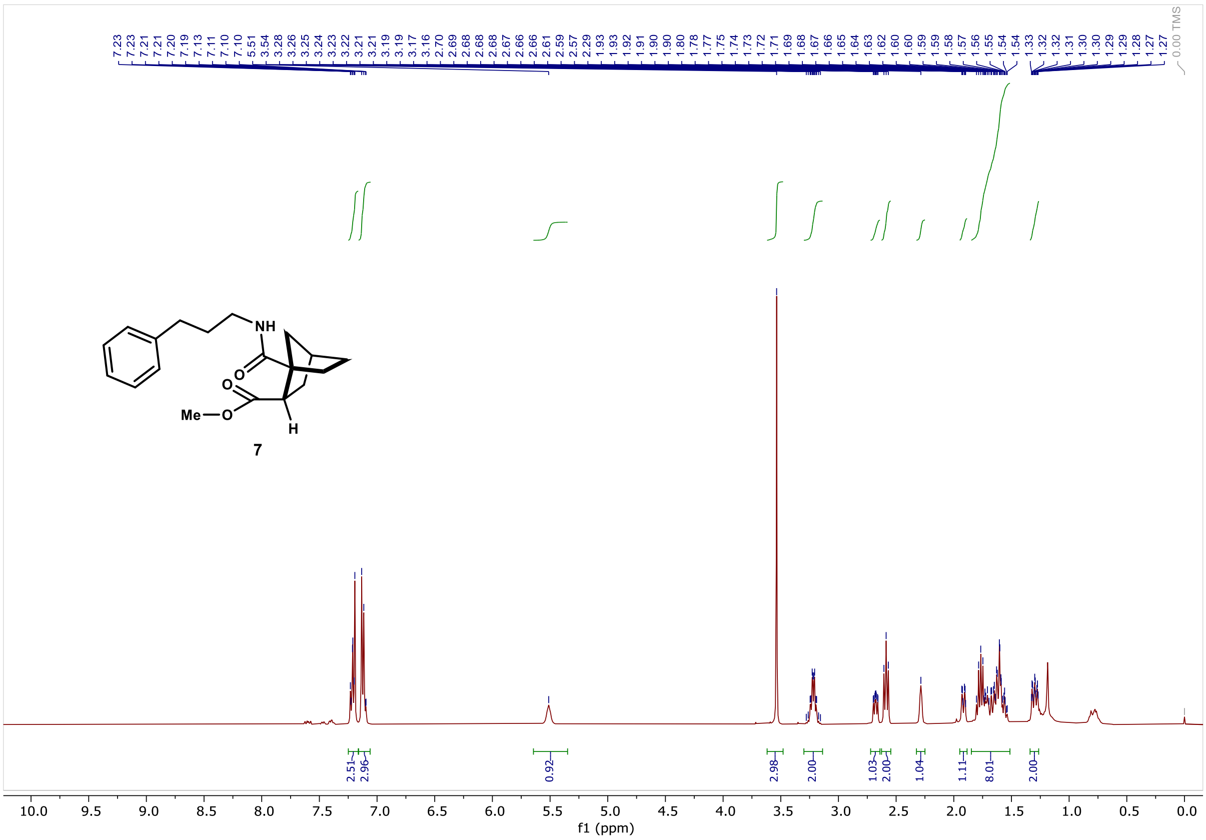

^{13}C NMR (101 MHz, Chloroform-*d*) of **7**:

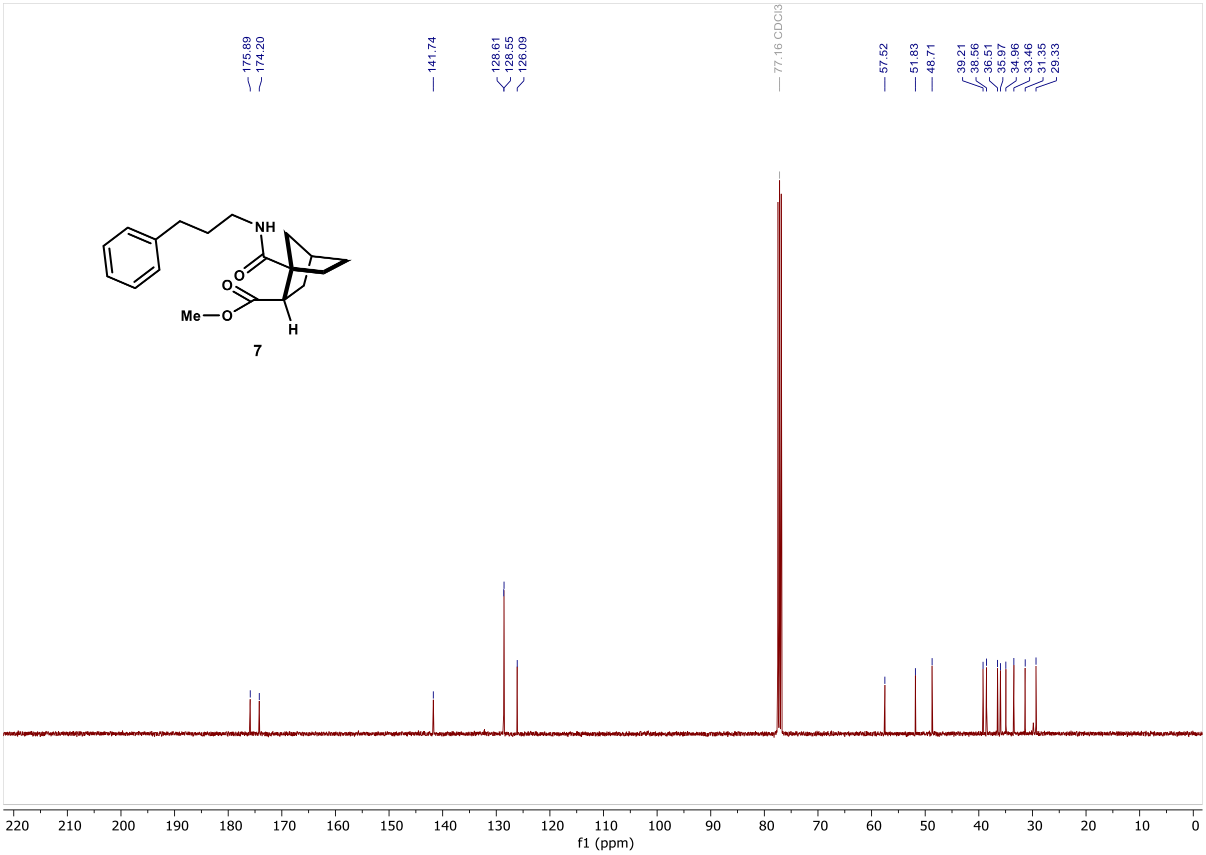

^{1}H NMR (500 MHz, Chloroform-*d*) of **8**:

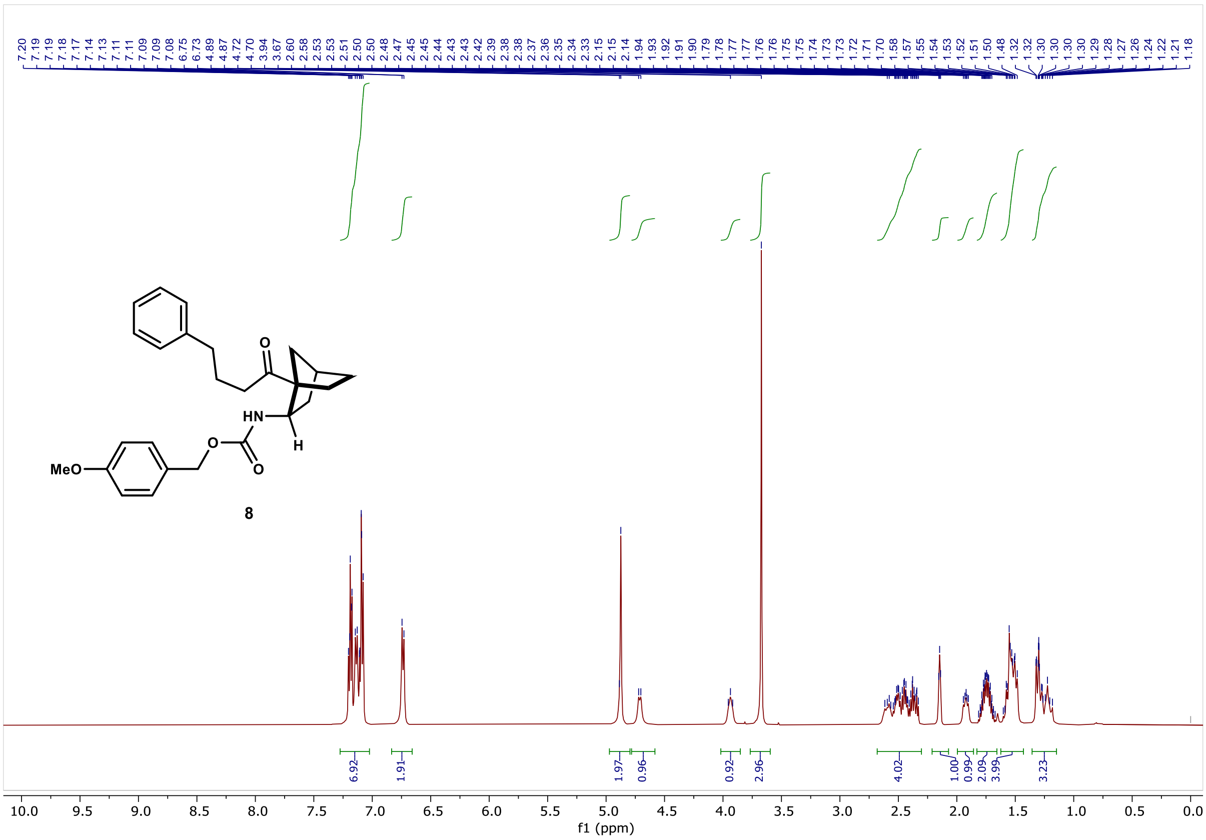

^{13}C NMR (126 MHz, Chloroform-*d*) of **8**:

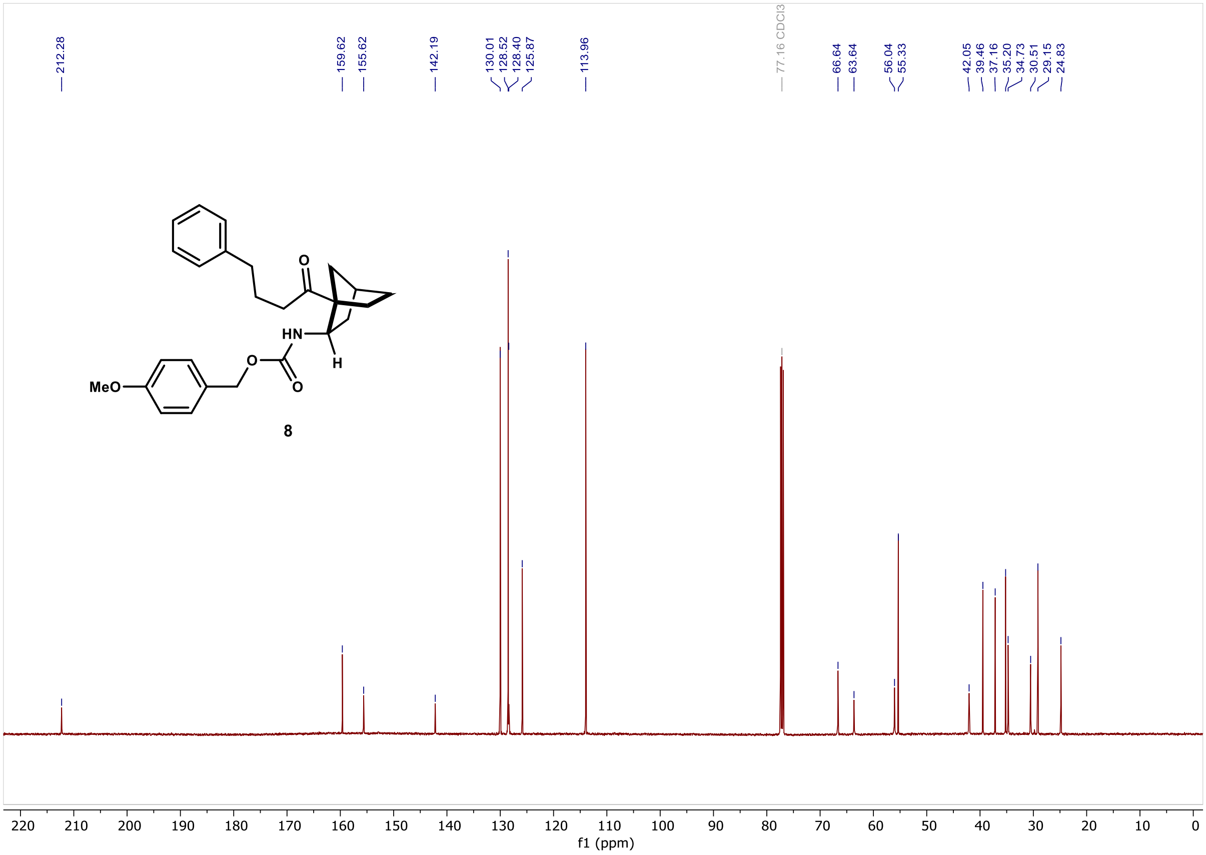

^{1}H NMR (400 MHz, Chloroform-d) of **9**:

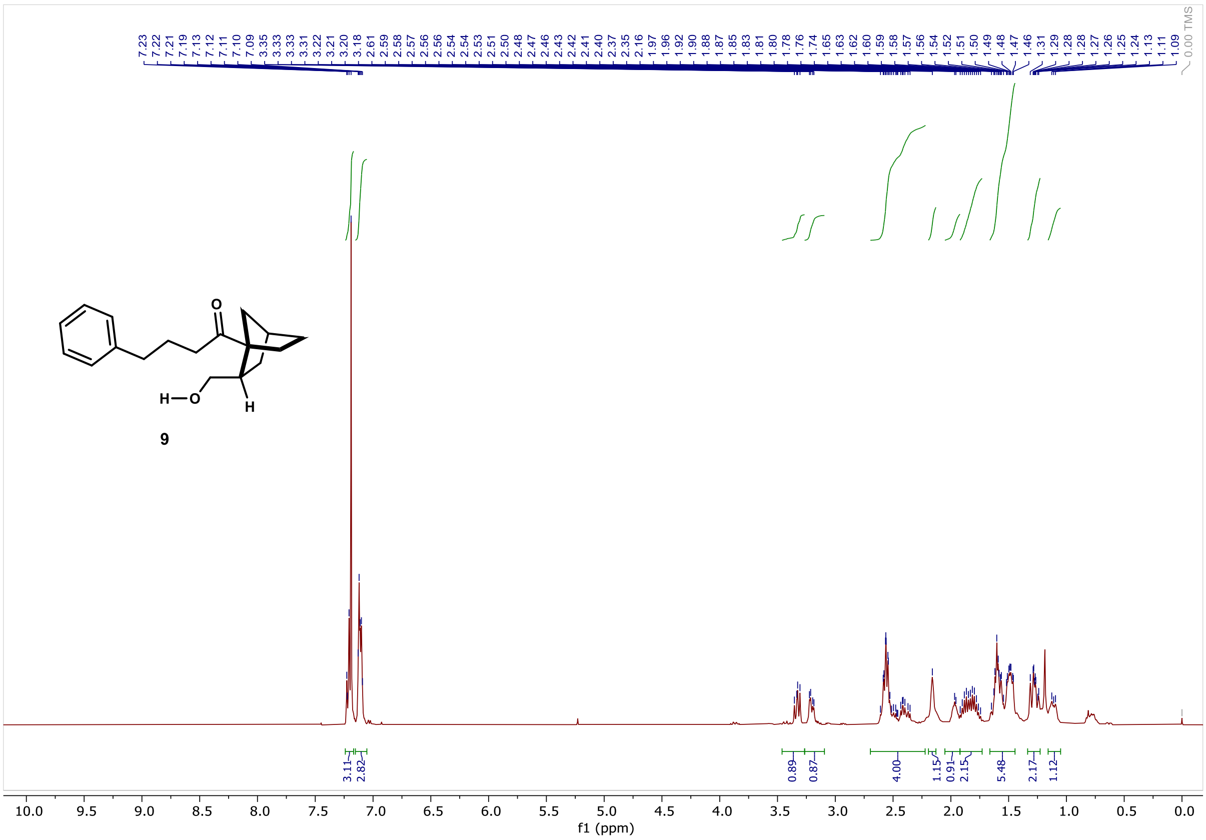

^{13}C NMR (101 MHz, Chloroform-d) of **9**:

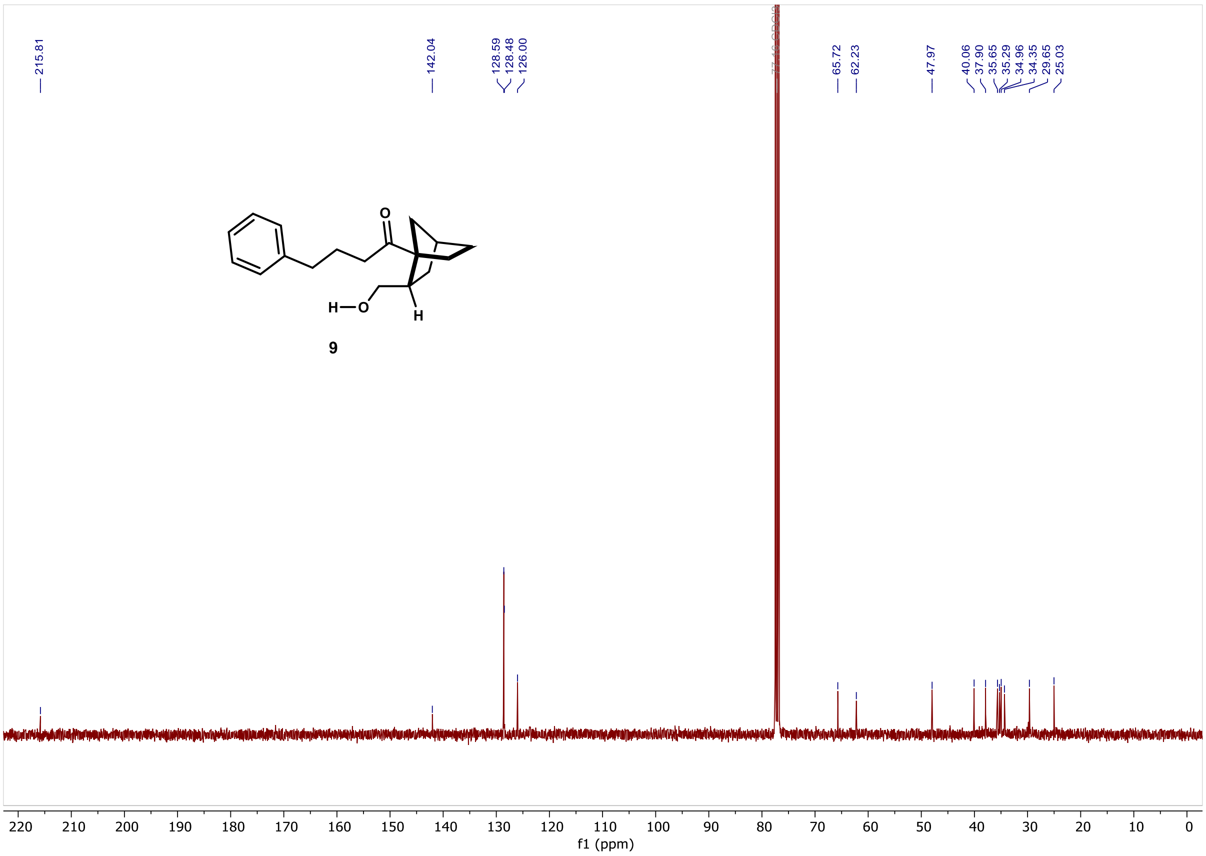

^{1}H NMR (500 MHz, Benzene-*d6*) of **10-*exo* and 10-*endo***:

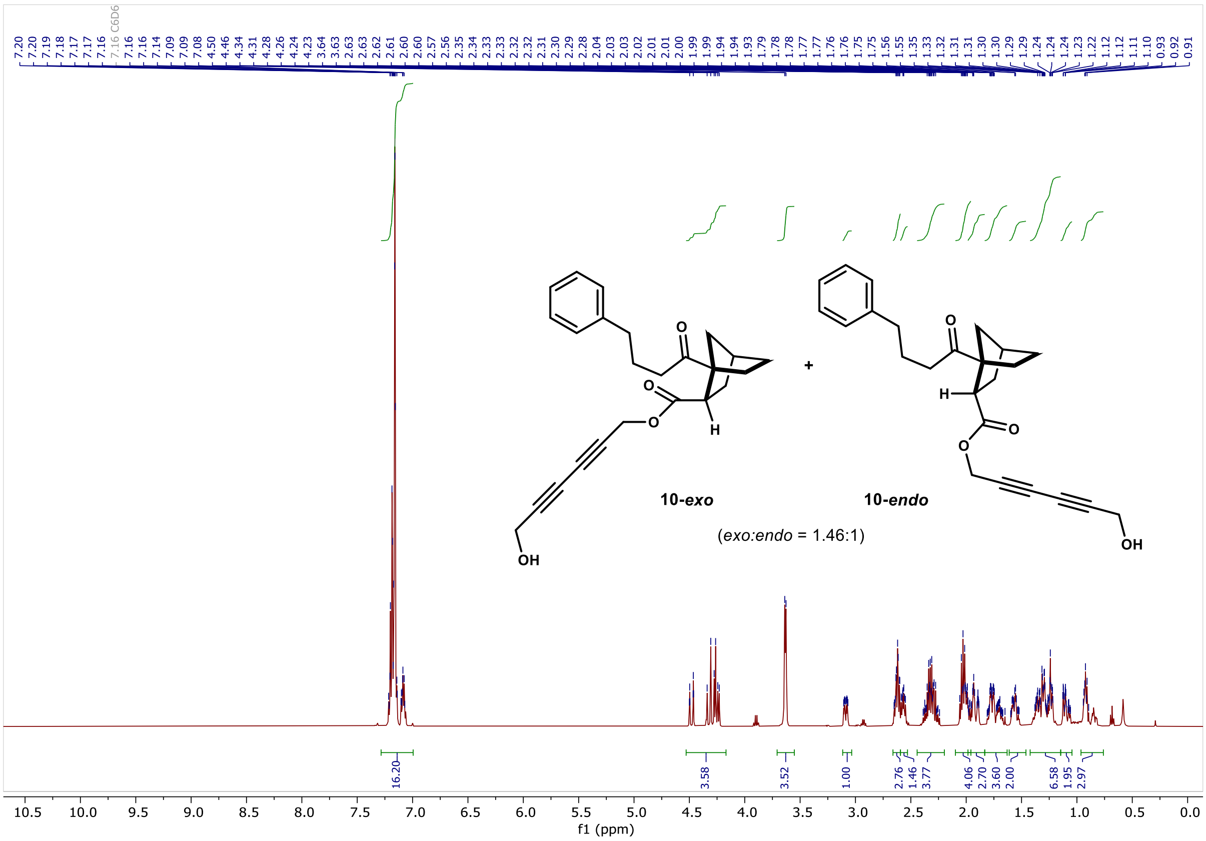

^{13}C NMR (126 MHz, Benzene-*d6*) of **10-*exo* and 10-*endo***:

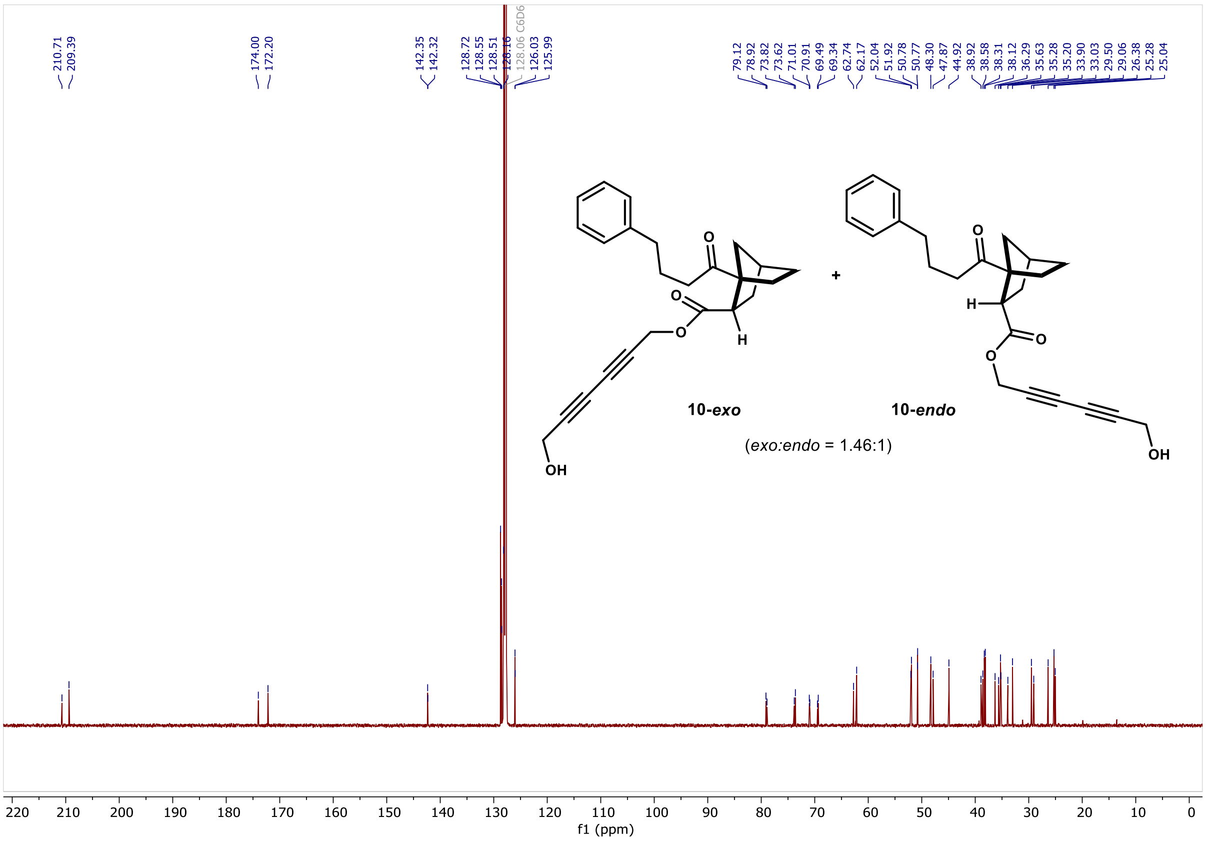

^{1}H NMR (500 MHz, Acetonitrile-*d3*) of **11**:

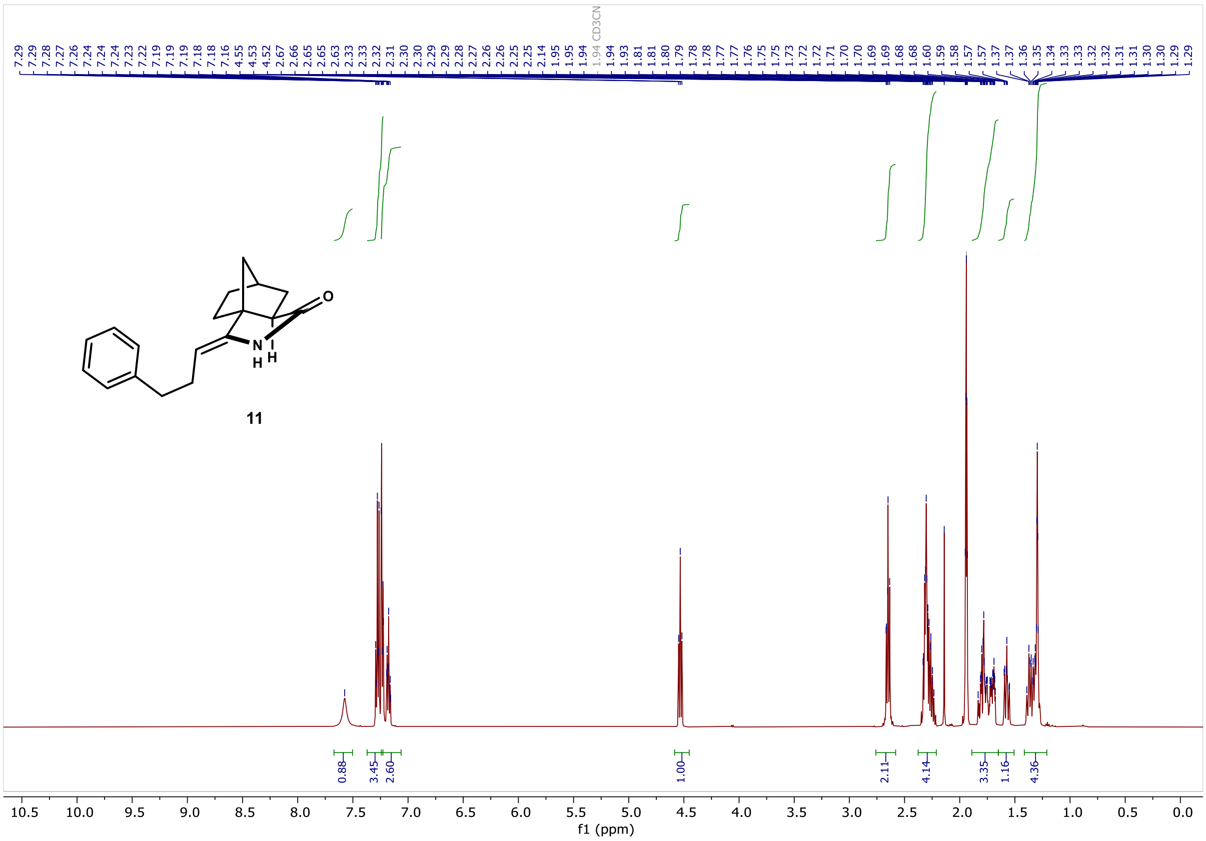

^{13}C NMR (126 MHz, Acetonitrile-*d3*) of **11**:

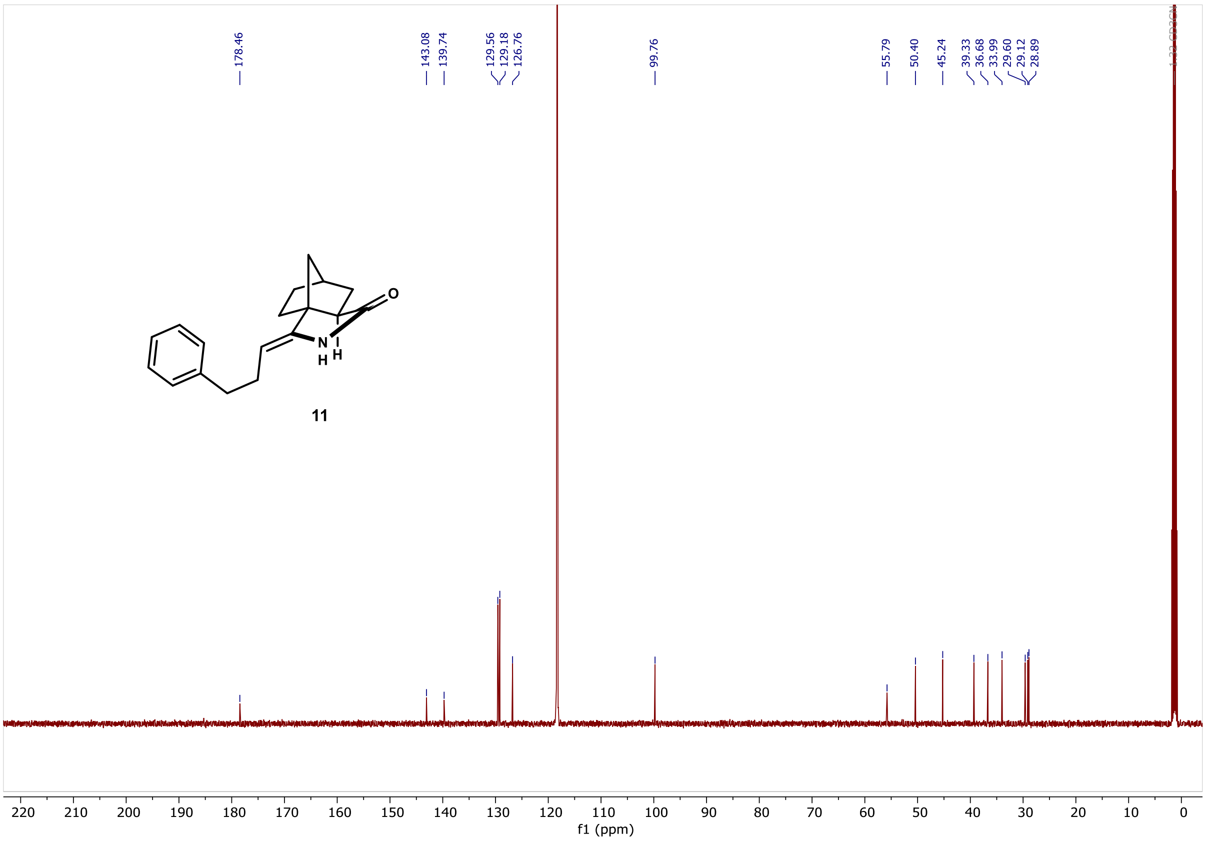

^{1}H NMR (400 MHz, Benzene-*d6*) of **12-*exo* and 12-*endo***:

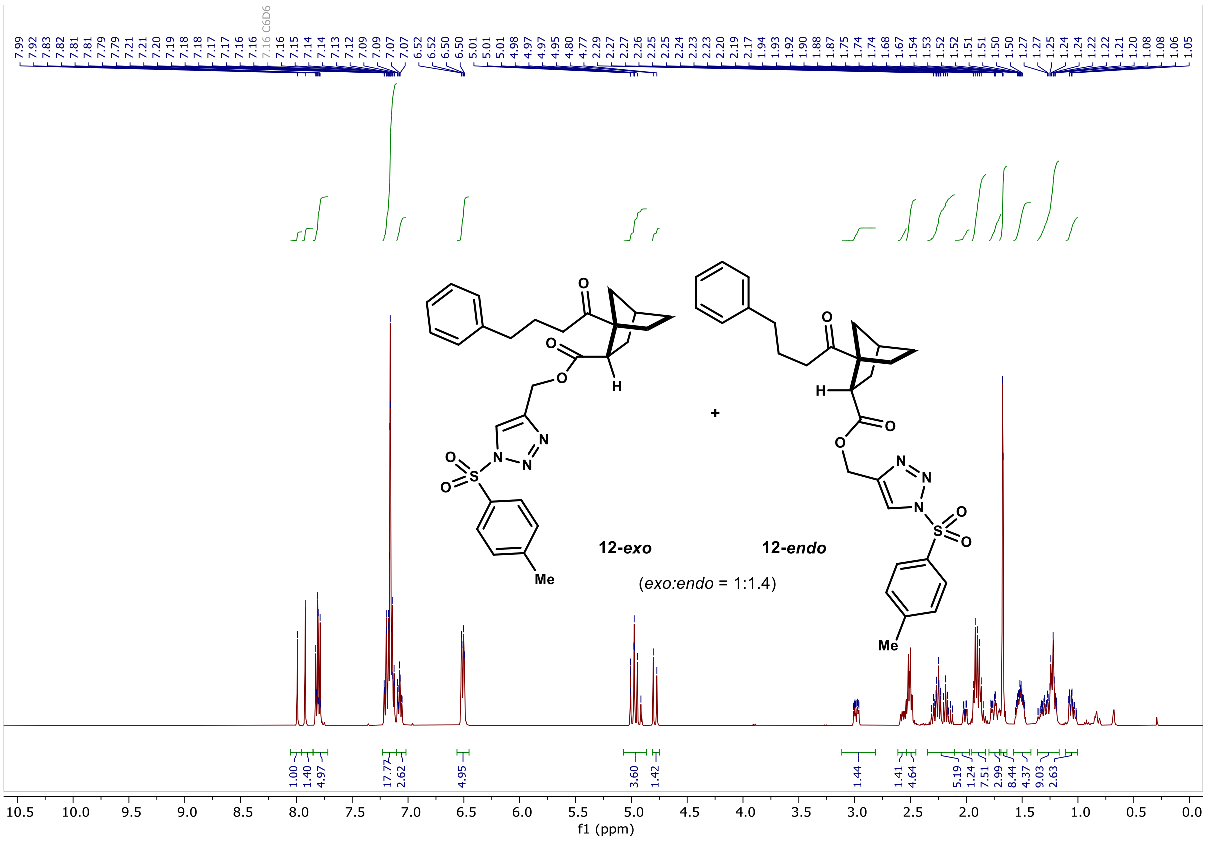

^{13}C NMR (126 MHz, Benzene-*d6*) of **12-*exo* and 12-*endo***:

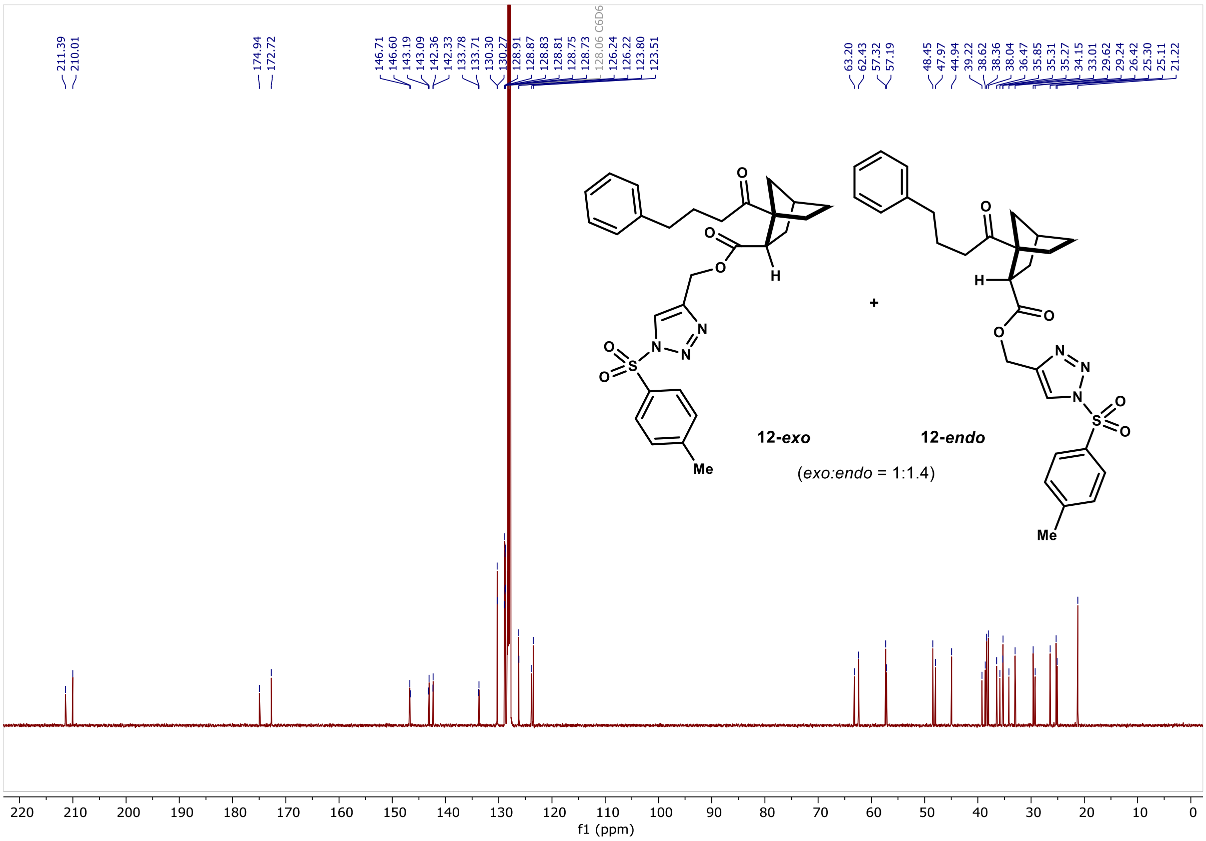

^{1}H NMR (500 MHz, Chloroform-*d*) of **SI-43**:

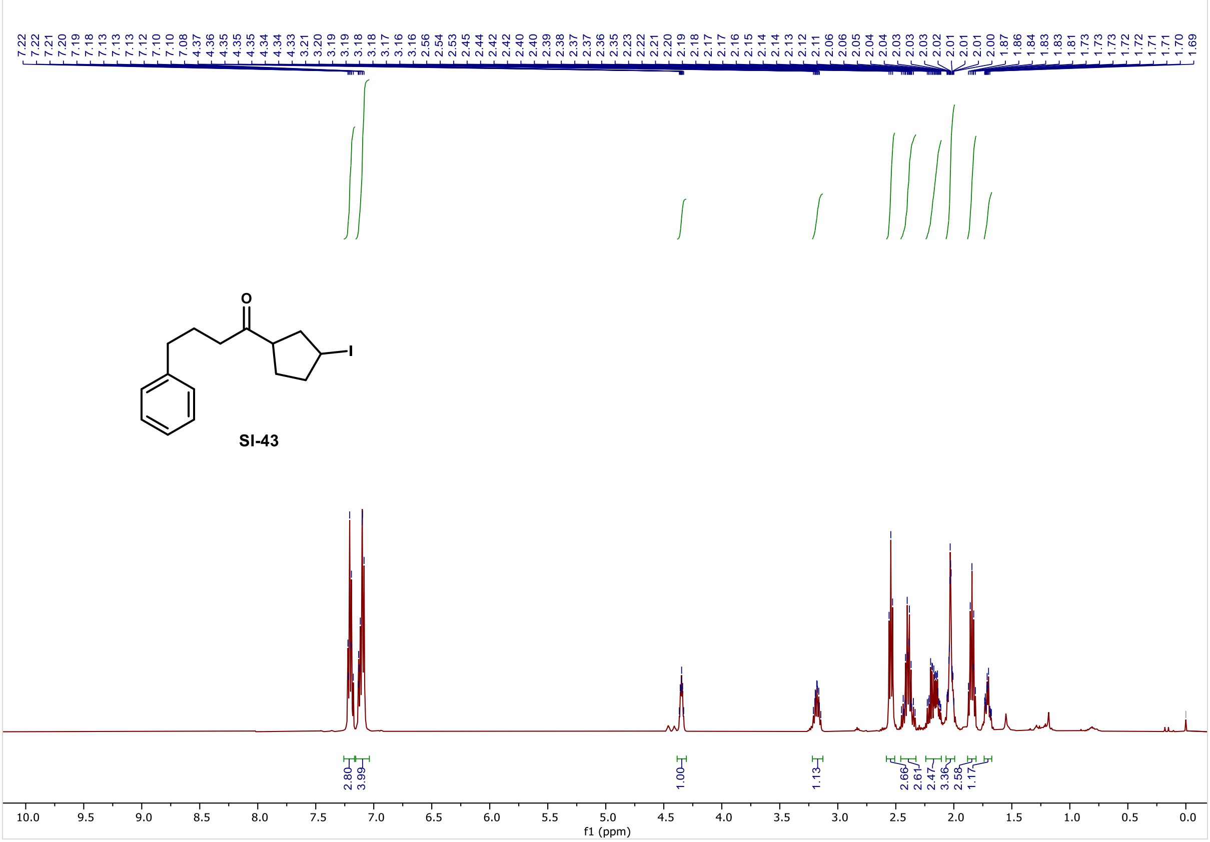

6

^{13}C NMR (126 MHz, Chloroform-*d*) of **SI-43**:

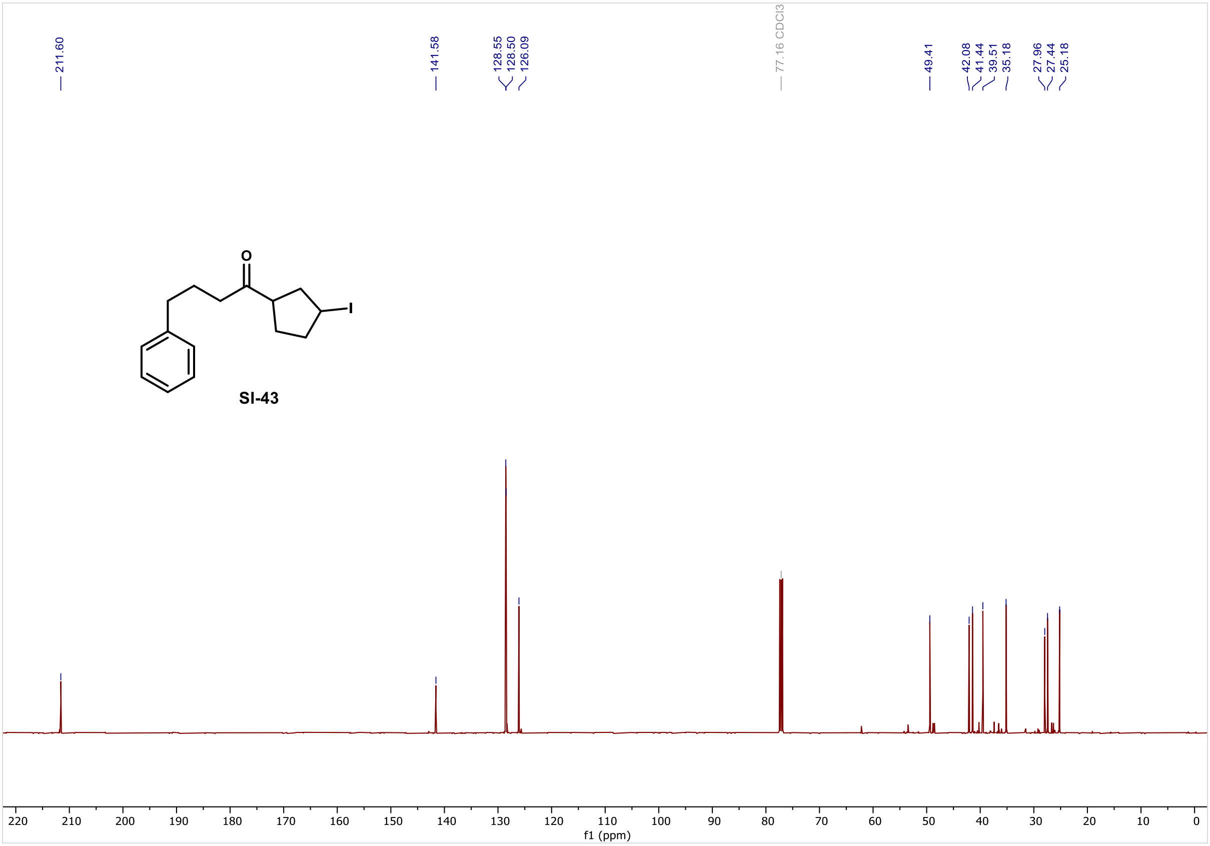

Supplement: Supplementary file 1 — Supporting Information [file ANIE-64-e202512018-s001.pdf]
